# Photoredox-catalyzed diastereoselective dearomative prenylation and reverse-prenylation of electron-deficient indole derivatives

**DOI:** 10.1038/s41467-023-39633-9

**Published:** 2023-06-30

**Authors:** Xuexue Chang, Fangqing Zhang, Shibo Zhu, Zhuang Yang, Xiaoming Feng, Yangbin Liu

**Affiliations:** 1 Institute of Chemical Biology, Shenzhen Bay Laboratory, Shenzhen, 518132 China; 2 State Key Laboratory of Biotherapy and Cancer Center, National Clinical Research Center for Geriatrics, West China Hospital of Sichuan University, Chengdu, 610041 China; 3 Key Laboratory of Green Chemistry & Technology, Ministry of Education, College of Chemistry, Sichuan University, Chengdu, 610064 China

**Keywords:** Photocatalysis, Synthetic chemistry methodology

## Abstract

Prenylated and reverse-prenylated indolines are privileged scaffolds in numerous naturally occurring indole alkaloids with a broad spectrum of important biological properties. Development of straightforward and stereoselective methods to enable the synthesis of structurally diverse prenylated and reverse-prenylated indoline derivatives is highly desirable and challenging. In this context, the most direct approaches to achieve this goal generally rely on transition-metal-catalyzed dearomative allylic alkylation of electron-rich indoles. However, the electron-deficient indoles are much less explored, probably due to their diminished nucleophilicity. Herein, a photoredox-catalyzed tandem Giese radical addition/Ireland–Claisen rearrangement is disclosed. Diastereoselective dearomative prenylation and reverse-prenylation of electron-deficient indoles proceed smoothly under mild conditions. An array of tertiary α-silylamines as radical precursors is readily incorporated in 2,3-disubstituted indolines with high functional compatibility and excellent diastereoselectivity (>20:1 d.r.). The corresponding transformations of the secondary α-silylamines provide the biologically important lactam-fused indolines in one-pot synthesis. Subsequently, a plausible photoredox pathway is proposed based on control experiments. The preliminary bioactivity study reveals a potential anticancer property of these structurally appealing indolines.

## Abstract (web summary)

Prenylated and reverse-prenylated indoline alkaloids are widely present in nature. Here, the authors demonstrate a tandem photoredox radical dearomatization/Claisen rearrangement of electron-deficient indoles for the rapid assembly of these privileged scaffolds.

## Introduction

**Fig. 1:**
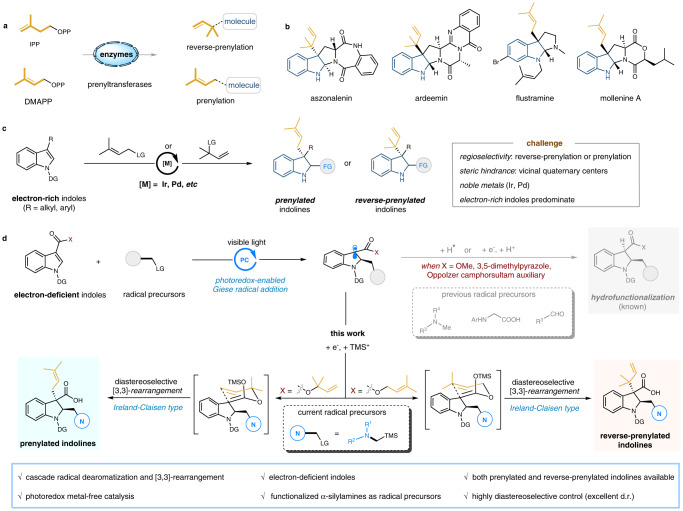
Synthesis of prenylated and reverse-prenylated indoline scaffolds. **a** Enzyme-catalyzed (reverse-)prenylation of complex molecules with isopentenyl pyrophosphate (IPP) or dimethylallyl pyrophosphate (DMAPP). **b** Representative naturally occurring prenylated and reverse-prenylated indoline products. **c** Previous work, transition metals-catalyzed allylic substituent reactions of electron-rich indoles. **d** This work, our designed strategy for the diastereoselective dearomative prenylation and reverse-prenylation of electron-deficient indoles via photocatalytic tandem Giese radical addition/Ireland–Claisen rearrangement. FG functional group, DG directing group, LG leaving group, PC photocatalyst, TMS trimethylsilyl.

In living organisms, dimethylallyl pyrophosphate (DMAPP) and isopentenyl pyrophosphate (IPP) as the precursors are usually utilized via enzyme catalysis to selectively introduce prenyl and reverse-prenyl motifs into various biological primary and secondary metabolites (Fig. 1a)^1–4^. As such, the post-translational proteins modified by a prenyl moiety are targeted to the correct membrane position and play a significant role in the biological signal transduction processes^5–7^. For small molecule metabolites, C3-prenylated and reverse-prenylated indoline scaffolds are frequently found in a variety of natural bioactive products^8–14^, such as aszonalenin and flustramine, exhibiting a series of anticancer, antibacterial, and antifungal properties (Fig. 1b). Intrigued by their wide-ranging spectrum of biological activities, the construction of these dimethylallyl-related indoline derivatives has attracted intensive attention from synthetic chemists^15–29^.

Obviously, it is a formidable task bearing several key challenges to the design of synthetic strategies to access the prenylated and reverse-prenylated indolines: 1) unsymmetrical reaction sites of the dimethyl allyl groups often lead to a prominent regioselectivity problem with competitive prenylation (3,3-dimethylallyl) or reverse-prenylation (1,1-dimethylallyl); 2) the synthesis of C3-reverse-prenylated indolines is a more difficult process with relatively low reactivity, since two vicinal all-carbon quaternary centers present substantial steric hindrance; 3) to control the diastereoselectivity at the C3 and C2 sites of indoline also faces significant obstacles. In principle, catalytic dearomative prenylation of readily available indoles via allylic substituent reactions has been recognized as one of the most straightforward methods to access the dimethylallyl-related indolines in a single step (Fig. 1c)^30–35^. However, these corresponding transformations usually depend on the use of electron-rich indoles via an intramolecular dearomative process, and the precious metals (Ir and Pd) are often necessary. By contrast, the electron-deficient indoles are rarely employed as nucleophiles in the realm of allylic alkylation reactions, owing to the mismatched electrical properties. In addition, comparing with numerous advancements in the dearomatization of electron-rich indoles^36–40^, the available dearomative reactions of electron-deficient indoles are much limited^41^. Therefore, it is distinctively challenging to achieve intermolecular dearomatization and prenylation of electron-deficient indoles simultaneously.

Recently, photoredox-enabled Giese-type radical addition^42–47^ has been implemented to the dearomatization of electron-deficient indoles. In general, diverse radical precursors, such as tertiary amines, *N*-arylglycines, aliphatic carboxylic acids and aldehydes, are initialized by the excited photocatalysts, which undergo a radical nucleophilic addition to the electron poor C2 = C3 bond of indoles. The resulting dearomatized radicals are then reduced to give the corresponding anions, followed by a rapid protonation to deliver the hydrofunctionalized indoline derivatives (Fig. 1d, top)^48–56^. Inspired by our continuing interest in sigmatropic rearrangements^57–60^, we envisage whether the incorporation of photoredox-enabled nucleophilic radical dearomatization of indoles and [3,3]-rearrangement (Ireland–Claisen type)^61–63^ could be developed to prepare the prenylated and reverse-prenylated indolines (Fig. 1d, bottom). Forinstance, when employing 1-(3,3-dimethylallyl) indole-3-carboxylates as electrophilic substrates and α-silylamines as nucleophilic radical precursors, the generated in situ carbanions can be captured by TMS^+^, leading to the formation of highly reactive silylketene acetals. Subsequent [3,3]-rearrangement takes place under mild conditions and gives the C3-reverse-prenylated and C2-aminoalkylated indoline derivatives. Similarly, only by adjusting the 1-(1,1-dimethylallyl) substituents on electrophilic indoles, the complementary C3-prenylated indoline derivatives are obtained with ease and efficiency.

Herein, we demonstrate our efforts towards the visible-light-induced Giese radical dearomatization/Ireland–Claisen rearrangement with an organic photoredox catalyst, providing an alternative approach to accomplish the valuable prenylation and reverse-prenylation of electron-deficient indoles in good to excellent yields. An array of natural products and pharmaceuticals containing an aminoalkyl group are selectively incorporated in indoline scaffolds, displaying good tolerance of diverse functional groups. Distinct from a related work by Glorius using terminal acrylate esters as radical acceptors^64^, here the prochiral C2-position of indoles will generate a new stereocenter after the radical addition, thus resulting in a challenging diastereoselective control for the subsequent [3,3]-rearrangement. Notably, after careful selection of *N*-directing groups and organic photocatalysts, the tandem Giese addition/rearrangement process was realized in an excellent diastereoselective manner, producing *trans*−2,3-difunctionalized indolines exclusively.

## Results

### Reaction optimization

To verify our strategy, the commercially available electron-deficient indole-3-carboxylic acid was converted to the corresponding 1-(3,3-dimethylallyl) ester, followed by a *N*-protection step to readily prepare the radical acceptor **1**. Considering the property of *N*-DG moiety can greatly affect the reactivity of indoles, we introduce diverse electron-withdrawing groups (such as Boc, Ac, Cbz, and Ts) on the nitrogen to decrease the electron density of indoles, thus making the indoles more reactive for the nucleophilic radical attack. Moreover, organic amines are prevalent motifs in a great variety of natural products and pharmaceuticals, which are used as the precursors for highly valuable α-aminoalkyl radicals under visible-light photoredox catalysis^65–79^. Consequently, we selected the *N*-Boc indole-ester **1a** and *N*-methyl-*N*-((trimethylsilyl)methyl)aniline **2a** as model substrates for the optimization of conditions. The mixture was irradiated by 1 W blue LEDs at room temperature and then heated at 60 °C to promote the thermal [3,3]-rearrangement process. After screening different photocatalysts, 4CzIPN was found to be the optimal catalyst, providing the dearomative reverse-prenylation product **3a** bearing vicinal all-carbon quaternary centers in 70% yield with moderate diastereoselectivity (3:1 d.r., Table 1, entry 4 vs entries 1–3). As expected, the valuable amine-moiety can be incorporated smoothly into indolines via Giese radical addition, however, a complicated diastereoselective control problem was accompanied for the sequential rearrangement. Further extensive optimization of solvents was investigated, and only slightly improved diastereomeric ratio (4:1 d.r.) was obtained in DMF without deterioration of the reactivity (78% yield, Table 1, entry 6). Then, we turned our attention to other *N*-protected indoles **1b**–**1d**. In general, the electrophilic indoles bearing *N*-electron withdrawing groups, such as Ac, Cbz, and Ts, were suitable substrates to afford the desired products in moderate to good yields. In contrast, no reaction occurred for *N*-H and *N*-Me indoles, indicating that the initial dearomatization process of the C2 = C3 bond necessitated the activation of the *N*-EWGs. Encouragingly, when tosyl-substituted indole **1d** was employed, the reverse-prenylated indoline **3d** was furnished as a single diastereoisomer with >20:1 d.r. (Table 1, entry 12). Further optimization of the concentration of the reactants and the photocatalyst loading, we were delighted to isolate the indoline **3d** in 71% yield with exclusive diastereoselectivity (Table 1, entry 13). Of note, small amounts of the Giese-type side products (hydro-aminoalkylation of indoles) were identified by ^1^H NMR analysis, probably due to the inevitable water in the reaction system. Additionally, confirmed by the control experiments, no reaction was observed in the absence of photocatalyst or light, indicating that a visible light mediated pathway was definitely involved (Table 1, entries 14 and 15).

**Table 1:** Reaction optimization for dearomative reverse-prenylation of indoles


entry^a^	substrate	PC	solvent	yield (%)^b^	d.r.^c^
1	**1a**	Ru(bpy)_3_(PF_6_)_2_	CH_3_CN	60	2.8:1
2	**1a**	Ir(bpy)_2_(dtbbpy)PF_6_	CH_3_CN	76	2.5:1
3	**1a**	Mes-Acr^+^PF_6_^-^	CH_3_CN	trace	--
4	**1a**	4CzIPN	CH_3_CN	70	3:1
5^d^	**1a**	4CzIPN	THF	75	3:1
6	**1a**	4CzIPN	DMF	78	4:1
7	**1a**	4CzIPN	DMSO	80	3:1
8	**1a**	4CzIPN	EtOAc	24	6:1
9	**1a**	4CzIPN	toluene	trace	--
10	**1b**	4CzIPN	DMF	80	2.4:1
11	**1c**	4CzIPN	DMF	30	2:1
12	**1d**	4CzIPN	DMF	65	>20:1
13^e^	**1d**	4CzIPN	DMF	71^f^	>20:1
14^g^	**1d**	**--**	DMF	0	N.D.
15^h^	**1d**	4CzIPN	DMF	0	N.D.

^a^ Reaction conditions: indoles **1** (0.1 mmol), α-silylamine **2a** (0.12 mmol), and photocatalyst (2.5 mol%) were performed in the indicated solvent (1.0 mL) at room temperature under the irradiation by 1 W blue LEDs for 2 h in argon. Then, the reaction vial was warmed at 60 °C for additional 3 h without blue LEDs.

^b^ Yield determined by ^1^H NMR with pyridine as the internal standard.

^c^ Diastereomeric ratio determined by ^1^H NMR analysis of the crude reaction mixture.

^d ^Irradiation time was extended to 48 h.

^e^ Indoles **1d** (0.3 mmol), α-silylamine **2a** (0.36 mmol), 4CzIPN (1.3 mol%), DMF (1.0 mL).

^f^ Isolated yield.

^g ^No photocatalyst.

^h^ Under dark. PC, photocatalyst; N.D., not determined.

### Access to reverse-prenylated indolines

**Fig. 2:**
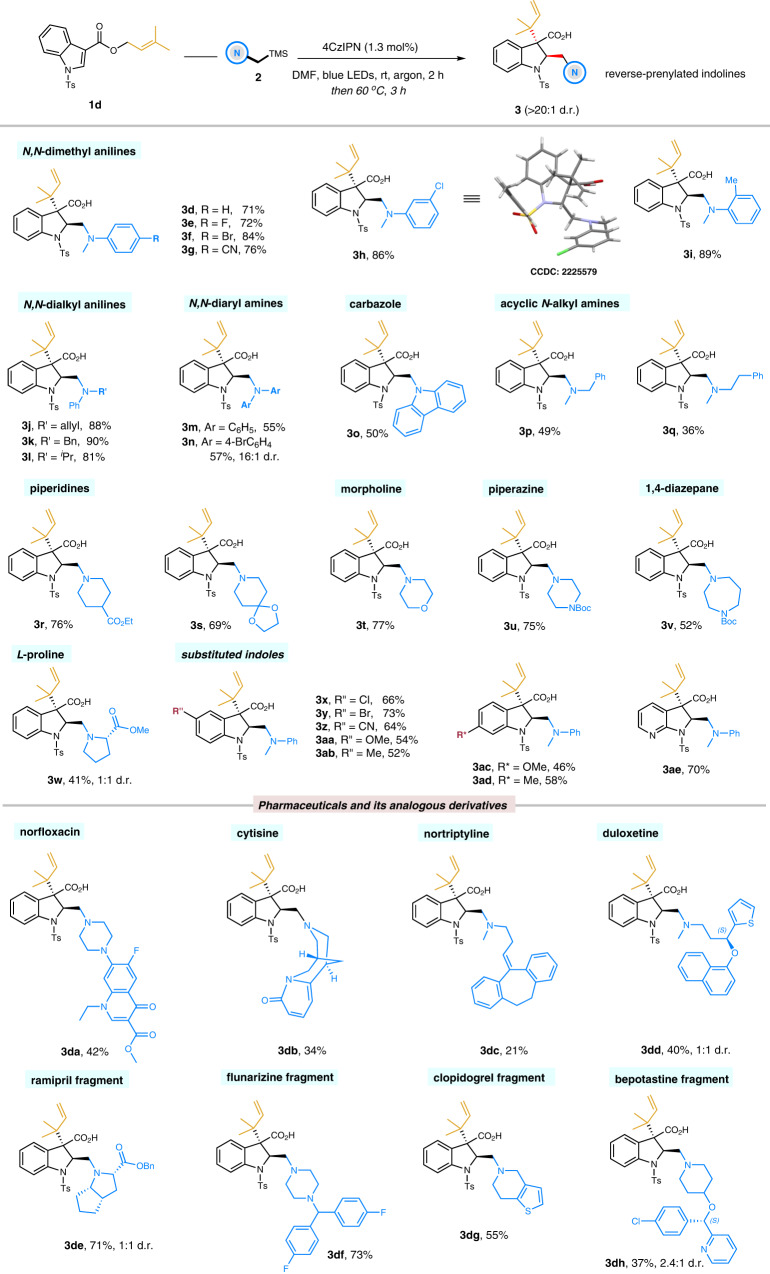
Substrate scope of reverse-prenylated indolines. Reaction conditions: solution of **1d** (0.3 mmol), **2** (0.36 mmol), and 4CzIPN (1.3 mol%) in DMF (1.0 mL) was irradiated by 1 W blue LEDs for 2 h in argon. Then the reaction vial was warmed at 60 °C for additional 3 h without blue LEDs. Isolated yields are shown. d.r. values were based on ^1^H NMR analysis.

With the optimized conditions in hand, we began to explore the generality of this dearomative reverse-prenylation with respect to electrophilic 1-(3,3-dimethylallyl) indole-3-carbonate **1d** and a range of functionalized tertiary α-silylamines **2** (Fig. 2). First, structurally diversified *N,N*-dimethyl(alkyl) anilines and *N,N*-diaryl amines were well tolerated to give the corresponding reverse-prenylated indolines (**3d–3n**) in good yields with excellent diastereoselectivities (55–90% yields, >20:1 d.r.). Electron-withdrawing and electron-donating substituents on varied positions of the aryl moiety were found to be compatible, including halogens, cyano, and methyl. The structure of reverse-prenylated indoline was determined by X-ray crystallographic analysis (**3h**, CCDC 2225579), demonstrating excellent *trans*-selectivity for the aminoalkyl and reverse-prenyl. Additionally, aniline derivatives bearing allyl, benzyl, and isopropyl groups exhibited exclusive terminal-methyl regioselectivity, as observed in **3j**–**3l**. Carbazole as an important core structure found in numerous natural compounds was also introduced into this catalytic system and showed modest reactivity (**3o**, 50% yield). We also investigated the acyclic aliphatic amines: *N*-methylbenzyl amine and exchange of the benzyl moiety with phenethyl smoothly afforded the desired 2,3-disubstituted indolines **3p** and **3q**. Subsequently, the cyclic aliphatic amines as the most frequent *N*-heterocyclic functions employed in drugs were examined. As such, substituted piperidines with varying functionalities (**3r** and **3s**) demonstrated good yields in 76% and 69%, respectively. Other relevant cyclic amines bearing additional heteroatoms and functionalities, including morpholine, piperazine, 1,4-diazepane, and L-proline, also underwent dearomatization smoothly and furnished the rearranged products **3t**–**3w** with analogous efficiencies. Substituted indoles possessing various functional groups on varied positions were also suitable substrates to deliver the reverse-prenylated indolines in good yields with excellent diastereoselectivities (46–73% yields, >20:1 d.r., **3x–3ae**). Given the tertiary amines as a common motif in pharmaceuticals, we were triggered to explore the capacity of this dearomative reverse-prenylation manifold on a range of pharmaceutically relevant compounds. As such, structurally complex drugs, including antibacterial infection norfloxacin (**3da**), acetylcholine receptor agonist cytisine (**3db**), calcium-channel blocker nortriptyline (**3dc**), and serotonin reuptake inhibitor duloxetine (**3dd**), were readily and selectively cross-coupled with indolines by the standard protocol. Alternatively, the significant amino-fragments found in other complex drugs such as ramipril (**3de**), flunarizine (**3df**), clopidogrel (**3dg**), and bepotastine (**3dh**), also afforded the corresponding indoline-adducts in good yields. Collectively, our catalytic platform provides a streamlined synthesis of structurally diverse reverse-prenylated indoline derivatives bearing a series of high-value-added amino-functionalities.

### Access to lactam-fused indolines

**Fig. 3:**
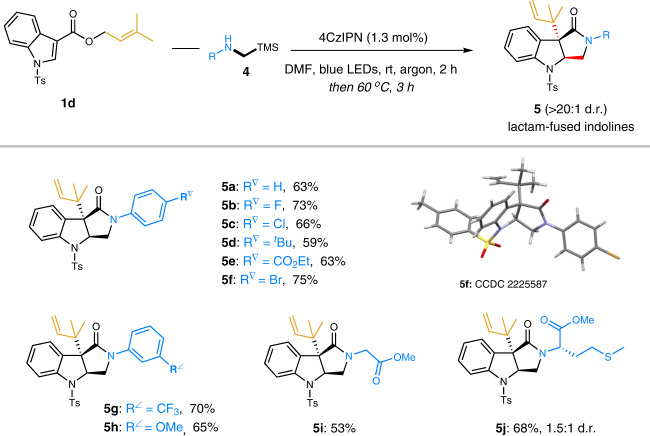
Substrate scope of the lactam-fused indolines from various secondary α-silylamines. Reaction conditions: solution of **1d** (0.3 mmol), **4** (0.36 mmol), and 4CzIPN (1.3 mol%) in DMF (1.0 mL) was irradiated by 1 W blue LEDs for 2 h in argon. Then the reaction vial was warmed at 60 °C for additional 3 h without blue LEDs. Isolated yields are shown. d.r. values were based on ^1^H NMR analysis.

We next investigated secondary α-silylamines bearing a free NH group as radical donors under optimal conditions (Fig. 3). Surprisingly, the unexpected lactam-fused indolines became the major products with excellent diastereoselectivities. We speculate that the acidic TMS^+^ in the reaction mixture may activate the indoline-3-carboxylic acid and facilitate the intramolecular amidation. Given the distinct advantage of lactam scaffolds in pharmaceuticals^80, 81^, we examined the generality of this one-pot synthesis of lactam-fused indolines. Good reactivity for a variety of secondary α-silylamines with diverse functional groups was displayed, including halogens, *tert*-butyl, ester, trifluoromethyl, and methoxyl (**5a–5h**). Also, the three-dimensional structure of compound **5f** was confirmed by X-ray diffraction analysis (CCDC 2225587). Furthermore, amino acids derivatives, glycine and L-methionine, were also applied to deliver the corresponding lactam-fused indolines **5i** and **5j** in good yields. Product **5j** was provided as two diastereoisomers due to the presence of an extra stereocenter.

### Access to prenylated indolines

**Fig. 4:**
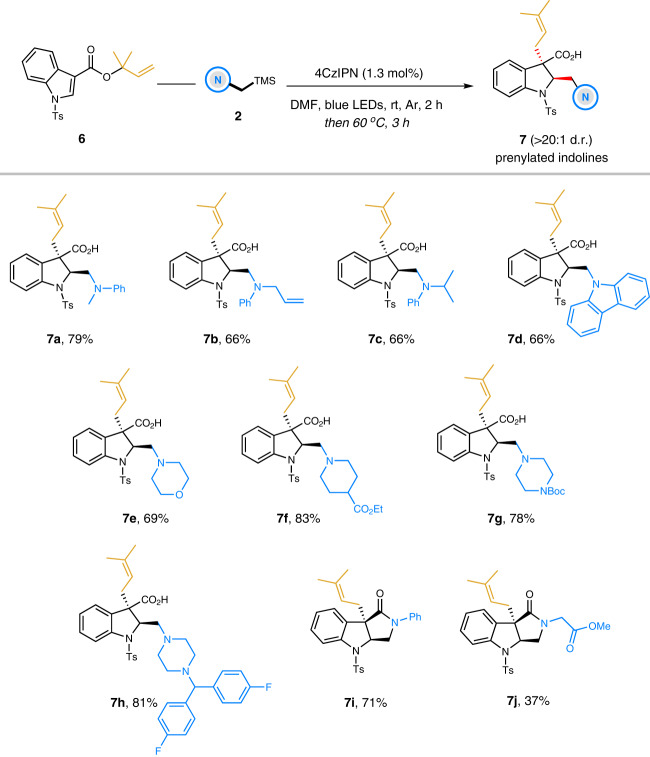
Substrate scope of prenylated indolines. Reaction conditions: solution of **6** (0.3 mmol), **2** (0.36 mmol), and 4CzIPN (1.3 mol%) in DMF (1.0 mL) was irradiated by 1 W blue LEDs for 2 h in argon. Then the reaction vial was warmed at 60  °C for additional 3 h without blue LEDs. Isolated yields are shown.

Next, we wondered if the complementary regioisomers, prenylated indolines, could be prepared via this uniform dearomatization-rearrangement sequence (Fig. 4). Delightfully, by simply changing the ester group at the C3 position of indoles into 1-(1,1-dimethylallyl) substituent (**6**), the expected transformations proceeded smoothly and afforded the prenylated indolines with good yields and excellent diastereoselectivities (**7a**–**7j**). A wide of tertiary α-silylamines bearing various functionalities and diverse structures, such as *N*,*N*-dialkyl anilines, carbazole, morpholine, piperidine and piperazines, were well tolerated and their corresponding prenylated indolines **7a**–**7h** were obtained in good to excellent yields (66–83%) with exclusive selectivity (> 20:1 d.r.). Similarly, when using secondary α-silylamines as the radical precursors, the lactam-fused indoline analogues **7i** and **7j** containing a C3-prenyl substituent were achieved with comparable results.

### Access to *trans*−2,3-disubstituted indolines

**Fig. 5:**
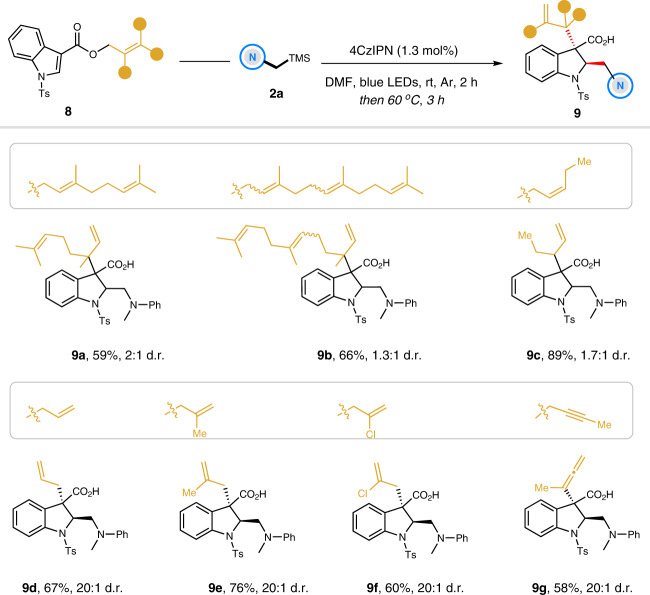
Preparation of other relevant trans-2,3-disubstituted indolines. Reaction conditions: solution of **8** (0.3 mmol), **2a** (0.36 mmol), and 4CzIPN (1.3 mol%) in DMF (1.0 mL) was irradiated by 1 W blue LEDs for 2 h in argon. Then the reaction vial was warmed at 60 °C for additional 3 h without blue LEDs. Isolated yields are shown. d.r. values were based on ^1^H NMR analysis.

In addition, other complex terpene-derived indole-3-carboxylates were found to be suitable substrates for this reaction (Fig. 5). As such, the photoredox-enabled dearomative reverse-geranylation/farnesylation proceeded smoothly under the standard conditions, giving the corresponding products **9a** and **9b** in moderate yields (59–66%), but with low diastereomeric ratios (2:1 and 1.3:1 d.r.). It is probably attributed to the difficulty in discriminating between the chairlike and boatlike transition states in the Ireland–Claisen rearrangement process. Also, the readily available (*Z*)-pent-2-en-1-ol derivative was investigated to afford the 2,3-disubstituted indoline **9c** with a similar level of diastereoselectivity (89% yield, 1.7:1 d.r.). Furthermore, different substituents in the allyl alcohols were also tested, such as hydrogen, methyl and chloro-groups. Gratifyingly, the corresponding products **9d**–**9f** can be obtained in good yields with exclusive diastereoselectivities. It was worthy of noting that the propargylic alcohol-derived indole was well tolerated, delivering the desired indoline **9g** with an allenyl-substituted quaternary center in good outcomes (58% yield, >20:1 d.r.).

### Synthetical application

**Fig. 6:**
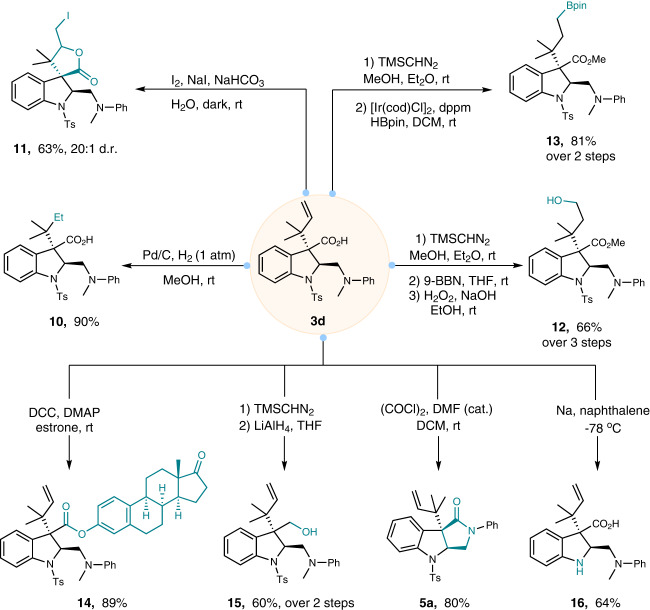
Synthetic transformations of the reverse-prenylated indolines. Conversion of the enantioenriched product **3d** to diverse chiral building blocks (for details, see Supplementary information).

To further demonstrate the synthetic potential of this mild dearomatization-rearrangement reaction of electrophilic indoles, we next explored divergent transformations of taking the reverse-prenylated products as an example (Fig. 6). Benefiting from the presence of diverse functional groups, such as terminal olefin, carboxylic acid, and amino, a variety of valuable synthetic building blocks were readily and selectively accessed after simple operations. Hydrogenation of the terminal olefin in reverse-prenyl moiety with palladium under a hydrogen atmosphere furnished **10** in 90% yield. Spiro-lactone **11** containing a primary alkyl iodide was directly prepared in 63% yield with excellent diastereoselectivity by an iodine-mediated halolactonization. Additionally, after an almost quantitative methyl-esterification, the olefin functionality was smoothly converted into primary alcohol **12** and organoboron **13** by a sequential hydroboration-oxidation (9-BBN and then H_2_O_2_), and an iridium-catalyzed hydroboration with pinacolborane, respectively. The synthetic utility of these indoline derivatives has also been shown to perform late-stage modifications of natural products to give their indoline-based analogous (for instance, estrone, **14**). The carboxylic acid group was also amenable to deliver the corresponding alcohol **15** under common conditions (reduction by LAH). Interestingly, when trying to prepare the acyl chloride from classical oxalyl chloride and DMF, an unexpected intramolecular demethylation-amidation reaction of the tertiary amine happened, resulting in the formation of lactam-fused indoline **5a** in 80% yield. Finally, deprotection of the *N*-tosyl indoline was accomplished by the single electron transfer reagent (sodium napthalenide), delivering the *N*-H free indoline derivative **16** in 64% yield.

### Mechanistic studies

**Fig. 7:**
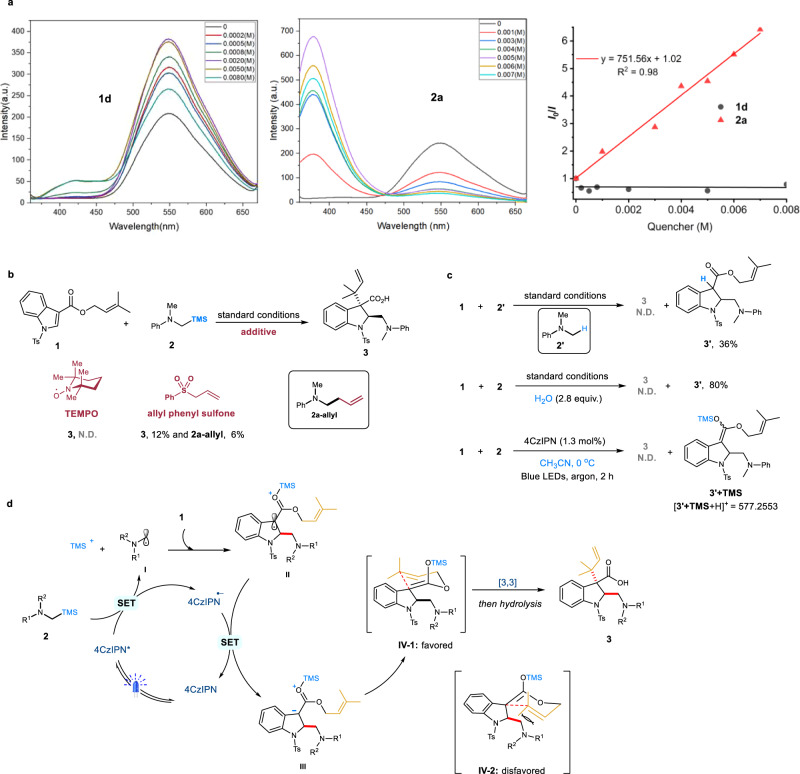
Mechanistic investigation. **a** Stern–Volmer fluorescence quenching experiments. **b** Radical trapping experiments. **c** Intermediate confirmation experiments. **d** Proposed reaction mechanism.

To gain more insight into the reaction, preliminary mechanistic studies were performed. The Stern–Volmer fluorescence quenching experiments were conducted for each component **1d** and **2a** (Fig. 7a). It was found that the excited photocatalyst 4CzIPN* was quenched significantly by the α-silylaniline **2a** rather than the indole derivative **1d**, which suggested the oxidative single-electron transfer of α-silylanilines probably triggered the photoreaction. Interestingly, the fluorescence intensity was obviously enhanced in the presence of **1d**, implying a possible interaction between the photocatalyst and the indole substrate^82^. When 2,2,6,6-tetramethylpiperidinyloxy (TEMPO) and allylic sulfoxide were used as radical-trapping reagents, the desired dearomatizaiton/rearrangement product **3** was dramatically suppressed (Fig. 7b). Moreover, the α-allylated *N*,*N*-dimethyl aniline (**2a-allyl**) was confirmed by ESI-HRMS (m/z calcd for C_11_H_15_N [M + H]^+^: 162.1277; found: 162.1278), indicating that the aminoalkyl radical might be involved in this transformation (Fig. 7b). Next, *N*,*N*-dimethyl aniline **2’** was employed as the nucleophilic radical source and exclusively gave the hydro-aminoalkylated product **3’** (Giese-type) in 36% yield, which might be attributed to the rapid protonation of the C3-benzylic anion (Fig. 7c, first). Similarly, once external H_2_O as a proton source was introduced into the standard reaction, the Giese-type side product **3’** became dominated and the [3,3]-rearrangement process was completely interrupted, further evidencing the generation of a carbanion in this reaction (Fig. 7c, second). To probe the reactive silylketene acetal intermediate, we modified the reaction conditions by switching DMF to CH_3_CN and reducing room temperature to 0 °C. In that case, the spontaneously thermal [3,3]-rearrangement became negligible, and the ESI-HRMS signal of **3’** + **TMS** (m/z calcd for C_32_H_40_N_2_O_4_SSi [M + H]^+^: 577.2551; found: 577.2553) was clearly observed (Fig. 7c, third). Accordingly, on the basis of the above control experiments along with previous works^48–52^, a plausible reaction mechanism was proposed (Fig. 7d): firstly, the excited 4CzIPN* (*E*_1/2_(PC^*^/PC^•^^-^) = +1.35 V vs. SCE in MeCN)^83^ underwent a SET oxidation of the α-silylaniline **2** (*E*_1/2_(**2a**^•^^+^/**2a**) = approx. +0.7 V vs. SCE in MeCN)^84^, followed by fragmentation to afford α-aminoalkyl radical **I** and TMS^+^. Next, the nucleophilic radical **I** attacked *N*-Ts indole derivative **1** in a similar Giese reaction pathway to generate C3-benzylic radical **II**, which was then reduced by the 4CzIPN^−^ to give a C3-benzylic anion **III**. Subsequent enolization of the anion **III** provided in situ the silylketene acetal intermediate **IV**. Finally, a spontaneous Ireland–Claisen rearrangement of **IV** furnished the dearomative reverse-prenylation of indoles, to deliver the product **3** in good yield with exclusive *trans*-selectivity of the newly formed C-C bonds (red color highlighted in **3**). We speculated that the *cis*-C3-allyl migration might be blocked by the C2-aminoalkyl substituent in transition state **IV-2**.

### Preliminary study on the anticancer activity

**Fig. 8:**
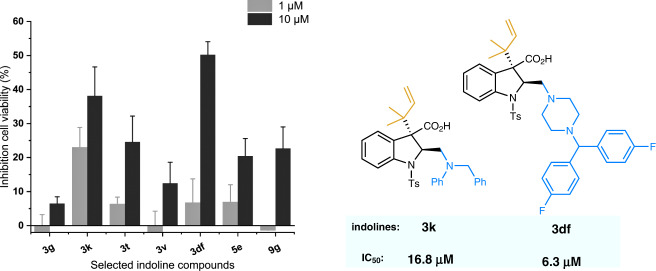
Biological activity study. Inhibition of the human leukemia cell line viability (MV4-11) induced by some selected indoline compounds. IC_50_ values for **3k** and **3df** in inhibiting MV4-11. Experiments were performed in duplicate (*n* = 3). Standard deviation (SD) values are shown as the error bars.

Considering prenylated/reverse-prenylated indolines as an important motif in bioactive natural products, we initially investigated the anticancer activity of several selected indolines by detecting the in vitro cytotoxicity against the human leukemia cell line MV4-11 (Fig. 8). It was found that most candidates had a preliminary inhibition effect at 10 μM. Notably, compounds **3k** and **3df** displayed a potential anticancer activity with IC_50_ values of 16.8 μM and 6.3 μM, respectively. We believe that further study of other indoline derivatives and biological properties is promising to discover more potential applications in medicinal chemistry.

## Discussion

In conclusion, we have developed an intermolecular dearomative prenylation and reverse-prenylation of indoles via a tandem Giese radical addition/Ireland–Claisen rearrangement. Distinct from conventional allylic substitution approaches mostly relying on electron-rich indoles, this photoredox-enabled protocol bearing transition-metal-free provides an efficient method to achieve (reverse-)prenylation of electron-deficient indoles. Moreover, after careful selection of organic photocatalysts and *N*-protection moieties, the 2,3-difunctionalized indoline derivatives were produced with exclusive diastereoselectivities (>20:1 d.r.). An array of structurally diverse amines including complex modified natural products and pharmaceuticals were employed as radical precursors, and were readily incorporated in indolines with high functional compatibility and isolated yields. Notably, by simple adjustment of 1-(1,1-dimethylallyl) or 1-(3,3-dimethylallyl) substituent in indole-3-carboxylates, both prenylated and reverse-prenylated indolines were selectively produced, respectively, without the disturbing regioselectivity issue. In addition, the current systems were found to work well with the secondary amines, efficiently affording the biologically important lactam-fused indolines in one-pot synthesis. The synthetic potential was further highlighted via diversification of reverse-prenylated products. Mechanistic studies revealed a possible photoredox-SET process, followed by the formation of silylketene acetals and subsequent [3,3]-rearrangement. Finally, the anticancer activity of these privileged indoline products was preliminarily explored, indicating potential application prospects in biological activity.

## Methods

### Materials

Unless otherwise specified, all chemicals were purchased from Leyan.com and Bide Pharmatech. All solvents were purified and dried according to standard methods before use.

### General procedure for the photoredox-catalyzed dearomative prenylation and reverse-prenylation of electron-deficient indoles

In the glovebox, to a flame-dried 8 mL reaction vial equipped with a stir bar were added dimethylallyl indole-3-carboxylate (0.3 mmol, 1.0 equiv.) and 4-CzIPN (3.0 mg, 0.0039 mmol, 0.013 equiv.) in dry DMF (0.5 mL). Then the solution of α-silylamine (0.36 mmol, 1.2 equiv.) in dry DMF (0.5 mL) was added. The vial was sealed and transferred out of the glove box. It was irradiated with a 1 W blue LED lamp (SYNLED) for 2 h at room temperature. Afterwards, the reaction mixture was allowed to heat at 60 °C for 3 h without light. When the reaction was completed (monitored by TLC), the crude mixture was quenched by water and extracted with ethyl acetate (10 mL × 2). The combined organic layers were washed with water (10 mL × 2) and brine (10 mL), dried over anhydrous Na_2_SO_4_, filtered, and concentrated by rotary evaporation. Then the residue was purified by silica gel flash chromatography to give the corresponding product.

## Supplementary information

Supplementary information

Peer Review File

## Data Availability

Crystallographic data for the structures reported in this Article have been deposited at the Cambridge Crystallographic Data Centre, under deposition numbers 2225579 (**3h**) and 2225587 (**5f**). Copies of the data can be obtained free of charge via https://www.ccdc.cam.ac.uk/structures/. All other data supporting the findings of this study are available from its Supplementary information or the corresponding author upon request.

## References

[1] Williams RM, Stocking EM, Sanz-Cervera JF (2000). Biosynthesis of prenylated alkaloids derived from tryptophan. Top. Curr. Chem..

[2] Oldfield E, Lin FY (2012). Terpene biosynthesis: modularity rules. Angew. Chem. Int. Ed..

[3] Chang W-C, Song H, Liu H-W, Liu P (2013). Current development in isoprenoid precursor biosynthesis and regulation. Curr. Opin. Chem. Biol..

[4] Tanner ME (2015). Mechanistic studies on the indole prenyltransferases. Nat. Prod. Rep..

[5] Walsh CT, Garneau-Tsodikova S, Gatto GJ (2005). Protein posttranslational modifcations: the chemistry of proteome diversifcations. Angew. Chem. Int. Ed..

[6] Palsuledesai CC, Distefano MD (2015). Protein prenylation: enzymes, therapeutics, and biotechnology applications. ACS Chem. Biol..

[7] Jeong A (2022). In vivo prenylomic profiling in the brain of a transgenic mouse model of Alzheimer’s disease reveals increased prenylation of a key set of proteins. ACS Chem. Biol..

[8] Wang HJ, Gloer JB, Dowd PF (1998). Mollenines A and B: new dioxomorpholines from the Ascostromata of Eupenicillium molle. J. Nat. Prod..

[9] Yin WB, Grundmann A, Cheng J, Li SM (2009). Acetylaszonalenin biosynthesis in Neosartorya fischeri. Identification of the biosynthetic gene cluster by genomic mining and functional proof of the genes by biochemical investigation. J. Biol. Chem..

[10] Li SM (2010). Prenylated indole derivatives from fungi: structure diversity, biological activities, biosynthesis and chemoenzymatic synthesis. Nat. Prod. Rep..

[11] Eamvijarn A, Gomes NM, Dethoup T, Roussis V, Kijjoa A (2013). Bioactive meroditerpenes and indole alkaloids from the soil fungus Neosartorya fischeri (KUFC 6344), and the marine-derived fungi Neosartorya laciniosa (KUFC 7896) and Neosartorya tsunodae (KUFC 9213). Tetrahedron.

[12] Melander RJ, Basak AK, Melander C (2020). Natural products as inspiration for the development of bacterial antibiofilm agents. Nat. Prod. Rep..

[13] Gu L, Sun FJ, Yang MH, Kong LY (2021). Ardeemins and citrinin dimer derivatives from Aspergillus terreus harbored in Pinellia ternate. Phytochem. Lett..

[14] Liu Y, Chen M-Q, Liu Y-Y, Zhang Y, Qian Z-J (2022). Mechanism of two alkaloids isolated from coral endophytic fungus for suppressing angiogenesis in atherosclerotic plaque in HUVEC. Int. Immunopharmacol..

[15] Lindel T, Marsch N, Adla SK (2012). Indole prenylation in alkaloid synthesis. Top. Curr. Chem..

[16] Hu Y-C, Min X-T, Ji D-W, Chen Q-A (2022). Catalytic prenylation and reverse prenylation of aromatics. Trends Chem..

[17] Austin JF, Kim S-G, Sinz CJ, Xiao W-J, MacMillan DWC (2004). Enantioselective organocatalytic construction of pyrroloindolines by a cascade addition–cyclization strategy: synthesis of (-)-fustramine B. Proc. Natl Acad. Sci. USA.

[18] Trost BM, Stiles DT (2007). Total synthesis of spirotryprostatin B via diastereoselective prenylation. Org. Lett..

[19] Itoh J, Han SB, Krische MJ (2009). Enantioselective allylation, crotylation, and reverse prenylation of substituted isatins: iridium-catalyzed C-C bond-forming transfer hydrogenation. Angew. Chem. Int. Ed..

[20] Trost BM, Malhotra S, Chan WH (2011). Exercising regiocontrol in palladium-catalyzed asymmetric prenylations and geranylation: unifying strategy toward flustramines A and B. J. Am. Chem. Soc..

[21] Zhang X, Han L, You S-L (2014). Ir-catalyzed intermolecular asymmetric allylic dearomatization reaction of indoles. Chem. Sci..

[22] Trost BM, Chan WH, Malhotra S (2017). Development of the regiodivergent asymmetric prenylation of 3-substituted oxindoles. Chem. Eur. J..

[23] Hakamata H, Sato S, Ueda H, Tokuyama H (2017). AgNTf_2_-mediated allylation with allylsilanes at C3a-position of hexahydropyrroloindoles: application to total syntheses of amauromine alkaloids. Org. Lett..

[24] Li DF, Liu K, Zhang JR, Zhao LM (2018). Access to 3-prenylated oxindoles by α-regioselective prenylation: application to the synthesis of (+/-)-debromoflustramine E. Org. Lett..

[25] Trost BM, Bai W-J, Hohn C, Bai Y, Cregg JJ (2018). Palladium-catalyzed asymmetric allylic alkylation of 3-substituted 1*H*-indoles and tryptophan derivatives with vinylcyclopropanes. J. Am. Chem. Soc..

[26] Hu Y-C, Ji D-W, Zhao C-Y, Zheng H, Chen Q-A (2019). Catalytic prenylation and reverse prenylation of indoles with isoprene: regioselectivity manipulation through choice of metal hydride. Angew. Chem. Int. Ed..

[27] Yu H, Zong Y, Xu T (2020). Total synthesis of (−)-penicimutanin a and related congeners. Chem. Sci..

[28] Zhao C-Y (2022). Bioinspired and ligand-regulated unnatural prenylation and geranylation of oxindoles with isoprene under Pd catalysis. Angew. Chem. Int. Ed..

[29] García-Domínguez P, de Lera AR (2022). Puzzling out the structure of novofumigatamide: total synthesis of constitutional isomers. Part II. J. Org. Chem..

[30] Kimura M, Futamata M, Mukai R, Tamaru Y (2005). Pd-catalyzed C3-selective allylation of indoles with allyl alcohols promoted by triethylborane. J. Am. Chem. Soc..

[31] Ruchti J, Carreira EM (2014). Ir-catalyzed reverse prenylation of 3-substituted indoles: total synthesis of (+)-aszonalenin and (-)-brevicompanine B. J. Am. Chem. Soc..

[32] Muller JM, Stark CB (2016). Diastereodivergent reverse prenylation of indole and tryptophan derivatives: total synthesis of amauromine, novoamauromine, and epi-amauromine. Angew. Chem. Int. Ed..

[33] Tu H-F, Zhang X, Zheng C, Zhu M, You S-L (2018). Enantioselective dearomative prenylation of indole derivatives. Nat. Catal..

[34] Khopade TM, Ajayan K, Joshi SS, Lane AL, Viswanathan R (2021). Bioinspired Brønsted acid-promoted regioselective tryptophan isoprenylations. ACS Omega.

[35] Joshi BD, Chisholm JD (2021). Formation of pyrroloindolines via the alkylation of tryptamines with trichloroacetimidates. Tetrahedron Lett..

[36] Repka LM, Reisman SE (2013). Recent developments in the catalytic, asymmetric construction of pyrroloindolines bearing all-carbon quaternary stereocenters. J. Org. Chem..

[37] Zhuo C-X, Zheng C, You S-L (2014). Transition-metal-catalyzed asymmetric allylic dearomatization reactions. Acc. Chem. Res..

[38] Roche SP, Youte Tendoung J-J, Treguier B (2015). Advances in dearomatization strategies of indoles. Tetrahedron.

[39] Zheng C, You S-L (2019). Catalytic asymmetric dearomatization (CADA) reaction-enabled total synthesis of indole-based natural products. Nat. Prod. Rep..

[40] Sheng F-T, Wang J-Y, Tan W, Zhang Y-C, Shi F (2020). Progresses in organocatalytic asymmetric dearomatization reactions of indole derivatives. Org. Chem. Front..

[41] Cerveri A, Bandini M (2020). Recent advances in the catalytic functionalization of “electrophilic” indoles. Chin. J. Chem..

[42] Giese B (1983). Formation of C-C bonds by addition of free radicals to alkenes. Angew. Chem. Int. Ed. Engl..

[43] Qin T (2017). Nickel catalyzed Barton decarboxylation and Giese reactions: a practical take on classic transforms. Angew. Chem. Int. Ed..

[44] ElMarrouni A, Ritts CB, Balsells J (2018). Silyl-mediated photoredox-catalyzed Giese reaction: addition of non-activated alkyl bromides. Chem. Sci..

[45] Liu H (2018). One-pot photomediated Giese reaction/Friedel–Crafts hydroxyalkylation/oxidative aromatization to access naphthalene derivatives from toluenes and enones. ACS Catal..

[46] Kanegusuku ALG, Castanheiro T, Ayer SK, Roizen JL (2019). Sulfamyl radicals direct photoredox-mediated Giese reactions at unactivated C(3)–H bonds. Org. Lett..

[47] Cheng Y-Z (2020). Intermolecular dearomatization of naphthalene derivatives by photoredox-catalyzed 1,2-hydroalkylation. Angew. Chem. Int. Ed..

[48] Huang X-L, Cheng Y-Z, Zhang X, You S-L (2020). Photoredox-catalyzed intermolecular hydroalkylative dearomatization of electron-deficient indole derivatives. Org. Lett..

[49] Zhang YT, Ji P, Guo F, Zeng FX, Wang W (2021). Photoredox asymmetric nucleophilic dearomatization of indoles with neutral radicals. ACS Catal..

[50] Zhang YT, Ji P, Wang CQ, Zhou ZY, Wang W (2021). Organophotocatalytic dearomatization of indoles, pyrroles and benzo(thio)furans via a Giese-type transformation. Commun. Chem..

[51] Varlet T, Bouchet D, Elslande EV, Masson G (2022). Decatungstate-photocatalyzed dearomative hydroacylation of indoles: direct synthesis of 2-acylindolines. Chem. Eur. J..

[52] Huang X-L, Cheng Y-Z, You S-L (2022). Visible-light enabled synthesis of cyclopropane-fused indolines via dearomatization of indoles. Org. Chem. Front..

[53] Zhou W-J (2020). Reductive dearomative arylcarboxylation of indoles with CO_2_ via visible-light photoredox catalysis. Nat. Commun..

[54] Chen S, Meervelt LV, der Eycken EVV, Sharma UK (2022). Visible-light-driven palladium-catalyzed radical tandem dearomatization of indoles with unactivated alkenes. Org. Lett..

[55] Chen S (2022). Visible-light-induced cascade difunctionalization of indoles enabled by the synergy of photoredox and photoexcited ketones: direct access to alkylated pyrrolophenanthridones. Org. Lett..

[56] Cai Y-P, Ma M-Y, Xu X, Song Q-H (2023). Visible-light-driven reductive dearomatization of *N*-arylformyl indoles in EDA complexes with a thiophenol via a HAT pathway. Org. Chem. Front..

[57] Liu YB, Liu XH, Feng XM (2022). Recent advances in metal-catalysed asymmetric sigmatropic rearrangements. Chem. Sci..

[58] Liu YB (2016). Synergistic kinetic resolution and asymmetric propargyl Claisen rearrangement for the synthesis of chiral allenes. Angew. Chem. Int. Ed..

[59] Liu Y (2022). Diastereodivergent synthesis of chiral α-aminoketones via a catalytic O–H insertion/Barnes–Claisen rearrangement reaction. ACS Catal..

[60] Wang LF (2022). [3,3]-Sigmatropic rearrangements of naphthyl 1-propargyl ethers: para-propargylation and catalytic asymmetric dearomatization. Angew. Chem. Int. Ed..

[61] Ireland RE, Mueller RH (1972). Claisen rearrangement of allyl esters. J. Am. Chem. Soc..

[62] Ireland RE, Mueller RH, Willard AK (1976). The ester enolate Claisen rearrangement. Stereochemical control through stereoselective enolate formation. J. Am. Chem. Soc..

[63] Ireland RE, Wipf P, Armstrong JD (1991). Stereochemical control in the ester enolate Claisen rearrangement. 1. Stereoselectivity in silyl ketene acetal formation. J. Org. Chem..

[64] Kleinmans R, Will LE, Schwarz JL, Glorius F (2021). Photoredox-enabled 1,2-dialkylation of alpha-substituted acrylates via Ireland–Claisen rearrangement. Chem. Sci..

[65] Nakajima K, Miyake Y, Nishibayashi Y (2016). Synthetic utilization of α‑aminoalkyl radicals and related species in visible light photoredox catalysis. Acc. Chem. Res..

[66] Leitch JA, Rossolini T, Rogova T, Maitland JAP, Dixon DJ (2020). α-Amino radicals via photocatalytic single-electron reduction of imine derivatives. ACS Catal..

[67] Shi L, Xia W (2012). Photoredox functionalization of C–H bonds adjacent to a nitrogen atom. Chem. Soc. Rev..

[68] Brumfield MA, Quillen SL, Yoon UC, Mariano PS (1984). A novel method for heteroatom-substituted free radical generation by photochemical electron transfer induced desilylation of RXCH_2_SiMe_3_ systems. J. Am. Chem. Soc..

[69] Hasegawa E, Xu W, Mariano PS, Yoon UC, Kim JU (1988). Electron-transfer-induced photoadditions of the silyl amine, Et_2_NCH_2_SiMe_3_, to.alpha.,.beta.-unsaturated cyclohexenones. Dual reaction pathways based on ion pair-selective cation-radical chemistry. J. Am. Chem. Soc..

[70] Espelt LR, McPherson, Iain S, Wiensch EM, Yoon TP (2015). Enantioselective conjugate additions of α-amino radicals via cooperative photoredox and Lewis acid catalysis. J. Am. Chem. Soc..

[71] Xie J, Yu J, Rudolph M, Rominger F, Hashmi ASK (2016). Monofluoroalkenylation of dimethylamino compounds through radical–radical cross-coupling. Angew. Chem. Int. Ed..

[72] Le C, Liang Y, Evans RW, Li X, MacMillan DWC (2017). Selective sp^3^ C–H alkylation via polarity-match-based cross-coupling. Nature.

[73] Lin S-X, Sun G-J, Kang Q (2017). A visible-light-activated rhodium complex in enantioselective conjugate addition of α-amino radicals with Michael acceptors. Chem. Commun..

[74] Lang SB, Wiles RJ, Kelly CB, Molander GA (2017). Photoredox generation of carbon-centered radicals enables the construction of 1,1-difluoroalkene carbonyl mimics. Angew. Chem. Int. Ed..

[75] Aycock RA, Pratt CJ, Jui NT (2018). Aminoalkyl radicals as powerful intermediates for the synthesis of unnatural amino acids and peptides. ACS Catal..

[76] Zheng S (2020). Selective 1,2-aryl-aminoalkylation of alkenes enabled by metallaphotoredox catalysis. Angew. Chem. Int. Ed..

[77] Leng L, Fu Y, Liu P, Ready JM (2020). Regioselective, photocatalytic α-functionalization of amines. J. Am. Chem. Soc..

[78] Walker MM (2020). Highly diastereoselective functionalization of piperidines by photoredox-catalyzed α-amino C–H arylation and epimerization. J. Am. Chem. Soc..

[79] Zheng S, Wang W, Yuan W (2022). Remote and proximal hydroaminoalkylation of alkenes enabled by photoredox/nickel dual catalysis. J. Am. Chem. Soc..

[80] Nay B, Riache N, Evanno L (2009). Chemistry and biology of non-tetramic γ-hydroxy-γ-lactams and γ-alkylidene-γ-lactams from natural sources. Nat. Prod. Rep..

[81] Caruano J, Muccioli GG, Robiette R (2016). Biologically active γ-lactams: synthesis and natural sources. Org. Biomol. Chem..

[82] Yi Y, Fan Z, Xi C (2022). Photoredox-catalyzed intermolecular dearomative trifluoromethylcarboxylation of indoles and heteroanalogues with CO_2_ and fluorinated radical precursors. Green. Chem..

[83] Luo J, Zhang J (2016). Donor−acceptor fluorophores for visible-light-promoted organic synthesis: photoredox/Ni dual catalytic C(sp^3^)–C(sp^2^) cross-coupling. ACS Catal..

[84] Remeur C, Kelly CB, Patel NR, Molander GA (2017). Aminomethylation of aryl halides using α-silylamines enabled by Ni/photoredox dual catalysis. ACS Catal..

