# Supplementary material for: Photoredox-catalyzed diastereoselective dearomative prenylation and reverse-prenylation of electron-deficient indole derivatives
Source: Nat Commun. 2023 Jun 30;14:3876. doi: 10.1038/s41467-023-39633-9 (PMC10313782; doi:10.1038/s41467-023-39633-9)
Supplement: Supplementary file 1 — Supplementary information [file 41467_2023_39633_MOESM1_ESM.pdf]

Supplementary Information

Photoredox-Catalysed Diastereoselective Dearomative Prenylation and Reverse-Prenylation of Electron-Deficient Indole Derivatives

Xuexue Chang,[1] Fangqing Zhang,[1] Shibo Zhu,[1] Zhuang Yang,[2] Xiaoming Feng,[1,3]* Yangbin Liu[1]*

[1]Institute of Chemical Biology, Shenzhen Bay Laboratory, Shenzhen 518132, China.

[2]State Key Laboratory of Biotherapy and Cancer Center, National Clinical Research Center for Geriatrics, West China Hospital of Sichuan University, Chengdu 610041, China.

[3]Key Laboratory of Green Chemistry & Technology, Ministry of Education, College of Chemistry, Sichuan University, Chengdu 610064, China.

*e-mail: xmfeng@scu.edu.cn; liuyb@szbl.ac.cn

Table of Contents

1. Supplementary notes **2**

2. Supplementary method A: synthesis of substrates **3**

3. Supplementary method B: photoredox-catalysed diastereoselective dearomative (reverse-)prenylation of electron-deficient indoles **19**

4. Supplementary method C: synthetic transformations **72**

5. Supplementary discussions **83**

5.1. Exploration of other radical precursors **83**

5.2. Mechanistic study **85**

5.3. Anticancer activity study **93**

5.4. X-Ray crystal data **94**

6. NMR spectrum **112**

7. Supplementary references **220**

1. Supplementary notes

All solvents were dried and distilled according to general practice prior to use. All reagents were purchased from commercial sources and used without further purification unless specified otherwise. Solvents for flash column chromatography were technical grade and distilled prior to use. Analytical thin-layer chromatography (TLC) was performed using Jiangyou silica gel plates with HSGF 254. Visualization of the developed chromatogram was performed by UV absorbance (254 nm) and appropriate stains. Flash column chromatography was performed using silica gel (300-400 mesh) from Leyan.com with the indicated solvent system according to standard techniques. $CDCl_3$ was bought from Leyan.com. ^{1}H NMR, ^{13}C NMR and ^{19}F NMR were recorded on Bruker NMR 400 MHz, 500 MHz, Zhongke 400 NMR. Coupling constants (*J*) are quoted in *Hz*. To describe the multiplicities of the signals, the following abbreviation were used: S (singlet), d (doublet), t (triplet), q (quartet), m (multiplet). Melting points were recorded on a Shanghai Jingke SGWX-4B melting-point Meter and are uncorrected. HRMS (ESI) analysis was performed by the Analytical Instrumentation Center at Peking University Shenzhen Graduate School and (HRMS) data were reported with ion mass/charge (m/z) ratios as values in atomic mass units.

2. Supplementary method A: synthesis of substrates

2.1 Synthesis of 3-Indole Esters

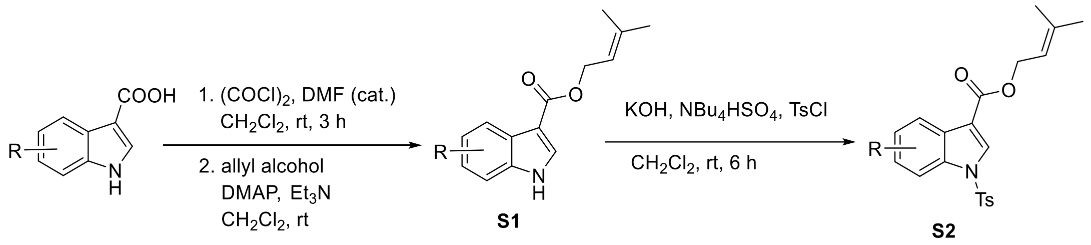

The compound indole-3-carboxylic acid was dissolved in dry CH_2Cl_2 (20 mL), and oxalyl chloride (30 mmol) was added dropwise at 0°C under the argon atmosphere. Afterwards, DMF (dry, 5 drops) was added and the reaction mixture was stirred for 3 h at room temperature. All volatiles were removed under reduced pressure and the acid chloride was subsequently used without further purification. To a solution of allyl alcohol (15 mmol), DMAP (2.5 mmol) in CH_2Cl_2 (10 ml) was added Et_3N (20 mmol), then it was cooled to 0°C. Acid chloride (10 mmol, dissolved in a minimum amount of CH_2Cl_2) was slowly added at 0°C. The resulting reaction mixture was allowed to warm up to room temperature overnight. Water was added to quench the reaction. The organic layer was washed with saturated aqueous solution of brine, dried over anhydrous Na_2SO_4, filtered and concentrated in vacuo. The residue was purified by column chromatography on silica gel (Petroleum ether / ethyl acetate = 10: 1, v/v) to afford the compound **S1**.

To a solution of **S1** in CH_2Cl_2 (10 ml) were added NBu_4HSO_4 (0.1 equiv.) and KOH (2.0 equiv.) at room temperature. After stirring for 1 h, TsCl (1.5 equiv.) was added and the reaction was stirred for 6 h. After completion of the reaction monitored by TLC, the organic layer was washed with saturated aqueous solution of brine, dried over anhydrous Na_2SO_4, filtered and concentrated in vacuo. The residue was purified by column chromatography on silica gel (Petroleum ether / ethyl acetate = 50: 1, v/v) to afford the compound **S2**.

3-methylbut-2-en-1-yl 1-tosyl-1*H*-indole-3-carboxylate (1d)

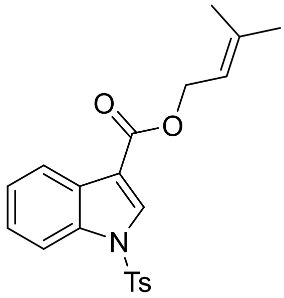

The title compound was prepared from indole-3-carboxylic acid (1.60 g, 10 mmol, 1.0 equiv.), 3-methyl-2-buten-1-ol (1.5 mL, 20 mmol, 2.0 equiv.). Product **1d** was obtained as a white solid (2.72 g, 7.1 mmol, 71% yield).

TLC: R_f = 0.4 (Petroleum ether /ethyl acetate 10:1).

^{1}H NMR (400 MHz, $CDCl_3$) δ 8.33 (s, 1H), 8.18 (dd, *J* = 6.9, 1.7 Hz, 1H), 8.00 (d, *J* = 7.3 Hz, 1H), 7.86 (d, *J* = 8.4 Hz, 2H), 7.44 – 7.33 (m, 2H), 7.28 (d, *J* = 8.2 Hz, 2H), 5.54 (t, *J* = 7.2 Hz, 1H), 4.88 (d, *J* = 7.2 Hz, 2H), 2.38 (s, 3H), 1.85 (s, 3H), 1.83 (s, 3H).

^{13}C NMR (101 MHz, $CDCl_3$) δ 163.8, 145.8, 139.3, 134.8, 134.7, 132.0, 130.2, 127.9, 127.2, 125.3, 124.4, 122.2, 118.7, 113.8, 113.3, 61.5, 25.9, 21.6, 18.2.

HRMS: m/z $[M+H]^+$ calcd for $C_{21}H_{22}NO_4S$: 384.1264; found: 384.1261.

m.p. 100 – 101 ^oC.

3-methylbut-2-en-1-yl 5-chloro-1-tosyl-1*H*-indole-3-carboxylate (1x)

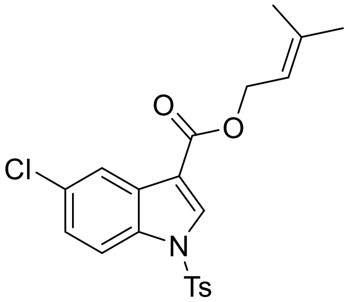

The title compound was prepared from 5-chloroindole-3-carboxylic acid (1.50 g, 10 mmol), 3-methyl-2-buten-1-ol (1.5 mL, 15 mmol, 1.5 equiv.). Product **1x** was obtained as a white solid (1.05 g, 2.50 mmol, 25% yield).

TLC: R_f = 0.4 (Petroleum ether /ethyl acetate 10:1).

^{1}H NMR (400 MHz, $CDCl_3$) δ 8.28 (s, 1H), 8.14 (d, *J* = 2.0 Hz, 1H), 7.89 (d, *J* = 8.9 Hz, 1H), 7.82 (d, *J* = 8.4 Hz, 2H), 7.35 – 7.28 (m, 3H), 5.53 – 5.45 (m, 1H), 4.85 (d, *J* = 7.2 Hz, 2H), 2.39 (s, 3H), 1.83 (s, 3H), 1.81 (s, 3H).

^{13}C NMR (101 MHz, $CDCl_3$) δ 163.3, 146.1, 139.6, 134.4, 133.2, 133.0, 130.5, 130.3, 129.1, 127.2, 125.7, 122.0, 118.5, 114.4, 113.3, 61.6, 25.8, 21.7, 18.2.

HRMS: m/z $[M+Na]^+$ calcd for $C_{21}H_{20}{}^{35}ClNNaO_4S$: 440.0694; found: 440.0694.

HRMS: m/z $[M+Na]^+$ calcd for $C_{21}H_{20}{}^{37}ClNNaO_4S$: 442.0664; found: 442.0659.

m.p. 108 – 109 ^oC.

3-methylbut-2-en-1-yl 5-bromo-1-tosyl-1*H*-indole-3-carboxylate (1y)

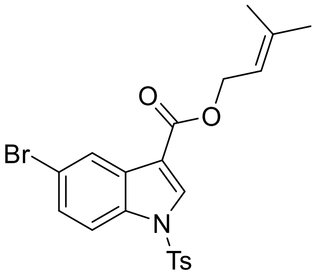

The title compound was prepared from 5-bromoindole-3-carboxylicacid (1.90 g, 8 mmol), 3-methyl-2-buten-1-ol (1.2 mL, 12 mmol, 1.5 equiv.). Product **1y** was obtained as a white solid (1.55 g, 3.36 mmol, 42% yield).

TLC: R_f = 0.4 (Petroleum ether /ethyl acetate 10:1).

^{1}H NMR (400 MHz, $CDCl_3$) δ 8.30 (d, *J* = 1.8 Hz, 1H), 8.26 (s, 1H), 7.85 – 7.79 (m, 3H), 7.46 (dd, *J* = 8.8, 1.9 Hz, 1H), 7.29 (d, *J* = 8.4 Hz, 2H), 5.49 (t, *J* = 7.2 Hz, 1H), 4.85 (d, *J* = 7.2 Hz, 2H), 2.39 (s, 3H), 1.82 (s, 3H), 1.81 (s, 3H).

^{13}C NMR (101 MHz, $CDCl_3$) δ 163.3, 146.1, 139.6, 134.4, 133.5, 132.8, 130.3, 129.5, 128.4, 127.2, 125.0, 118.5, 118.2, 114.7, 113.2, 61.7, 25.9, 21.7, 18.2.

HRMS: m/z $[M+H]^+$ calcd for $C_{21}H_{21}{}^{79}BrNO_4S$: 462.0369; found: 462.0368.

HRMS: m/z $[M+H]^+$ calcd for $C_{21}H_{21}{}^{81}BrNO_4S$: 464.0349; found: 464.0341.

m.p. 93 – 94 ^oC.

3-methylbut-2-en-1-yl 5-cyano-1-tosyl-1*H*-indole-3-carboxylate (1z)

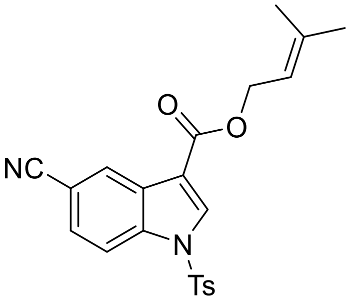

The title compound was prepared from 5-cyano-1-tosyl-1*H*-indole-3-carboxylic acid (1.86 g, 10 mmol, 1.0 equiv.), 3-methyl-2-buten-1-ol (1.5 mL, 15 mmol, 1.5 equiv.). Product **1z** was obtained as a white solid (1.23 g, 2.80 mmol, 28% yield).

TLC: R_f = 0.4 (Petroleum ether /ethyl acetate 10:1).

^{1}H NMR (400 MHz, $CDCl_3$) δ 8.48 (d, *J* = 1.6 Hz, 1H), 8.36 (s, 1H), 8.05 (d, *J* = 8.7 Hz, 1H), 7.83 (d, *J* = 8.1 Hz, 2H), 7.60 (dd, *J* = 8.6, 1.6 Hz, 1H), 7.31 (d, *J* = 8.1 Hz, 2H), 5.47 (t, *J* = 7.2 Hz, 1H), 4.85 (d, *J* = 7.2 Hz, 2H), 2.38 (s, 3H), 1.81 (s, 3H), 1.79 (s, 3H).

^{13}C NMR (101 MHz, $CDCl_3$) δ 162.9, 146.6, 140.0, 136.4, 134.1, 133.7, 130.5, 128.3, 127.9, 127.5, 127.3, 119.1, 118.2, 114.3, 113.6, 108.2, 61.9, 25.9, 21.7, 18.2.

HRMS: m/z $[M+H]^+$ calcd for $C_{22}H_{21}N_2O_4S$: 409.1217; found: 409.1216.

m.p. 127 – 128 ^oC.

3-methylbut-2-en-1-yl 5-methoxy-1-tosyl-1*H*-indole-3-carboxylate (1aa)

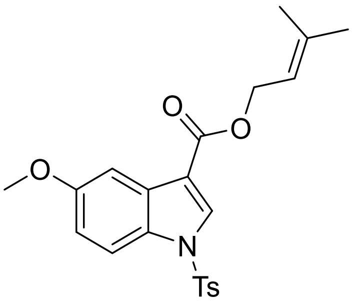

The title compound was prepared from 5-methoxy-1-tosyl-1*H*-indole-3-carboxylic acid (1.91 g, 10 mmol, 1.0 equiv.), 3-methyl-2-buten-1-ol (1.5 mL, 15 mmol, 1.5 equiv.). Product **1aa** was obtained as a white solid (1.54 g, 3.72 mmol, 37% yield).

TLC: R_f = 0.4 (Petroleum ether /ethyl acetate 10:1).

^{1}H NMR (400 MHz, $CDCl_3$) δ 8.21 (s, 1H), 7.83 (d, *J* = 9.1 Hz, 1H), 7.79 (d, *J* = 8.4 Hz, 2H), 7.61 (d, *J* = 2.6 Hz, 1H), 7.26 – 7.22 (m, 2H), 6.96 (dd, *J* = 9.1, 2.6 Hz, 1H), 5.49 (t, *J* = 7.2 Hz, 1H), 4.82 (d, *J* = 7.2 Hz, 2H), 3.83 (s, 3H), 2.35 (s, 3H), 1.80 (s, 3H), 1.78 (s, 3H).

^{13}C NMR (101 MHz, $CDCl_3$) δ 163.9, 157.2, 145.7, 139.4, 134.7, 132.3, 130.2, 129.4, 129.1, 127.1, 118.7, 115.1, 114.2, 113.5, 103.8, 61.4, 55.6, 25.8, 21.6, 18.2.

HRMS: m/z $[M+H]^+$ calcd for $C_{22}H_{24}NO_5S$: 414.1370; found: 414.1370.

m.p. 98 – 99 °C.

3-methylbut-2-en-1-yl 5-methyl-1-tosyl-1*H*-indole-3-carboxylate (1ab)

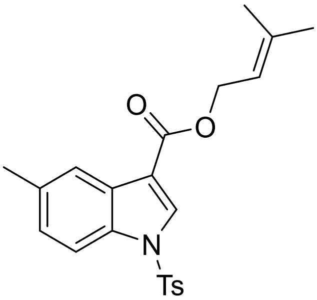

The title compound was prepared from 5-methyl-1-tosyl-1*H*-indole-3-carboxylic acid (1.72 g, 10 mmol, 1.0 equiv.), 3-methyl-2-buten-1-ol (1.5 mL, 15 mmol, 1.5 equiv.). Product **1ab** was obtained as a white solid (749 mg, 1.89 mmol, 19% yield).

TLC: R_f = 0.4 (Petroleum ether /ethyl acetate 10:1).

^{1}H NMR (400 MHz, $CDCl_3$) δ 8.23 (s, 1H), 7.94 (s, 1H), 7.84 – 7.78 (m, 3H), 7.24 (d, *J* = 8.1 Hz, 2H), 7.17 (d, *J* = 7.7 Hz, 1H), 5.49 (t, *J* = 7.2 Hz, 1H), 4.83 (d, *J* = 7.2 Hz, 2H), 2.43 (s, 3H), 2.34 (s, 3H), 1.81 (s, 3H), 1.79 (s, 3H).

^{13}C NMR (101 MHz, $CDCl_3$) δ 163.9, 145.7, 139.2, 134.7, 134.2, 133.1, 132.0, 130.1, 128.1, 127.1, 126.8, 122.0, 118.7, 113.5, 113.0, 61.4, 25.9, 21.6, 21.5, 18.2.

HRMS: m/z $[M+H]^+$ calcd for $C_{22}H_{24}NO_4S$: 398.1421; found: 398.1426.

m.p. 93 – 94 °C.

3-methylbut-2-en-1-yl 6-methoxy-1-tosyl-1*H*-indole-3-carboxylate (1ac)

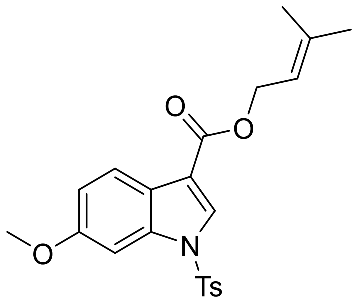

The title compound was prepared from 6-methoxy-1-tosyl-1*H*-indole-3-carboxylic acid (1.91 g, 10 mmol, 1.0 equiv.), 3-methyl-2-buten-1-ol (1.5 mL, 15 mmol, 1.5 equiv.). Product **1ac** was obtained as a white solid (1.55 g, 3.75 mmol, 38% yield).

TLC: R_f = 0.4 (Petroleum ether /ethyl acetate 10:1).

^{1}H NMR (400 MHz, $CDCl_3$) δ 8.19 (s, 1H), 8.02 (d, *J* = 8.8 Hz, 1H), 7.84 (d, *J* = 8.4 Hz, 2H), 7.50 (d, *J* = 2.3 Hz, 1H), 7.31 (s, 1H), 7.29 (s, 1H), 6.99 (dd, *J* = 8.8, 2.3 Hz, 1H), 5.51 (t, *J* = 7.2 Hz, 1H), 4.85 (d, *J* = 7.2 Hz, 2H), 3.91 (s, 3H), 2.40 (s, 3H), 1.84 (s, 3H), 1.82 (s, 3H).

^{13}C NMR (101 MHz, $CDCl_3$) δ 163.9, 158.3, 145.7, 139.3, 135.9, 134.7, 130.8, 130.2, 127.1, 122.8, 121.6, 118.6, 113.9, 113.4, 97.5, 61.5, 55.8, 25.9, 21.7, 18.2.

HRMS: m/z $[M+H]^+$ calcd for $C_{22}H_{24}NO_5S$: 414.1370; found: 414.1369.

m.p. 126 – 127 ^oC.

3-methylbut-2-en-1-yl 6-methyl-1-tosyl-1*H*-indole-3-carboxylate (1ad)

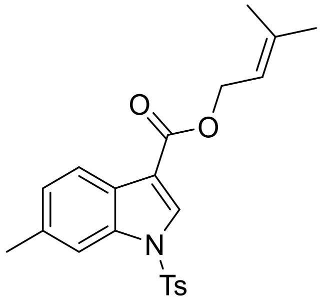

The title compound was prepared from 6-methyl-1-tosyl-1*H*-indole-3-carboxylic acid (1.72 g, 10 mmol, 1.0 equiv.), 3-methyl-2-buten-1-ol (1.5 mL, 15 mmol, 1.5 equiv.). Product **1ad** was obtained as a white solid (1.09 g, 2.70 mmol, 27% yield).

TLC: R_f = 0.4 (Petroleum ether /ethyl acetate 10:1).

^{1}H NMR (400 MHz, $CDCl_3$) δ 8.20 (s, 1H), 7.99 (d, *J* = 8.1 Hz, 1H), 7.81 (d, *J* = 8.4 Hz, 2H), 7.76 (s, 1H), 7.27 (s, 1H), 7.25 (s, 1H), 7.15 (d, *J* = 7.5 Hz, 1H), 5.49 (t, *J* = 7.2 Hz, 1H), 4.82 (d, *J* = 7.2 Hz, 2H), 2.48 (s, 3H), 2.36 (s, 3H), 1.81 (s, 3H), 1.78 (s, 3H).

^{13}C NMR (101 MHz, $CDCl_3$) δ 163.87, 145.65, 139.22, 135.59, 135.25, 134.83, 131.42, 130.17, 127.10, 125.97, 125.58, 121.75, 118.69, 113.75, 113.31, 61.44, 25.86, 22.00, 21.66, 18.18.

HRMS: m/z $[M+H]^+$ calcd for $C_{22}H_{24}NO_4S$: 398.1421; found: 398.1426.

m.p. 81 – 82 ^oC.

3-methylbut-2-en-1-yl 1-tosyl-1*H*-pyrrolo[2,3-b] pyridine-3-carboxylate (1ae)

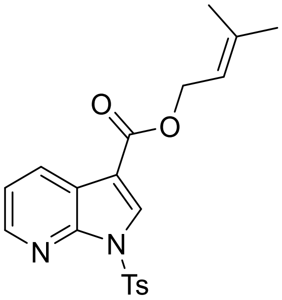

The title compound was prepared from 1-tosyl-1*H*-pyrrolo[2,3-b] pyridine-3-carboxylic acid (1.62 g, 10 mmol, 1.0 equiv.), 3-methyl-2-buten-1-ol (1.5 mL, 15 mmol, 1.5 equiv.). Product **1ae** was obtained as a white solid (924 mg, 2.40 mmol, 24% yield).

TLC: R_f = 0.4 (Petroleum ether /ethyl acetate 10:1).

^{1}H NMR (400 MHz, $CDCl_3$) δ 8.46 (dd, *J* = 4.8, 1.7 Hz, 1H), 8.41 (s, 1H), 8.38 (dd, *J* = 8.0, 1.7 Hz, 1H), 8.12 (d, *J* = 8.5 Hz, 2H), 7.30 (s, 1H), 7.29 – 7.26 (m, 2H), 5.47 (t, *J* = 7.2 Hz, 1H), 4.83 (d, *J* = 7.2 Hz, 2H), 2.37 (s, 3H), 1.80 (s, 3H), 1.78 (s, 3H).

^{13}C NMR (101 MHz, $CDCl_3$) δ 163.3, 146.9, 145.9, 145.7, 139.6, 134.6, 131.7, 130.8, 129.8, 128.5, 120.6, 120.0, 118.4, 110.7, 61.6, 25.9, 21.7, 18.2.

HRMS: m/z $[M+H]^+$ calcd for $C_{20}H_{21}N_2O_4S$: 385.1217; found: 385.1216.

m.p. 96 – 97 °C.

***(E)*-3,7-dimethylocta-2,6-dien-1-yl 1-tosyl-1*H*-indole-3-carboxylate (8a)**

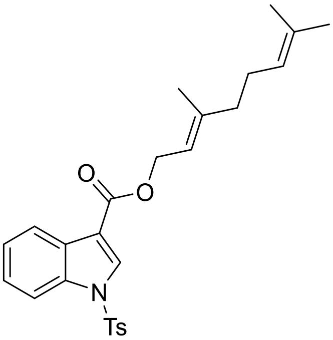

The title compound was prepared from indole-3-carboxylic acid (1.29 g, 8 mmol, 1.0 equiv.), geraniol (1.73 mL, 6.60 mmol, 1.0 equiv.). Product **8a** was obtained as a white solid (1.16 g, 2.60 mmol, 39% yield).

TLC: R_f = 0.3 (Petroleum ether /ethyl acetate 20:1).

^{1}H NMR (400 MHz, $CDCl_3$) δ 8.30 (s, 1H), 8.18 – 8.13 (m, 1H), 7.99 – 7.95 (m, 1H), 7.84 (d, *J* = 8.4 Hz, 2H), 7.40 – 7.32 (m, 2H), 7.28 (d, *J* = 8.2 Hz, 2H), 5.53 – 5.49 (m, 1H), 5.14 – 5.11 (m, 1H), 4.87 (d, *J* = 7.1 Hz, 2H), 2.38 (s, 3H), 2.21 – 2.05 (m, 4H), 1.80 (s, 3H), 1.70 (s, 3H), 1.63 (s, 3H).

^{13}C NMR (101 MHz, $CDCl_3$) δ 163.8, 145.8, 142.5, 134.8, 134.7, 132.0, 131.9, 130.2, 127.9, 127.2, 125.3, 124.4, 123.8, 122.2, 118.3, 113.8, 113.3, 61.5, 39.6, 26.3, 25.7, 21.7, 17.8, 16.6.

HRMS: m/z $[M+H]^+$ calcd for $C_{26}H_{30}NO_4S$: 452.1890; found: 452.1895.

m.p. 86 – 87 °C.

3,7,11-trimethyldodeca-2,6,10-trien-1-yl 1-tosyl-1*H*-indole-3-carboxylate (8b)

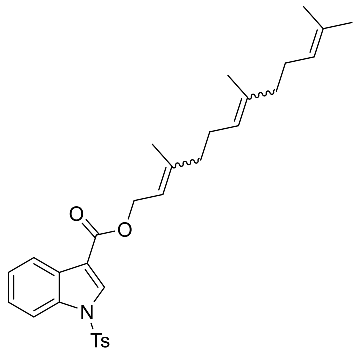

The title compound was prepared from indole-3-carboxylic acid (1.29 g, 8 mmol, 1.0 equiv.), farnesol (2.48 mL, 6.60 mmol, 1.5 equiv.). Product **8b** was obtained as a colorless oil (0.70 g, 1.34 mmol, 22% yield, mixture of isomers about 5:1).

TLC: R_f = 0.3 (Petroleum ether /ethyl acetate 20:1).

^{1}H NMR (400 MHz, $CDCl_3$) δ 8.27 (s, 1H), 8.13 (d, *J* = 7.9 Hz, 1H), 7.95 (d, *J* = 7.6 Hz, 1H), 7.82 (d, *J* = 8.3 Hz, 2H), 7.37 – 7.30 (m, 2H), 7.27 – 7.24 (m, 2H), 5.49 (t, *J* = 6.5 Hz, 1H), 5.15 – 5.03 (m, 2H), 4.85 (d, *J* = 7.1 Hz, 2H), 2.35 (s, 3H), 2.18 – 2.02 (m, 7H), 1.99 – 1.94 (m, 1H), 1.81 (s, 0.5H), 1.78 (s, 2.5H), 1.67 (d, *J* = 5.5 Hz, 3H), 1.61 – 1.57 (m, 6H).

^{13}C NMR (101 MHz, $CDCl_3$) δ 163.7, 145.7, 142.5, 142.4, 135.6, 135.5, 134.8, 134.6, 132.0, 131.6, 131.3, 130.1, 127.8, 127.1, 125.3, 124.4, 124.3, 124.2, 123.6, 122.2, 118.3, 113.7, 113.3, 61.4, 39.8, 39.7, 39.5, 32.0, 26.7, 26.6, 26.2, 26.0, 25.7, 25.6, 23.4, 21.6, 17.6, 16.6, 16.0.

HRMS: m/z $[M+H]^+$ calcd for $C_{31}H_{38}NO_4S$: 520.2516; found: 520.2513.

***(Z)*-pent-2-en-1-yl 1-tosyl-1*H*-indole-3-carboxylate (8c)**

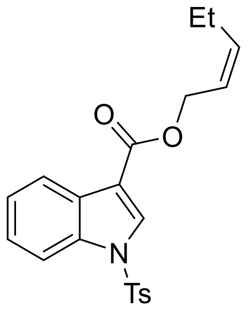

The title compound was prepared from Indole-3-carboxylic acid (0.97 g, 6 mmol, 1.0 equiv.), *cis*-pent-2-ene-1-ol (0.85 mL, 12 mmol, 1.5 equiv.). Product **8c** was obtained as a white solid (1.43 g, 3.73 mmol, 78% yield).

TLC: R_f = 0.5 (Petroleum ether /ethyl acetate 20:1).

^{1}H NMR (400 MHz, $CDCl_3$) δ 8.31 (s, 1H), 8.16 (d, *J* = 7.3 Hz, 1H), 7.98 (d, *J* = 8.0 Hz, 1H), 7.84 (d, *J* = 8.3 Hz, 2H), 7.42 – 7.31 (m, 2H), 7.27 (d, *J* = 7.8 Hz, 2H), 5.79 – 5.63 (m, 2H), 4.91 (d, *J* = 6.4 Hz, 2H), 2.37 (s, 3H), 2.26 – 2.18 (m, 2H), 1.06 (t, *J* = 7.5 Hz, 3H).

^{13}C NMR (101 MHz, $CDCl_3$) δ 163.7, 145.8, 137.3, 134.9, 134.7, 132.1, 130.2, 127.8, 127.2, 125.4, 124.4, 122.7, 122.2, 113.6, 113.35, 60.4, 21.6, 21.0, 14.2.

HRMS: m/z $[M+H]^+$ calcd for $C_{21}H_{22}NO_4S$: 384.1264; found: 384.1261.

m.p. 89 – 90 °C.

allyl 1-tosyl-1*H*-indole-3-carboxylate (8d)

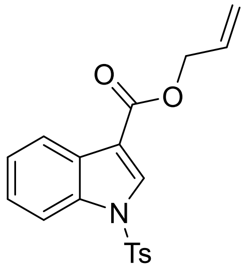

The title compound was prepared from indole-3-carboxylic acid (0.84 g, 5.20 mmol, 1.0 equiv.), allyl alcohol (0.53 mL, 7.80 mmol, 1.5 equiv.). Product **8d** was obtained as a white solid (1.07 g, 3.01 mmol, 58% yield).

TLC: R_f = 0.5 (Petroleum ether /ethyl acetate 30:1).

^{1}H NMR (400 MHz, $CDCl_3$) δ 8.35 (s, 1H), 8.18 (d, *J* = 7.4 Hz, 1H), 8.01 (d, *J* = 7.8 Hz, 1H), 7.87 (d, *J* = 8.1 Hz, 2H), 7.43 – 7.35 (m, 2H), 7.30 (d, *J* = 7.9 Hz, 2H), 6.15 – 6.06 (m, 1H), 5.47 (d, *J* = 17.2 Hz, 1H), 5.36 (d, *J* = 10.4 Hz, 1H), 4.89 (d, *J* = 5.5 Hz, 2H), 2.39 (s, 3H).

^{13}C NMR (101 MHz, $CDCl_3$) δ 163.4, 145.9, 134.8, 134.6, 132.2, 132.2, 130.2, 127.8, 127.2, 125.4, 124.5, 122.2, 118.6, 113.4, 113.4, 65.3, 217.

HRMS: m/z $[M+H]^+$ calcd for $C_{19}H_{18}NO_4S$: 356.0951; found: 356.0950.

m.p. 103 – 104 °C.

2-methylallyl 1-tosyl-1*H*-indole-3-carboxylate (8e)

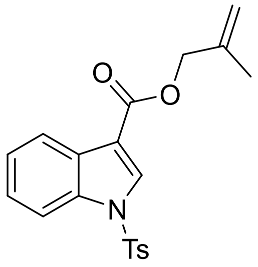

The title compound was prepared from idole-3-carboxylic acid (1.60 g, 10 mmol, 1.0 equiv.), 2-methyl-2-propen-1-ol (1.26 mL, 15 mmol, 1.5 equiv.). Product **8e** was obtained as a white solid (1.09 g, 3.0 mmol, 30% yield).

TLC: R_f = 0.4 (Petroleum ether /ethyl acetate 20:1).

^{1}H NMR (400 MHz, $CDCl_3$) δ 8.35 (s, 1H), 8.21 – 8.16 (m, 1H), 8.04 – 7.99 (m, 1H), 7.88 (d, *J* = 8.4 Hz, 2H), 7.43 – 7.36 (m, 2H), 7.34 – 7.30 (m, 2H), 5.14 (s, 1H), 5.05 (d, *J* = 0.6 Hz, 1H), 4.81 (s, 2H), 2.41 (s, 3H), 1.90 (s, 3H).

^{13}C NMR (101 MHz, $CDCl_3$) δ 163.4, 145.9, 140.0, 134.9, 134.6, 132.2, 130.2, 127.8, 127.2, 125.4, 124.5, 122.2, 113.5, 113.4, 67.8, 21.7, 19.7.

HRMS: m/z $[M+H]^+$ calcd for $C_{20}H_{20}NO_4S$: 370.1108; found: 370.1109.

m.p. 95 – 96 °C.

2-chloroallyl 1-tosyl-1*H*-indole-3-carboxylate (8f)

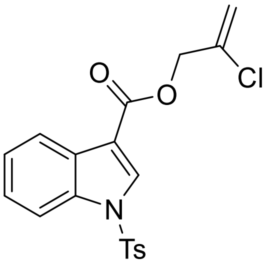

The title compound was prepared from indole-3-carboxylic acid (1.60 g, 10 mmol, 1.0 equiv.), 2-chloroallyl alcohol (1.39 g, 15 mmol, 1.5 quiv.). Product **8f** was obtained as a white solid (1.31 g, 3.4 mmol, 34% yield).

TLC: R_f = 0.4 (Petroleum ether /ethyl acetate 20:1).

^{1}H NMR (400 MHz, $CDCl_3$) δ 8.37 (s, 1H), 8.18 (d, *J* = 7.5 Hz, 1H), 8.00 (d, *J* = 8.4 Hz, 1H), 7.88 (d, *J* = 8.3 Hz, 2H), 7.44 – 7.36 (m, 2H), 7.31 (d, *J* = 8.5 Hz, 2H), 5.61 (s, 1H), 5.52 (s, 1H), 4.96 (s, 2H), 2.40 (s, 3H).

^{13}C NMR (101 MHz, $CDCl_3$) δ 162.7, 145.9, 136.0, 134.8, 134.6, 132.5, 130.3, 127.6, 127.2, 125.5, 124.6, 122.1, 115.4, 113.4, 112.8, 66.0, 21.7.

HRMS: m/z $[M+H]^+$ calcd for $C_{19}H_{17}{}^{35}ClNO_4S$: 390.0561; found: 390.0563.

HRMS: m/z $[M+H]^+$ calcd for $C_{19}H_{17}{}^{37}ClNO_4S$: 392.0532; found: 392.0531.

m.p. 72 – 73 ^{o}C.

but-2-yn-1-yl 1-tosyl-1*H*-indole-3-carboxylate (8g)

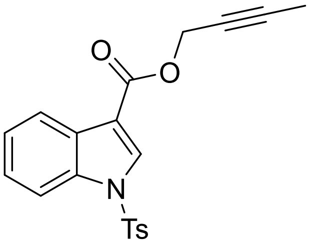

The title compound was prepared from indole-3-carboxylic acid (1.60 g, 10 mmol, 1.0 equiv.), 2-butyn-1-0l (1.12 mL, 15 mmol, 1.5 equiv.). Product **8g** was obtained as a white solid (1.91 g, 5.20 mmol, 52% yield).

TLC: R_f = 0.4 (Petroleum ether /ethyl acetate 20:1).

^{1}H NMR (400 MHz, $CDCl_3$) δ 8.34 (s, 1H), 8.17 (dd, *J* = 6.7, 2.0 Hz, 1H), 7.98 (dd, *J* = 6.9, 1.6 Hz, 1H), 7.85 (d, *J* = 8.4 Hz, 2H), 7.41 – 7.35 (m, 2H), 7.29 (d, *J* = 8.2 Hz, 2H), 4.92 (q, *J* = 2.3 Hz, 2H), 2.38 (s, 3H), 1.91 (t, *J* = 2.4 Hz, 3H).

^{13}C NMR (101 MHz, $CDCl_3$) δ 163.0, 145.9, 134.8, 134.6, 132.4, 130.2, 127.7, 127.2, 125.5, 124.5, 122.2, 113.3, 112.9, 83.5, 73.2, 52.9, 21.7, 3.8.

HRMS: m/z $[M+H]^+$ calcd for $C_{20}H_{18}NO_4S$: 368.0951; found: 368.0947.

m.p. 136 – 137 ^{o}C.

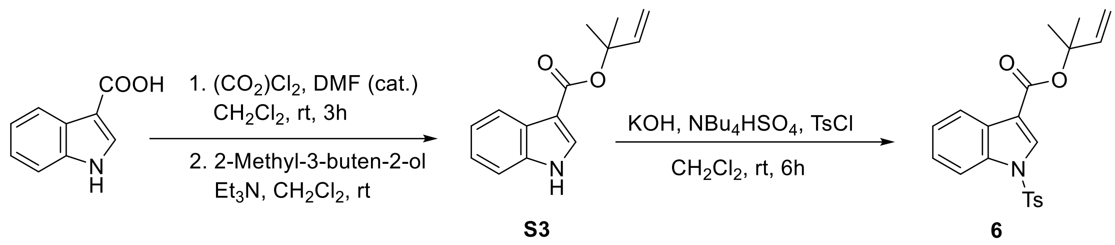

The compound **6** was synthesized according to a modified literature procedure.[1] Indole-3-carboxylic acid (10 mmol) was dissolved in dry CH_2Cl_2 (20 mL) and oxalyl chloride (30 mmol) was added dropwise at 0°C under the argon atmosphere. Afterwards, DMF (dry, 5 drops) was added and the reaction mixture was stirred for 3 h at room temperature. All volatiles were removed under reduced pressure and the acid chloride was subsequently used without further purification. To an oven-dried round-bottom flask were added the 2-methyl-3-buten-2-ol (8.0 mmol), triethylamine (8.8 mmol), and dry CH_2Cl_2 (10 mL) under an argon atmosphere. The mixture was cooled to 0 °C, and the acid chloride (10 mmol) was added dropwise. The reaction was allowed to warm up to room temperature and stirred overnight. Water was added to quench the reaction. The organic layer was washed with saturated aqueous solution of NH_4Cl, brine, dried over anhydrous Na_2SO_4, filtered and concentrated in vacuo. The residue was purified by silica gel column chromatography (Petroleum ether / ethyl acetate = 10 :1, v/v) to afford the compound **S3**.

To a solution of **S3** in CH_2Cl_2 (10 ml) were added NBu_4HSO_4 (0.1 equiv.) and KOH (2.0 equiv.) at room temperature. After stirring for 1 h, TsCl (1.5 equiv.) was added and the reaction was stirred for 6 h. After completion of the reaction monitored by TLC, the organic layer was washed with saturated aqueous solution of brine, dried over anhydrous Na_2SO_4, filtered and concentrated in vacuo. The residue was purified by column chromatography on silica gel (Petroleum ether / EtOAc= 50: 1, v/v) to afford the product of **6.**

2-methylbut-3-en-2-yl 1-tosyl-1*H*-indole-3-carboxylate (6)

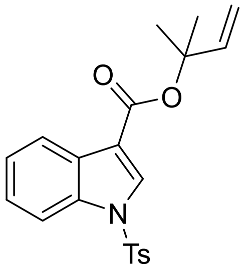

The title compound was prepared from Indole-3-carboxylic acid (1.60 g, 10 mmol, 1.2 equiv.), 2-methyl-3-buten-2-ol (0.83 ml, 8.0 mmol, 1.0 equuiv.). Product **6** was obtained as a white solid (0.974 g, 2.54 mmol, 32% yield).

TLC: R_f = 0.4 (Petroleum ether /ethyl acetate 20:1).

^{1}H NMR (400 MHz, $CDCl_3$) δ 8.26 (s, 1H), 8.16 (d, *J* = 7.3 Hz, 1H), 7.99 (d, *J* = 7.8 Hz, 1H), 7.87 (d, *J* = 8.0 Hz, 2H), 7.41 – 7.35 (m, 2H), 7.33 – 7.29 (m, 2H), 6.29 (dd, *J* = 17.5, 10.9 Hz, 1H), 5.31 (d, *J* = 17.5 Hz, 1H), 5.20 (d, *J* = 10.9 Hz, 1H), 2.39 (s, 3H), 1.73 (s, 6H).

^{13}C NMR (101 MHz, $CDCl_3$) δ 162.7, 145.8, 142.5, 134.9, 134.7, 131.8, 130.2, 127.9, 127.2, 125.3, 124.3, 122.2, 114.8, 113.3, 113.1, 81.7, 26.9, 21.7.

HRMS: m/z $[M+H]^+$ calcd for $C_{21}H_{22}NO_4S$: 384.1264; found: 384.1266.

m.p. 92 – 93 °C.

2.2 Synthesis of α-Silylamines

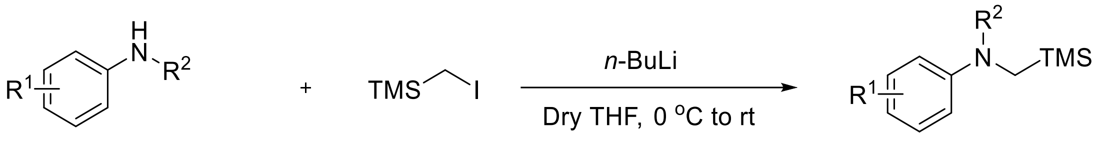

R^2 = H, CH_3, Ph, Isopropyl, Benzyl

2

To a solution of amines (8 mmol) in dry THF (10 mL) under argon atmosphere at 0 °C was added *n*-BuLi (2.5 M in *n*-Hexane, 8.8 mmol) dropwise. The reaction was stirred at 0 °C for 2 h. Then (iodomethyl)trimethylsilane (8 mmol) was added slowly to the flask at 0 °C, and the resulting solution was stirred at room temperature overnight. Afterwards, the reaction was quenched with water and extracted with EtOAc. The combined organic layers were dried with anhydrous Na_2SO_4 and concentrated under reduced pressure. The residue was purified by flash chromatography on silica gel (100 % Petroleum ether) to α-silylamines **2**.

4-(methyl((trimethylsilyl)methyl)amino)benzonitrile (2g)

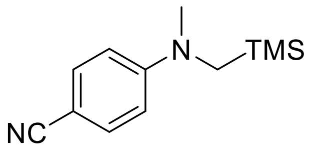

The title compound was prepared from 4-(N-methylamino)benzonitrile (1.0 g, 7.5 mmol, 1.0 equiv.), (iodomethyl)trimethylsilane (1.11 mL, 7.5 mmol, 1.0 equiv.). Product **2g** was obtained as a yellow oil (1.92 g, 8.81 mmol, 71% yield).

TLC: R_f = 0.4 (Petroleum ether).

^{1}H NMR (400 MHz, $CDCl_3$) δ 7.42 (d, *J* = 8.7 Hz, 2H), 6.57 (d, *J* = 8.7 Hz, 2H), 3.01 (s, 3H), 2.96 (s, 2H), 0.11 (s, 9H).

^{13}C NMR (101 MHz, $CDCl_3$) δ 153.4, 134.8, 122.6, 112.4. 97.2, 45.2, 41.6, 0.3.

HRMS: m/z $[M+H]^+$ calcd for $C_{12}H_{19}N_2Si$: 219.1312; found: 219.1321.

***N*,2-dimethyl-*N*-((trimethylsilyl)methyl)aniline (2i)**

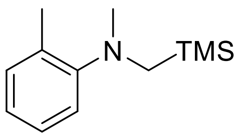

The title compound was prepared from 2-dimethyl-benzenamin (1.0 mL, 8.0 mmol, 1.0 equiv.), (iodomethyl)trimethylsilane (1.12 mL, 8.0 mmol, 1.0 equiv.). Product **2i** was obtained as a yellow oil (1.24 g, 6.0 mmol, 75% yield).

TLC: R_f = 0.4 (Petroleum ether).

^{1}H NMR (500 MHz, $CDCl_3$) δ 7.20 – 7.16 (m, 3H), 7.00 – 6.96 (m, 1H), 2.67 (s, 3H), 2.53 (s, 2H), 2.35 (s, 3H), 0.06 (s, 9H).

^{13}C NMR (101 MHz, $CDCl_3$) δ 154.9, 133.1, 130.9, 126.4, 122.9, 120.0, 48.3, 46.3, 18.2, 1.3.

HRMS: m/z $[M+H]^+$ calcd for $C_{12}H_{22}NSi$: 208.1516; found: 208.1517.

***N*-allyl-*N*-((trimethylsilyl)methyl)aniline (2j)**

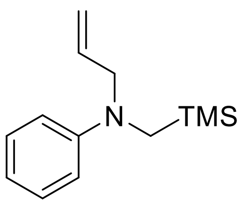

The title compound was prepared from *N*-allybenzenamine (1.08 mL, 8.0 mmol, 1.0 equiv.), (iodomethyl)trimethylsilane (1.12 mL, 8.0 mmol, 1.0 equiv.). Product **2j** was obtained as a lightly yellow oil (1.27 g, 5.8 mmol, 72% yield).

TLC: R_f = 0.3 (Petroleum ether).

^{1}H NMR (400 MHz, $CDCl_3$) δ 7.24 – 7.18 (m, 2H), 6.68 – 5.77 (m, 3H), 5.90 – 5.77 (m, 1H), 5.22 – 5.10 (m, 2H), 3.93 (d, *J* = 4.8 Hz, 2H), 2.89 (s, 2H), 0.13 (s, 9H).

^{13}C NMR (101 MHz, $CDCl_3$) δ 150.7, 134.8, 130.2, 117.6, 116.4, 113.4, 56.0, 43.0, 0.2.

HRMS: m/z $[M+H]^+$ calcd for $C_{13}H_{22}NSi$: 220.1516; found: 220.1516.

4-(tert-butyl)-N-((trimethylsilyl)methyl)aniline (4d)

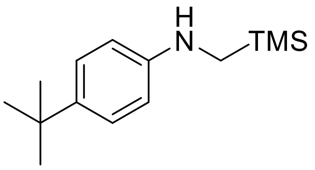

The title compound was prepared from 4-tert-butylaniline (0.54 g, 5.0 mmol, 1.0 equiv.), (iodomethyl)trimethylsilane (0.78 mL, 5.0 mmol, 1.0 equiv.). Product **4d** was obtained as an orange solid (812 mg, 3.5 mmol, 69% yield).

TLC: R_f = 0.4 (Petroleum ether /ethyl acetate 20:1).

^{1}H NMR (400 MHz, $CDCl_3$) δ 7.28 (d, *J* = 8.0 Hz, 2H), 6.70 (d, *J* = 8.1 Hz, 2H), 3.44 (s, 1H), 2.55 (s, 2H), 1.35 (m, 9H), 0.19 (s, 9H).

^{13}C NMR (101 MHz, $CDCl_3$) δ 151.2, 142.7, 128.8, 115.0, 36.8, 36.6, 34.5, 0.3.

HRMS: m/z $[M+H]^+$ calcd for $C_{14}H_{26}NSi$: 236.1829; found: 236.1829.

m.p. 38 – 39 ^oC.

3-methoxy-N-((trimethylsilyl)methyl)aniline (4h)

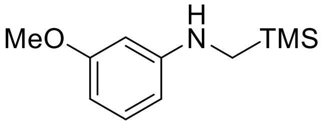

The title compound was prepared from 3-methoxy-benzenamine (0.62 mL, 5.0 mmol, 1.0 equiv.), (iodomethyl)trimethylsilane (0.78 mL, 5.0 mmol, 1.0 equiv.). Product **4h** was obtained as an orange oil (648 mg, 3.1 mmol, 62% yield).

TLC: R_f = 0.4 (Petroleum ether /ethyl acetate 20:1).

^{1}H NMR (400 MHz, $CDCl_3$) δ 7.16 – 7.00 (m, 1H), 6.31 – 6.22 (m, 3H), 3.79 (s, 3H), 2.49 (s, 2H), 0.14 (s, 9H).

^{13}C NMR (101 MHz, $CDCl_3$) δ 164.2, 155.3, 133.2, 109.0, 105.6, 101.5, 58.4, 36.9, 0.7.

HRMS: m/z $[M+H]^+$ calcd for $C_{11}H_{20}NOSi$: 210.1309; found: 210.1307.

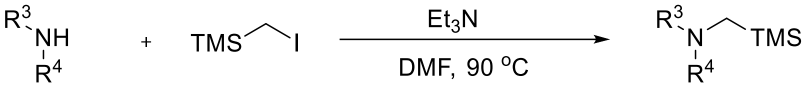

2

To a solution of the secondary amine or its HCl salt (8 mmol) in DMF (1 M) were added Et_3N (32 mmol) and (iodomethyl)trimethylsilane (8.8 mmol) dropwise under an argon atmosphere. The reaction was heated to 90 °C and was stirred for 16 h. Upon completion of the reaction, the organic layer was extracted with EtOAc (3 x 25 mL), then washed with brine (15 mL) and dried over anhydrous Na_2SO_4. The organic solvent was removed under reduced pressure and the crude reaction residue was purified by flash column chromatography on silica gel to give the α-silylamine.[2]

Methyl-1-ethyl-6-fluoro-4-oxo-7-(4-((trimethylsilyl)methyl)piperazin-1-yl)-1,4-dihydroquinoline-3-carboxylate (2da)

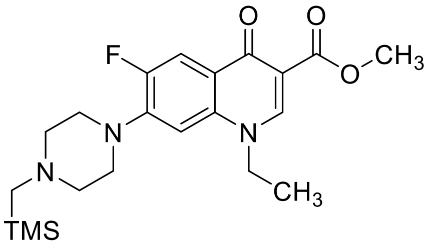

The title compound was prepared from methyl-1-ethyl-6-fluoro-4-oxo-7-(piperazin-1-yl)-1,4-dihydroquinoline-3-carboxylate (433 mg, 1.3 mmol, 1.0 equiv.), (iodomethyl)trimethylsilane (0.23 mL, 1.43 mmol, 1.1 equiv.), Et_3N (0.72 mL, 5.2 mmol, 4.0 equiv.). Product **2da** was obtained as a white solid (245 mg, 58% yield).

TLC: R_f = 0.4 (Dichloromethane / methanol 10:1).

^{1}H NMR (400 MHz, $CDCl_3$) δ 8.40 (s, 1H), 8.01 (d, *J* = 13.3 Hz, 1H), 6.70 (d, *J* = 6.5 Hz, 1H), 4.17 (d, *J* = 7.0 Hz, 2H), 3.89 (s, 3H), 3.29 (s, 4H), 2.76 (s, 4H), 2.13 (s, 2H), 1.51 (t, *J* = 6.9 Hz, 3H), 0.12 (s, 9H).

^{13}C NMR (101 MHz, $CDCl_3$) δ 174.5, 168.0, 154.7 (d, $^1J_{C-F}$ = 249.5 Hz), 149.7, 146.0 (d, $^3J_{C-F}$ = 10.8 Hz), 137.5, 125.3 (d, $^4J_{C-F}$ =6.6 Hz), 115.1 (d, $^2J_{C-F}$ = 22.9 Hz), 111.5, 105.4, 57.8, 53.5, 52.0, 51.0, 50.5, 15. 9, 0.3.

HRMS: m/z $[M+H]^+$ calcd for $C_{21}H_{31}FN_3O_3Si$: 420.2113; found: 420.2114.

m.p. 133 – 144 °C.

(1R,5R)-3-((trimethylsilyl)methyl)-1,2,3,4,5,6-hexahydro-8*H*-1,5-methanopyrido-*[1,2-a][1,5]*diazocin-8-one (2db)

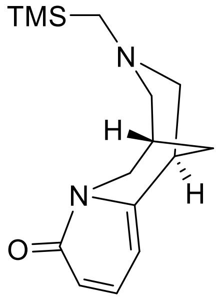

The title compound was prepared from cytysine (1.0 g, 5.0 mmol, 1.0 equiv.), (iodomethyl)trimethylsilane (1.17 mL, 5.5 mmol, 1.1 equiv.), Et_3N (2.78 mL, 20 mmol, 4.0 equiv.). Product **2db** was obtained as an orange oil (1.12 g, 4.06 mmol, 81% yield).

TLC: R_f = 0.5 (Dichloromethane / methanol 20:1).

^{1}H NMR (400 MHz, $CDCl_3$) δ 7.56 (dd, *J* = 8.9, 7.0 Hz, 1H), 6.71 (d, *J* = 9.0 Hz, 1H), 6.28 (d, *J* = 6.8 Hz, 1H), 4.37 (d, *J* = 15.3 Hz, 1H), 4.14 (dd, *J* = 15.3, 6.6 Hz, 1H), 3.21 (s, 1H), 3.13 (t, *J* = 12.7 Hz, 2H), 2.67 (s, 1H), 2.58 (t, *J* = 11.7 Hz, 2H), 2.16 – 2.01 (m, 4H), 0.15 (s, 9H).

^{13}C NMR (101 MHz, $CDCl_3$) δ 165.4, 153.6, 140.4, 118.2, 106.3, 66.2, 65.6, 52.0, 51.71, 37.8, 30.4, 27.3, 0.3.

HRMS: m/z $[M+H]^+$ calcd for $C_{15}H_{25}N_2OSi$: 277.1731; found: 277.1731.

benzyl 1-((trimethylsilyl)methyl)octahydrocyclopenta*[b]*pyrrole-2-carboxylate (2de)

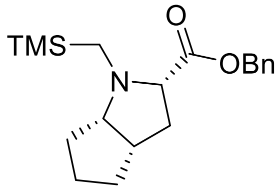

The title compound was prepared from *(S,S,S)*-2-azabicyclo*[3,3,0]*octane-3-carboxylic acid benzyl ester hydrochloride (1.7 g, 6.0 mmol, 1.0 equiv.), (iodomethyl)trimethylsilane (1.0 mL, 6.6 mmol, 1.1 equiv.), Et_3N (3.4 mL, 24 mmol, 4.0 equiv.). Product **2de** was obtained as a yellow oil (1.6 g, 4.83 mmol, 81% yield).

TLC: R_f = 0.4 (Petroleum ether).

^{1}H NMR (400 MHz, $CDCl_3$) δ 7.36 – 7.30 (m, 5H), 5.21 – 5.08 (m, 2H), 3.19 (dd, *J* = 10.8, 5.9 Hz, 1H), 3.03 – 2.95 (m, 1H), 2.64 – 2.54 (m, 1H), 2.30 (d, *J* = 14.6 Hz, 1H), 2.22 – 2.18 (m, 1H), 1.95 (d, *J* = 14.6 Hz, 1H), 1.79 – 1.67 (m, 2H), 1.54 – 1.44 (m, 4H), 1.40 – 1.32 (m, 1H), 0.05 (s, 9H).

^{13}C NMR (101 MHz, $CDCl_3$) δ 173.7, 136.2, 128.5, 128.4, 128.1, 72.0, 71.8, 66.0, 46.9, 41.9, 36.9, 34.7, 32.7, 23.9, –1.0.

HRMS: m/z $[M+Na]^+$ calcd for $C_{19}H_{29}NO_2NaSi$: 354.1860; found: 354.1858.

1-(bis(4-fluorophenyl)methyl)-4-((trimethylsilyl)methyl)piperazine (2df)

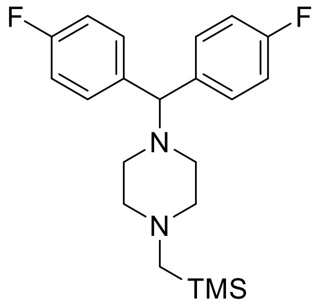

The title compound was prepared from 4,4'-difluorobenzhydrylpiperazine (1.0 g, 3.5 mmol, 1.0 equiv.), (iodomethyl)trimethylsilane (0.52 mL, 3.85 mmol, 1.1 equiv.), Et_3N (1.95 mL, 14 mmol, 4.0 equiv.). Product **2df** was obtained as a lightly yellow solid (995 mg, 2.66 mmol, 76% yield).

TLC: R_f = 0.3 (Petroleum ether /ethyl acetate 5:1).

^{1}H NMR (400 MHz, $CDCl_3$) δ 7.39 – 7.33 (m, 4H), 7.01 – 6.95 (m, 4H), 4.20 (s, 1H), 2.68 – 2.08 (br, 8H), 1.94 (s, 2H), 0.06 (s, 9H).

^{13}C NMR (101 MHz, $CDCl_3$) δ 163.2 (d, $^1J_{C\text{-}F}$ = 244.0 Hz), 139.9 (d, $^4J_{C\text{-}F}$ = 2.8 Hz), 130.6 (d, $^3J_{C\text{-}F}$ = 7.7 Hz), 116.7 (d, $^2J_{C\text{-}F}$ = 21.1 Hz), 76.0, 58.6, 53.5, 52.1, 0.2.

^{19}F NMR (376 MHz, CDCl3) δ –115.8.

HRMS: m/z $[M+H]^+$ calcd for $C_{21}H_{29}F_2N_2Si$: 375.2063; found: 375.2065.

m.p. 62 – 63 °C.

5-((trimethylsilyl)methyl)-4,5,6,7-tetrahydrothieno*[3,2-c]*pyridine (2dg)

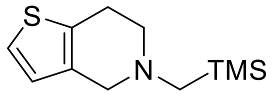

The title compound was prepared from 4*H*,5*H*,6*H*,7*H*-thieno[3,2-c]pyridine hydrochloride (1.4 g, 8.0 mmol, 1.0 equiv), (iodomethyl)trimethylsilane (1.31 mL, 8.8 mmol, 1.1 equiv.), Et_3N (4.45 mL, 32 mmol, 4.0 equiv.). Product **2dg** was obtained as a yellow oil (1.5 g, 6.67 mmol, 84% yield).

TLC: R_f = 0.2 (Petroleum ether /ethyl acetate 5:1).

^{1}H NMR (400 MHz, $CDCl_3$) δ 7.09 (d, *J* = 5.1 Hz, 1H), 6.73 (d, *J* = 5.1 Hz, 1H), 3.54 (s, 2H), 2.91 (t, *J* = 5.6 Hz, 2H), 2.76 (t, *J* = 5.7 Hz, 2H), 2.14 (s, 2H), 0.14 (s, 9H).

^{13}C NMR (101 MHz, $CDCl_3$) δ 134.3, 133.1, 125.3, 122.5, 57.0, 54.2, 50.0, 25.6, –1.1.

HRMS: m/z $[M+H]^+$ calcd for $C_{11}H_{20}NSSi$: 226.1080; found: 226.1079.

3. Supplementary method B: photoredox-catalysed diastereoselective dearomative (reverse-)prenylation of electron-deficient indoles

In the glovebox, to a flame-dried 8 mL reaction vial equipped with a stir bar were added dimethylallyl indole-3-carboxylate (0.3 mmol, 1.0 equiv.) and 4-CzIPN (3.0 mg, 0.0039 mmol, 0.013 equiv.) in dry DMF (0.5 mL). Then the solution of α-silylamine (0.36 mmol, 1.2 equiv.) in dry DMF (0.5 mL) was added. The vial was sealed and transferred out of the glove box. It was irradiated with a 1 W blue LED lamp (SYNLED) for 2 h at room temperature. Afterwards, the reaction mixture was allowed to heat at 60 °C for 3 h without light. When the reaction was completed (monitored by TLC), the crude mixture was quenched by water and extracted with ethyl acetate (10 mL x 2). The combined organic layers were washed with water (10 mL x 2) and brine (10 mL), dried over anhydrous Na_2SO_4, filtered, and concentrated by rotary evaporation. Then the residue was purified by silica gel flash chromatography to give the corresponding product (Supplementary Fig. 1).

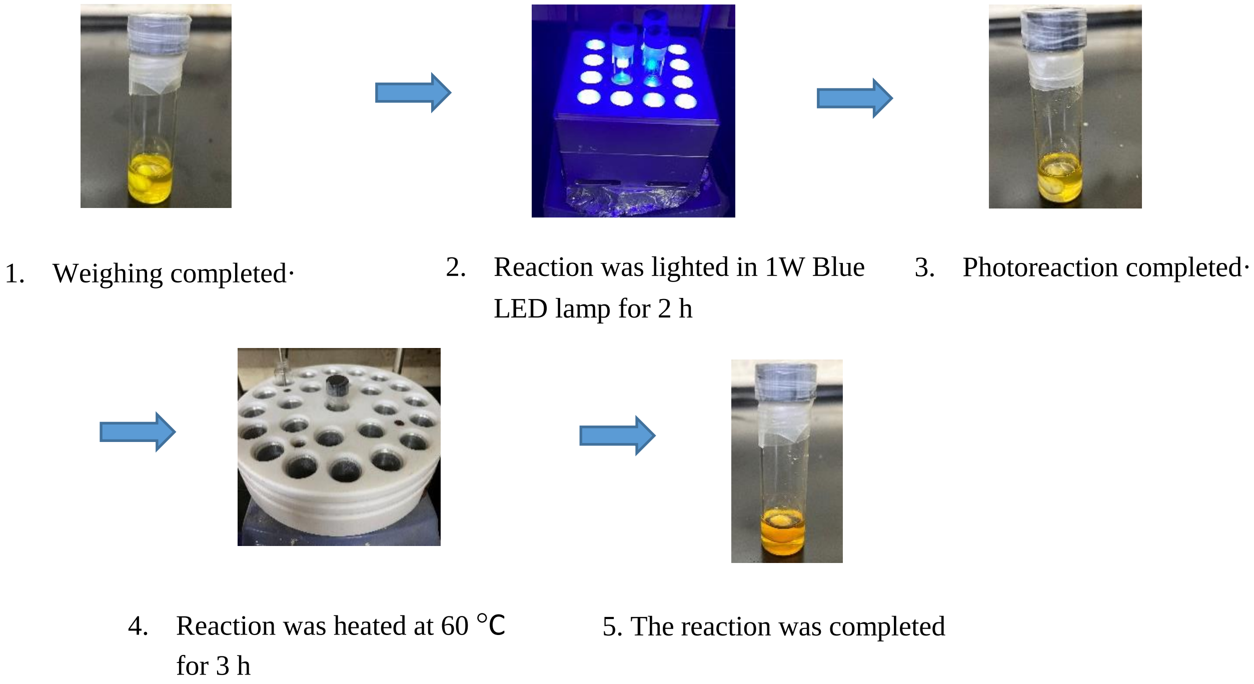

Supplementary Fig. 1. The reaction workflow.

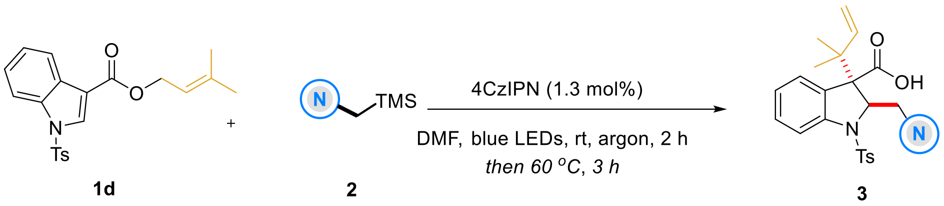

2-((methyl(phenyl)amino)methyl)-3-(2-methylbut-3-en-2-yl)-1-tosylindoline-3-carboxylic acid (3d)

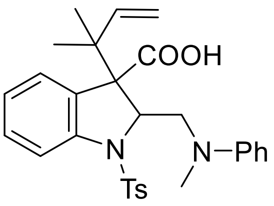

Following the general procedure, 3-methylbut-2-en-1-yl 1-tosyl-1*H*-indole-3-carboxylate (77.0 mg, 0.2 mmol, 1.0 equiv.), *N*-methyl-*N*-((trimethylsilyl)methyl)aniline (46.3 mg, 0.24 mmol, 1.2 equiv.), 4CzIPN (2.0 mg, 0.0026 mmol, 0.013 equiv.), lighted in 1 W Blue LED for 2 h, then 60 °C reaction for 3 h. Product **3d** was obtained as a yellow solid (72 mg, 71% yield).

TLC: R_f = 0.3 (Petroleum ether /ethyl acetate 2:1).

^{1}H NMR (400 MHz, $CDCl_3$) δ 7.67 (d, *J* = 8.0 Hz, 2H), 7.58 – 7.50 (m, 2H), 7.33 – 7.29 (m, 1H), 7.23 – 7.15 (m, 2H), 7.12 (d, *J* = 7.9 Hz, 2H), 7.08 – 7.04 (m, 1H), 6.90 – 6.84 (m, 1H), 6.81 (d, *J* = 7.6 Hz, 2H), 6.10 (dd, *J* = 17.2, 10.9 Hz, 1H), 4.91 (t, *J* = 5.9 Hz, 1H), 4.86 – 4.72 (m, 2H), 3.62 (dd, *J* = 13.9, 6.6 Hz, 1H), 3.46 (dd, *J* = 13.7, 6.3 Hz, 1H), 2.68 (s, 3H), 2.33 (s, 3H), 0.96 (s, 3H), 0.58 (s, 3H).

^{13}C NMR (101 MHz, $CDCl_3$) δ 175.2, 148.5, 143.9, 143.8, 141.5, 137.2, 131.3, 129.5, 129.2, 129.0, 128.8, 127.0, 122.9, 120.7, 116.6, 114.4, 114.3, 65.8, 64.3, 56.1, 43.8, 41.8, 24.2, 22.0, 21.5.

HRMS: m/z $[M+H]^+$ calcd for $C_{29}H_{33}N_2O_4S$: 505.2156; found: 505.2157.

m.p. 174 – 175 °C.

2-(((4-fluorophenyl)(methyl)amino)methyl)-3-(2-methylbut-3-en-2-yl)-1-tosylindoline-3-carboxylic acid (3e)

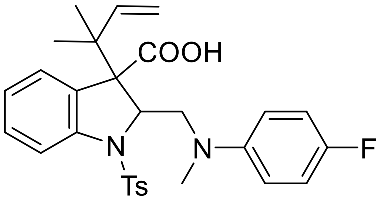

Following the general procedure, 3-methylbut-2-en-1-yl-1-tosyl-1*H*-indole-3-carboxylate (115.0 mg, 0.3 mmol, 1.0 equiv.), 4-fluoro-*N*-methyl-*N*-((trimethylsilyl)methyl)aniline (75.9 mg, 0.36 mmol, 1.2 equiv.), 4CzIPN (3.0 mg, 0.0039 mmol, 0.013 equiv.), lighted in 1 W Blue LED for 2 h, then 60 °C reaction for 3 h. Product **3e** was obtained as a yellow solid (112 mg, 72% yield).

TLC: R_f = 0.4 (Petroleum ether /ethyl acetate 2:1).

^{1}H NMR (400 MHz, $CDCl_3$) δ 7.69 (d, *J* = 8.2 Hz, 2H), 7.51 (d, *J* = 8.1 Hz, 2H), 7.31 – 7.27 (m, 1H), 7.15 (d, *J* = 8.2 Hz, 2H), 7.08 – 7.02 (m, 1H), 6.91 – 6.83 (m, 2H), 6.76 (dd, *J* = 8.8, 4.4 Hz, 2H), 6.07 (dd, *J* = 17.4, 10.8 Hz, 1H), 4.87 – 4.73 (m, 3H), 3.57 (dd, *J* = 13.9, 6.5 Hz, 1H), 3.45 (dd, *J* = 14.0, 6.4 Hz, 1H), 2.65 (s, 3H), 2.34 (s, 3H), 0.93 (s, 3H), 0.53 (s, 3H).

^{13}C NMR (101 MHz, $CDCl_3$) δ 175.8, 157.7 (d, $^1J_{C\text{-}F}$ = 238.2 Hz), 145.4, 144.0, 143.8, 141.5, 137.1, 131.1, 129.5, 129.0, 128.9, 127.0, 122.9, 118.6 (d, $^3J_{C\text{-}F}$ = 7.5 Hz), 115.4 (d, $^2J_{C\text{-}F}$ = 22.1 Hz), 114.4, 114.2, 65.4, 64.6, 56.4, 43.8, 42.8, 24.1, 21.9, 21.5.

^{19}F NMR (376 MHz, $CDCl_3$) δ –123.5.

HRMS: m/z $[M+H]^+$ calcd for $C_{29}H_{32}FN_2O_4S$: 523.2061; found: 523.2063.

m.p. 93 – 94°C.

2-(((4-bromophenyl)(methyl)amino)methyl)-3-(2-methylbut-3-en-2-yl)-1-tosylindoline-3-carboxylic acid (3f)

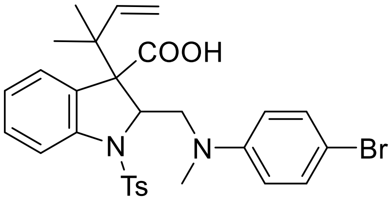

Following the general procedure, 3-methylbut-2-en-1-yl-1-tosyl-1*H*-indole-3-carboxylate (115.0 mg, 0.3 mmol, 1.0 equiv.), 4-bromo-*N*-((trimethylsilyl)-methyl)aniline (97.6 mg, 0.36 mmol, 1.2 equiv.), 4CzIPN (3.0 mg, 0.0039 mmol, 0.013 equiv.), lighted in 1 W Blue LED for 2 h, then 60 °C reaction for 3 h. Product **3f** was obtained as a white solid (147 mg, 84% yield).

TLC: R_f = 0.3 (Petroleum ether /ethyl acetate 2:1).

^{1}H NMR (400 MHz, $CDCl_3$) δ 7.69 (d, *J* = 8.3 Hz, 2H), 7.55 (d, *J* = 8.0 Hz, 1H), 7.50 – 7.45 (m, 1H), 7.42 – 7.33 (m, 1H), 7.19 (d, *J* = 9.0 Hz, 2H), 7.12 – 7.10 (m, 3H), 6.45 (d, *J* = 9.0 Hz, 2H), 6.12 (dd, *J* = 17.4, 10.9 Hz, 1H), 5.05 (t, *J* = 6.6 Hz, 1H), 4.99 (d, *J* = 10.8 Hz, 1H), 4.93 (d, *J* = 17.4 Hz, 1H), 3.52 – 3.39 (m, 2H), 2.63 (s, 3H), 2.36 (s, 3H), 0.99 (s, 3H), 0.74 (s, 3H).

^{13}C NMR (101 MHz, $CDCl_3$) δ 176.6, 148.8, 143.9, 143.4, 141.7, 137.3, 131.5, 131.1, 129.4, 129.1, 128.7, 126.8, 123.1, 116.5, 114.9, 114.8, 110.9, 65.0, 64.9, 55.1, 43.9, 40.4, 24.1, 22.2, 21.5.

HRMS: m/z $[M+H]^+$ calcd for $C_{29}H_{32}{}^{79}BrN_2O_4S$: 583.1261; found: 583.1264.

HRMS: m/z $[M+H]^+$ calcd for $C_{29}H_{32}{}^{81}BrN_2O_4S$: 585.1241; found: 585.1245.

m.p. 87 – 88 °C.

2-(((4-cyanophenyl)(methyl)amino)methyl)-3-(2-methylbut-3-en-2-yl)-1-tosylindoline-3-carboxylic acid (3g)

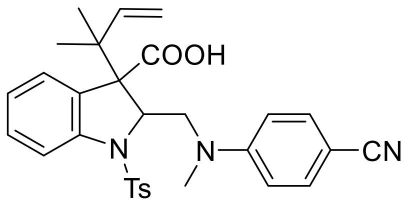

Following the general procedure, 3-methylbut-2-en-1-yl 1-tosyl-1*H*-indole-3-carboxylate (115.0 mg, 0.3 mmol, 1.0 equiv.), 4-(methyl((trimethylsilyl)-methyl)amino)-benzonitrile (78.5 mg, 0.36 mmol, 1.2 equiv.), 4CzIPN (3.0 mg, 0.0039 mmol, 0.013 equiv.), lighted in 1 W Blue LED for 2 h, then 60 °C reaction for 3 h. Product **3g** was obtained as a yellow solid (120 mg, 76% yield).

TLC: R_f = 0.3 (Petroleum ether /ethyl acetate 2:1).

^{1}H NMR (400 MHz, $CDCl_3$) δ 7.64 (d, *J* = 8.2 Hz, 2H), 7.58 (d, *J* = 8.1 Hz, 1H), 7.48 (d, *J* = 7.6 Hz, 1H), 7.39 – 7.33 (m, 1H), 7.32 (d, *J* = 8.5 Hz, 2H), 7.16 – 7.08 (m, 3H), 6.45 (d, *J* = 8.8 Hz, 2H), 6.12 (dd, *J* = 17.4, 10.8 Hz, 1H), 5.09 (dd, *J* = 9.2, 4.1 Hz, 1H), 5.01 (d, *J* = 10.9 Hz, 1H), 4.93 (d, *J* = 17.5 Hz, 1H), 3.63 (dd, *J* = 14.3, 3.5 Hz, 1H), 3.45 (dd, J = 14.5, 9.4 Hz, 1H), 2.77 (s, 3H), 2.34 (s, 3H), 0.94 (s, 3H), 0.71 (s, 3H).

^{13}C NMR (101 MHz, $CDCl_3$) δ 175.5, 152.7, 144.2, 143.3, 141.4, 137.1, 133.1, 130.8, 129.5, 129.4, 128.8, 127.0, 123.4, 120.4, 115.3, 115.0, 112.6, 98.4, 65.2, 64.4, 54.2, 43.7, 39.2, 24.2, 22.2, 21.6.

HRMS: m/z $[M+H]^+$ calcd for $C_{30}H_{32}N_3O_4S$: 530.2108; found: 530.2110.

m.p. 171 – 172 °C.

2-(((3-chlorophenyl)(methyl)amino)methyl)-3-(2-methylbut-3-en-2-yl)-1-tosylindoline-3-carboxylic acid (3h)

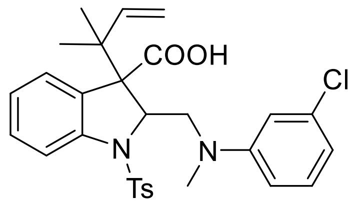

Following the general procedure, 3-methylbut-2-en-1-yl-1-tosyl-1*H*-indole-3-carboxylate (115.0 mg, 0.3 mmol, 1.0 equiv.), 3-chloro-*N*-methyl-*N*-((trimethylsilyl)me-thyl)aniline (81.7 mg, 0.36 mmol, 1.2 equiv.), 4CzIPN (3.0 mg, 0.0039 mmol, 0.013 equiv.), lighted in 1 W Blue LED for 2 h, then 60 °C reaction for 3 h. Product **3h** was obtained as a yellow solid (138 mg, 86% yield).

TLC: R_f = 0.2 (Petroleum ether /ethyl acetate 2:1).

^{1}H NMR (400 MHz, $CDCl_3$) δ 7.68 (d, *J* = 8.1 Hz, 2H), 7.56 (d, *J* = 8.1 Hz, 1H), 7.47 (d, *J* = 7.7 Hz, 1H), 7.38 – 7.34 (m, 1H), 7.15 – 7.07 (m, 3H), 6.98 (t, *J* = 8.1 Hz, 1H), 6.65 (d, *J* = 7.8 Hz, 1H), 6.47 (s, 1H), 6.41 (d, *J* = 8.4 Hz, 1H), 6.12 (dd, *J* = 17.4, 10.8 Hz, 1H), 5.10 – 5.02 (m, 1H), 4.99 (d, *J* = 10.9 Hz, 1H), 4.93 (d, *J* = 17.5 Hz, 1H), 3.50 – 3.39 (m, 2H), 2.62 (s, 3H), 2.3 (s, 3H), 0.98 (s, 3H), 0.74 (s, 3H).

^{13}C NMR (101 MHz, $CDCl_3$) δ 175.8, 151.1, 143.8, 143.4, 141.8, 137.3, 134.5, 130.9, 129.6, 129.4, 129.1, 128.7, 126.8, 123.1, 118.1, 115.1, 114.8, 114.5, 112.6, 77.4, 77.3, 77.1, 76.7, 64.9, 54.8, 43.9, 39.9, 24.0, 22.2, 21.5.

HRMS: m/z $[M+H]^+$ calcd for $C_{29}H_{32}{}^{35}ClN_2O_4S$: 539.1766; found: 539.1765.

HRMS: m/z $[M+H]^+$ calcd for $C_{29}H_{32}{}^{37}ClN_2O_4S$: 541.1736; found: 541.1729.

m.p. 177 – 178 °C.

2-((methyl(phenyl)amino)methyl)-3-(2-methylbut-3-en-2-yl)-1-tosylindoline-3-carboxylic acid (3i)

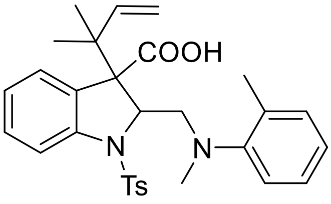

Following the general procedure, 3-methylbut-2-en-1-yl 1-tosyl-1*H*-indole-3-carboxylate (77.0 mg, 0.2 mmol, 1.0 equiv.), 2-dimethyl-*N*-((trimethylsilyl)methyl)aniline (49.7 mg, 0.24 mmol, 1.2 equiv.), 4CzIPN (2.0 mg, 0.0026 mmol, 0.013 equiv.), lighted in 1 W Blue LED for 2 h, then 60 °C reaction for 3 h. Product **3i** was obtained as a white solid (93 mg, 89% yield).

TLC: R_f = 0.4 (Petroleum ether /ethyl acetate 2:1).

^{1}H NMR (400 MHz, $CDCl_3$) δ 7.74 (d, *J* = 8.3 Hz, 2H), 7.65 (t, *J* = 8.1 Hz, 2H), 7.58 (d, *J* = 8.1 Hz, 1H), 7.33 (t, *J* = 7.5 Hz, 1H), 7.26 – 7.22 (m, 3H), 7.21 – 7.13 (m, 2H), 7.08 – 7.00 (m, 1H), 5.85 (dd, *J* = 17.2, 11.0 Hz, 1H), 4.50 (s, 1H), 4.48 – 4.38 (m, 2H), 4.08 (dd, *J* = 13.5, 2.2 Hz, 1H), 3.46 (dd, *J* = 13.5, 9.7 Hz, 1H), 2.68 (s, 3H), 2.38 (s, 3H), 2.29 (s, 3H), 0.83 (s, 3H), 0.12 (s, 3H).

^{13}C NMR (101 MHz, $CDCl_3$) δ 173.6, 146.5, 144.6, 143.5, 141.1, 136.3, 133.0, 132.0, 130.9, 129.9, 129.6, 128.7, 127.3, 126.9, 125.8, 122.9, 120.8, 114.0, 113.4, 66.8, 63.3, 56.9, 46.2, 43.7, 24.0, 21.6, 21.3, 18.0.

HRMS: m/z $[M+H]^+$ calcd for $C_{30}H_{35}N_2O_4S$: 519.2312; found: 519.2318.

m.p. 186 – 187 °C.

2-((allyl(phenyl)amino)methyl)-3-(2-methylbut-3-en-2-yl)-1-tosylindoline-3-carboxylic acid (3j)

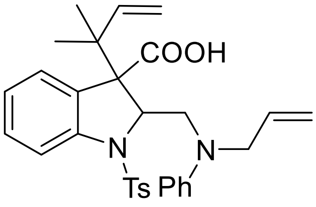

Following the general procedure, 3-methylbut-2-en-1-yl 1-tosyl-1*H*-indole-3-carboxylate (115.0 mg, 0.3 mmol, 1.0 equiv.), *N*-allyl-*N*-((trimethylsilyl)methyl)aniline (78.8 mg, 0.36 mmol, 1.2 equiv.), 4CzIPN (3.0 mg, 0.0039 mmol, 0.013 equiv.), lighted in 1 W Blue LED for 2 h, then 60 °C reaction for 3 h. Product **3j** was obtained as a white solid (140 mg, 88% yield).

TLC: R_f = 0.5 (Petroleum ether /ethyl acetate 2:1).

^{1}H NMR (400 MHz, $CDCl_3$) δ 7.67 (d, *J* = 8.3 Hz, 2H), 7.55 (d, *J* = 8.2 Hz, 2H), 7.31 (t, *J* = 8.3 Hz, 1H), 7.24 (t, *J* = 7.9 Hz, 2H), 7.16 (d, *J* = 8.2 Hz, 2H), 7.10 – 7.04 (m, 1H), 6.93 – 6.88 (m, 3H), 6.08 (dd, *J* = 17.4, 10.9 Hz, 1H), 5.69 – 5.56 (m, 1H), 5.02 (dd, *J* = 25.1, 13.6 Hz, 2H), 4.92 (t, *J* = 6.7 Hz, 1H), 4.82 – 4.70 (m, 2H), 3.67 – 3.52 (m, 4H), 2.36 (s, 3H), 0.94 (s, 3H), 0.56 (s, 3H).

^{13}C NMR (101 MHz, $CDCl_3$) δ 175.9, 146.6, 143.9, 143.8, 141.5, 137.1, 131.4, 131.3, 129.5, 129.1, 129.0, 128.8, 127.1, 123.0, 120.8, 119.2, 117.8, 114.5, 114.2, 65.9, 64.1, 56.7, 52.6, 43.7, 24.2, 22.1, 21.6.

HRMS: m/z $[M+H]^+$ calcd for $C_{31}H_{35}N_2O_4S$: 531.2312; found: 531.2315.

m.p. 159 – 160 °C.

2-((benzyl(phenyl)amino)methyl)-3-(2-methylbut-3-en-2-yl)-1-tosylindoline-3-carboxylic acid (3k)

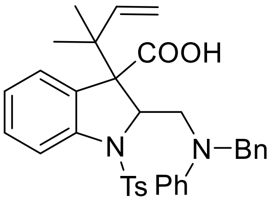

Following the general procedure, 3-methylbut-2-en-1-yl 1-tosyl-1*H*-indole-3-carboxylate (115.0 mg, 0.3 mmol, 1.0 equiv.), *N*-benzyl-*N*-((trimethylsilyl)methyl)aniline (96.8 mg, 0.36 mmol, 1.2 equiv.), 4CzIPN (3.0 mg, 0.0039 mmol, 0.013 equiv.), lighted in 1 W Blue LED for 2 h, then 60 °C reaction for 3 h. Product **3k** was obtained as a white solid (157 mg, 90% yield).

TLC: R_f = 0.3 (Petroleum ether /ethyl acetate 2:1).

^{1}H NMR (400 MHz, $CDCl_3$) δ 7.69 – 7.67 (m, 2H), 7.59 – 7.53 (m, 2H), 7.32 (d, *J* = 7.6 Hz, 1H), 7.26 – 7.13 (m, 7H), 7.08 – 7.02 (m, 1H), 6.96 – 6.92 (m, 3H), 6.80 – 6.84 (m, 2H), 6.13 – 6.04 (m, 1H), 5.02 – 4.92 (m, 1H), 4.79 – 4.72 (m, 2H), 4.39 – 4.25 (m, 1H), 4.18 – 4.08 (m, 1H), 3.78 – 3.62 (m, 2H), 2.39 (s, 3H), 0.94 (s, 3H), 0.56 (s, 3H).

^{13}C NMR (101 MHz, $CDCl_3$) δ 175.9, 146.5, 144.0, 143.8, 141.4, 137.2, 135.8, 131.4, 129.6, 129.0, 128.8, 128.4, 128.2, 127.3, 127.2, 123.0, 120.7, 118.0, 114.4, 114.3, 65.9, 64.1, 58.0, 53.0, 43.7, 24.1, 22.1, 21.6.

HRMS: m/z $[M+H]^+$ calcd for $C_{35}H_{37}N_2O_4S$: 581.2469; found: 581.2463.

m.p. 96 – 97 °C.

2-((isopropyl(phenyl)amino)methyl)-3-(2-methylbut-3-en-2-yl)-1-tosylindoline-3-carboxylic acid (3l)

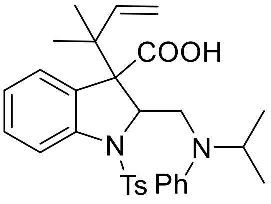

Following the general procedure, 3-methylbut-2-en-1-yl-1-tosyl-1*H*-indole-3-carboxylate (115.0 mg, 0.3 mmol, 1.0 equiv.), *N*-isopropyl-*N*-((trimethylsilyl)methyl)aniline (79.6 mg, 0.36 mmol, 1.2 equiv.), 4CzIPN (3.0 mg, 0.0039 mmol, 0.013 equiv.), lighted in 1 W Blue LED for 2 h, then 60 °C reaction for 3 h. Product **3l** was obtained as a white solid (130 mg, 81% yield).

TLC: R_f = 0.2 (Petroleum ether /ethyl acetate 2:1).

^{1}H NMR (400 MHz, $CDCl_3$) δ 7.95 (d, *J* = 7.7 Hz, 1H), 7.59 (d, J = 8.1 Hz, 1H), 7.54 (d, *J* = 8.3 Hz, 2H), 7.47 – 7.40 (m, 5H), 7.25 – 7.23 (m, 1H), 7.16 (d, *J* = 8.2 Hz, 2H), 7.07 – 7.01 (m, 1H), 5.91 (dd, *J* = 17.4, 10.8 Hz, 1H), 4.42 (d, *J* = 10.8 Hz, 1H), 4.34 (d, *J* = 17.4 Hz, 1H), 4.24 (d, *J* = 8.7 Hz, 1H), 4.13 (d, *J* = 13.1 Hz, 1H), 3.54 – 3.47 (m, 1H), 3.32 (dd, *J* = 13.3, 10.0 Hz, 1H), 2.34 (s, 3H), 1.14 (d, *J* = 6.6 Hz, 3H), 1.07 (d, *J* = 6.5 Hz, 3H), 0.82 (s, 3H), 0.05 (s, 3H).

^{13}C NMR (101 MHz, $CDCl_3$) δ 172.7, 144.5, 144.4, 140.5, 138.4, 136.1, 131.0, 130.6, 129.7, 129.0, 128.4, 127.7, 127.2, 125.5, 123.1, 113.4, 112.9, 68.6, 61.8, 58.1, 53.7, 43.6, 24.6, 21.6, 21.3, 18.7, 17.1.

HRMS: m/z $[M+H]^+$ calcd for $C_{31}H_{37}N_2O_4S$: 533.2474; found: 533.2472.

m.p. 99 – 100 °C.

2-((diphenylamino)methyl)-3-(2-methylbut-3-en-2-yl)-1-tosylindoline-3-carboxylic acid (3m)

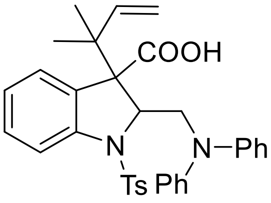

Following the general procedure, 3-methylbut-2-en-1-yl-1-tosyl-1*H*-indole-3-carboxylate (77.0 mg, 0.2 mmol, 1.0 equiv.), *N*-phenyl-*N*-((trimethylsilyl)methyl)aniline (61.2 mg, 0.24 mmol, 1.2 equiv.), 4CzIPN (2.0 mg, 0.0026 mmol, 0.013 equiv.), lighted in 1 W Blue LED for 2 h, then 60 °C reaction for 3 h. Product **3m** was obtained as a yellow oil (62 mg, 55% yield).

TLC: R_f = 0.3 (Petroleum ether /ethyl acetate 2:1).

^{1}H NMR (500 MHz, $CDCl_3$) δ 7.74 (d, *J* = 8.0 Hz, 2H), 7.35 (d, *J* = 8.0 Hz, 1H), 7.28 – 7.22 (m, 2H), 7.21 – 7.16 (m, 6H), 7.06 – 7.06 (m, 1H), 6.98 – 6.88 (m, 6H), 5.93 (dd, *J* = 17.5, 11.0 Hz, 1H), 4.89 (t, *J* = 5.5 Hz, 1H), 4.77 (d, *J* = 11.0 Hz, 1H), 4.67 (d, *J* = 17.5 Hz, 1H), 4.21 (dd, *J* = 14.0, 5.5 Hz, 1H), 4.06 (dd, *J* = 14.0, 5.5 Hz, 1H), 2.36 (s, 3H), 0.73 (s, 3H), 0.39 (s, 3H).

^{13}C NMR (126 MHz, $CDCl_3$) δ 175.9, 148.5, 144.2, 143.5, 141.9, 137.3, 131.1, 129.8, 128.9, 128.8, 128.7, 127.4, 122.7, 122.6, 122.4, 114.4, 65.3, 64.4, 53.7, 43.9, 23.9, 22.1, 21.6.

HRMS: m/z $[M+H]^+$ calcd for $C_{34}H_{35}N_2O_4S$: 567.2312; found: 567.2315.

m.p. 165 – 166 °C.

2-((bis(4-bromophenyl)amino)methyl)-3-(2-methylbut-3-en-2-yl)-1-tosylindoline-3-carboxylic acid (3n)

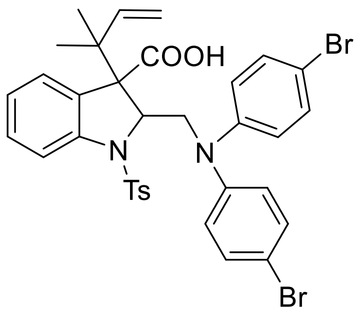

Following the general procedure, 3-methylbut-2-en-1-yl-1-tosyl-1*H*-indole-3-carboxylate (77.0 mg, 0.2 mmol, 1.0 equiv.), 4-bromo-*N*-(4-bromophenyl)-*N*-((trimethylsilyl)methyl)aniline (98.6 mg, 0.24 mmol, 1.2 equiv.), 4CzIPN (2.0 mg, 0.0026 mmol, 0.013 equiv.), lighted in 1 W Blue LED for 2 h, then 60 °C reaction for 3 h. Product **3n** was obtained as a white solid (55 mg, 57% yield, 16:1 d.r.).

TLC: R_f = 0.4 (Petroleum ether /ethyl acetate 2:1).

^{1}H NMR (400 MHz, $CDCl_3$) δ 7.72 (d, *J* = 8.2 Hz, 2H), 7.34 (d, *J* = 8.0 Hz, 1H), 7.30 – 7.23 (m, 8H), 7.12 – 7.06 (m, 1H), 6.82 – 6.72 (m, 4H), 5.86 (dd, *J* = 17.4, 10.8 Hz, 1H), 4.80 (d, *J* = 11.0 Hz, 1H), 4.73 (t, *J* = 4.9 Hz, 1H), 4.65 (d, *J* = 17.4 Hz, 1H), 4.23 (dd, *J* = 14.3, 4.8 Hz, 1H), 4.12 (dd, *J* = 14.3, 5.1 Hz, 1H), 2.39 (s, 3H), 0.65 (s, 3H), 0.17 (s, 3H).

^{13}C NMR (101 MHz, $CDCl_3$) δ 176.5, 146.8, 144.5, 143.0, 141.5, 136.52, 131.9, 130.3, 129.8, 128.9, 128.5, 127.2, 123.9, 122.9, 115.7, 114.7, 114.0, 65.2, 63.7, 53.3, 43.9, 23.7, 21.6, 21.4 .

HRMS: m/z $[M+H]^+$ calcd for $C_{34}H_{33}{}^{79}Br_2N_2O_4S$: 723.0522; found: 723.0518.

HRMS: m/z $[M+H]^+$ calcd for $C_{34}H_{33}{}^{81}Br_2N_2O_4S$: 725.0502; found: 725.0508.

m.p. 95 – 96 °C.

2-((9*H*-carbazol-9-yl)methyl)-3-(2-methylbut-3-en-2-yl)-1-tosylindoline-3-carboxylic acid (3o)

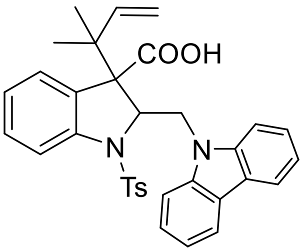

Following the general procedure, 3-methylbut-2-en-1-yl-1-tosyl-1*H*-indole-3-carboxylate (77.0 mg, 0.2 mmol, 1.0 equiv.), 9-((trimethylsilyl)methyl)-9*H*-carbazole (60.7 mg, 0.24 mmol, 1.2 equiv.), 4CzIPN (2.0 mg, 0.0026 mmol, 0.013 equiv.), lighted in 1 W Blue LED for 2 h, then 60 °C reaction for 3 h. Product **3o** was obtained as a lightly yellow solid (56 mg, 50% yield).

TLC: R_f = 0.2 (Petroleum ether /ethyl acetate 2:1).

^{1}H NMR (400 MHz, $CDCl_3$) δ 7.81 (d, *J* = 7.6 Hz, 2H), 7.67 (d, *J* = 7.7 Hz, 1H), 7.49 – 7.44 (m, 2H), 7.32 – 7.28 (m, 2H), 7.23 – 7.19 (m, 1H), 7.14 – 7.07 (m, 4H), 6.87 (d, *J* = 8.2 Hz, 2H), 6.51 (d, *J* = 8.1 Hz, 2H), 6.23 (dd, *J* = 17.4, 10.9 Hz, 1H), 5.76 (dd, *J* = 10.9, 4.2 Hz, 1H), 5.15 (dd, *J* = 14.0, 10.1 Hz, 2H), 4.41 (dd, *J* = 14.6, 4.2 Hz, 1H), 4.08 (dd, *J* = 14.6, 11.0 Hz, 1H), 2.19 (s, 3H), 1.16 (s, 3H), 1.13 (s, 3H).

^{13}C NMR (101 MHz, $CDCl_3$) δ 176.6, 143.2, 142.5, 141.9, 141.4, 136.4, 131.0, 129.3, 129.0, 128.8, 125.8, 125.3, 123.6, 123.5, 119.9, 119.3, 116.6, 115.2, 108.9, 66.5, 64.76, 46.1, 43.9, 24.5, 22.8, 21.5.

HRMS: m/z $[M+H]^+$ calcd for $C_{34}H_{33}N_2O_4S$: 565.2156; found: 565.2157.

m.p. 114 – 115 °C.

2-((benzyl(methyl)amino)methyl)-3-(2-methylbut-3-en-2-yl)-1-tosylindoline-3-carboxylic acid (3p)

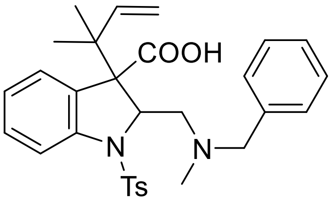

Following the general procedure, 3-methylbut-2-en-1-yl 1-tosyl-1*H*-indole-3-carboxylate (77.0 mg, 0.2 mmol, 1.0 equiv.), *N*-benzyl-*N*-methyl-1-(trimethylsilyl)methanemine (49.7 mg, 0.24 mmol, 1.2 equiv.), 4CzIPN (2.0 mg, 0.0026 mmol, 0.013 equiv.), lighted in 1 W Blue LED for 2 h, then 60 °C reaction for 3 h. Product **3p** was obtained as a colorless oil (76 mg, 49% yield).

TLC: R_f = 0.2 (Petroleum ether /ethyl acetate 2:1).

1H NMR (400 MHz, CDCl3) δ 8.03 (d, *J* = 7.7 Hz, 1H), 7.79 (d, *J* = 8.2 Hz, 2H), 7.54 (d, *J* = 8.1 Hz, 1H), 7.45 – 7.41 (m, 5H), 7.31 – 7.21 (m, 3H), 7.10 – 7.02 (m, 1H), 6.39 (dd, *J* = 17.6, 10.8 Hz, 1H), 4.97 (d, *J* = 11.2 Hz, 1H), 4.76 (d, *J* = 17.6 Hz, 1H), 4.66 (d, *J* = 10.4 Hz, 1H), 4.40 – 3.64 (br, 2H), 3.49 (d, *J* = 12.7 Hz, 1H), 3.29 – 3.21 (m, 1H), 2.54 (s, 3H), 2.40 (s, 3H), 1.07 (s, 3H), 0.03 (s, 3H).

^{13}C NMR (101 MHz, $CDCl_3$) δ 172.5, 146.6, 145.0, 140.0, 136.1, 131.4, 131.1, 130.9, 130.0, 129.9, 129.5, 129.4, 128.3, 127.2, 123.3, 113.3, 112.4, 68.8, 60.8, 60.6, 43.6, 38.7, 25.1, 21.6, 20.4.

HRMS: m/z $[M+H]^+$ calcd for $C_{30}H_{35}N_2O_4S$: 519.2312; found: 519.2314.

2-((methyl(phenethyl)amino)methyl)-3-(2-methylbut-3-en-2-yl)-1-tosylindoline-3-carboxylic acid (3q)

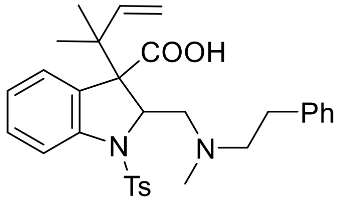

Following the general procedure, 3-methylbut-2-en-1-yl-1-tosyl-1*H*-indole-3-carboxylate (77.0 mg, 0.2 mmol, 1.0 equiv.), *N*-methyl-2-phenyl-*N*-((trimethylsilyl)methyl)ethan-1-amine (53.0 mg, 0.24 mmol, 1.2 equiv.), 4CzIPN (2.0 mg, 0.0026 mmol, 0.013 equiv.), lighted in 1 W Blue LED for 2 h, then 60 °C reaction for 3 h. Product **3q** was obtained as a lightly yellow solid (57.6 mg, 36% yield).

TLC: R_f = 0.3 (Petroleum ether /ethyl acetate 2:1).

^{1}H NMR (400 MHz, $CDCl_3$) δ 7.99 (d, *J* = 7.7 Hz, 1H), 7.78 (d, *J* = 8.3 Hz, 2H), 7.54 (d, *J* = 8.1 Hz, 1H), 7.34 – 7.26 (m, 5H), 7.24 – 7.18 (m, 3H), 7.08 – 7.02 (m, 1H), 6.36 (dd, *J* = 17.6, 10.8 Hz, 1H), 5.00 (d, *J* = 11.0 Hz, 1H), 4.75 (d, *J* = 17.6 Hz, 1H), 4.57 (d, *J* = 9.9 Hz, 1H), 3.48 (d, *J* = 12.6 Hz, 1H), 3.34 – 3.16 (br, 1H), 3.17 – 2.97 (m, 4H), 2.81 (s, 3H), 2.40 (s, 3H), 1.03 (s, 3H), 0.02 (s, 3H).

^{13}C NMR (101 MHz, $CDCl_3$) δ 172.6, 146.5, 145.0, 140.0, 136.4, 136.0, 131.5, 130.8, 130.0, 129.0, 128.7, 128.3, 127.2, 127.1, 123.3, 113.2, 112.5, 68.8, 60.6, 59.9, 57.2, 43.5, 40.3, 30.6, 25.1, 21.6, 20.4.

HRMS: m/z $[M+Na]^+$ calcd for $C_{31}H_{36}N_2NaO_4S$: 555.2288; found: 555.2291.

m.p. 98 – 99 °C.

2-((4-(ethoxycarbonyl)piperidin-1-yl)methyl)-3-(2-methylbut-3-en-2-yl)-1-tosylindoline-3-carboxylic acid (3r)

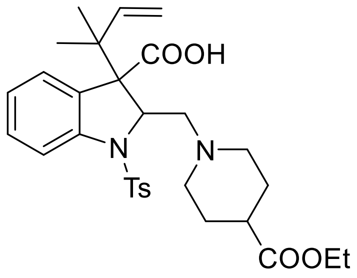

Following the general procedure, 3-methylbut-2-en-1-yl-1-tosyl-1*H*-indole-3-carboxylate (115.0 mg, 0.3 mmol, 1.0 equiv.), ethyl-1-((trimethylsilyl)methyl)-piperidine-4-carboxylate (87.5 mg, 0.36 mmol, 1.2 equiv.), 4CzIPN (3.0 mg, 0.0039 mmol, 0.013 equiv.), lighted in 1 W Blue LED for 2 h, then 60 °C reaction for 3 h. Product **3r** was obtained as a yellow solid (126 mg, 76% yield).

TLC: R_f = 0.3 (Dichloromethane / methanol 30:1).

^{1}H NMR (400 MHz, $CDCl_3$) δ 8.06 (d, *J* = 7.8 Hz, 1H), 7.88 (d, *J* = 8.2 Hz, 2H), 7.62 (d, *J* = 8.1 Hz, 1H), 7.39 (d, *J* = 9.6 Hz, 2H), 7.35 – 7.31 (m, 1H), 7.18 – 7.14 (m, 1H), 6.43 (dd, *J* = 17.5, 10.9 Hz, 1H), 5.14 (d, *J* = 10.8 Hz, 1H), 4.89 (d, *J* = 17.6 Hz, 1H), 4.64 (d, *J* = 11.0 Hz, 1H), 4.31 – 4.25 (m, 2H), 3.50 (d, *J* = 12.8 Hz, 1H), 3.22 – 3.10 (br, 1H), 3.08 – 3.02 (m, 1H), 2.93 – 2.45 (br, 6H), 2.44 – 1.66 (m, 5H), 1.37 (d, *J* = 7.1 Hz, 3H), 1.14 (s, 3H), 0.10 (d, *J* = 2.8 Hz, 3H).

^{13}C NMR (101 MHz, CDCl3) δ 172.9, 172.1, 146.3, 145.0, 140.1, 136.0, 131.2, 130.9, 129.9, 128.4, 127.2, 123.3, 113.3, 112.6, 69.1, 61.1, 60.2, 59.9, 50.6, 43.6, 25.9, 25.0, 21.6, 20.3, 14.2.

HRMS: m/z $[M+H]^+$ calcd for $C_{30}H_{39}N_2O_6S$: 555.2523; found: 555.2525.

m.p. 78 – 79 °C.

2-((1,4-dioxa-8-azaspiro[4.5]decan-8-yl)methyl)-3-(2-methylbut-3-en-2-yl)-1-tosyl-indoline-3-carboxylic acid (3s)

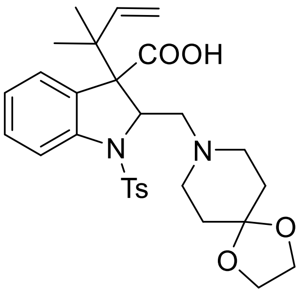

Following the general procedure, 3-methylbut-2-en-1-yl 1-tosyl-1*H*-indole-3-carboxylate (115.0 mg, 0.3 mmol, 1.0 equiv.), 8-((trimethylsilyl)methyl)-1,4-dioxa-8-azaspiro-*[4.5]*decane (82.4 mg, 0.36 mmol, 1.2 equiv.), 4CzIPN (3.0 mg, 0.0039 mmol, 0.013 equiv.), lighted in 1 W Blue LED for 2 h, then 60 °C reaction for 3 h. Product **3s** was obtained as a lightly yellow solid (111 mg, 69% yield).

TLC: R_f = 0.2 (Dichloromethane / methanol 20:1).

^{1}H NMR (400 MHz, $CDCl_3$) δ 7.96 (d, *J* = 7.8 Hz, 1H), 7.80 (d, *J* = 8.2 Hz, 2H), 7.55 (d, *J* = 8.1 Hz, 1H), 7.32 (d, *J* = 8.1 Hz, 2H), 7.29 – 7.23 (m, 1H), 7.10 – 7.04 (m, 1H), 6.39 (dd, *J* = 17.6, 10.8 Hz, 1H), 5.05 (d, *J* = 10.8 Hz, 1H), 4.81 (d, *J* = 17.6 Hz, 1H), 4.54 (d, *J* = 10.8 Hz, 1H), 4.04 – 3.98 (br, 4H), 3.62 – 2.86 (br, 6H), 2.42 (s, 3H), 2.06 – 1.96 (m, 2H), 1.95 – 1.81 (m, 2H), 1.07 (s, 3H), 0.02 (s, 3H).

^{13}C NMR (101 MHz, CDCl3) δ 172.4, 146.4, 145.0, 140.1, 135.9, 131.4, 130.8, 130.0, 128.4, 127.2, 123.2, 113.2, 112.6, 104.7, 69.0, 64.8, 60.2, 59.6, 50.2, 43.5, 33.0, 25.0, 21.6, 20.3.

HRMS: m/z $[M+H]^+$ calcd for $C_{29}H_{37}N_2O_6S$: 541.2367; found: 541.2366.

m.p. 110 – 111 °C.

3-(2-methylbut-3-en-2-yl)-2-(morpholinomethyl)-1-tosylindoline-3-carboxylic acid (3t)

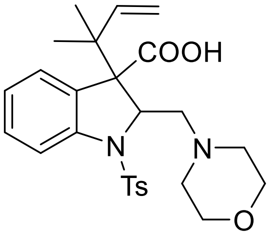

Following the general procedure, 3-methylbut-2-en-1-yl-1-tosyl-1*H*-indole-3-carboxylate (77.0 mg, 0.2 mmol, 1.0 equiv.), 4-((trimethylsilyl)methyl)morpholine (41.5 mg, 0.24 mmol, 1.2 equiv.), 4CzIPN (2.0 mg, 0.0026 mmol, 0.013 equiv.), lighted in 1 W Blue LED for 2 h, then 60 °C reaction for 3 h. Product **3t** was obtained as a white solid (75 mg, 77% yield).

TLC: R_f = 0.3 (Petroleum ether /ethyl acetate 2:1).

^{1}H NMR (400 MHz, $CDCl_3$) δ 7.94 (d, *J* = 7.7 Hz, 1H), 7.82 (d, *J* = 7.9 Hz, 2H), 7.54 (d, *J* = 8.1 Hz, 1H), 7.34 – 7.28 (m, 3H), 7.12 – 7.06 (m, 1H), 6.26 (dd, *J* = 17.5, 10.8 Hz, 1H), 5.05 (d, *J* = 10.8 Hz, 1H), 4.87 (d, *J* = 17.6 Hz, 1H), 4.59 (d, *J* = 10.3 Hz, 1H), 3.94 – 3.66 (m, 4H), 3.34 (d, *J* = 12.9 Hz, 1H), 3.26 – 2.69 (m, 5H), 2.44 (s, 3H), 1.06 (s, 3H), 0.16 (s, 3H).

^{13}C NMR (101 MHz, $CDCl_3$) δ 171.9, 145.6, 144.9, 140.4, 136.3, 130.6, 130.0, 129.7, 127.2, 123.2, 113.9, 112.8, 68.5, 65.3, 60.6, 59.9, 52.4, 43.9, 24.8, 21.6, 20.5.

HRMS: m/z $[M+H]^+$ calcd for $C_{26}H_{33}N_2O_5S$: 485.2105; found: 485.2107.

m.p. 110 – 111 °C.

2-((4-(tert-butoxycarbonyl)piperazin-1-yl)methyl)-3-(2-methylbut-3-en-2-yl)-1-tosylindoline-3-carboxylic acid (3u)

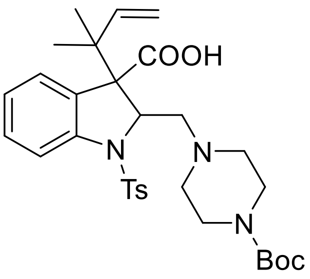

Following the general procedure, 3-methylbut-2-en-1-yl 1-tosyl-1*H*-indole-3-carboxylate (115.0 mg, 0.3 mmol, 1.0 equiv.), tert-butyl-4-((trimethylsilyl)methyl)piperazine-1-carboxylate (97.9 mg, 0.36 mmol, 1.2 equiv.), 4CzIPN (3.0 mg, 0.0039 mmol, 0.013 equiv.), lighted in 1 W Blue LED for 2 h, then 60 °C reaction for 3 h. Product **3u** was obtained as a white solid (132 mg, 75% yield).

TLC: R_f = 0.2 (Petroleum ether /ethyl acetate 2:1).

^{1}H NMR (400 MHz, $CDCl_3$) δ 7.91 (d, J = 7.8 Hz, 1H), 7.80 (d, J = 7.7 Hz, 2H), 7.52 (d, J = 8.1 Hz, 1H), 7.31 (d, J = 8.8 Hz, 2H), 7.26 (d, J = 7.8 Hz, 1H), 7.09 – 7.05 (m, 1H), 6.21 (dd, J = 17.5, 10.8 Hz, 1H), 5.05 (d, J = 10.8 Hz, 1H), 4.86 (d, J = 17.6 Hz, 1H), 4.57 (d, J = 10.1 Hz, 1H), 4.07 – 3.32 (br, 4H), 3.30 (d, J = 12.9 Hz, 1H), 3.12 – 2.85 (m, 3H), 2.78 – 2.66 (br, 2H), 2.42 (s, 3H), 1.49 (s, 9H), 1.04 (s, 3H), 0.15 (s, 3H).

^{13}C NMR (101 MHz, CDCl3) δ 171.9, 154.2, 145.4, 144.9, 140.4, 136.3, 130.5, 130.0, 128.7, 127.2, 123.2, 114.1, 112.8, 80.7, 68.3, 60.4, 60.2, 52.1, 43.9, 42.3, 41.6, 28.4, 24.7, 21.6, 20.6.

HRMS: m/z $[M+H]^+$ calcd for $C_{31}H_{42}N_3O_6S$: 584.2789; found: 584.2789.

m.p. 120 – 121 °C.

2-((4-(tert-butoxycarbonyl)-1,4-diazepan-1-yl)methyl)-3-(2-methylbut-3-en-2-yl)-1-tosylindoline-3-carboxylic acid (3v)

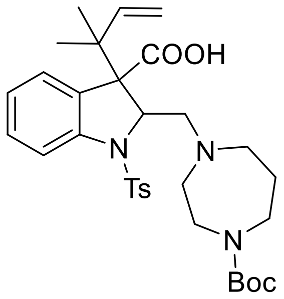

Following the general procedure, 3-methylbut-2-en-1-yl 1-tosyl-1*H*-indole-3-carboxylate (115.0 mg, 0.3 mmol, 1.0 equiv.), tert-butyl-4-((trimethylsilyl)methyl)-1,4-diazepane-1-carboxylate (103 mg, 0.36 mmol, 1.2 equiv.), 4CzIPN (3.0 mg, 0.0039 mmol, 0.013 equiv.), lighted in 1 W Blue LED for 2 h, then 60 °C reaction for 3 h. Product **3v** was obtained as a yellow oil (93 mg, 52% yield).

TLC: R_f = 0.2 (Petroleum ether /ethyl acetate 2:1).

^{1}H NMR (400 MHz, $CDCl_3$) δ 7.94 (d, *J* = 7.6 Hz, 1H), 7.78 (d, *J* = 7.6 Hz, 2H), 7.49 (d, *J* = 8.0 Hz, 1H), 7.31 – 7.27 (m, 2H), 7.25 – 7.21 (m, 1H), 7.08 – 7.02 (m, 1H), 6.32 – 6.24 (m, 1H), 5.04 – 4.96 (m, 1H), 4.79 (dd, *J* = 17.6, 5.7 Hz, 1H), 4.58 – 4.48 (m, 1H), 3.84 – 3.16 (m, 7H), 3.07 – 2.91 (m, 3H), 2.40 (s, 3H), 2.20 – 2.10 (m, 1H), 2.05 – 1.95 (m, 1H), 1.48 (s, 9H), 1.02 (s, 3H), 0.15 – 0.01 (m, 3H).

^{13}C NMR (101 MHz, CDCl3) δ 172.4, 155.1, 154.8, 146.2, 146.1, 144.9, 140.1, 136.1, 131.1, 130.7, 129.9, 128.4, 127.2, 123.2, 113.5, 112.5, 80.7, 80.6, 68.6, 68.4, 61.1, 60.9, 60.3, 59.9, 55.5, 53.6, 44.9, 43.8, 43.6, 43.0, 28.4, 25.3, 25.1, 25.0, 21.6, 20.5, 20.4.

HRMS: m/z $[M+H]^+$ calcd for $C_{32}H_{44}N_3O_6S$: 598.2945; found: 598.2948.

2-(((S)-2-(methoxycarbonyl)pyrrolidin-1-yl)methyl)-3-(2-methylbut-3-en-2-yl)-1-tosylindoline-3-carboxylic acid (3w)

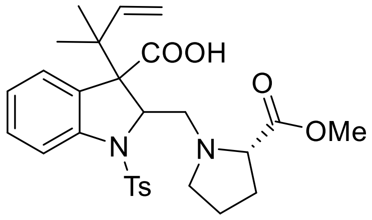

Following the general procedure, 3-methylbut-2-en-1-yl-1-tosyl-1*H*-indole-3-carboxylate (115.0 mg, 0.3 mmol, 1.0 equiv.), methyl ((trimethylsilyl)methyl)-L-prolinate (77.4 mg, 0.36 mmol, 1.2 equiv.), 4CzIPN (3.0 mg, 0.0039 mmol, 0.013 equiv.), lighted in 1 W Blue LED for 2 h, then 60 °C reaction for 3 h. Product **3w** was obtained as a yellow oil (65 mg, 41% yield, 1:1 d.r.).

TLC: R_f = 0.2 (Dichloromethane / methanol 20:1).

^{1}H NMR (400 MHz, $CDCl_3$) δ 7.80 (d, *J* = 7.8 Hz, 1H), 7.76 (d, *J* = 8.2 Hz, 2H), 7.50 (d, *J* = 8.1 Hz, 1H), 7.27 (d, *J* = 8.6 Hz, 2H), 7.21 (t, *J* = 7.5 Hz, 1H), 7.00 (t, *J* = 7.5 Hz, 1H), 6.19

(dd, J = 17.5, 10.8 Hz, 1H), 5.01 (d, J = 11.0 Hz, 1H), 4.78 (d, J = 17.5 Hz, 1H), 4.43 (dd, J = 8.5, 2.0 Hz, 1H), 3.90 (d, J = 5.6 Hz, 1H), 3.73 (s, 3H), 3.47 (d, J = 11.6 Hz, 1H), 3.37 – 3.25 (m, 2H), 3.08 (dd, J = 15.4, 8.2 Hz, 1H), 2.38 (s, 3H), 2.16 – 2.08 (m, 1H), 2.06 – 1.97 (m, 1H), 1.97 – 1.84 (m, 2H), 0.95 (s, 3H), 0.12 (s, 3H).

^{13}C NMR (101 MHz, CDCl3) δ 172.8, 171.2, 145.2, 144.7, 140.7, 136.3, 130.9, 130.2, 129.9, 128.5, 127.3, 123.0, 113.9, 112.8, 67.2, 64.5, 63.5, 56.3, 52.6, 52.2, 43.9, 28.6, 24.6, 22.4, 21.6, 20.8.

HRMS: m/z $[M+H]^+$ calcd for $C_{28}H_{35}N_2O_6S$: 527.2210; found: 527.2213.

5-chloro-2-((methyl(phenyl)amino)methyl)-3-(2-methylbut-3-en-2-yl)-1-tosylindoline-3-carboxylic acid (3x)

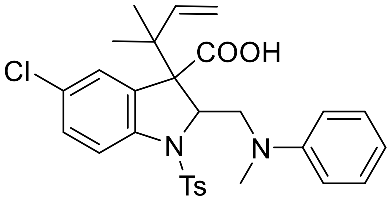

Following the general procedure, 3-methylbut-2-en-1-yl-5-chloro-1-tosyl-1*H*-indole-3-carboxylate (125.1 mg, 0.3 mmol, 1.0 equiv.), *N*-methyl-*N*-((trimethylsilyl)methyl)aniline (77.0 mg, 0.36 mmol, 1.2 equiv.), 4CzIPN (3.0 mg, 0.0039 mmol, 0.013 equiv.), lighted in 1 W Blue LED for 2 h, then 60 °C reaction for 3 h. Product **3x** was obtained as a lightly yellow oil (106 mg, 66% yield).

TLC: R_f = 0.4 (Petroleum ether /ethyl acetate 2:1).

^{1}H NMR (400 MHz, $CDCl_3$) δ 7.62 (d, J = 8.0 Hz, 2H), 7.59 (d, J = 1.6 Hz, 1H), 7.46 (d, J = 8.7 Hz, 1H), 7.27 – 7.19 (m, 3H), 7.13 (d, J = 8.1 Hz, 2H), 6.95 – 6.89 (m, 1H), 6.85 (d, J = 8.0 Hz, 2H), 6.06 (dd, J = 17.4, 10.8 Hz, 1H), 4.86 – 4.80 (m, 2H), 4.74 (d, J = 17.4 Hz, 1H), 3.64 (dd, J = 14.0, 6.4 Hz, 1H), 3.49 (dd, J = 14.0, 6.6 Hz, 1H), 2.69 (s, 3H), 2.33 (s, 3H), 0.93 (s, 3H), 0.54 (s, 3H).

^{13}C NMR (101 MHz, $CDCl_3$) δ 174.2, 147.8, 144.2, 143.6, 140.1, 136.7, 133.2, 129.6, 129.3, 129.1, 128.7, 128.2, 127.0, 121.6, 117.1, 115.1, 114.5, 66.0, 64.5, 56.4, 43.7, 42.3, 24.2, 22.0, 21.5.

HRMS: m/z $[M+H]^+$ calcd for $C_{29}H_{32}{}^{35}ClN_2O_4S$: 539.1766; found: 539.1761.

HRMS: m/z $[M+H]^+$ calcd for $C_{29}H_{32}{}^{37}ClN_2O_4S$: 541.1736; found: 541.1729.

5-bromo-2-((methyl(phenyl)amino)methyl)-3-(2-methylbut-3-en-2-yl)-1-tosylindoline-3-carboxylic acid (3y)

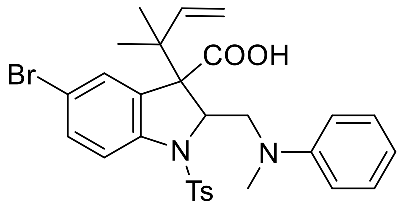

Following the general procedure, 3-methylbut-2-en-1-yl-5-bromo-1-tosyl-1*H*-indole-3-carboxylate (114.9 mg, 0.3 mmol, 1.0 equiv.), *N*-methyl-*N*-((trimethylsilyl)methyl)aniline (77.0 mg, 0.36 mmol, 1.2 equiv.), 4CzIPN (3.0 mg, 0.0039 mmol, 0.013 equiv.), lighted in 1 W Blue LED for 2 h, then 60 °C reaction for 3 h. Product **3y** was obtained as a lightly yellow solid (128 mg, 73% yield).

TLC: R_f = 0.3 (Petroleum ether /ethyl acetate 2:1).

^{1}H NMR (400 MHz, $CDCl_3$) δ 7.74 (d, *J* = 1.6 Hz, 1H), 7.62 (d, *J* = 8.3 Hz, 2H), 7.44 – 7.37 (m, 2H), 7.25 – 7.21 (m, 2H), 7.13 (d, *J* = 8.2 Hz, 2H), 6.96 – 6.90 (m, 1H), 6.87 (d, *J* = 8.1 Hz, 2H), 6.04 (dd, *J* = 17.4, 10.8 Hz, 1H), 4.83 – 4.79 (m, 2H), 4.73 (d, *J* = 17.4 Hz, 1H), 3.65 (dd, *J* = 14.0, 6.4 Hz, 1H), 3.48 (dd, *J* = 14.0, 6.7 Hz, 1H), 2.70 (s, 3H), 2.34 (s, 3H), 0.92 (s, 3H), 0.52 (s, 3H).

^{13}C NMR (101 MHz, $CDCl_3$) δ 174.2, 147.8, 144.3, 143.6, 140.6, 136.7, 133.5, 132.1, 131.6, 129.6, 129.2, 127.0, 121.7, 117.2, 115.7, 115.5, 114.5, 65.9, 64.4, 56.3, 43.7, 42.4, 24.2, 21.9, 21.5.

HRMS: m/z $[M+H]^+$ calcd for $C_{29}H_{32}{}^{79}BrN_2O_4S$: 583.1261; found: 583.1262.

HRMS: m/z $[M+H]^+$ calcd for $C_{29}H_{32}{}^{81}BrN_2O_4S$: 585.1241; found: 585.1239.

m.p. 95 – 96 °C.

5-cyano-2-((methyl(phenyl)amino)methyl)-3-(2-methylbut-3-en-2-yl)-1-tosylindoline-3-carboxylic acid (3z)

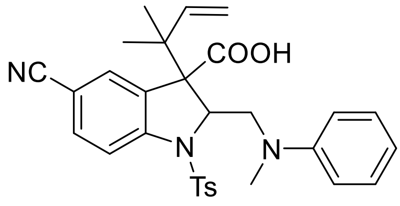

Following the general procedure, 3-methylbut-2-en-1-yl 5-cyano-1-tosyl-1*H*-indole-3-carboxylate (122.0 mg, 0.3 mmol, 1.0 equiv.), *N*-methyl-*N*-((trimethylsilyl)methyl)aniline (70 mg, 0.36 mmol, 1.2 equiv.), 4CzIPN (3.0 mg, 0.0039 mmol, 0.013 equiv.), lighted in 1 W Blue LED for 2

h, then 60 °C reaction for 3 h. Product **X-58** was obtained as a lightly yellow oil (101 mg, 64% yield).

TLC: R_f = 0.2 (Petroleum ether /ethyl acetate 2:1).

^{1}H NMR (400 MHz, $CDCl_3$) δ 7.91 (s, 1H), 7.69 – 7.45 (m, 5H), 7.24 –7.16 (m, 2H), 7.16 (d, *J* = 7.8 Hz, 2H), 7.04 – 6.76 (m, 3H), 6.10 – 5.86 (m, 1H), 4.87 – 4.73 (m, 2H), 4.66 (d, *J* = 17.2 Hz, 1H), 3.68 – 3.54 (m, 1H), 3.53 – 3.41 (m, 1H), 2.69 (s, 3H), 2.31 (s, 3H), 0.89 (s, 3H), 0.49 (s, 3H).

^{13}C NMR (101 MHz, $CDCl_3$) δ 173.4, 147.0, 145.2, 144.8, 143.2, 136.1, 133.4, 133.2, 132.5, 129.7, 129.3, 127.0, 122.7, 119.3, 117.7, 114.8, 114.3, 106.0, 66.0, 64.3, 56.7, 43.7, 42.8, 24.0, 21.8, 21.6.

HRMS: m/z $[M+H]^+$ calcd for $C_{30}H_{32}N_3O_4S$: 530.2108; found: 530.2109.

5-methoxy-2-((methyl(phenyl)amino)methyl)-3-(2-methylbut-3-en-2-yl)-1-tosylindoline-3-carboxylic acid (3aa)

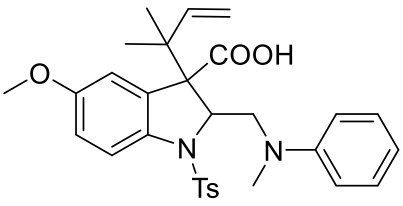

Following the general procedure, 3-methylbut-2-en-1-yl 5-methoxy-1-tosyl-1*H*-indole-3-carboxylate (124 mg, 0.3 mmol, 1.0 equiv.), *N*-methyl-*N*-((trimethylsilyl)methyl)aniline (70 mg, 0.36 mmol, 1.2 equiv.), 4CzIPN (3.0 mg, 0.0039 mmol, 0.013 equiv.), lighted in 1 W Blue LED for 2 h, then 60 °C reaction for 3 h. Product **3aa** was obtained as a lightly yellow solid (86 mg, 54% yield).

TLC: R_f = 0.3 (Petroleum ether /ethyl acetate 2:1).

^{1}H NMR (400 MHz, $CDCl_3$) δ 7.65 (d, *J* = 8.0 Hz, 2H), 7.47 (d, *J* = 8.8 Hz, 1H), 7.25 – 7.18 (m, 3H), 7.13 (d, *J* = 8.1 Hz, 2H), 6.93 – 6.83 (m, 4H), 6.13 (dd, *J* = 17.4, 10.8 Hz, 1H), 4.86 (d, *J* = 9.7 Hz, 2H), 4.80 (d, *J* = 17.4 Hz, 1H), 3.82 (s, 3H), 3.66 (dd, *J* = 14.0, 6.7 Hz, 1H), 3.49 (dd, *J* = 14.0, 6.6 Hz, 1H), 2.72 (s, 3H), 2.34 (s, 3H), 0.96 (s, 3H), 0.59 (s, 3H).

^{13}C NMR (101 MHz, $CDCl_3$) δ 175.0, 155.7, 148.0, 144.0, 143.8, 137.1, 135.0, 132.7, 129.4, 129.1, 127.0, 121.1, 116.7, 115.3, 114.8, 114.3, 114.1, 66.1, 64.3, 56.5, 55.8, 43.7, 42.0, 24.3, 22.1, 21.5.

HRMS: m/z $[M+H]^+$ calcd for $C_{30}H_{35}N_2O_5S$: 535.2261; found: 535.2262.

m.p. 164 – 165 °C.

5-methyl-2-((methyl(phenyl)amino)methyl)-3-(2-methylbut-3-en-2-yl)-1-tosylindoline-3-carboxylic acid (3ab)

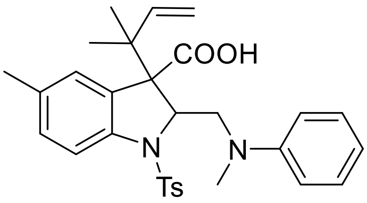

Following the general procedure, 3-methylbut-2-en-1-yl 5-methyl-1-tosyl-1*H*-indole-3-carboxylate (119.1 mg, 0.3 mmol, 1.0 equiv.), *N*-methyl-*N*-((trimethylsilyl)methyl)aniline (70 mg, 0.36 mmol, 1.2 equiv.), 4CzIPN (3.0 mg, 0.0039 mmol, 0.013 equiv.), lighted in 1 W Blue LED for 2 h, then 60 °C reaction for 3 h. Product **3ab** was obtained as a lightly yellow solid (81 mg, 52% yield).

TLC: R_f = 0.3 (Petroleum ether /ethyl acetate 2:1).

^{1}H NMR (400 MHz, $CDCl_3$) δ 7.64 (d, *J* = 8.3 Hz, 2H), 7.41 (d, *J* = 8.2 Hz, 1H), 7.36 (s, 1H), 7.22 – 7.16 (m, 2H), 7.12 – 7.08 (m, 3H), 6.90 – 6.86 (m, 1H), 6.83 (d, *J* = 8.1 Hz, 2H), 6.09 (dd, *J* = 17.4, 10.8 Hz, 1H), 4.87 – 4.74 (m, 3H), 3.63 (dd, *J* = 14.0, 6.6 Hz, 1H), 3.44 (dd, *J* = 13.9, 6.8 Hz, 1H), 2.69 (s, 3H), 2.33 (s, 3H), 2.31 (s, 3H), 0.94 (s, 3H), 0.52 (s, 3H).

^{13}C NMR (101 MHz, $CDCl_3$) δ 175.4, 148.3, 144.1, 143.8, 139.1, 137.2, 132.5, 131.2, 129.6, 129.5, 129.5, 129.1, 127.0, 121.0, 116.8, 114.2, 114.1, 65.9, 64.3, 56.3, 43.7, 42.0, 24.3, 22.0, 21.5, 21.3.

HRMS: m/z $[M+H]^+$ calcd for $C_{30}H_{35}N_2O_4S$: 519.2312; found: 519.2313.

m.p. 179 – 180 °C.

6-methoxy-2-((methyl(phenyl)amino)methyl)-3-(2-methylbut-3-en-2-yl)-1-tosylindoline-3-carboxylic acid (3ac)

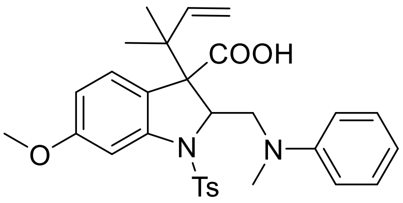

Following the general procedure, 3-methylbut-2-en-1-yl 6-methoxy-1-tosyl-1*H*-indole-3-carboxylate (124 mg, 0.3 mmol, 1.0 equiv.), *N*-methyl-*N*-((trimethylsilyl)methyl)aniline (70 mg, 0.36 mmol, 1.2 equiv.), 4CzIPN (3.0 mg, 0.0039 mmol, 0.013 equiv.), lighted in 1 W

Blue LED for 2 h, then 60 °C reaction for 3 h. Product **3ac** was obtained as a lightly yellow oil (74 mg, 46% yield).

TLC: R_f = 0.3 (Petroleum ether /ethyl acetate 2:1).

^{1}H NMR (400 MHz, $CDCl_3$) δ 7.66 (d, *J* = 8.0 Hz, 2H), 7.42 (d, *J* = 8.6 Hz, 1H), 7.21 – 7.15 (m, 2H), 7.13 – 7.07 (m, 3H), 6.88 – 6.76 (m, 3H), 6.59 (dd, *J* = 8.6, 1.4 Hz, 1H), 6.05 (dd, *J* = 17.5, 10.9 Hz, 1H), 4.87 – 4.71 (m, 3H), 3.83 (s, 3H), 3.62 (dd, *J* = 14.0, 6.5 Hz, 1H), 3.46 (dd, *J* = 14.0, 6.5 Hz, 1H), 2.68 (s, 3H), 2.32 (s, 3H), 0.89 (s, 3H), 0.51 (s, 3H).

^{13}C NMR (101 MHz, $CDCl_3$) δ 175.4, 160.3, 148.5, 144.0, 144.0, 142.6, 137.0, 129.5, 129.0, 127.1, 123.4, 120.8, 116.7, 114.1, 108.3, 100.8, 65.1, 64.9, 56.1, 55.6, 43.8, 42.0, 24.1, 22.0, 21.5.

HRMS: m/z $[M+H]^+$ calcd for $C_{30}H_{35}N_2O_5S$: 535.2261; found: 535.2263.

6-methyl-2-((methyl(phenyl)amino)methyl)-3-(2-methylbut-3-en-2-yl)-1-tosylindoline-3-carboxylic acid (3ad)

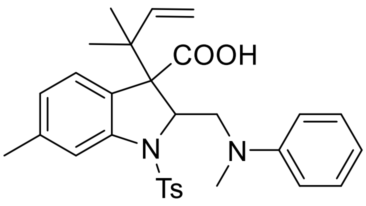

3ad

Following the general procedure, 3-methylbut-2-en-1-yl 6-methyl-1-tosyl-1*H*-indole-3-carboxylate (119.1 mg, 0.3 mmol, 1.0 equiv.), *N*-methyl-*N*-((trimethylsilyl)methyl)aniline (70 mg, 0.36 mmol, 1.2 equiv.), 4CzIPN (3.0 mg, 0.0039 mmol, 0.013 equiv.), lighted in 1 W Blue LED for 2 h, then 60 °C reaction for 3 h. Product **3ad** was obtained as a yellow solid (90 mg, 58% yield).

Due to its low solubility in the deuterium solvents, product **3ad** was derived via a convenient quantitative methyl-esterification. To a solution of **3ad** (1.0 equiv.) in MeOH and Et_2O (v:v = 1:4) was added $TMSCHN_2$ (2.0 M in hexanes, 5.0 equiv.) slowly at room temperature. After stirring for 1 h, all volatiles were removed under reduced pressure and the crude was subsequently purified by column chromatography (Petroleum ether / ethyl acetate = 5: 1) to afford product **3ad-OMe**.

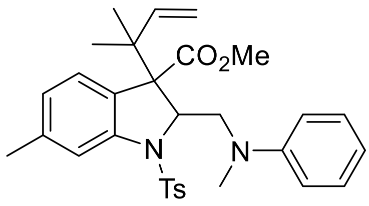

3ad-OMe

TLC: R_f = 0.4 (Petroleum ether /ethyl acetate 20:1). **^{1}H NMR** (400 MHz, $CDCl_3$) δ 7.71 (d, *J* = 8.4 Hz, 2H), 7.36 – 7.30 (m, 2H), 7.15 – 7.07 (m, 4H), 6.92 (dd, *J* = 7.7, 1.6 Hz, 1H), 6.73 – 6.67 (m, 1H), 6.52 (d, *J* = 8.2 Hz, 2H), 6.19 (dd, *J* = 17.5, 10.9 Hz, 1H), 5.15 (dd, *J* = 7.6, 5.7 Hz, 1H), 5.06 (d, *J* = 10.8 Hz, 1H), 4.99 (d, *J* = 17.5 Hz, 1H), 3.51 (s, 3H), 3.38 – 3.28 (m, 2H), 2.58 (s, 3H), 2.44 (s, 3H), 2.32 (s, 3H), 0.98 (s, 3H), 0.85 (s, 3H).

^{13}C NMR (101 MHz, $CDCl_3$) δ 171.5, 150.6, 144.0, 143.4, 142.0, 138.9, 137.9, 129.31, 128.7, 128.6, 128.3, 126.9, 123.9, 117.8, 115.6, 114.3, 65.8, 64.7, 55.0, 51.7, 44.0, 39.7, 24.1, 22.5, 21.9, 21.5.

HRMS: m/z $[M+H]^+$ calcd for $C_{31}H_{37}N_2O_4S$: 533.2469; found: 533.2468.

2-((methyl(phenyl)amino)methyl)-3-(2-methylbut-3-en-2-yl)-1-tosyl-2,3-dihydro-1H-pyrrolo[2,3-b]pyridine-3-carboxylic acid (3ae)

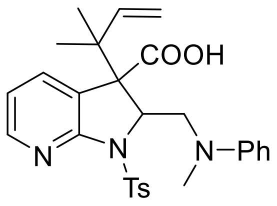

Following the general procedure, 3-methylbut-2-en-1-yl 1-tosyl-1*H*-pyrrolo[2,3-b]pyridine-3-carboxylate (115.8 mg, 0.3 mmol, 1.0 equiv.), *N*-methyl-*N*-((trimethylsilyl)methyl)aniline (70 mg, 0.36 mmol, 1.2 equiv.), 4CzIPN (3.0 mg, 0.0039 mmol, 0.013 equiv.), lighted in 1 W Blue LED for 2 h, then 60 °C reaction for 3 h. Product **3ae** was obtained as a lightly yellow solid (106 mg, 70% yield).

TLC: R_f = 0.2 (Petroleum ether /ethyl acetate 2:1).

^{1}H NMR (400 MHz, $CDCl_3$) δ 8.28 (d, *J* = 5.0 Hz, 1H), 8.00 (d, *J* = 8.0 Hz, 2H), 7.94 (d, *J* = 7.7 Hz, 1H), 7.15 – 7.05 (m, 4H), 6.97 – 6.92 (m, 1H), 6.88 – 6.82 (m, 1H), 6.70 (d, *J* = 8.0 Hz, 2H), 6.01 (dd, *J* = 17.3, 10.8 Hz, 1H), 5.11 – 5.03 (m, 1H), 4.98 – 4.84 (m, 2H), 3.38 (d, *J* = 6.8 Hz, 2H), 2.56 (s, 3H), 2.30 (s, 3H), 1.06 (s, 3H), 0.92 (s, 3H).

^{13}C NMR (101 MHz, $CDCl_3$) δ 173.7, 154.9, 148.1, 147.9, 143.6, 142.9, 138.0, 137.6, 129.1, 129.0, 127.8, 124.6, 121.3, 118.1, 116.6, 115.0, 64.1, 62.2, 56.7, 44.2, 41.3, 23.7, 22.3, 21.6.

HRMS: m/z $[M+H]^+$ calcd for $C_{28}H_{32}N_3O_4S$: 506.2108; found: 506.2106.

2-((4-(1-ethyl-6-fluoro-3-(methoxycarbonyl)-4-oxo-1,4-dihydroquinolin-7-yl)piperazin-1-yl)methyl)-3-(2-methylbut-3-en-2-yl)-1-tosylindoline-3-carboxylic acid (3da)

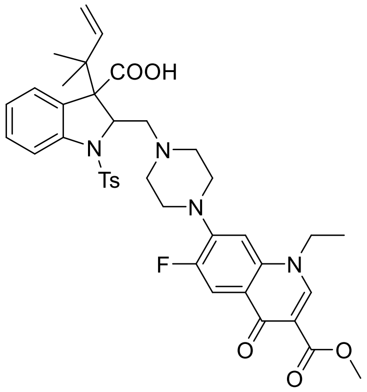

Following the general procedure, 3-methylbut-2-en-1-yl-1-tosyl-1*H*-indole-3-car-boxylate (76.7 mg, 0.2 mmol, 1.0 equiv.), methyl 1-ethyl-6-fluoro-4-oxo-7-(4-((trimeth-ylsilyl)methyl)piperazin-1-yl)-1,4-dihydroqui-noline-3-carboxylate (101.0 mg, 0.24 mmol, 1.2 equiv.), 4CzIPN (2.0 mg, 0.0026 mmol, 0.013 equiv.), lighted in 1 W Blue LED for 2 h, then 60 °C reaction for 3 h. Product **3da** was obtained as a white solid (61 mg, 42% yield).

TLC: R_f = 0.4 (Dichloromethane / methanol 10:1).

^{1}H NMR (400 MHz, $CDCl_3$) δ 8.52 (s, 1H), 8.18 (d, *J* = 12.8 Hz, 1H), 7.97 (d, *J* = 7.7 Hz, 1H), 7.84 (d, *J* = 7.9 Hz, 2H), 7.57 (d, *J* = 7.8 Hz, 1H), 7.37 – 7.31 (m, 3H), 7.14 – 7.10 (m, 1H), 6.83 (d, *J* = 5.8 Hz, 1H), 6.23 (dd, *J* = 17.3, 10.6 Hz, 1H), 5.08 (d, *J* = 10.8 Hz, 1H), 4.91 (d, *J* = 17.8 Hz, 1H), 4.65 (d, *J* = 10.3 Hz, 1H), 4.28 – 4.22 (m, 2H), 3.97 (s, 3H), 3.64 – 3.22 (m, 7H), 3.13 – 3.05 (m, 3H), 2.46 (s, 3H), 1.60 (t, *J* = 7.1 Hz, 3H), 1.08 (s, 3H), 0.20 (s, 3H).

^{13}C NMR (101 MHz, $CDCl_3$) δ173.0, 166.5, 154.5, 148.5, 145.3, 145.0, 140.4, 136.2, 136.1, 130.5, 130.0, 128.9, 127.2, 123.3, 114.3, 114.2, 114.1, 112.8, 110.5, 104.5, 68.6, 60.2, 52.2, 49.1, 48.5, 44.0, 27.2, 24.7, 22.7, 21.6, 20.6, 14.5, 14.1.

^{19}F NMR (376 MHz, $CDCl_3$) δ –123.8.

HRMS: m/z $[M+H]^+$ calcd for $C_{39}H_{44}FN_4O_7S$: 731.2909; found: 731.2911.

m.p. 233 – 234 °C.

3-(2-methylbut-3-en-2-yl)-2-(((1R,5S)-8-oxo-1,5,6,8-tetrahydro-2H-1,5-methanopyrido[1,2-*a*][1,5]diazocin-3(4*H*)-yl)methyl)-1-tosylindoline-3-carboxylic acid (3db)

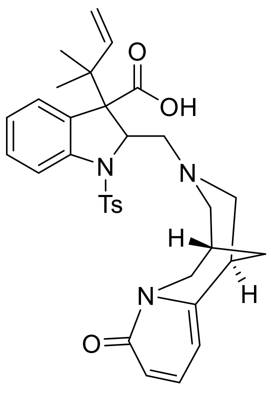

Following the general procedure, 3-methylbut-2-en-1-yl-1-tosyl-1*H*-indole-3-carboxylate (115.0 mg, 0.3 mmol, 1.0 equiv.), (1*R*,5*R*)-3-((trimethylsilyl)methyl)-1,2,3,4,5,6-hexahydro-8*H*-1,5-methanopyrido*[1,2-a][1,5]*diazocin-8-one (99.0 mg, 0.36 mmol, 1.2 equiv.), 4CzIPN (3.0 mg, 0.0039 mmol, 0.013 equiv.), lighted in 1 W Blue LED for 2 h, then 60 °C reaction for 3 h. Product **3db** was obtained as a white solid (60 mg, 34% yield).

TLC: R_f = 0.3 (Dichloromethane / methanol 20:1).

^{1}H NMR (400 MHz, $CDCl_3$) δ 7.81 (d, *J* = 8.2 Hz, 2H), 7.45 – 7.35 (m, 3H), 7.33 – 7.27 (m, 2H), 7.22 – 7.18 (m, 1H), 7.01 – 6.95 (m, 1H), 6.60 (d, *J* = 8.8 Hz, 1H), 6.18 (d, *J* = 6.7 Hz, 1H), 5.75 (dd, *J* = 17.1, 10.9 Hz, 1H), 4.77 (d, *J* = 10.9 Hz, 1H), 4.70 (d, *J* = 17.4 Hz, 1H), 4.46 (d, *J* = 7.9 Hz, 1H), 4.24 (d, *J* = 15.5 Hz, 1H), 3.86 (dd, *J* = 15.5, 6.6 Hz, 1H), 3.45 (d, *J* = 10.5 Hz, 1H), 3.10 – 2.98 (m, 3H), 2.82 (d, *J* = 12.9 Hz, 1H), 2.46 (d, *J* = 10.7 Hz, 1H), 2.42 – 2.36 (m, 5H), 1.86 – 1.78 (m, 2H), 0.77 (s, 3H), 0.34 (s, 3H).

^{13}C NMR (101 MHz, $CDCl_3$) δ 171.5, 164.0, 150.6, 144.7, 144.3, 141.0, 138.8, 137.3, 131.6, 129.8, 129.1, 128.4, 127.1, 122.6, 117.0, 113.9, 112.6, 106.0, 65.4, 63.5, 61.0, 60.9, 59.4, 49.5, 43.6, 35.1, 28.0, 25.9, 23.7, 21.8, 21.6.

HRMS: m/z $[M+H]^+$ calcd for $C_{33}H_{38}N_3O_5S$: 588.2527; found: 588.2528.

m.p. 160 – 161 °C.

2-(((3-(10,11-dihydro-5H-dibenzo[*a,d*][7]annulen-5-ylidene)propyl)(methyl)-amino)methyl)-3-(2-methylbut-3-en-2-yl)-1-tosylindoline-3-carboxylic acid (3dc)

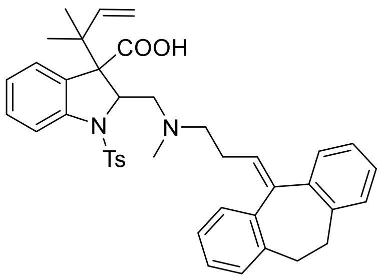

Following the general procedure, 3-methylbut-2-en-1-yl-1-tosyl-1*H*-indole-3-car-boxylate (115.0 mg, 0.3 mmol, 1.0 equiv.), 3-(10,11-dihydro-5*H*-dibenzo[*a,d*][7]annulen-5-ylidene)-*N*-methyl-*N*-((trimethylsilyl)meth-yl)propan-1-amine (125.6 mg, 0.36 mmol, 1.2 equiv.), 4CzIPN (3.0 mg, 0.0039 mmol, 0.013 equiv.), lighted in 1W Blue

LED for 2h, then 60 °C reaction for 3 h. Product **3dc** was obtained as a yellow oil (42 mg, 21% yield).

TLC: R_f = 0.2 (Dichloromethane / methanol 30:1).

^{1}H NMR (400 MHz, $CDCl_3$) δ 7.98 (d, *J* = 7.7 Hz, 1H), 7.77 (d, *J* = 8.1 Hz, 2H), 7.53 (d, *J* = 7.8 Hz, 1H), 7.29 (d, *J* = 8.4 Hz, 2H), 7.27 – 7.20 (m, 4H), 7.20 – 7.16 (m, 3H), 7.12 – 7.05 (m, 3H), 6.41 – 6.19 (m, 1H), 5.88 – 5.68 (m, 1H), 5.04 – 4.83 (m, 1H), 4.73 (dd, *J* = 17.9, 9.4 Hz, 1H), 4.50 (d, *J* = 10.1 Hz, 1H), 3.41 – 3.25 (m, 3H), 3.05 – 2.91 (m, 3H), 2.91 – 2.71 (m, 2H), 2.68 – 2.48 (s, 5H), 2.41 (s, 3H), 1.08 (s, 3H), -0.01 (s, 3H).

^{13}C NMR (101 MHz, $CDCl_3$) δ 172.5, 146.9, 146.7, 145.0, 140.2, 140.0, 139.2, 139.1, 137.1, 136.0, 131.4, 130.9, 130.2, 130.0, 128.5, 128.3, 128.2, 127.7, 127.2, 126.3, 126.2, 124.2, 123.3, 113.1, 112.4, 68.8, 60.5, 43.4, 33.7, 32.0, 25.1, 24.4, 21.6, 20.4.

HRMS: m/z $[M+H]^+$ calcd for $C_{41}H_{45}N_2O_4S$: 661.3095; found: 661.3096.

2-((methyl((*R*)-4-(naphthalen-1-yloxy)-4-(thiophen-2-yl)butyl)amino)methyl)-3-(2-methylbut-3-en-2-yl)-1-tosylindoline-3-carboxylic acid (3dd)

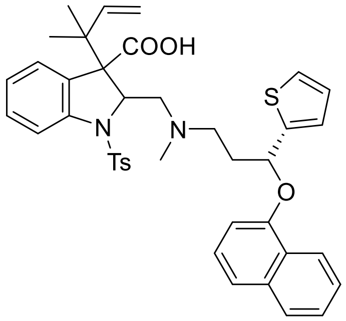

Following the general procedure, 3-methylbut-2-en-1-yl-1-tosyl-1*H*-indole-3-carboxylate (115.0 mg, 0.3 mmol, 1.0 equiv.), (*R*)-*N*-methyl-4-(naphthalen-1-yloxy)-4-(thiophen-2-yl)-*N*-((trimethylsilyl) methyl)butan-1-amine (115.0 mg, 0.36 mmol, 1.2 equiv.), 4CzIPN (3.0 mg, 0.0039 mmol, 0.013 equiv.), lighted in 1 W Blue LED for 2 h, then 60 °C reaction for 3 h. Product **3dd** was obtained as a yellow oil (83 mg, 40% yield, 1:1 d.r.).

TLC: R_f = 0.2 (Dichloromethane / methanol 30:1).

^{1}H NMR (400 MHz, $CDCl_3$) δ 8.34 – 8.26 (m, 1H), 8.01 – 7.95 (m, 1H), 7.80 – 7.76 (m, 1H), 7.73 (d, *J* = 8.3 Hz, 1H), 7.69 (d, *J* = 8.0 Hz, 1H), 7.57 – 7.49 (m, 2H), 7.46 – 7.38 (m, 2H), 7.29 – 7.27 (m, 0.5H), 7.25 – 7.19 (m, 3.5H), 7.17 (d, *J* = 8.0 Hz, 1H), 7.10 – 7.03 (m, 2H), 6.96 – 6.90 (m, 1H), 6.85 – 6.79 (m, 1H), 6.32 – 6.16 (m, 1H), 5.85 – 5.75 (m, 0.5H), 5.74 – 5.68 (m, 0.5H), 4.96 – 4.74 (m, 1H), 4.70 – 4.56 (m, 1H), 4.49 (d, *J* = 10.7 Hz, 1H), 3.49 – 3.26 (m, 1.5H), 3.25 – 2.98 (m, 2.5H), 2.86 – 2.66 (m, 3H), 2.62 – 2.50

(m, 1.5H), 2.41– 2.33 (m, 3.5H), 1.00 (s, 1.5H), 0.97 (s, 1.5H), 0.00 (s, 1.5H), -0.04 (s, 1.5H).

^{13}C NMR (101 MHz, $CDCl_3$) δ 172.4, 152.6, 152.5, 146.4, 146.3, 144.9, 143.2, 140.0, 135.9, 134.6, 134.5, 131.5, 131.3, 130.9, 129.9, 128.4, 128.3, 127.7, 127.6, 127.2, 127.1, 127.0, 126.6, 125.9, 125.7, 125.6, 125.4, 125.3, 125.2, 123.3, 123.2, 122.0, 121.8, 121.3, 121.2, 113.3, 113.1, 112.5, 107.5, 107.2, 73.9, 73.8, 68.8, 60.6, 60.5, 60.0, 59.8, 43.5, 43.4, 40.0, 33.3, 32.9, 25.1, 25.0, 21.6, 20.4, 20.3.

HRMS: m/z $[M+H]^+$ calcd for $C_{40}H_{43}N_2O_5S_2$: 695.2608; found: 695.2609.

2-((2-((benzyloxy)carbonyl)hexahydrocyclopenta[b]pyrrol-1(2H)-yl)methyl)-3-(2-methylbut-3-en-2-yl)-1-tosylindoline-3-carboxylic acid (3de)

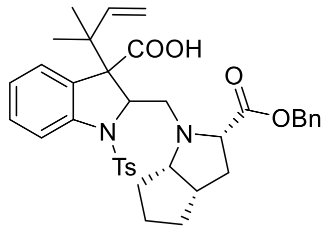

Following the general procedure, 3-methylbut-2-en-1-yl-1-tosyl-1*H*-indole-3-carboxylate (115.0 mg, 0.3 mmol, 1.0 equiv.), benzyl-1-((trimethylsilyl)methyl)-octahydrocyclopenta[b]pyrole-2-carboxylate (119.2 mg, 0.36 mmol, 1.2 equiv.), 4CzIPN (3.0 mg, 0.0039 mmol, 0.013 equiv.), lighted in 1 W Blue LED for 2 h, then 60 °C reaction for 3 h. Product **3de** was obtained as a pink oil (137 mg, 71% yield, 1:1 d.r.).

TLC: R_f = 0.2 (Dichloromethane / methanol 30:1).

^{1}H NMR (400 MHz, $CDCl_3$) δ 7.99 (d, J = 7.6 Hz, 1H), 7.78 (d, J = 8.3 Hz, 2H), 7.51 – 7.45 (m, 3H), 7.44 – 7.36 (m, 3H), 7.30 – 7.28 (m, 1H), 7.26 – 7.24 (m, 1H), 7.23– 7.19 (m, 1H), 7.07 – 7.03 (m, 1H), 6.04 (dd, J = 17.4, 10.8 Hz, 1H), 5.32 – 5.24 (m, 2H), 4.67 – 4.57 (m, 2H), 4.39 (d, J = 8.7 Hz, 1H), 4.32 – 4.24 (m, 1H), 3.53 (q, J = 7.6 Hz, 1H), 3.46 (d, J = 13.1 Hz, 1H), 3.18 (dd, J = 13.2, 9.9 Hz, 1H), 2.73 – 2.62 (m, 1H), 2.61 – 2.51 (m, 1H), 2.41 (s, 3H), 2.14 – 2.05 (m, 1H), 1.98 – 1.85 (m, 2H), 1.75 – 1.65 (m, 1H), 1.61 – 1.49 (m, 1H), 1.39 – 1.29 (m, 1H), 1.21 – 1.11 (m, 1H), 0.97 (s, 3H), 0.22 (s, 3H).

^{13}C NMR (101 MHz, $CDCl_3$) δ 171.9, 171.3, 145.2, 144.7, 140.2, 136.4, 134.8, 130.6, 130.6, 129.8, 129.1, 128.8, 128.7, 128.4, 127.2, 123.0, 113.2, 112.3, 74.8, 68.1, 67.7, 65.6, 62.9, 60.3, 43.7, 42.0, 34.3, 32.1, 30.7, 25.2, 24.6, 21.6, 21.3.

HRMS: m/z $[M+H]^+$ calcd for $C_{37}H_{43}N_2O_6S$: 643.2836; found: 643.2837.

2-((4-(bis(4-fluorophenyl)methyl)piperazin-1-yl)methyl)-3-(2-methylbut-3-en-2-yl)-1-tosylindoline-3-carboxylic acid (3df)

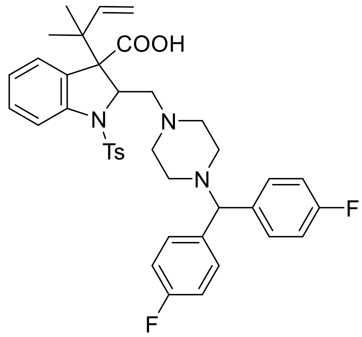

Following the general procedure, 3-methylbut-2-en-1-yl-1-tosyl-1*H*-indole-3-carboxylate (115.0 mg, 0.3 mmol, 1.0 equiv.), 1-(bis(4-fluorophenyl)methyl)-4-((trimethylsilyl)methy-l)piperazine (134.6 mg, 0.36 mmol, 1.2 equiv.), 4CzIPN (3.0 mg, 0.0039 mmol, 0.013 equiv.), lighted in 1 W Blue LED for 2 h, then 60 °C reaction for 3 h. Product **3df** was obtained as a white solid (150 mg, 73% yield).

TLC: R_f = 0.2 (Dichloromethane / methanol 30:1).

^{1}H NMR (400 MHz, $CDCl_3$) δ 7.95 (d, *J* = 7.1 Hz, 1H), 7.76 (d, *J* = 8.3 Hz, 2H), 7.53 (d, *J* = 8.0 Hz, 1H), 7.39 – 7.30 (m, 4H), 7.29 – 7.21 (m, 3H), 7.07 – 6.96 (m, 5H), 6.27 (dd, *J* = 17.6, 10.8 Hz, 1H), 4.94 (d, *J* = 10.9, 1.2 Hz, 1H), 4.73 (dd, *J* = 17.6, 1.2 Hz, 1H), 4.53 – 4.47 (m, 1H), 4.28 (s, 1H), 3.93 – 3.43 (br, 1H), 3.41 (d, *J* = 11.6 Hz, 1H), 3.10 – 2.60 (br, 6H), 2.47 – 2.15 (br, 5H), 1.01 (s, 3H), -0.02 (s, 3H).

^{13}C NMR (101 MHz, $CDCl_3$) δ 172.0, 162.1 (d, $^1J_{C\text{-}F}$ = 245.1 Hz), 146.2, 144.9, 140.1, 137.2 (d, $^4J_{C\text{-}F}$ = 3.0 Hz), 137.0 (d, $^4J_{C\text{-}F}$ = 2.9 Hz), 136.0, 130.9, 130.8, 129.9, 129.1 (d, $^3J_{C\text{-}F}$ = 7.9 Hz), 129.0 (d, $^3J_{C\text{-}F}$ = 8.0 Hz), 128.5, 127.2, 123.3, 115.8 (d, $^2J_{C\text{-}F}$ = 21.2 Hz), 113.4, 112.6, 73.9, 69.0, 60.1, 59.7, 49.6, 43.6, 25.0, 21.6, 20.3.

^{19}F NMR (376 MHz, $CDCl_3$) δ –114.5, –114.6.

HRMS: m/z $[M+H]^+$ calcd for $C_{39}H_{42}F_2N_3O_4S$: 686.2859; found: 686.2859.

m.p. 152 – 153 °C.

2-((6,7-dihydrothieno[3,2-c]pyridin-5(4*H*)-yl)methyl)-3-(2-methylbut-3-en-2-yl)-1-tosylindoline-3-carboxylic acid (3dg)

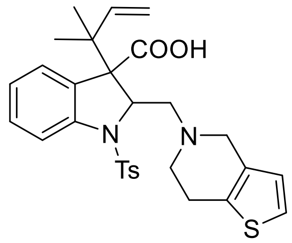

Following the general procedure, 3-methylbut-2-en-1-yl-1-tosyl-1*H*-indole-3-carboxylate (115.0 mg, 0.3 mmol, 1.0 equiv.), 5-((trimethylsilyl)methyl)-4,5,6,7-tetrahydro-thieno*[3,2-c]*pyridine (81.0 mg, 0.36 mmol, 1.2 equiv.), 4CzIPN (3.0 mg, 0.0039 mmol, 0.013 equiv.), lighted in 1 W Blue LED for 2

h, then 60 °C reaction for 3 h. Product **3dg** was obtained as a yellow oil (88 mg, 55% yield).

TLC: R_f = 0.4 (Dichloromethane / methanol 30:1).

^{1}H NMR (400 MHz, $CDCl_3$) δ 7.89 (d, *J* = 7.4 Hz, 1H), 7.79 – 7.73 (m, 2H), 7.51 – 7.45 (m, 1H), 7.29 – 7.21 (m, 3H), 7.17 – 7.13 (m, 1H), 7.06 – 7.00 (m, 1H), 6.73 – 6.65 (m, 1H), 6.27 (dd, *J* = 17.4, 10.8 Hz, 1H), 5.05 (d, *J* = 10.7 Hz, 1H), 4.87 (d, *J* = 17.6 Hz, 1H), 4.70 (d, *J* = 10.0 Hz, 1H), 4.01 (s, 2H), 3.48 – 3.26 (m, 2H), 3.20 – 3.00 (m, 3H), 2.98 – 2.86 (m, 1H), 2.39 (s, 3H), 1.04 (s, 3H), 0.18 (s, 3H).

^{13}C NMR (101 MHz, $CDCl_3$) δ 172.4, 146.0, 144.8, 140.4, 136.5, 132.0, 131.3, 130.6, 129.9, 129.4, 128.5, 127.1, 125.0, 124.5, 123.1, 113.6, 112.8, 68.2, 61.1, 58.8, 51.8, 49.7, 43.8, 24.8, 23.0, 21.6, 20.7.

HRMS: m/z $[M+H]^+$ calcd for $C_{29}H_{33}N_2O_4S_2$: 537.1876; found: 537.1878.

2-((4-((S)-(4-chlorophenyl)(pyridin-2-yl)methoxy)piperidin-1-yl)methyl)-3-(2-methylbut-3-en-2-yl)-1-tosylindoline-3-carboxylic acid (3dh)

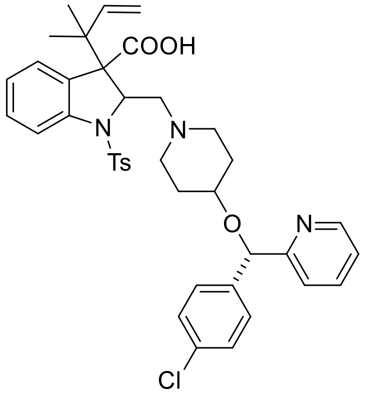

Following the general procedure, 3-methylbut-2-en-1-yl-1-tosyl-1*H*-indole-3-carboxylate (115.0 mg, 0.3 mmol, 1.0 equiv.), 2-((4-chlorophenyl)((1-((trimethylsilyl)methyl)piperidin-4-yl)oxy)methyl)pyridine (139.7 mg, 0.36 mmol, 1.2 equiv.), 4CzIPN (3.0 mg, 0.0039 mmol, 0.013 equiv.), lighted in 1 W Blue LED for 2 h, then 60 °C reaction for 3 h. Product **3dh** was obtained as a yellow oil (78 mg, 37% yield, 2.4:1 d.r.).

TLC: R_f = 0.4 (Dichloromethane / methanol 20:1).

^{1}H NMR (400 MHz, $CDCl_3$) δ 8.45 – 8.43 (m, 1H), 7.82 (d, *J* = 7.5 Hz, 1H), 7.72 (d, *J* = 8.2 Hz, 2H), 7.65 – 7.57 (m, 1H), 7.45 – 7.39 (m, 2H), 7.31 (d, *J* = 8.3 Hz, 2H), 7.24 – 7.18 (m, 4H), 7.17 – 7.05 (m, 2H), 6.99 – 6.93 (m, 1H), 6.33 (dd, *J* = 17.6, 10.8 Hz, 1H), 5.51 (s, 1H), 4.95 (d, *J* = 11.0 Hz, 1H), 4.72 (d, *J* = 17.6 Hz, 1H), 4.51 (d, *J* = 9.1 Hz, 1H), 3.64 – 3.54 (m, 1H), 3.34 – 3.20 (m, 1H), 3.16 – 2.96 (m, 2H), 2.94 – 2.76 (m, 2H), 2.74 –

2.56 (m, 1H), 2.30 (s, 3H), 2.02 – 1.92 (m, 2H), 1.84 – 1.76 (m, 2H), 0.98 (s, 3H), 0.06 (s, 3H).

^{13}C NMR (101 MHz, $CDCl_3$) δ 173.7, 162.6, 162.2, 150.2, 150.1, 147.6, 145.8, 141.5, 141.1, 140.7, 138.3, 138.2, 137.5, 134.7, 134.5, 133.0, 131.7, 131.0, 129.8, 129.7, 129.3, 128.2, 124.2, 124.0, 123.8, 121.7, 121.7, 114.2, 113.9, 82.5, 82.2, 69.8, 61.6, 61.2, 54.0, 50.8, 50.6, 44.6, 30.6, 30.1, 26.1, 22.7, 21.7, 0.3.

HRMS: m/z $[M+H]^+$ calcd for $C_{39}H_{43}{}^{35}ClN_3O_5S$: 700.2606; found: 700.2609.

HRMS: m/z $[M+H]^+$ calcd for $C_{39}H_{43}{}^{37}ClN_3O_5S$: 702.2576; found: 702.2574.

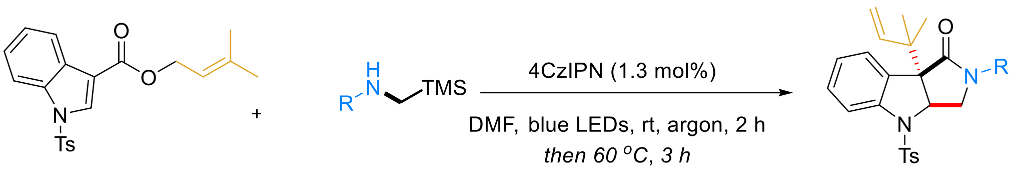

8b-(2-methylbut-3-en-2-yl)-2-phenyl-4-tosyl-3,3a,4,8*b*-tetrahydropyrrolo[*3,4-b*]indol-1(2*H*)-one (5a)

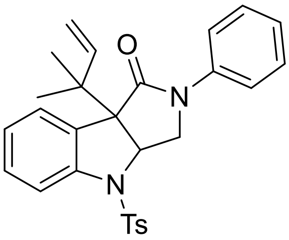

Following the general procedure, 3-methylbut-2-en-1-yl 1-tosyl-1*H*-indole-3-carboxylate (77.0 mg, 0.2 mmol, 1.0 equiv.), *N*-((trimethylsilyl)methyl)aniline (46.3 mg, 0.24 mmol, 1.2 equiv.), 4CzIPN (2.0 mg, 0.0026 mmol, 0.013 equiv.), lighted in 1 W Blue LED for 2 h, then 60 °C reaction for 3 h. Product **5a** was obtained as a yellow solid (59 mg, 63% yield).

TLC: R_f = 0.4 (Petroleum ether /ethyl acetate 20:1).

^{1}H NMR (400 MHz, $CDCl_3$) δ 7.82 (d, *J* = 8.2 Hz, 2H), 7.72 (d, *J* = 8.2 Hz, 1H), 7.67 (d, *J* = 7.7 Hz, 1H), 7.61 (d, *J* = 8.5 Hz, 2H), 7.39 – 7.35 (m, 2H), 7.34 – 7.28 (m, 3H), 7.19 – 7.15 (m, 1H), 7.08 – 7.04 (m, 1H), 5.67 (dd, *J* = 17.2, 10.8 Hz, 1H), 4.85 (d, *J* = 17.4 Hz, 1H), 4.76 (d, *J* = 10.7 Hz, 1H), 4.58 (dd, *J* = 7.4, 2.4 Hz, 1H), 4.2 (dd, *J* = 11.2, 7.5 Hz, 1H), 4.18 (dd, *J* = 11.2, 2.4 Hz, 1H), 2.43 (s, 3H), 1.09 (s, 3H), 0.79 (s, 3H).

^{13}C NMR (101 MHz, $CDCl_3$) δ 171.3, 144.8, 142.9, 142.2, 138.6, 134.8, 130.0, 129.5, 129.0, 128.8, 127.4, 127.2, 125.2, 123.7, 120.5, 113.9, 113.7, 63.2, 61.4, 55.5, 42.9, 22.5, 22.0, 21.7.

HRMS: m/z $[M+H]^+$ calcd for $C_{28}H_{29}N_2O_3S$: 473.1893; found: 473.1895.

m.p. 136 – 137 °C.

2-(4-fluorophenyl)-8*b*-(2-methylbut-3-en-2-yl)-4-tosyl-3,3a,4,8*b*-tetrahydropyrrolo-*[3,4-b]*indol-1(2*H*)-one (5b)

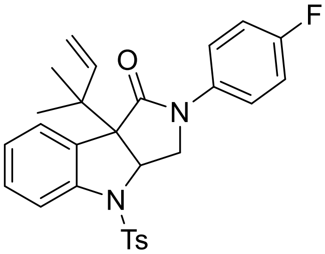

Following the general procedure, 3-methylbut-2-en-1-yl 1-tosyl-1*H*-indole-3-carboxylate (77.0 mg, 0.2 mmol, 1.0 equiv.), 4-fluoro-*N*-((trimethylsilyl)methyl)aniline (47.3 mg, 0.24 mmol, 1.2 equiv.), 4CzIPN (2.0 mg, 0.0026 mmol,

0.013 equiv.), lighted in 1 W Blue LED for 2 h, then 60 °C reaction for 3 h. Product **5b** was obtained as a yellow solid (72 mg, 73% yield).

TLC: R_f = 0.4 (Petroleum ether /ethyl acetate 20:1).

^{1}H NMR (500 MHz, $CDCl_3$) δ 7.81 (d, *J* = 8.3 Hz, 2H), 7.72 (d, *J* = 8.2 Hz, 1H), 7.65 (d, *J* = 7.7 Hz, 1H), 7.59 – 7.55 (m, 2H), 7.35 – 7.29 (m, 3H), 7.07 – 7.02 (m, 3H), 5.65 (dd, *J* = 17.2, 10.8 Hz, 1H), 4.84 (d, *J* = 17.4 Hz, 1H), 4.77 (d, *J* = 10.7 Hz, 1H), 4.57 (dd, *J* = 7.5, 2.5 Hz, 1H), 4.22 (dd, *J* = 11.2, 7.5 Hz, 1H), 4.14 (dd, *J* = 11.2, 2.5 Hz, 1H), 2.43 (s, 3H), 1.08 (s, 3H), 0.78 (s, 3H).

^{13}C NMR (101 MHz, CDCl3) δ 171.2, 159.8 (d, $^1J_{C\text{-}F}$ = 244.0 Hz), 144.8, 142.8, 142.1, 134.7, 134.6 (d, $^4J_{C\text{-}F}$ = 2.4 Hz), 129.9, 129.6, 128.7, 127.4, 127.2, 123.7, 122.3 (d, $^3J_{C\text{-}F}$ = 7.9Hz), 115.6 (d, $^2J_{C\text{-}F}$ = 22.3 Hz), 113.9, 113.7, 63.1, 61.4, 55.8, 42.8, 22.5, 21.9, 21.6.

^{19}F NMR (376 MHz, $CDCl_3$) δ –116.6.

HRMS: m/z $[M+H]^+$ calcd for $C_{28}H_{28}FN_2O_3S$: 491.1799; found: 491.1801.

m.p. 72 – 73 °C.

2-(4-chlorophenyl)-8*b*-(2-methylbut-3-en-2-yl)-4-tosyl-3,3a,4,8*b*-tetrahydropyrrolo-*[3,4-b]*indol-1(2*H*)-one (5c)

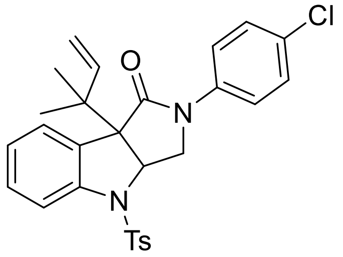

Following the general procedure, 3-methylbut-2-en-1-yl 1-tosyl-1*H*-indole-3-carboxylate (115.0 mg, 0.3 mmol, 1.0 equiv.), 4-chloro-*N*-((trimethyl-silyl)methyl)aniline (76.6 mg, 0.36 mmol, 1.2 equiv.), 4CzIPN (3.0 mg, 0.0039 mmol, 0.013 equiv.), lighted in 1 W Blue LED for 2 h, then 60 °C reaction for 3 h. Product **5c** was obtained as a lightly yellow oil (99 mg, 66% yield).

TLC: R_f = 0.4(Petroleum ether /ethyl acetate 20:1).

^{1}H NMR (400 MHz, $CDCl_3$) δ 7.81 (d, *J* = 8.3 Hz, 2H), 7.72 (d, *J* = 8.2 Hz, 1H), 7.65 (d, *J* = 7.7 Hz, 1H), 7.60 – 7.56 (m, 2H), 7.35 – 7.29 (m, 5H), 7.09 – 7.03 (m, 1H), 5.64 (dd, *J* = 17.3, 10.7 Hz, 1H), 4.84 (d, *J* = 17.4 Hz, 1H), 4.76 (d, *J* = 10.7 Hz, 1H), 4.57 (dd, *J* = 7.4, 2.6 Hz, 1H), 4.22 (dd, *J* = 11.2, 7.4 Hz, 1H), 4.14 (dd, *J* = 11.2, 2.5 Hz, 1H), 2.42 (s, 3H), 1.08 (s, 3H), 0.77 (s, 3H).

^{13}C NMR (101 MHz, $CDCl_3$) δ 171.4, 144.9, 142.7, 142.1, 137.1, 134.7, 130.3, 129.9, 129.6, 128.9, 128.6, 127.4, 127.1, 123.7, 121.5, 114.0, 113.7, 63.2, 61.3, 55.4, 42.8, 22.5, 22.0, 21.6.

HRMS: m/z $[M+H]^+$ calcd for $C_{28}H_{28}ClN_2O_3S$: 507.1504; found: 507.1508.

2-(4-(tert-butyl)phenyl)-8*b*-(2-methylbut-3-en-2-yl)-4-tosyl-3,3a,4,8*b*-tetrahydropy-rrolo*[3,4-b]*indol-1(2*H*)-one (5d)

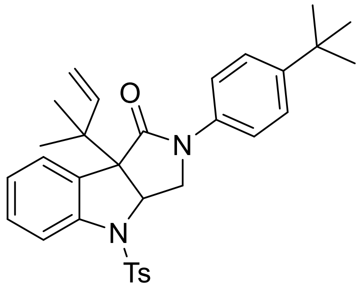

Following the general procedure, 3-methylbut-2-en-1-yl-1-tosyl-1*H*-indole-3-carboxylate (77.0 mg, 0.2 mmol, 1.0 equiv.), 4-(tert-butyl)-*N*-((trimethylsilyl)methy-l)aniline (56.4 mg, 0.24 mmol, 1.2 equiv.), 4CzIPN (2.0 mg, 0.0026 mmol, 0.013 equiv.), lighted in 1 W Blue LED for 2 h, then 60 °C reaction for 3 h. Product **5d** was obtained as a yellow solid (93 mg, 59% yield).

TLC: R_f = 0.4 (Petroleum ether /ethyl acetate 20:1).

^{1}H NMR (400 MHz, $CDCl_3$) δ 7.84 (d, *J* = 6.7 Hz, 2H), 7.74 (d, *J* = 8.2 Hz, 1H), 7.68 (d, *J* = 7.7 Hz, 1H), 7.56 – 7.50 (m, 2H), 7.41 – 7.35 (m, 2H), 7.33 – 7.27 (m, 3H), 7.08 – 7.02 (m, 1H), 5.69 (dd, *J* = 16.6, 11.1 Hz, 1H), 4.86 (d, *J* = 17.4 Hz, 1H), 4.77 (d, *J* = 10.7 Hz, 1H), 4.60 (d, *J* = 7.3 Hz, 1H), 4.26 – 4.21 (m, 1H), 4.18 – 4.12 (m, 1H), 2.45 (s, 3H), 1.33 (s, 9H), 1.11 (s, 3H), 0.81 (s, 3H).

^{13}C NMR (101 MHz, $CDCl_3$) δ 171.1, 148.2, 144.8, 143.0, 142.1, 135.9, 134.9, 129.9, 129.4, 128.8, 127.4, 127.2, 125.8, 123.6, 120.1, 113.7, 113.6, 63.1, 61.5, 55.5, 42.8, 34.4, 31.3, 22.5, 22.0, 21.6.

HRMS: m/z $[M+Na]^+$ calcd for $C_{32}H_{36}N_2NaO_3S$: 551.2339; found: 551.2338.

m.p. 247 – 248 °C.

ethyl-4-(8*b*-(2-methylbut-3-en-2-yl)-1-oxo-4-tosyl-3,3a,4,8*b*-tetrahydropyrrolo*[3,4-b]*indol-2(1*H*)-yl)benzoate (5e)

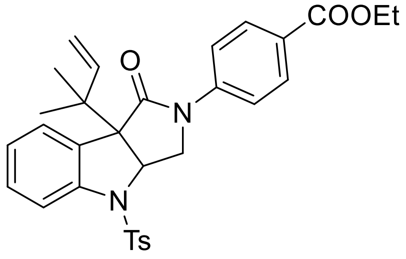

Following the general procedure, 3-methylbut-2-en-1-yl-1-tosyl-1*H*-indole-3-carboxylate (115.0 mg, 0.3 mmol, 1.0 equiv.), ethyl 1-((trimethylsilyl)methyl)piperidine-4-carboxylate (87.5 mg, 0.36 mmol, 1.2 equiv.), 4CzIPN (3.0 mg, 0.0039 mmol, 0.013 equiv.), lighted in 1 W Blue LED for 2 h, then 60 °C reaction for 3 h. Product **5e** was obtained as a yellow solid (103 mg, 63% yield).

TLC: R_f = 0.3 (Petroleum ether /ethyl acetate 2:1).

^{1}H NMR (400 MHz, $CDCl_3$) δ 8.07 (d, *J* = 8.1 Hz, 2H), 7.83 (d, *J* = 7.5 Hz, 2H), 7.78 – 7.72 (m, 3H), 7.68 (d, *J* = 7.5 Hz, 1H), 7.38 – 7.33 (m, 3H), 7.012 – 7.06 (m, 1H), 5.67 (dd, *J* = 16.4, 11.2 Hz, 1H), 4.87 (d, *J* = 17.3 Hz, 1H), 4.80 (d, *J* = 10.5 Hz, 1H), 4.61 (d, *J* = 6.8 Hz, 1H), 4.41 – 4.37 (m, 2H), 4.32 – 4.26 (m, 1H), 4.24 – 4.18 (m, 1H), 2.45 (s, 3H), 1.43 (t, *J* = 6.9 Hz, 3H), 1.11 (s, 3H), 0.80 (s, 3H).

^{13}C NMR (101 MHz, $CDCl_3$) δ 171.8, 166.0, 144.9, 142.6, 142.4, 142.1, 134.7, 130.5, 129.9, 129.7, 128.5, 127.4, 127.1, 126.6, 123.8, 119.2, 114.1, 113.7, 63.4, 61.2, 61.0, 55.3, 42.9, 22.4, 22.0, 21.6, 14.4.

HRMS: m/z $[M+H]^+$ calcd for $C_{31}H_{33}N_2O_5S$: 545.2105; found: 545.2107.

m.p. 183 – 184 °C.

2-(4-bromophenyl)-8*b*-(2-methylbut-3-en-2-yl)-4-tosyl-3,3a,4,8*b*-tetrahydropyrrolo*[3,4-b]*indol-1(2*H*)-one (5f)

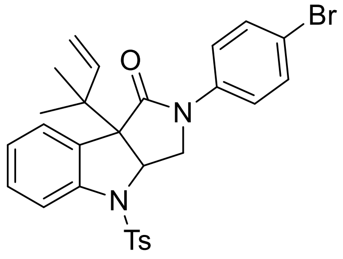

Following the general procedure, 3-methylbut-2-en-1-yl-1-tosyl-1*H*-indole-3-carboxylate (115.0 mg, 0.3 mmol, 1.0 equiv.), 4-bromo-*N*-((trimethylsilyl)-methyl)aniline (92.5 mg, 0.36 mmol, 1.2 equiv.), 4CzIPN (3.0 mg, 0.0039 mmol, 0.013 equiv.), lighted in 1 W Blue LED for 2 h, then 60 °C reaction for 3 h. Product **5f** was obtained as a yellow solid (124 mg, 75% yield).

TLC: R_f = 0.4(Petroleum ether /ethyl acetate 20:1).

^{1}H NMR (400 MHz, $CDCl_3$) δ 7.71 (d, *J* = 7.1 Hz, 2H), 7.63 (d, *J* = 8.1 Hz, 1H), 7.55 (d, *J* = 7.7 Hz, 1H), 7.43 (d, *J* = 7.3 Hz, 2H), 7.37 (d, *J* = 8.9 Hz, 2H), 7.25 – 7.17 (m, 3H), 6.99 – 6.93 (m, 1H), 5.54 (dd, *J* = 16.8, 11.0 Hz, 1H), 4.74 (d, *J* = 17.4 Hz, 1H), 4.67 (d, *J* = 10.6 Hz, 1H), 4.48 (d, *J* = 7.1 Hz, 1H), 4.16 – 4.08 (m, 1H), 4.04 (d, *J* = 11.1 Hz, 1H), 2.32 (s, 3H), 0.98 (s, 3H), 0.68 (s, 3H).

^{13}C NMR (101 MHz, $CDCl_3$) δ 171.4, 144.9, 142.7, 142.1, 137.6, 134.7, 131.9, 129.9, 129.6, 128.6, 127.4, 127.1, 123.7, 121.8, 118.0, 114.0, 113.7, 63.3, 61.3, 55.4, 42.8, 22.5, 22.0, 21.6.

HRMS: m/z $[M+H]^+$ calcd for $C_{28}H_{28}{}^{79}BrN_2O_3S$: 551.0999; found: 551.0997.

HRMS: m/z $[M+H]^+$ calcd for $C_{28}H_{28}{}^{81}BrN_2O_3S$: 553.0978; found: 553.0977.

m.p. 137 – 138 °C.

8*b*-(2-methylbut-3-en-2-yl)-4-tosyl-2-(3-(trifluoromethyl)phenyl)-3,3a,4,8*b*-tetrahydropyrrolo*[3,4-b]*indol-1(2*H*)-one (5g)

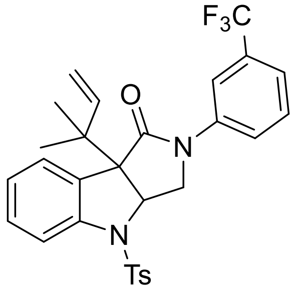

Following the general procedure, 3-methylbut-2-en-1-yl-1-tosyl-1*H*-indole-3-carboxylate (115.0 mg, 0.3 mmol, 1.0 equiv.), 3-(trifluoromethyl)-*N*-((trimethylsilyl)methyl)aniline (88.9 mg, 0.36 mmol, 1.2 equiv.), 4CzIPN (3.0 mg, 0.0039 mmol, 0.013 equiv.), lighted in 1 W Blue LED for 2 h, then 60 °C reaction for 3 h. Product **5g** was obtained as a lightly yellow oil (99 mg, 70% yield).

TLC: R_f = 0.5(Petroleum ether /ethyl acetate 20:1).

^{1}H NMR (400 MHz, $CDCl_3$) δ 7.92 (s, 1H), 7.77 – 7.71 (m, 3H), 7.67 (d, *J* = 8.2 Hz, 1H), 7.60 (d, *J* = 7.7 Hz, 1H), 7.44 – 7.40 (m, 1H), 7.35 (d, *J* = 7.7 Hz, 1H), 7.30 – 7.20 (m, 3H), 7.05 – 6.97 (m, 1H), 5.59 (dd, *J* = 17.1, 10.8 Hz, 1H), 4.79 (d, *J* = 17.4 Hz, 1H), 4.72 (d, *J* = 10.7 Hz, 1H), 4.56 – 4.52 (m, 1H), 4.21 (dd, *J* = 11.1, 7.5 Hz, 1H), 4.14 – 4.10 (m, 1H), 2.36 (s, 3H), 1.03 (s, 3H), 0.71 (s, 3H).

^{13}C NMR (101 MHz, $CDCl_3$) δ 171.7, 145.0, 142.6, 142.2, 139.1, 134.6, 131.4 (q, $^2J_{C\text{-}F}$ = 32.5 Hz), 129.9, 129.7, 129.5, 128.5, 127.4, 127.2, 124.1 (q, $^1J_{C\text{-}F}$ = 271.2 Hz), 123.8,

123.0, 121.6 (q, ${}^{3}J_{C\text{-}F}$ = 4.2 Hz), 116.9 (q, ${}^{3}J_{C\text{-}F}$ = 3.8 Hz), 114.1, 113.7, 63.4, 61.3, 55.3, 42.9, 22.4, 22.0, 21.6.

^{19}F NMR (376 MHz, $CDCl_3$) δ –65.6.

HRMS: m/z $[M+H]^+$ calcd for $C_{29}H_{28}F_3N_2O_3S$: 541.1767; found: 541.1769.

2-(3-methoxyphenyl)-8b-(2-methylbut-3-en-2-yl)-4-tosyl-3,3a,4,8b-tetrahydropyrrolo*[3,4-b]*indol-1(2*H*)-one (5h)

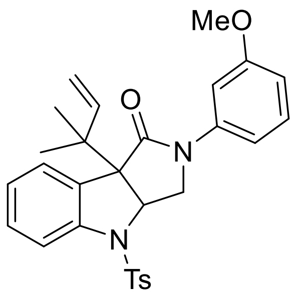

Following the general procedure, 3-methylbut-2-en-1-yl-1-tosyl-1*H*-indole-3-carboxylate (115.0 mg, 0.3 mmol, 1.0 equiv.), 3-methoxy-*N*-((trimethylsilyl)methyl)aniline (75.2 mg, 0.36 mmol, 1.2 equiv.), 4CzIPN (3.0 mg, 0.0039 mmol, 0.013 equiv.), lighted in 1 W Blue LED for 2 h, then 60 °C reaction for 3 h. Product **5h** was obtained as a lightly yellow oil (98 mg, 65% yield).

TLC: R_f = 0.4 (Petroleum ether /ethyl acetate 20:1).

^{1}H NMR (400 MHz, $CDCl_3$) δ 7.80 (d, *J* = 8.3 Hz, 2H), 7.72 (d, *J* = 8.1 Hz, 1H), 7.66 (d, *J* = 7.7 Hz, 1H), 7.37 – 7.33 (m, 1H), 7.34 – 7.29 (m, 3H), 7.27 – 7.22 (m, 1H), 7.11 – 7.02 (m, 2H), 6.72 (dd, *J* = 8.0, 2.2 Hz, 1H), 5.67 (dd, *J* = 17.3, 10.7 Hz, 1H), 4.83 (d, *J* = 17.4 Hz, 1H), 4.76 (d, *J* = 10.8 Hz, 1H), 4.57 (dd, *J* = 7.4, 2.5 Hz, 1H), 4.23 (dd, *J* = 11.3, 7.5 Hz, 1H), 4.15 (dd, *J* = 11.3, 2.6 Hz, 1H), 3.79 (s, 3H), 2.40 (s, 3H), 1.07 (s, 3H), 0.79 (s, 3H).

^{13}C NMR (101 MHz, $CDCl_3$) δ 171.3, 160.0, 144.8, 142.9, 142.2, 139.8, 134.7, 129.9, 129.6, 129.5, 128.8, 127.4, 127.2, 123.7, 113.9, 113.6, 112.4, 111.2, 106.2, 63.4, 61.4, 55.7, 55.4, 42.8, 22.5, 22.0, 21.6.

HRMS: m/z $[M+H]^+$ calcd for $C_{29}H_{31}N_2O_4S$: 503.1999; found: 503.1996.

methyl-2-(8*b*-(2-methylbut-3-en-2-yl)-1-oxo-4-tosyl-3,3a,4,8*b*-tetrahydropyrrolo*[3,4-b]*indol-2(1*H*)-yl)acetate (5i)

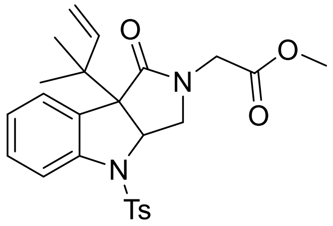

Following the general procedure, 3-methylbut-2-en-1-yl 1-tosyl-1*H*-indole-3-carboxylate (115.0 mg, 0.3 mmol, 1.0 equiv.), methyl ((trimethylsilyl)methyl)glycinate (77.4 mg, 0.36 mmol, 1.2 equiv.), 4CzIPN (3.0 mg, 0.0039 mmol, 0.013 equiv.), lighted in 1 W Blue LED for 2 h, then 60 °C reaction for 3 h. Product **5i** was obtained as a white solid (74 mg, 53% yield).

TLC: R_f = 0.5 (Petroleum ether /ethyl acetate 20:1).

^{1}H NMR (400 MHz, $CDCl_3$) δ 7.78 (d, *J* = 8.3 Hz, 2H), 7.71 (d, *J* = 8.1 Hz, 1H), 7.60 (d, *J* = 7.7 Hz, 1H), 7.33 – 7.28 (m, 3H), 7.05 – 7.01 (m, 1H), 5.71 (dd, *J* = 17.3, 10.7 Hz, 1H), 4.79 (d, *J* = 17.4 Hz, 1H), 4.74 (d, *J* = 10.8 Hz, 1H), 4.51 (dd, *J* = 7.3, 2.0 Hz, 1H), 4.42 (d, *J* = 17.6 Hz, 1H), 4.01 (dd, *J* = 10.8, 7.4 Hz, 1H), 3.74 (s, 3H), 3.68 (dd, *J* = 10.9, 2.0 Hz, 1H), 3.63 (d, *J* = 17.6 Hz, 1H), 2.41 (s, 3H), 1.01 (s, 3H), 0.82 (s, 3H).

^{13}C NMR (101 MHz, $CDCl_3$) δ 172.6, 168.6, 144.7, 143.1, 142.1, 134.7, 129.8, 129.3, 128.8, 127.4, 127.0, 123.5, 113.6, 113.4, 62.4, 62.0, 54.9, 52.3, 44.0, 42.5, 22.5, 21.9, 21.6.

HRMS: m/z $[M+H]^+$ calcd for $C_{25}H_{29}N_2O_5S$: 469.1792; found: 469.1794.

m.p. 50 – 51 °C.

methyl-(2*S*)-2-(8b-(2-methylbut-3-en-2-yl)-1-oxo-4-tosyl-3,3a,4,8b-tetrahydropyrrolo[*3,4-b*]indol-2(1*H*)-yl)-4-(methylthio)butanoate (5j)

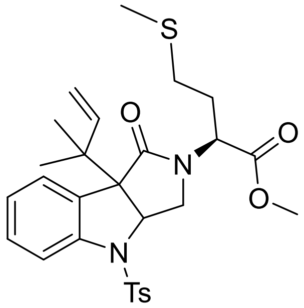

Following the general procedure, 3-methylbut-2-en-1-yl-1-tosyl-1*H*-indole-3-carboxylate (77.0 mg, 0.2 mmol, 1.0 equiv.), methyl ((trimethylsilyl)-methyl)-*L*-methioninate (60.0 mg, 0.24 mmol, 1.2 equiv.), 4CzIPN (2.0 mg, 0.0026 mmol, 0.013 equiv.), lighted in 1 W Blue LED for 2 h, then 60 °C reaction for 3 h. Product **5j** was obtained as a yellow oil (74 mg, 68% yield, 1.5:1 d.r.).

TLC: R_f = 0.4 (Petroleum ether/ethyl acetate 10:1).

^{1}H NMR (400 MHz, $CDCl_3$) δ 7.81 – 7.75 (m, 2H), 7.71 (dd, *J* = 10.7, 8.4 Hz, 1H), 7.61 – 7.57 (m, 1H), 7.33 – 7.29 (m, 3H), 7.05 – 6.99 (m, 1H), 5.73 – 5.59 (m, 1H), 4.91 – 4.85

(m, 0.4H), 4.84 – 4.70 (m, 2.6H), 4.54 – 4.48 (m, 1H), 3.92 (dd, J = 11.0, 7.2 Hz, 0.4H), 3.81 (dd, *J* = 10.8, 7.6 Hz, 0.6H), 3.74 (s, 1.2H), 3.72 – 3.68 (m, 0.6H), 3.66 – 3.62 (m, 0.4H), 3.57 (s, 1.8H), 2.50 – 2.46 (m, 0.6H), 2.42 (s, 3H), 2.38 – 2.18 (m, 2.4H), 2.10 (s, 1.8H), 2.07 – 1.97 (m, 1H), 1.91 (s, 1.2H), 1.04 (s, 1.8H), 0.99 (s, 1.2H), 0.80 (s, 1.2H), 0.73 (s, 1.8H).

^{13}C NMR (101 MHz, $CDCl_3$) δ 173.0, 172.8, 170.6, 170.2, 144.8, 144.7, 143.1, 142.9, 142.0, 141.9, 135.0, 134.5, 129.8, 129.4, 129.4, 128.9, 128.4, 127.4, 127.2, 127.1, 127.0, 123.7, 123.4, 113.7, 113.6, 113.4, 113.3, 62.6, 62.4, 62.3, 62.2, 53.7, 52.8, 52.4, 52.2, 51.2, 42.5, 42.2, 30.5, 30.3, 28.1, 27.7, 22.6, 22.4, 21.9, 21.6, 15.7, 15.4.

HRMS: m/z $[M+H]^+$ calcd for $C_{28}H_{35}N_2O_5S_2$: 543.1982; found: 543.1986.

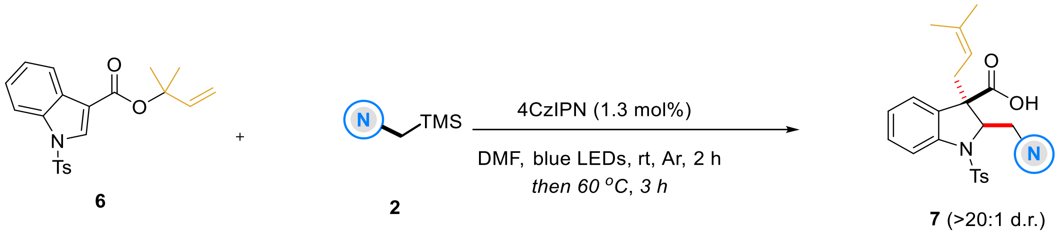

2-((methyl(phenyl)amino)methyl)-3-(3-methylbut-2-en-1-yl)-1-tosylindoline-3-carboxylic acid (7a)

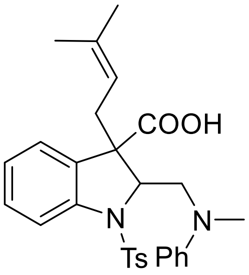

Following the general procedure, 2-methylbut-3-en-2-yl-1-tosyl-1*H*-indole-3-carboxylate (77 mg, 0.2 mmol, 1.0 equiv.), *N*-methyl-*N*-((trimethylsilyl)methyl)aniline (57.9 mg, 0.3 mmol, 1.5 equiv.), 4CzIPN (2.0 mg, 0.0026 mmol, 0.013 equiv.), lighted in 1 W Blue LED for 2 h, then 60 °C reaction for 3 h. Product **7a** was obtained as a colorless oil (80 mg, 79% yield).

TLC: R_f = 0.2 (Petroleum ether /ethyl acetate 2:1).

^{1}H NMR (400 MHz, $CDCl_3$) δ 7.69 (d, *J* = 8.3 Hz, 1H), 7.66 (d, *J* = 8.4 Hz, 2H), 7.34 – 7.28 (m, 3H), 7.20 – 7.14 (m, 3H), 7.07 – 7.03 (m, 1H), 6.93 – 6.83 (m, 3H), 4.87 (t, *J* = 7.1 Hz, 1H), 4.56 (t, *J* = 7.0 Hz, 1H), 3.71 – 3.58 (m, 2H), 2.96 (s, 3H), 2.34 (s, 3H), 2.26 (dd, *J* = 13.7, 5.3 Hz, 1H), 1.53 (s, 3H), 1.43 (dd, *J* = 13.7, 9.2 Hz, 1H), 0.63 (s, 3H).

^{13}C NMR (101 MHz, $CDCl_3$) δ 176.9, 148.8, 144.3, 140.2, 136.8, 135.5, 132.9, 129.7, 129.2, 128.8, 127.4, 127.3, 123.9, 118.7, 117.0, 116.1, 114.8, 67.9, 59.0, 54.1, 41.6, 38.7, 25.8, 21.5, 17.1.

HRMS: m/z $[M+H]^+$ calcd for $C_{29}H_{33}N_2O_4S$: 505.2156; found: 505.2157.

2-((9H-carbazol-9-yl)methyl)-3-(3-methylbut-2-en-1-yl)-1-tosylindoline-3-carboxylic acid (7b)

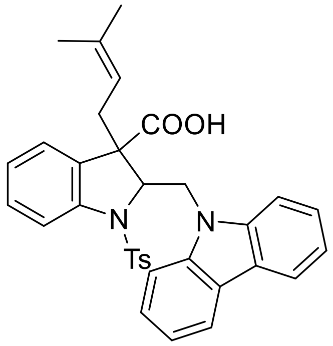

Following the general procedure, 2-methylbut-3-en-2-yl 1-tosyl-1*H*-indole-3-carboxylate (77 mg, 0.2 mmol, 1.0 equiv.), 9-((trimethylsilyl)methyl)-9*H*-carbazole (75.9 mg, 0.3 mmol, 1.5 equiv.), 4CzIPN (2.0 mg, 0.0026 mmol, 0.013 equiv.), lighted in 1 W Blue LED for 2 h, then 60 °C reaction for 3 h. Product **7b** was obtained as a white soild (74.5 mg, 66% yield).

TLC: R_f = 0.2 (Petroleum ether /ethyl acetate 2:1).

^{1}H NMR (400 MHz, $CDCl_3$) δ 8.03 (d, *J* = 7.6 Hz, 2H), 7.58 (d, *J* = 8.0 Hz, 1H), 7.45 – 7.37 (m, 4H), 7.29 – 7.15 (m, 7H), 6.87 (d, *J* = 8.1 Hz, 2H), 5.03 (dd, *J* = 9.8, 4.4 Hz, 1H), 4.98 (t, *J* = 7.7 Hz, 1H), 4.50 (dd, *J* = 14.8, 4.5 Hz, 1H), 4.39 (dd, *J* = 14.8, 9.8 Hz, 1H), 2.64 (dd, *J* = 13.7, 6.3 Hz, 1H), 2.26 (s, 3H), 1.88 (dd, *J* = 13.6, 9.1 Hz, 1H), 1.58 (s, 3H), 0.82 (s, 3H).

^{13}C NMR (101 MHz, Acetone) δ 171.8, 144.1, 141.3, 140.5, 136.4, 136.0, 133.8, 129.5, 128.8, 128.2, 127.0, 125.6, 124.1, 123.3, 120.1, 119.0, 117.3, 117.2, 108.9, 68.6, 59.3, 44.1, 38.3, 25.1, 20.5, 16.6.

HRMS: m/z $[M+H]^+$ calcd for $C_{34}H_{33}N_2O_4S$: 565.2156; found: 565.2159.

m.p. 243 – 244 °C.

2-((allyl(phenyl)amino)methyl)-3-(3-methylbut-2-en-1-yl)-1-tosylindoline-3-carboxylic acid (7c)

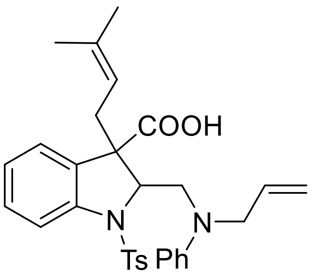

Following the general procedure, 2-methylbut-3-en-2-yl 1-tosyl-1*H*-indole-3-carboxylate (77 mg, 0.2 mmol, 1.0 equiv.), *N*-allyl-*N*-((trimethylsilyl)methyl)aniline (65.7 mg, 0.3 mmol, 1.5 equiv.), 4CzIPN (2.0 mg, 0.0026 mmol, 0.013 equiv.), lighted in 1 W Blue LED for 2 h, then 60 °C reaction for 3 h. Product **7c** was obtained as a lightly yellow oil (70 mg, 66% yield).

TLC: R_f = 0.2 (Petroleum ether /ethyl acetate 2:1).

^{1}H NMR (400 MHz, $CDCl_3$) δ 7.70 (d, *J* = 8.1 Hz, 1H), 7.64 (d, *J* = 8.2 Hz, 2H), 7.33 – 7.29 (m, 3H), 7.18 – 7.14 (m, 3H), 7.06 – 7.02 (m, 1H), 6.96 (d, *J* = 8.1 Hz, 2H), 6.90 – 6.84 (m, 1H), 5.78 – 5.69 (m, 1H), 5.08 – 5.03 (m, 2H), 4.88 – 4.84 (m, 1H), 4.57 (t, *J* = 7.1 Hz, 1H), 3.98 (dd, *J* = 16.5, 5.1 Hz, 1H), 3.85 (dd, *J* = 16.5, 6.0 Hz, 1H), 3.68 (dd, *J* = 14.3, 6.9 Hz, 1H), 3.59 (dd, *J* = 14.3, 7.3 Hz, 1H), 2.33 (s, 3H), 2.21 (dd, *J* = 13.7, 5.3 Hz, 1H), 1.50 (s, 3H), 1.36 (dd, *J* = 13.7, 9.3 Hz, 1H), 0.59 (s, 3H).

^{13}C NMR (101 MHz, $CDCl_3$) δ 177.3, 147.5, 144.3, 140.2, 136.8, 135.4, 133.0, 132.8, 129.7, 129.2, 128.8, 127.3, 123.9, 119.1, 117.6, 117.0, 116.1, 116.0, 67.8, 59.0, 56.6, 51.4, 38.6, 25.8, 21.5, 17.0.

HRMS: m/z $[M+H]^+$ calcd for $C_{31}H_{35}N_2O_4S$: 531.2312; found: 531.2315.

2-((isopropyl(phenyl)amino)methyl)-3-(3-methylbut-2-en-1-yl)-1-tosylindoline-3-carboxylic acid (7d)

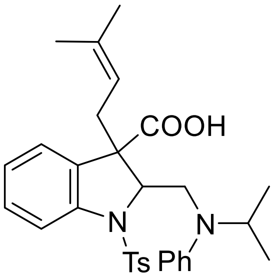

Following the general procedure, 2-methylbut-3-en-2-yl-1-tosyl-1*H*-indole-3-carboxylate (77 mg, 0.2 mmol, 1.0 equiv.), *N*-isopropyl-*N*-((trimethylsilyl)methyl)aniline (66.3 mg, 0.3 mmol, 1.5 equiv.), 4CzIPN (2.0 mg, 0.0026 mmol, 0.013 equiv.), lighted in 1 W Blue LED for 2 h, then 60 °C reaction for 3 h. Product **7d** was obtained as a lightly yellow oil (63 mg, 67% yield).

TLC: R_f = 0.3 (Petroleum ether /ethyl acetate 1:1).

^{1}H NMR (400 MHz, $CDCl_3$) δ 7.68 (d, *J* = 7.6 Hz, 1H), 7.60 (d, *J* = 8.1 Hz, 1H), 7.54 – 7.50 (m, 2H), 7.48 (d, *J* = 7.6 Hz, 2H), 7.43 – 7.39 (m, 1H), 7.33 (d, *J* = 8.3 Hz, 2H), 7.25 – 7.17 (m, 1H), 7.08 – 6.98 (m, 3H), 4.63 – 4.53 (m, 1H), 3.96 – 3.93 (m, 2H), 3.50 – 3.40 (m, 2H), 2.27 (s, 3H), 1.73 (dd, *J* = 14.3, 8.6 Hz, 1H), 1.48 (s, 3H), 1.31 – 1.25 (m, 1H), 1.13 (s, 3H), 1.12 (s, 3H), 0.89 (s, 3H).

^{13}C NMR (101 MHz, $CDCl_3$) δ 173.6, 144.4, 139.7, 139.1, 135.8, 134.5, 134.0, 129.7, 129.3, 128.5, 127.3, 126.9, 125.7, 124.3, 117.7, 115.0, 64.3, 61.3, 57.6, 52.9, 41.6, 25.7, 21.5, 19.2, 17.5, 17.4.

HRMS: m/z $[M+H]^+$ calcd for $C_{31}H_{37}N_2O_4S$: 533.2469; found: 533.2473.

3-(3-methylbut-2-en-1-yl)-2-(morpholinomethyl)-1-tosylindoline-3-carboxylic acid (7e)

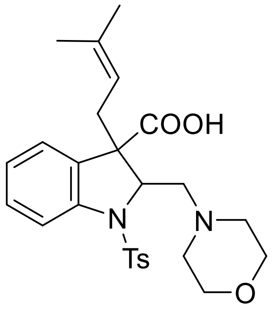

Following the general procedure, 2-methylbut-3-en-2-yl-1-tosyl-1*H*-indole-3-carboxylate (77 mg, 0.2 mmol, 1.0 equiv.), 4-((trimethylsilyl)methyl)morpholine (52.0 mg, 0.3 mmol, 1.5 equiv.), 4CzIPN (2.0 mg, 0.0026 mmol, 0.013 equiv.), lighted in 1 W Blue LED for 2 h, then 60 °C reaction for 3 h. Product **7e** was obtained as a lightly white solid (67 mg, 69% yield).

TLC: R_f = 0.3 (Petroleum ether /ethyl acetate 2:1).

^{1}H NMR (400 MHz, $CDCl_3$) δ 7.69 (d, *J* = 8.2 Hz, 2H), 7.60 (d, *J* = 8.1 Hz, 1H), 7.43 (d, *J* = 7.6 Hz, 1H), 7.26 – 7.20 (m, 3H), 7.04 – 6.98 (m, 1H), 4.97 (t, *J* = 6.8 Hz, 1H), 4.30 (dd, *J* = 8.5, 3.8 Hz, 1H), 3.78 – 3.64 (m, 4H), 3.20 – 3.03 (m, 2H), 2.95 – 2.85 (m, 2H), 2.81 – 2.74 (m, 2H), 2.35 (s, 3H), 1.91 (dd, *J* = 14.1, 5.9 Hz, 1H), 1.79 (dd, *J* = 14.0, 8.0 Hz, 1H), 1.56 (s, 3H), 0.86 (s, 3H).

^{13}C NMR (101 MHz, $CDCl_3$) δ 174.4, 144.7, 139.5, 136.3, 134.8, 133.8, 129.9, 128.6, 127.2, 127.1, 124.2, 118.0, 114.8, 65.5, 65.3, 60.6, 60.0, 52.9, 40.7, 25.9, 21.6, 17.4.

HRMS: m/z $[M+H]^+$ calcd for $C_{26}H_{33}N_2O_5S$: 485.2105; found: 485.2107.

m.p. 75 – 76 °C.

2-((4-(ethoxycarbonyl)piperidin-1-yl)methyl)-3-(3-methylbut-2-en-1-yl)-1-tosylindoline-3-carboxylic acid (7f)

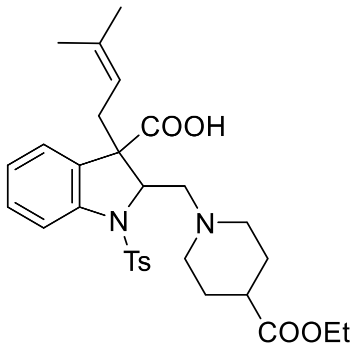

Following the general procedure, 2-methylbut-3-en-2-yl-1-tosyl-1*H*-indole-3-carboxylate (77 mg, 0.2 mmol, 1.0 equiv.), ethyl 1-((trimethylsilyl)methyl)piperidine-4-carboxylate (73.0 mg, 0.3 mmol, 1.5 equiv.), 4CzIPN (2.0 mg, 0.0026 mmol, 0.013 equiv.), lighted in 1 W Blue LED for 2 h, then 60 °C reaction for 3 h. Product **7f** was obtained as a lightly yellow oil (92 mg, 83% yield).

TLC: R_f = 0.3 (Dichloromethane /methanol 20:1).

^{1}H NMR (400 MHz, $CDCl_3$) δ 7.65 (d, *J* = 8.2 Hz, 3H), 7.55 (d, *J* = 8.0 Hz, 1H), 7.22 – 7.15 (m, 3H), 7.02 – 6.96 (m, 1H), 5.00 (t, *J* = 6.8 Hz, 1H), 4.28 (dd, *J* = 7.9, 4.6 Hz, 1H), 4.13 (q, *J* = 7.1 Hz, 2H), 3.47 – 3.21 (br, 1H), 3.20 – 3.06 (m, 3H), 3.05 – 2.59 (m, 2H), 2.58 – – 2.40 (br, 1H), 2.32 (s, 3H), 2.14 – 2.06 (m, 1H), 2.04 – 1.90 (m, 3H), 1.84 (dd, *J* = 14.1, 7.6 Hz, 1H), 1.74 (dd, *J* = 14.1, 6.1 Hz, 1H), 1.57 (s, 3H), 1.23 (t, *J* = 7.1 Hz, 3H), 0.88 (s, 3H).

^{13}C NMR (101 MHz, $CDCl_3$) δ 173.7, 173.1, 144.8, 138.9, 136.0, 134.7, 134.2, 130.0, 128.4, 127.7, 127.1, 124.4, 118.4, 114.5, 64.2, 61.5, 61.0, 59.4, 51.9, 51.0, 41.6, 38.6, 25.9, 21.6, 17.6, 14.2.

HRMS: m/z $[M+H]^+$ calcd for $C_{30}H_{39}N_2O_6S$: 555.2523; found: 555.2525.

2-((4-(tert-butoxycarbonyl)piperazin-1-yl)methyl)-3-(3-methylbut-2-en-1-yl)-1-tosylindoline-3-carboxylic acid (7g)

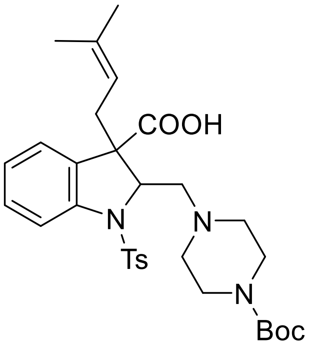

Following the general procedure, 2-methylbut-3-en-2-yl 1-tosyl-1*H*-indole-3-carboxylate (77 mg, 0.2 mmol, 1.0 equiv.), tert-butyl 4-((trimethylsilyl)methyl)piperazine-1-carboxylate (82.0 mg, 0.3 mmol, 1.5 equiv.), 4CzIPN (2.0 mg, 0.0026 mmol, 0.013 equiv.), lighted in 1 W Blue LED for 2 h, then 60 °C reaction for 3 h. Product **7g** was obtained as a white solid (91 mg, 78% yield).

TLC: R_f = 0.5 (Dichloromethane / methanol 20:1).

^{1}H NMR (400 MHz, $CDCl_3$) δ 7.69 (d, *J* = 8.3 Hz, 2H), 7.59 (d, *J* = 8.0 Hz, 1H), 7.37 (d, *J* = 7.5 Hz, 1H), 7.24 – 7.20 (m, 3H), 7.02 – 6.96 (m, 1H), 4.94 (t, *J* = 6.8 Hz, 1H), 4.29 (dd, *J* = 8.1, 4.2 Hz, 1H), 3.55 – 3.33 (br, 4H), 3.14 – 3.01 (m, 2H), 2.82 – 2.60 (m, 4H), 2.34 (s, 3H), 1.97 (dd, *J* = 14.0, 5.5 Hz, 1H), 1.73 (dd, *J* = 13.9, 8.2 Hz, 1H), 1.55 (s, 3H), 1.45 (s, 9H), 0.81 (s, 3H).

^{13}C NMR (101 MHz, $CDCl_3$) δ 174.7, 154.3, 144.6, 139.7, 136.3, 135.0, 133.7, 129.9, 128.5, 127.1, 124.1, 118.0, 114.8, 80.3, 66.1, 60.1, 59.6, 52.6, 42.7, 42.1, 40.4, 28.4, 25.9, 21.5, 17.4.

HRMS: m/z $[M+H]^+$ calcd for $C_{31}H_{42}N_3O_6S$: 584.2789; found: 584.2791.

m.p. 93 – 94 °C.

2-((4-(bis(4-fluorophenyl)methyl)piperazin-1-yl)methyl)-3-(3-methylbut-2-en-1-yl)-1-tosylindoline-3-carboxylic acid (7h)

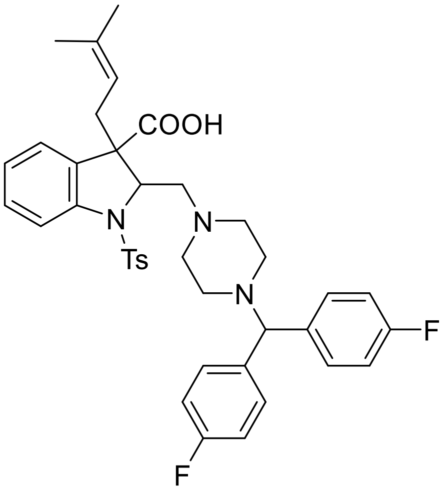

Following the general procedure, 2-methylbut-3-en-2-yl-1-tosyl-1*H*-indole-3-carboxylate (77 mg, 0.2 mmol, 1.0 equiv.), 1-(bis(4-fluorophenyl)methyl)-4-((trimethylsilyl)methy-l)piperazine (112.3 mg, 0.3 mmol, 1.5 equiv.), 4CzIPN (2.0 mg, 0.0026 mmol, 0.013 equiv.), lighted in 1 W Blue LED for 2 h, then 60 °C reaction for 3 h. Product **7h** was obtained as a white solid (111 mg, 81% yield).

TLC: R_f = 0.2 (Dichloromethane / methanol 2:1).

^{1}H NMR (400 MHz, $CDCl_3$) δ 7.65 (d, *J* = 8.3 Hz, 2H), 7.61 – 7.51 (m, 2H), 7.37 – 7.29 (m, 4H), 7.23 – 7.17 (m, 3H), 7.01 – 6.95 (m, 5H), 4.99 (t, *J* = 6.2 Hz, 1H), 4.32 – 4.20 (m, 2H), 3.36 – 2.80 (m, 6H), 2.79 – 2.35 (br, 4H), 2.34 (s, 3H), 1.96 – 1.62(m, 2H), 1.55 (s, 3H), 0.88 (s, 3H).

^{13}C NMR (101 MHz, $CDCl_3$) δ 173.7, 162.1 (d, $^1J_{C\text{-}F}$ = 245.3 Hz), 144.8, 139.0, 136.9 (d, $^4J_{C\text{-}F}$ = 3.5 Hz), 136.0, 134.2, 130.0, 129.3, 129.2, 129.1, 128.5, 127.5, 127.0, 124.4, 118.3, 115.8 (d, $^2J_{C\text{-}F}$ = 21.1 Hz), 114.6, 73.9, 64.3, 61.4, 59.4, 52.3, 49.7, 41.5, 25.9, 21.5, 17.5.

^{19}F NMR (376 MHz, CDCl3) δ –114.6.

HRMS: m/z $[M+H]^+$ calcd for $C_{39}H_{42}F_2N_3O_4S$: 686.2859; found: 686.2851.

m.p. 146 – 147 °C.

8b-(3-methylbut-2-en-1-yl)-2-phenyl-4-tosyl-3,3a,4,8b-tetrahydropyrrolo*[3,4-b]*indol-1(2*H*)-one (7i)

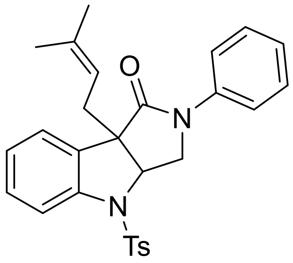

Following the general procedure, 2-methylbut-3-en-2-yl-1-tosyl-1*H*-indole-3-carboxylate (77 mg, 0.2 mmol, 1.0 equiv.), *N*-((trimethylsilyl)methyl)aniline (112.3 mg, 0.3 mmol, 1.5 equiv.), 4CzIPN (2.0 mg, 0.0026 mmol, 0.013 equiv.), lighted in 1 W Blue LED for 2 h, then 60 °C reaction for 3 h. Product **7i** was obtained as a colorless oil (67 mg, 71% yield).

TLC: R_f = 0.3 (Petroleum ether /ethyl acetate 20:1).

^{1}H NMR (400 MHz, $CDCl_3$) δ 7.72 (d, *J* = 8.3 Hz, 2H), 7.68 (d, *J* = 8.1 Hz, 1H), 7.65 – 7.61 (m, 2H), 7.44 (dd, *J* = 7.6, 0.8 Hz, 1H), 7.38 – 7.32 (m, 2H), 7.32 – 7.28 (m, 1H), 7.26 (d, *J* = 7.6 Hz, 2H), 7.19 – 7.13 (m, 1H), 7.12 – 7.08 (m, 1H), 4.65 – 4.61 (m, 1H), 4.54 (dd, *J* = 6.4, 3.2 Hz, 1H), 4.33 – 4.25 (m, 2H), 2.68 (dd, *J* = 14.8, 6.5 Hz, 1H), 2.38 (s, 3H), 2.22 (dd, *J* = 14.8, 7.9 Hz, 1H), 1.51 (s, 3H), 1.45 (s, 3H).

^{13}C NMR (101 MHz, $CDCl_3$) δ 172.3, 144.7, 141.1, 138.6, 136.6, 134.1, 131.4, 129.9, 129.4, 129.0, 127.3, 125.1, 124.7, 124.6, 120.1, 117.3, 114.5, 62.2, 58.9, 55.0, 34.6, 25.9, 21.6, 18.2.

HRMS: m/z $[M+H]^+$ calcd for $C_{28}H_{29}N_2O_3S$: 473.1893; found: 473.1893.

methyl 2-(8b-(3-methylbut-2-en-1-yl)-1-oxo-4-tosyl-3,3a,4,8b-tetrahydropyrrolo*[3,* *4-b]*indol-2(1*H*)-yl)acetate (7j)

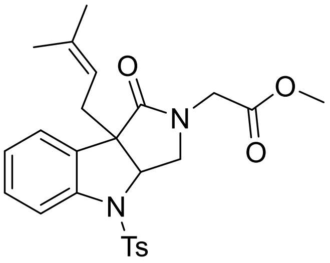

Following the general procedure, 2-methylbut-3-en-2-yl-1-tosyl-1*H*-indole-3-carboxylate (77 mg, 0.2 mmol, 1.0 equiv.), methyl ((trimethylsilyl)methyl)glycinate (52.5 mg, 0.3 mmol, 1.5 equiv.), 4CzIPN (2.0 mg, 0.0026 mmol, 0.013 equiv.), lighted in 1 W Blue LED for 2 h, then 60 °C reaction for 3 h. Product **7j** was obtained as a white soild (35 mg, 37% yield).

TLC: R_f = 0.2 (Petroleum ether /ethyl acetate 10:1).

^{1}H NMR (500 MHz, $CDCl_3$) δ 7.67 (d, *J* = 8.3 Hz, 2H), 7.64 (d, *J* = 8.2 Hz, 1H), 7.35 (d, *J* = 7.5 Hz, 1H), 7.28 – 7.24 (m, 1H), 7.23 (d, *J* = 8.0 Hz, 2H), 7.06 – 7.02 (m, 1H), 4.64 (t, *J* = 7.0 Hz, 1H), 4.44 (dd, *J* = 7.1, 1.9 Hz, 1H), 4.35 (d, *J* = 17.6 Hz, 1H), 4.01 (dd, *J* = 10.9, 7.2 Hz, 1H), 3.78 (dd, *J* = 10.9, 1.9 Hz, 1H), 3.72 (s, 3H), 3.69 (d, *J* = 17.6 Hz, 1H), 2.58 (dd, *J* = 14.9, 6.1 Hz, 1H), 2.36 (s, 3H), 2.18 (dd, *J* = 14.9, 8.1 Hz, 1H), 1.52 (s, 3H), 1.41 (s, 3H).

^{13}C NMR (101 MHz, $CDCl_3$) δ 173.8, 168.5, 144.7, 141.3, 136.2, 131.2, 129.9, 129.3, 127.4, 124.7, 124.6, 117.6, 114.5, 63.3, 57.4, 54.5, 52.3, 44.1, 34.3, 25.8, 21.6, 18.1.

HRMS: m/z $[M+H]^+$ calcd for $C_{25}H_{29}N_2O_5S$: 469.1792; found: 469.1795.

m.p. 169 – 170 °C

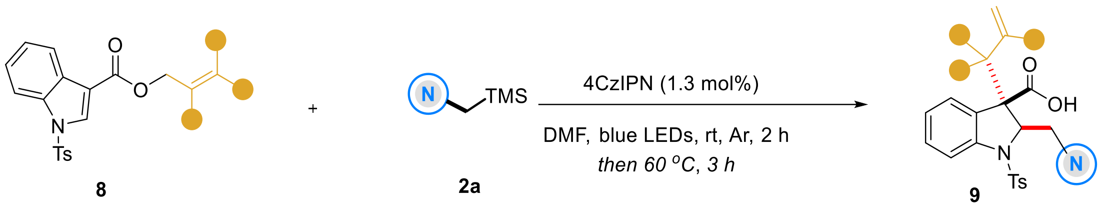

3-(3,7-dimethylocta-1,6-dien-3-yl)-2-((methyl(phenyl)amino)methyl)-1-tosyl-indoline-3-carboxylic acid (9a)

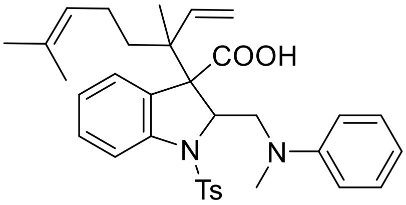

Following the general procedure, *(E)*-3,7-dimethylocta-2,6-dien-1-yl-1-tosyl-1*H*-indole-3-carboxylate (90.2 mg, 0.2 mmol, 1.0 equiv.), *N*-methyl-*N*-((trimethylsilyl)methyl)aniline (57.9 mg, 0.3 mmol, 1.5 equiv.), 4CzIPN (2.0 mg, 0.0026 mmol, 0.013 equiv.), lighted in 1 W Blue LED for 2 h, then 60 °C reaction for 3 h. Product **9a** was obtained as a colorless oil (67 mg, 59% yield, 2:1 d.r.).

TLC: R_f = 0.3 (Petroleum ether /ethyl acetate 2:1).

^{1}H NMR (400 MHz, $CDCl_3$) δ 7.74 – 7.68 (m, 2H), 7.64 – 7.58 (m, 0.66H), 7.52 – 7.48 (m, 1.34H), 7.33 – 7.29 (m, 1H), 7.24 – 7.18 (m, 2H), 7.16 – 7.11 (m, 2H), 7.09 – 7.03 (m, 1H), 6.95 – 6.80 (m, 3H), 6.20 (dd, *J* = 17.5, 11.0 Hz, 0.34H), 5.60 (dd, *J* = 17.4, 10.9 Hz, 0.66H), 5.10 – 4.90 (m, 3H), 4.82 – 4.76 (m, 1H),3.67 (dd, *J* = 14.0, 6.9 Hz, 1H), 3.53 – 3.47 (m, 1H), 2.73 (s, 1H), 2.68 (s, 2H), 2.36 (s, 3H), 1.87 – 1.81 (m, 0.66H), 1.75 – 1.59 (m, 5.34H), 1.52 – 1.50 (m, 3H), 1.48 – 1.40 (m, 0.33H), 1.25 – 1.19 (m, 0.67H), 0.76 (s, 2H), 0.37 (s, 1H).

^{13}C NMR (101 MHz, $CDCl_3$) δ 175.7, 175.5, 148.8, 148.1, 144.0, 143.7, 142.5, 141.8, 141.6, 140.7, 137.6, 137.0, 131.4, 131.2, 131.1, 129.8, 129.5, 129.4, 129.1, 129.0, 128.8, 127.0, 124.7, 124.5, 123.0, 122.6, 121.3, 120.2, 116.9, 116.4, 116.2, 114.4, 114.2, 66.9, 66.6, 64.3, 63.8, 56.4, 55.9, 47.7, 47.1, 42.1, 41.8, 35.4, 34.4, 25.8, 25.7, 23.2, 23.1, 21.5, 17.7, 17.6, 17.5, 16.6.

HRMS: m/z $[M+H]^+$ calcd for $C_{34}H_{41}N_2O_4S$: 573.2782; found: 573.2783.

2-((methyl(phenyl)amino)methyl)-1-tosyl-3-(3,6,11-trimethyldodeca-1,6,10-trien-3-yl)indoline-3-carboxylic acid (9b)

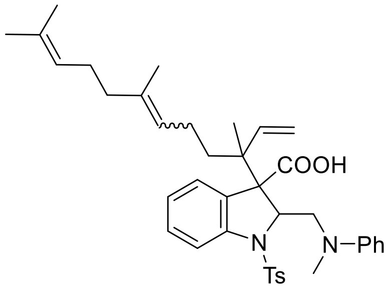

Following the general procedure, *(2E,6E)*-3,7,11-trimethyldodeca-2,6,10-trien-1-yl 1-tosyl-1*H*-indole-3-carboxyla-te (103.8 mg, 0.2 mmol, 1.0 equiv.), *N*-methyl-*N*-((trimethylsilyl)methyl)aniline (57.9 mg, 0.3 mmol, 1.5 equiv.), 4CzIPN (2.0 mg, 0.0026 mmol, 0.013 equiv.), lighted in 1 W Blue LED for 2 h, then 60 °C reaction for 3 h. Product **9b** was obtained as a colorless oil (169 mg, 66% yield, 1.3:1 d.r.).

TLC: R_f = 0.3 (Petroleum ether /ethyl acetate 2:1).

^{1}H NMR (400 MHz, $CDCl_3$) δ 7.73 – 7.67 (m, 2H), 7.65 – 7.59 (m, 1H), 7.51 – 7.46 (m, 1H), 7.35 – 7.30 (m, 1H), 7.24 – 7.18 (m, 2H), 7.17 – 7.11 (m, 2H), 7.10 – 7.02 (m, 1H), 6.95 – 6.77 (m, 3H), 6.21 – 6.15 (m, 0.43H), 5.66 – 5.55 (m, 0.57H), 5.14 – 4.88 (m, 4H), 4.77 (d, *J* = 16.8 Hz, 1H), 3.71 – 3.62 (m, 1H), 3.48 (dd, *J* = 13.5, 5.8 Hz, 1H), 2.72 (s, 1.3H), 2.67 (s, 1.7H), 2.13 – 1.83 (m, 5H), 1.75 – 1.59 (m, 10H), 1.53 – 1.49 (m, 2H), 1.52 (s, 1.7H), 0.55 (s, 1.3H).

^{13}C NMR (101 MHz, $CDCl_3$) δ 175.6, 175.3, 148.9, 148.2, 144.0, 143.7, 142.5, 141.7, 141.6, 140.7, 137.7, 137.0, 135.1, 135.0, 134.9, 134.8, 131.5, 131.3, 131.2, 129.5, 129.4, 129.0, 128.8, 127.0, 125.3, 125.2, 124.6, 124.5, 124.4, 124.3, 123.0, 122.5, 121.2, 120.2, 116.9, 116.4, 114.4, 114.2, 66.9, 66.8, 66.6, 64.2, 63.8, 56.3, 55.8, 47.7, 47.2, 47.1, 42.2, 41.9, 39.8, 39.7, 35.6, 35.3, 34.5, 34.3, 32.0, 31.9, 26.8, 26.7, 26.6, 25.8, 23.5, 23.2, 23.1, 23.0, 22.9, 21.5, 17.8, 17.7, 17.5, 16.6, 16.0.

HRMS: m/z $[M+H]^+$ calcd for $C_{39}H_{49}N_2O_4S$: 641.3408; found: 641.3412.

2-((methyl(phenyl)amino)methyl)-3-(pent-1-en-3-yl)-1-tosylindoline-3-carboxylic acid (9c)

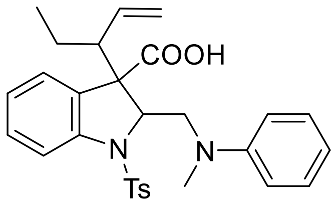

Following the general procedure, (*Z*)-pent-2-en-1-yl 1-tosyl-1*H*-indole-3-carboxylate (116.0 mg, 0.3 mmol, 1.0 equiv.), *N*-((trimethylsilyl)methyl)aniline (69.5 mg, 0.36 mmol, 1.2 equiv.), 4CzIPN (3.0 mg, 0.0039 mmol, 0.013 equiv.), lighted in 1W Blue LED for 2h, then 60 °C reaction for 3h. Product **9c** was obtained as a yellow oil (131 mg, 89% yield, 1.7:1 d.r.).

TLC: R_f = 0.3 (Petroleum ether /ethyl acetate 2:1).

^{1}H NMR (400 MHz, $CDCl_3$) δ 7.68 (d, *J* = 7.5 Hz, 0.64H), 7.64 – 7.55 (m, 3H), 7.44 (d, *J* = 7.4 Hz, 0.36H), 7.33 –7.25 (m, 3H), 7.17 – 6.65 (m, 6H), 5.67 – 5.57 (m, 0.64H), 5.27 – 5.14 (m, 0.36H), 4.94 (dd, *J* = 10.3, 1.7 Hz, 0.64H), 4.71 – 4.51 (m, 2H), 4.04 (dd, *J* = 17.0, 1.7 Hz, 0.36H), 3.71 – 3.49 (m, 2H), 2.88 (s, 1.1H), 2.84 (s, 1.9H), 2.34 (s, 1.9H), 2.33 (s, 1.1H), 1.34 – 1.21 (m, 2.36H), 1.17 – 1.01 (m, 0.64H), 0.49 (t, *J* = 6.9 Hz, 1.1H), 0.37 (t, *J* = 7.2 Hz, 1.9H).

^{13}C NMR (101 MHz, $CDCl_3$) δ 174.4, 174.0, 147.7, 147.6, 144.3, 144.1, 140.3, 140.0, 137.6, 136.2, 136.0, 135.8, 132.5, 132.3, 129.6, 129.5, 129.4, 129.3, 129.2, 128.9, 128.7, 127.2, 127.1, 123.8, 123.3, 121.8, 121.2, 119.4, 119.2, 117.5, 117.0, 115.7, 115.4, 65.6, 65.1, 63.1, 63.0, 55.4, 55.2, 55.0, 54.4, 42.7, 42.5, 22.6, 21.5, 21.4, 20.7, 12.2.

HRMS: m/z $[M+H]^+$ calcd for $C_{29}H_{33}N_2O_4S$: 505.2156; found: 505.2157.

3-allyl-2-((methyl(phenyl)amino)methyl)-1-tosylindoline-3-carboxylic acid (9d)

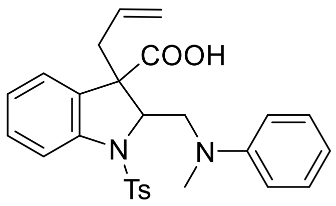

Following the general procedure, allyl 1-tosyl-1*H*-indole-3-carboxylate (106.5 mg, 0.3 mmol, 1.0 equiv.), *N*-((trimethylsilyl)methyl)aniline (69.5 mg, 0.36 mmol, 1.2 equiv.), 4CzIPN (3.0 mg, 0.0039 mmol, 0.013 equiv.), lighted in 1 W Blue LED for 2 h, then 60 °C reaction for 3 h. Product **9d** was obtained as a lightly yellow solid (102 mg, 67% yield).

TLC: R_f = 0.3 (Petroleum ether /ethyl acetate 2:1).

^{1}H NMR (400 MHz, $CDCl_3$) δ 7.70 (d, *J* = 8.1 Hz, 1H), 7.57 (d, J = 8.3 Hz, 2H), 7.33 – 7.29 (m, 2H), 7.27 – 7.23 (m, 2H), 7.17 (d, *J* = 8.2 Hz, 2H), 7.10 – 7.06 (m, 1H), 6.96 – 6.82 (m, 3H), 5.39 – 5.29 (m, 1H), 4.74 (dd, *J* = 10.2, 1.5 Hz, 1H), 4.40 (t, *J* = 7.1 Hz, 1H), 4.32 (d, *J* = 17.0 Hz, 1H), 3.70 – 3.61 (m, 2H), 2.91 (s, 3H), 2.34 (s, 3H), 2.16 (dd, *J =* 13.5, 6.8 Hz, 1H), 1.37 (dd, *J =* 13.5, 7.7 Hz, 1H).

^{13}C NMR (101 MHz, $CDCl_3$) δ 175.9, 148.3, 144.4, 140.4, 135.1, 133.2, 131.9, 129.7, 129.2, 129.0, 127.3, 127.1, 124.3, 119.6, 119.5, 116.6, 115.5, 66.9, 59.0, 54.2, 44.2, 42.1, 21.5.

HRMS: m/z $[M+H]^+$ calcd for $C_{27}H_{29}N_2O_4S$: 477.1843; found: 477.1847.

m.p. 82 – 83 °C.

2-((methyl(phenyl)amino)methyl)-3-(2-methylallyl)-1-tosylindoline-3-carboxylic acid (9e)

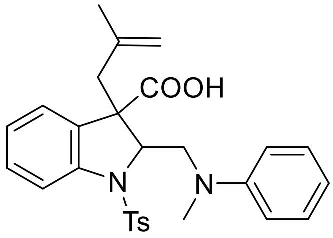

Following the general procedure, 2-methylallyl 1-tosyl-1*H*-indole-3-carboxylate (73.8 mg, 0.2 mmol, 1.0 equiv.), *N*-methyl-*N*-((trimethylsilyl)methyl)aniline (46.3 mg, 0.24 mmol, 1.2 equiv.), 4CzIPN (2.0 mg, 0.0026 mmol, 0.013 equiv.), lighted in 1 W Blue LED for 2 h, then 60 °C reaction for 3 h. Product **9e** was obtained as a lightly yellow oil (75 mg, 76% yield).

TLC: R_f = 0.2 (Petroleum ether /ethyl acetate 2:1).

^{1}H NMR (400 MHz, $CDCl_3$) δ 7.69 (d, *J* = 8.1 Hz, 1H), 7.60 (d, *J* = 8.3 Hz, 2H), 7.33 – 7.27 (m, 2H), 7.27 – 7.23 (m, 2H), 7.15 (d, *J* = 8.1 Hz, 2H), 7.08 – 7.02 (m, 1H), 6.86 – 6.82 (m, 3H), 4.51 (s, 1H), 4.45 (t, *J* = 6.9 Hz, 1H), 3.88 (s, 1H), 3.67 – 3.55 (m, 2H), 2.93 (s, 3H), 2.52 (d, *J* = 13.3 Hz, 1H), 2.32 (s, 3H), 1.31 (d, *J* = 13.3 Hz, 1H), 1.11 (s, 3H).

^{13}C NMR (101 MHz, $CDCl_3$) δ 176.6, 148.3, 144.3, 140.2, 139.8, 135.5, 132.3, 129.6, 129.2, 129.0, 127.8, 127.3, 124.0, 118.9, 116.5, 116.1, 114.5, 67.6, 59.0, 53.9, 46.5, 41.5, 23.6, 21.5.

HRMS: m/z $[M+H]^+$ calcd for $C_{28}H_{31}N_2O_4S$: 491.1999; found: 491.2001.

3-(2-chloroallyl)-2-((methyl(phenyl)amino)methyl)-1-tosylindoline-3-carboxylic acid (9f)

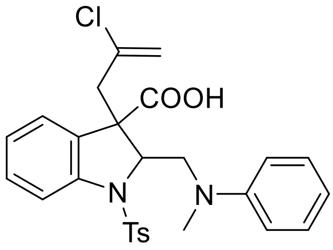

Following the general procedure, 2-chloroallyl-1-tosyl-1*H*-indole-3-carboxylate (77.8 mg, 0.2 mmol, 1.0 equiv.), *N*-methyl-*N*-((trimethylsilyl)methyl)aniline (46.3 mg, 0.24 mmol, 1.2 equiv.), 4CzIPN (2.0 mg, 0.0026 mmol, 0.013 equiv.), lighted in 1 W Blue LED for 2 h, then 60 °C reaction for 3 h. Product **9f** was obtained as a lightly yellow oil (61 mg, 60% yield).

TLC: R_f = 0.2 (Petroleum ether /ethyl acetate 1:1).

^{1}H NMR (400 MHz, $CDCl_3$) δδ 7.65 (d, *J* = 8.1 Hz, 1H), 7.60 (d, *J* = 8.3 Hz, 2H), 7.43 (d, *J* = 7.6 Hz, 1H), 7.34 – 7.30 (m, 1H), 7.25 (d, *J* = 7.9 Hz, 2H), 7.16 (d, *J* = 8.2 Hz, 2H), 7.10 – 7.06 (m, 1H), 6.86 – 6.80 (m, 3H), 4.87 (s, 1H), 4.50 (t, *J* = 6.8 Hz, 1H), 4.14 (s, 1H), 3.67 – 3.53 (m, 2H), 2.94 (s, 3H), 2.81 (d, *J* = 14.1 Hz, 1H), 2.34 (s, 3H), 1.72 (d, *J* = 14.1 Hz, 1H).

^{13}C NMR (101 MHz, $CDCl_3$) δ 175.4, 148.1, 144.5, 140.1, 136.5, 135.5, 131.4, 129.8, 129.3, 128.1, 127.3, 124.3, 118.9, 117.5, 116.6, 114.4, 67.1, 58.7, 53.8, 47.7, 41.3, 21.5.

HRMS: m/z $[M+H]^+$ calcd for $C_{27}H_{28}{}^{35}ClN_2O_4S$: 511.1453; found: 511.1452.

HRMS: m/z $[M+H]^+$ calcd for $C_{27}H_{28}{}^{37}ClN_2O_4S$: 513.1423; found: 513.1422.

3-(buta-2,3-dien-2-yl)-2-((methyl(phenyl)amino)methyl)-1-tosylindoline-3-carboxylic acid (9g)

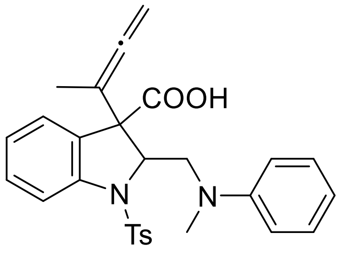

Following the general procedure, but-2-yn-1-yl-1-tosyl-1*H*-indole-3-carboxylate (110.1 mg, 0.3 mmol, 1.0 equiv.), *N*-((trimethylsilyl)methyl)aniline (69.5 mg, 0.36 mmol, 1.2 equiv.), 4CzIPN (3.0 mg, 0.0039 mmol, 0.013 equiv.), lighted in 1 W Blue LED for 2 h, then 60 °C reaction for 3 h. Product **9g** was obtained as a yellow solid (85 mg, 58% yield).

TLC: R_f = 0.2 (Petroleum ether /ethyl acetate 2:1).

^{1}H NMR (400 MHz, $CDCl_3$) δ 7.62 (d, *J* = 8.1 Hz, 1H), 7.54 (d, *J* = 8.1 Hz, 2H), 7.48 (d, *J* = 7.7 Hz, 1H), 7.35 – 7.29 (m, 3H), 7.12 (d, *J* = 8.2 Hz, 2H), 7.10 – 7.06 (m, 1H), 7.03 (d, *J* = 8.2 Hz, 2H), 6.99 – 6.93 (m, 1H), 4.83 (t, *J* = 6.9 Hz, 1H), 4.28 – 4.22 (m, 1H), 3.95 (dd, *J* = 10.3, 2.9 Hz, 1H), 3.75 (dd, *J* = 14.0, 7.4 Hz, 1H), 3.65 (dd, *J* = 14.0, 6.4 Hz, 1H), 2.97 (s, 3H), 2.34 (s, 3H), 1.40 (s, 3H).

^{13}C NMR (101 MHz, $CDCl_3$) δ 207.3, 174.4, 147.6, 143.9, 140.2, 135.8, 132.3, 129.4, 129.3, 129.1, 128.7, 127.2, 123.9, 120.7, 116.3, 116.0, 101.5, 65.9, 61.9, 55.0, 42.5, 21.5, 15.9.

HRMS: m/z $[M+H]^+$ calcd for $C_{28}H_{29}N_2O_4S$: 489.1843; found: 489.1846.

m.p. 140 – 141 °C.

4. Supplementary method C: synthetic transformations

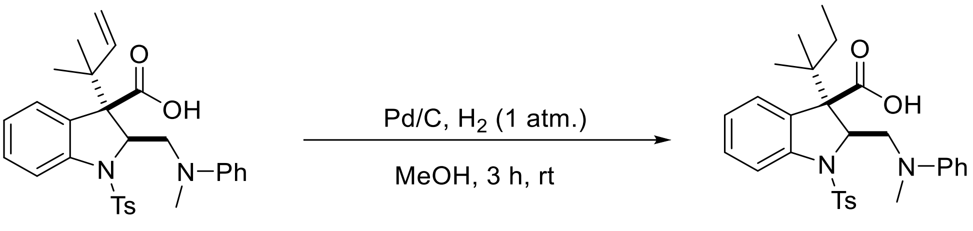

3d **10**

To a solution of **3d** (100.8 mg, 0.2 mmol, 1.0 equiv.) in methanol (10 mL) was hydrogenated at 1 atmaosphere of hydrogen using palladium-on-carbon (5% wetted with ca. 55% water, 45 mg) as catalyst for 3 h. The mixture was filtered and concentrated in vacuo, the residue was purified by column chromatography on silica gel (Petroleum ether / ethyl acetate = 10: 1) to afford the product of **10** (91 mg, 0.18 mmol，90%).

2-((methyl(phenyl)amino)methyl)-3-(tert-pentyl)-1-tosylindoline-3-carboxylic acid (10)

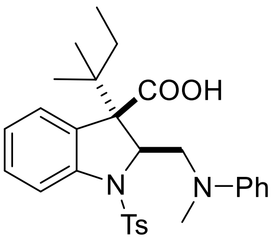

Product **10** was obtained as a white solid (91 mg, 90% yield).

TLC: R_f = 0.2 (Petroleum ether /ethyl acetate 20:1).

^{1}H NMR (400 MHz, $CDCl_3$) δ 7.67 (d, *J* = 8.1 Hz, 2H), 7.58 – 7.51 (m, 2H), 7.32 – 7.28 (m, 1H), 7.22 – 7.16 (m, 2H), 7.11 (d, *J* = 8.2 Hz, 2H), 7.08 – 7.04 (m, 1H), 6.84 – 6.80 (m, 1H), 6.74 (d, *J* = 7.4 Hz, 2H), 5.04 – 4.96 (m, 1H), 3.59 – 3.47 (m, 2H), 2.65 (s, 3H), 2.32 (s, 3H), 1.26 – 1.14 (br, 1H), 0.94 – 0.88 (br, 1H), 0.84 (s, 3H), 0.77 (s, 3H), 0.56 (t, *J* = 7.0 Hz, 3H).

^{13}C NMR (101 MHz, $CDCl_3$) δ 176.1, 149.1, 143.7, 141.5, 137.3, 131.5, 129.5, 129.4, 129.0, 128.7, 127.0, 122.9, 119.8, 115.9, 114.5, 67.1, 63.8, 56.0, 41.5, 41.1, 28.9, 21.7, 21.5, 8.5.

HRMS: m/z $[M+H]^+$ calcd for $C_{29}H_{35}N_2O_4S$: 507.2312; found: 507.2315.

m.p. 94 – 95 °C.

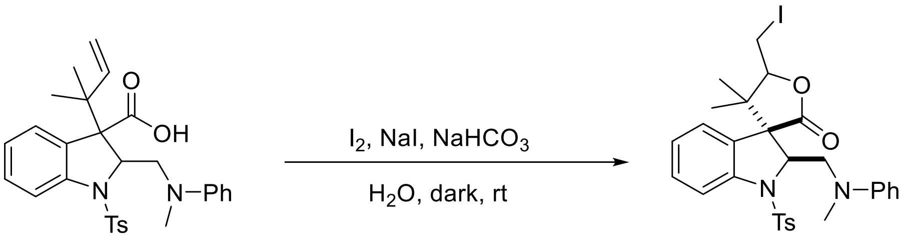

3d **11**

To a suspension of **3d** (100.8 mg, 0.2 mmol, 1.0 equiv.) and $NaHCO_3$ (50.4 mg, 0.6 mmol, 3.0 equiv.) in H_2O (1.0 mL) was added NaI (90.0 mg, 1.2 mmol, 6.0 equiv.), followed by I_2 (50.8 mg, 0.2 mmol, 1.0 equiv.) under argon atmosphere. The flask was covered with aluminum foil. Then the reaction mixture was stirred in dark for 4 h before being uncovered to reveal a brown precipitate. The reaction was extracted with CH_2Cl_2, washed with 10% $Na_2S_2O_3$, 10% $NaHCO_3$, and brine. The organic phase was dried over anhydrous Na_2SO_4, filtered and concentrated in vacuo. The residue was purified by column chromatography on silica gel (Petroleum ether / ethyl acetate = 50: 1) to afford the product of **11** (80 mg, 0.13 mmol, 63%)**.**

5-(iodomethyl)-4,4-dimethyl-2'-((methyl(phenyl)amino)methyl)-1'-tosyl-4,5-dihydro-2H-spiro[furan-3,3'-indolin]-2-one (11)

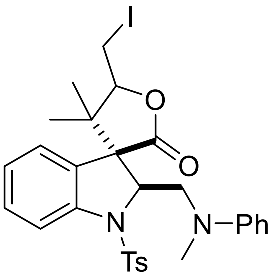

Product **11** was obtained as a white solid (80 mg, 63% yield).

TLC: R_f = 0.6 (Petroleum ether /ethyl acetate 10:1).

^{1}H NMR (500 MHz, $CDCl_3$) δ 7.85 (d, *J* = 8.3 Hz, 2H), 7.80 (d, *J* = 8.2 Hz, 1H), 7.54 – 7.48 (m, 1H), 7.42 – 7.38 (m, 2H), 7.32 (d, *J* = 8.2 Hz, 2H), 7.27 – 7.22 (m, 2H), 7.11 (d, *J* = 8.2 Hz, 2H), 6.98 – 6.92 (m, 1H), 4.97 (dd, *J* = 9.2, 5.8 Hz, 1H), 4.61 (dd, *J* = 9.6, 3.7 Hz, 1H), 4.04 (dd, *J* = 14.5, 5.7 Hz, 1H), 3.94 (dd, *J* = 14.5, 9.3 Hz, 1H), 3.28 (dd, *J* = 10.8, 3.7 Hz, 1H), 3.24 – 3.14 (m, 1H), 2.95 (s, 3H), 2.48 (s, 3H), 0.88 (s, 3H), 0.44 (s, 3H).

^{13}C NMR (101 MHz, $CDCl_3$) δ 174.0, 150.4, 144.5, 141.6, 135.7, 130.1, 129.8, 129.2, 127.3, 126.2, 124.6, 118.6, 116.6, 115.4, 86.3, 63.0, 62.5, 53.6, 46.6, 43.0, 21.9, 21.6, 18.4, 0.1.

HRMS: m/z $[M+H]^+$ calcd for $C_{29}H_{32}IN_2O_4S$: 631.1122; found: 631.1123.

m.p. 194 – 195 °C.

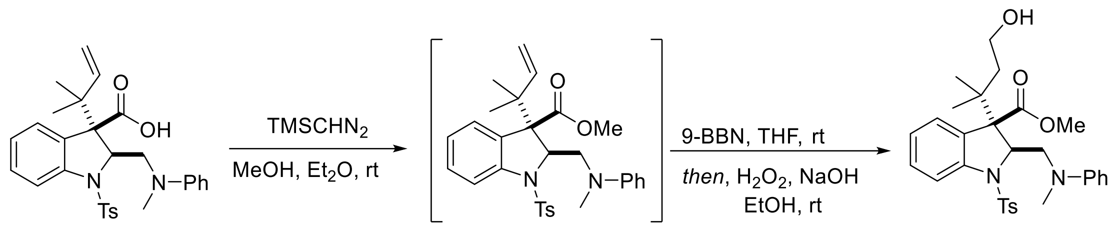

3d **12**

To a solution of **3d** (100.8 mg, 0.2 mmol, 1.0 equiv) in MeOH (1.0 mL) and Et_2O (4.0 mL) was added $TMSCHN_2$ (2.0 M in hexane, 0.5 mL, 1.0 mmol, 5.0 equiv.) slowly at room temperature. After stirring for 1 h, the reaction mixture was concentrated under reduced pressure. The residue was purified by column chromatography (Petroleum ether / ethyl acetate = 20: 1) to afford the methyl-indoline (97 mg, 0.187 mmol, 93%).

The above methyl-indoline (97 mg, 0.187 mmol) was dissolved in 9-BBN (0.5 M in tetrahydrofuran, 1.7 mL, 0.85 mmol) under an argon atmosphere, and the resulting solution was stirred 24 h at room temperature. The reaction was diluted with ethanol (6.0 mL) and treated with 4 M sodium hydroxide (1.0 mL). Then 30% hydrogen peroxide (1.2 mL) was added dropwise at 0 °C. The reaction was stirred at 0 °C for 1 h, which was then quenched by addition of saturated NH_4Cl. The mixture was extracted with diethyl ether. The combine organic phases were washed with brine, dried over anhydrous Na_2SO_4, and concentrated under reduced pressure. The residue was purified by column chromatography (Petroleum ether / ethyl acetate = 5: 1) to afford product **12** (71.0 mg, 0.132 mmol, 66% yield, over two steps).

Methyl-2-((methyl(phenyl)amino)methyl)-3-(2-methylbut-3-en-2-yl)-1-tosylindoline-3-carboxylate

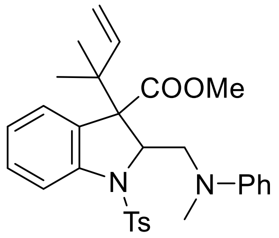

Methyl-indoline was obtained as a lightly yellow oil (97 mg, 93% yield).

TLC: R_f = 0.4 (Petroleum ether /ethyl acetate 10:1).

^{1}H NMR (400 MHz, $CDCl_3$) δ 7.71 (d, *J* = 8.3 Hz, 2H), 7.53 (d, *J* = 8.0 Hz, 1H), 7.46 (d, *J* = 7.7 Hz, 1H), 7.38 – 7.32 (m, 1H), 7.13 – 7.08 (m, 5H), 6.75 – 6.67 (m, 1H), 6.52 (d, *J* = 8.0 Hz, 2H), 6.19 (dd, *J* = 17.5, 10.9 Hz, 1H), 5.18 (t, *J* = 6.6 Hz, 1H), 5.07 (dd, *J* = 10.9, 1.0 Hz, 1H), 4.99 (dd, *J* = 17.5, 1.0 Hz, 1H), 3.52 (s, 3H), 3.36 (d, *J* = 6.5 Hz, 2H), 2.59 (s, 3H), 2.32 (s, 3H), 1.00 (s, 3H), 0.87 (s, 3H).

^{13}C NMR (101 MHz, $CDCl_3$) δ 171.3, 150.6, 143.9, 143.5, 141.9, 137.9, 131.6, 129.3, 128.8, 128.7, 128.6, 126.9, 122.8, 117.9, 114.9, 114.4, 114.3, 65.6, 65.0, 55.0, 51.7, 44.0, 39.7, 24.1, 22.6, 21.5.

HRMS: m/z $[M+H]^+$ calcd for $C_{30}H_{35}N_2O_4S$: 519.2312; found: 519.2315.

methyl-3-(4-hydroxy-2-methylbutan-2-yl)-2-((methyl(phenyl)amino)methyl)-1-tosylindoline-3-carboxylate (12)

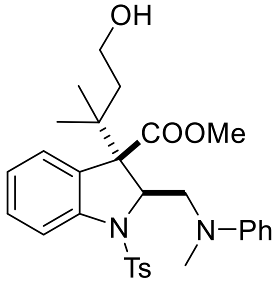

Product **12** was obtained as a colorless oil (71 mg, 66% yield).

TLC: R_f = 0.2 (Petroleum ether /ethyl acetate 2:1).

^{1}H NMR (500 MHz, $CDCl_3$) δ 7.68 (d, *J* = 8.3 Hz, 2H), 7.49 (d, *J* = 8.1 Hz, 2H), 7.36 – 7.32 (m, 1H), 7.12 – 7.08 (m, 3H), 7.06 (d, *J* = 8.2 Hz, 2H), 6.73 – 6.67 (m, 1H), 6.49 (d, *J* = 8.2 Hz, 2H), 5.23 (dd, *J* = 8.6, 5.0 Hz, 1H), 3.58 – 3.52 (m, 5H), 3.37 – 3.28 (m, 2H), 2.55 (s, 3H), 2.29 (s, 3H), 1.91 – 1.83 (m, 1H), 1.46 – 1.39 (m, 1H), 0.94 (s, 3H), 0.93 (s, 3H).

^{13}C NMR (101 MHz, $CDCl_3$) δ 171.6, 150.4, 143.5, 141.8, 137.8, 131.6, 129.3, 129.0, 128.9, 128.7, 126.9, 123.1, 117.9, 115.1, 114.1, 66.3, 64.6, 59.6, 55.4, 51.8, 40.4, 39.8, 39.7, 23.1, 23.0, 21.5.

HRMS: m/z $[M+H]^+$ calcd for $C_{30}H_{37}N_2O_5S$: 537.2418; found: 537.2419.

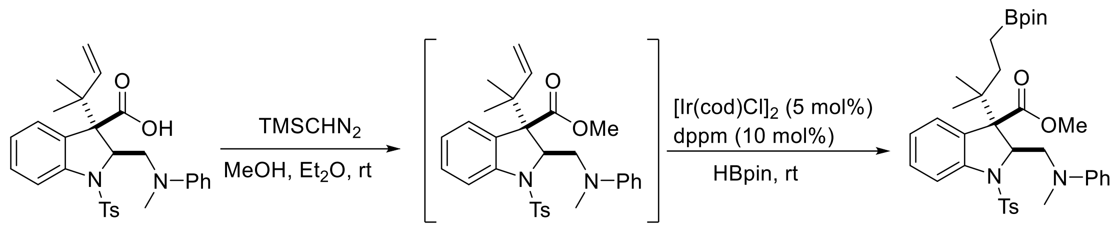

3d **13**

To a solution of **3d** (100.8 mg, 0.2 mmol, 1.0 equiv) in MeOH (1.0 mL) and Et_2O (4.0 mL) was added $TMSCHN_2$ (2.0 M in hexane, 0.5 mL, 1.0 mmol, 5.0 equiv.) slowly at room temperature. After stirring for 1 h, the reaction mixture was concentrated under reduced pressure. The residue was purified by column chromatography (Petroleum ether / ethyl acetate = 20: 1) to afford the methyl-indoline.

Under argon atmosphere, the tube was charged with $[Ir(cod)Cl]_2$ (6.7 mg, 0.01 mmol, 0.05 equiv.), bis(diphenylphosphino)methane (7.7 mg, 0.02 mmol, 0.1 equiv.) and anhydrous CH_2Cl_2 (1.0 mL). The reaction mixture was stirred for 30 min at room temperature. After that, a solution of the above methyl-indoline in anhydrous CH_2Cl_2 (5.0 mL) was added, then HBpin (145 µL, 1.0 mmol, 5.0 equiv.) was added at room temperature. The tesulting solution was stirred overnight. The reaction mixture was quenched with MeOH (1.0 mL) and concentrated under reduced pressure. The residue was purificed by column chromatography on silica gel (Petroleum ether / ethyl acetate = 30: 1) to afford product **13** (105 mg, 0.16 mmol, 81% yield, over two steps).

methyl 2-((methyl(phenyl)amino)methyl)-3-(2-methyl-4-(4,4,5,5-tetramethyl-1,3,2-dioxaborolan-2-yl)butan-2-yl)-1-tosylindoline-3-carboxylate (13)

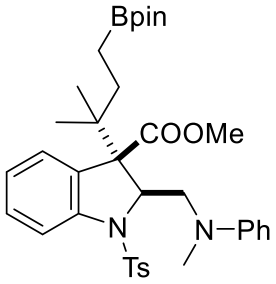

Product **13** was obtained as a lightly yellow oil (105 mg, 81% yield).

TLC: R_f = 0.3 (Petroleum ether /ethyl acetate 20:1).

^{1}H NMR (500 MHz, $CDCl_3$) δ 7.68 (d, *J* = 8.3 Hz, 2H), 7.53 (d, *J* = 7.1 Hz, 1H), 7.49 (d, *J* = 8.0 Hz, 1H), 7.34 – 7.28 (m, 1H), 7.12 – 7.06 (m, 3H), 7.04 (d, *J* = 8.2 Hz, 2H), 6.70 – 6.64 (m, 1H), 6.46 (d, *J* = 8.1 Hz, 2H), 5.28 (dd, *J* = 9.2, 4.2 Hz, 1H), 3.56 (s, 3H), 3.37 (dd, *J* = 14.3, 4.2 Hz, 1H), 3.25 (dd, *J* = 14.3, 9.3 Hz, 1H), 2.55 (s, 3H), 2.28 (s, 3H), 1.87 – 1.81 (m, 1H), 1.42 – 1.36 (m, 1H), 1.24 (s, 6H), 1.23 (s, 6H), 0.94 (s, 3H), 0.68 (s, 3H), 0.64 (t, *J* = 8.2 Hz, 2H).

^{13}C NMR (126 MHz, $CDCl_3$) δ 171.9, 150.7, 143.3, 141.9, 138.2, 132.2, 129.4, 129.3, 128.8, 128.6, 127.0, 122.9, 117.7, 115.1, 114.0, 83.1, 66.6, 64.9, 55.5, 51.6, 42.0, 39.5, 30.7, 25.0, 24.9, 22.1, 22.0, 21.5.

HRMS: m/z $[M+H]^+$ calcd for $C_{36}H_{48}BN_2O_6S$: 647.3321; found: 647.3326.

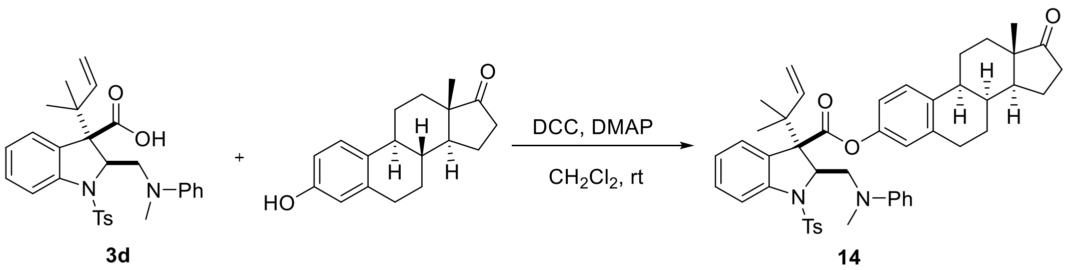

DCC (62.0 mg, 0.36 mmol, 1.8 equiv.) and DMAP (26 mg, 0.1 mmol, 0.5 equiv.) were added to the solution of **3d** (100.8 mg, 0.2 mmol, 1.0 equiv.) in dichloromethane (4.0 mL) at room temperature. The resultant mixture was stirred for 10 min, then the estrone (81mg, 0.3 mmol, 1.5 equiv.) was added and stirred for 8 h. The reaction mixture was filtered and the organic layer was washed with 4% hydrochloric acid, saturated aqueous sodium bicarbonate and brine, dried over sodium sulfate. After filtration and concentration in vacuo, the resulting residue was purified by silica gel flash column chromatography (Petroleum ether / ethyl acetate = 5: 1) to afford **14** (134 mg, 0.177 mmol, 89%).

(8*S*,9*S*,13*S*,14*S*)-13-methyl-17-oxo-7,8,9,11,12,13,14,15,16,17-decahydro-6H-cyclopenta[*a*]phenanthren-3-yl-2-((methyl(phenyl)amino)methyl)-3-(2-methylbut-3-en-2-yl)-1-tosylindoline-3-carboxylate (14)

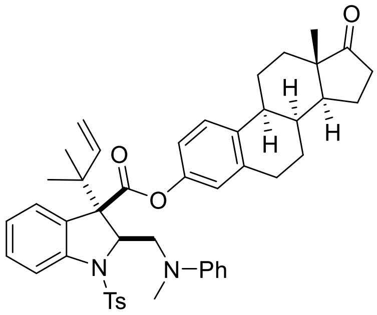

Product **14** was obtained as a lightly yellow oil (134 mg, 89% yield).

TLC: R_f = 0.4 (Petroleum ether /ethyl acetate 5:1).

^{1}H NMR (400 MHz, $CDCl_3$) δ 7.74 (d, *J* = 8.2 Hz, 2H), 7.71 (d, *J* = 7.6 Hz, 1H), 7.61 (d, *J* = 8.1 Hz, 1H), 7.47 – 7.41 (m, 1H), 7.31 (d, *J* = 8.2 Hz, 1H), 7.21 – 7.09 (m, 5H), 6.88 (d, *J* = 8.5 Hz, 1H), 6.81 – 6.73 (m, 2H), 6.56 (d, *J* = 8.3 Hz, 2H), 6.36 (dd, *J* = 17.4, 10.8 Hz, 1H), 5.42 (dd, *J* = 9.6, 3.7 Hz, 1H), 5.22 (d, *J* = 11.2 Hz, 1H), 5.17 (d, *J* = 17.5 Hz, 1H), 3.64 (dd, *J* = 14.2, 3.6 Hz, 1H), 3.49 (dd, *J* = 14.1, 9.8 Hz, 1H), 2.94 – 2.87 (m, 2H), 2.62 (s, 3H), 2.59 – 2.51 (m, 1H), 2.46 – 2.40 (m, 1H), 2.35 (s, 3H), 2.32 – 2.26 (m, 1H), 2.26 – 2.16 (m, 1H), 2.12 – 1.99 (m, 3H), 1.68 – 1.45 (m, 6H), 1.21 (s, 3H), 1.13 (s, 3H), 0.95 (s, 3H).

^{13}C NMR (101 MHz, $CDCl_3$) δ 220.8, 169.8, 150.3, 148.2, 143.5, 143.4, 142.0, 138.3, 138.0, 137.8, 131.3, 129.3, 129.1, 128.9, 128.8, 126.9, 126.5, 123.1, 121.3, 118.4, 117.8, 115.5, 114.9, 113.9, 65.5, 65.3, 55.6, 50.4, 48.0, 44.4, 44.2, 39.5, 38.0, 35.9, 31.6, 29.5, 26.3, 25.8, 24.3, 22.9, 21.6, 21.5, 13.9.

HRMS: m/z $[M+H]^+$ calcd for $C_{47}H_{53}N_2O_5S$: 757.3670; found: 757.3671.

m.p. 94 – 95 ^{o}C.

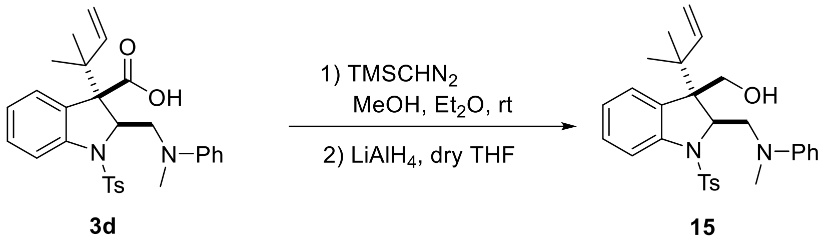

To a solution of **3d** (100.8 mg, 0.2 mmol, 1.0 equiv.) in MeOH (1.0 mL) and Et_2O (4.0 mL) was added $TMSCHN_2$ (2.0 M in hexanes, 0.5 mL, 1.0 mmol, 5.0 equiv.) slowly at room temperature. After stirring for 1 h, all volatiles were removed under reduced pressure and the compound was subsequently used without further purification.

The above mixture was dissolved in dry THF (1.0 mL), which was added into the stirred suspension of $LiAlH_4$ (23.5 mg, 0.6 mmol, 3.0 equiv.) in THF (1.0 mL) at 0 ^oC under an Ar. The reaction mixture was stirred at room tempreture for 2 h. Then it was quenched by water and 2.0 M HCl and extracted by ethyl acetate. The combined organic layers were dried over anhydrous Na_2SO_4, filtered and concentrated under reduced pressure. The residue was purified by column chromatography (Petroleum ether / ethyl acetate = 10: 1) to afford product **15** (59.0 mg, 0.12 mmol, 60% yield, over two steps).

(2-((methyl(phenyl)amino)methyl)-3-(2-methylbut-3-en-2-yl)-1-tosylindolin-3-yl)methanol (15)

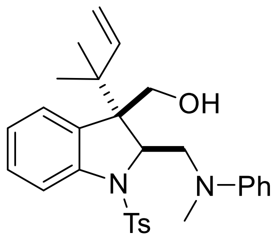

Product **15** was obtained as a yellow oil (59 mg, 60% yield).

TLC: R_f = 0.4 (Petroleum ether /ethyl acetate 5:1).

^{1}H NMR (400 MHz, $CDCl_3$) δ 7.82 (d, *J* = 7.8 Hz, 2H), 7.67 (d, *J* = 7.8 Hz, 1H), 7.41 – 7.35 (m, 2H), 7.31 – 7.23 (m, 5H), 7.12 – 6.99 (m, 3H), 6.18 – 6.04 (m, 1H), 4.83 (d, *J* = 8.3 Hz, 1H), 4.73 – 4.61 (m, 2H), 4.17 (d, *J* = 13.1 Hz, 1H), 3.96 (d, *J* = 13.1 Hz, 1H), 3.85 (d, *J* = 13.2 Hz, 1H), 3.65 – 3.53 (m, 1H), 2.96 (s, 3H), 2.40 (s, 3H), 0.86 (s, 3H), 0.31 (s, 3H).

^{13}C NMR (101 MHz, $CDCl_3$) δ 150.7, 145.8, 144.3, 141.7, 136.7, 132.8, 129.8, 129.3, 128.8, 127.3, 125.4, 123.2, 122.6, 119.9, 114.5, 112.9, 64.9, 64.1, 58.9, 56.2, 44.5, 43.8, 23.5, 22.2, 21.6.

HRMS: m/z $[M+H]^+$ calcd for $C_{29}H_{35}N_2O_3S$: 491.2363; found: 491.2364.

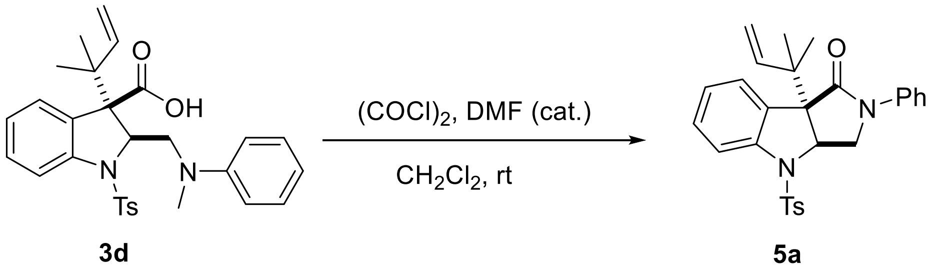

The compound **3d** (100.8 mg, 0.2 mmol, 1.0 equiv) was dissolved in dry CH_2Cl_2 (5 mL), and oxalyl chloride (38 μL, 0.45 mmol, 1.5 equiv.) was added dropwise at 0°C under the argon atmosphere. Afterwards, DMF (dry, 5 drops) was added, and the reaction mixture was stirred for 3 h at room temperature. Then it was concentrated under reduced pressure. The residue was purified by column chromatography (Petroleum ether / ethyl acetate = 30: 1) to afford product **5a** (87.0 mg, 0.184 mmol, 80% yield).

8b-(2-methylbut-3-en-2-yl)-2-phenyl-4-tosyl-3,3a,4,8b-tetrahydropyrrolo*[3,4-b]*indol-1(2*H*)-one (5a)

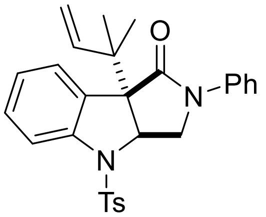

Product **5a** was obtained as a yellow solid (87 mg, 80% yield).

TLC: R_f = 0.4 (Petroleum ether /ethyl acetate 20:1).

^{1}H NMR (400 MHz, $CDCl_3$) δ 7.82 (d, *J* = 8.3 Hz, 2H), 7.73 (d, *J* = 8.1 Hz, 1H), 7.68 (d, *J* = 0.8 Hz, 1H), 7.64 – 7.58 (m, 2H), 7.40 – 7.34 (m, 2H), 7.34 – 7.29 (m, 3H), 7.20 – 7.14 (m, 1H), 7.09 – 7.03 (m, 1H), 5.68 (dd, *J* = 17.3, 10.7 Hz, 1H), 4.85 (d, *J* = 17.4 Hz, 1H), 4.77 (d, *J* = 10.8 Hz, 1H), 4.59 (dd, *J* = 7.4, 2.6 Hz, 1H), 4.26 (dd, *J* = 11.3, 7.5 Hz, 1H), 4.18 (dd, *J* = 11.3, 2.6 Hz, 1H), 2.42 (s, 3H), 1.10 (s, 3H), 0.80 (s, 3H).

^{13}C NMR (101 MHz, $CDCl_3$) δ 171.2, 144.7, 142.9, 142.1, 138.6, 134.9, 129.8, 129.5, 128.9, 128.8, 127.4, 127.2, 125.2, 123.6, 120.4, 113.8, 113.6, 63.2, 61.4, 55.5, 42.8, 22.5, 22.0, 21.6.

HRMS: m/z $[M+H]^+$ calcd for $C_{28}H_{29}N_2O_3S$: 473.1893; found: 473.1895.

m.p. 136 – 137 ^{o}C.

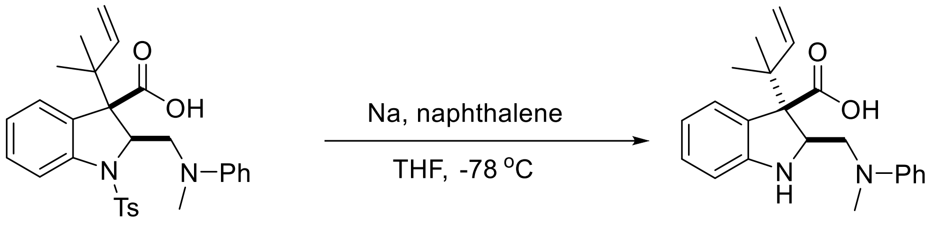

3d **16**

To a solution of naphthalene (256.3 mg, 2 mmol, 10.0 equiv.) in dry THF (2 mL) was added metal sodium (46.0 mg, 2 mmol, 10.0 equiv.) at room temperature. The mixture was stirred for 2 h to yield a deep-blue sodium–naphthalenide solution. The freshly prepared sodium–naphthalenide solution was added slowly to the solution of **3d** (100.8 mg, 0.2 mmol, 1.0 equiv.) in THF (3 mL) at –78 °C until the color of reaction mixture maintained unchanged blue. After 30 min, the mixture was quenched with saturated aqueous NH_4Cl solution (10 mL), warmed to room temperature and diluted with ethyl acetate. The layers were separated and the aqueous phase was extracted with ethyl acetate. The combined organic phases were washed with brine, dried over anhydrous Na_2SO_4, and concentrated in vacuo. The residue was purified by flash column chromatography (Petroleum ether / ethyl acetate = 5: 1) to afford compound **15** (45 mg, 0.129 mmol, 64% yield).

2-((methyl(phenyl)amino)methyl)-3-(2-methylbut-3-en-2-yl)indoline-3-carboxylic acid (16)

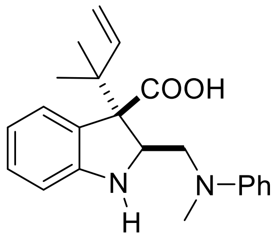

Product **16** was obtained as a yellow oil (45 mg, 64% yield).

TLC: R_f = 0.6 (Petroleum ether /ethyl acetate 5:1).

^{1}H NMR (400 MHz, $CDCl_3$) δ 7.95 – 7.65 (br, 1H), 7.59 (d, *J* = 7.4 Hz, 1H), 7.31 – 7.21 (m, 3H), 6.92 – 6.86 (m, 1H), 6.85 – 6.81 (m, 1H), 6.78 (d, *J* = 8.1 Hz, 2H), 6.66 (d, *J* = 7.7 Hz, 1H), 6.35 (dd, *J* = 17.4, 10.8 Hz, 1H), 5.19 (dd, *J* = 10.9, 0.9 Hz, 1H), 5.12 (dd, *J* = 17.4, 0.9 Hz, 1H), 4.32 (dd, *J* = 11.1, 3.3 Hz, 1H), 3.55 (dd, *J* = 14.0, 3.3 Hz, 1H), 3.29 (dd, *J* = 13.8, 11.2 Hz, 1H), 2.99 (s, 3H), 1.26 (s, 3H), 1.19 (s, 3H).

^{13}C NMR (101 MHz, $CDCl_3$) δ 178.1, 150.4, 150.3, 144.4, 129.4, 129.1, 128.7, 126.9, 118.3, 117.7, 113.7, 113.2, 110.1, 65.8, 60.3, 55.8, 44.1, 39.1, 24.1, 22.6.

HRMS: m/z $[M+H]^+$ calcd for $C_{22}H_{27}N_2O_2$: 351.2067; found: 351.2067.

5. Supplementary discussions

5.1. Exploration of other radical precursors

We investigated some other radical precursors to explore the structural diversity. It was found that the acid derivative (*N*-phenylglycine) underwent hydroalkylative dearomatization of indole **1d** smoothly, however, the Ireland-Claisen rearrangement was completely suppressed (Supplementary Fig. 2a). On the basis of a related work by Glorius (*Chem. Sci.* **2021**, *12*, 2816–2822) using boronic esters as radical precursors, we conducted a similar experimental procedure with *N*-Ts indole **1d** and cyclohexylboronic ester, affording the desired dearomative reverse-prenylation product in 32% yield over 3 steps (Supplementary Fig. 2b).

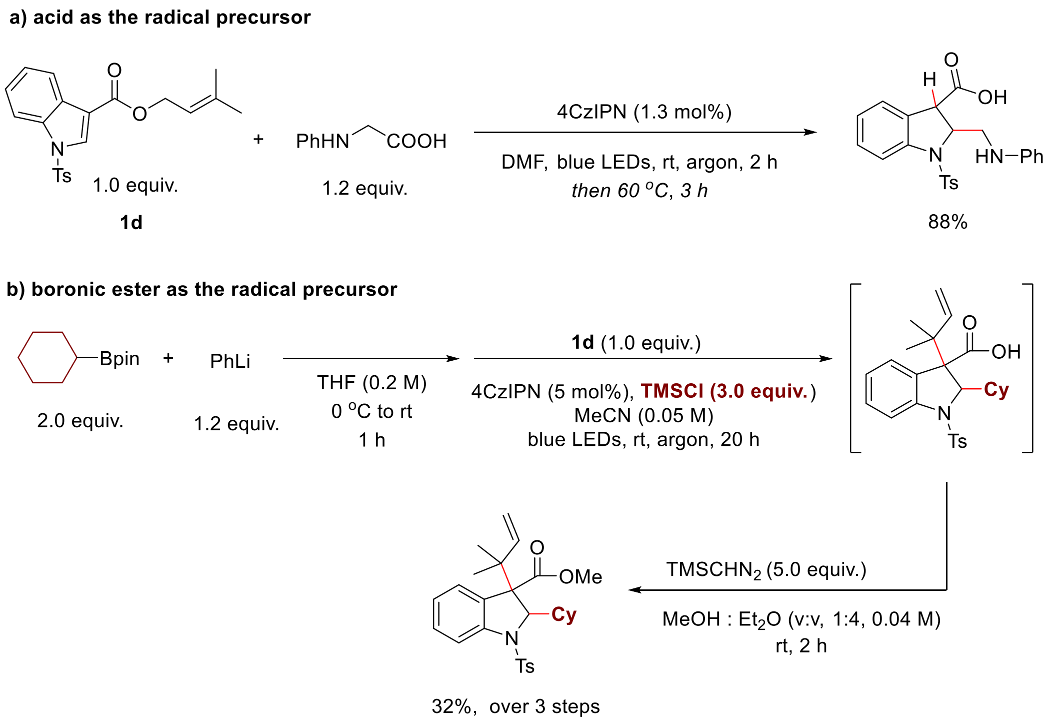

Supplementary Fig. 2. Investigation of N-phenylglycine and cyclohexylboronic ester.

When employing the benzyl trifluoroborate as a radical precursor, the dearomatizaiton/rearrangement product was not detected, but an unexpected desulfonylation of the *N*-Ts indole happened even in the presence of TMSCl (Supplementary Fig. 3a). Besides, we also attempted to evaluate the reaction of benzyl silane and *N*-Ts indole **1d**. Under the standard conditions, no desired product was determined. Further optimization of the reaction conditions by varying photocatalysts

and solvents was still failed to obtain the corresponding product, probably due to the high oxidation potential of benzyl silanes (Supplementary Fig. 3b).

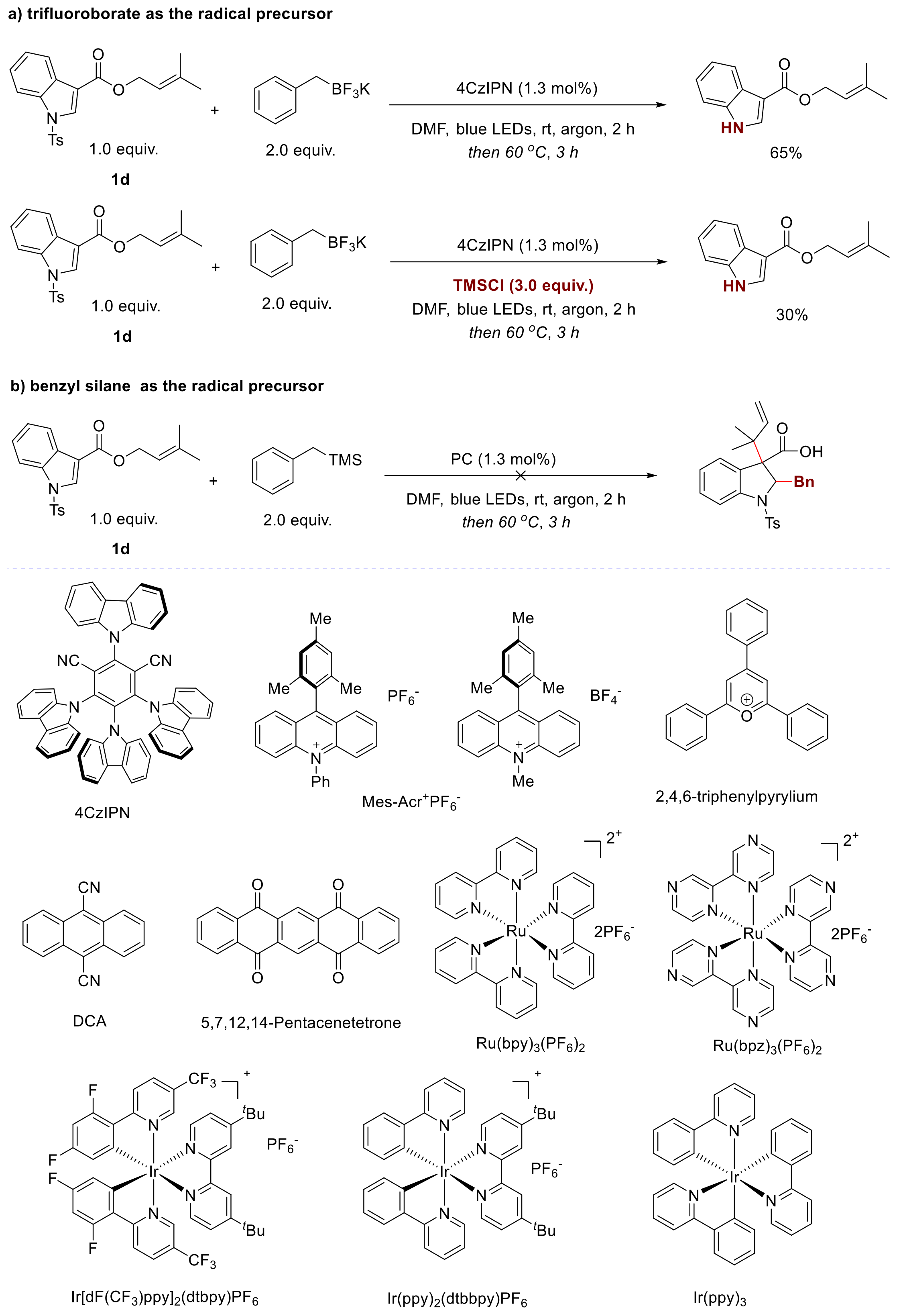

Supplementary Fig. 3. Investigation of benzyl trifluoroborate.

5.2. Mechanistic study

5.2.1 Radical trapping experiment with TEMPO

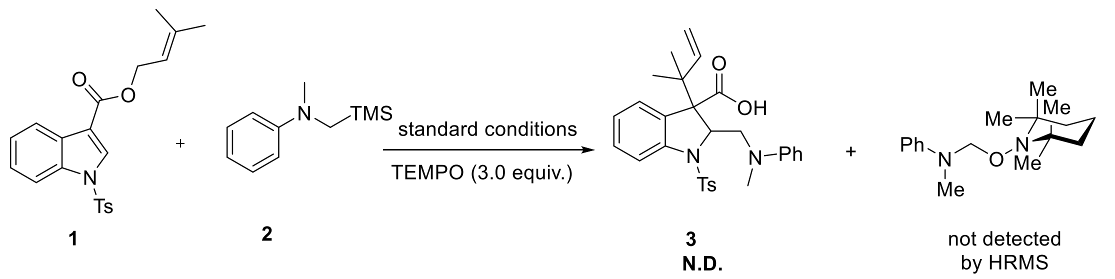

In the glovebox, to a 10 mL reaction vial equipped with a stir bar were added 3-methylbut-2-en-1-yl-1-tosylindoline-3-carboxylate **1** (77.0 mg, 0.2 mmol, 1.0 equiv), 4-CzIPN (2.0 mg, 0.0026 mmol, 0.013 equiv) and TEMPO (94.0 mg, 0.6 mmol, 3.0 equiv.) in dry DMF (0.5 mL). Then the solution of *N*-methyl-*N*-((trimethylsilyl)methyl)aniline **2** (38.0 mg, 0.24 mmol, 1.2 equiv) in dry DMF (0.5 mL) was added. The vial was sealed, transferred out of the glove box, and then irradiated with a 1 W blue LED lamp for 2 h at room temperature. Afterwards, the reaction mixture was allowed to heat at 60 °C for 3 h without light. When the reaction was completed (monitored by TLC), the reaction was quenched by saturated aqueous NH_4Cl solution (2mL), extracted with ethyl acetate (10 mL x 2). The combined organic layers were washed with H_2O (10 mL x 2) and brine (10 mL), dried over anhydrous Na_2SO_4, filtered, and concentrated in vacuo. The crude residue was detected by ^{1}H NMR and HRMS analysis. None of targeted product **3** was found and the expected TEMPO radical adduct wasn't detected by HRMS. The complete inhibition of the reaction suggested a possible radical reaction pathway.

5.2.2 Radical trapping experiment with (allylsulfonyl)benzene

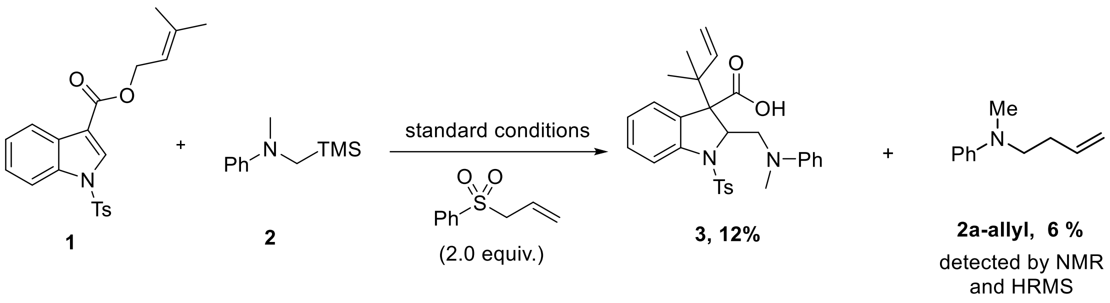

In the glovebox, to a 10 mL reaction vial equipped with a stir bar were added 3-methylbut-2-en-1-yl-1-tosylindoline-3-carboxylate **1** (77.0 mg, 0.2 mmol, 1.0 equiv), 4-CzIPN (2.0 mg, 0.0026 mmol, 0.013 equiv) and (allylsulfonyl)benzene (73.0 mg, 0.9 mmol, 2.0 equiv.) in dry DMF (0.5 mL). Then the solution of *N*-methyl-*N*-((trimethylsilyl)methyl)aniline **2** (38.0 mg, 0.24 mmol, 1.2 equiv) in dry DMF (0.5 mL) was added. The vial was sealed, transferred out of the glove box, and then irradiated with a 1 W blue LED lamp for 2 h at room temperature. Afterwards, the reaction mixture was allowed to heat at 60 °C for 3 h without light. When the reaction was completed (monitored by TLC), the reaction was quenched by saturated aqueous NH_4Cl solution (2mL) and extracted with ethyl acetate (10 mL x 2). The combined organic layers were washed with H_2O (10 mL x 2) and brine (10 mL), dried over Na_2SO_4, filtered, and concentrated in vacuo. The crude residue was detected by ^{1}H NMR and HRMS analysis, and 12 % yield of targeted product **3** was found. In addition, the residue **2a-allyl** was examined through HRMS analysis (m/z calcd for $C_{11}H_{15}N$ $[M+H]^+$: 162.1277; found: 162.1278). The decreased yield of **3** and the expected radical-adduct **2a-allyl** further confirmed a possible radical reaction pathway.

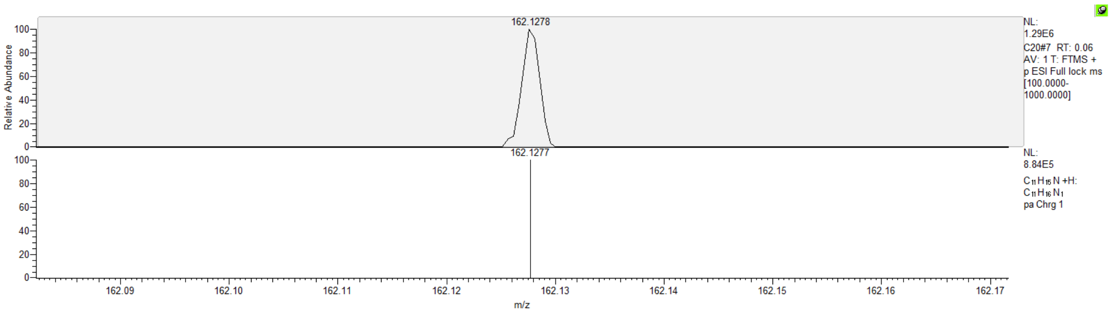

5.2.3 The intermediate confirmation experiment

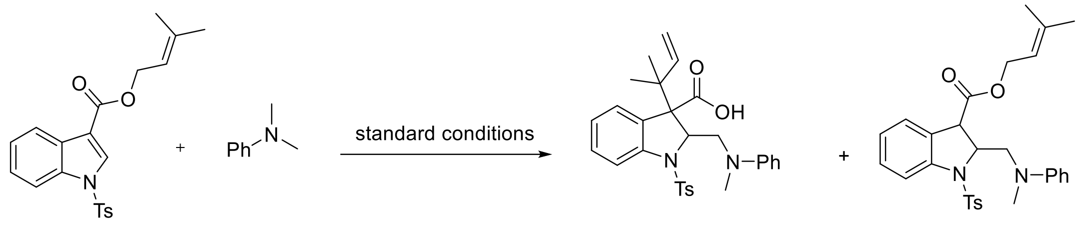

In the glovebox, to a 10 mL reaction vial equipped with a stir bar were added 3-methylbut-2-en-1-yl-1-tosylindoline-3-carboxylate **1** (77.0 mg, 0.2 mmol, 1.0 equiv) 4-CzIPN (2.0 mg, 0.0026 mmol, 0.013 equiv) and dry DMF (0.5 mL). Then the solution of *N,N*-dimethylaniline **2'** (29.0 mg, 0.24 mmol, 1.2 equiv) in dry DMF (0.5 mL) was added. The vial was sealed, transferred out of the glove box, and then irradiated with a 1 W blue LED lamp for 2 h at room temperature. Afterwards, the reaction mixture was allowed to heat at 60 °C for 3 h without light. When the reaction was completed (monitored by TLC), the reaction was quenched by saturated aqueous NH_4Cl solution (2mL) and extracted with ethyl acetate (10 mL x 2). The combined organic layers were washed with H_2O (10 mL x 2) and brine (10 mL), dried over anhydrous Na_2SO_4, filtered, and concentrated in vacuo. The residue was detected by ^{1}H NMR analysis, and none of the targeted product **3** was found. However, the hydro-aminoalkylated product **3'** was isolated by flash column chromatography on silica gel (36 mg, 0.072 mmol, 36% yield).

3-methylbut-2-en-1-yl-2-((methyl(phenyl)amino)methyl)-1-tosylindoline-3-carboxylate (3')

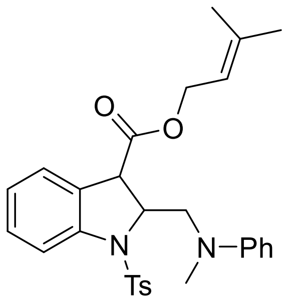

Product **3'** was obtained as a yellow oil (36 mg, 36% yield).

TLC: R_f = 0.2 (Petroleum ether /ethyl acetate 20:1).

^{1}H NMR (400 MHz, $CDCl_3$) δ 7.72 (d, *J* = 8.1 Hz, 1H), 7.64 (d, *J* = 8.3 Hz, 2H), 7.37 – 7.28 (m, 3H), 7.26 (d, J = 7.4 Hz, 1H), 7.18 (d, J = 8.1 Hz, 2H), 7.10 (t, *J* = 7.5 Hz, 1H), 6.83 (d, *J* = 8.2 Hz, 2H), 6.78 (t, *J* = 7.2 Hz, 1H), 5.10 – 4.99 (m, 2H), 4.31 – 4.20 (m, 2H), 3.94 (dd, *J* = 14.6, 5.0 Hz, 1H), 3.83 (d, *J* = 2.5 Hz, 1H), 3.49 (dd, *J* = 14.6, 9.7 Hz, 1H), 3.07 (s, 3H), 2.35 (s, 3H), 1.73 (s, 3H), 1.59 (s, 3H).

^{13}C NMR (101 MHz, $CDCl_3$) δ 170.1, 149.5, 144.0, 141.2, 139.2, 134.2, 129.5, 129.3, 129.2, 128.4, 127.6, 126.8, 124.7, 118.0, 117.2, 116.5, 112.7, 62.7, 62.2, 56.8, 49.4, 39.6, 25.7, 21.6, 18.0.

HRMS: m/z $[M+H]^+$ calcd for $C_{29}H_{33}N_2O_4S$: 505.2156; found: 505.2156.

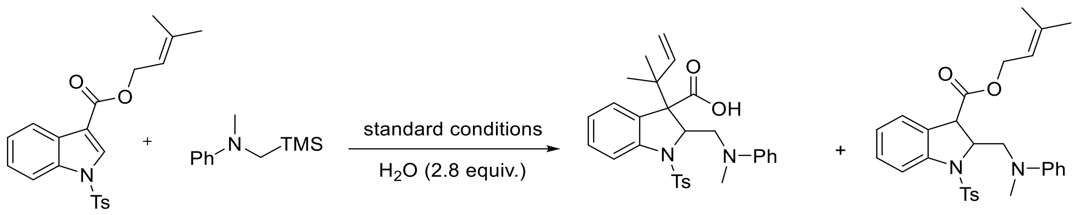

1 **2** **3**, **N.D.** **3'**, **80%**

In the glovebox, to a 10 mL reaction vial equipped with a stir bar were added 3-methylbut-2-en-1-yl-1-tosylindoline-3-carboxylate **1** (77.0 mg, 0.2 mmol, 1.0 equiv), 4-CzIPN (38.0 mg, 0.0026 mmol, 0.013 equiv) and dry DMF (0.5 mL). Then the solution of *N*-methyl-*N*-((trimethylsilyl)methyl)aniline **2** (29.0 mg, 0.24 mmol, 1.2 equiv) in dry DMF (0.5 mL) and H_2O (10.0 µL, 0.56 mmol, 2.8 equiv.) were added. The vial was sealed, transferred out of the glove box, and then irradiated with a 1 W blue LED lamp for 2 h at room temperature. Afterwards, the reaction mixture was allowed to heat at 60 °C for 3 h without light. When the reaction was completed (monitored by TLC), the reaction was quenched by saturated aqueous NH_4Cl solution (2mL) and extracted with ethyl acetate (10 mL x 2). The combined organic layers were washed with H_2O (10 mL x 2) and brine (10 mL), dried over anhydrous Na_2SO_4, filtered, and concentrated in vacuo. The residue was detected by ^1H NMR analysis, and none of targeted product **3** was found. However, the hydro-aminoalkylated product **3'** was isolated by flash column chromatography on silica gel (81 mg, 0.16 mmol, 80% yield).

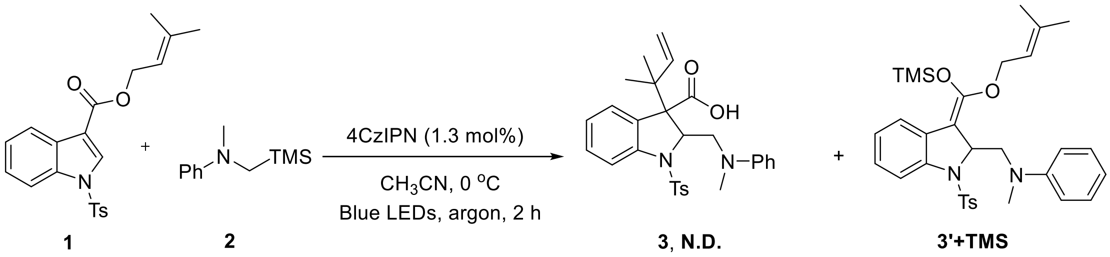

In the glovebox, to a 10 mL reaction vial equipped with a stir bar were added 3-methylbut-2-en-1-yl-1-tosylindoline-3-carboxylate **1** (77.0 mg, 0.2 mmol, 1.0 equiv), 4-CzIPN (38.0 mg, 0.0026 mmol, 0.013 equiv) and dry CH_3CN (0.5 mL). Then the solution of *N*-methyl-*N*-((trimethylsilyl)methyl)aniline **2** (38.0 mg, 0.24 mmol, 1.2 equiv) in dry CH_3CN (0.5 mL) was added. The vial was sealed, transferred out of the glove box, and then irradiated with a 1 W blue LED lamp for 2 h at 0 °C. The reaction mixture was detected by HRMS analysis. It was found that there was no targeted product **3**, however, the ion-peak of **3d'+TMS** was confirmed (m/z calcd for $C_{32}H_{40}N_2O_4SSi$ $[M+H]^+$: 577.2551; found: 577.2553).

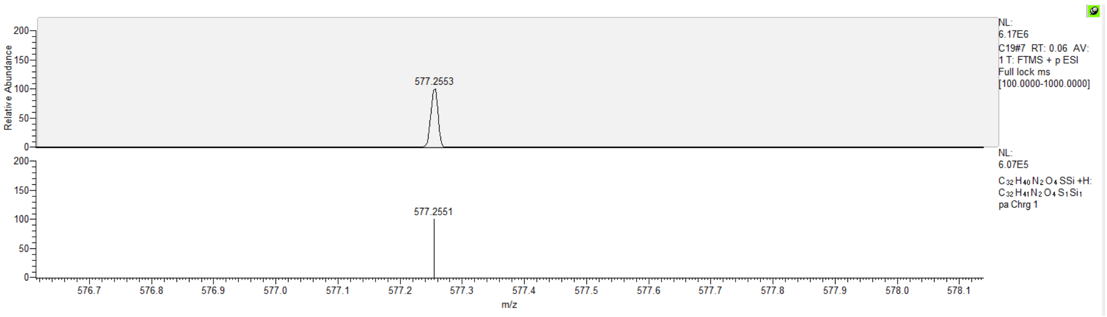

5.2.4 Stern–Volmer Luminescence Quenching Analysis

Stern–Volmer luminescence quenching analysis was carried out to identify quenchers of the excited photoredox catalyst (Supplementary Fig. 4–6). Therefore, the luminescence of the excited photocatalyst is measured in the presence of varying concentrations of potential quencher.

The quenching experiments were tested on an American Perkinelmer LS-55 spectrofluorometer using Bioroyee quartz cuvette. All samples were prepared in an argon-filled glovebox with degassed and dry solvents. The quenching experiments were run with freshly prepared solutions of 1.0 × 10^{-5} M 4CzIPN and the appropriate amount of quencher in CH_3CN at room temperature. The solutions were irradiated at 340 nm and luminescence was measured at 548 nm (CH_3CN as the solvent). The varying concentrations of the potential quencher were achieved by diluting the respective stock solutions in the cuvettes. The samples were sealed with PTFE stoppers and removed from the glovebox for the measurement.

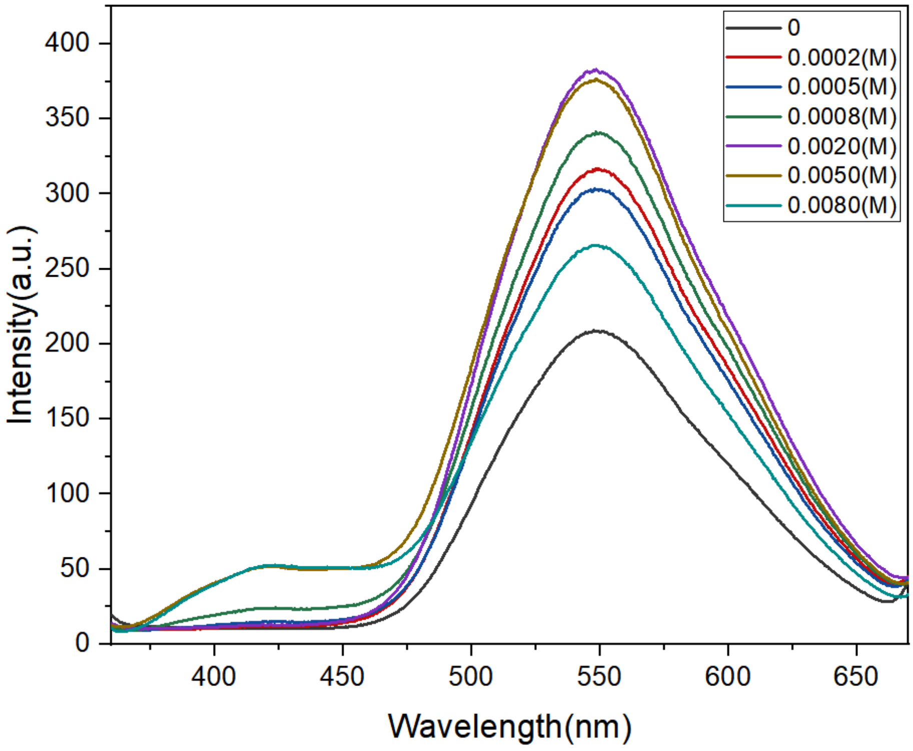

Supplementary Fig. 4. Stein-Volmer luminescence quenching of 4CzIPN with **1d** in CH_3CN.

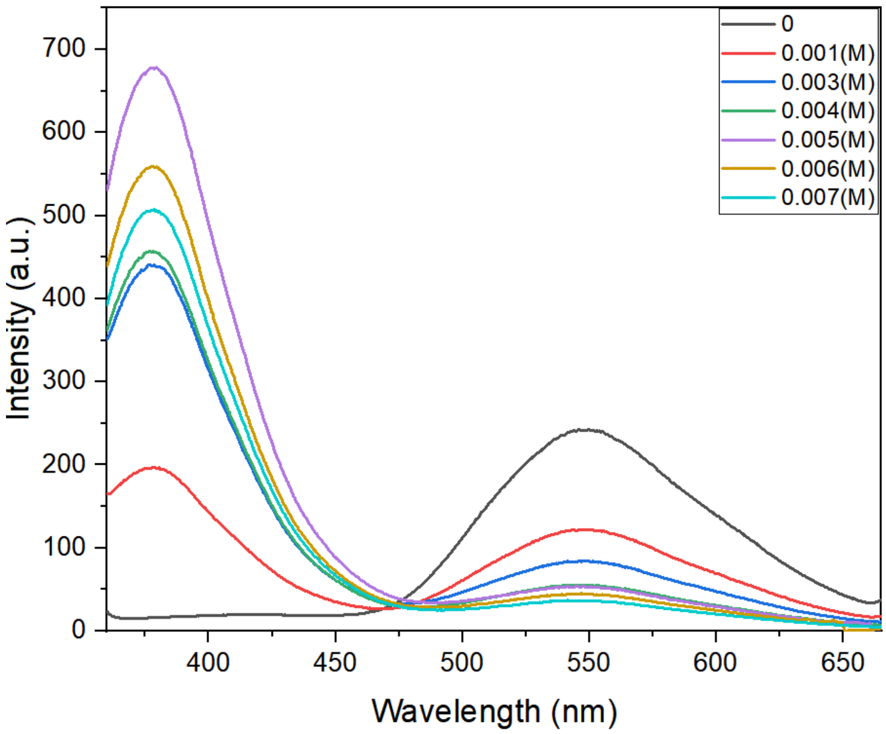

Supplementary Fig. 5. Stein-Volmer luminescence quenching of 4CzIPN with **2a** in CH_3CN.

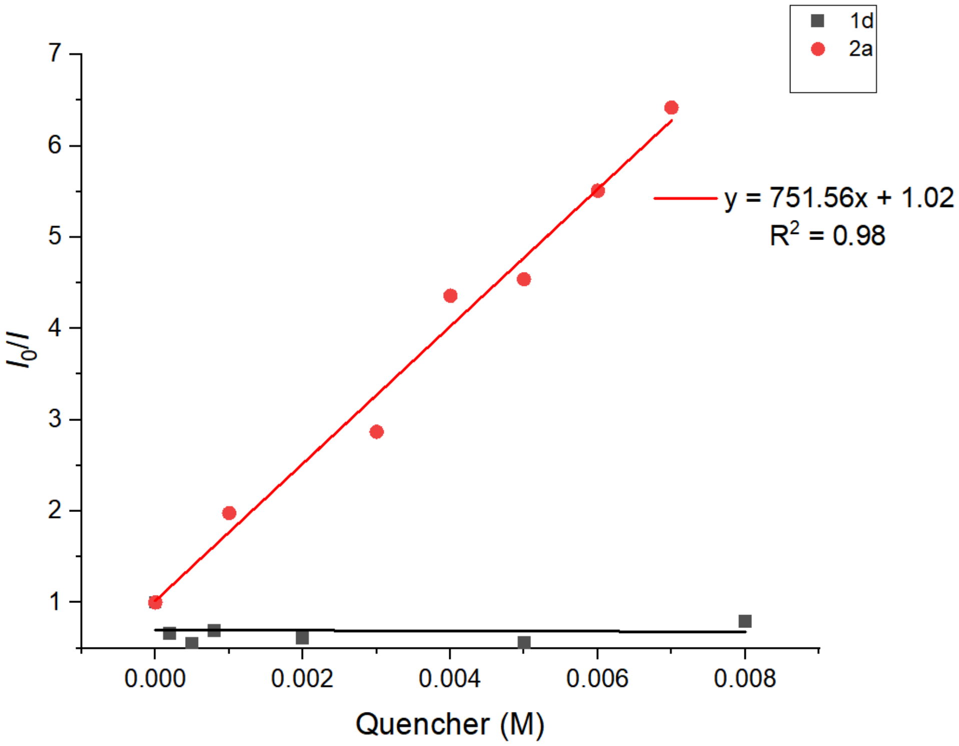

Supplementary Fig. 6. Stein-Volmer luminescence quenching of 4CzIPN with varying concentrations of **2a** (red) and **1d** (black) in CH_3CN.

5.3. Anticancer activity study

Cell culture

MV4-11 cells were cultured in IMEM (Gibco, Milano, Italy). The above media contained 10% fetal bovine serum (FBS) (Invitrogen, Milano, Italy), 100 units/mL penicillin (Gibco, Milano, Italy), and 100 μg/mL streptomycin (Gibco, Milano, Italy). Cells were incubated at 37 °C in a humidified atmosphere of 5% CO_2. The source of the tumor cell lines was from American Type Culture Collection (ATTC). Tumor cells were subcultured and seeded in vitro by State Key Laboratory of Biotherapy and Cancer Center, National Clinical Research Center for Geriatrics, West China Hospital of Sichuan University, China.

Cell viability assay

Cells in logarithmic phase were seeded into 96-well culture plates at densities of 3000-5000 cells per well and subsequently treated with various concentrations of compounds for 72 h in final volumes of 200 μL. Upon end point, 20 μL of MTT (5 mg/mL) was added to each well, and the cells were incubated for an additional 1-3 h. After carefully removal of the medium, the precipitates were dissolved in 150 μL of DMSO via mechanically shaking, and then absorbance values at a wavelength of 570 nm were taken on a spectrophotometer (Molecular Devices, Sunnyvale, USA). IC50 values were calculated using percentage of growth versus untreated control (Supplementary Fig. 7).

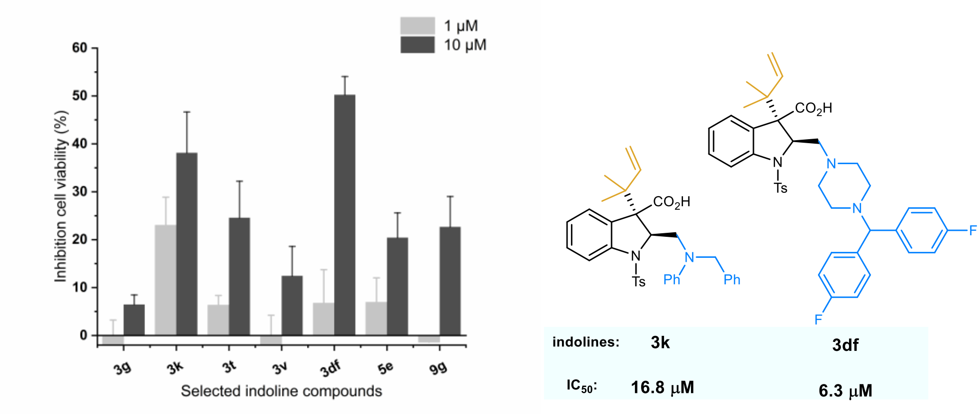

Supplementary Fig. 7. Inhibition of the human leukemia cell line viability (MV-4-11) induced by selected indoline compounds. Experiments were performed in duplicate (n = 3). Standard deviation (SD) values are shown as the error bars.

5.4. X-Ray crystal data

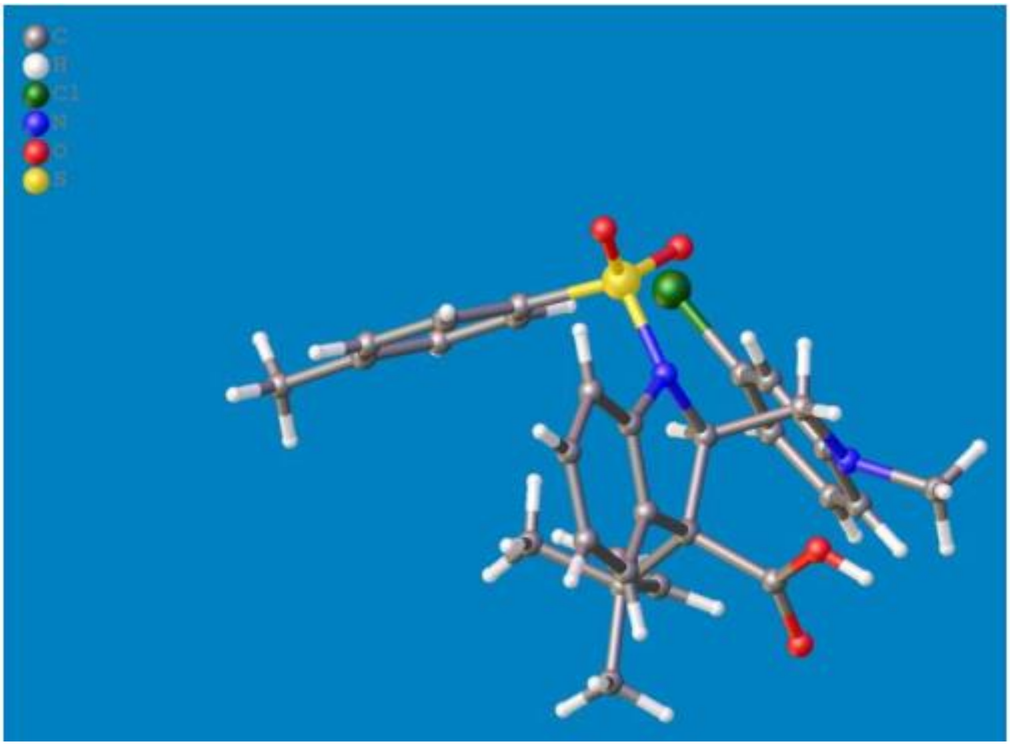

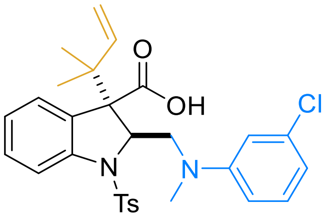

3h

Supplementary Fig. 8: X-ray crystallographic studies of compound **3h**.

Table S1. Crystal data and structure refinement for **3h**.

CCDC	2225579
Empirical formula	$C_{29}H_{29}ClN_2O_4S$
Formula weight	537.05
Temperature	180(2) K
Wavelength	1.54184Å
Crystal system	triclinic
Space group	*P*-1
Unit cell dimensions	
a = 7.98610(10)Å	α= 87.5110(10)°
b = 10.3649(2) Å	β= 83.5660(10)°
c = 17.4993(3) Å	γ = 68.2010(10)°
Volume	1336.45(4) Å
Z	2

Z'	1
Density (calculated)	1.335 g cm^{-3}
μ	2.304 mm^{-1}
Radiation type	CuK$_\alpha$
Min. and max. transmission	2.541 and 76.786
Measured reflections	37514
Independent reflections	5364
Reflections with I >2(I)	5292
Rint	0.0328
Restraints / parameters	0/340
Goodness-of-fit on F_2	1.038
Final R indices [I>2sigma(I)]	R_1 = 0.0399, wR_2 = 0.1065
R indices (all data)	R_1 = 0.0401, wR_2 = 0.1067
Largest diff. peak and hole	0.549 and -0.444 Å^{-3}

Table S2: Fractional Atomic Coordinates (×104) and Equivalent Isotropic Displacement Parameters (Å2×103). *Ueq* is defined as 1/3 of the trace of the orthogonalised *Uij*.

**Atom**	**x**	**y**	**z**	***Ueq***
S10	9725.9(5)	7044.7(4)	3646.1(2)	35.37(13)
Cl30	5434.7(6)	12097.5(4)	2983.3(3)	45.42(14)
O18	10670.1(14)	4757.3(12)	944.3(6)	32.8(3)
O17	7999.8(14)	5797.1(11)	485.9(6)	31.9(2)
O12	9800.6(18)	8340.8(13)	3362.5(7)	43.7(3)
O11	11094.5(18)	6161.0(15)	4095.5(7)	48.5(3)
N1	9796.5(17)	6139.6(13)	2880.9(7)	29.5(3)
N14	9065(2)	8237.2(14)	1050.5(7)	34.4(3)
C16	8913.3(19)	5452.3(14)	1030.1(8)	25.2(3)
C9	8760.8(18)	6857.3(14)	2223.6(8)	23.8(3)
C7	9109.7(19)	4432.6(15)	2330.7(8)	26.2(3)
C8	8149.6(18)	5735.0(14)	1874.3(8)	22.9(3)
C19	5997.9(18)	6183.2(15)	1995.1(8)	25.3(3)
C2	9950(2)	4720.6(16)	2916.9(8)	29.3(3)
C13	9980(2)	7448.8(16)	1692.3(9)	30.2(3)
C29	7314(2)	10158.3(16)	1923.8(9)	32.6(3)
C20	5111.0(19)	7627.2(15)	1668.6(9)	29.2(3)
C6	9254(2)	3076.3(16)	2211.1(10)	34.0(3)
C24	7696(2)	9546.4(16)	1196.1(9)	33.7(3)
C28	5851(2)	11392.7(17)	2065.5(10)	36.5(4)
C22	5397(2)	6127.5(18)	2856.0(9)	33.8(3)
C36	7307(3)	6383.8(19)	4657.3(9)	40.5(4)
C23	5349(2)	5188.4(17)	1590.6(10)	35.0(3)
C31	7596(2)	7392.5(18)	4169.6(9)	35.1(3)
C3	10869(2)	3685.0(19)	3415.9(10)	41.5(4)

C25	6607(3)	10265.7(19)	620.0(11)	44.6(4)
C5	10162(2)	2036.6(17)	2711.6(11)	42.1(4)
C32	6192(3)	8651.8(19)	4063.0(10)	42.4(4)
C21	3897(2)	8712.3(18)	2034.5(12)	42.2(4)
C35	5597(3)	6645(2)	5030.7(10)	46.0(4)
C27	4728(3)	12078.0(18)	1513.5(12)	45.9(4)
C33	4493(3)	8878(2)	4440.3(11)	48.3(5)
C26	5162(3)	11504(2)	784.1(13)	50.4(5)
C34	4159(3)	7882(2)	4922.8(10)	47.0(4)
C4	10937(2)	2351.4(19)	3305.7(11)	46.5(5)
C15	10106(4)	8014(2)	300.2(13)	69.2(7)
C37	2293(3)	8146(3)	5318.4(14)	67.0(7)

Table S3: Anisotropic Displacement Parameters (×104). The anisotropic displacement factor exponent takes the form: *-2π²[h2a*2 × U11+ ... +2hka* × b* × U12]*

**Atom**	***U11***	***U22***	***U33***	***U23***	***U13***	***U12***
S10	37.8(2)	41.9(2)	27.0(2)	-4.10(15)	-8.19(15)	-13.56(18)
Cl30	44.1(2)	35.6(2)	53.1(3)	-8.04(18)	3.19(19)	-12.15(18)
O18	25.6(5)	40.2(6)	23.9(5)	-3.7(4)	2.3(4)	-3.2(4)
O17	29.4(5)	38.1(6)	22.3(5)	-3.2(4)	-1.6(4)	-5.7(4)
O12	54.7(8)	46.3(7)	37.6(6)	-7.7(5)	-6.0(5)	-26.3(6)
O11	44.1(7)	63.1(8)	35.7(6)	-2.5(6)	-17.4(5)	-12.6(6)
N1	31.8(6)	29.8(6)	24.2(6)	-0.6(5)	-7.1(5)	-7.1(5)
N14	44.9(8)	33.8(7)	26.0(6)	1.4(5)	2.2(5)	-18.2(6)
C16	25.5(7)	23.5(7)	24.1(7)	-2.2(5)	0.4(5)	-6.7(5)
C9	22.9(6)	24.5(7)	21.9(6)	-0.4(5)	-2.4(5)	-6.1(5)
C7	22.7(6)	24.4(7)	26.4(7)	1.6(5)	2.3(5)	-4.2(5)
C8	22.2(6)	22.3(6)	21.4(6)	-0.5(5)	0.4(5)	-5.6(5)
C19	22.0(6)	24.9(7)	27.2(7)	-1.1(5)	0.0(5)	-7.3(5)
C2	25.6(7)	29.3(7)	26.4(7)	1.6(6)	0.7(5)	-3.6(6)
C13	27.2(7)	31.6(8)	32.3(7)	-2.0(6)	2.2(6)	-12.8(6)
C29	36.0(8)	28.6(7)	37.0(8)	3.3(6)	-5.9(6)	-16.2(6)
C20	23.5(7)	27.7(7)	34.8(8)	1.7(6)	-3.2(6)	-7.9(6)
C6	29.6(7)	25.8(7)	42.0(9)	0.2(6)	1.9(6)	-6.4(6)
C24	41.4(9)	29.8(8)	36.0(8)	4.3(6)	-4.0(7)	-20.7(7)
C28	38.2(8)	28.2(8)	47.2(9)	-1.4(7)	-3.7(7)	-17.3(7)
C22	27.4(7)	40.1(8)	31.1(8)	1.0(6)	4.7(6)	-11.4(6)
C36	50.2(10)	42.8(9)	24.9(7)	0.5(6)	-7.1(7)	-12.3(8)
C23	29.5(8)	31.6(8)	45.4(9)	-4.1(7)	-4.1(6)	-12.5(6)
C31	40.9(9)	39.3(8)	23.1(7)	-5.2(6)	-5.1(6)	-11.6(7)
C3	38.8(9)	42.5(9)	33.9(8)	8.7(7)	-7.7(7)	-4.0(7)
C25	61.7(12)	42.0(10)	39.8(9)	7.4(7)	-15.2(8)	-28.1(9)

C5	33.6(8)	25.7(8)	57.6(11)	7.4(7)	5.0(8)	-3.7(7)
C32	52.2(10)	35.7(9)	32.9(8)	-3.9(7)	-2.5(7)	-8.8(8)
C21	34.1(8)	30.8(8)	54.4(11)	-0.6(7)	0.2(7)	-5.0(7)
C35	60.7(12)	53.5(11)	27.3(8)	-1.6(7)	-2.5(8)	-25.5(9)
C27	45.8(10)	28.9(8)	66.2(12)	6.2(8)	-18.1(9)	-14.7(7)
C33	47.9(10)	46.3(10)	38.7(9)	-9.2(8)	-3.3(8)	-2.9(8)
C26	59.8(12)	39.4(10)	59.2(12)	15.7(8)	-28.3(10)	-22.2(9)
C34	48.5(10)	62.6(12)	29.5(8)	-15.1(8)	-0.6(7)	-19.5(9)
C4	38.6(9)	37.2(9)	50.0(10)	18.2(8)	-2.3(8)	-0.8(7)
C15	98.0(19)	50.0(12)	43.4(11)	6.9(9)	30.3(12)	-20.1(12)
C37	55.0(13)	99.0(19)	48.7(12)	-22.5(12)	7.8(10)	-31.7(13)

Table S4: Bond Lengths in Å for **3h**.

Atom	Atom	Length/Å
S10	O11	1.4278(13)
S10	O12	1.4316(13)
S10	N1	1.6521(13)
S10	C31	1.7571(17)
Cl30	C28	1.7395(18)
O18	C16	1.3120(17)
O17	C16	1.2245(18)
N1	C2	1.429(2)
N1	C9	1.4974(17)
N14	C24	1.403(2)
N14	C15	1.452(2)
N14	C13	1.457(2)
C16	C8	1.5308(18)
C9	C13	1.542(2)
C9	C8	1.5859(19)
C7	C2	1.386(2)
C7	C6	1.390(2)
C7	C8	1.5242(19)
C8	C19	1.5961(18)
C19	C20	1.516(2)
C19	C22	1.534(2)
C19	C23	1.541(2)
C2	C3	1.389(2)
C29	C28	1.384(2)
C29	C24	1.399(2)
C20	C21	1.315(2)
C6	C5	1.391(2)
C24	C25	1.406(2)
C28	C27	1.380(3)
C36	C35	1.380(3)

C36	C31	1.390(2)
C31	C32	1.391(2)
C3	C4	1.384(3)
C25	C26	1.385(3)
C5	C4	1.376(3)
C32	C33	1.382(3)
C35	C34	1.390(3)
C27	C26	1.384(3)
C33	C34	1.388(3)
C34	C37	1.502(3)

Table S5: Bond Angles in ° for **3h**.

**Atom**	**Atom**	**Atom**	**Angle/°**
O11	S10	O12	119.95(8)
O11	S10	N1	106.50(7)
O12	S10	N1	106.08(7)
O11	S10	C31	108.74(8)
O12	S10	C31	107.73(8)
N1	S10	C31	107.19(7)
C2	N1	C9	109.90(11)
C2	N1	S10	123.23(10)
C9	N1	S10	119.55(10)
C24	N14	C15	117.49(14)
C24	N14	C13	118.69(13)
C15	N14	C13	117.00(16)
O17	C16	O18	122.64(13)
O17	C16	C8	124.53(12)
O18	C16	C8	112.80(12)
N1	C9	C13	107.67(11)
N1	C9	C8	105.18(11)
C13	C9	C8	117.69(11)
C2	C7	C6	119.96(14)
C2	C7	C8	111.60(13)
C6	C7	C8	128.41(14)
C7	C8	C16	107.50(11)
C7	C8	C9	102.44(11)
C16	C8	C9	110.68(11)
C7	C8	C19	112.07(11)
C16	C8	C19	112.57(11)
C9	C8	C19	111.08(11)
C20	C19	C22	111.39(12)
C20	C19	C23	107.94(12)
C22	C19	C23	106.66(12)
C20	C19	C8	109.56(11)

C22	C19	C8	109.04(11)
C23	C19	C8	112.25(11)
C7	C2	C3	121.11(15)
C7	C2	N1	110.42(12)
C3	C2	N1	128.37(15)
N14	C13	C9	112.33(12)
C28	C29	C24	119.91(15)
C21	C20	C19	126.83(15)
C7	C6	C5	119.12(16)
C29	C24	N14	121.48(15)
C29	C24	C25	117.60(16)
N14	C24	C25	120.87(15)
C27	C28	C29	122.94(17)
C27	C28	Cl30	119.24(14)
C29	C28	Cl30	117.82(13)
C35	C36	C31	118.97(17)
C36	C31	C32	120.73(17)
C36	C31	S10	119.77(13)
C32	C31	S10	119.44(14)
C4	C3	C2	118.00(17)
C26	C25	C24	120.62(18)
C4	C5	C6	120.00(16)
C33	C32	C31	118.91(18)
C36	C35	C34	121.61(18)
C28	C27	C26	116.93(17)
C32	C33	C34	121.63(18)
C27	C26	C25	121.86(17)
C33	C34	C35	118.13(18)
C33	C34	C37	120.6(2)
C35	C34	C37	121.3(2)
C5	C4	C3	121.71(16)

Table S6: Torsion Angles in ° for **3h**.

Atom	Atom	Atom	Atom	Angle/°
O11	S10	N1	C2	44.09(14)
O12	S10	N1	C2	172.94(12)
C31	S10	N1	C2	-72.17(13)
O11	S10	N1	C9	-168.86(11)
O12	S10	N1	C9	-40.01(13)
C31	S10	N1	C9	74.88(12)
C2	N1	C9	C13	-122.54(13)
S10	N1	C9	C13	86.40(13)
C2	N1	C9	C8	3.74(14)
S10	N1	C9	C8	-147.32(10)

C2	C7	C8	C16	123.46(13)
C6	C7	C8	C16	-54.75(19)
C2	C7	C8	C9	6.80(14)
C6	C7	C8	C9	-171.42(14)
C2	C7	C8	C19	-112.34(13)
C6	C7	C8	C19	69.44(18)
O17	C16	C8	C7	138.35(15)
O18	C16	C8	C7	-39.87(16)
O17	C16	C8	C9	-110.52(16)
O18	C16	C8	C9	71.27(15)
O17	C16	C8	C19	14.5(2)
O18	C16	C8	C19	-163.76(12)
N1	C9	C8	C7	-6.08(13)
C13	C9	C8	C7	113.76(13)
N1	C9	C8	C16	-120.44(12)
C13	C9	C8	C16	-0.60(16)
N1	C9	C8	C19	113.75(12)
C13	C9	C8	C19	-126.41(13)
C7	C8	C19	C20	168.26(12)
C16	C8	C19	C20	-70.41(15)
C9	C8	C19	C20	54.34(14)
C7	C8	C19	C22	46.12(16)
C16	C8	C19	C22	167.45(12)
C9	C8	C19	C22	-67.80(14)
C7	C8	C19	C23	-71.84(15)
C16	C8	C19	C23	49.49(16)
C9	C8	C19	C23	174.24(12)
C6	C7	C2	C3	-3.2(2)
C8	C7	C2	C3	178.45(14)
C6	C7	C2	N1	173.53(13)
C8	C7	C2	N1	-4.86(16)
C9	N1	C2	C7	0.50(16)
S10	N1	C2	C7	150.28(11)
C9	N1	C2	C3	176.88(15)
S10	N1	C2	C3	-33.3(2)
C24	N14	C13	C9	72.48(17)
C15	N14	C13	C9	-137.14(17)
N1	C9	C13	N14	-176.96(11)
C8	C9	C13	N14	64.52(16)
C22	C19	C20	C21	-3.7(2)
C23	C19	C20	C21	113.08(18)
C8	C19	C20	C21	-124.42(17)
C2	C7	C6	C5	3.5(2)
C8	C7	C6	C5	-178.41(14)

C28	C29	C24	N14	-174.25(14
C28	C29	C24	C25	3.2(2)
C15	N14	C24	C29	-144.2(2)
C13	N14	C24	C29	6.1(2)
C15	N14	C24	C25	38.4(2)
C13	N14	C24	C25	-171.35(15)
C24	C29	C28	C27	-0.6(2)
C24	C29	C28	Cl30	179.57(12)
C35	C36	C31	C32	0.6(3)
C35	C36	C31	S10	-176.38(13)
O11	S10	C31	C36	-33.42(16)
O12	S10	C31	C36	-164.87(13)
N1	S10	C31	C36	81.35(14)
O11	S10	C31	C32	149.60(14)
O12	S10	C31	C32	18.14(16)
N1	S10	C31	C32	-95.64(14)
C7	C2	C3	C4	0.6(2)
N1	C2	C3	C4	-175.41(15)
C29	C24	C25	C26	-2.8(3)
N14	C24	C25	C26	174.67(16)
C7	C6	C5	C4	-1.4(2)
C36	C31	C32	C33	-1.1(3)
S10	C31	C32	C33	175.88(14)
C31	C36	C35	C34	0.8(3)
C29	C28	C27	C26	-2.4(3)
Cl30	C28	C27	C26	177.38(14)
C31	C32	C33	C34	0.2(3)
C28	C27	C26	C25	2.8(3)
C24	C25	C26	C27	-0.2(3)
C32	C33	C34	C35	1.1(3)
C32	C33	C34	C37	-179.22(18)
C36	C35	C34	C33	-1.7(3)
C36	C35	C34	C37	178.69(18)
C6	C5	C4	C3	-1.2(3)
C2	C3	C4	C5	1.5(3)

Table S7: Hydrogen Fractional Atomic Coordinates (×104) and Equivalent Isotropic Displacement Parameters (Å2×103). *Ueq* is defined as 1/3 of the trace of the orthogonalised U_{ij}.

Atom	x	y	z	*Ueq*
H18	11028.81	4631.38	473.81	49
H9	7653.11	7647	2430.72	29
H13A	10357.18	8059.44	1995.6	36
H13B	11086.2	6673.4	1489.33	36

H29	8058.35	9727.37	2320.29	39
H20	5470.07	7762.4	1144.61	35
H6	8738.81	2862.7	1793.07	41
H22A	5953.93	5175.42	3045.58	51
H22B	4073.63	6420.22	2935.41	51
H22C	5778.43	6751.38	3136.98	51
H36	8271	5527.87	4732.42	49
H23A	5544.92	5292.34	1032.14	52
H23B	4054.24	5412.12	1745.36	52
H23C	6034	4228.37	1737.24	52
H3	11433.66	3886.1	3820.53	50
H25	6865.16	9898.96	113.61	54
H5	10247.75	1109.06	2643.29	51
H32	6398.87	9345.56	3736.14	51
H21A	3491.02	8633.6	2559.59	51
H21B	3426.09	9575.04	1773.82	51
H35	5398.97	5963.01	5369.66	55
H27	3703.51	12905.71	1628.54	55
H33	3529.43	9735.23	4367.68	58
H26	4449.35	11972.66	384.84	60
H4	11533.51	1634.88	3649.16	56
H15A	10649.19	8720.96	207.58	104
H15B	9305.59	8082.32	-97.48	104
H15C	11067.61	7089.1	284.21	104
H37A	1430.88	8336.05	4932.6	100
H37B	1933.13	8949.04	5660.49	100
H37C	2297.43	7326.25	5620.1	100

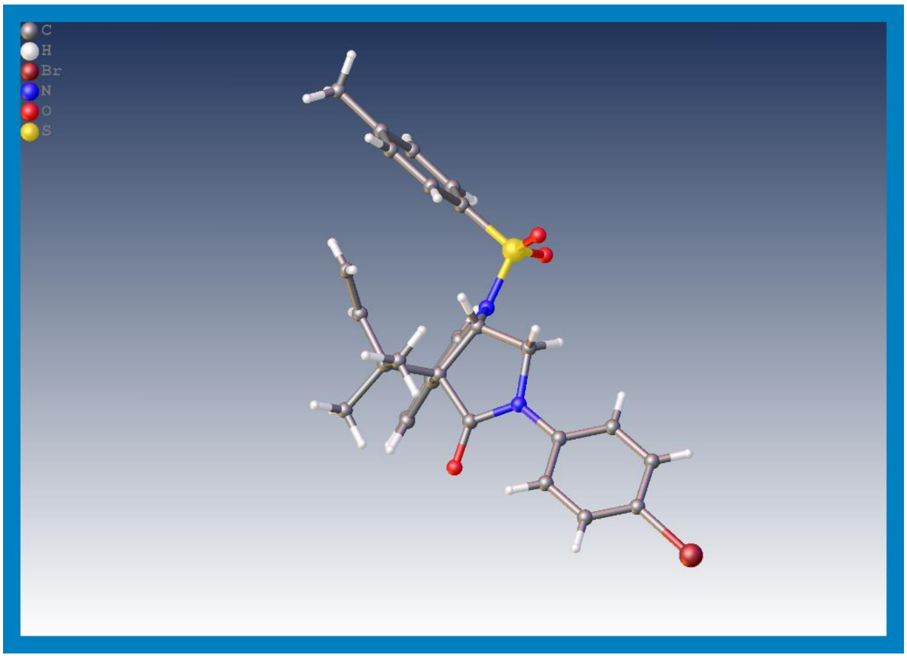

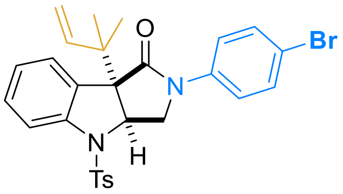

5f

Supplementary Fig. 9: X-ray crystallographic studies of compound **5f**.

Table S8. Crystal data and structure refinement for **5f**.

CCDC	2225587
Empirical formula	$C_{28}H_{25}BrN_2O_3S$
Formula weight	549.47
Temperature	251(2) K
Wavelength	1.54184Å
Crystal system	triclinic
Space group	*P*-1
Unit cell dimensions	
a = 9.08270(10)Å	α= 98.4850(10)°
b = 10.78140(10) Å	β= 105.6770(10)°
c = 14.5676(2) Å	γ = 105.9980(10)°
Volume	1281.71(3) Å
Z	2
Z’	1
Density (calculated)	1.424 g cm^{-3}
μ	3.208 mm^{-1}

Radiation type	CuKα
Min. and max. transmission	3.246 and 76.918
Measured reflections	37842
Independent reflections	5133
Reflections with I >2(I)	4637
Rint	0.0317
Restraints / parameters	0/320
Goodness-of-fit on F_2	1.071
Final R indices [I>2sigma(I)]	R_1 = 0.0353, wR_2 = 0.1019
R indices (all data)	R_1 = 0.0377, wR_2 = 0.1040
Largest diff. peak and hole	0.429 and -0.553 Å^{-3}

Table S9: Fractional Atomic Coordinates (×104) and Equivalent Isotropic Displacement Parameters (Å2×103). *Ueq* is defined as 1/3 of the trace of the orthogonalised U_{ij}.

Atom	x	y	z	*Ueq*
Br19	6621.4(4)	-2601.9(3)	5261.4(2)	99.50(15)
S26	9928.4(5)	5914.7(4)	3288.0(3)	54.13(14)
O20	4602.0(19)	596.6(13)	1703.0(10)	74.9(4)
O28	9650.6(19)	6078.6(14)	4211.8(9)	72.9(4)
N10	6322.5(16)	1879.9(13)	3214.4(10)	46.2(3)
O27	11421.8(16)	5794.8(14)	3224.6(12)	70.1(4)
N9	8469.2(18)	4561.5(14)	2606.6(10)	53.2(4)
C6	6707.5(19)	2983.5(16)	1229.4(12)	45.7(3)
C1	8244(2)	3938.1(15)	1632.0(12)	45.8(3)
C8	7019.4(19)	4015.2(16)	2884.9(11)	44.8(3)
C13	6357(2)	856.2(16)	3726.8(12)	46.2(3)
C29	9624(2)	7235.9(16)	2770.4(12)	48.7(4)
C7	5769.2(19)	2990.1(16)	1943.6(11)	46.1(4)
C11	5457(2)	1670.8(17)	2248.1(12)	50.0(4)
C2	9336(2)	4139.2(18)	1122.5(14)	55.8(4)
C14	7760(2)	968.7(18)	4459.8(13)	54.6(4)
C12	7333(2)	3256.0(18)	3679.4(13)	60.3(5)
C30	10620(2)	7818.3(18)	2271.4(14)	54.8(4)
C18	4997(2)	-262.2(19)	3501.9(15)	57.7(4)
C17	5069(2)	-1282(2)	3969.2(16)	62.7(5)
C5	6295(2)	2155.6(18)	306.4(13)	55.2(4)
C34	8400(3)	7707(2)	2885.5(17)	65.5(5)
C15	7827(3)	-48(2)	4932.7(14)	62.0(5)
C16	6489(3)	-1169.9(19)	4664.8(15)	59.3(4)
C3	8864(3)	3334(2)	186.2(15)	60.7(5)
C4	7381(3)	2348(2)	-209.4(14)	61.3(5)
C33	8175(3)	8753(2)	2496.6(19)	71.3(6)
C21	4165(2)	3348(2)	1588.7(14)	59.4(5)

C31	10389(3)	8882(2)	1900.9(16)	62.7(5)
C32	9173(2)	9369.6(19)	2009.7(16)	62.3(5)
C24	3338(3)	3256(3)	2374.9(18)	75.2(6)
C25	2967(3)	2380(3)	619.9(17)	84.6(7)
C22	4609(3)	4719(3)	1413.2(19)	76.7(6)
C35	8935(4)	10543(3)	1619(3)	94.0(8)
C23	4275(4)	5742(3)	1817(3)	105.1(9)

Table S10: Anisotropic Displacement Parameters (×104). The anisotropic displacement factor exponent takes the form: -2π^2[h2a*2 × U11+ ... +2hka* × b* × U12]

Atom	*U11*	*U22*	*U33*	*U23*	*U13*	*U12*
Br19	132.5(3)	84.8(2)	108.2(3)	63.61(18)	53.5(2)	42.78(19)
S26	53.1(2)	42.3(2)	45.5(2)	8.35(17)	2.03(17)	-2.36(17)
O20	88.8(10)	46.1(7)	50.0(7)	4.8(6)	-1.1(7)	-12.0(7)
O28	87.8(10)	56.4(8)	40.1(6)	5.7(6)	4.7(6)	-10.2(7)
N10	47.1(7)	38.6(7)	40.6(7)	8.5(5)	7.0(5)	2.9(5)
O27	47.7(7)	56.4(8)	87.5(10)	21.8(7)	2.0(6)	6.3(6)
N9	53.1(8)	45.1(8)	42.9(7)	3.5(6)	11.7(6)	-4.5(6)
C6	48.8(8)	40.9(8)	40.2(8)	9.9(6)	7.5(6)	10.4(6)
C1	50.3(8)	37.5(8)	42.3(8)	10.5(6)	9.2(6)	8.8(6)
C8	47.3(8)	39.4(8)	39.0(8)	7.6(6)	8.1(6)	7.8(6)
C13	50.2(8)	42.6(8)	42.4(8)	10.4(6)	15.9(7)	9.4(7)
C29	49.8(8)	39.4(8)	45.2(8)	4.0(6)	11.6(7)	4.2(6)
C7	43.6(8)	43.1(8)	39.8(8)	8.0(6)	4.7(6)	6.0(6)
C11	47.6(8)	45.8(9)	43.3(8)	8.9(7)	7.2(7)	3.5(7)
C2	55.2(9)	48.9(9)	58.9(10)	15.6(8)	18.6(8)	8.9(7)
C14	55.1(9)	49.5(9)	47.8(9)	12.2(7)	9.7(7)	6.5(7)
C12	68.5(11)	43.4(9)	43.5(9)	10.4(7)	2.0(8)	-5.0(8)
C30	50.4(9)	52.5(10)	63.8(11)	17.3(8)	22.5(8)	15.3(7)
C18	49.1(9)	53.9(10)	63.2(11)	18.3(8)	16.7(8)	5.6(7)
C17	64.2(11)	52.0(10)	69.9(12)	21.5(9)	29.1(9)	5.8(8)
C5	59.3(10)	46.6(9)	45.4(9)	5.0(7)	7.1(7)	8.6(7)
C34	69.8(12)	53.2(10)	77.8(13)	10.2(9)	39.7(10)	14.6(9)
C15	67.4(11)	64.5(11)	52.1(10)	20.8(9)	14.6(8)	20.3(9)
C16	78.2(12)	53.9(10)	57.6(10)	24.6(8)	34.0(9)	22.4(9)
C3	72.4(12)	58.6(11)	58.9(11)	18.5(9)	30.7(9)	22.3(9)
C4	79.0(12)	55.9(11)	46.3(9)	7.4(8)	19.7(9)	21.4(9)
C33	67.6(12)	55.9(11)	95.8(16)	9.7(11)	35.5(11)	24.5(9)
C21	48.2(9)	70.7(12)	53.3(10)	16.6(9)	6.5(8)	19.6(8)
C31	65.2(11)	59.0(11)	71.9(12)	27.1(9)	29.9(10)	19.6(9)
C32	62.9(11)	48.8(10)	67.9(12)	11.8(9)	13.0(9)	16.4(8)
C24	61.2(12)	93.5(16)	75.5(14)	24.0(12)	24.0(10)	29.2(11)
C25	54.4(11)	115(2)	61.9(12)	7.6(13)	-5.1(10)	23.5(12)

C22	75.0(13)	86.0(16)	81.5(15)	40.2(13)	21.3(11)	39.5(12)
C35	96.0(18)	69.1(15)	124(2)	40.2(15)	26.9(16)	37.3(14)
C23	90.5(18)	95(2)	135(3)	40.9(19)	26.7(18)	41.8(16)

Table S11: Bond Lengths in Å for **5f**.

Atom	Atom	Length/Å
Br19	C16	1.8962(19)
S26	O27	1.4234(16)
S26	O28	1.4296(15)
S26	N9	1.6329(13)
S26	C29	1.7592(18)
O20	C11	1.214(2)
N10	C11	1.364(2)
N10	C13	1.423(2)
N10	C12	1.456(2)
N9	C1	1.412(2)
N9	C8	1.475(2)
C6	C5	1.391(2)
C6	C1	1.392(2)
C6	C7	1.513(2)
C1	C2	1.381(3)
C8	C12	1.523(2)
C8	C7	1.553(2)
C13	C14	1.386(2)
C13	C18	1.389(2)
C29	C34	1.382(3)
C29	C30	1.385(3)
C7	C11	1.527(2)
C7	C21	1.581(2)
C2	C3	1.388(3)
C14	C15	1.384(3)
C30	C31	1.380(3)
C18	C17	1.384(3)
C17	C16	1.370(3)
C5	C4	1.383(3)
C34	C33	1.374(3)
C15	C16	1.374(3)
C3	C4	1.374(3)
C33	C32	1.386(3)
C21	C22	1.501(3)
C21	C24	1.531(3)
C21	C25	1.541(3)
C31	C32	1.382(3)
C32	C35	1.506(3)

C22	C23	1.320(4)

Table 12: Bond Angles in ° for **5f**.

Atom	Atom	Atom	Angle/°
O27	S26	O28	120.61(10)
O27	S26	N9	108.24(9)
O28	S26	N9	104.04(8)
O27	S26	C29	107.59(9)
O28	S26	C29	108.43(9)
N9	S26	C29	107.26(8)
C11	N10	C13	124.42(13)
C11	N10	C12	113.99(14)
C13	N10	C12	121.43(13)
C1	N9	C8	111.19(12)
C1	N9	S26	126.31(12)
C8	N9	S26	121.27(11)
C5	C6	C1	118.78(16)
C5	C6	C7	130.30(15)
C1	C6	C7	110.82(14)
C2	C1	C6	122.16(15)
C2	C1	N9	128.42(15)
C6	C1	N9	109.35(14)
N9	C8	C12	112.37(15)
N9	C8	C7	104.89(13)
C12	C8	C7	107.01(13)
C14	C13	C18	119.23(16)
C14	C13	N10	119.92(14)
C18	C13	N10	120.85(15)
C34	C29	C30	120.23(18)
C34	C29	S26	119.21(15)
C30	C29	S26	120.55(14)
C6	C7	C11	109.18(14)
C6	C7	C8	103.52(13)
C11	C7	C8	103.91(13)
C6	C7	C21	115.55(14)
C11	C7	C21	111.99(14)
C8	C7	C21	111.75(14)
O20	C11	N10	125.38(17)
O20	C11	C7	124.93(15)
N10	C11	C7	109.66(13)
C1	C2	C3	117.68(17)
C13	C14	C15	120.34(17)
N10	C12	C8	105.39(13)
C31	C30	C29	119.35(17)

C17	C18	C13	120.34(18)
C16	C17	C18	119.27(17)
C4	C5	C6	119.41(17)
C33	C34	C29	119.43(19)
C16	C15	C14	119.23(18)
C17	C16	C15	121.47(18)
C17	C16	Br19	119.35(15)
C15	C16	Br19	119.18(16)
C4	C3	C2	121.15(18)
C3	C4	C5	120.69(17)
C34	C33	C32	121.49(19)
C22	C21	C24	111.8(2)
C22	C21	C25	107.98(19)
C24	C21	C25	108.17(18)
C22	C21	C7	108.36(16)
C24	C21	C7	108.75(16)
C25	C21	C7	111.76(17)
C30	C31	C32	121.31(19)
C31	C32	C33	118.17(19)
C31	C32	C35	121.4(2)
C33	C32	C35	120.5(2)
C23	C22	C21	126.8(3)

Table S13: Torsion Angles in ° for **5f**.

Atom	Atom	Atom	Atom	Angle/°
O27	S26	N9	C1	-47.66(18)
O28	S26	N9	C1	-177.08(16)
C29	S26	N9	C1	68.16(17)
O27	S26	N9	C8	146.09(14)
O28	S26	N9	C8	16.68(16)
C29	S26	N9	C8	-98.09(14)
C5	C6	C1	C2	3.8(3)
C7	C6	C1	C2	-179.46(16
C5	C6	C1	N9	-173.52(15)
C7	C6	C1	N9	3.26(19)
C8	N9	C1	C2	-177.34(17)
S26	N9	C1	C2	15.2(3)
C8	N9	C1	C6	-0.28(19)
S26	N9	C1	C6	-167.69(13)
C1	N9	C8	C12	113.25(16)
S26	N9	C8	C12	-78.61(17)
C1	N9	C8	C7	-2.64(18)
S26	N9	C8	C7	165.50(12)
C11	N10	C13	C14	146.09(18)

C12	N10	C13	C14	-29.2(2)
C11	N10	C13	C18	-33.8(3)
C12	N10	C13	C18	150.87(18)
O27	S26	C29	C34	-169.29(15)
O28	S26	C29	C34	-37.33(17)
N9	S26	C29	C34	74.46(16)
O27	S26	C29	C30	9.32(17)
O28	S26	C29	C30	141.28(15)
N9	S26	C29	C30	-106.92(15)
C5	C6	C7	C11	61.4(2)
C1	C6	C7	C11	-114.91(15)
C5	C6	C7	C8	171.61(17)
C1	C6	C7	C8	-4.70(18)
C5	C6	C7	C21	-65.9(2)
C1	C6	C7	C21	117.82(16)
N9	C8	C7	C6	4.25(17)
C12	C8	C7	C6	-115.30(16)
N9	C8	C7	C11	118.31(14)
C12	C8	C7	C11	-1.23(18)
N9	C8	C7	C21	-120.76(15)
C12	C8	C7	C21	119.70(17)
C13	N10	C11	O20	0.4(3)
C12	N10	C11	O20	176.0(2)
C13	N10	C11	C7	-177.69(14)
C12	N10	C11	C7	-2.1(2)
C6	C7	C11	O20	-66.2(2)
C8	C7	C11	O20	-176.12(19)
C21	C7	C11	O20	63.1(2)
C6	C7	C11	N10	111.95(15)
C8	C7	C11	N10	2.00(18)
C21	C7	C11	N10	-118.78(16)
C6	C1	C2	C3	-1.1(3)
N9	C1	C2	C3	175.60(18)
C18	C13	C14	C15	2.8(3)
N10	C13	C14	C15	-177.16(17)
C11	N10	C12	C8	1.2(2)
C13	N10	C12	C8	176.96(15)
N9	C8	C12	N10	-114.47(16)
C7	C8	C12	N10	0.1(2)
C34	C29	C30	C31	0.8(3)
S26	C29	C30	C31	-177.85(15)
C14	C13	C18	C17	-3.3(3)
N10	C13	C18	C17	176.64(17)
C13	C18	C17	C16	0.8(3)

C1	C6	C5	C4	-3.6(3)
C7	C6	C5	C4	-179.63(18)
C30	C29	C34	C33	0.3(3)
S26	C29	C34	C33	178.92(16)
C13	C14	C15	C16	0.2(3)
C18	C17	C16	C15	2.2(3)
C18	C17	C16	Br19	-177.04(15)
C14	C15	C16	C17	-2.7(3)
C14	C15	C16	Br19	176.52(15)
C1	C2	C3	C4	-1.7(3)
C2	C3	C4	C5	1.8(3)
C6	C5	C4	C3	0.9(3)
C29	C34	C33	C32	-1.5(3)
C6	C7	C21	C22	-60.4(2)
C11	C7	C21	C22	173.75(16)
C8	C7	C21	C22	57.6(2)
C6	C7	C21	C24	177.82(16)
C11	C7	C21	C24	52.0(2)
C8	C7	C21	C24	-64.2(2)
C6	C7	C21	C25	58.5(2)
C11	C7	C21	C25	-67.4(2)
C8	C7	C21	C25	176.49(18)
C29	C30	C31	C32	-0.6(3)
C30	C31	C32	C33	-0.5(3)
C30	C31	C32	C35	179.1(2)
C34	C33	C32	C31	1.6(3)
C34	C33	C32	C35	-178.0(2)
C24	C21	C22	C23	-4.0(3)
C25	C21	C22	C23	114.9(3)
C7	C21	C22	C23	-123.9(3)

Table S14: Hydrogen Fractional Atomic Coordinates (×104) and Equivalent Isotropic Displacement Parameters (Å2×103). Ueq is defined as 1/3 of the trace of the orthogonalised U_{ij}.

**Atom**	**x**	**y**	**z**	***Ueq***
H8	6597.89	4726.39	3090.2	54
H2	10361.67	4798.34	1399.94	67
H14	8670.32	1739.23	4636.86	66
H12A	8480.05	3331.75	3912.49	72
H12B	7034.67	3601.12	4238.25	72
H30	11445.24	7492.25	2185.77	66
H18	4023.42	-326.05	3029.8	69
H17	4153.52	-2044.53	3810.95	75
H5	5288.25	1471.45	35.67	66

H34	7727.17	7314.69	3226.83	79
H15	8777.33	29.31	5431.31	74
H3	9572.47	3465.61	-183.6	73
H4	7100.15	1797.97	-837.24	74
H33	7325.02	9057.87	2562.32	86
H31	11071.94	9283.28	1567.96	75
H24A	3102.5	2366.69	2483.85	113
H24B	2335.57	3449.76	2156.68	113
H24C	4050.77	3894.38	2982.37	113
H25A	3404.18	2516.04	90.43	127
H25B	1939.44	2542.92	476.95	127
H25C	2802.64	1472.76	682.28	127
H22	5185.69	4864.35	969.72	92
H35A	7785.41	10405.12	1355.78	141
H35B	9416.52	10637.53	1103.67	141
H35C	9450.56	11343.26	2146.69	141
H23A	3701.47	5648.79	2265.16	126
H23B	4611.84	6562.7	1655.1	126

6. NMR spectrum

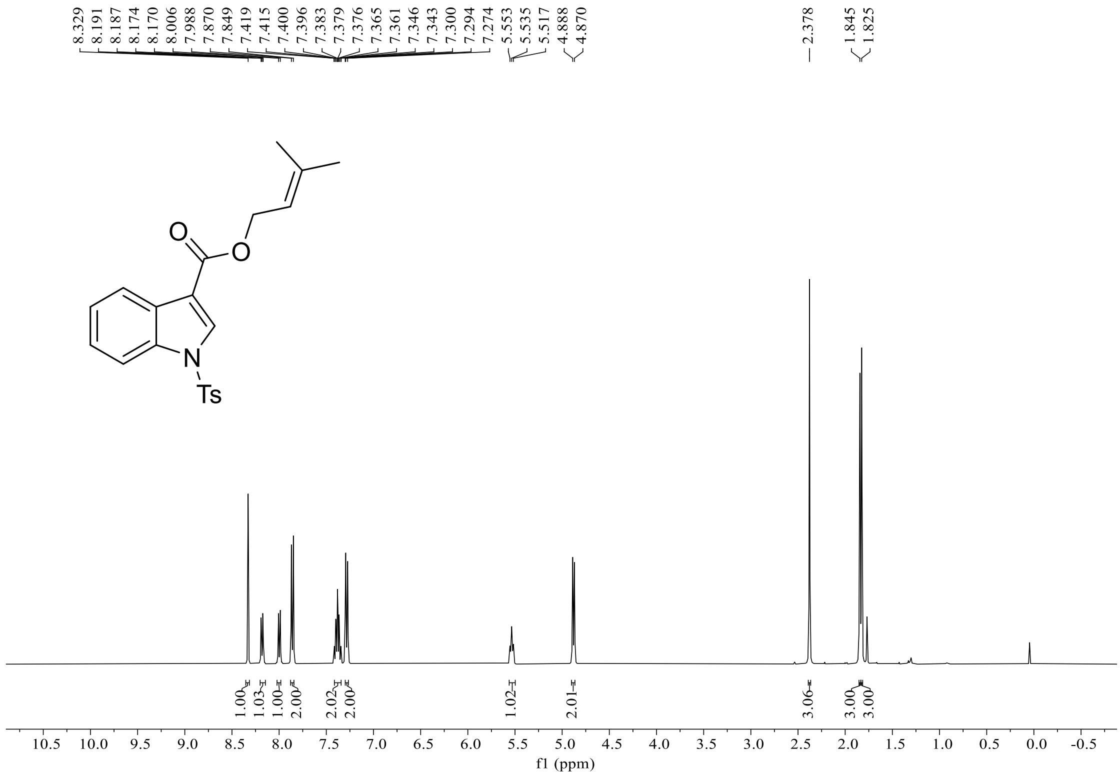

Supplementary Fig. 10. ^{1}H NMR of compound **1d** (400 MHZ, $CDCl_3$)

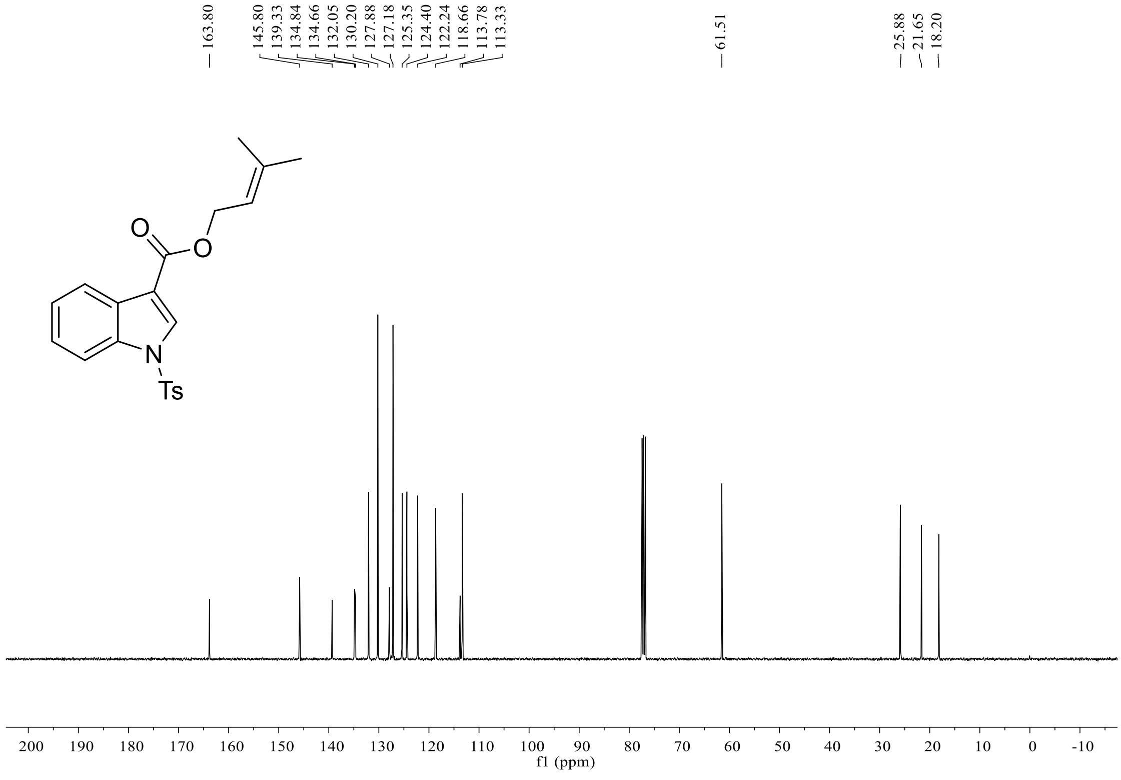

Supplementary Fig. 11. ^{13}C NMR of compound **1d** (100 MHZ, $CDCl_3$)

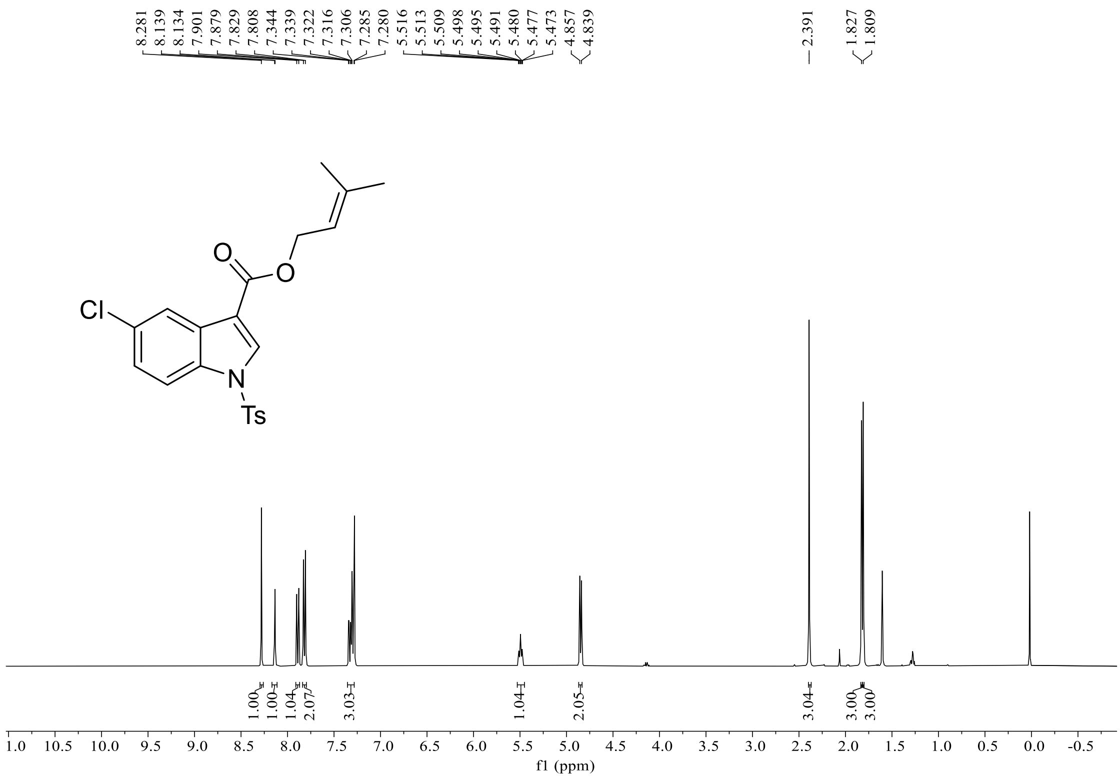

Supplementary Fig. 12. ^{1}H NMR of compound **1x** (400 MHZ, $CDCl_3$)

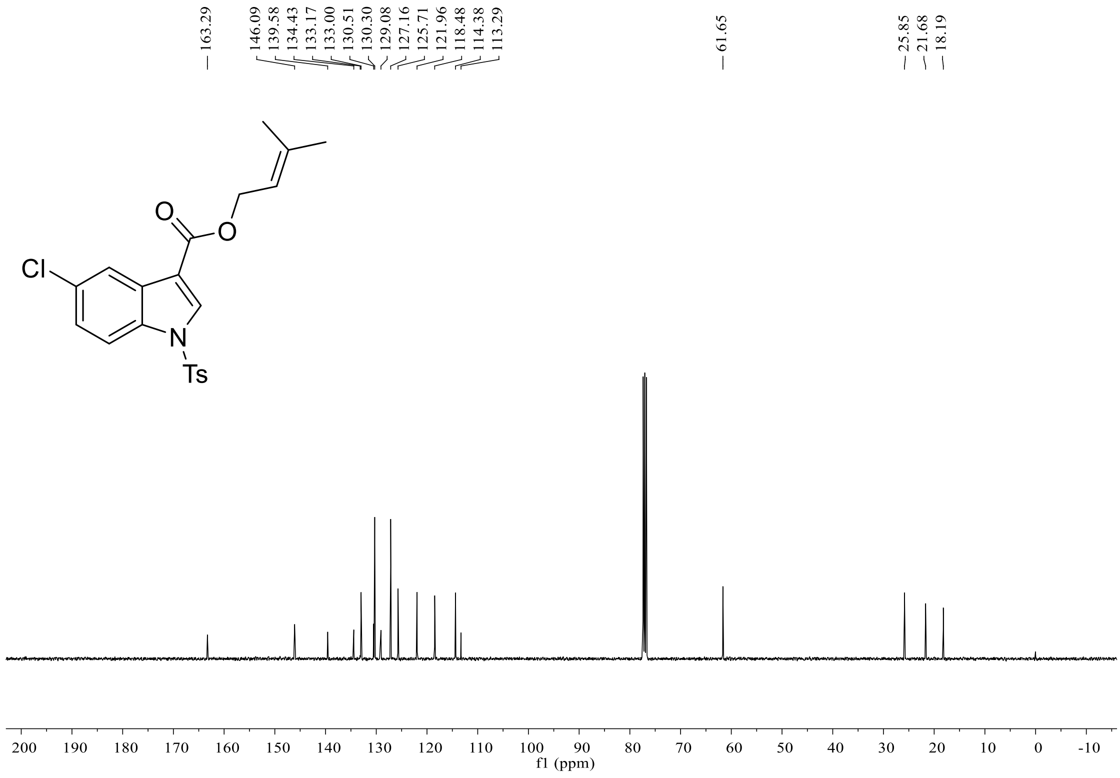

Supplementary Fig. 13. ^{13}C NMR of compound **1x** (100 MHZ, $CDCl_3$)

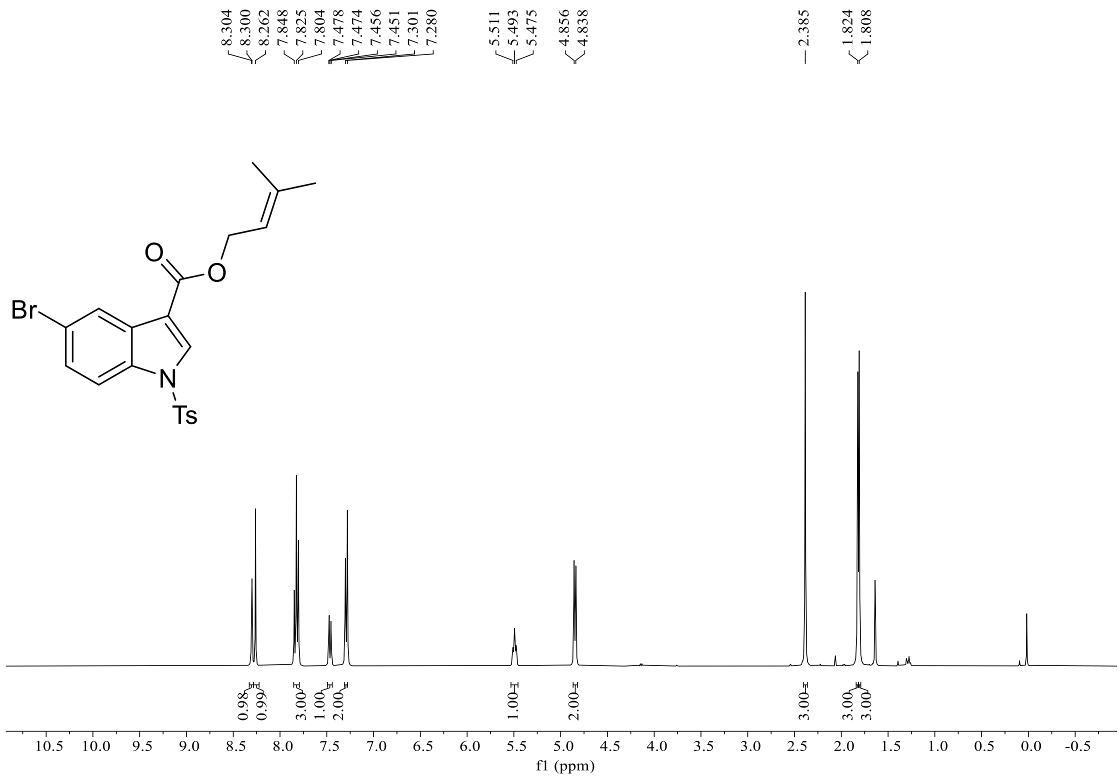

Supplementary Fig. 14. ^{1}H NMR of compound **1y** (400 MHZ, $CDCl_3$)

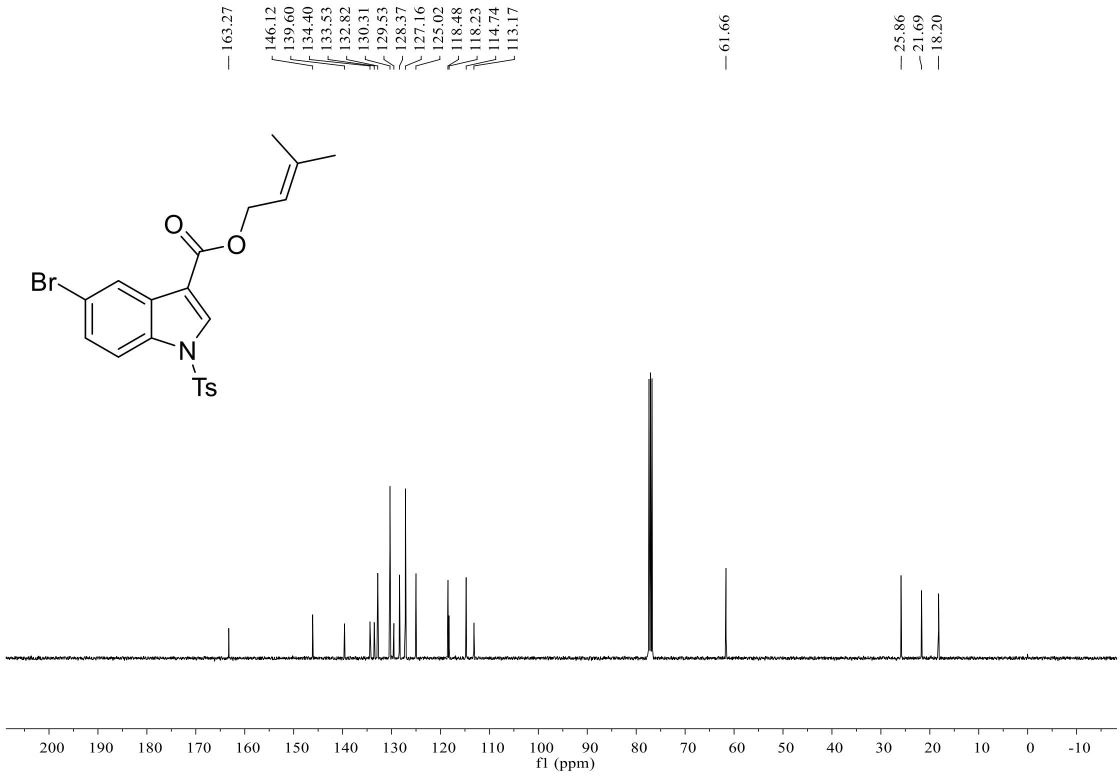

Supplementary Fig. 15. ^{13}C NMR of compound **1y** (100 MHZ, $CDCl_3$)

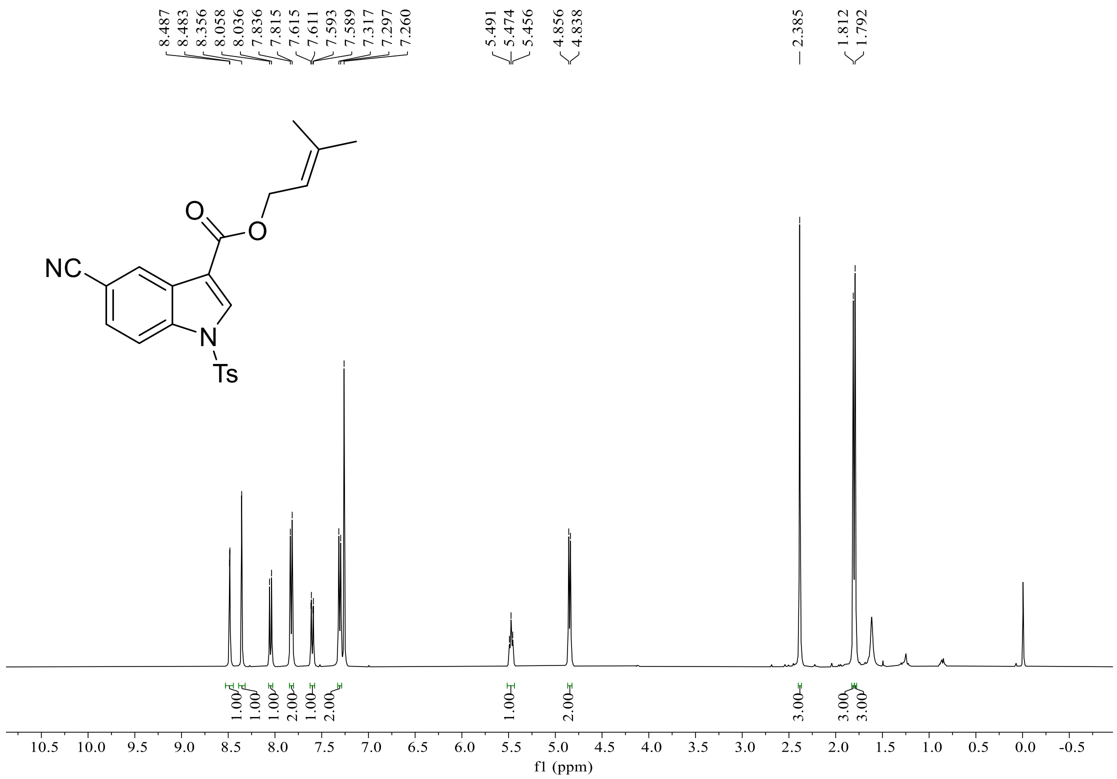

Supplementary Fig. 16. ^{1}H NMR of compound **1z** (400 MHZ, $CDCl_3$)

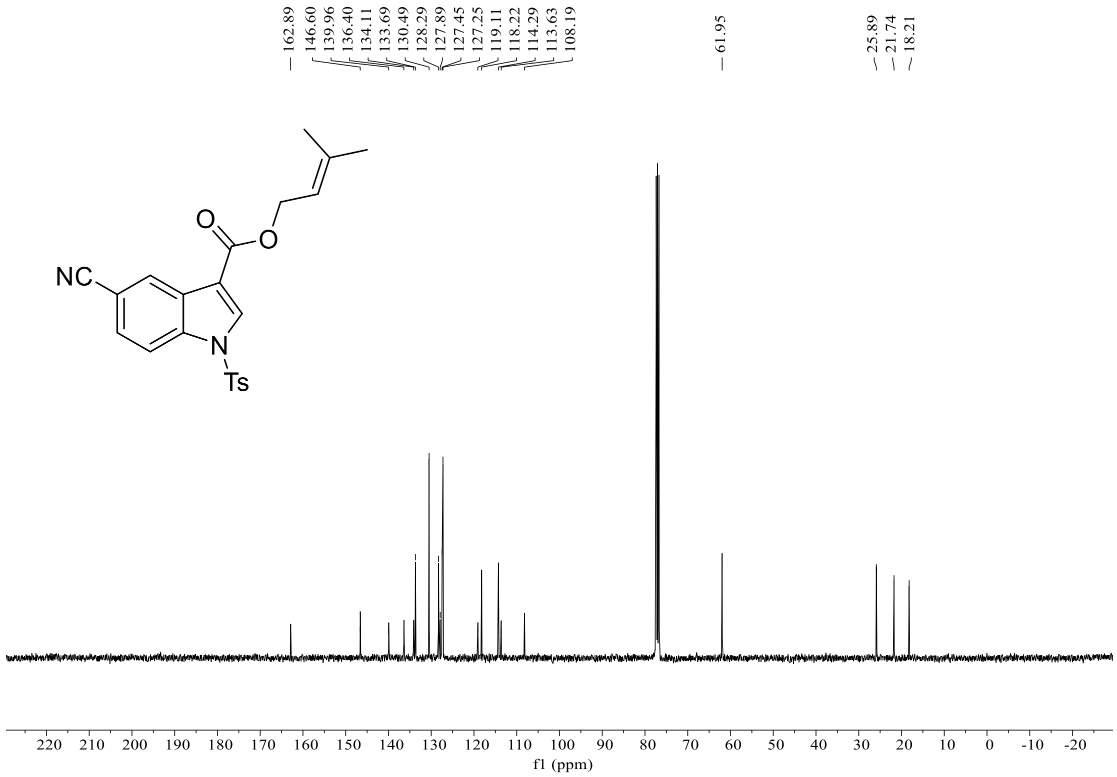

Supplementary Fig. 17. ^{13}C NMR of compound **1z** (100 MHZ, $CDCl_3$)

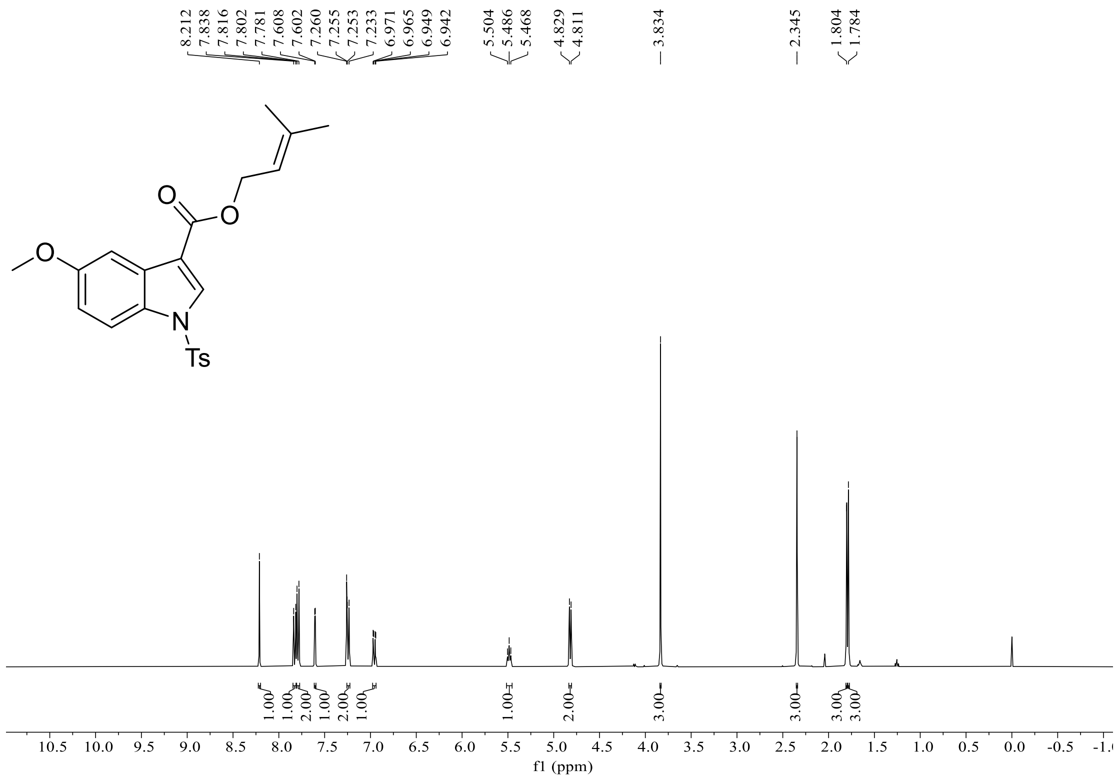

Supplementary Fig. 18. ^{1}H NMR of compound **1aa** (400 MHZ, $CDCl_3$)

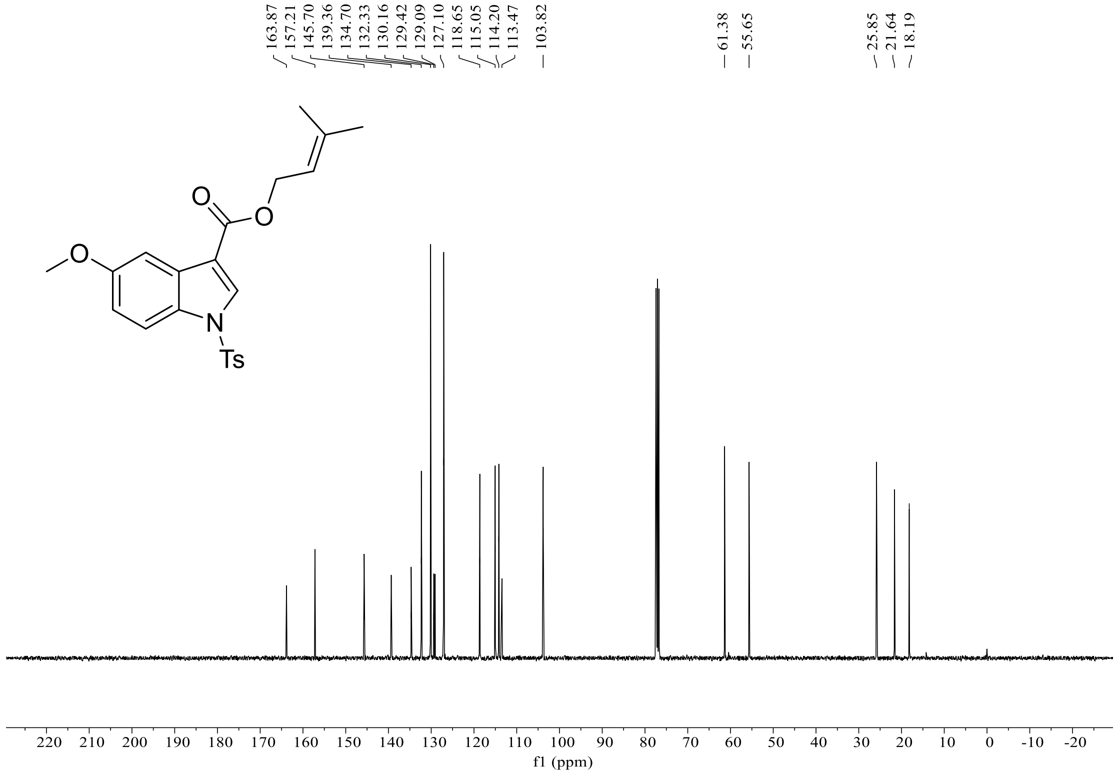

Supplementary Fig. 19. ^{13}C NMR of compound **1aa** (100 MHZ, $CDCl_3$)

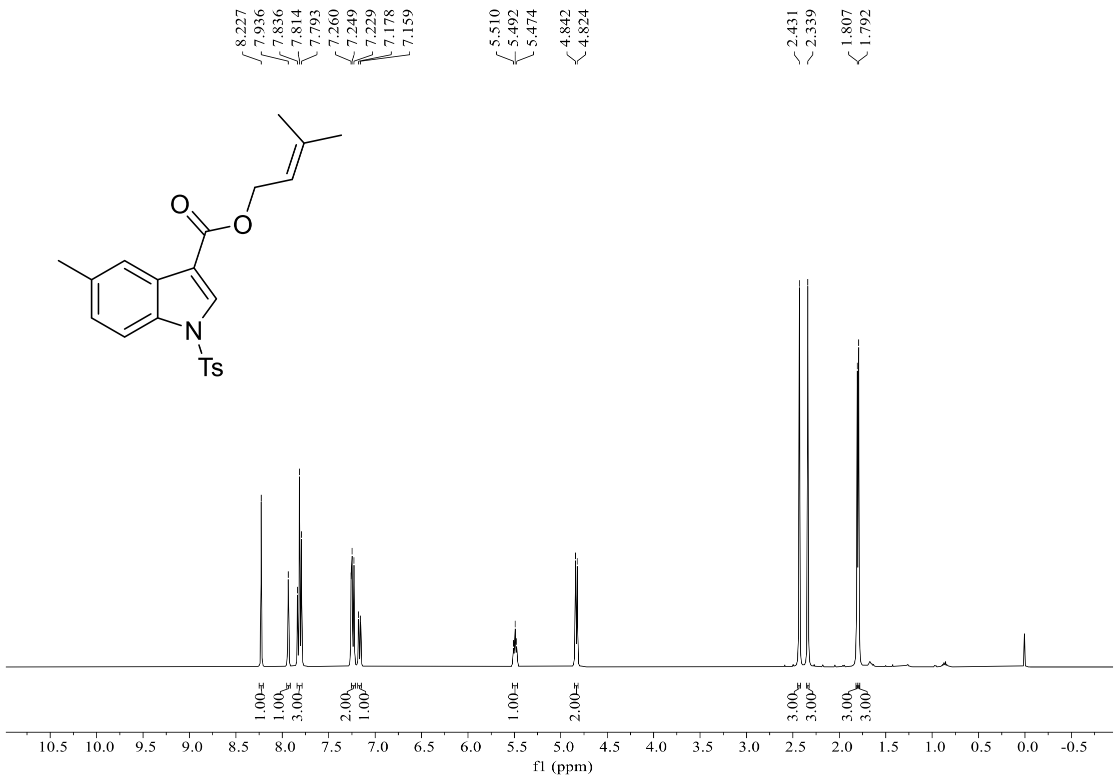

Supplementary Fig. 20. ^{1}H NMR of compound **1ab** (400 MHZ, $CDCl_3$)

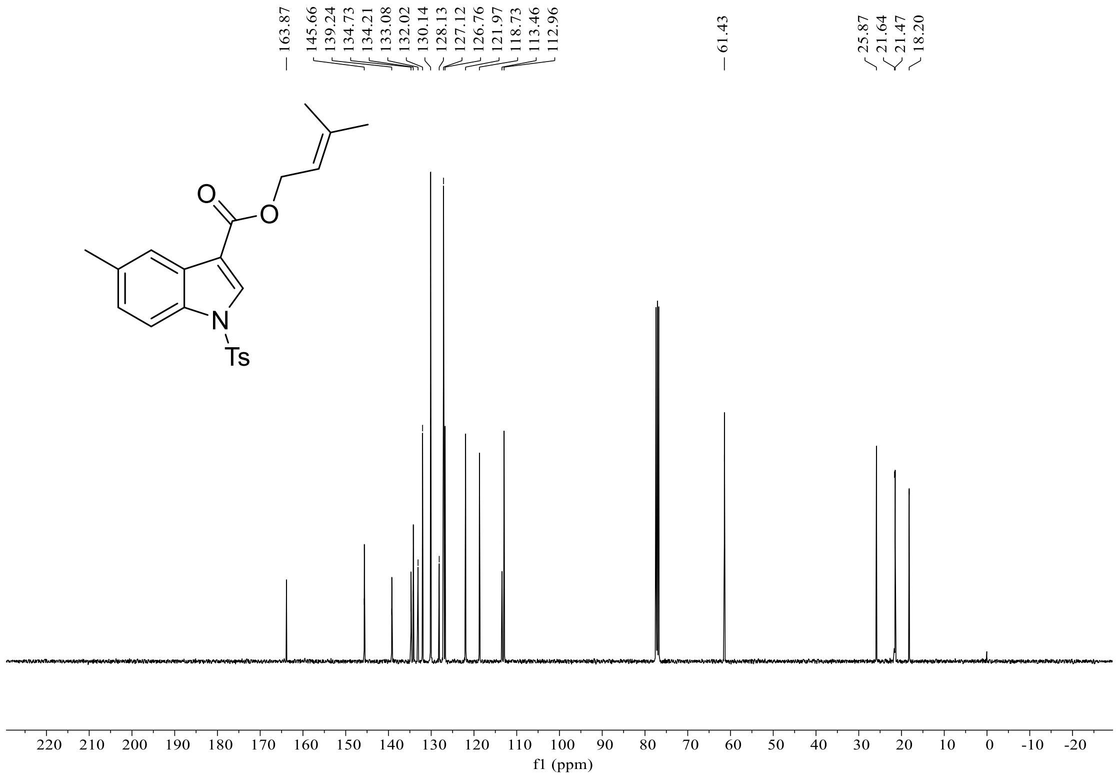

Supplementary Fig. 21. ^{13}C NMR of compound **1ab** (100 MHZ, $CDCl_3$)

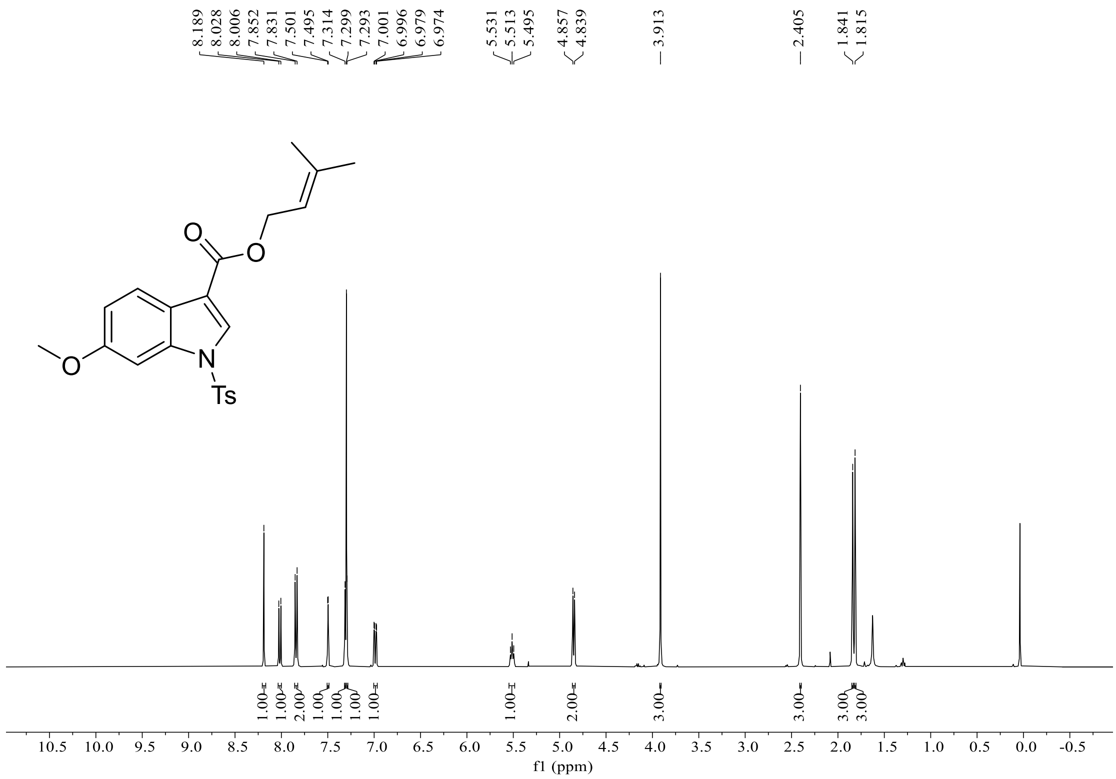

Supplementary Fig. 22. ^{1}H NMR of compound **1ac** (400 MHZ, $CDCl_3$)

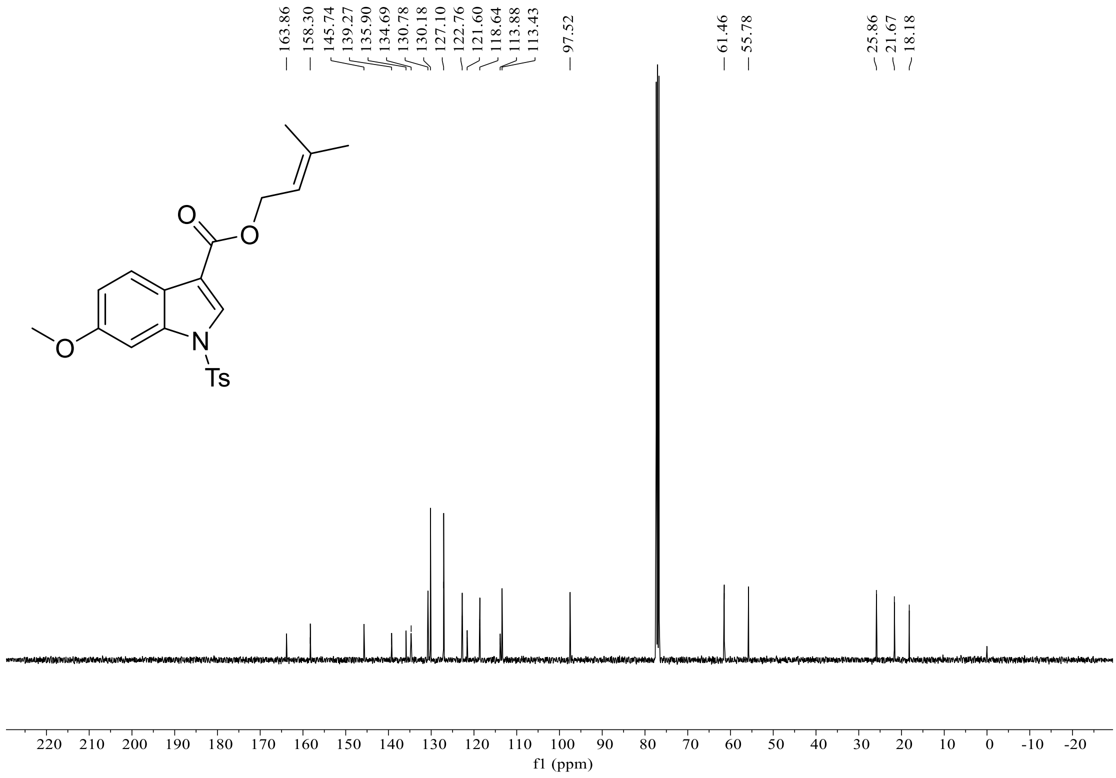

Supplementary Fig. 23. ^{13}C NMR of compound **1ac** (100 MHZ, $CDCl_3$)

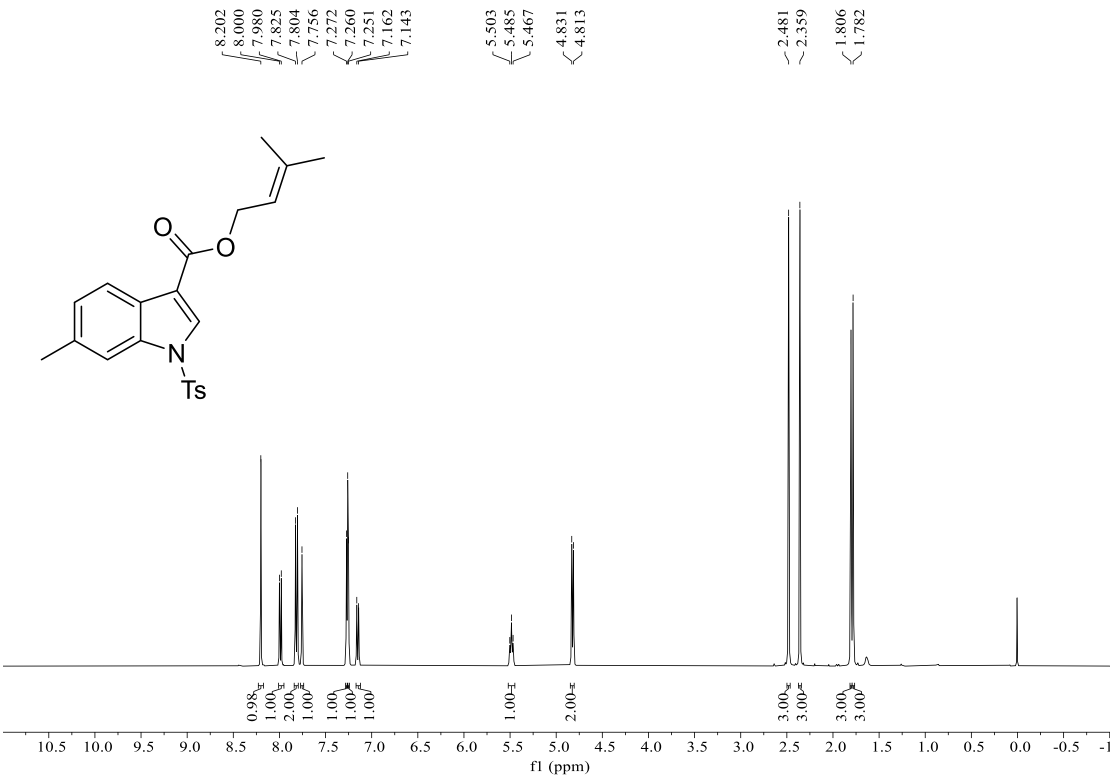

Supplementary Fig. 24. ^{1}H NMR of compound **1ad** (400 MHZ, $CDCl_3$)

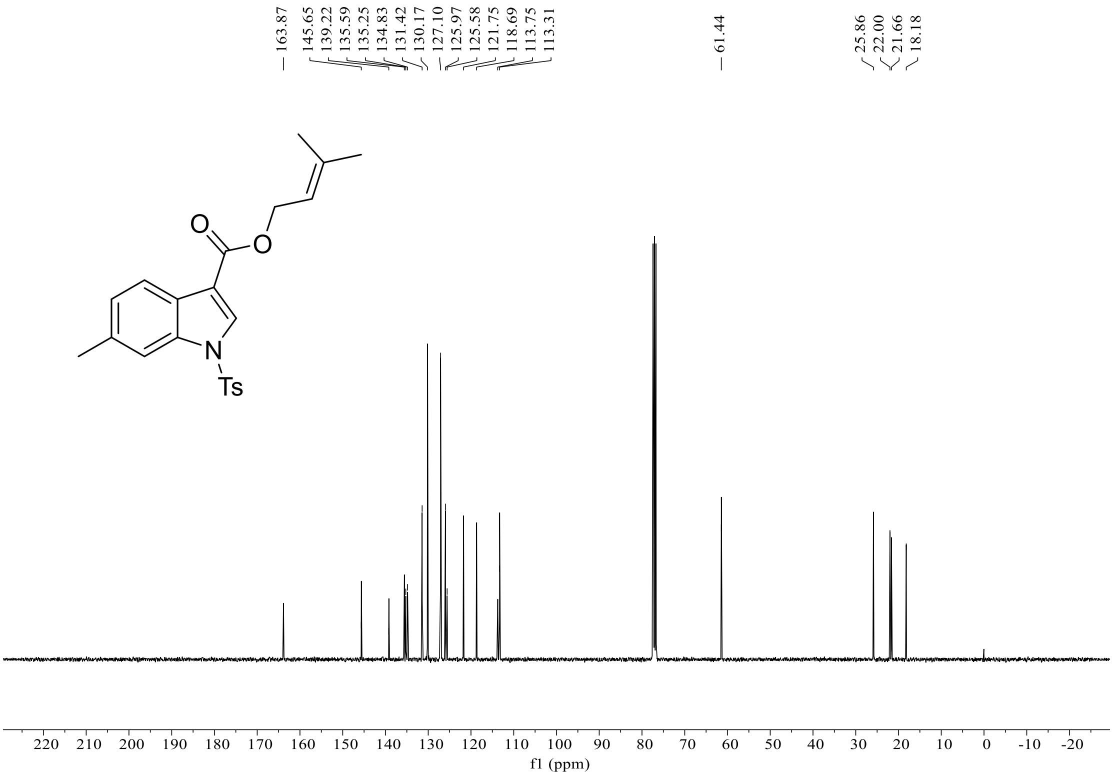

Supplementary Fig. 25. ^{13}C NMR of compound **1ad** (100 MHZ, $CDCl_3$)

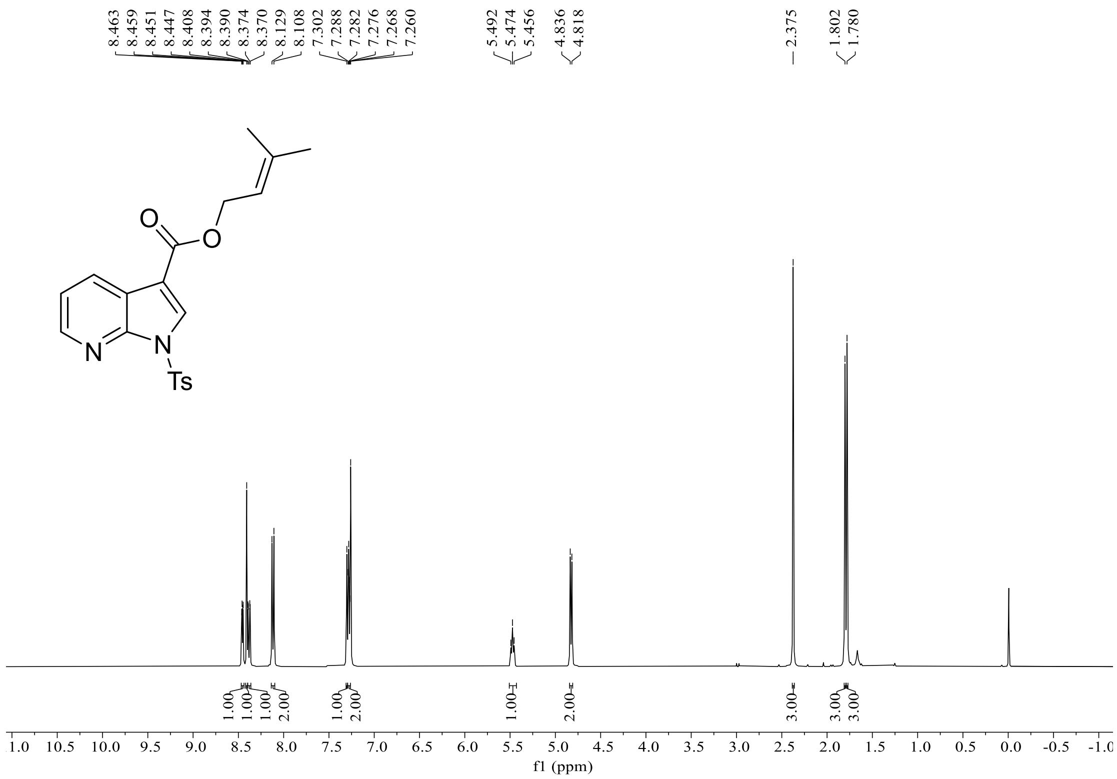

Supplementary Fig. 26. ^{1}H NMR of compound **1ae** (400 MHZ, $CDCl_3$)

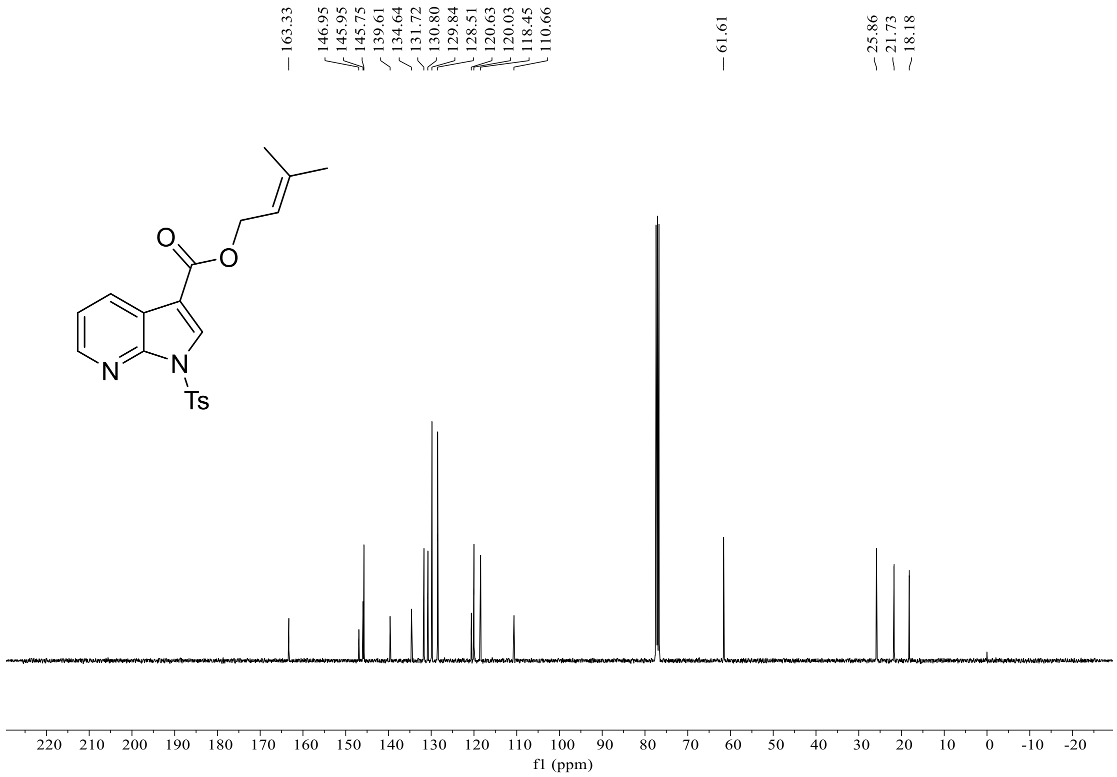

Supplementary Fig. 27. ^{13}C NMR of compound **1ae** (100 MHZ, $CDCl_3$)

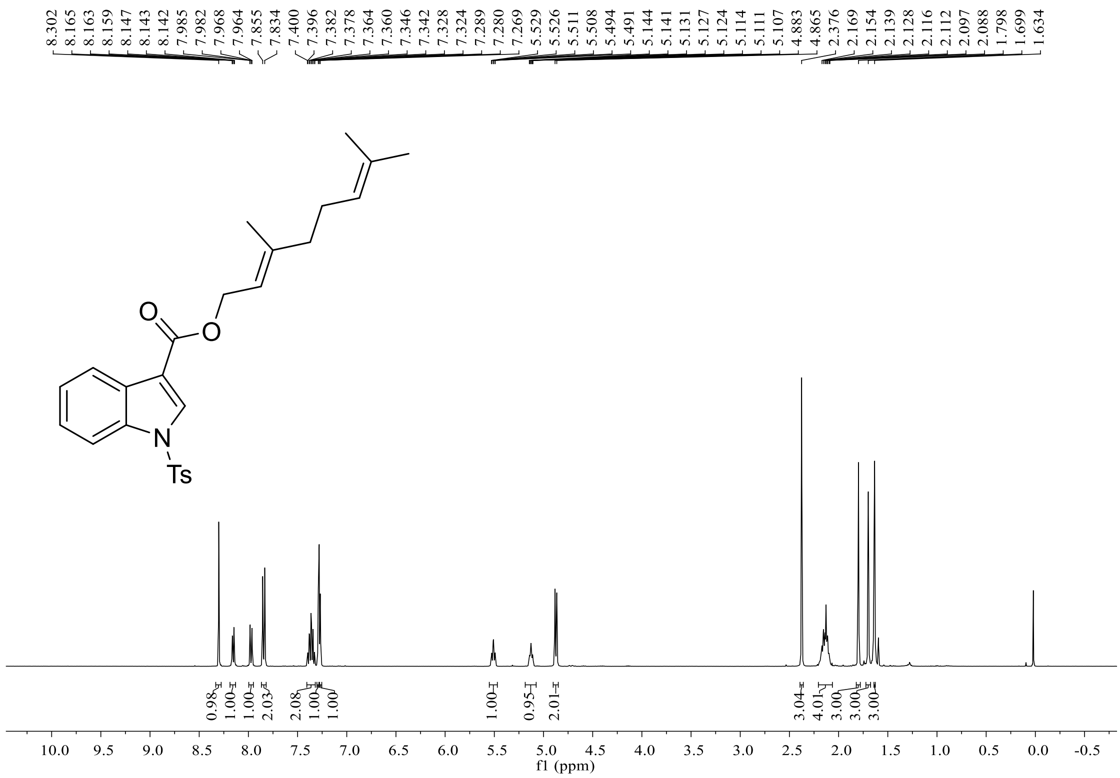

Supplementary Fig. 28. ^{1}H NMR of compound **8a** (400 MHZ, $CDCl_3$)

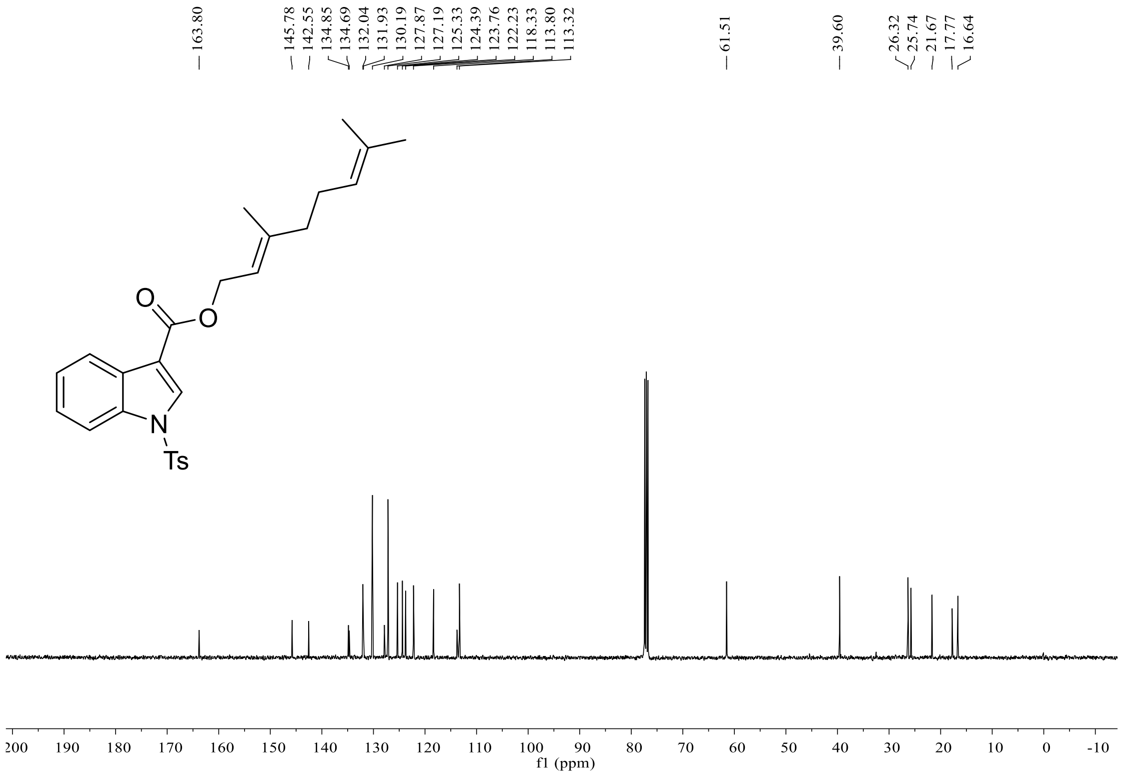

Supplementary Fig. 29. ^{13}C NMR of compound **8a** (100 MHZ, $CDCl_3$)

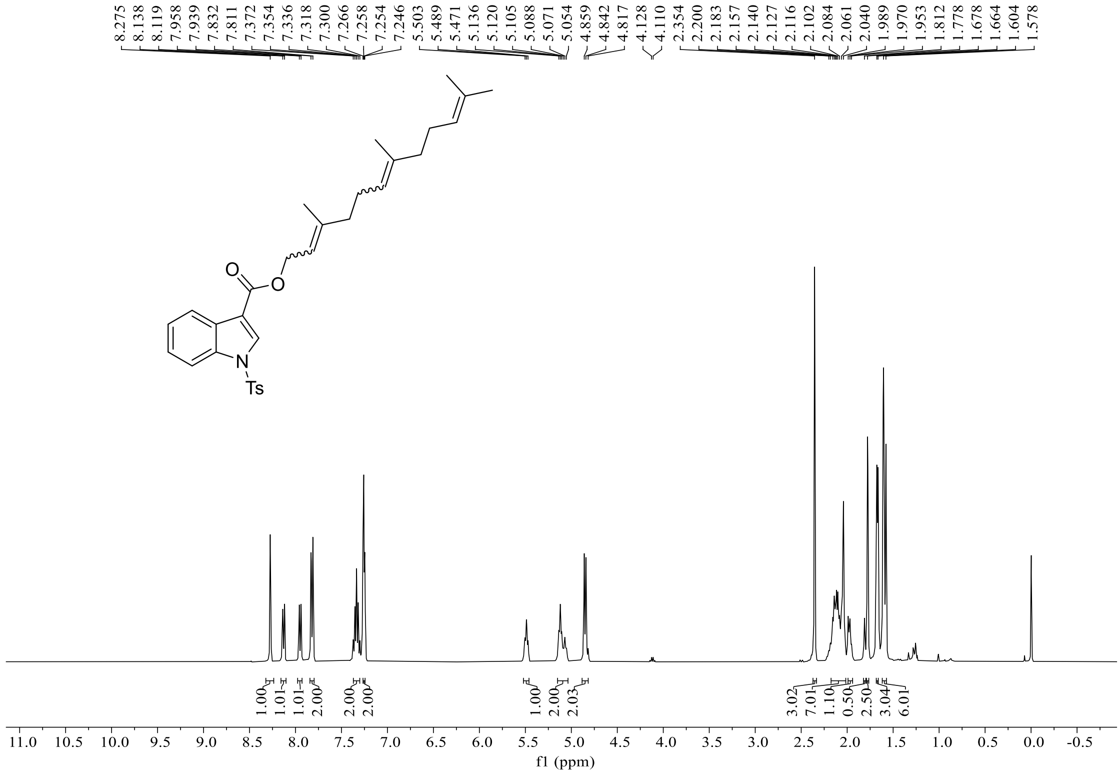

Supplementary Fig. 30. ^{1}H NMR of compound **8b** (400 MHZ, $CDCl_3$)

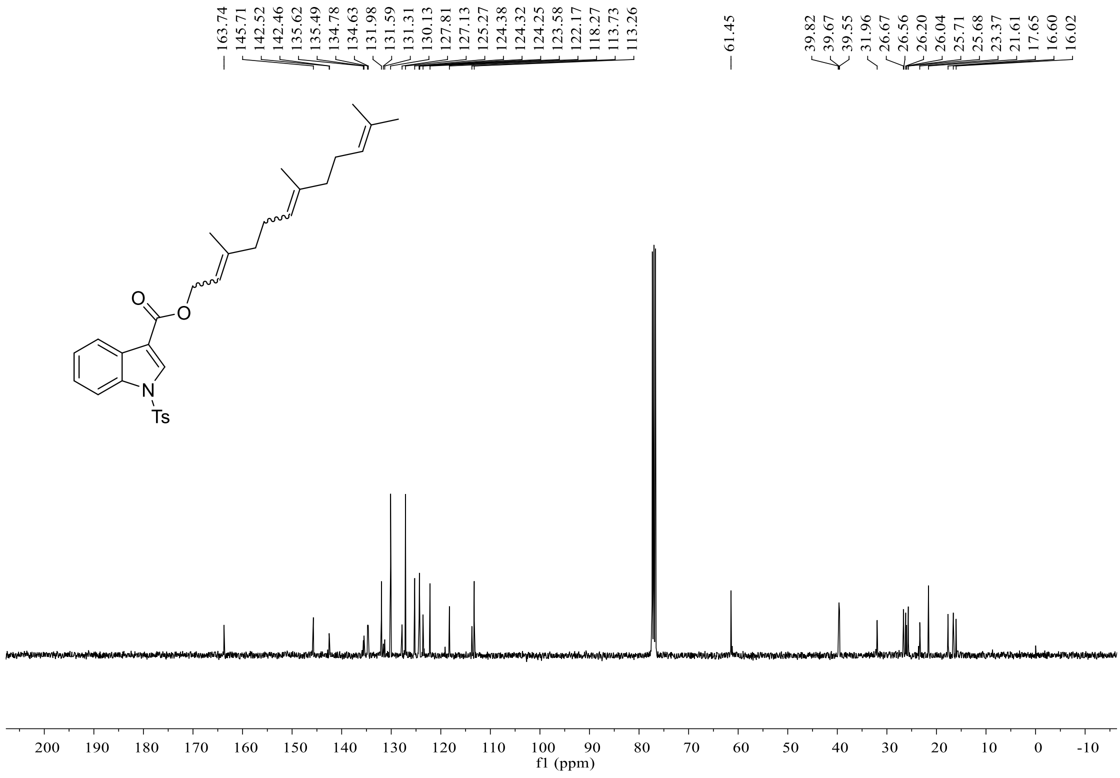

Supplementary Fig. 31. ^{13}C NMR of compound **8b** (100 MHZ, $CDCl_3$)

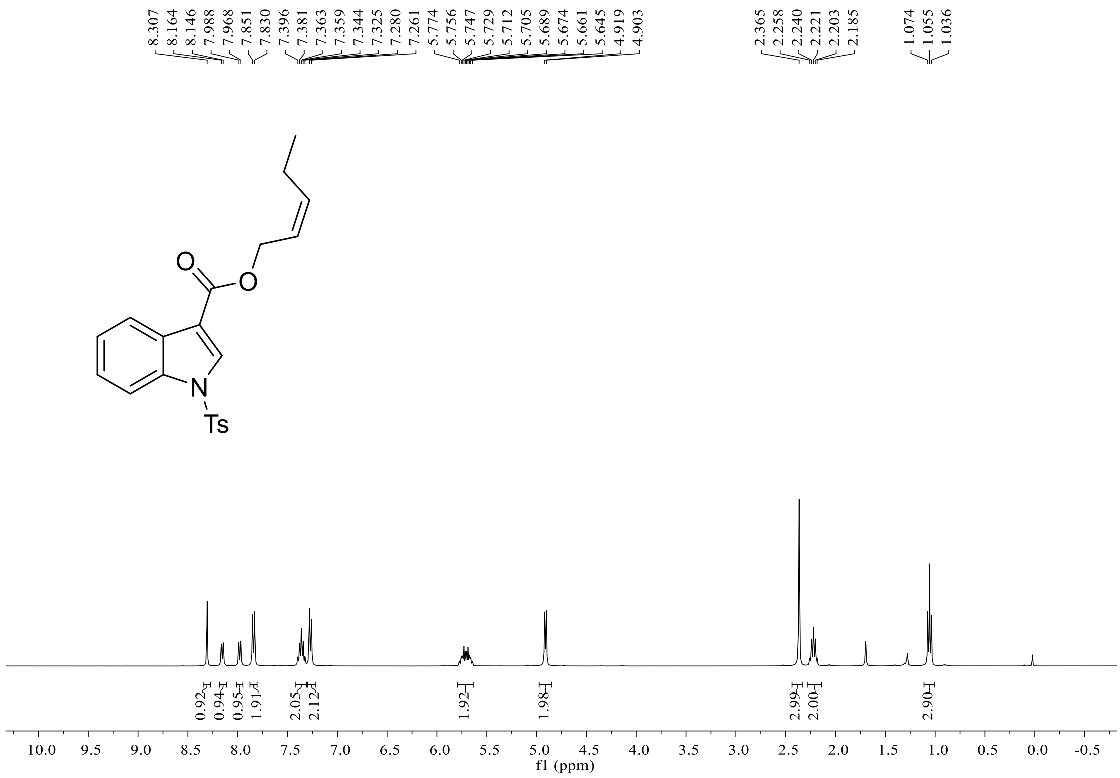

Supplementary Fig. 32. ^{1}H NMR of compound **8c** (400 MHZ, $CDCl_3$)

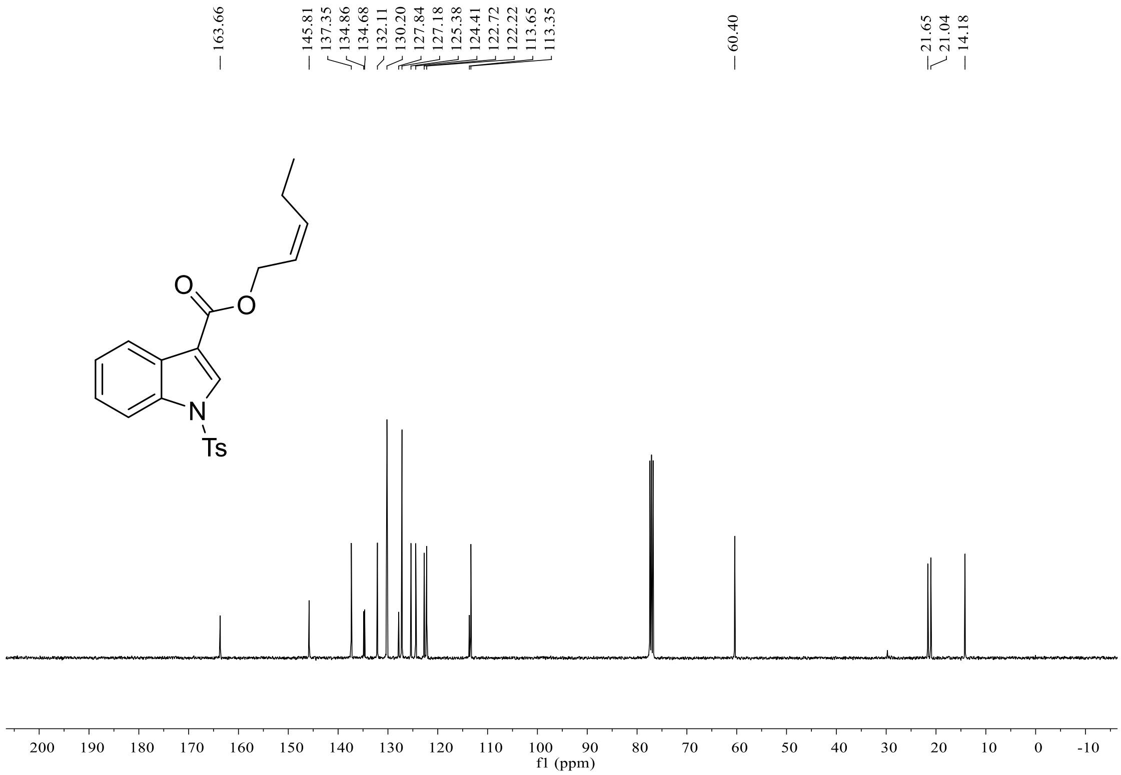

Supplementary Fig. 33. ^{13}C NMR of compound **8c** (100 MHZ, $CDCl_3$)

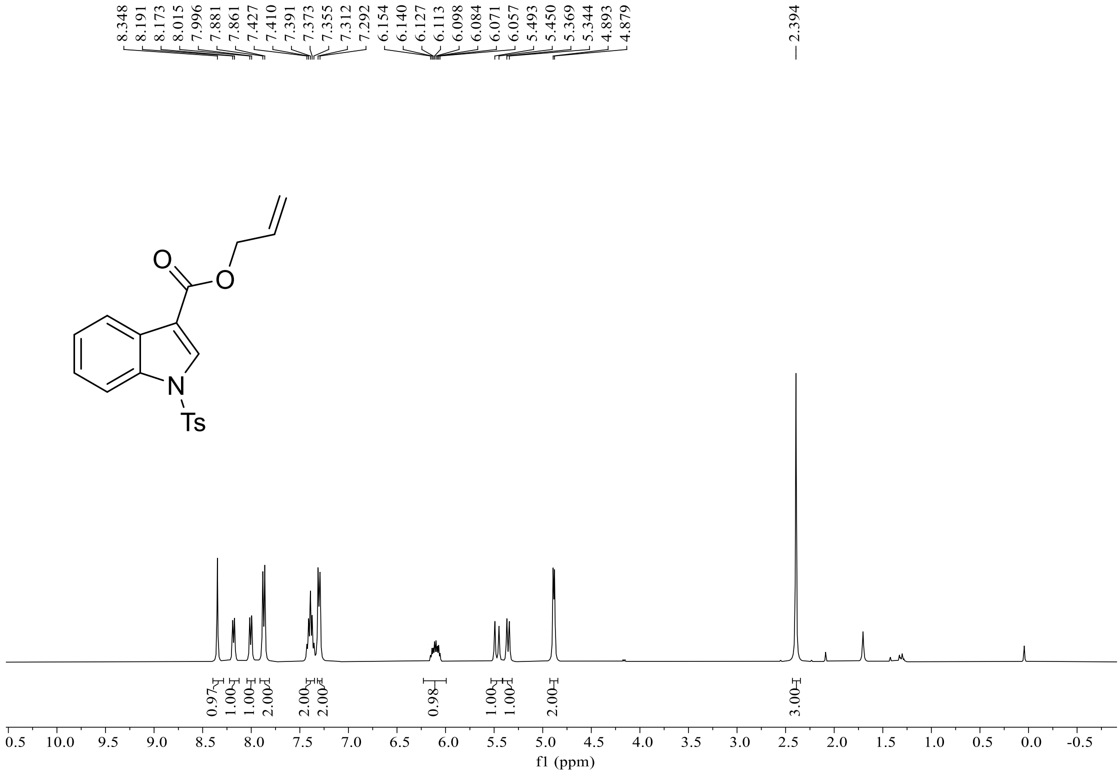

Supplementary Fig. 34. ^{1}H NMR of compound **8d** (400 MHZ, $CDCl_3$)

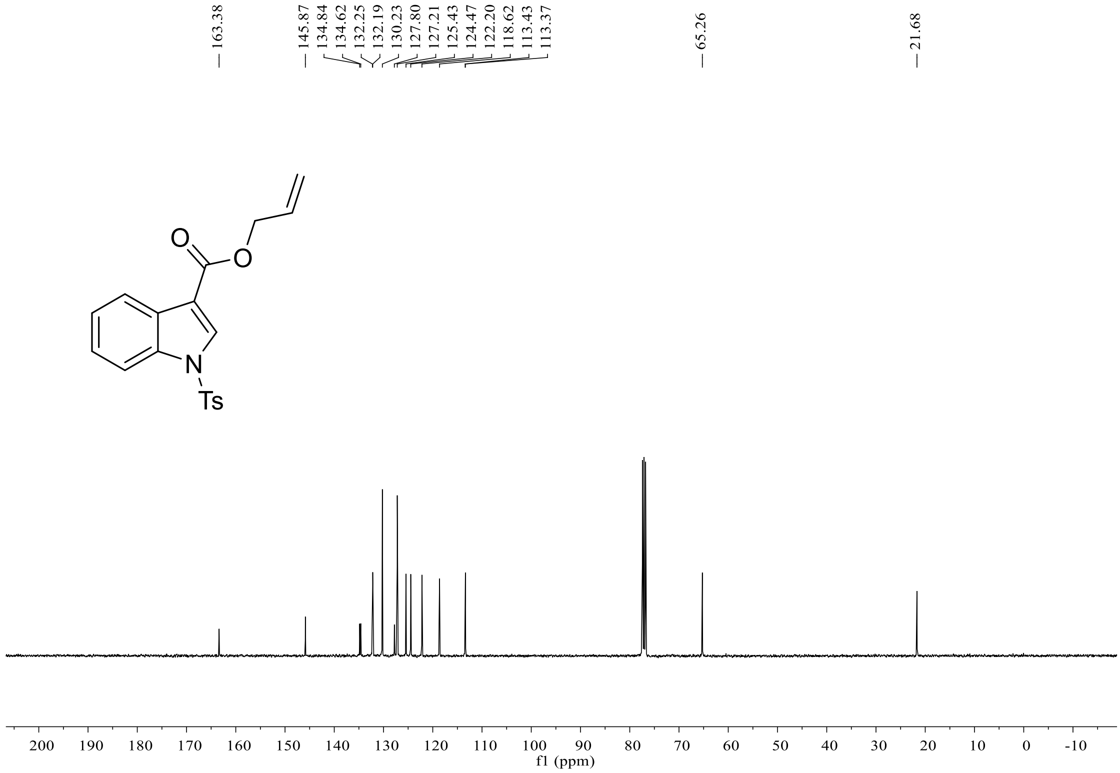

Supplementary Fig. 35. ^{13}C NMR of compound **8d** (100 MHZ, $CDCl_3$)

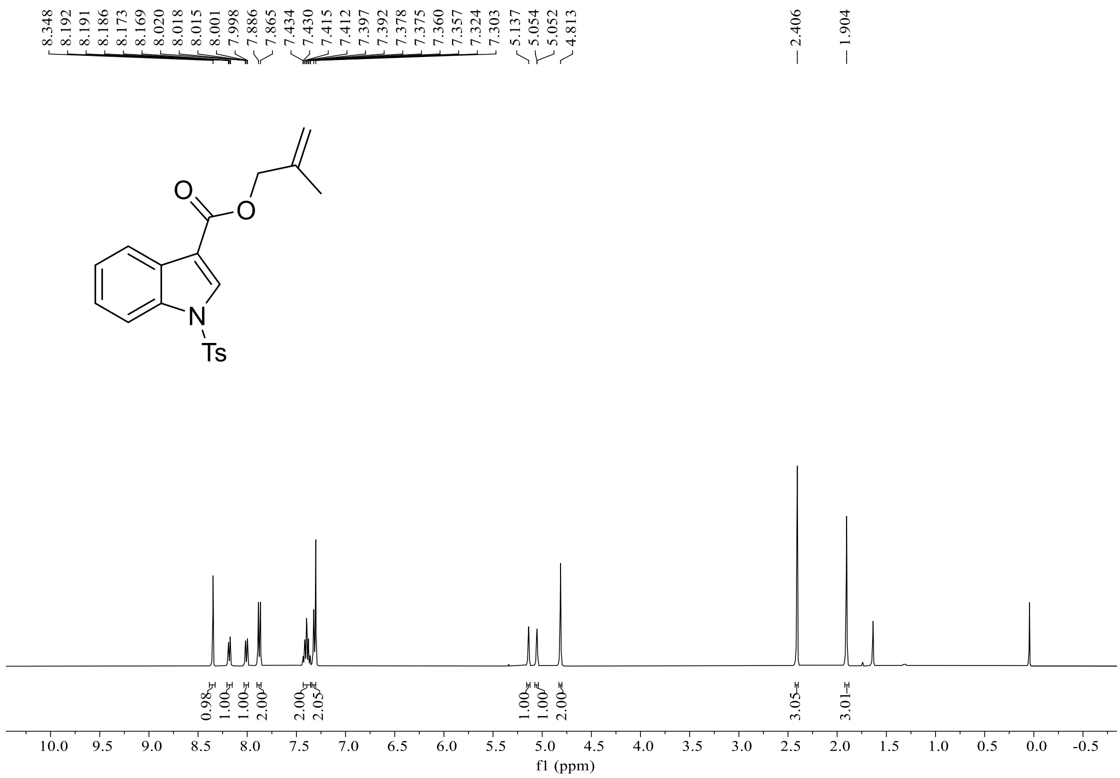

Supplementary Fig. 36. ^1H NMR of compound **8e** (400 MHZ, $CDCl_3$)

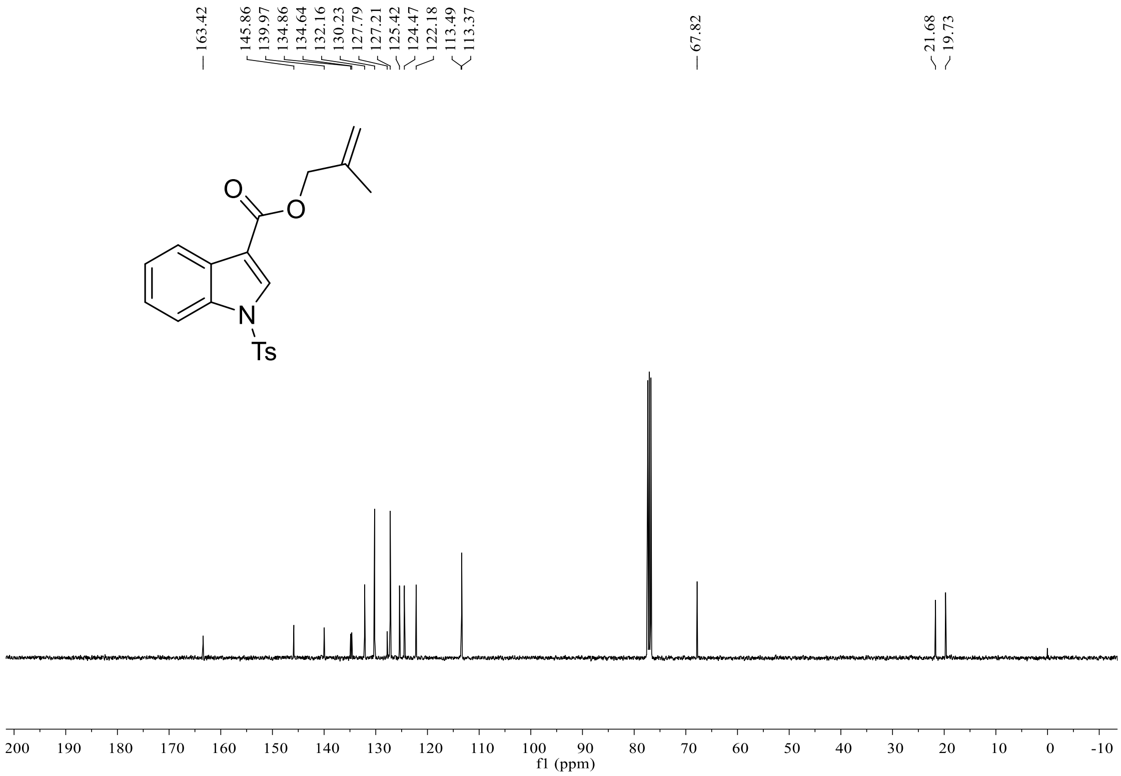

Supplementary Fig. 37. ^{13}C NMR of compound **8e** (100 MHZ, $CDCl_3$)

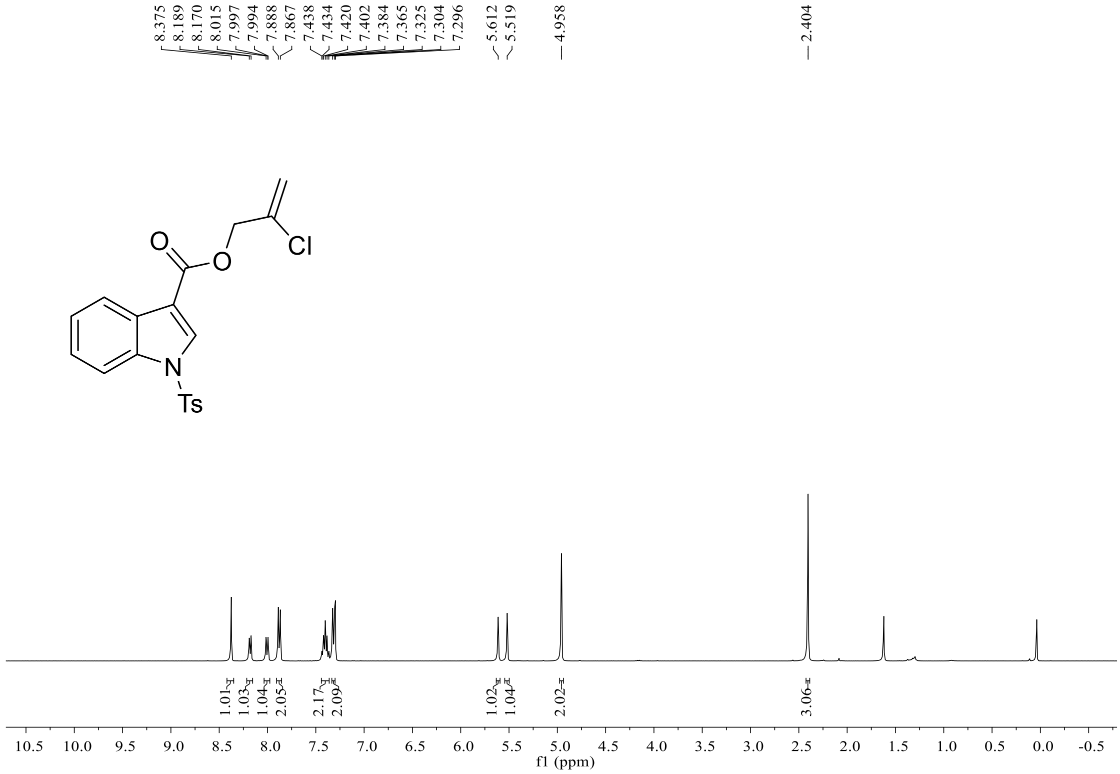

Supplementary Fig. 38. ^{1}H NMR of compound **8f** (400 MHZ, $CDCl_3$)

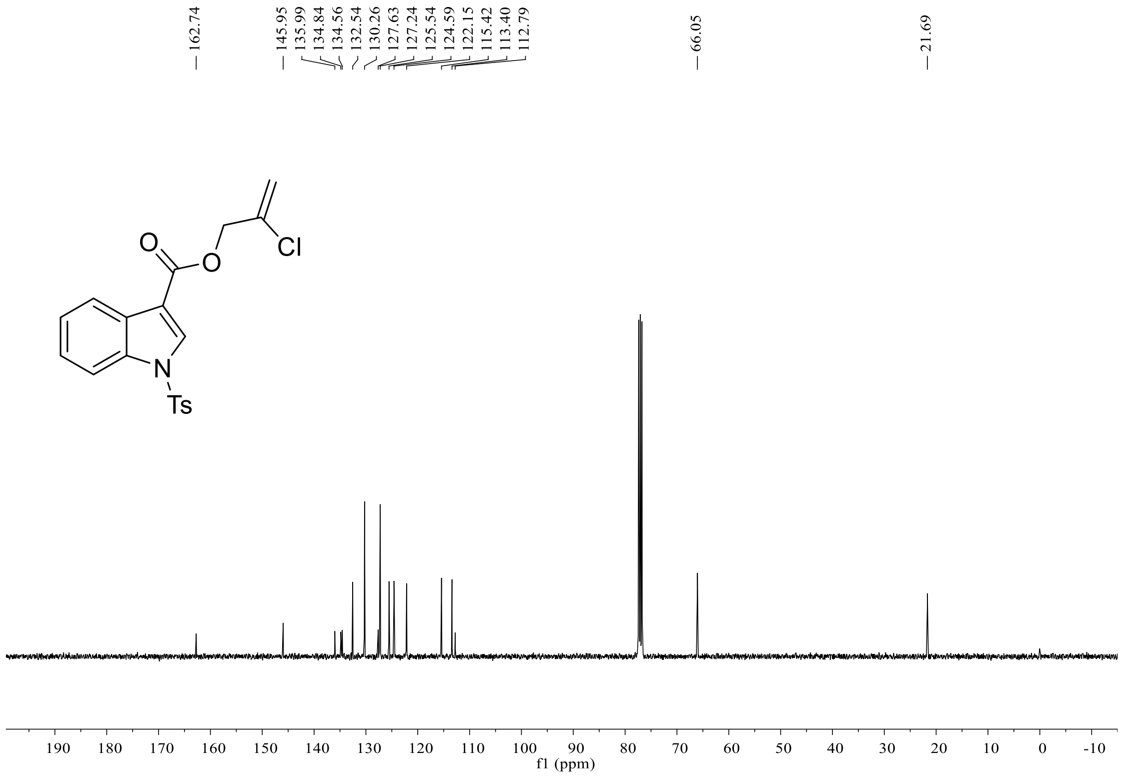

Supplementary Fig. 39. ^{13}C NMR of compound **8f** (100 MHZ, $CDCl_3$)

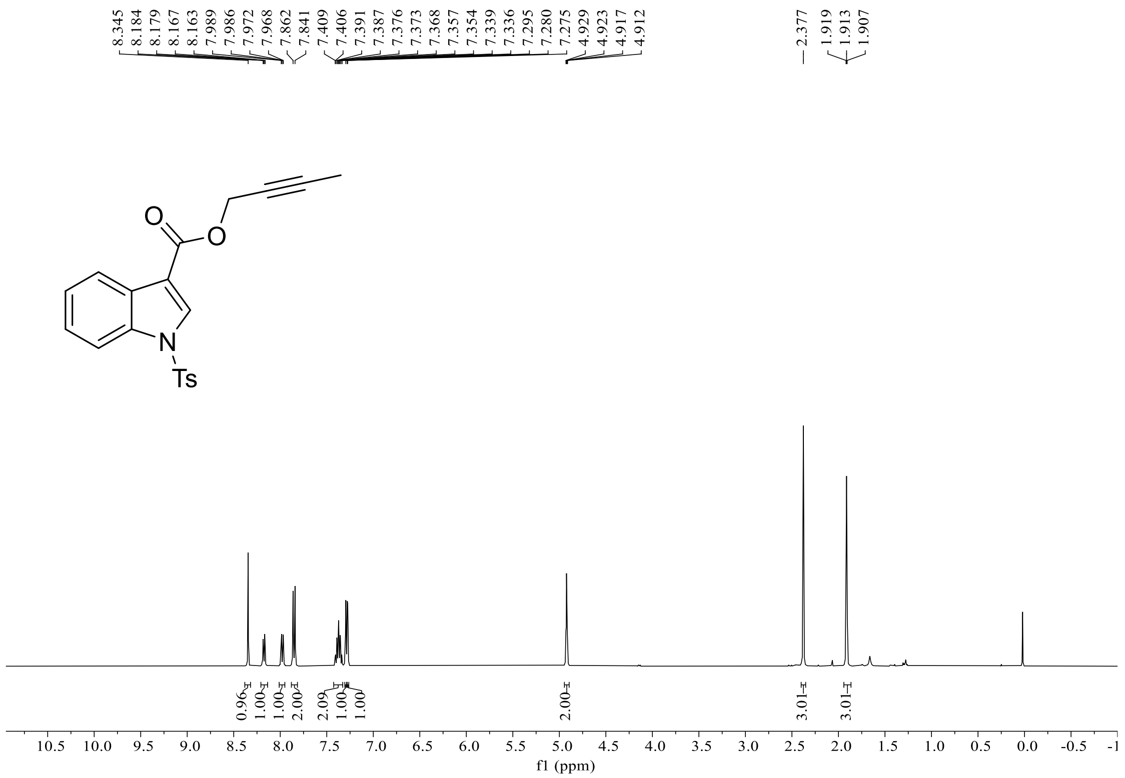

Supplementary Fig. 40. ^{1}H NMR of compound **8g** (400 MHZ, $CDCl_3$)

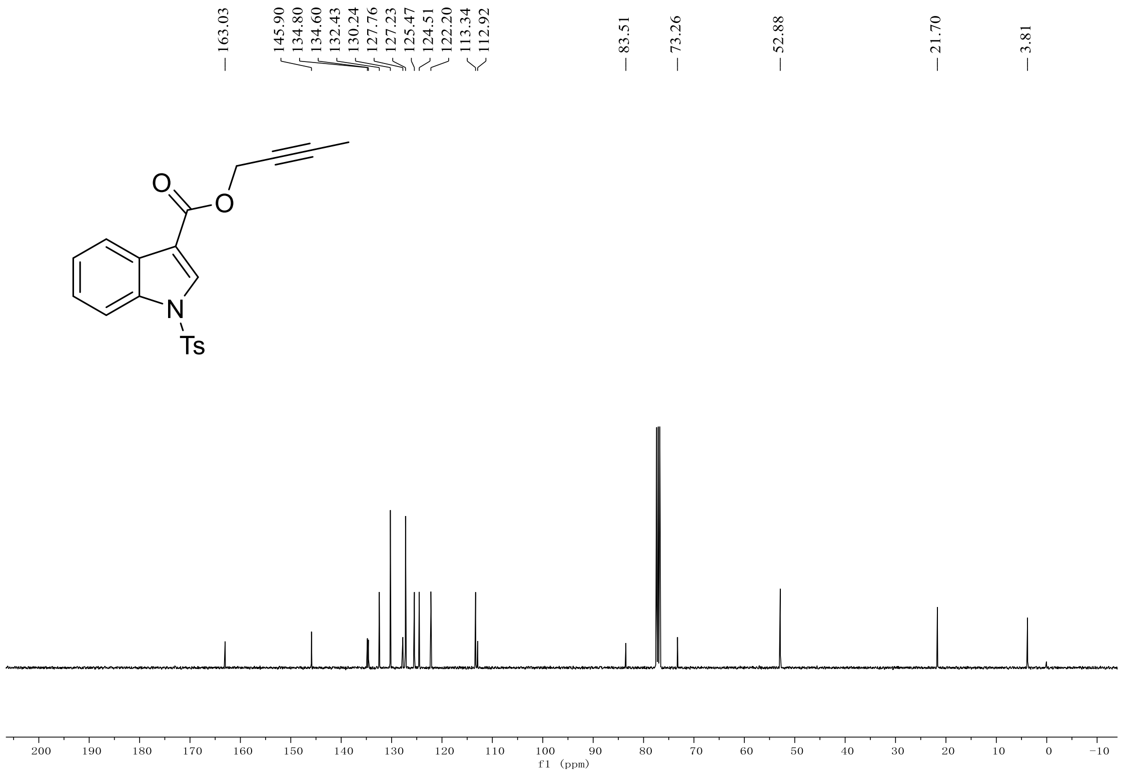

Supplementary Fig. 41. ^{13}C NMR of compound **8g** (100 MHZ, $CDCl_3$)

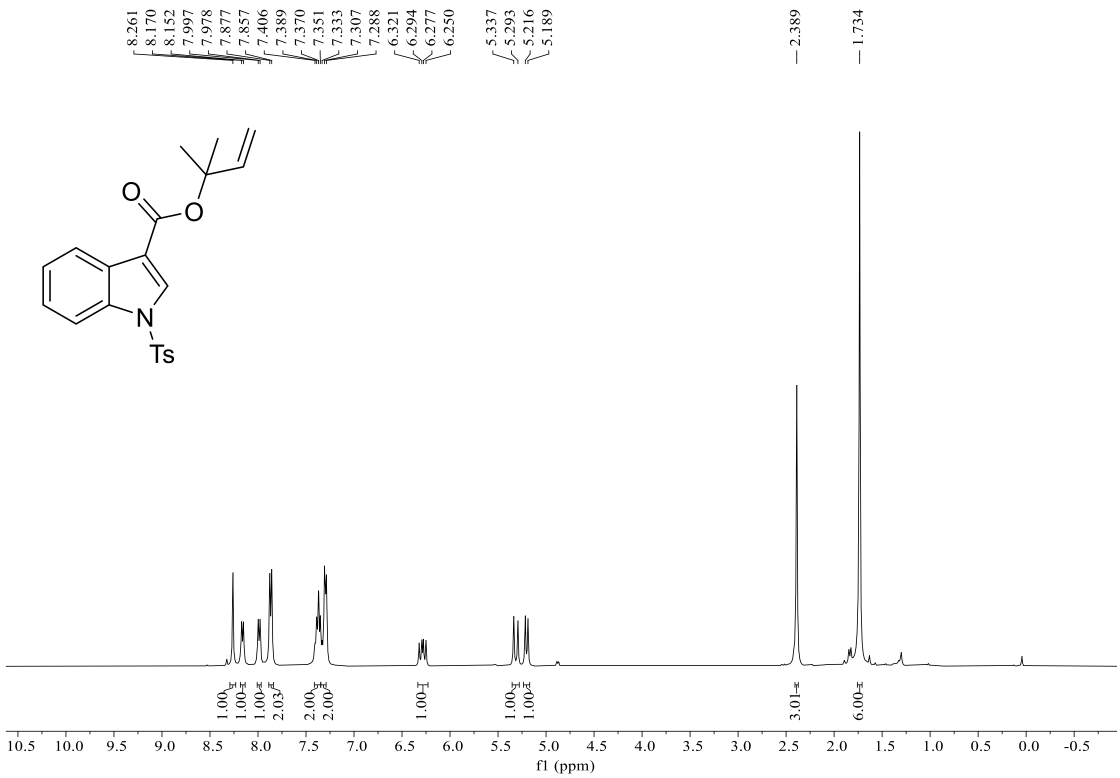

Supplementary Fig. 42. ^{1}H NMR of compound **6** (400 MHZ, $CDCl_3$)

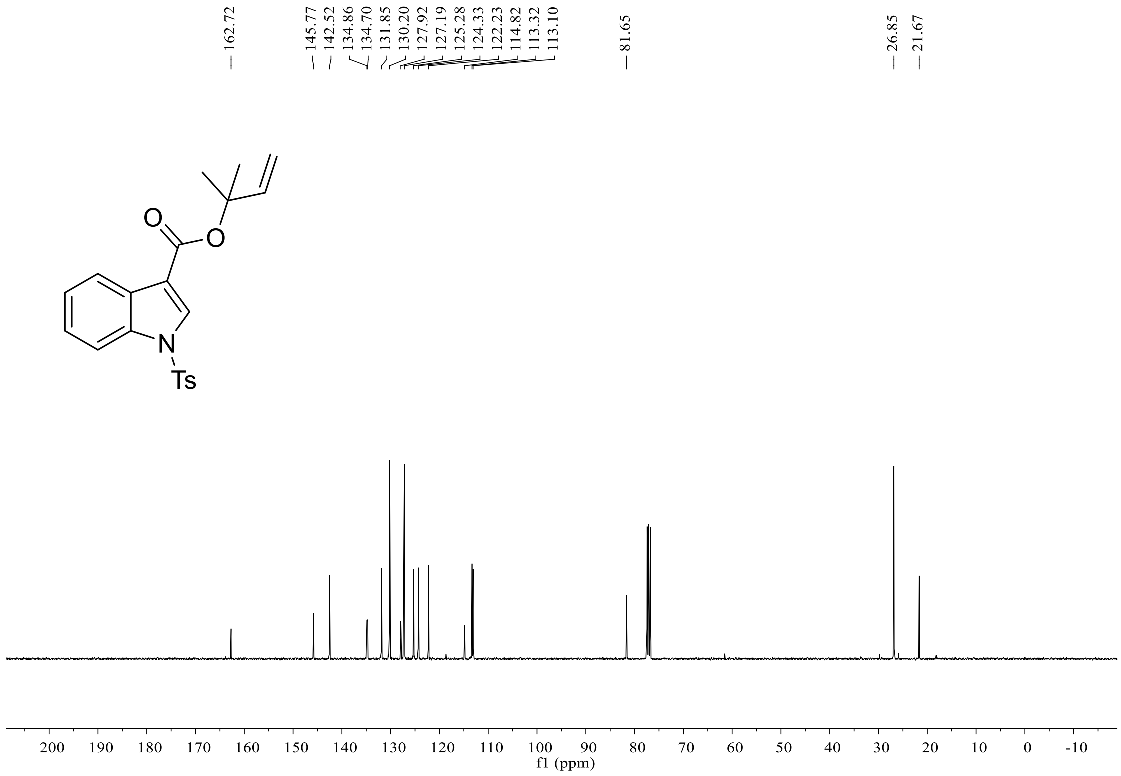

Supplementary Fig. 43. ^{13}C NMR of compound **6** (100 MHZ, $CDCl_3$)

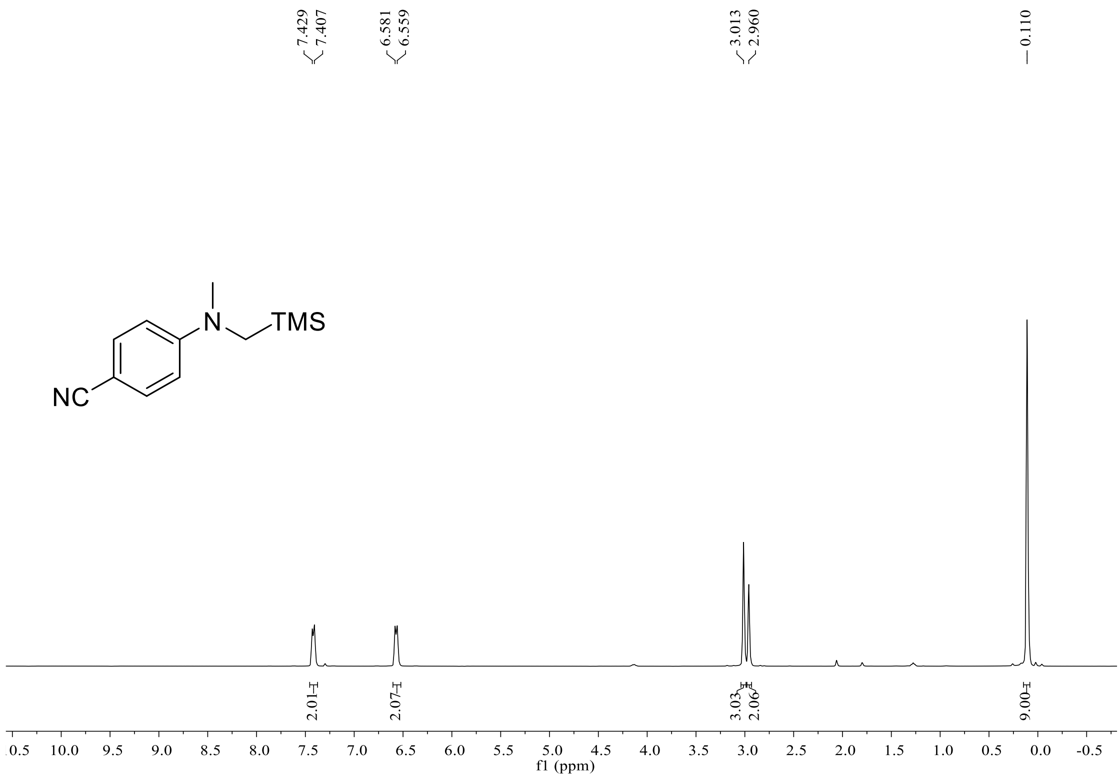

Supplementary Fig. 44. ^{1}H NMR of compound **2g** (400 MHZ, $CDCl_3$)

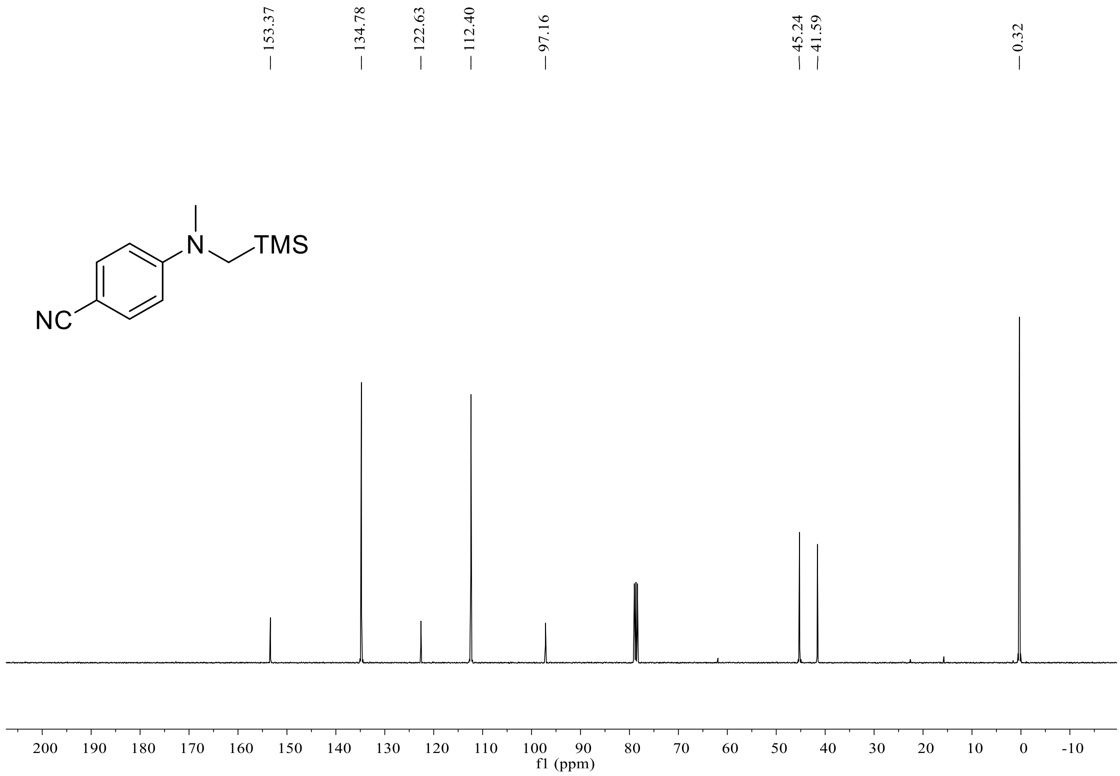

Supplementary Fig. 45. ^{13}C NMR of compound **2g** (100 MHZ, $CDCl_3$)

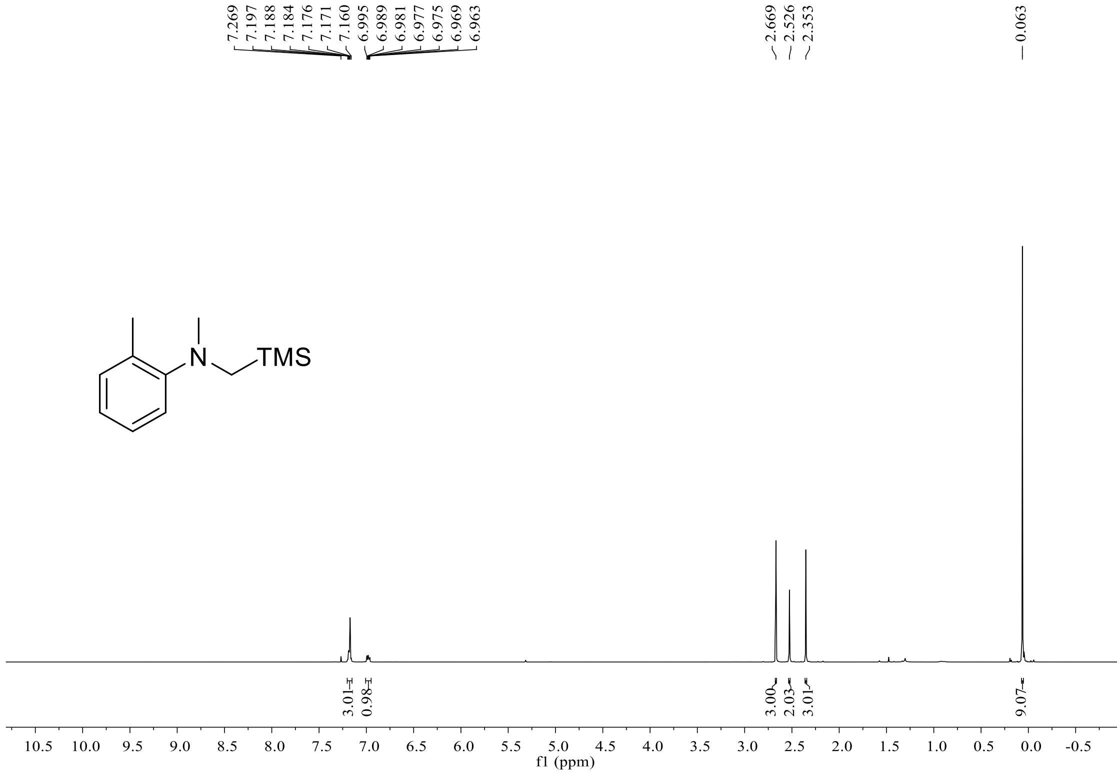

Supplementary Fig. 46. ^{1}H NMR of compound **2i** (500 MHZ, $CDCl_3$)

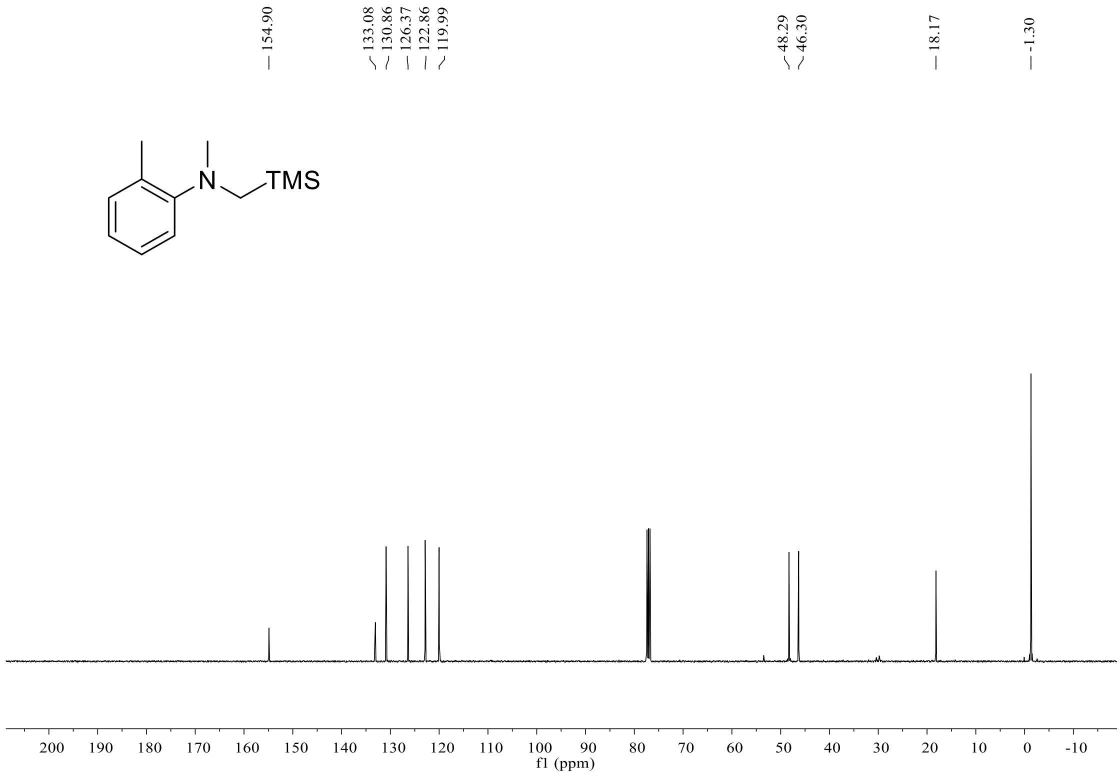

Supplementary Fig. 47. ^{13}C NMR of compound **2i** (125 MHZ, $CDCl_3$)

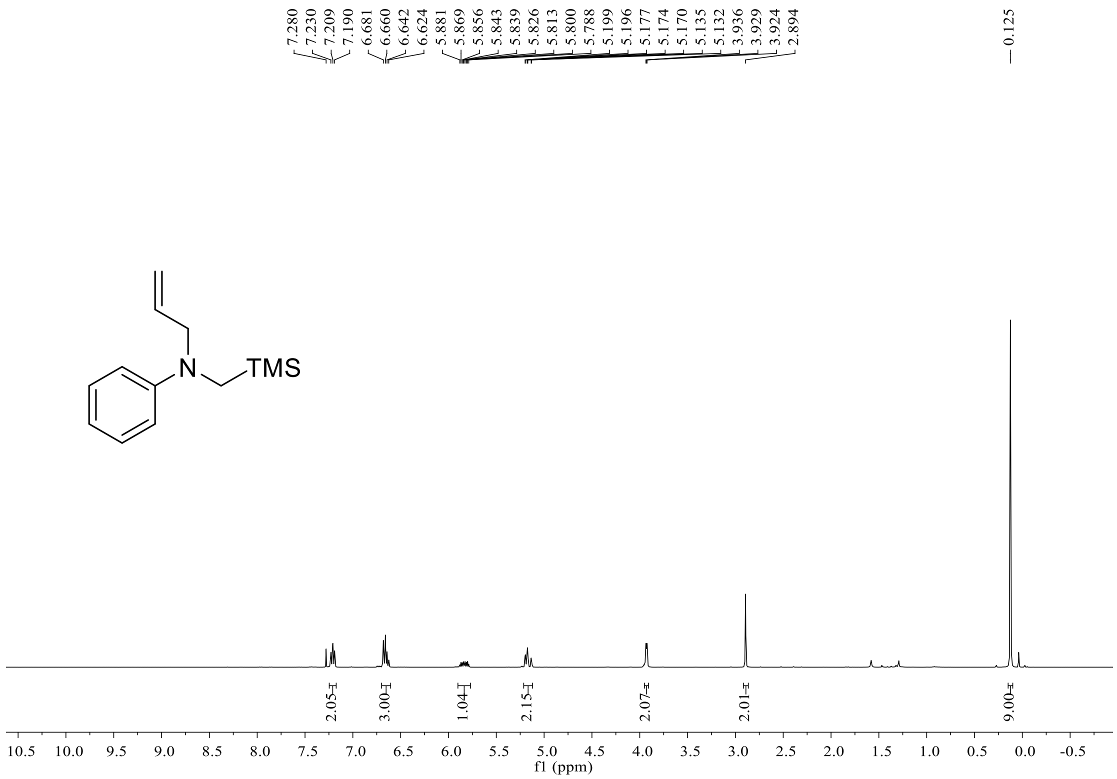

Supplementary Fig. 48. ^{1}H NMR of compound **2j** (400 MHZ, $CDCl_3$)

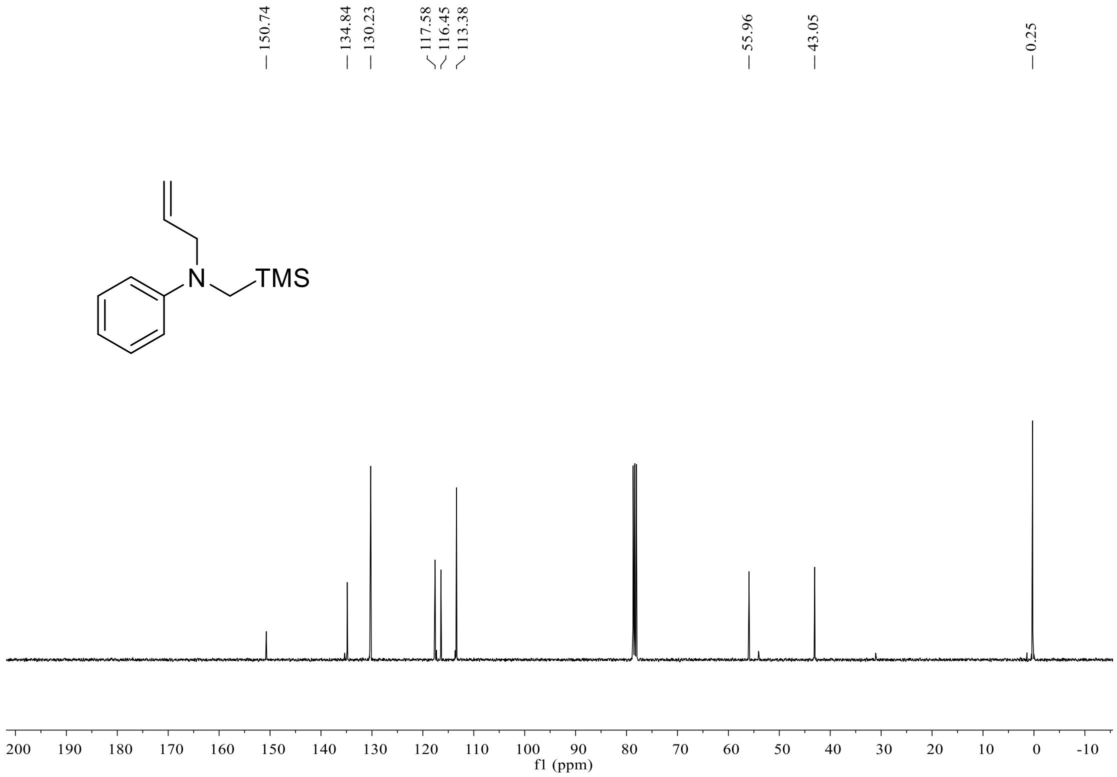

Supplementary Fig. 49. ^{13}C NMR of compound **2j** (100 MHZ, $CDCl_3$)

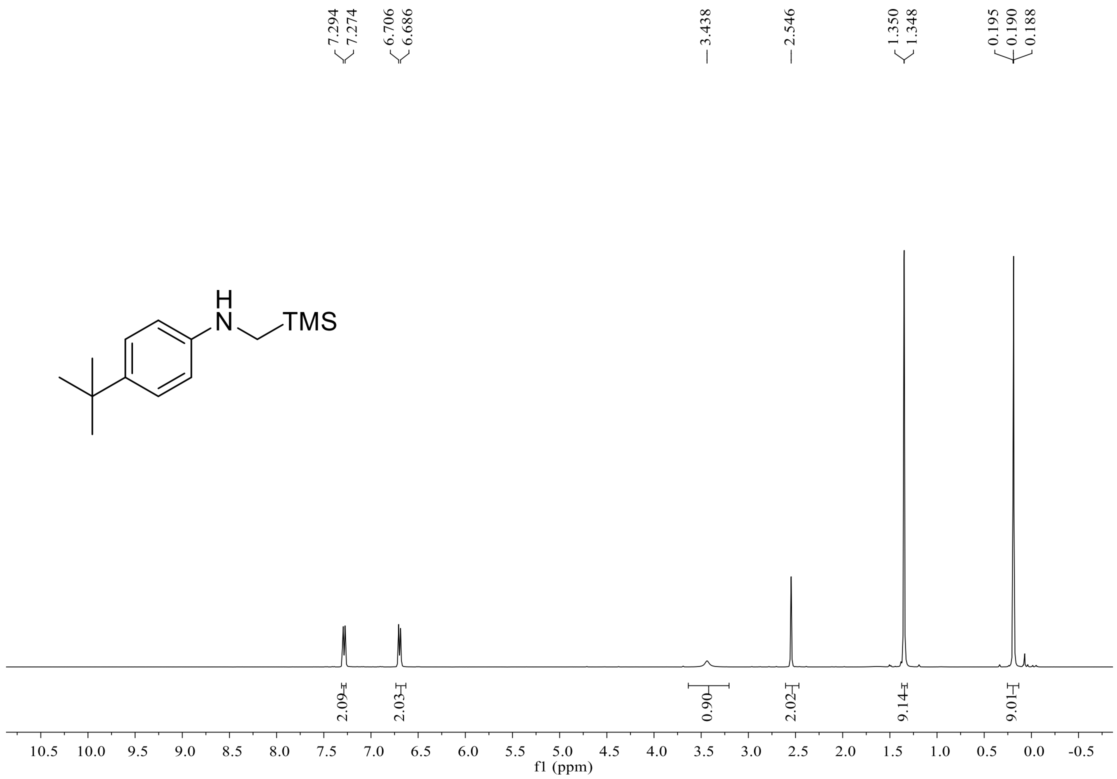

Supplementary Fig. 50. ^{1}H NMR of compound **4d** (400 MHZ, $CDCl_3$)

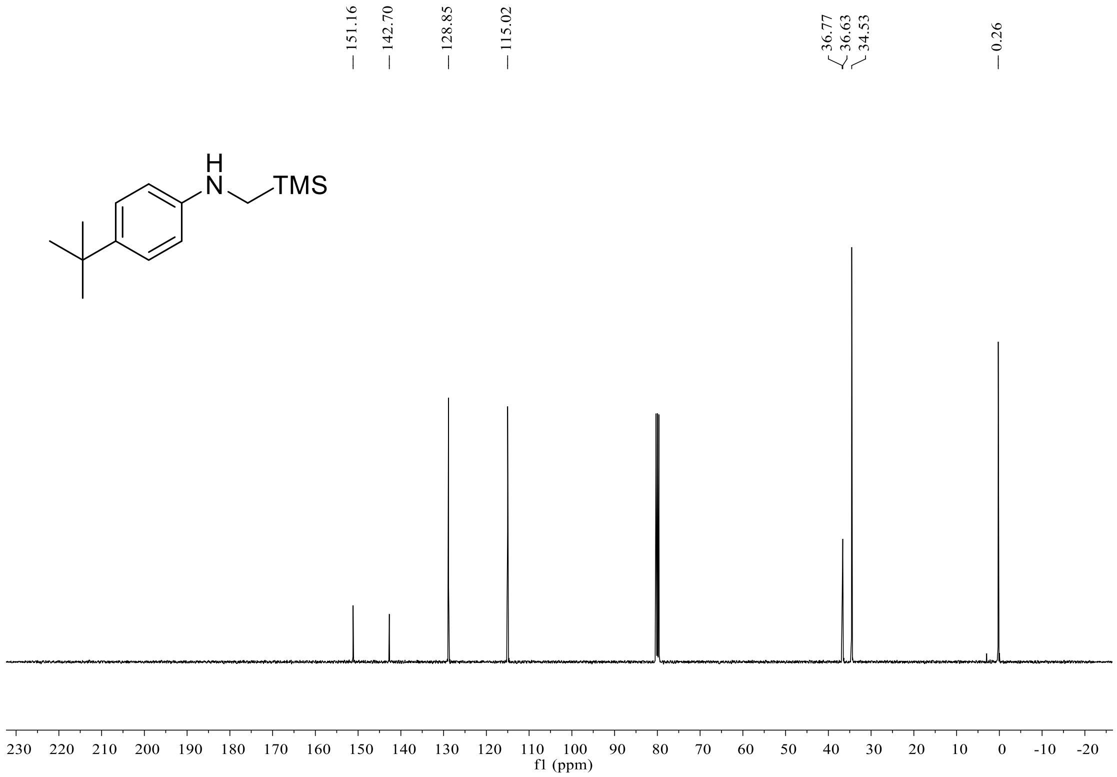

Supplementary Fig. 51. ^{13}C NMR of compound **4d** (100 MHZ, $CDCl_3$)

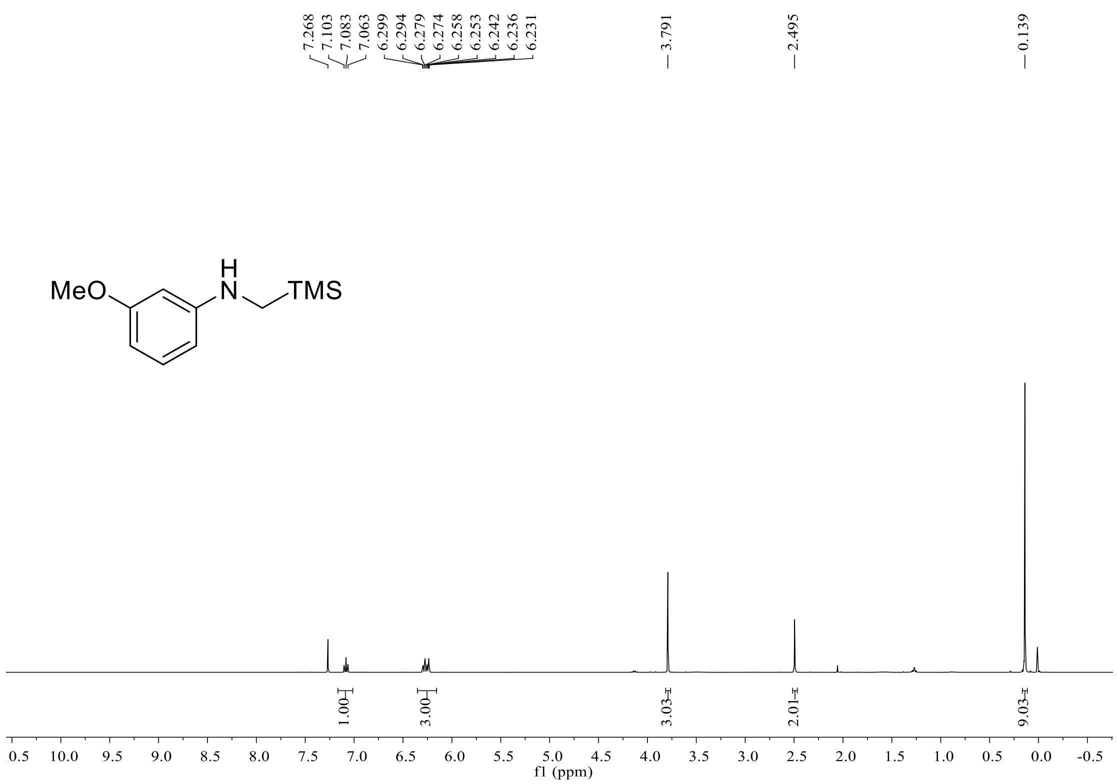

Supplementary Fig. 52. ^{1}H NMR of compound **4h** (400 MHZ, $CDCl_3$)

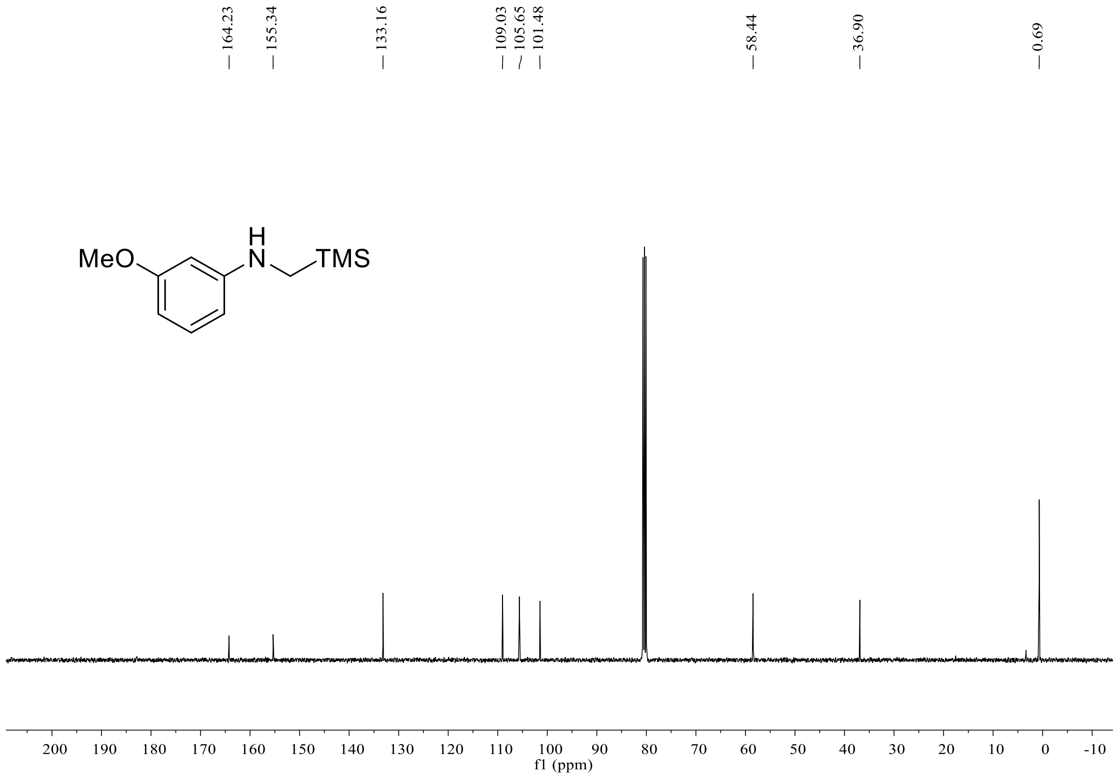

Supplementary Fig. 53. ^{13}C NMR of compound **4h** (100 MHZ, $CDCl_3$)

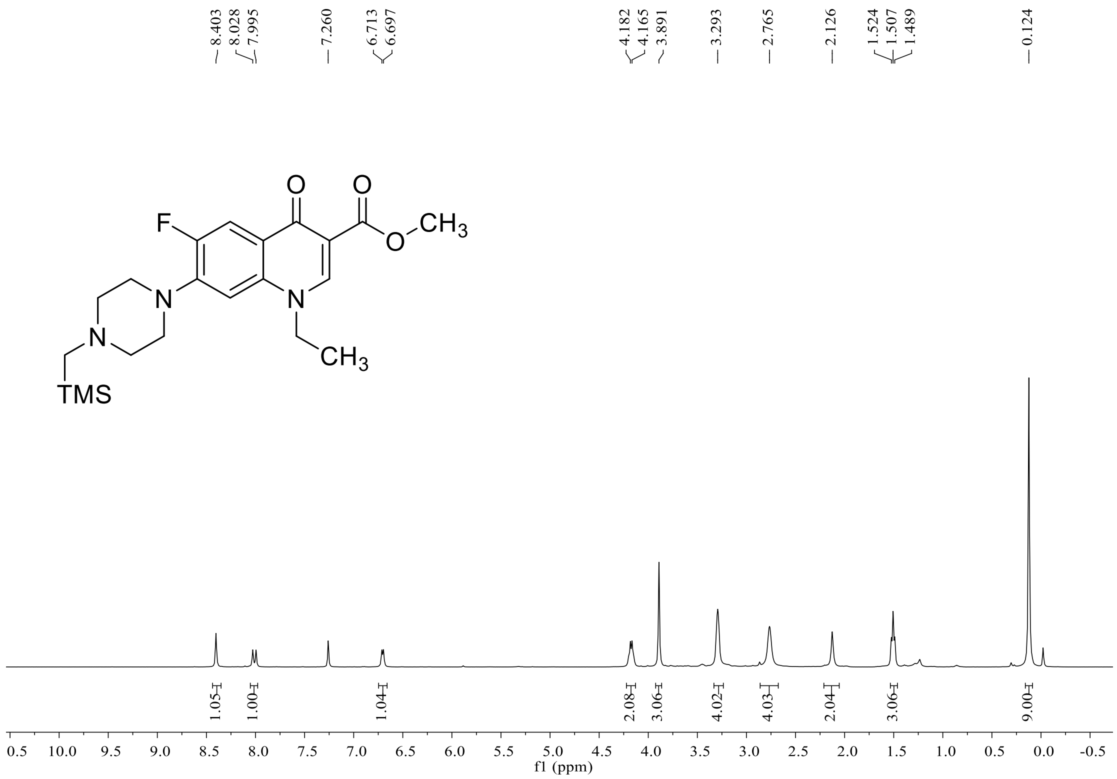

Supplementary Fig. 54. ^{1}H NMR of compound **2da** (400 MHZ, $CDCl_3$)

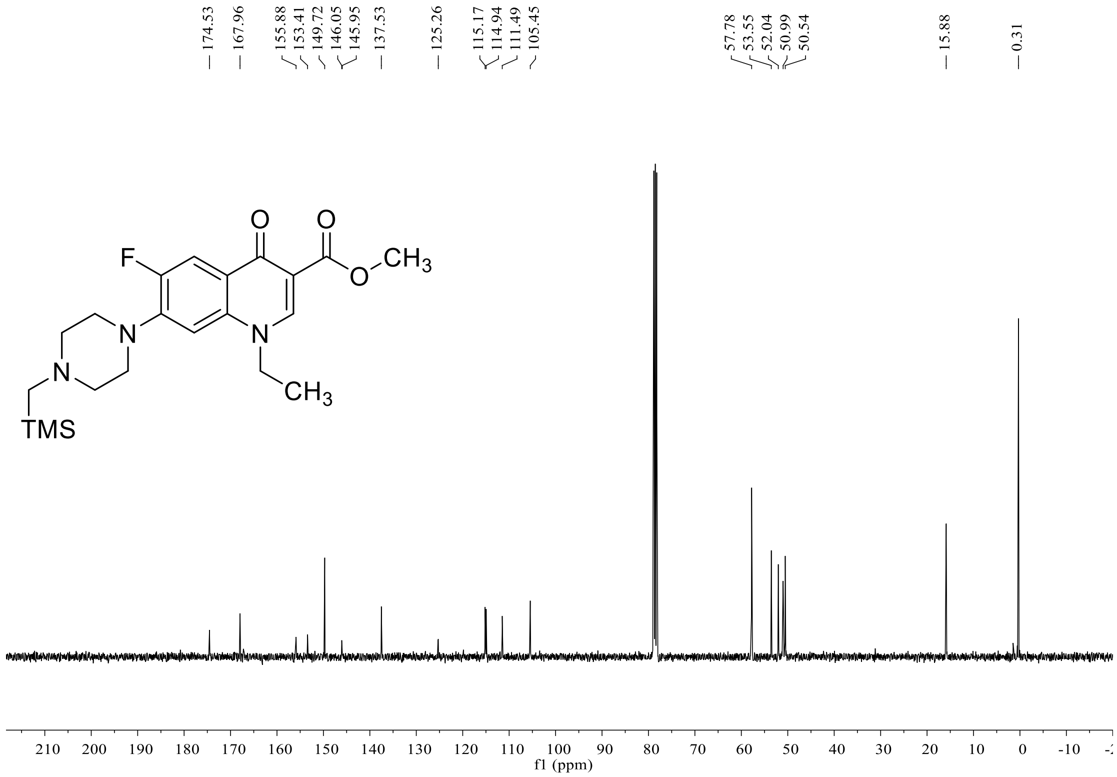

Supplementary Fig. 55. ^{13}C NMR of compound **2da** (100 MHZ, $CDCl_3$)

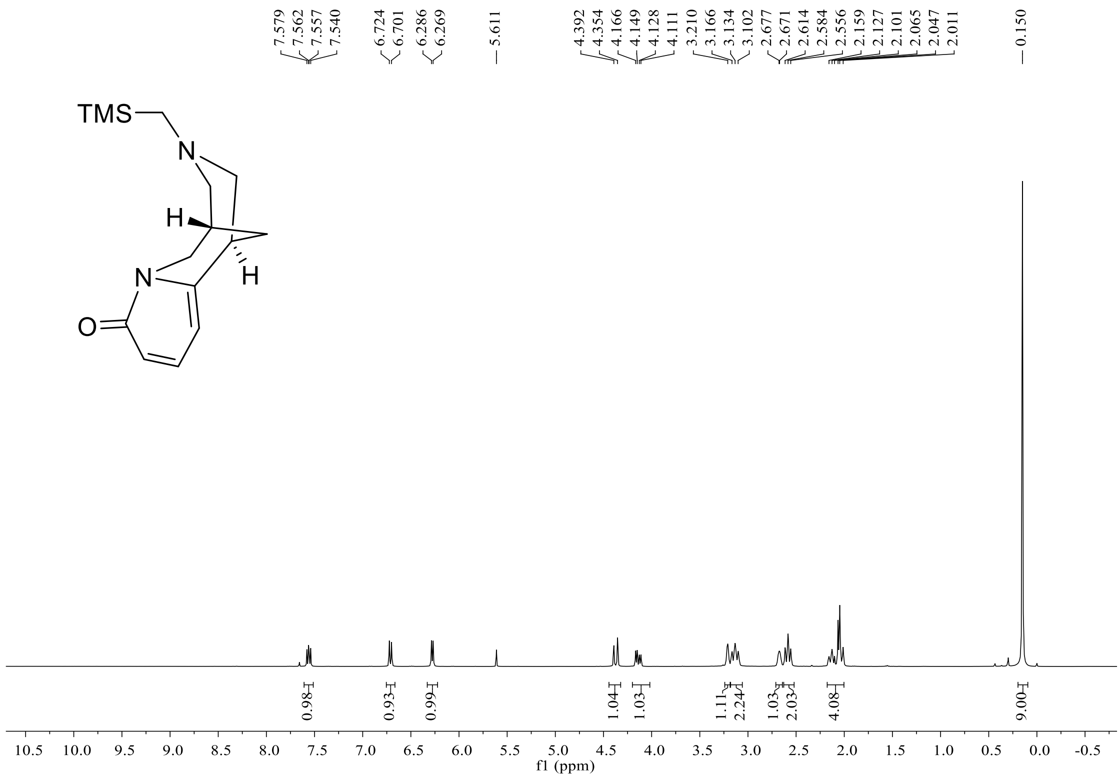

Supplementary Fig. 56. ^{1}H NMR of compound **2db** (400 MHZ, $CDCl_3$)

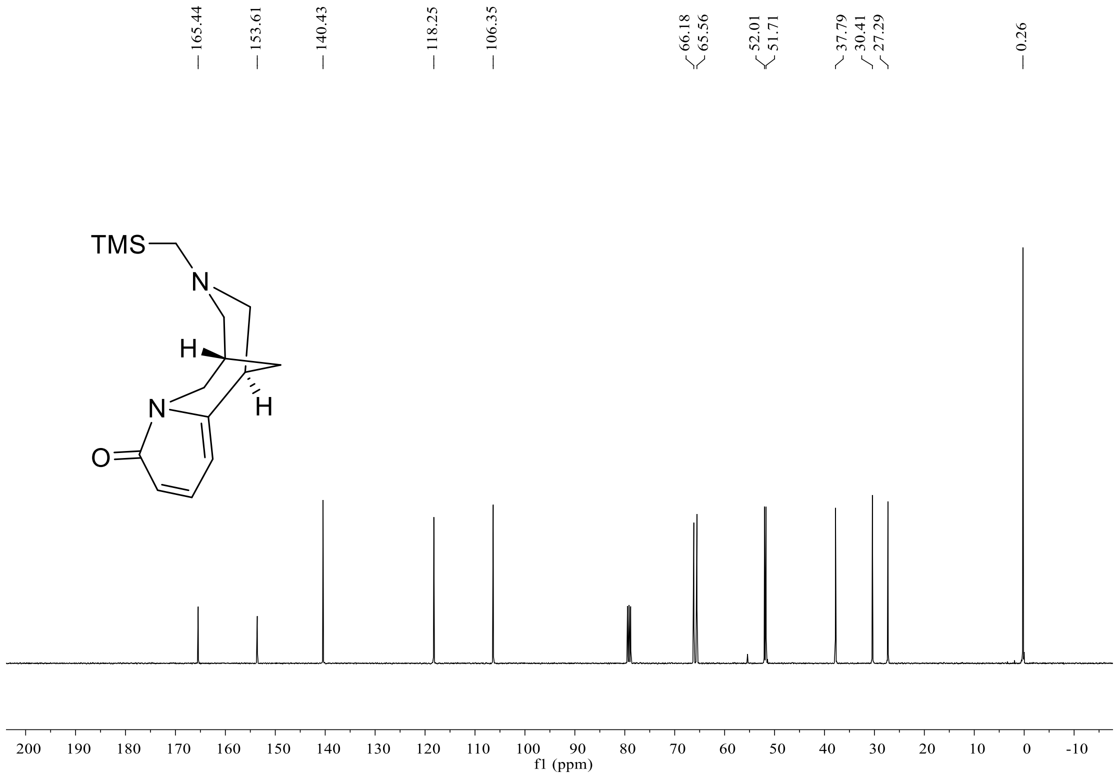

Supplementary Fig. 57. ^{13}C NMR of compound **2db** (100 MHZ, $CDCl_3$)

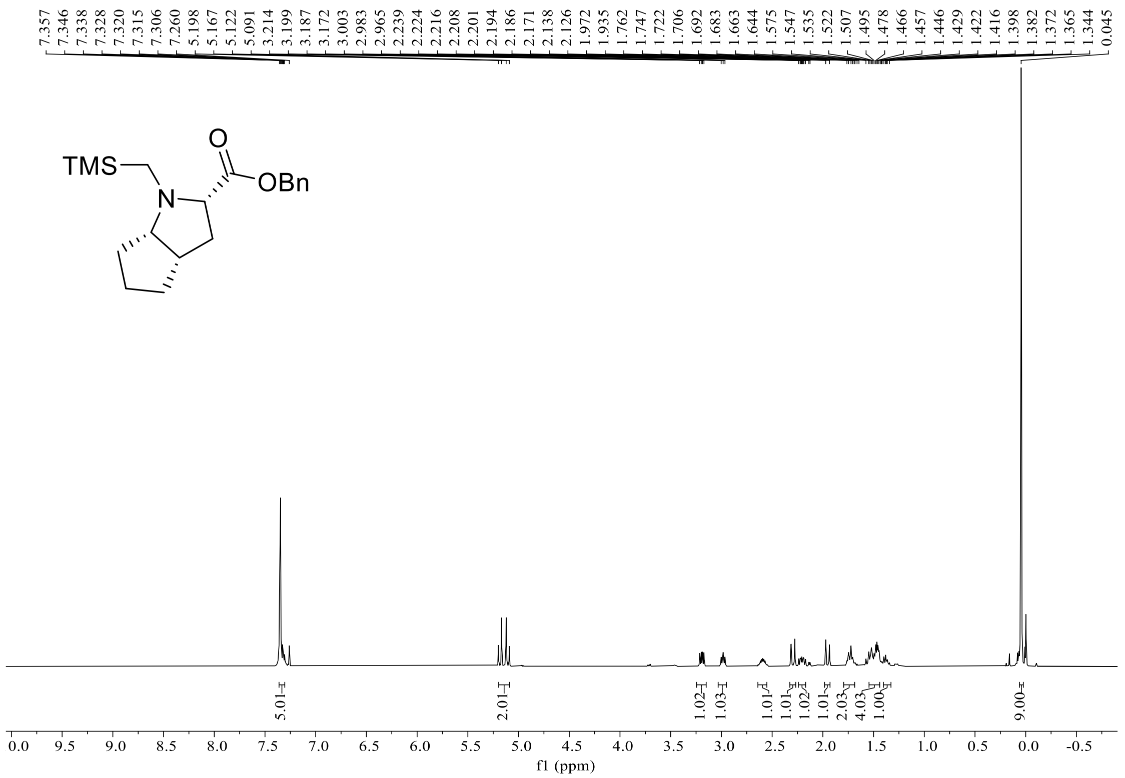

Supplementary Fig. 58. ^{1}H NMR of compound **2de** (400 MHZ, $CDCl_3$)

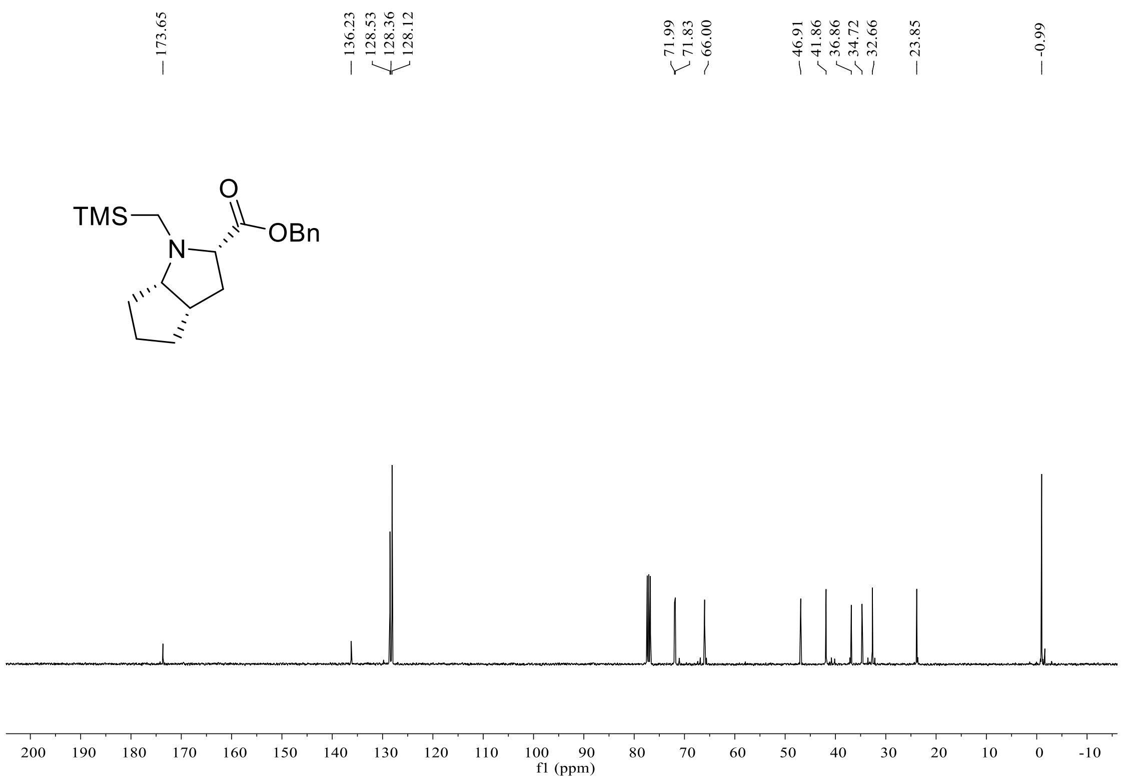

Supplementary Fig. 59. ^{13}C NMR of compound **2de** (100 MHZ, $CDCl_3$)

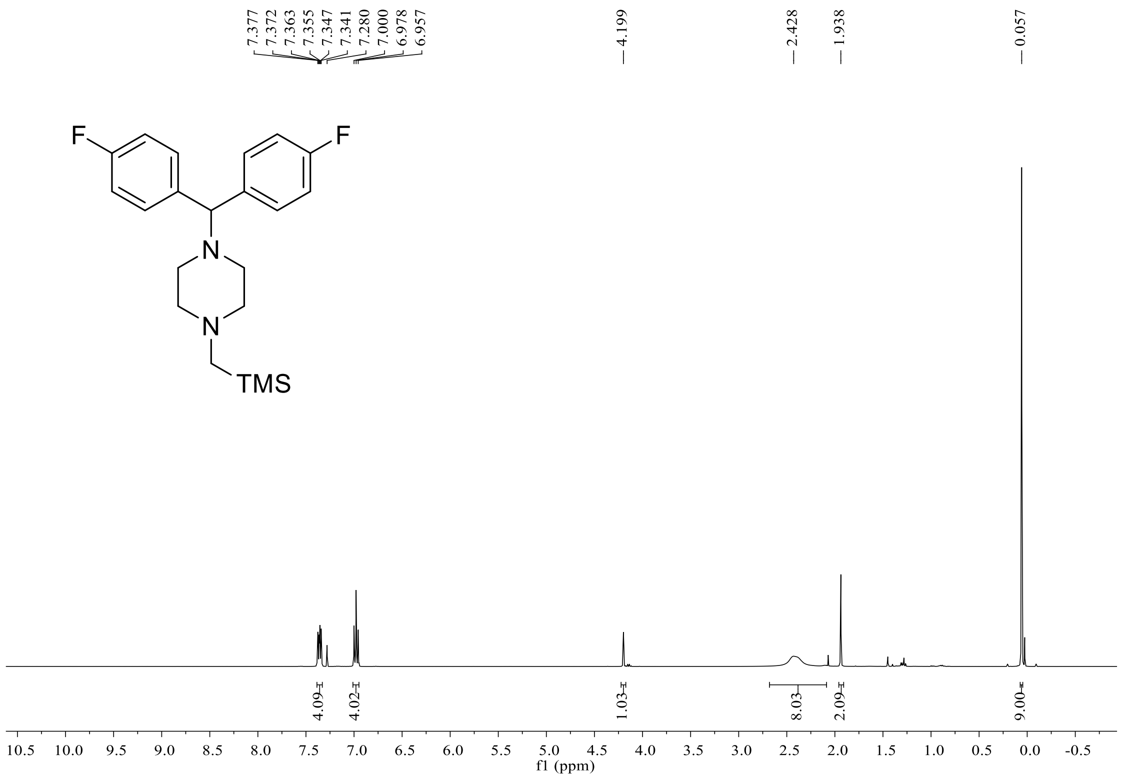

Supplementary Fig. 60. ^{1}H NMR of compound **2df** (400 MHZ, $CDCl_3$)

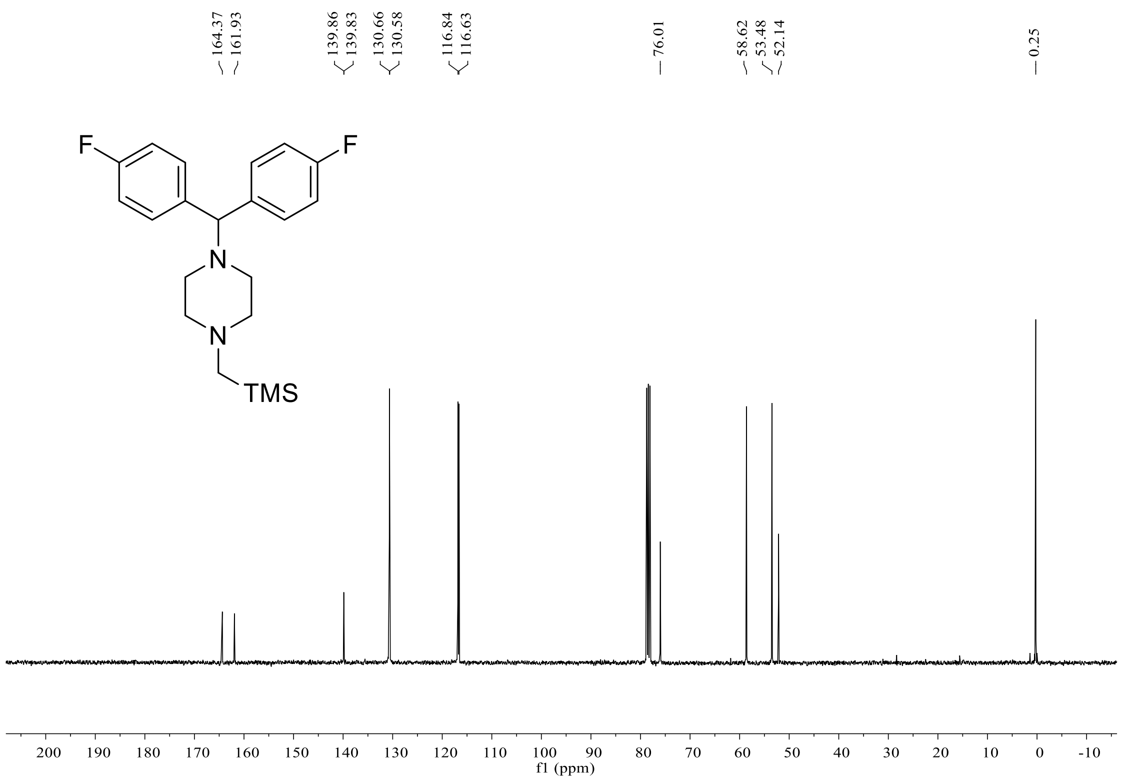

Supplementary Fig. 61. ^{13}C NMR of compound **2df** (100 MHZ, $CDCl_3$)

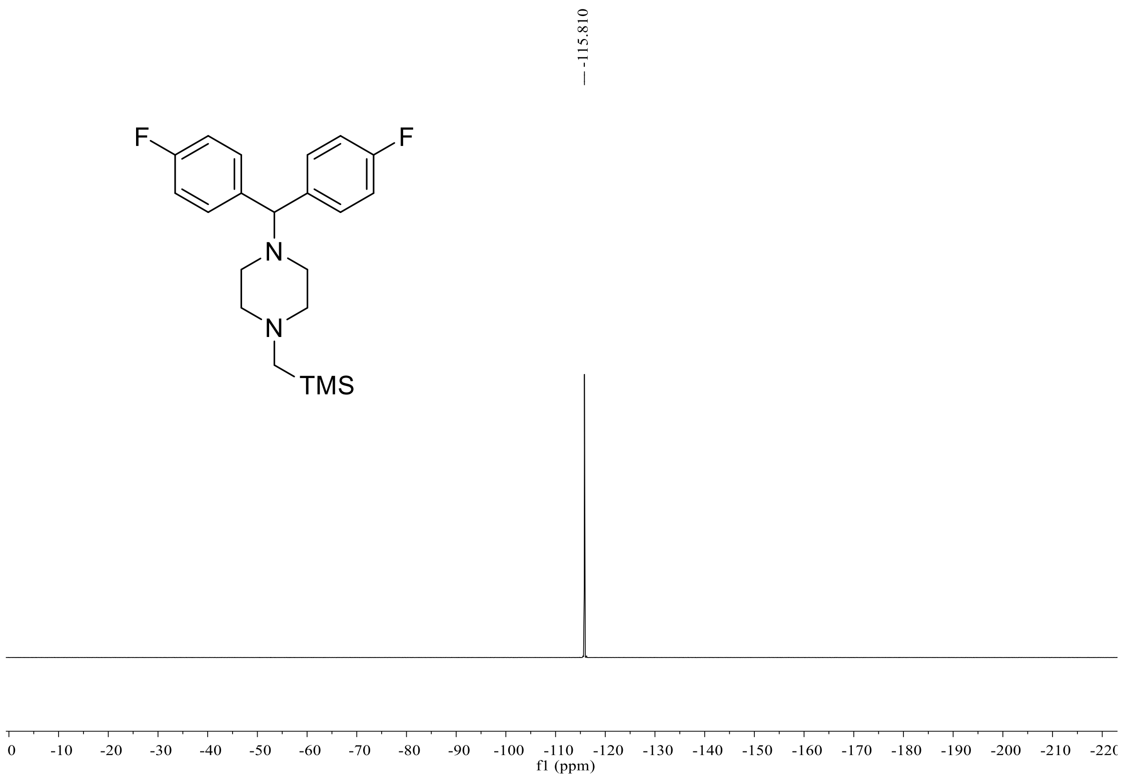

Supplementary Fig. 62. ^{19}F NMR of compound **2df** (376 MHZ, $CDCl_3$)

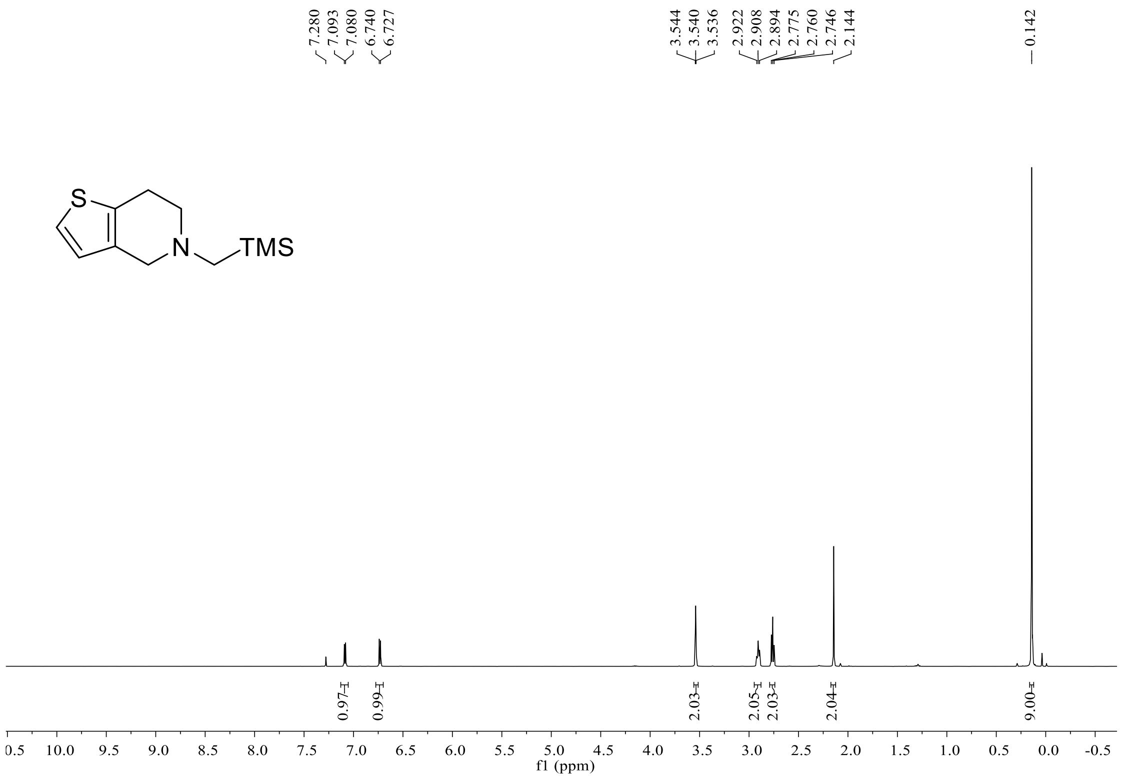

Supplementary Fig. 63. ^{1}H NMR of compound **2dg** (400 MHZ, $CDCl_3$)

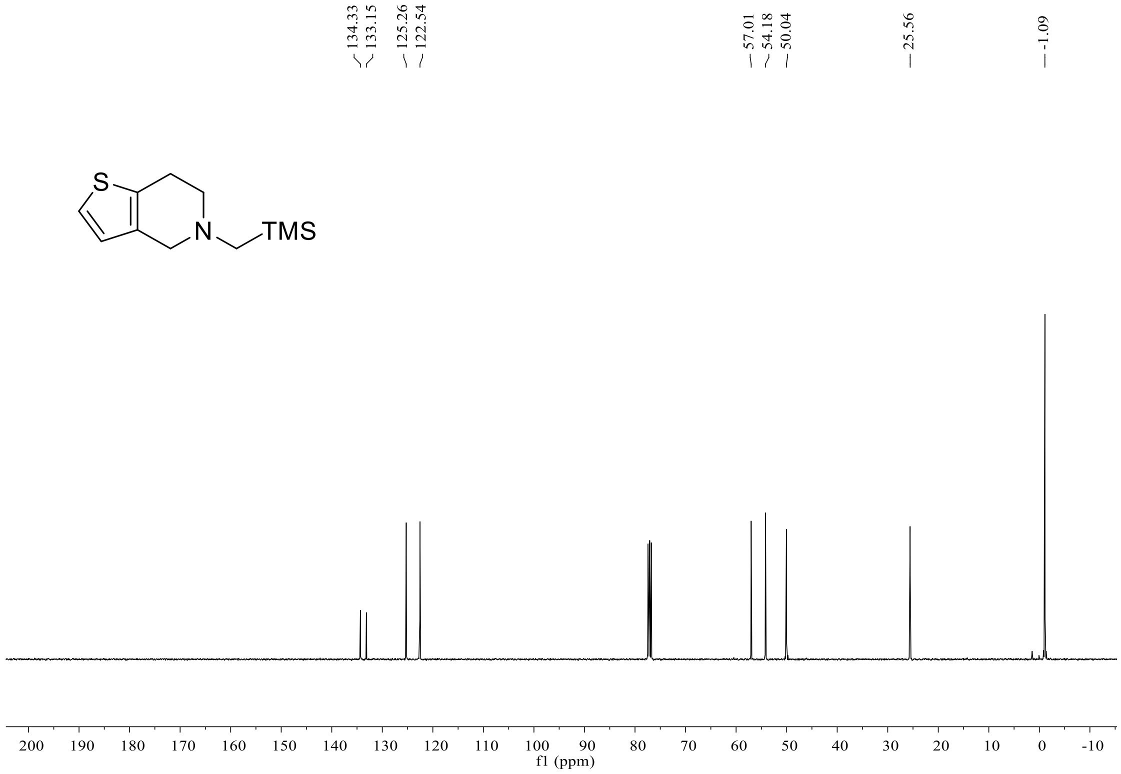

Supplementary Fig. 64. ^{13}C NMR of compound **2dg** (100 MHZ, $CDCl_3$)

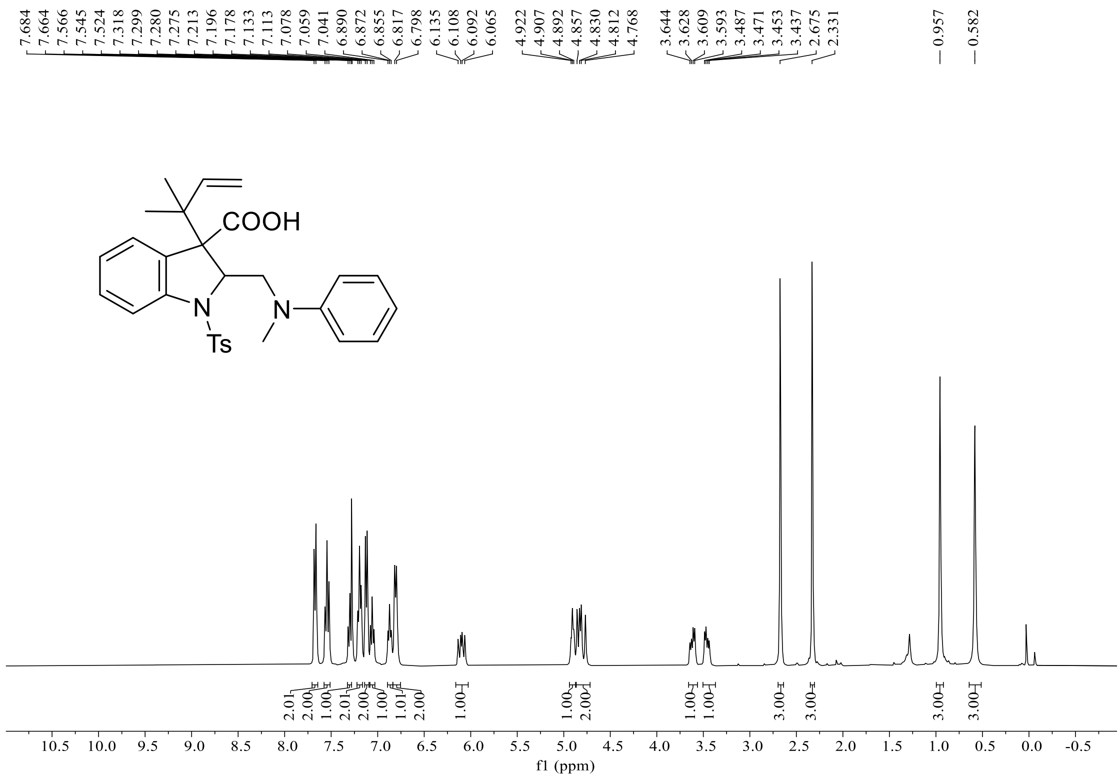

Supplementary Fig. 65. ^1H NMR of compound **3d** (400 MHZ, $CDCl_3$)

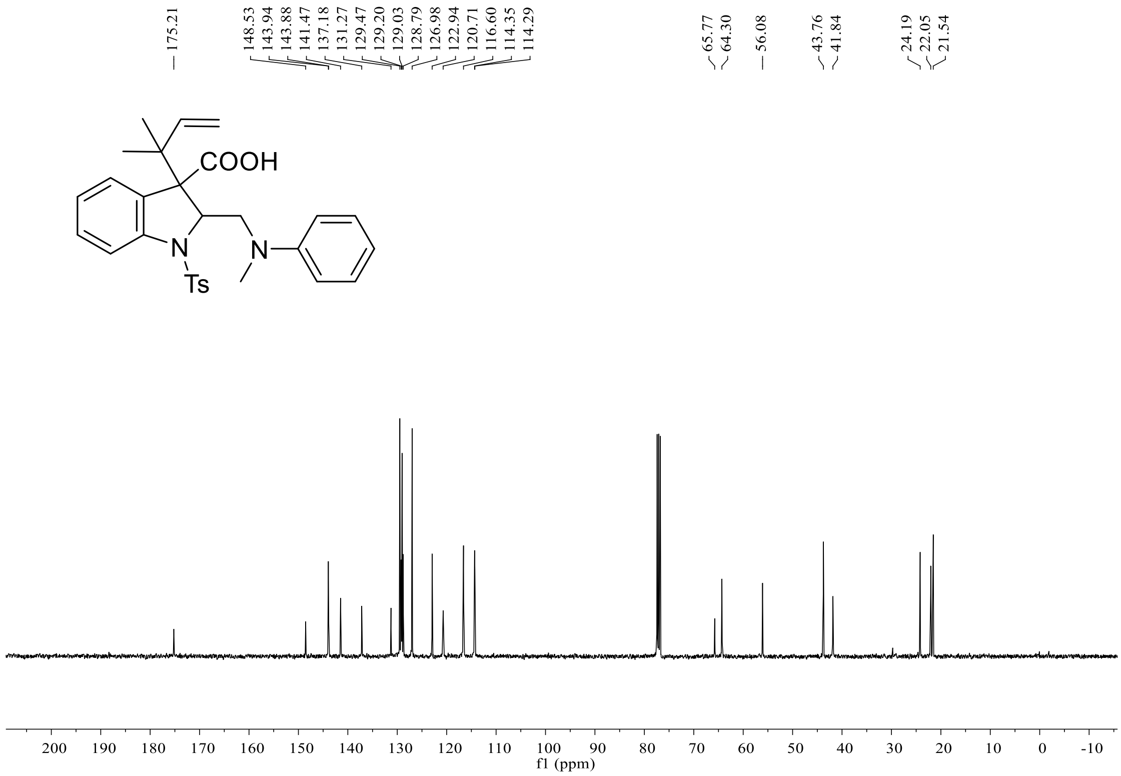

Supplementary Fig. 66. ^{13}C NMR of compound **3d** (100 MHZ, $CDCl_3$)

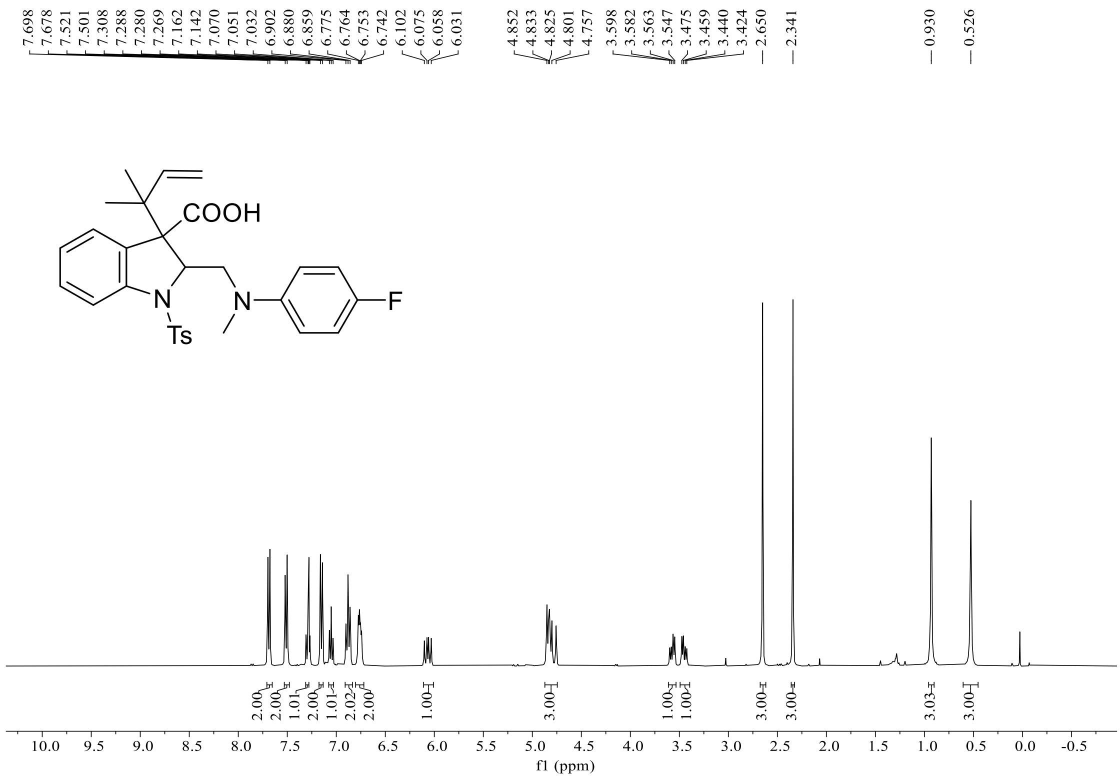

Supplementary Fig. 67. ^{1}H NMR of compound **3e** (400 MHZ, $CDCl_3$)

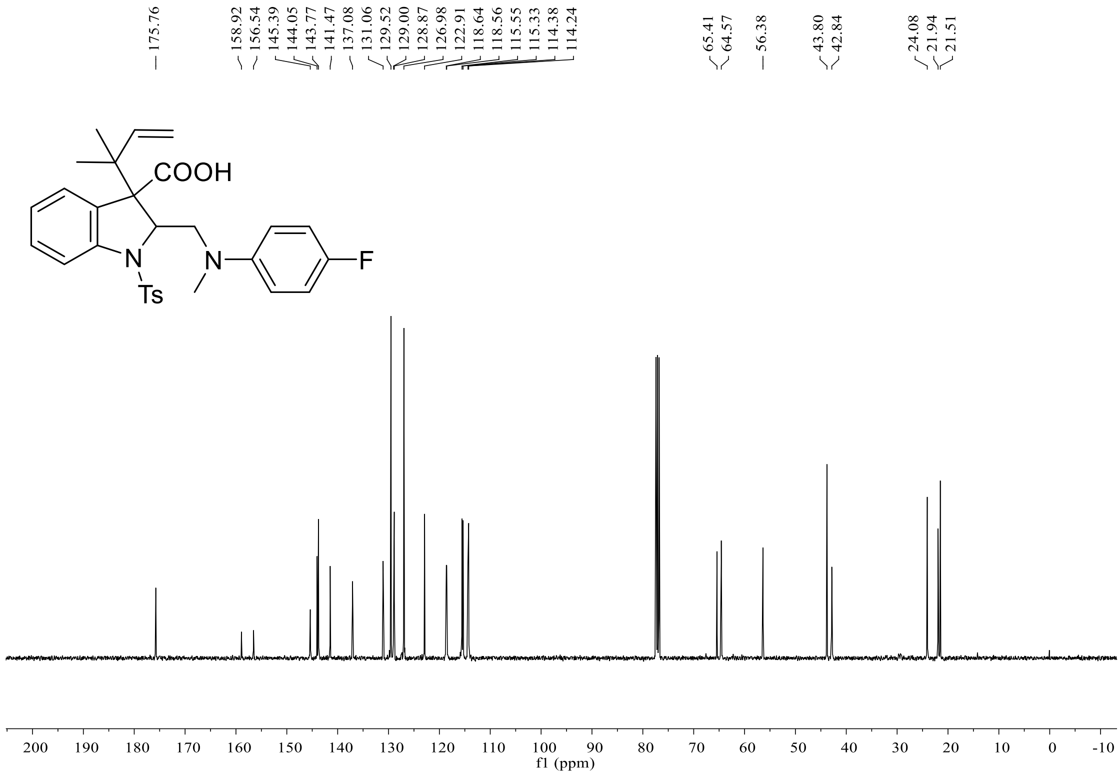

Supplementary Fig. 68. ^{13}C NMR of compound **3e** (100 MHZ, $CDCl_3$)

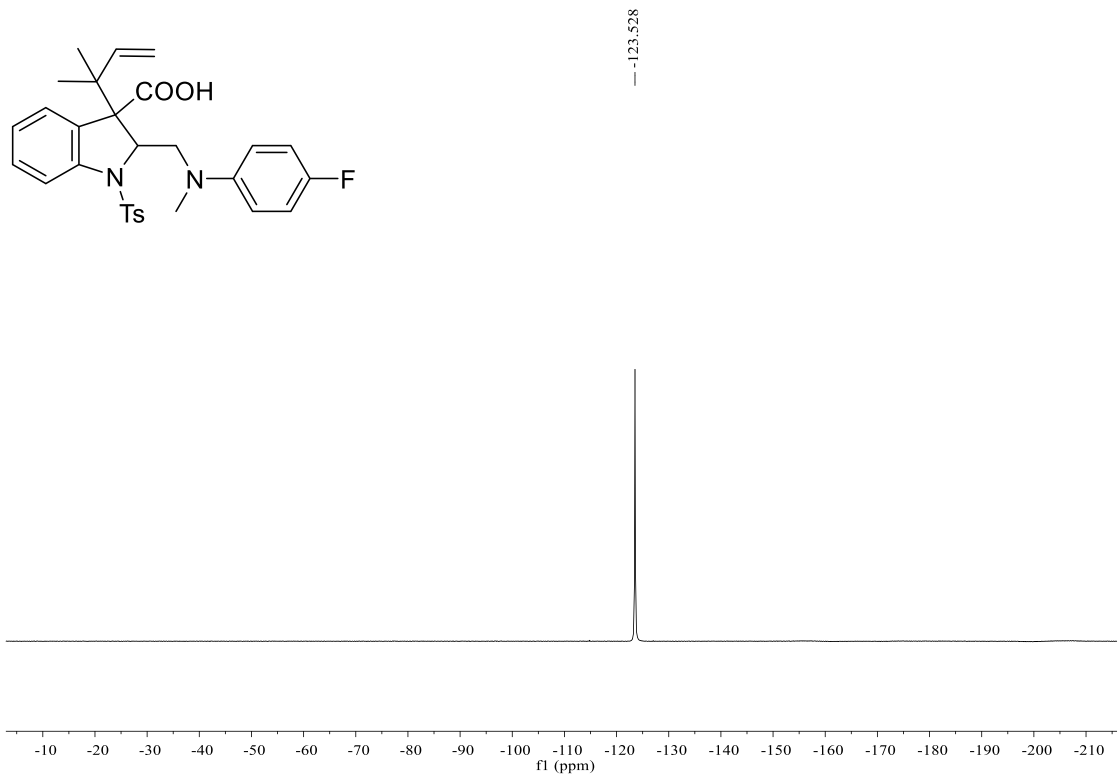

Supplementary Fig. 69. ^{19}F NMR of compound **3e** (376 MHZ, $CDCl_3$)

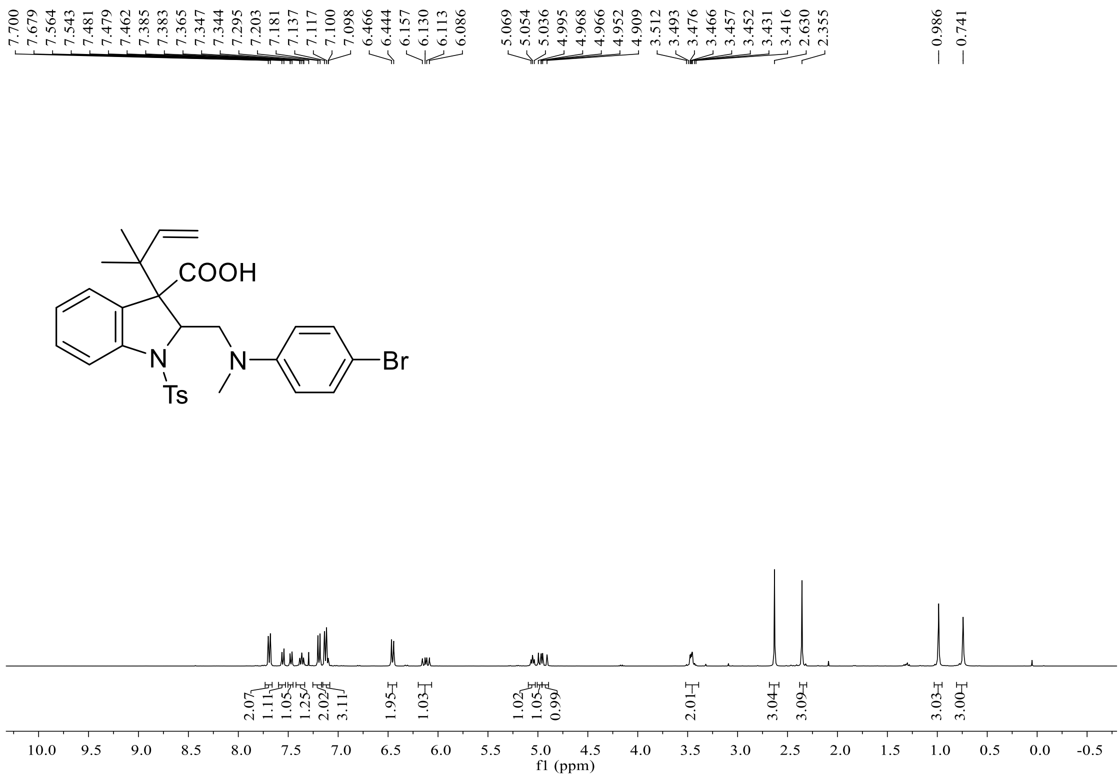

Supplementary Fig. 70. ^{1}H NMR of compound **3f** (400 MHZ, $CDCl_3$)

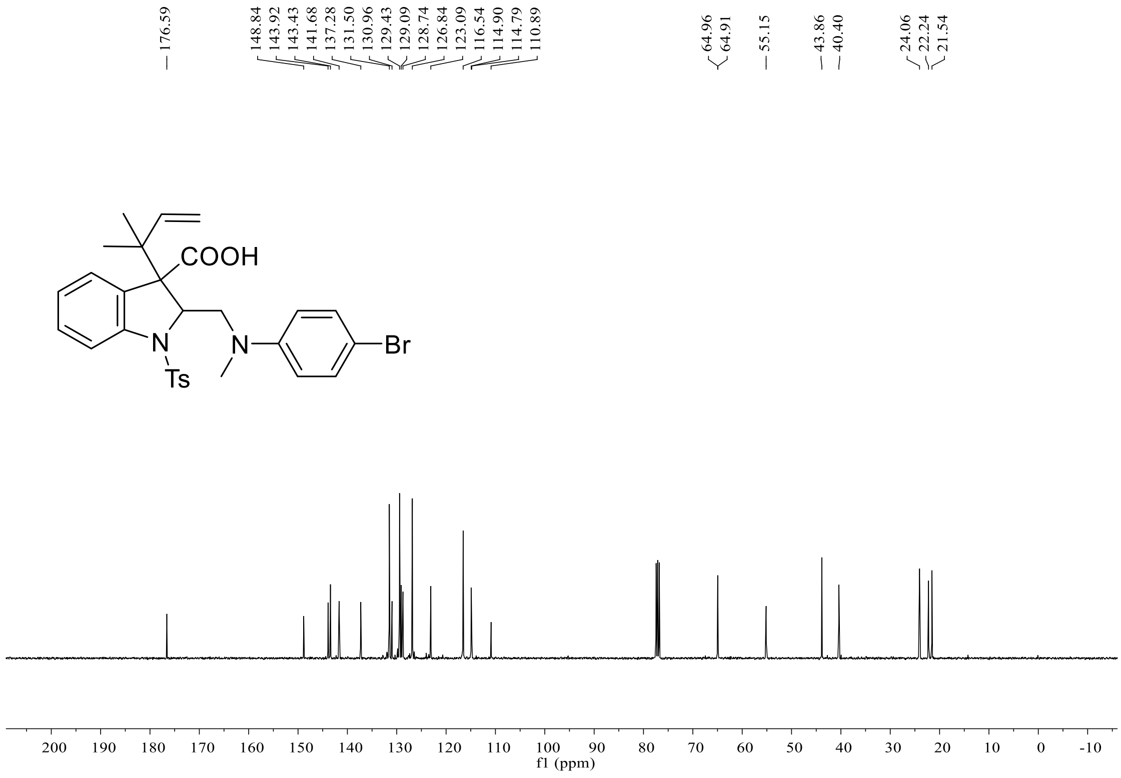

Supplementary Fig. 71. ^{13}C NMR of compound **3f** (100 MHZ, $CDCl_3$)

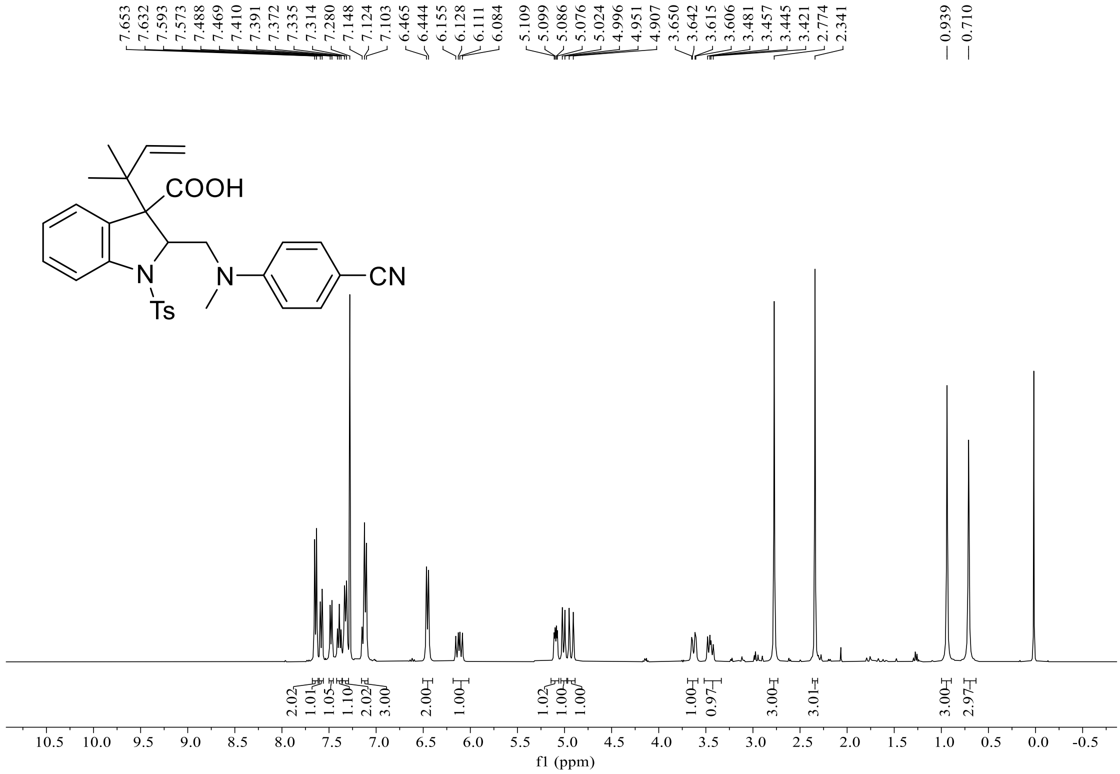

Supplementary Fig. 72. ^{1}H NMR of compound **3g** (400 MHZ, $CDCl_3$)

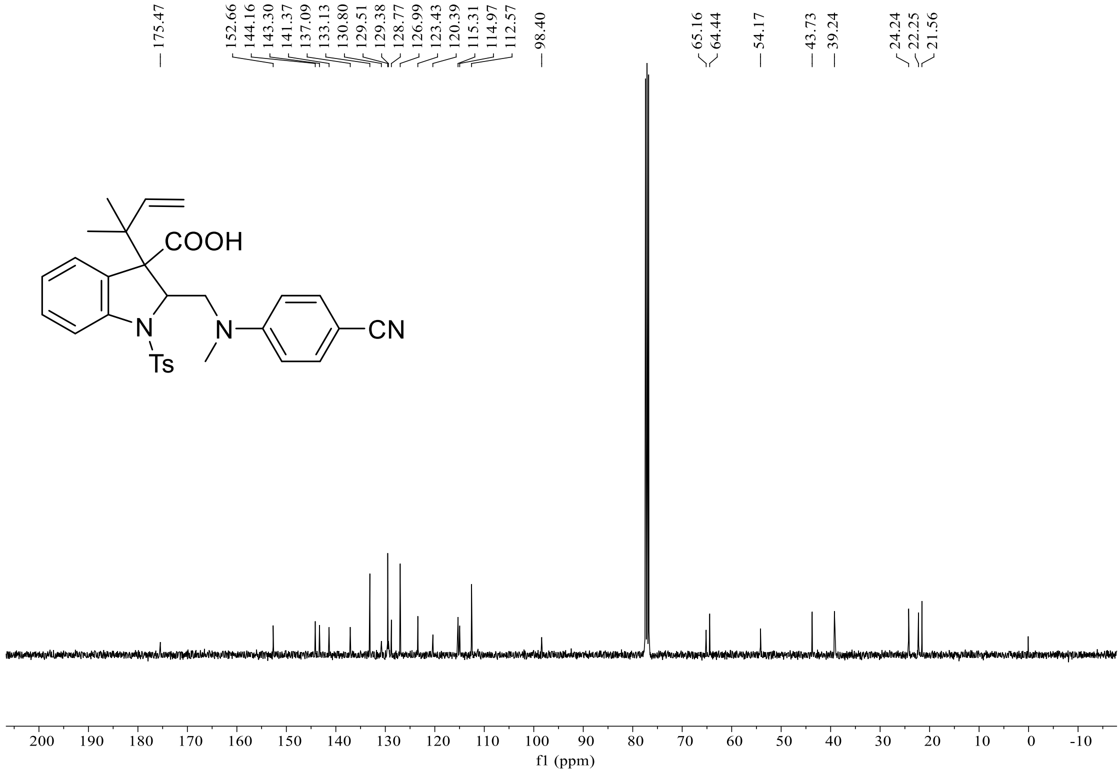

Supplementary Fig. 73. ^{13}C NMR of compound **3g** (100 MHZ, $CDCl_3$)

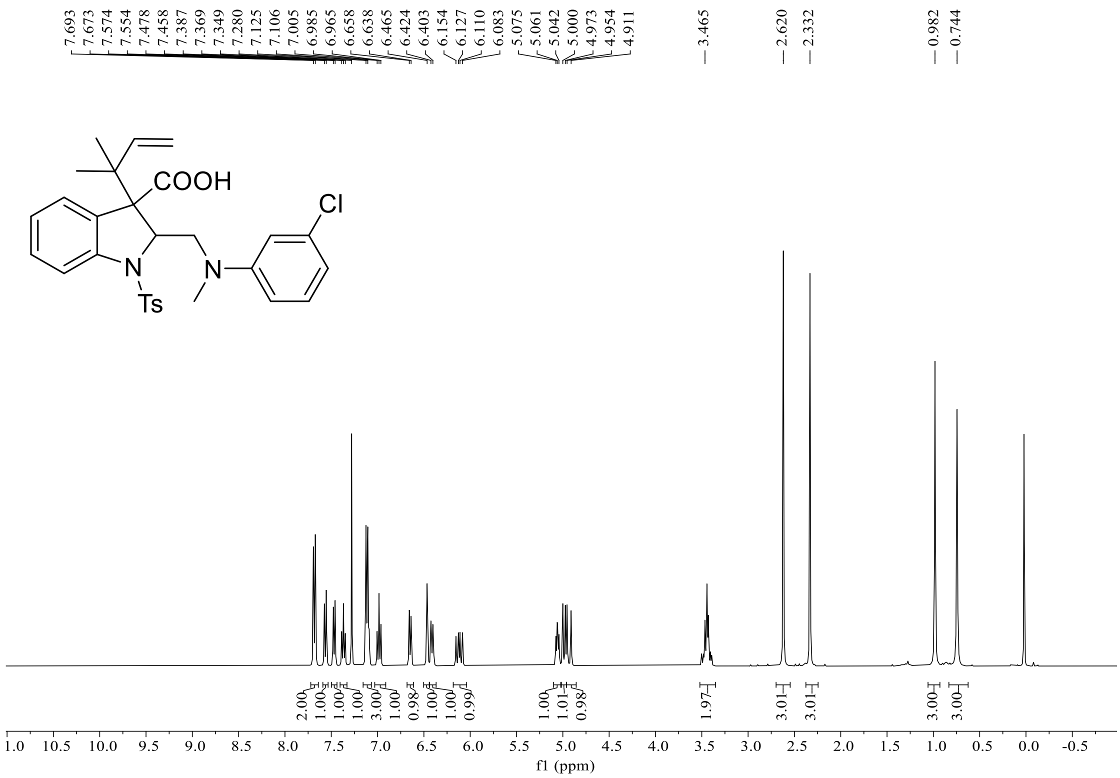

Supplementary Fig. 74. ^{1}H NMR of compound **3h** (400 MHZ, $CDCl_3$)

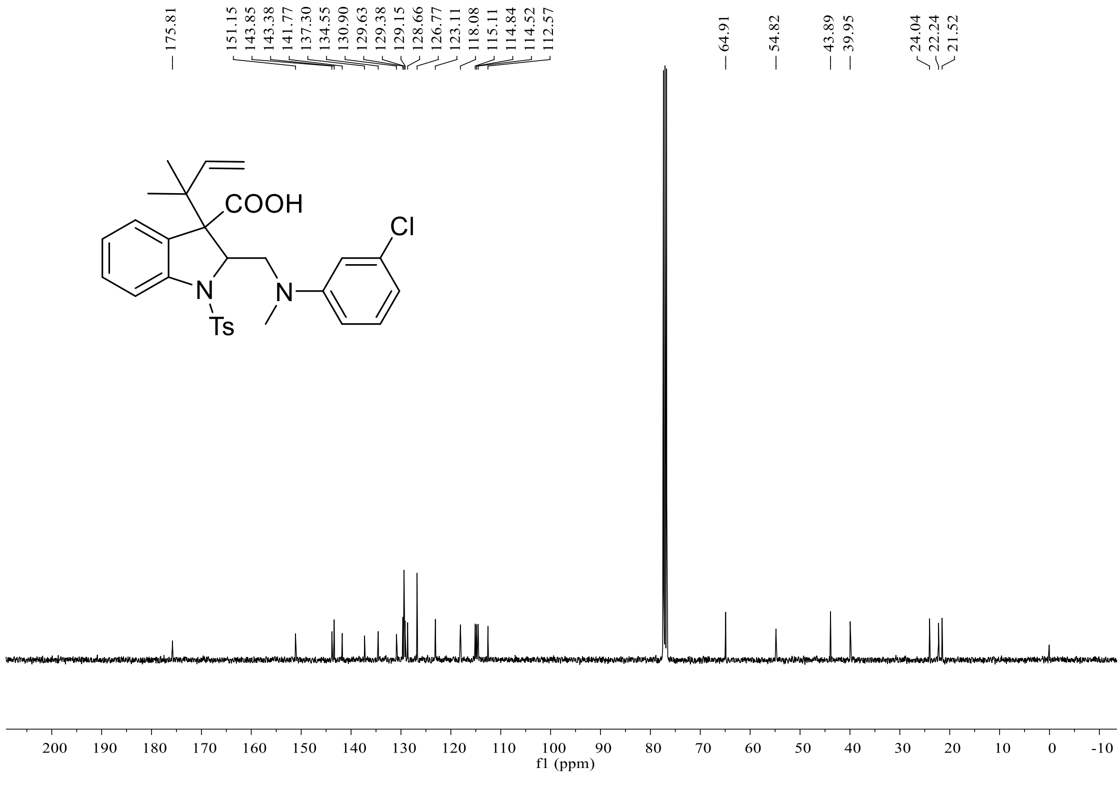

Supplementary Fig. 75. ^{13}C NMR of compound **3h** (100 MHZ, $CDCl_3$)

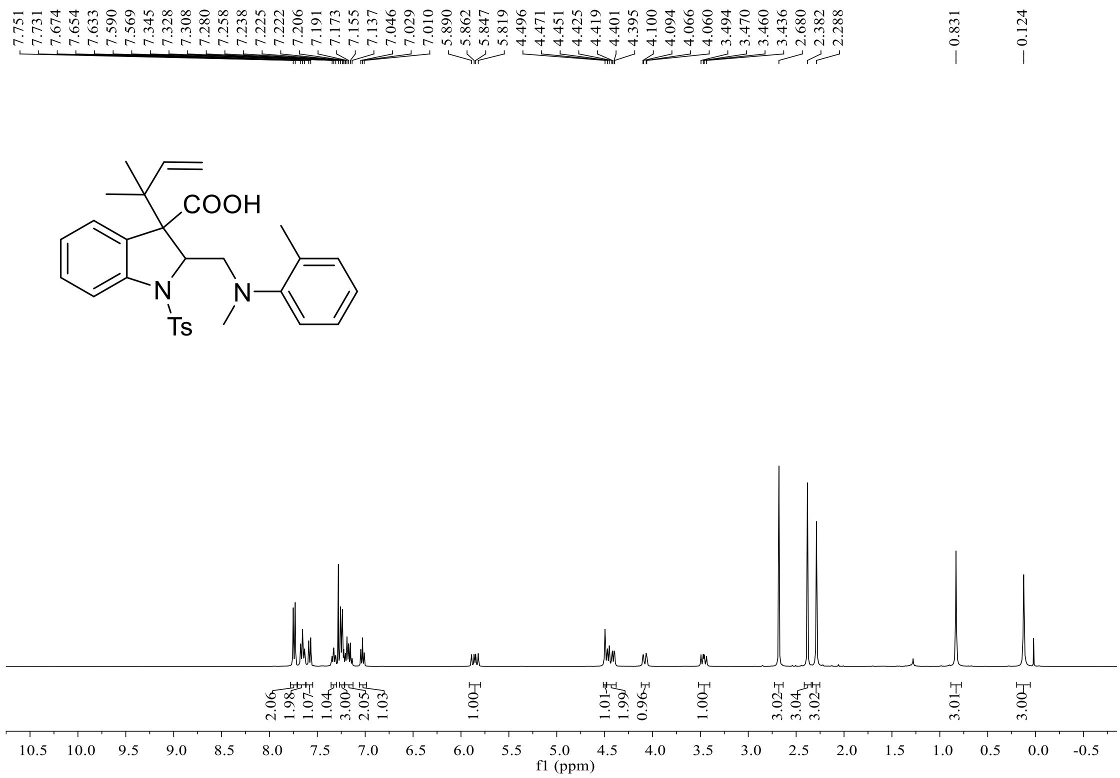

Supplementary Fig. 76. ^{1}H NMR of compound **3i** (400 MHZ, $CDCl_3$)

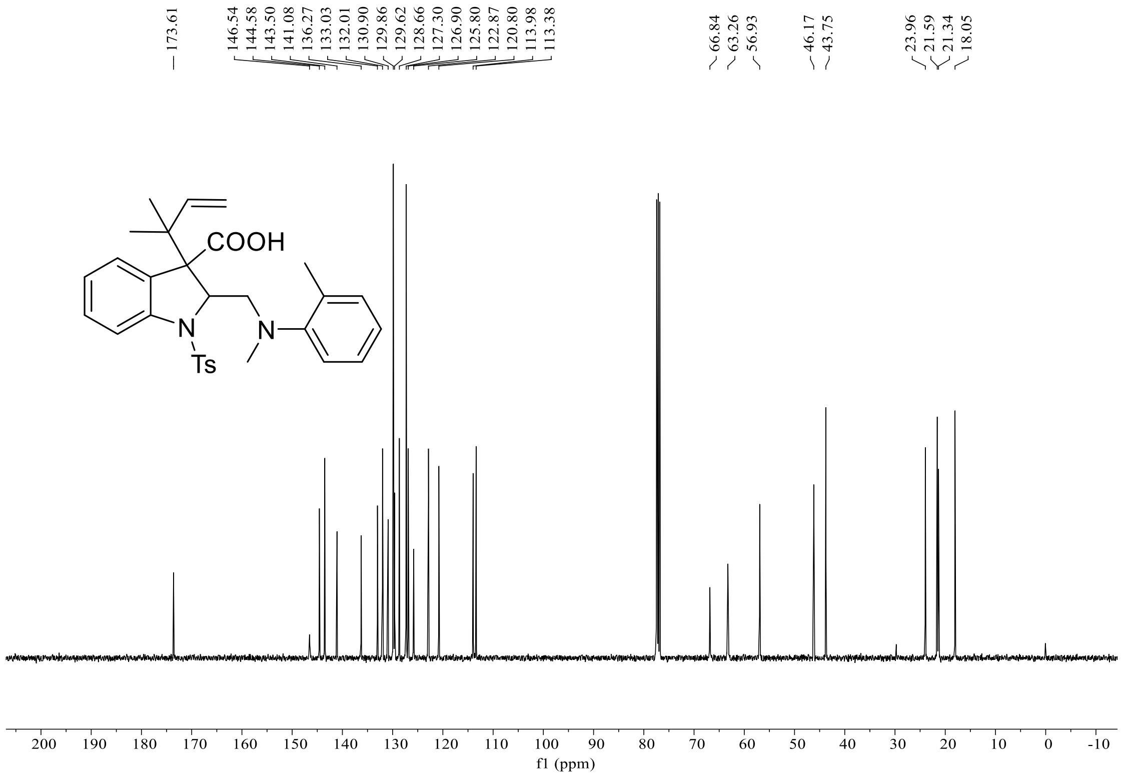

Supplementary Fig. 77. ^{13}C NMR of compound **3i** (100 MHZ, $CDCl_3$)

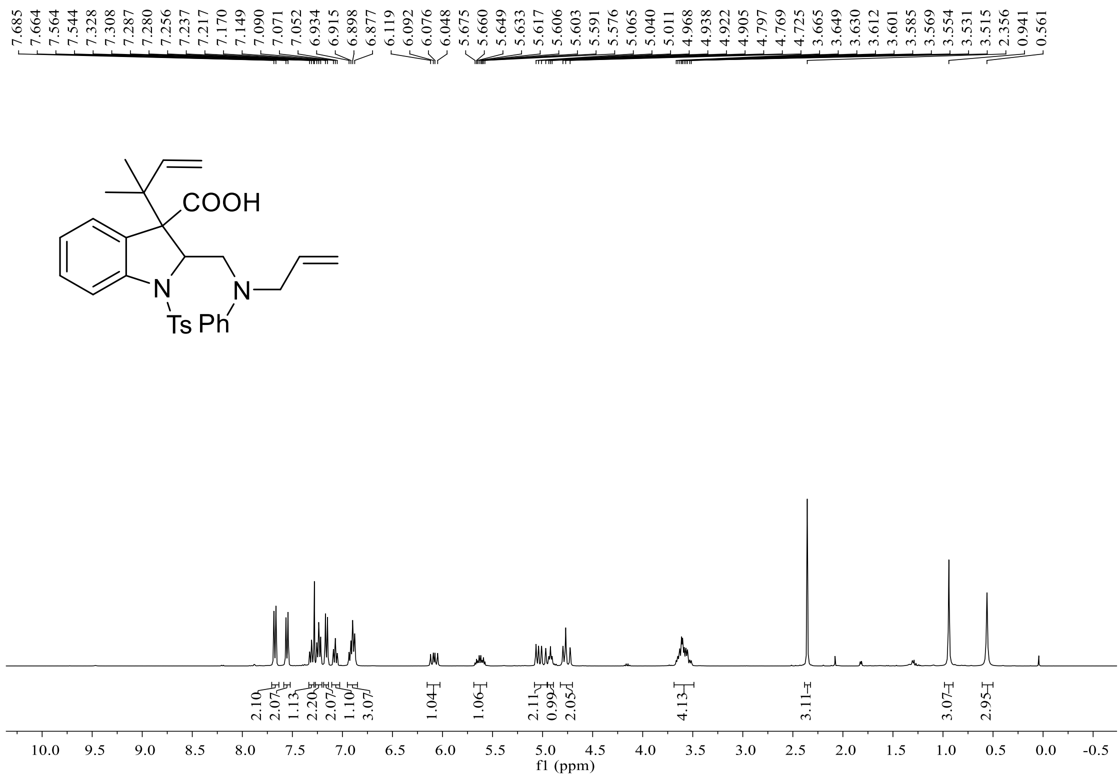

Supplementary Fig. 78. ^{1}H NMR of compound **3j** (400 MHZ, $CDCl_3$)

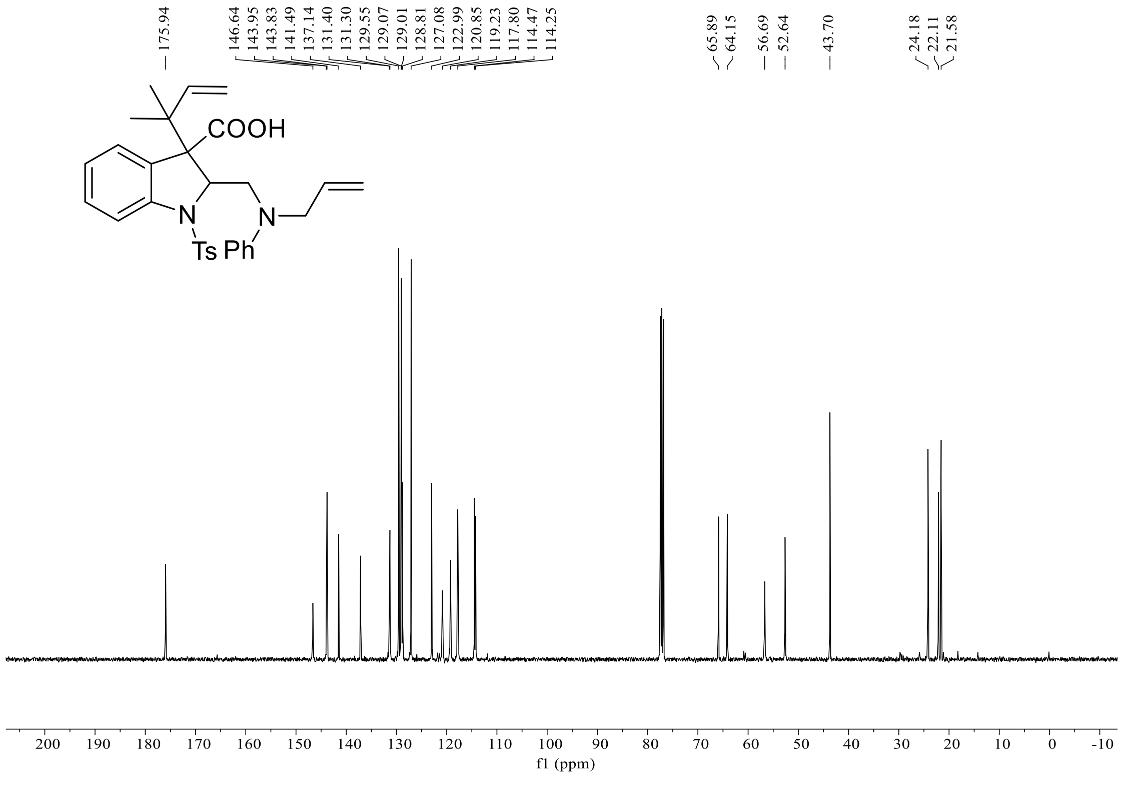

Supplementary Fig. 79. ^{13}C NMR of compound **3j** (100 MHZ, $CDCl_3$)

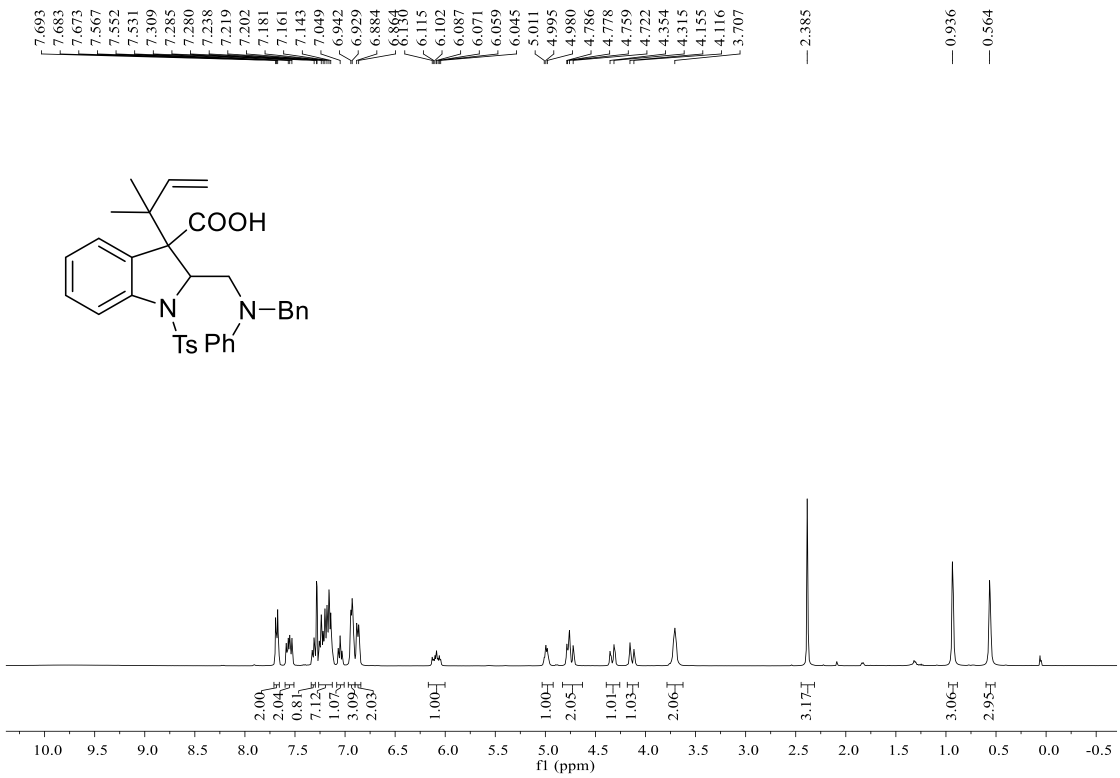

Supplementary Fig. 80. ^{1}H NMR of compound **3k** (400 MHZ, $CDCl_3$)

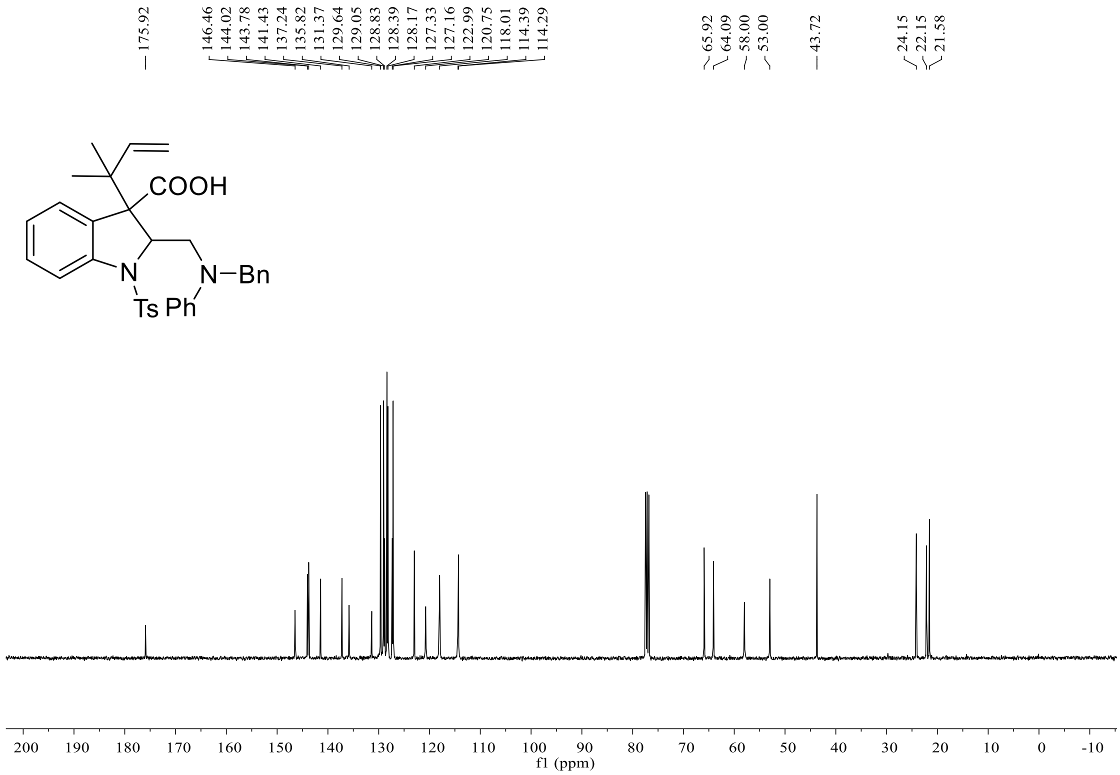

Supplementary Fig. 81. ^{13}C NMR of compound **3k** (100 MHZ, $CDCl_3$)

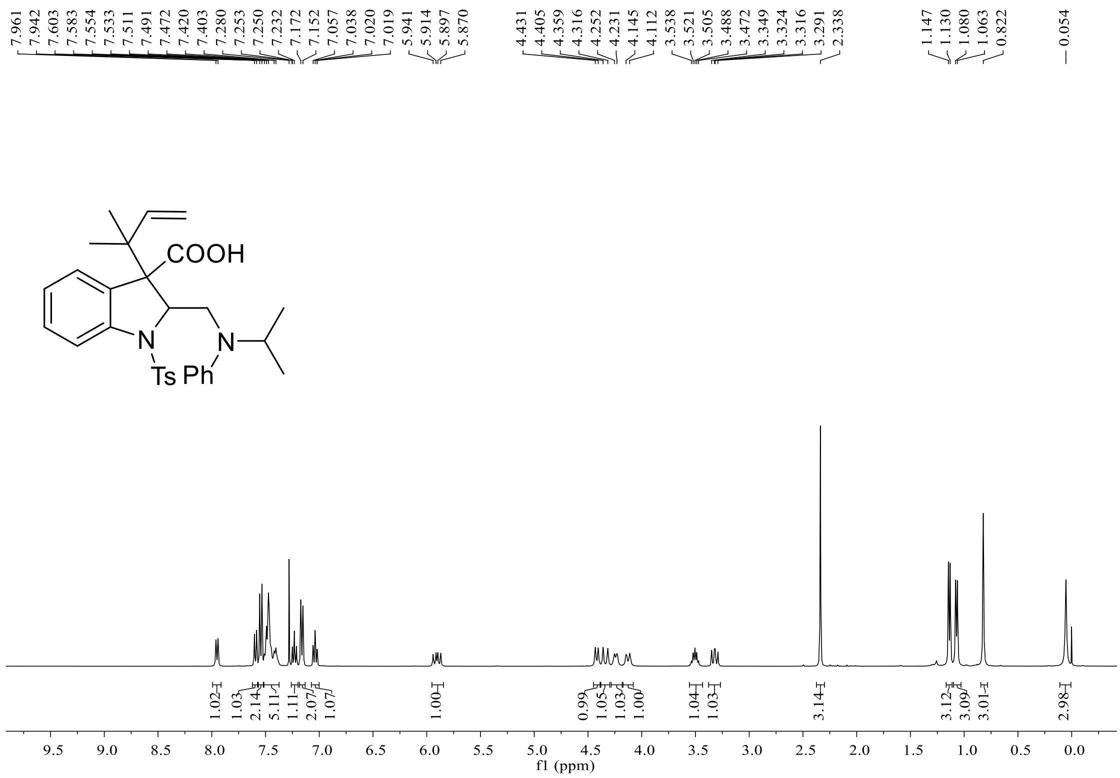

Supplementary Fig. 82. ^{1}H NMR of compound **3l** (400 MHZ, $CDCl_3$)

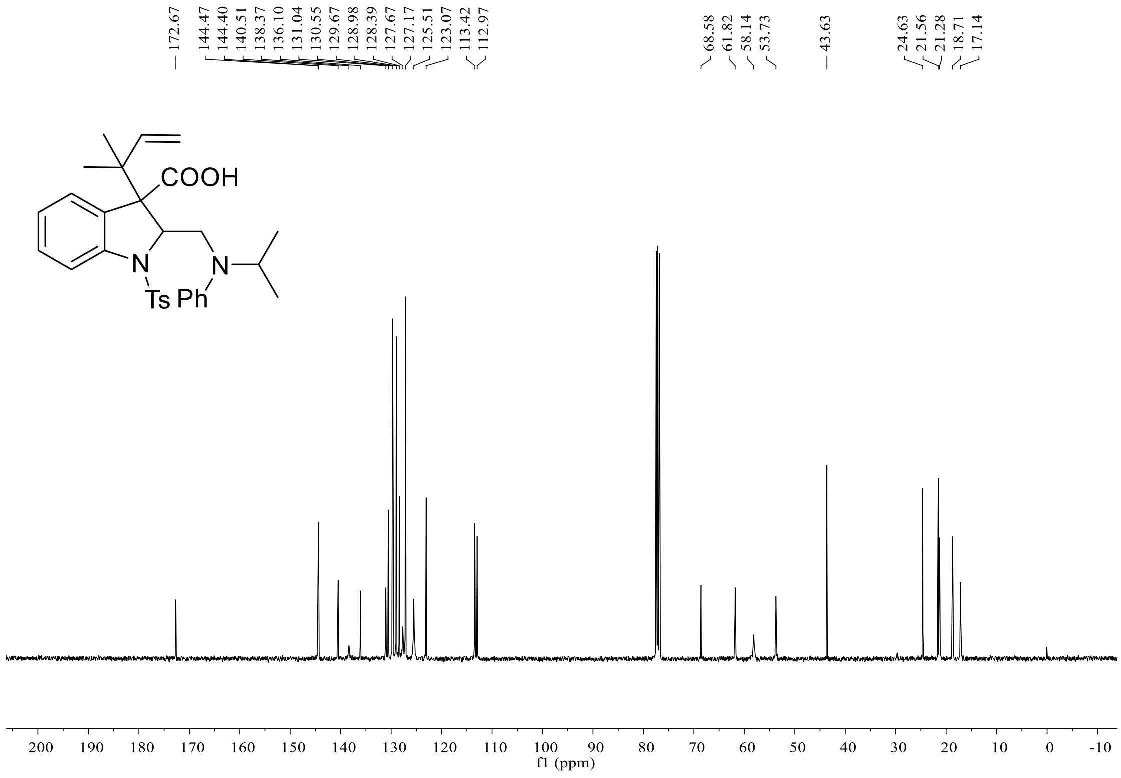

Supplementary Fig. 83. ^{13}C NMR of compound **3l** (100 MHZ, $CDCl_3$)

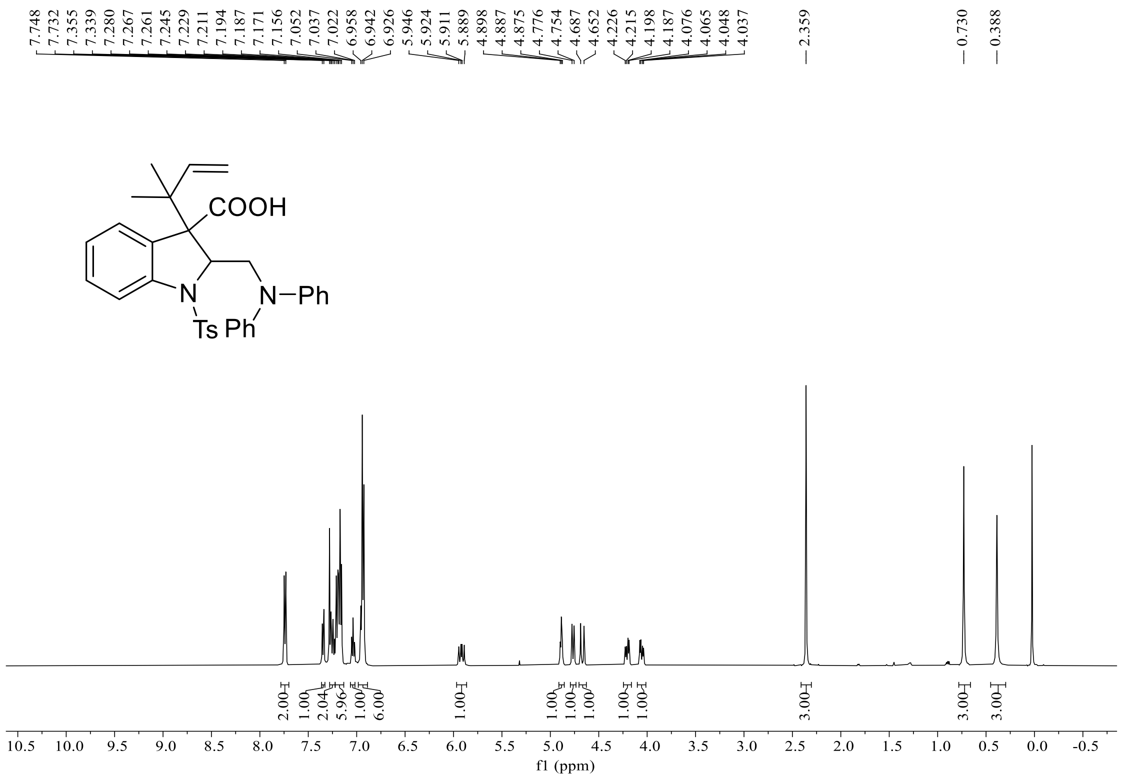

Supplementary Fig. 84. ^{1}H NMR of compound **3m** (500 MHZ, $CDCl_3$)

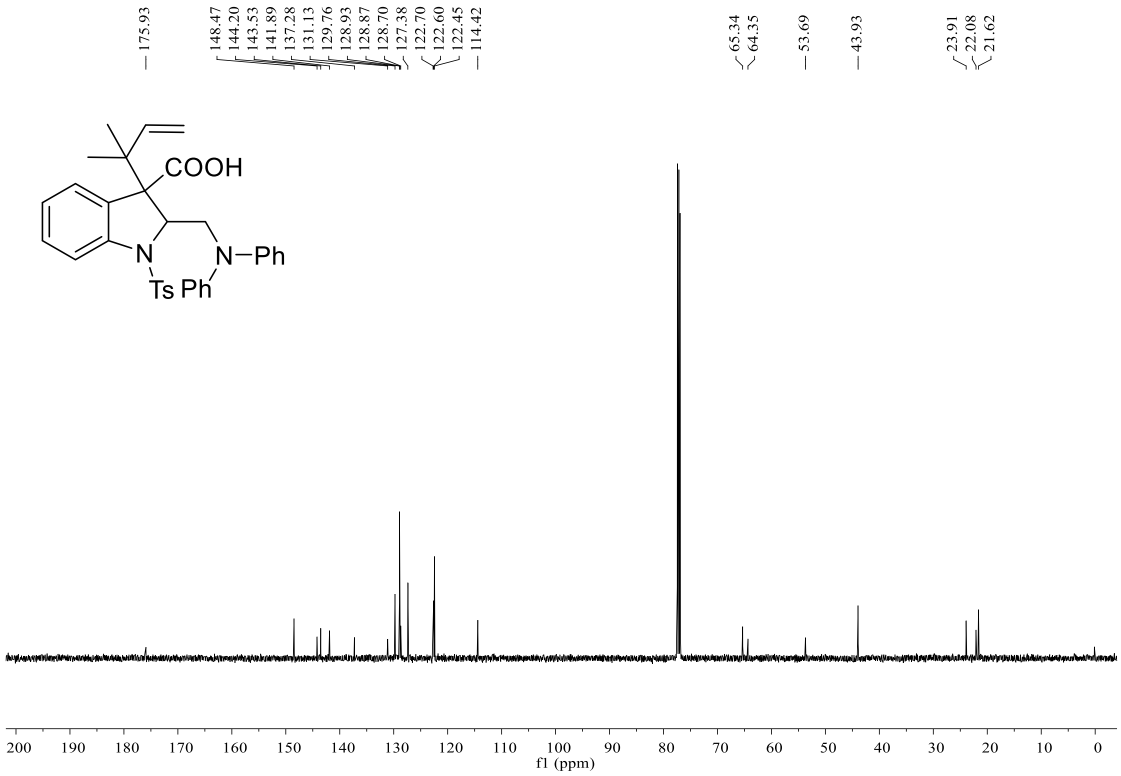

Supplementary Fig. 85. ^{13}C NMR of compound **3m** (125 MHZ, $CDCl_3$)

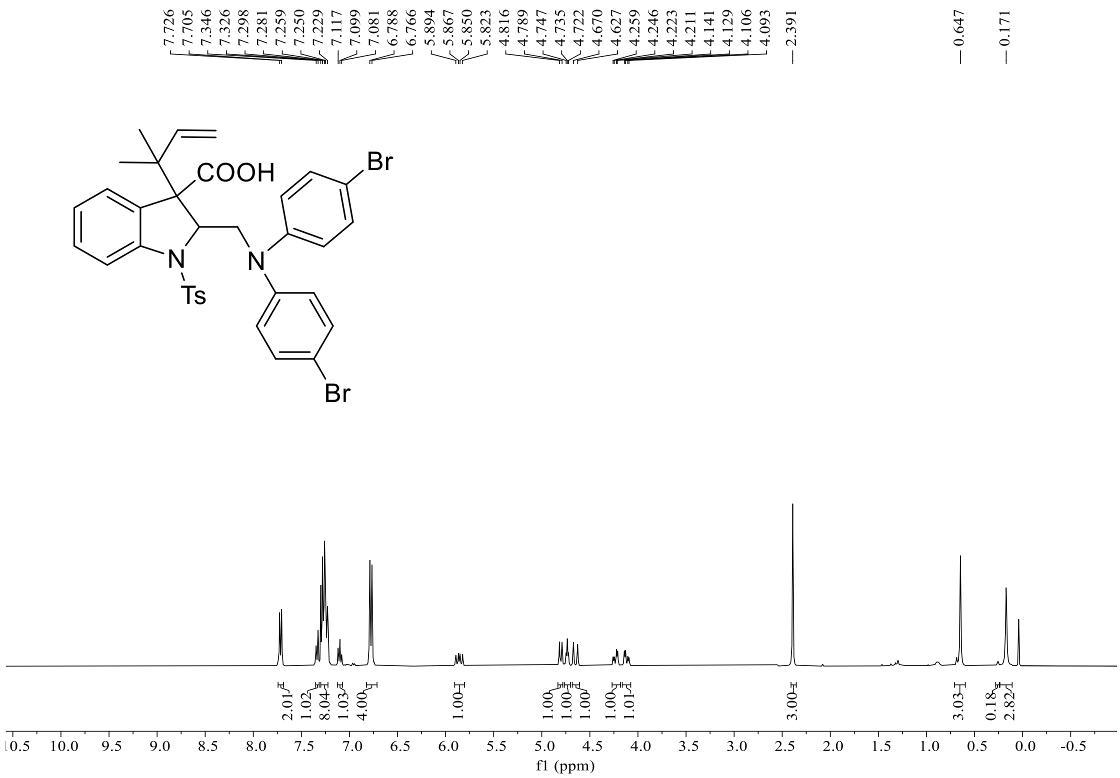

Supplementary Fig. 86. ¹H NMR of compound **3n** (400 MHZ, $CDCl_3$)

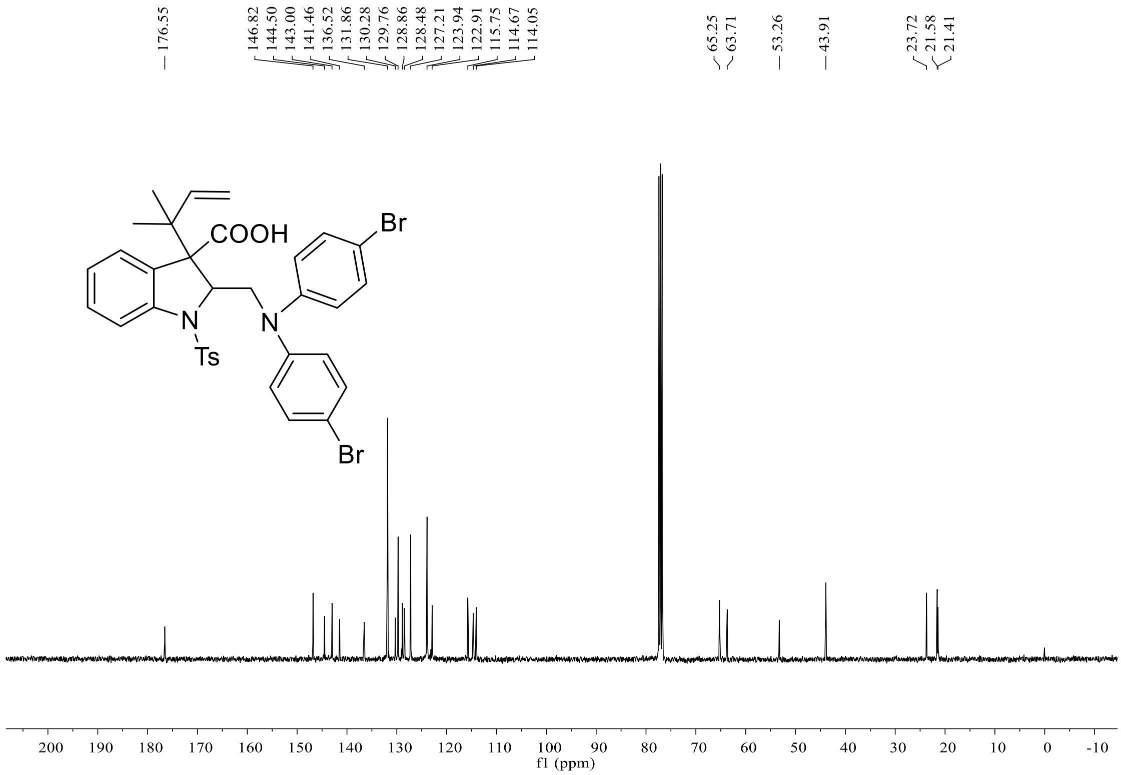

Supplementary Fig. 87. ¹³C NMR of compound **3n** (100 MHZ, $CDCl_3$)

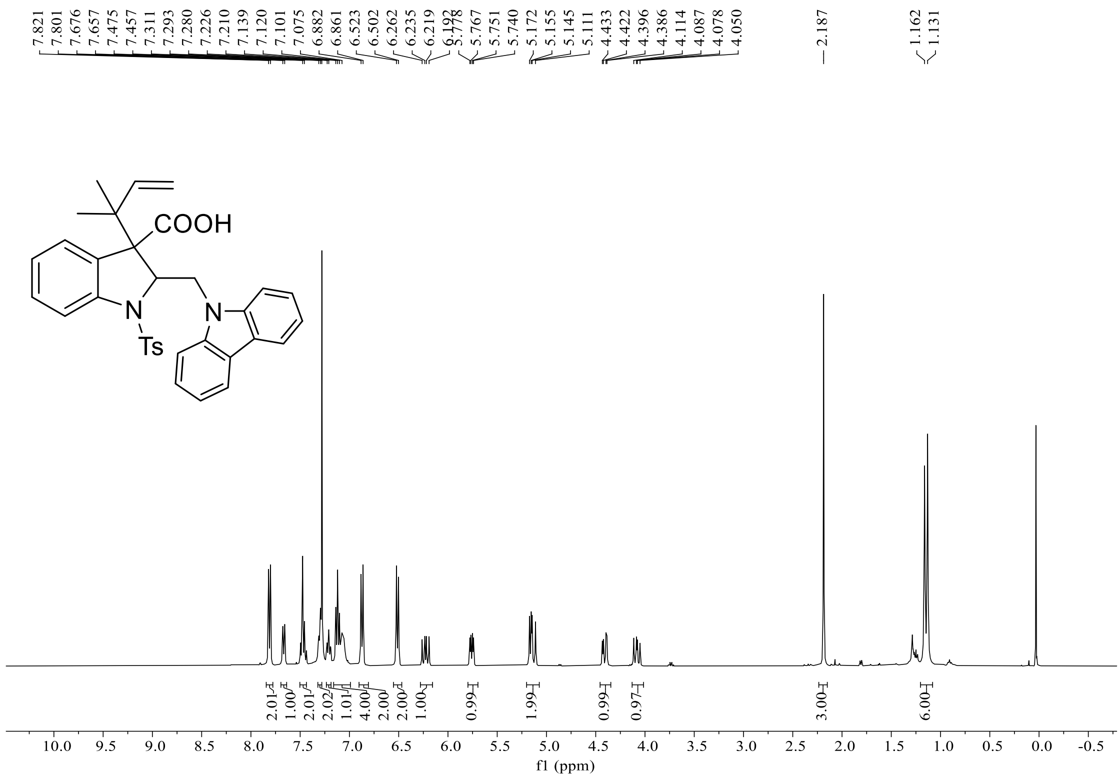

Supplementary Fig. 88. ^{1}H NMR of compound **3o** (400 MHZ, $CDCl_3$)

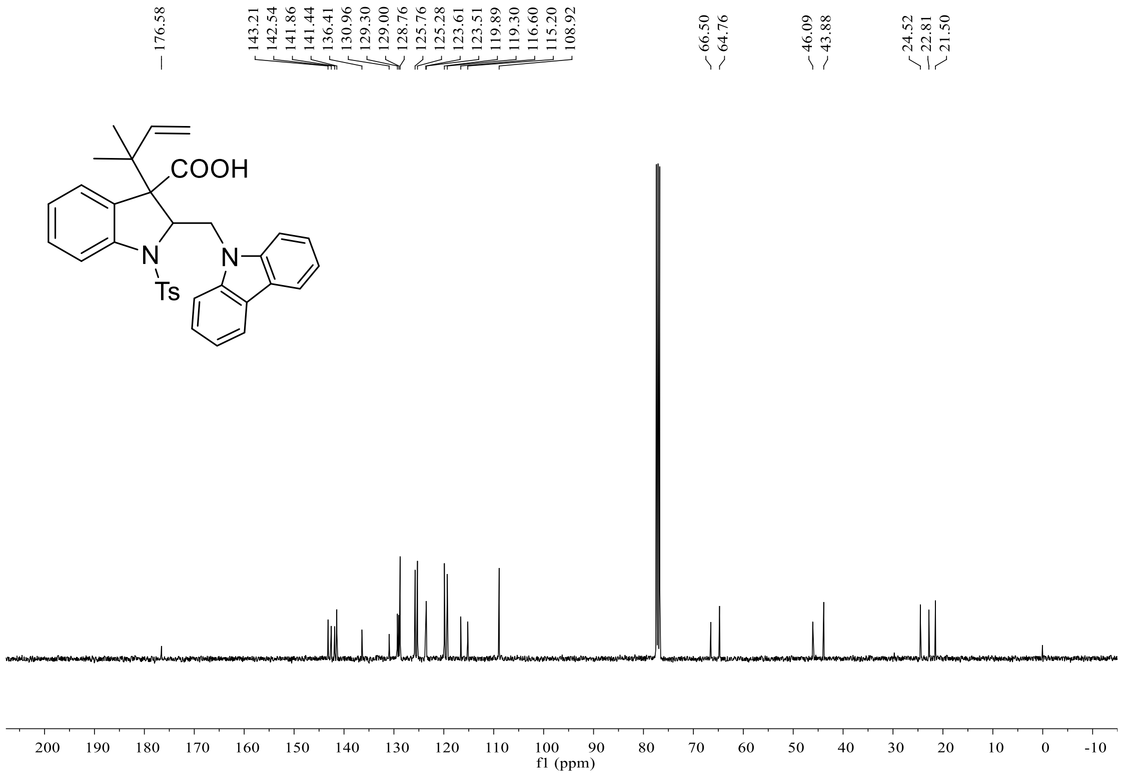

Supplementary Fig. 89. ^{13}C NMR of compound **3o** (100 MHZ, $CDCl_3$)

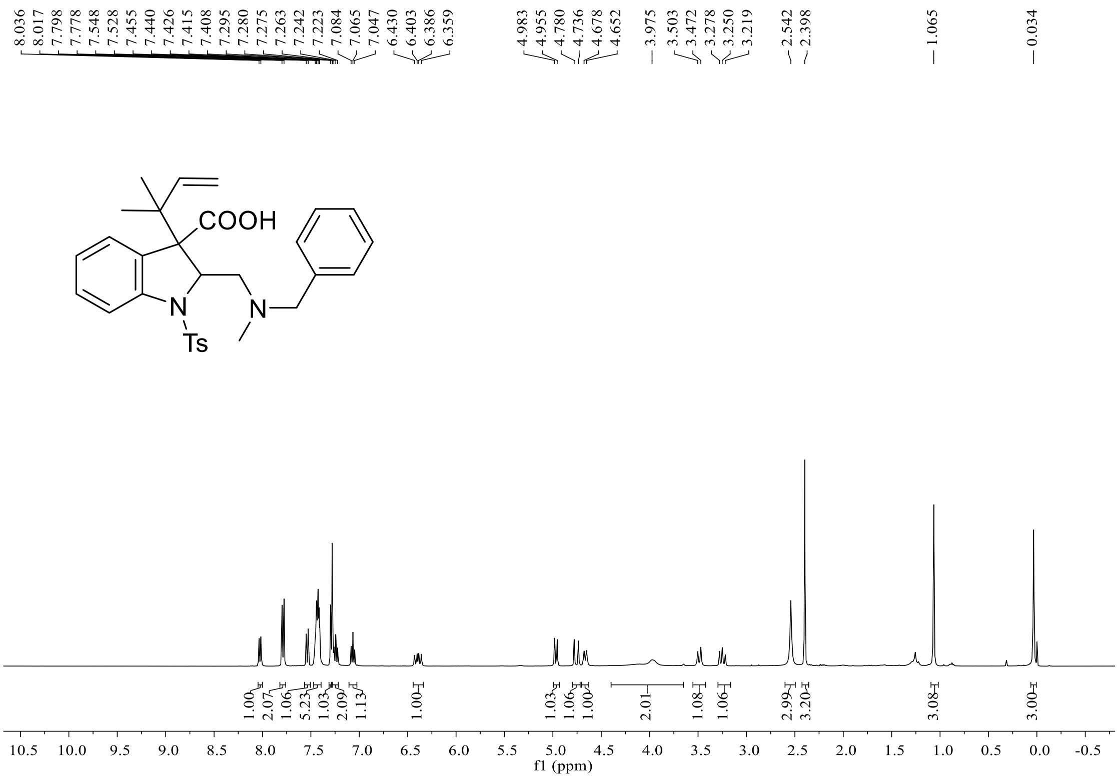

Supplementary Fig. 90. ^{1}H NMR of compound **3p** (400 MHZ, $CDCl_3$)

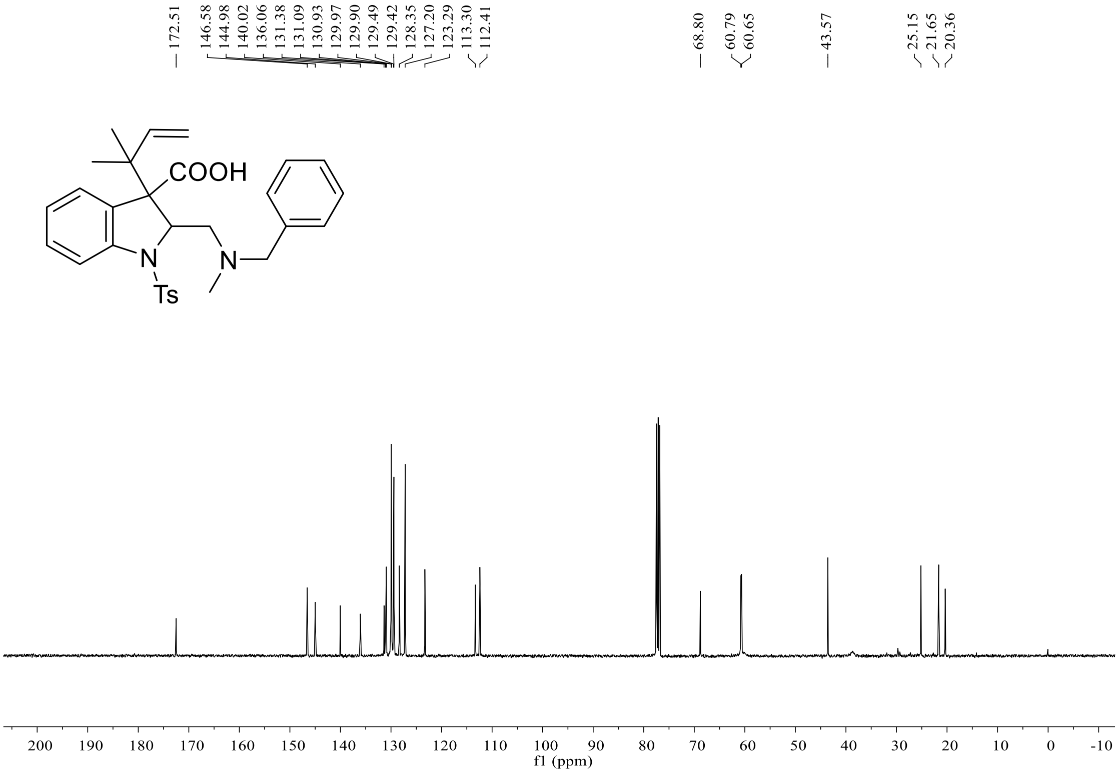

Supplementary Fig. 91. ^{13}C NMR of compound **3p** (100 MHZ, $CDCl_3$)

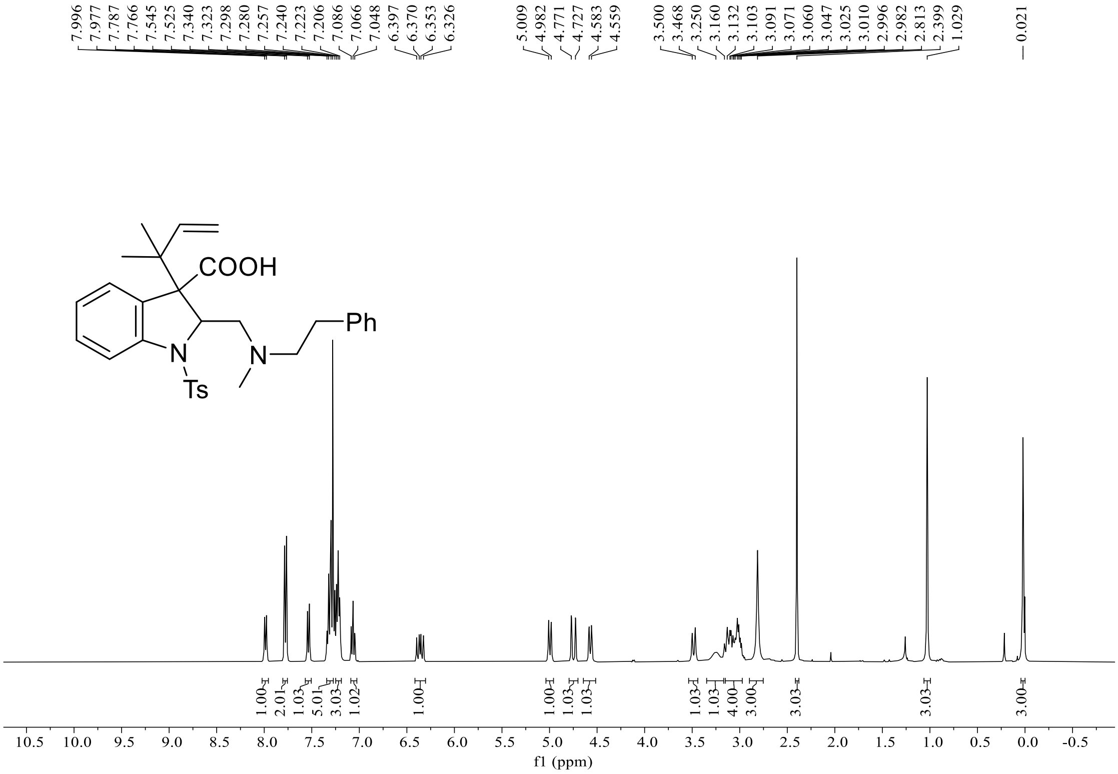

Supplementary Fig. 92. ^{1}H NMR of compound **3q** (400 MHZ, $CDCl_3$)

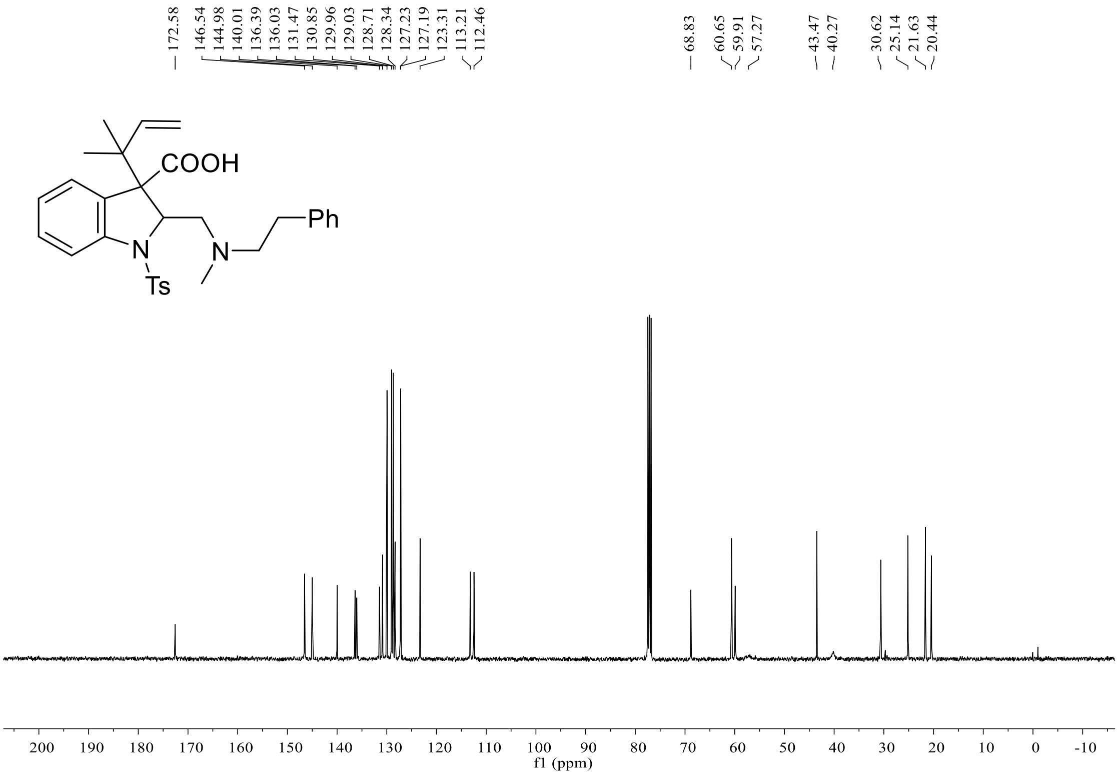

Supplementary Fig. 93. ^{13}C NMR of compound **3q** (100 MHZ, $CDCl_3$)

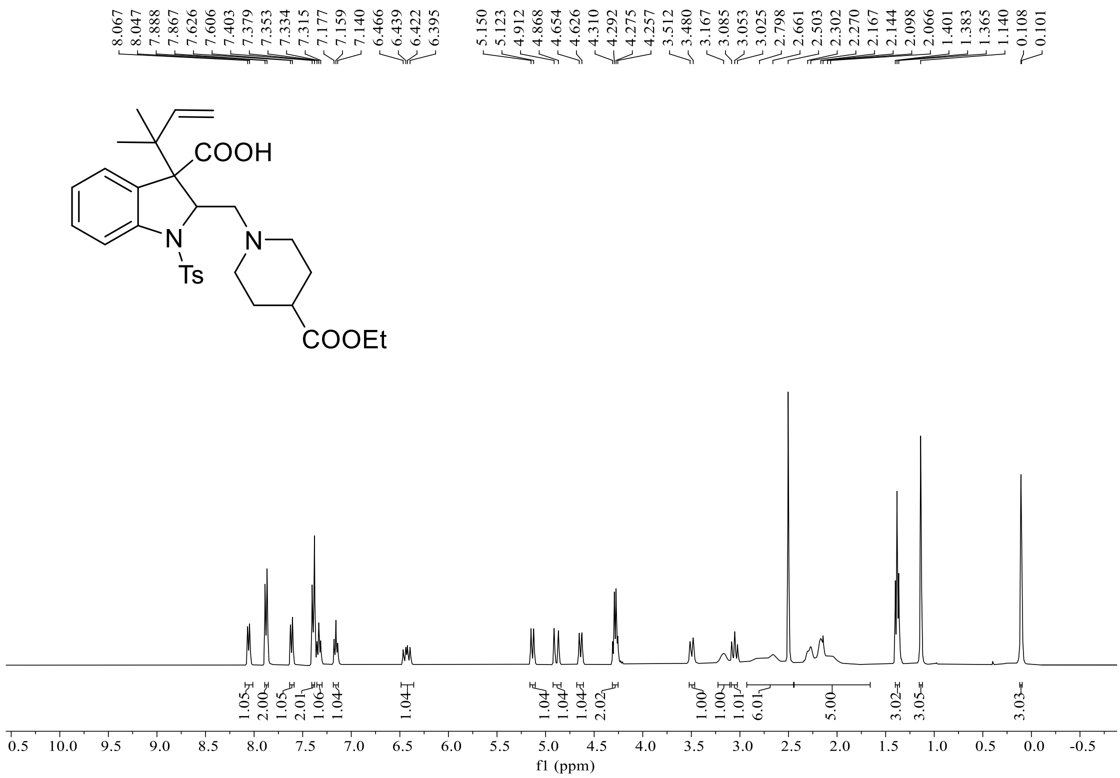

Supplementary Fig. 94. ^{1}H NMR of compound **3r** (400 MHZ, $CDCl_3$)

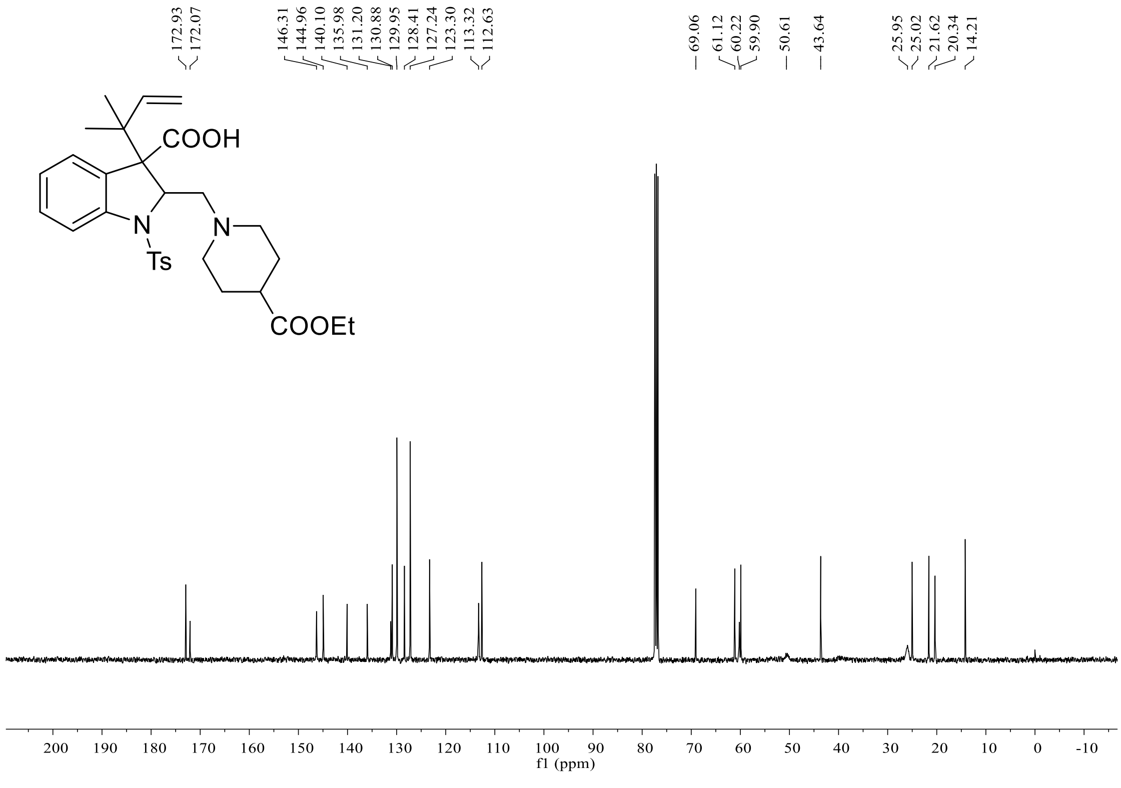

Supplementary Fig. 95. ^{13}C NMR of compound **3r** (100 MHZ, $CDCl_3$)

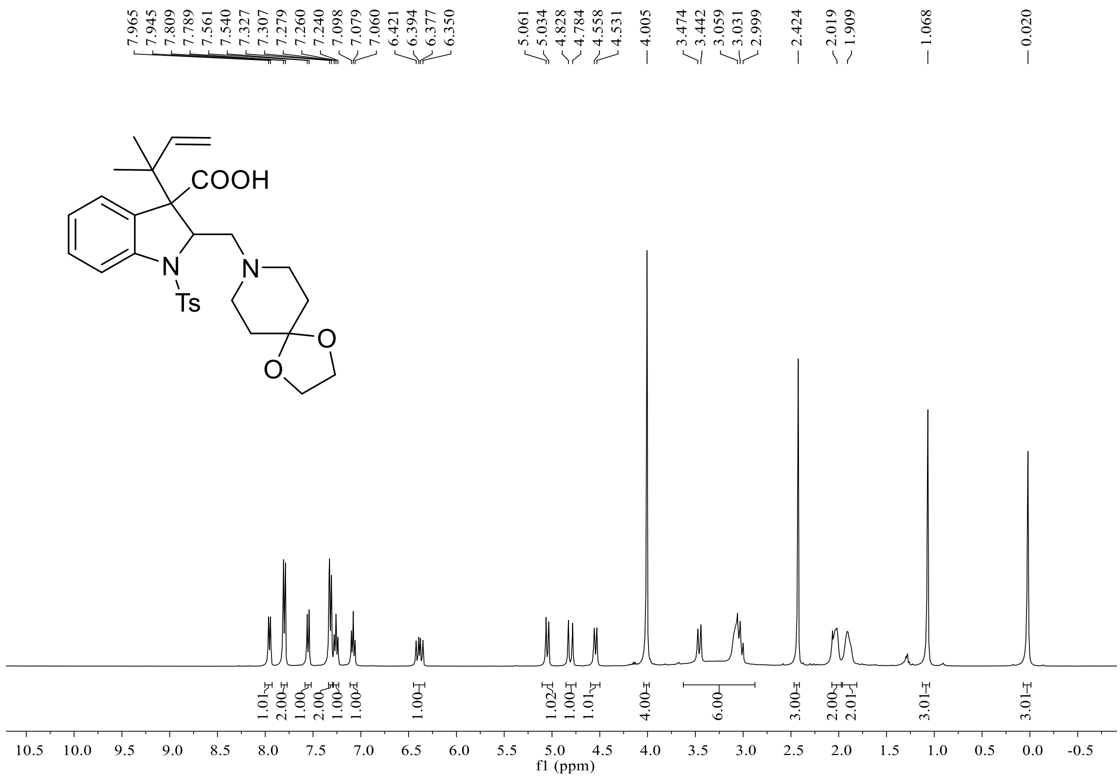

Supplementary Fig. 96. ^{1}H NMR of compound **3s** (400 MHZ, $CDCl_3$)

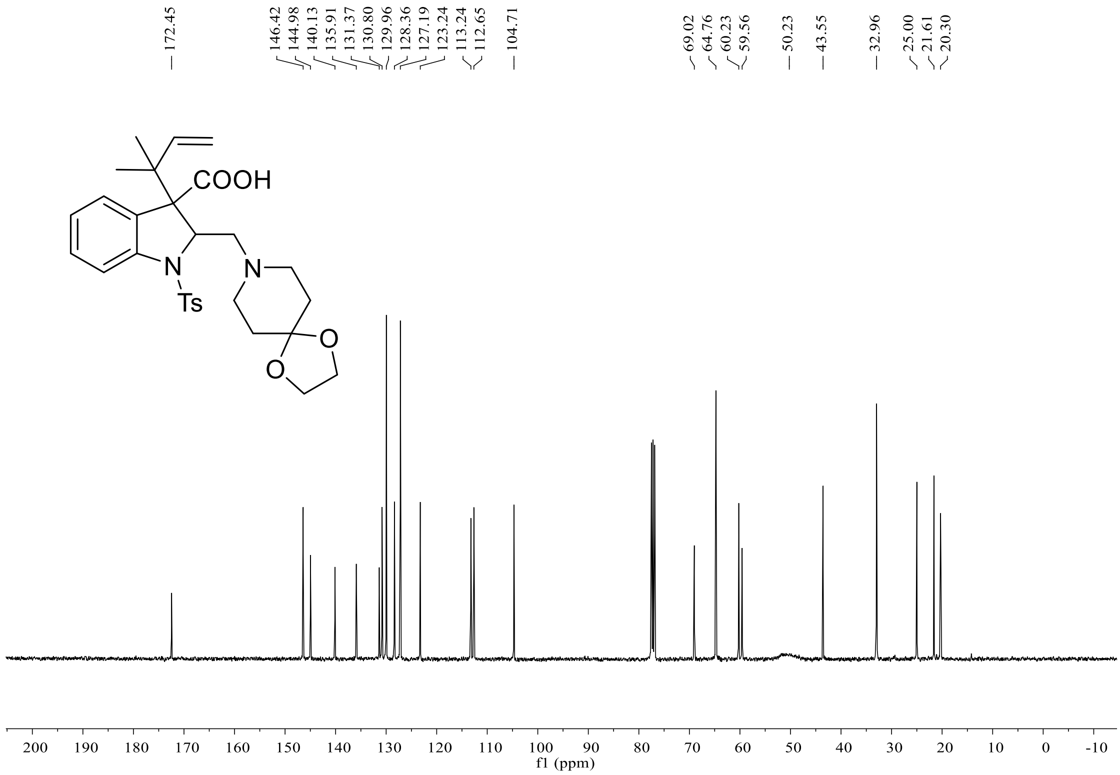

Supplementary Fig. 97. ^{13}C NMR of compound **3s** (100 MHZ, $CDCl_3$)

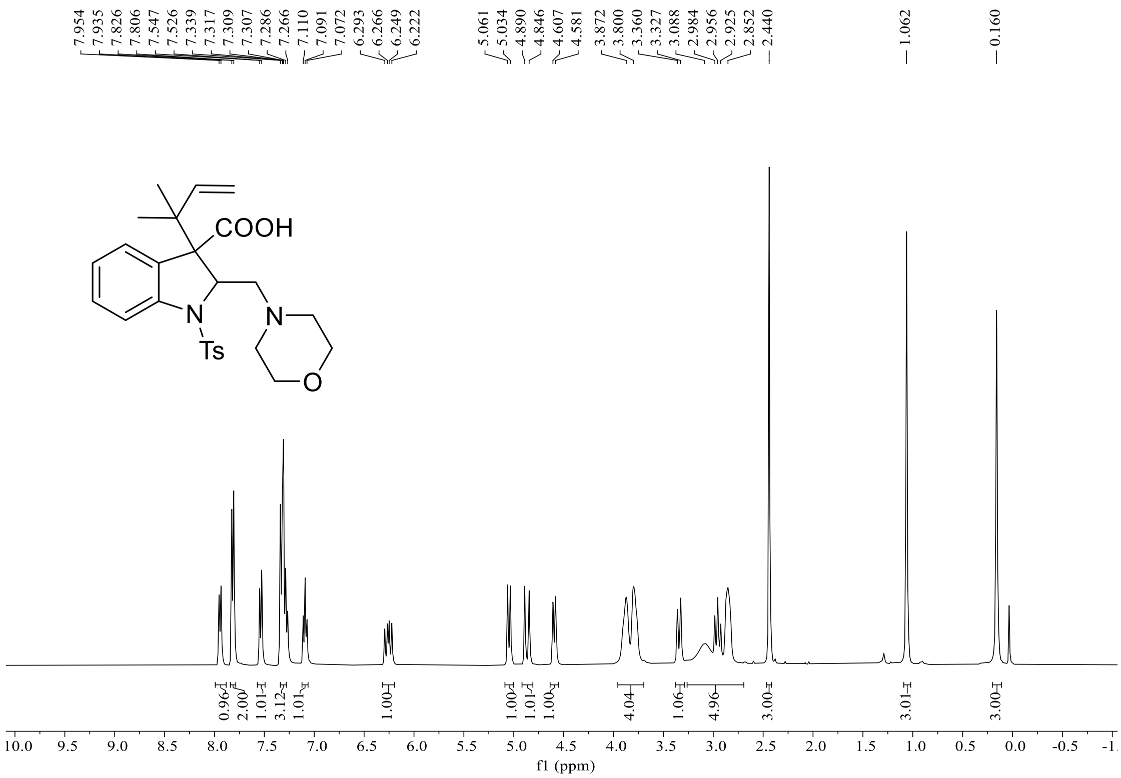

Supplementary Fig. 98. ^{1}H NMR of compound **3t** (400 MHZ, $CDCl_3$)

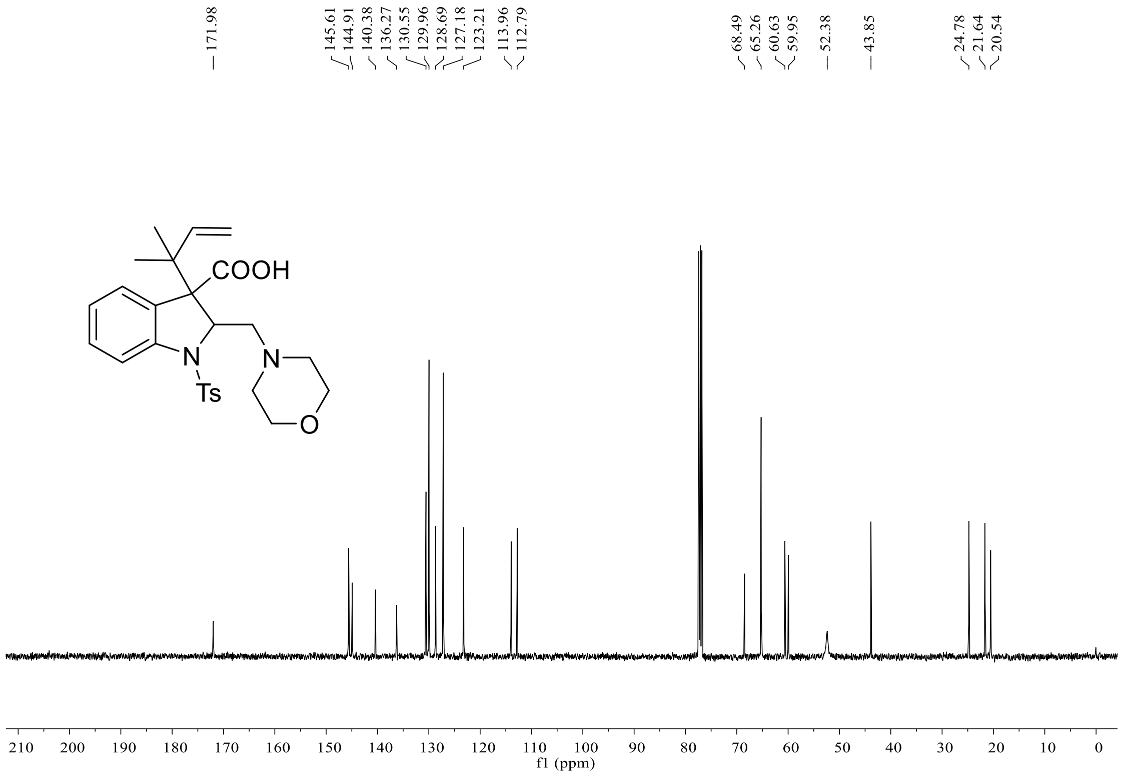

Supplementary Fig. 99. ^{13}C NMR of compound **3t** (100 MHZ, $CDCl_3$)

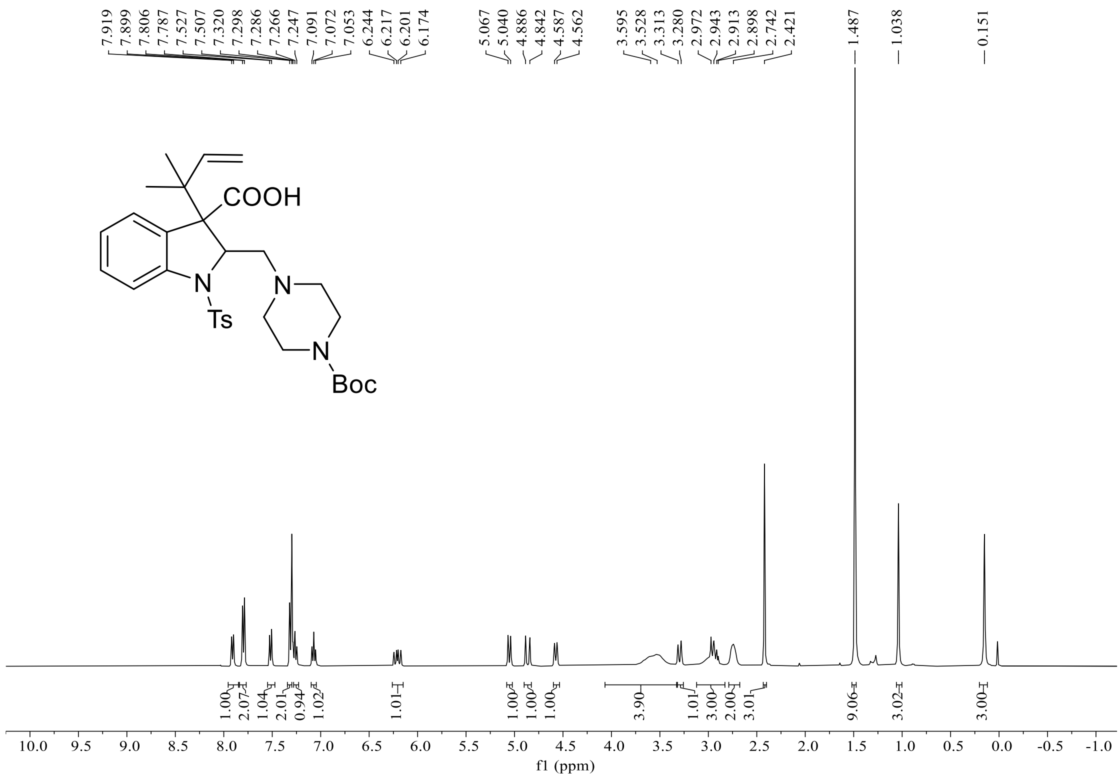

Supplementary Fig. 100. ^{1}H NMR of compound **3u** (400 MHZ, $CDCl_3$)

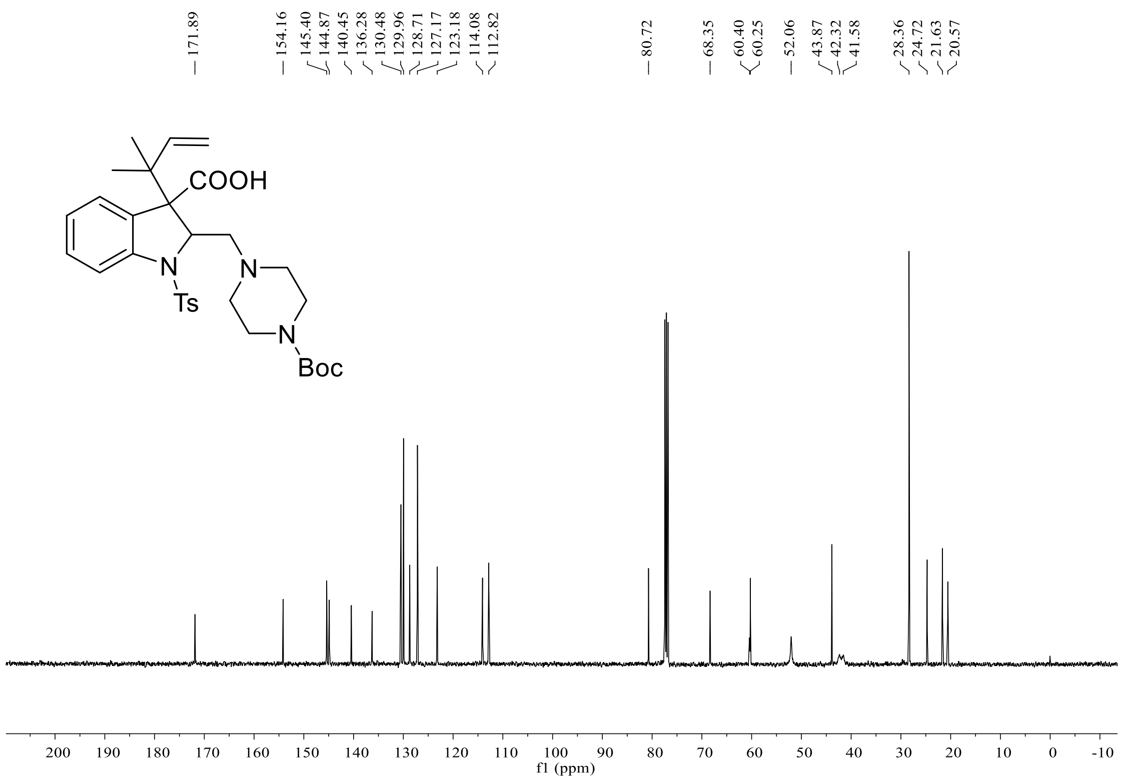

Supplementary Fig. 101. ^{13}C NMR of compound **3u** (100 MHZ, $CDCl_3$)

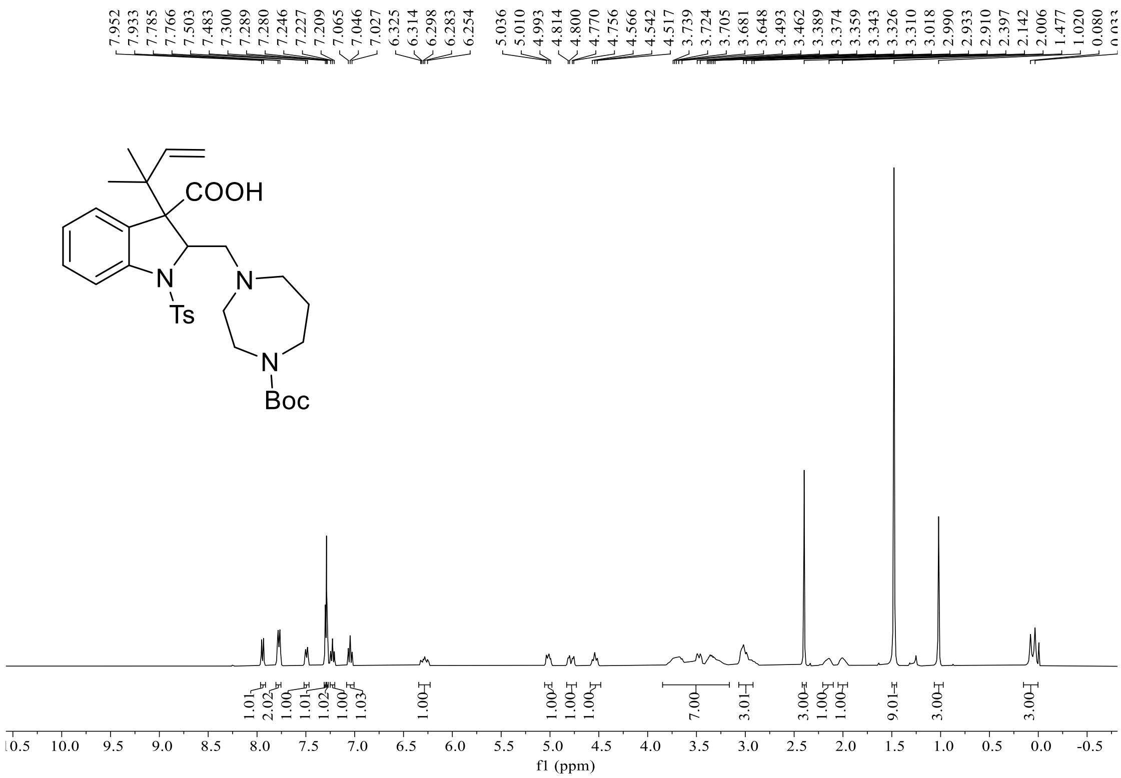

Supplementary Fig. 102. ^{1}H NMR of compound **3v** (400 MHZ, $CDCl_3$)

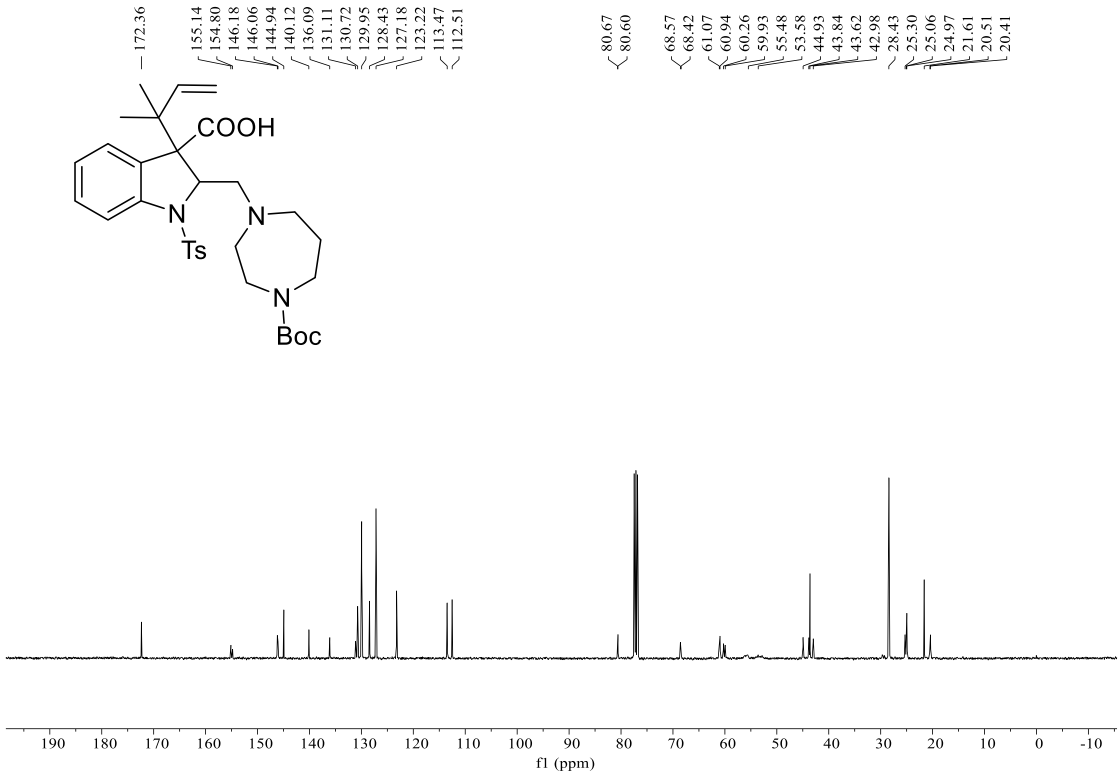

Supplementary Fig. 103. ^{13}C NMR of compound **3v** (100 MHZ, $CDCl_3$)

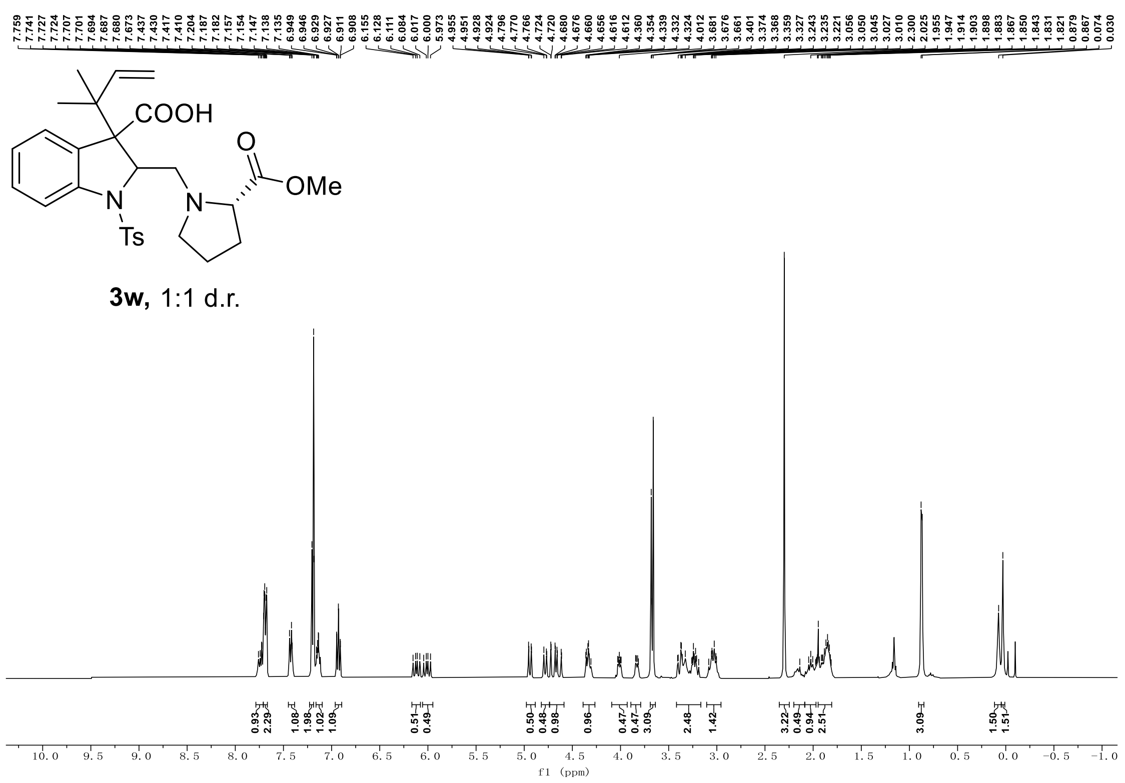

Supplementary Fig. 104. ^{1}H NMR of compound **3w** (diastereoisomer mixture, 400 MHZ, $CDCl_3$)

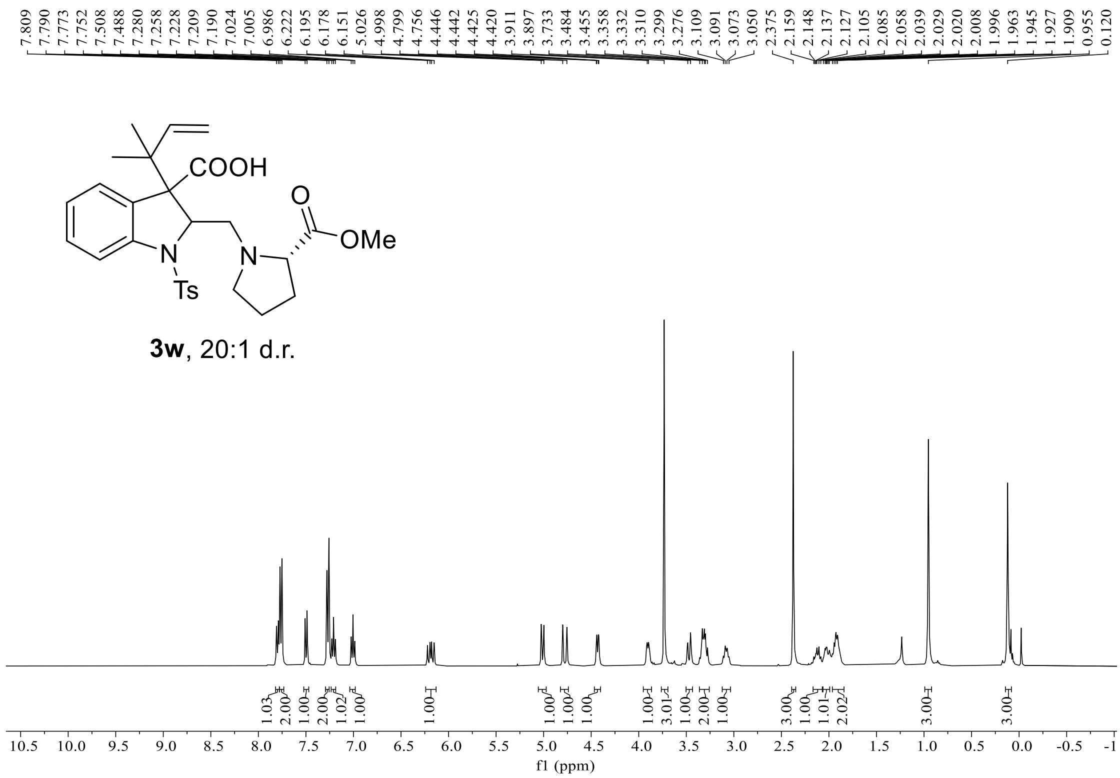

Supplementary Fig. 105. ^{1}H NMR of compound **3w** (400 MHZ, $CDCl_3$)

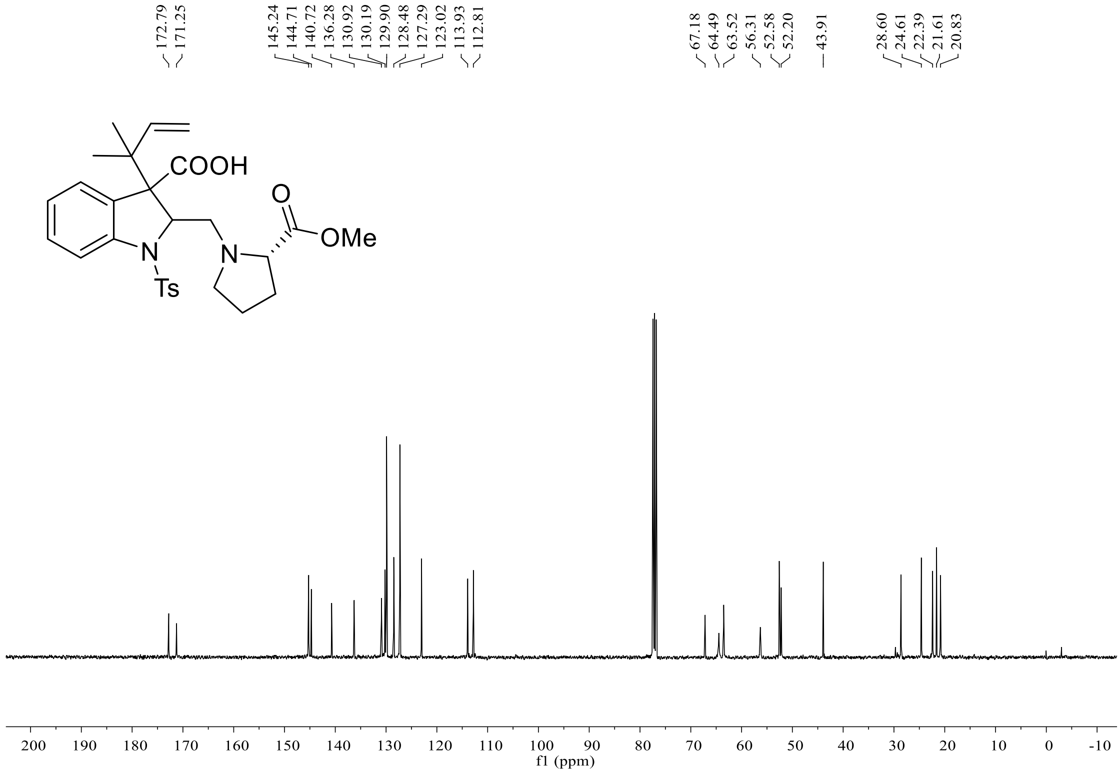

Supplementary Fig. 106. ^{13}C NMR of compound **3w** (100 MHZ, $CDCl_3$)

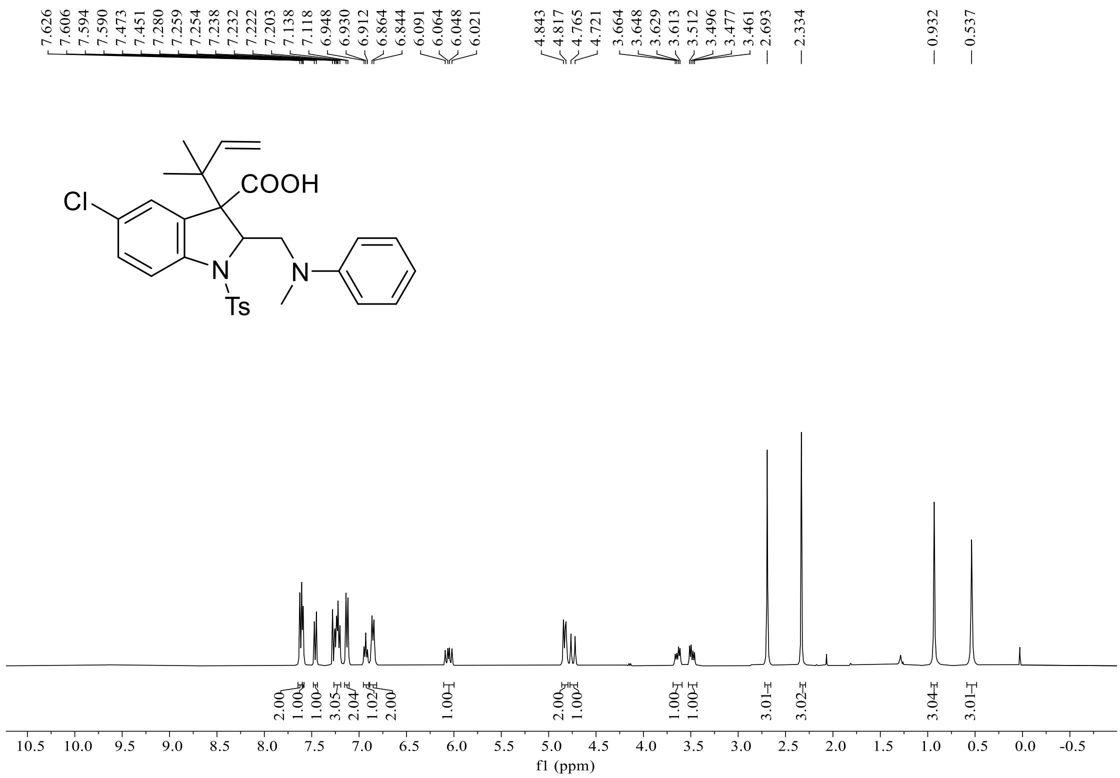

Supplementary Fig. 107. ^{1}H NMR of compound **3x** (400 MHZ, $CDCl_3$)

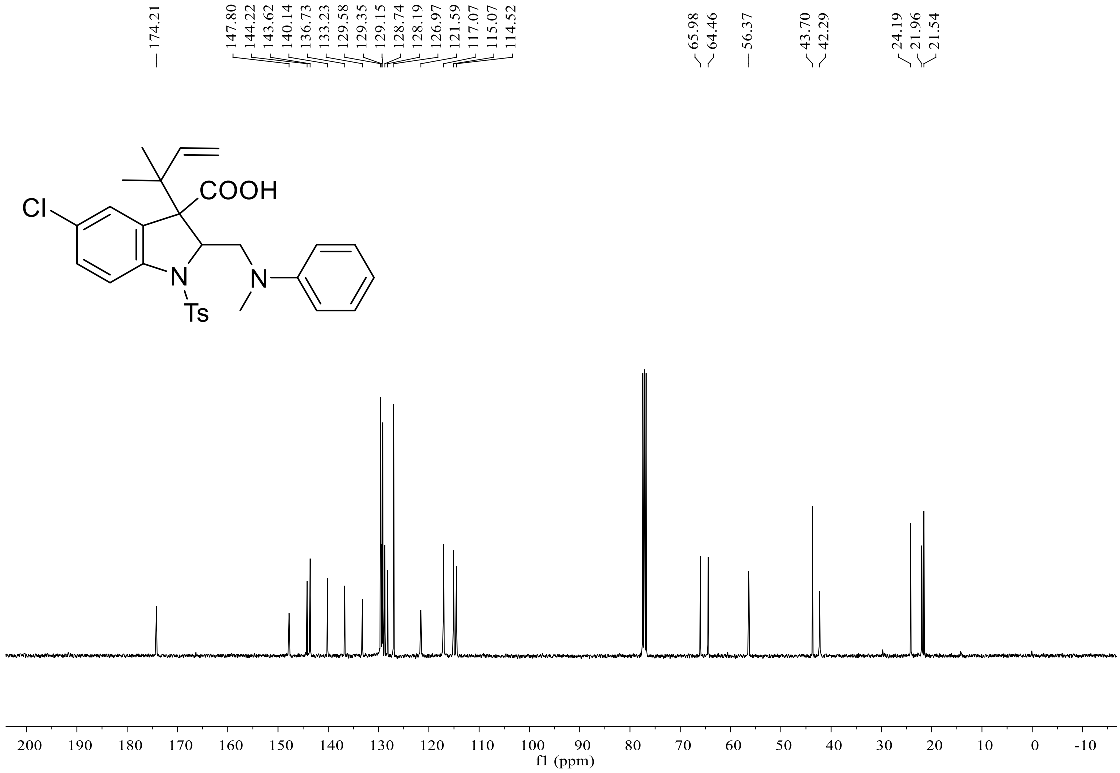

Supplementary Fig. 108. ^{13}C NMR of compound **3x** (100 MHZ, $CDCl_3$)

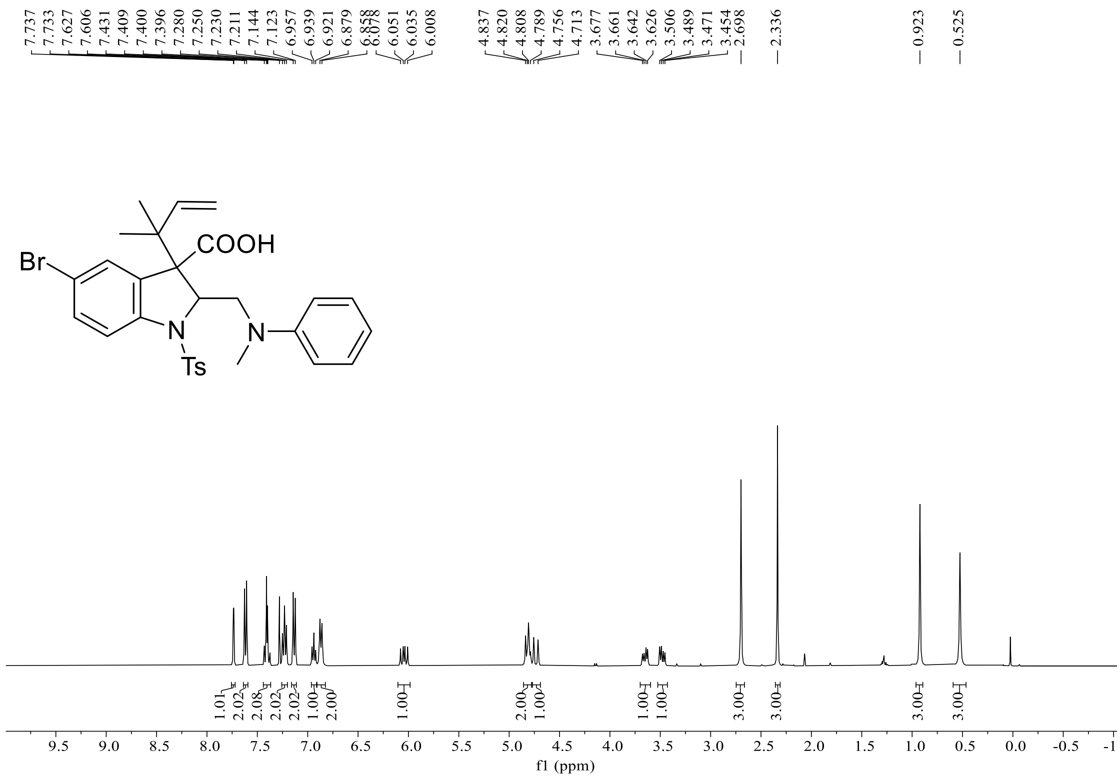

Supplementary Fig. 109. ^{1}H NMR of compound **3y** (400 MHZ, $CDCl_3$)

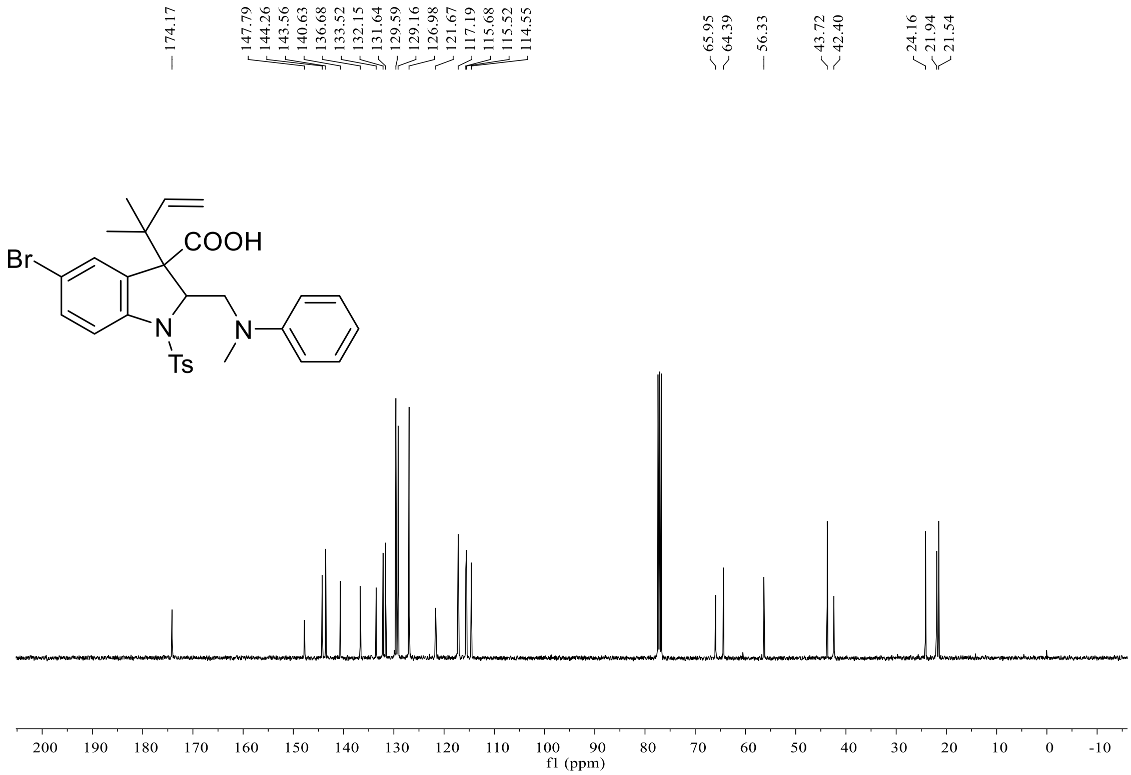

Supplementary Fig. 110. ^{13}C NMR of compound **3y** (100 MHZ, $CDCl_3$)

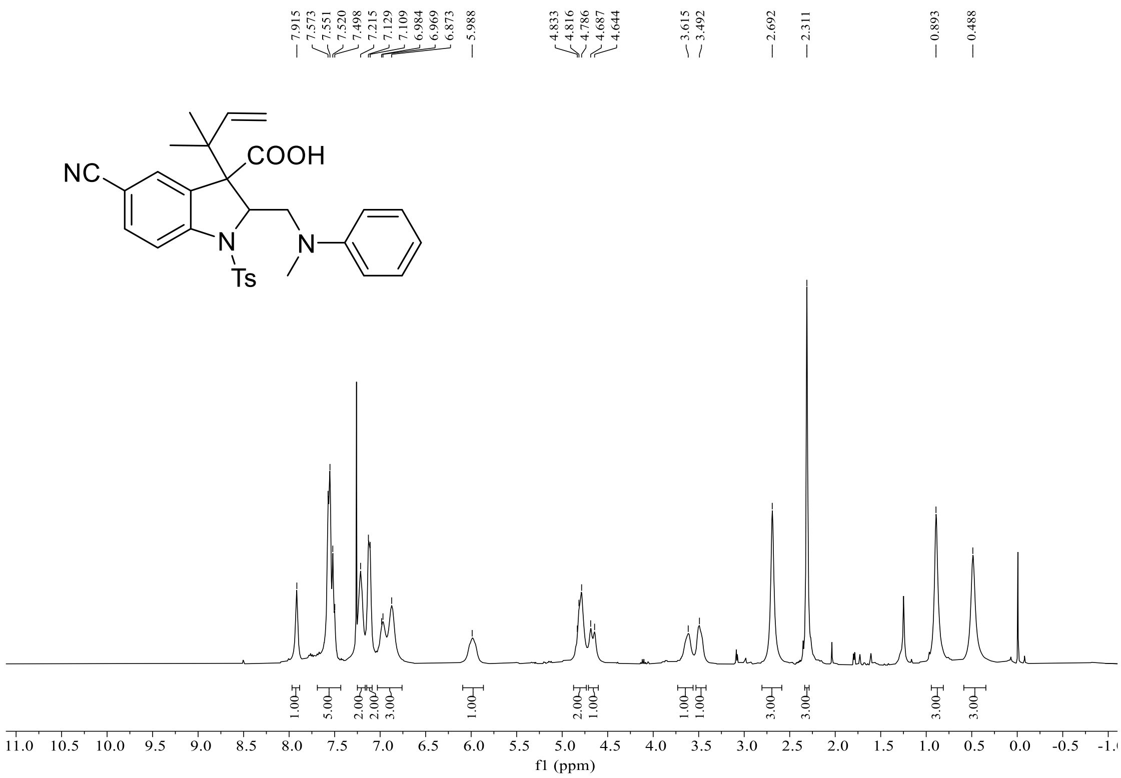

Supplementary Fig. 111. ^{1}H NMR of compound **3z** (400 MHZ, $CDCl_3$)

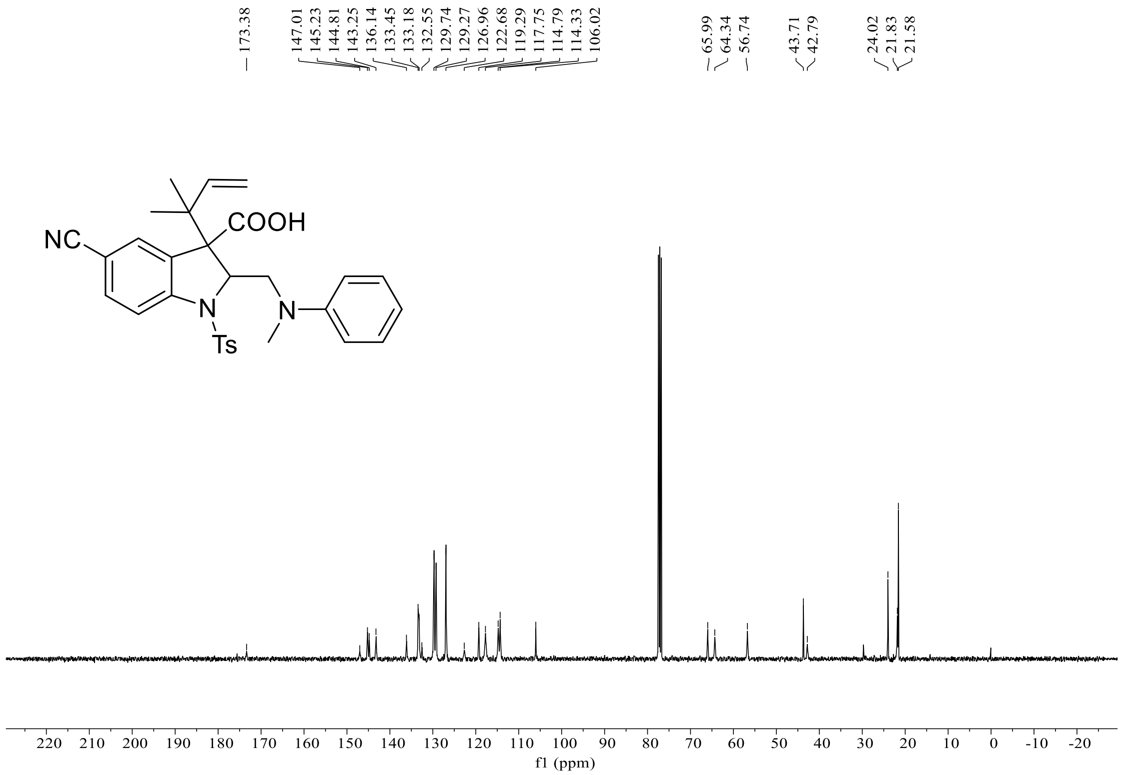

Supplementary Fig. 112. ^{13}C NMR of compound **3z** (100 MHZ, $CDCl_3$)

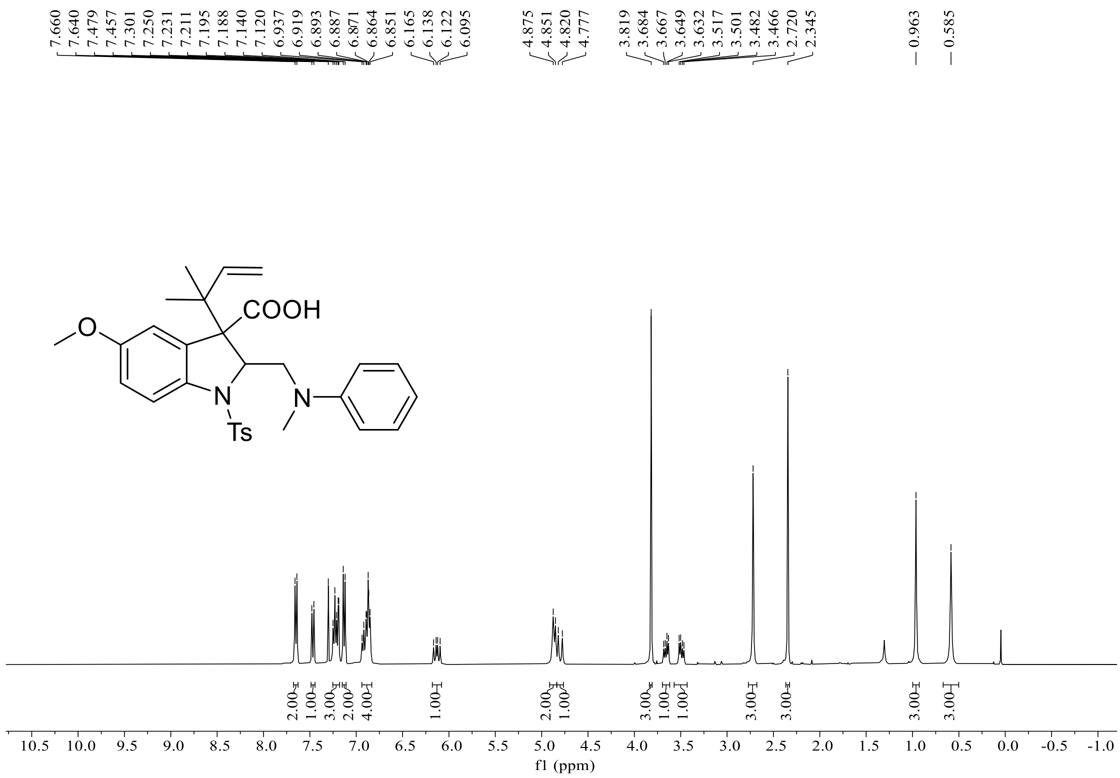

Supplementary Fig. 113. ^{1}H NMR of compound **3aa** (400 MHZ, $CDCl_3$)

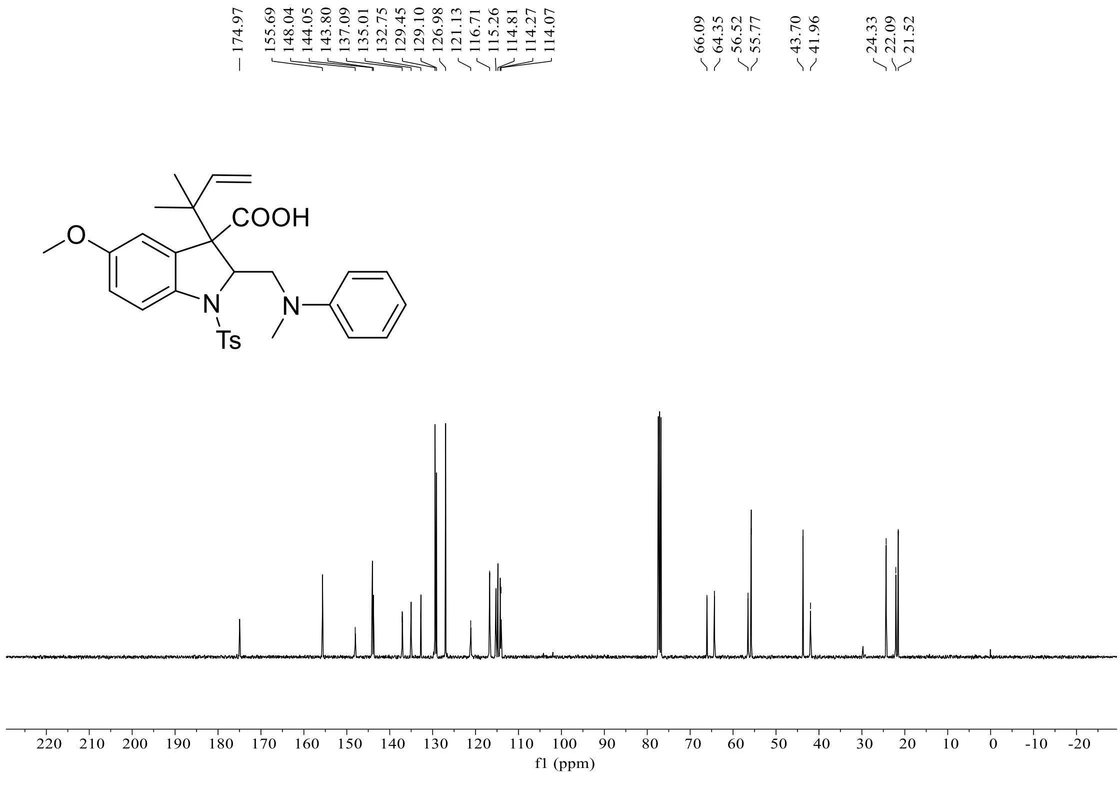

Supplementary Fig. 114. ^{13}C NMR of compound **3aa** (100 MHZ, $CDCl_3$)

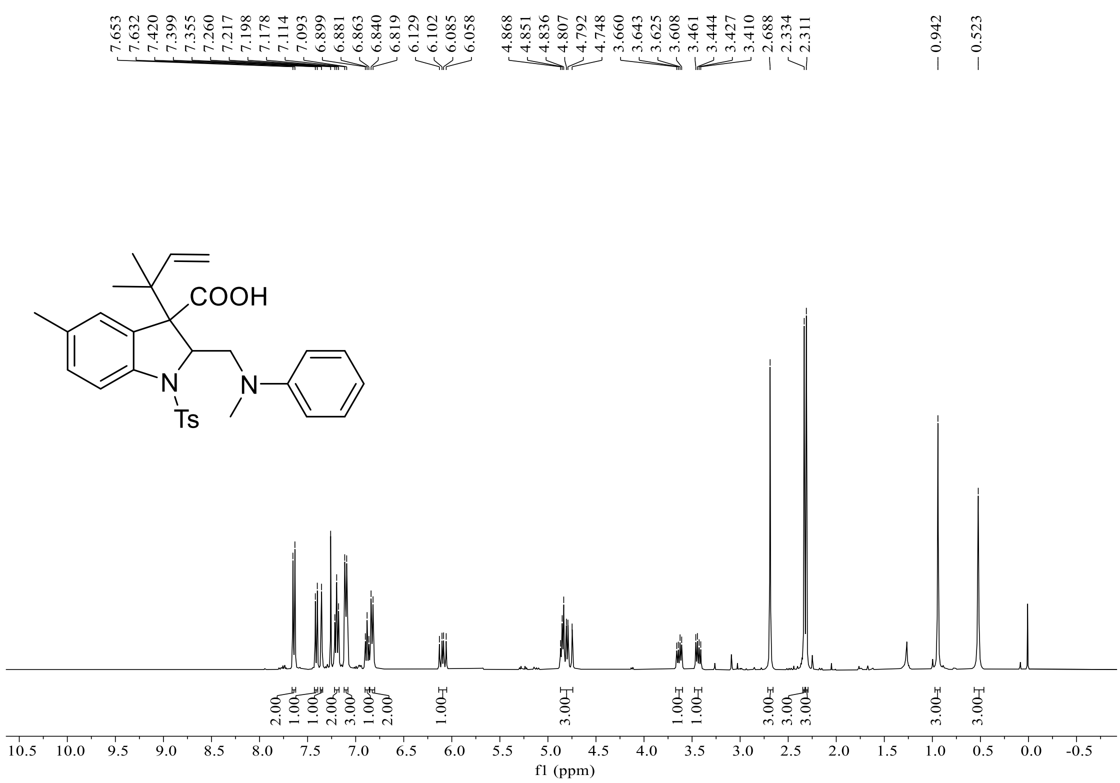

Supplementary Fig. 115. ^{1}H NMR of compound **3ab** (400 MHZ, $CDCl_3$)

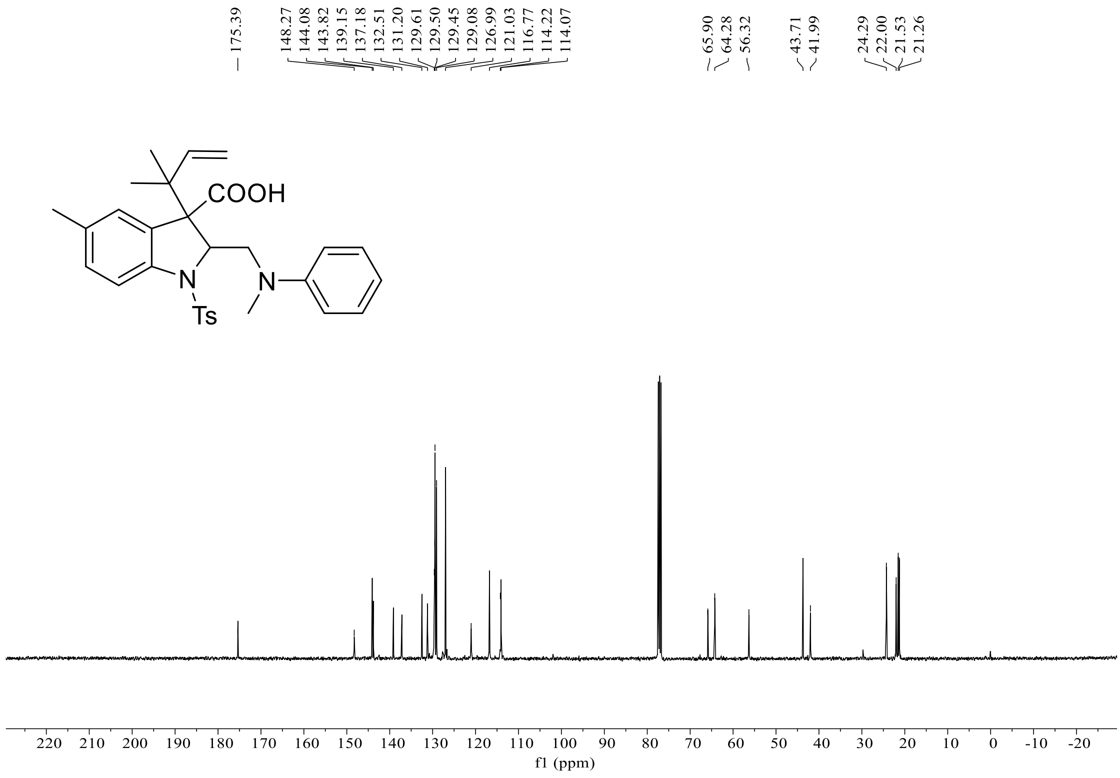

Supplementary Fig. 116. ^{13}C NMR of compound **3ab** (100 MHZ, $CDCl_3$)

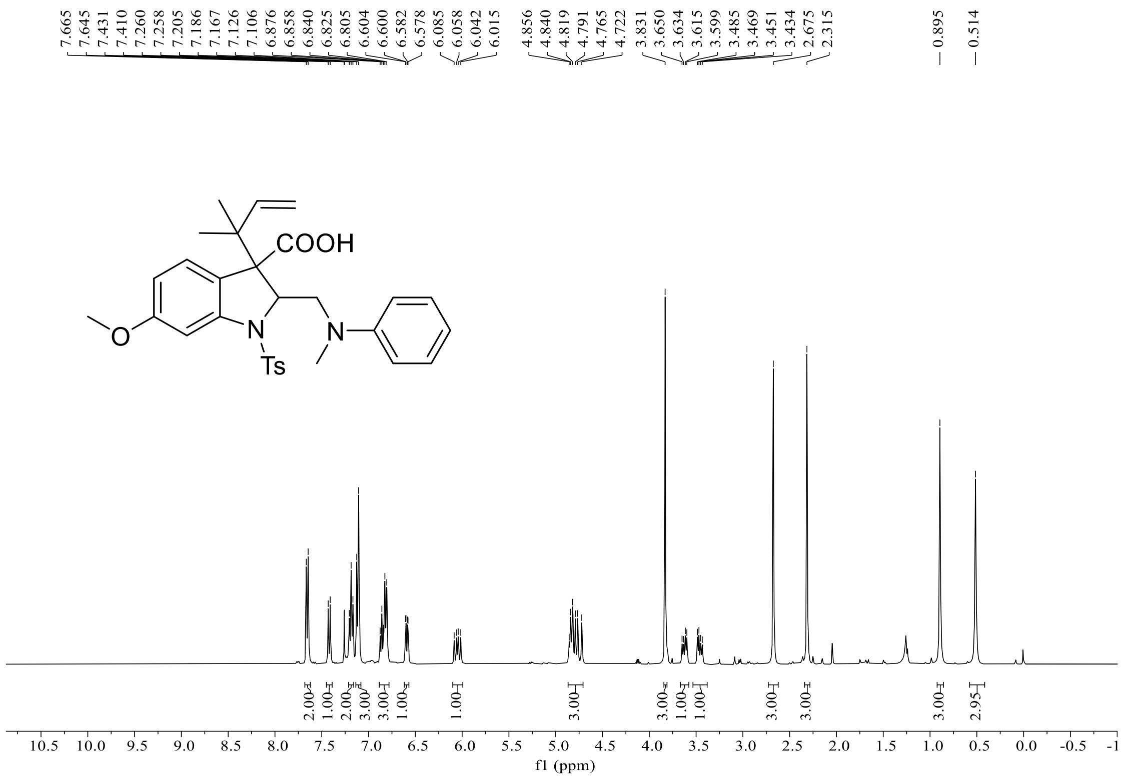

Supplementary Fig. 117. ^{1}H NMR of compound **3ac** (400 MHZ, $CDCl_3$)

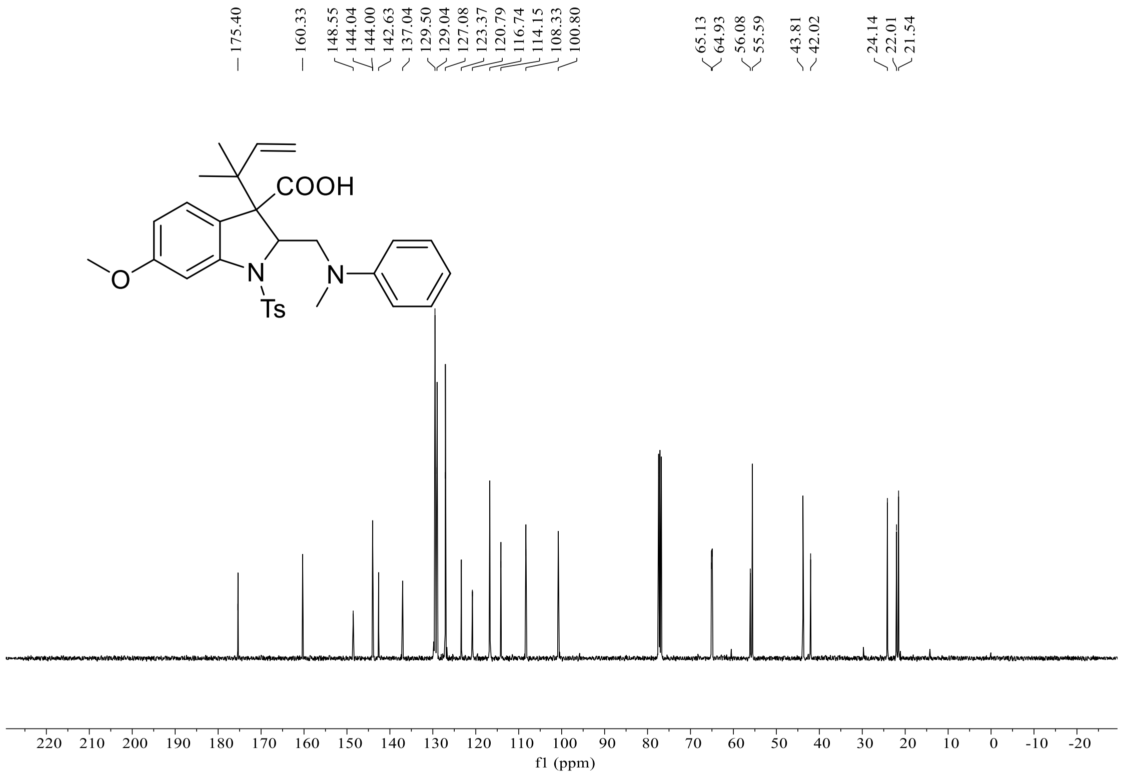

Supplementary Fig. 118. ^{13}C NMR of compound **3ac** (100 MHZ, $CDCl_3$)

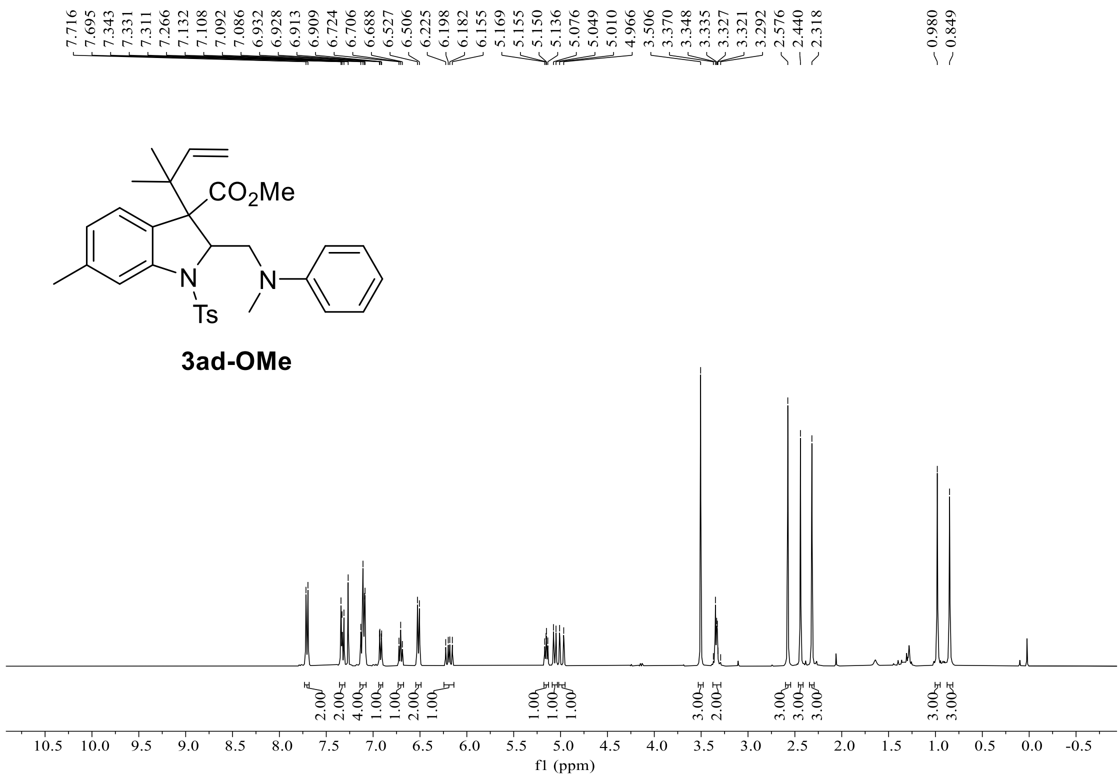

Supplementary Fig. 119. ^{1}H NMR of compound **3ad-OMe** (400 MHZ, $CDCl_3$)

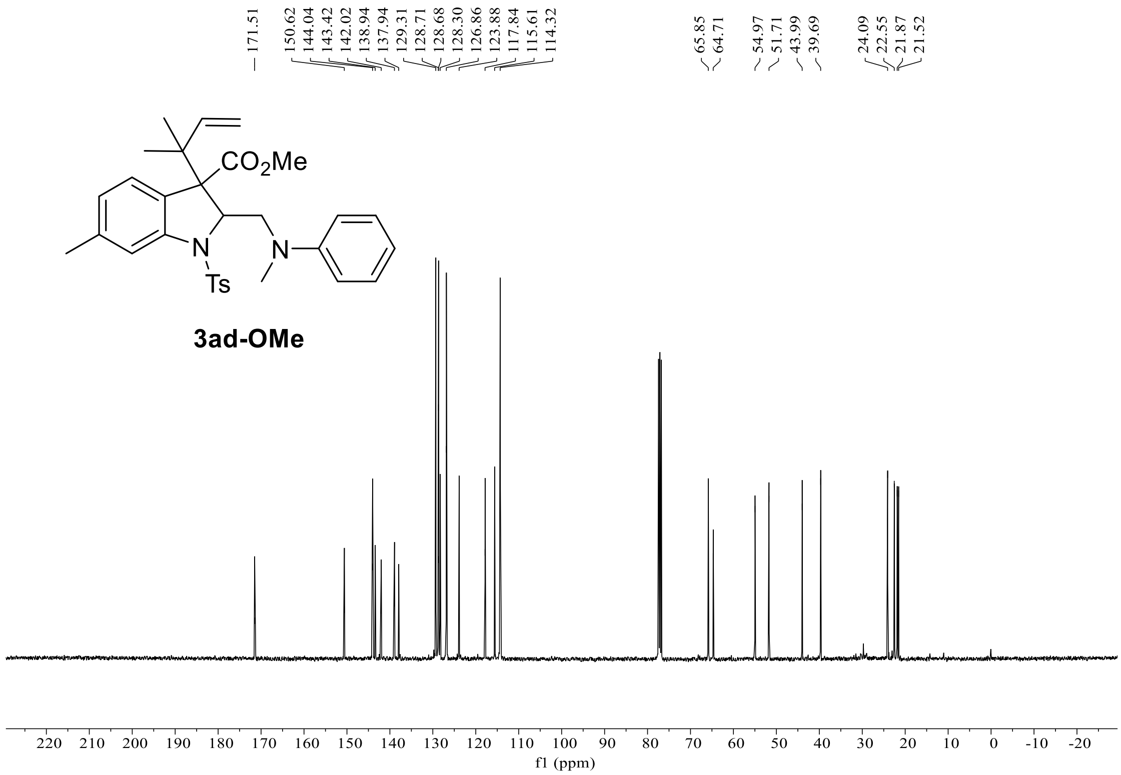

Supplementary Fig. 120. ^{13}C NMR of compound **3ad-OMe** (100 MHZ, $CDCl_3$)

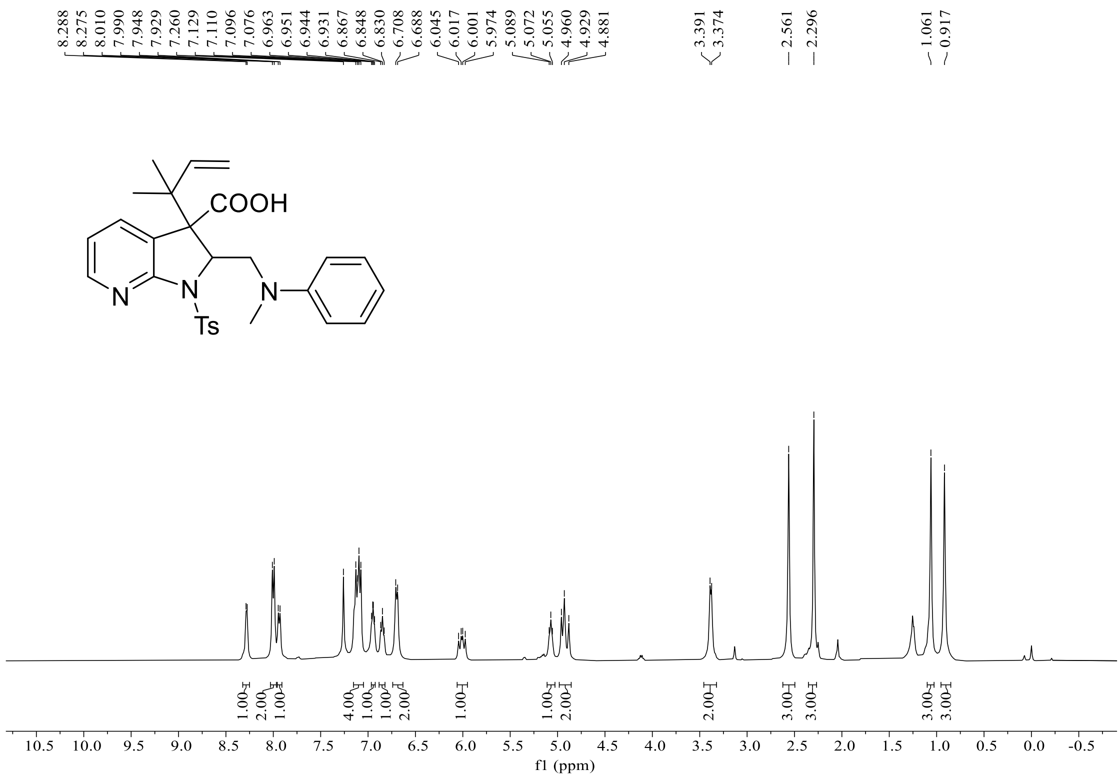

Supplementary Fig. 121. ^{1}H NMR of compound **3ae** (400 MHZ, $CDCl_3$)

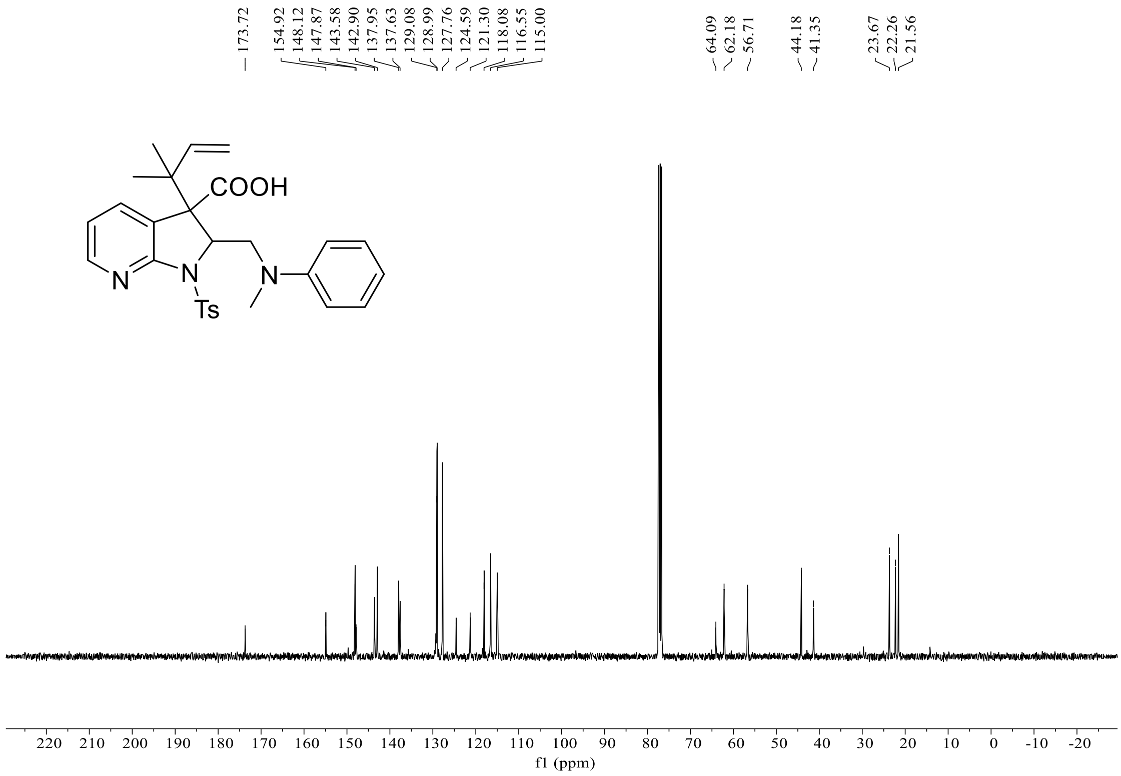

Supplementary Fig. 122. ^{13}C NMR of compound **3ae** (100 MHZ, $CDCl_3$)

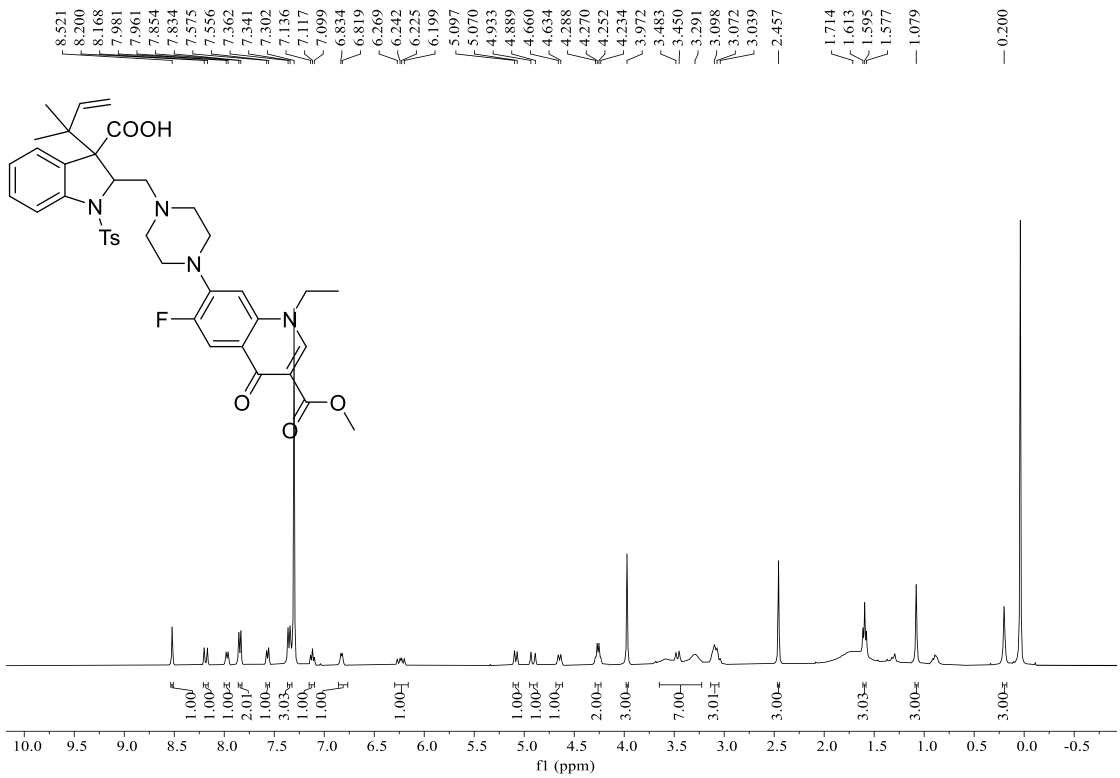

Supplementary Fig. 123. ^{1}H NMR of compound **3da** (400 MHZ, $CDCl_3$)

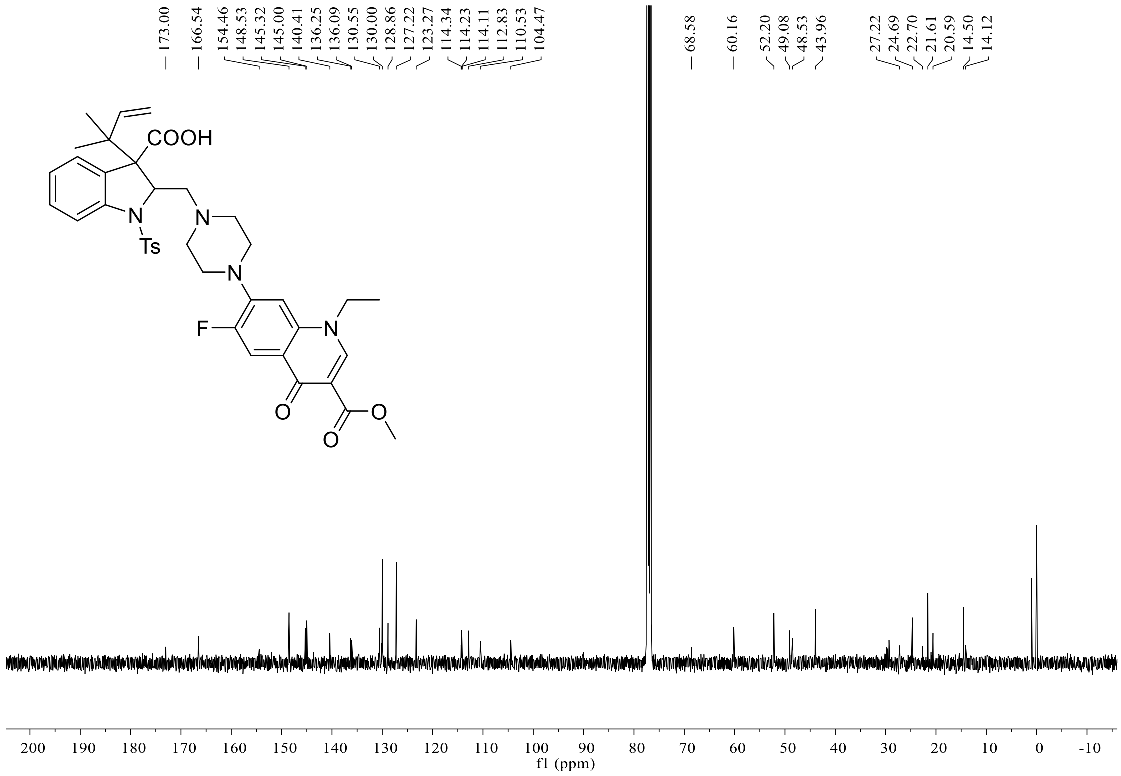

Supplementary Fig. 124. ^{13}C NMR of compound **3da** (100 MHZ, $CDCl_3$)

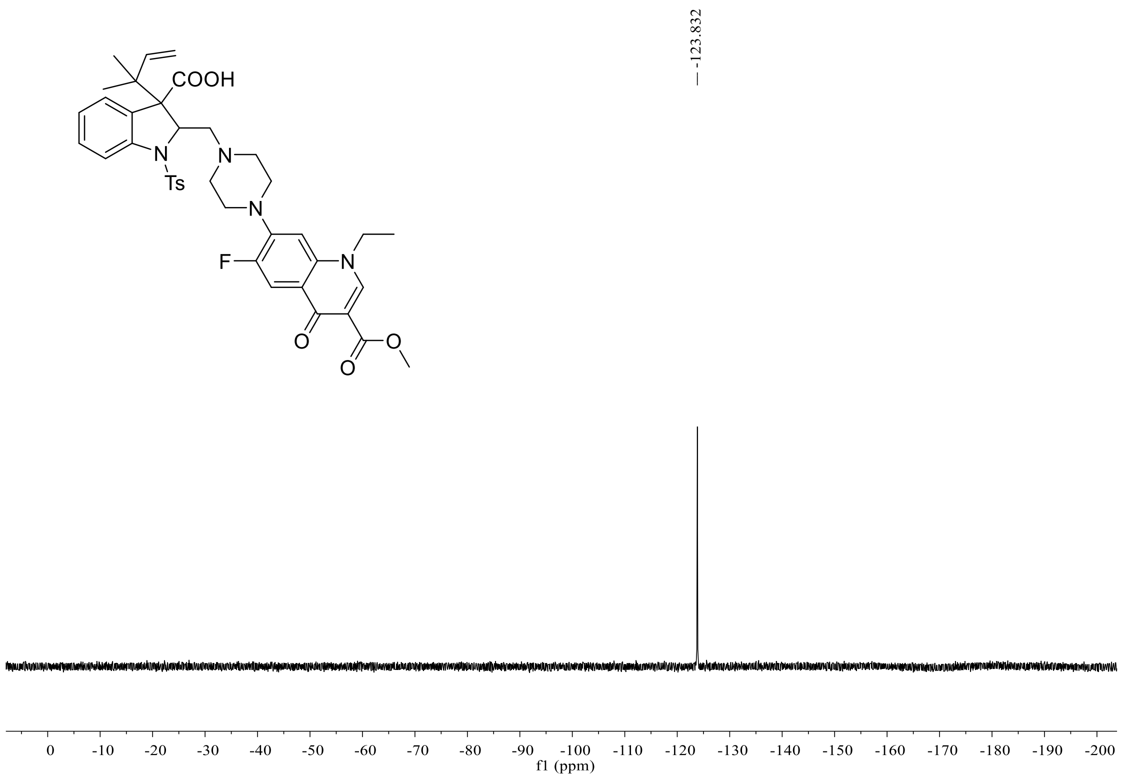

Supplementary Fig. 125. ^{19}F NMR of compound **3da** (376 MHZ, $CDCl_3$)

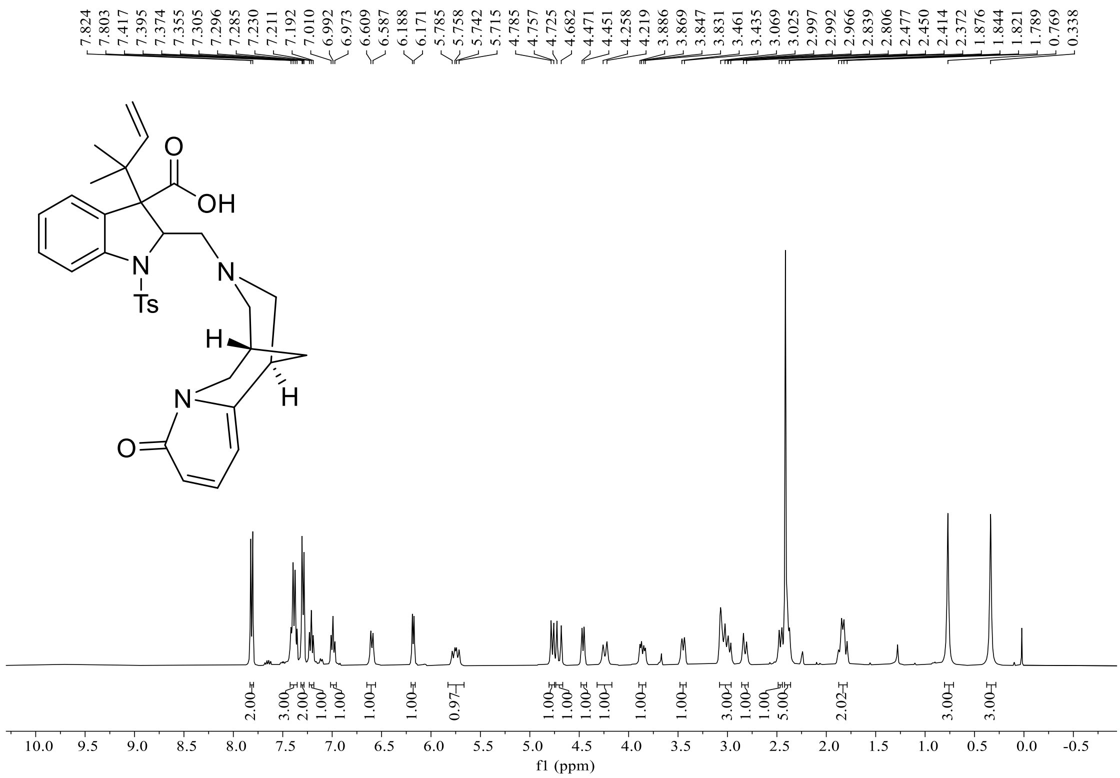

Supplementary Fig. 126. ^{1}H NMR of compound **3db** (400 MHZ, $CDCl_3$)

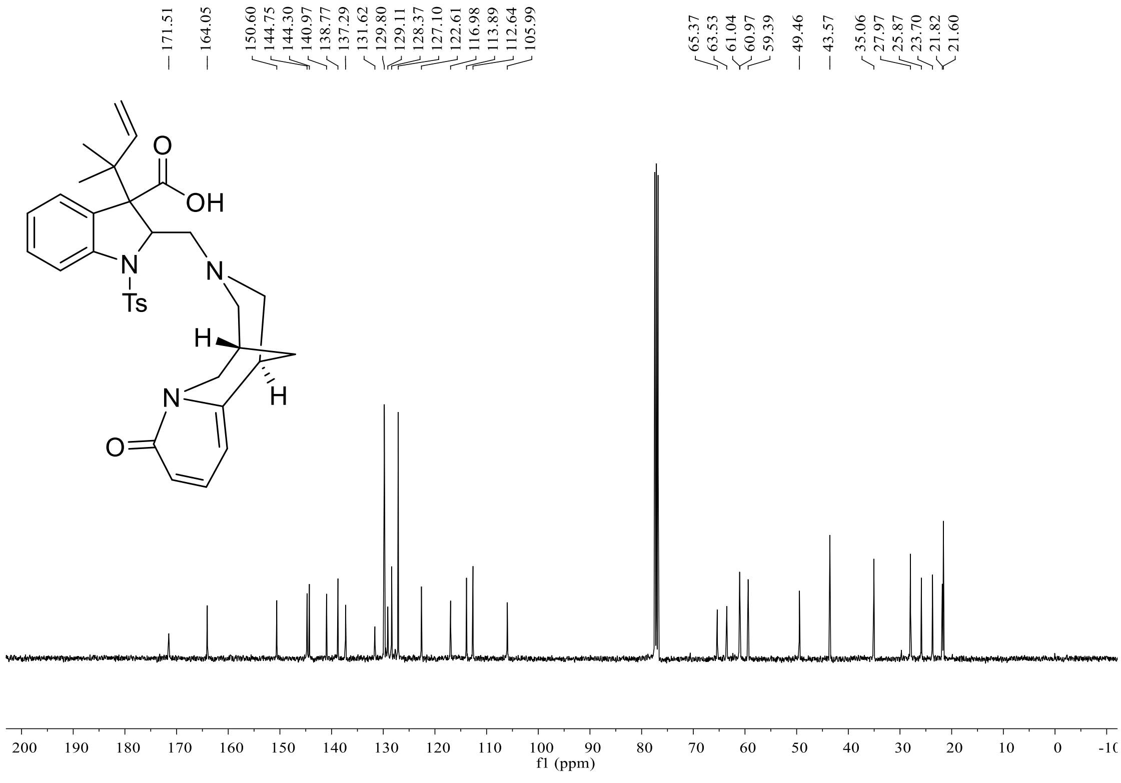

Supplementary Fig. 127. ^{13}C NMR of compound **3db** (100 MHZ, $CDCl_3$)

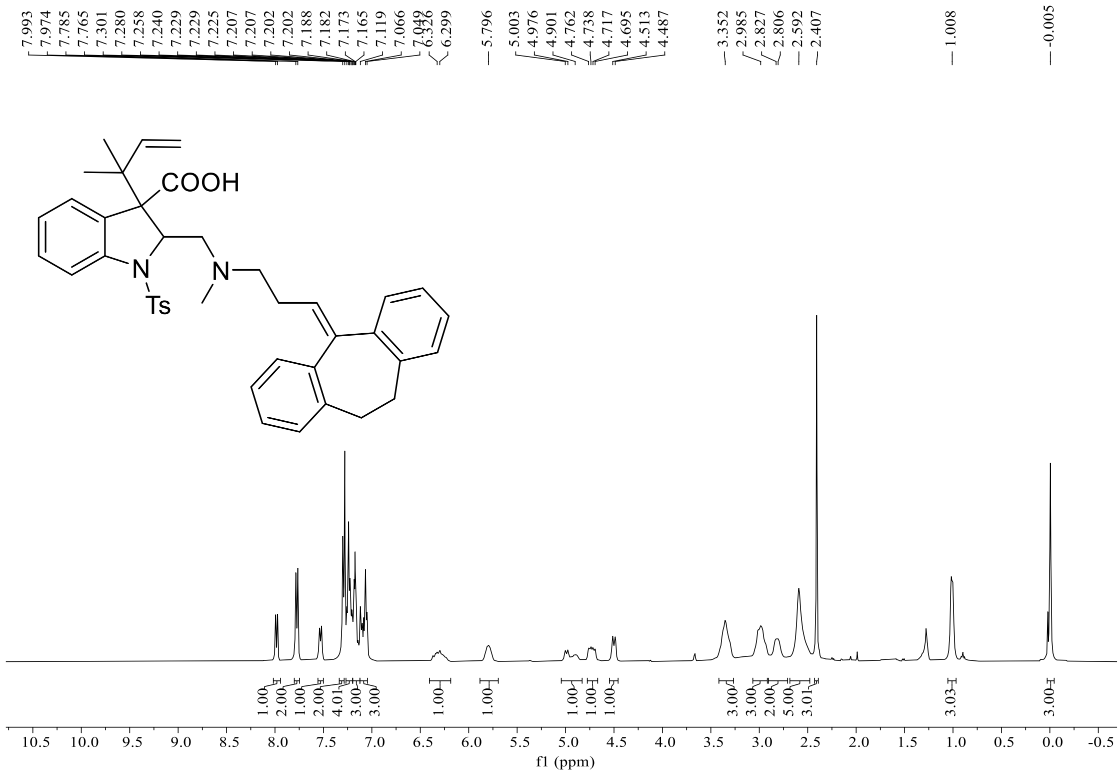

Supplementary Fig. 128. ^{1}H NMR of compound **3dc** (400 MHZ, $CDCl_3$)

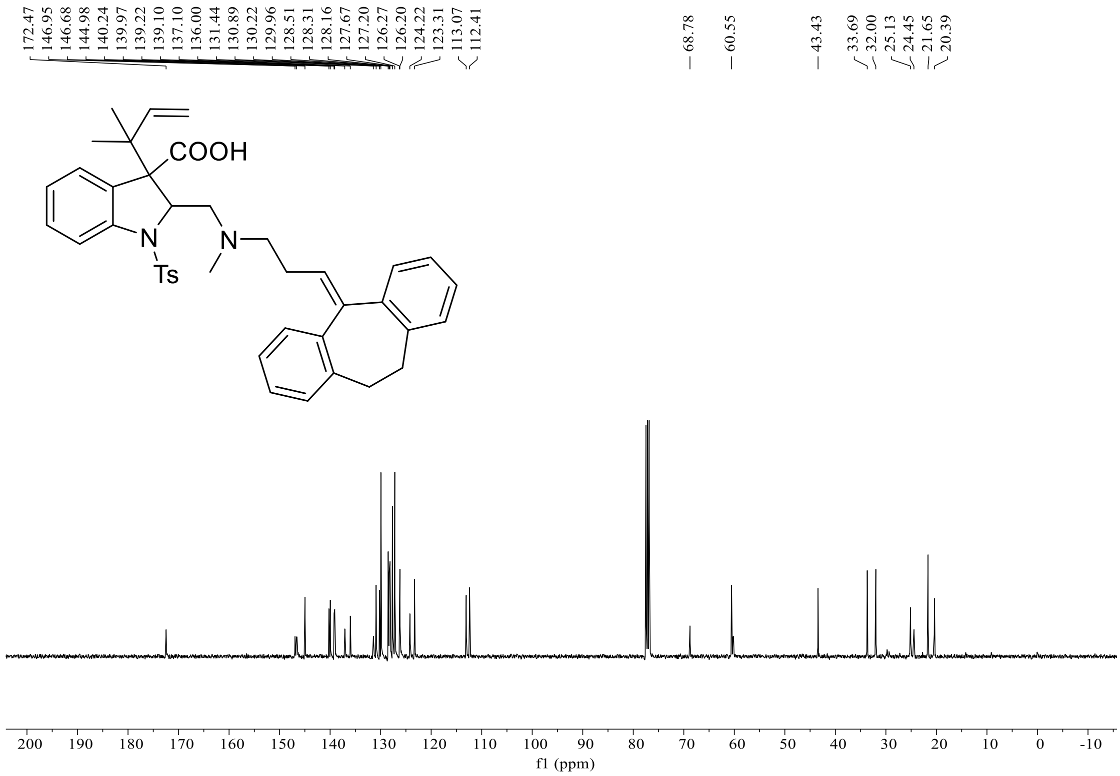

Supplementary Fig. 129. ^{13}C NMR of compound **3dc** (100 MHZ, $CDCl_3$)

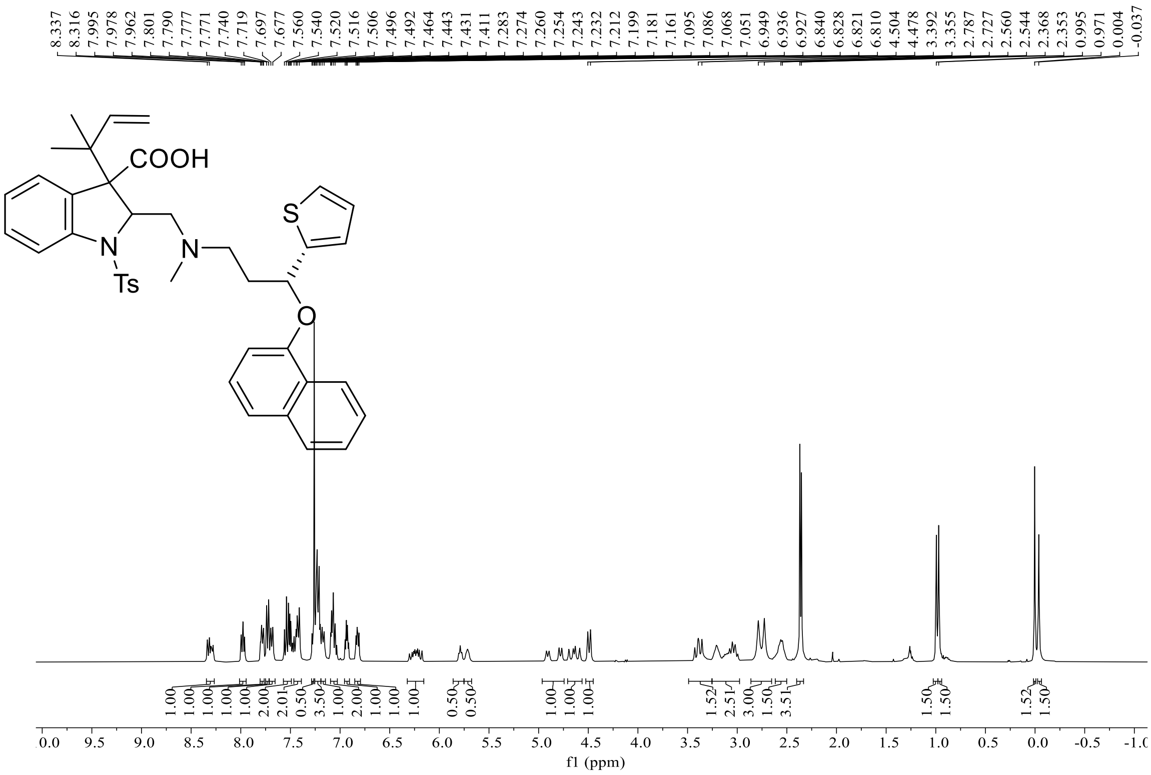

Supplementary Fig. 130. ^{1}H NMR of compound **3dd** (400 MHZ, $CDCl_3$)

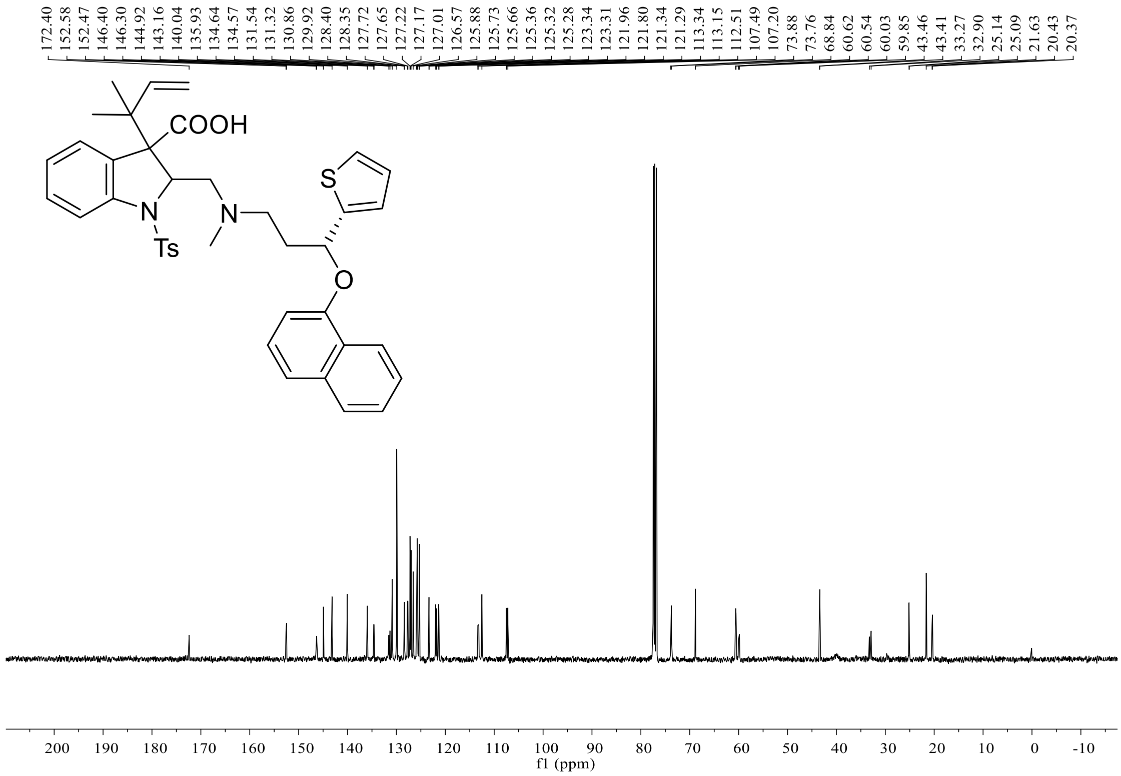

Supplementary Fig. 131. ^{13}C NMR of compound **3dd** (100 MHZ, $CDCl_3$)

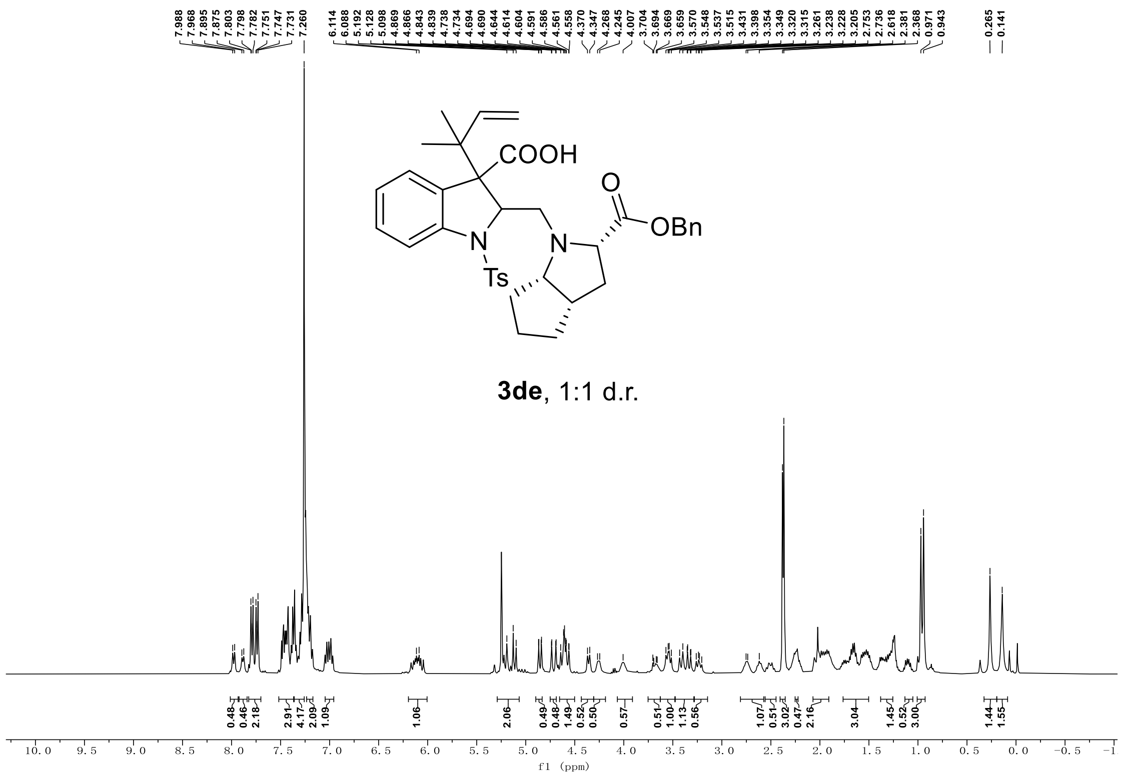

Supplementary Fig. 132. ¹H NMR of compound **3de** (diastereoisomer mixture, 400 MHZ, $CDCl_3$)

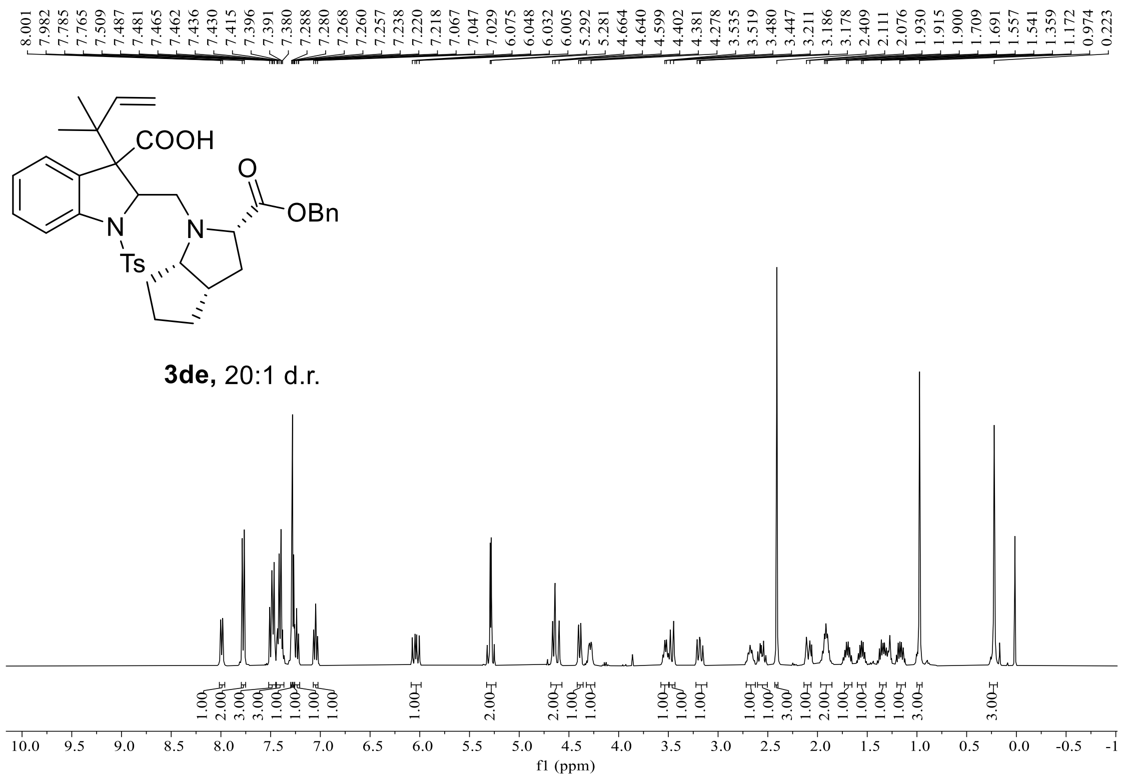

Supplementary Fig. 133. ^{1}H NMR of compound **3de** (400 MHZ, $CDCl_3$)

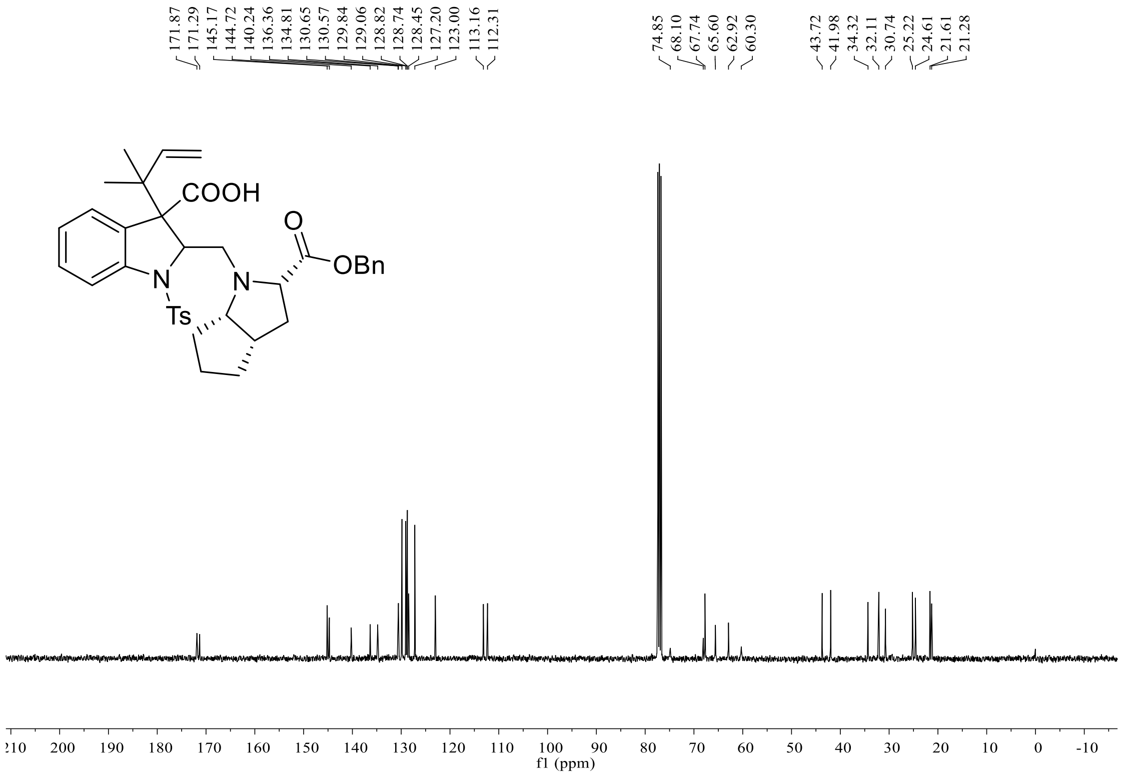

Supplementary Fig. 134. ^{13}C NMR of compound **3de** (100 MHZ, $CDCl_3$)

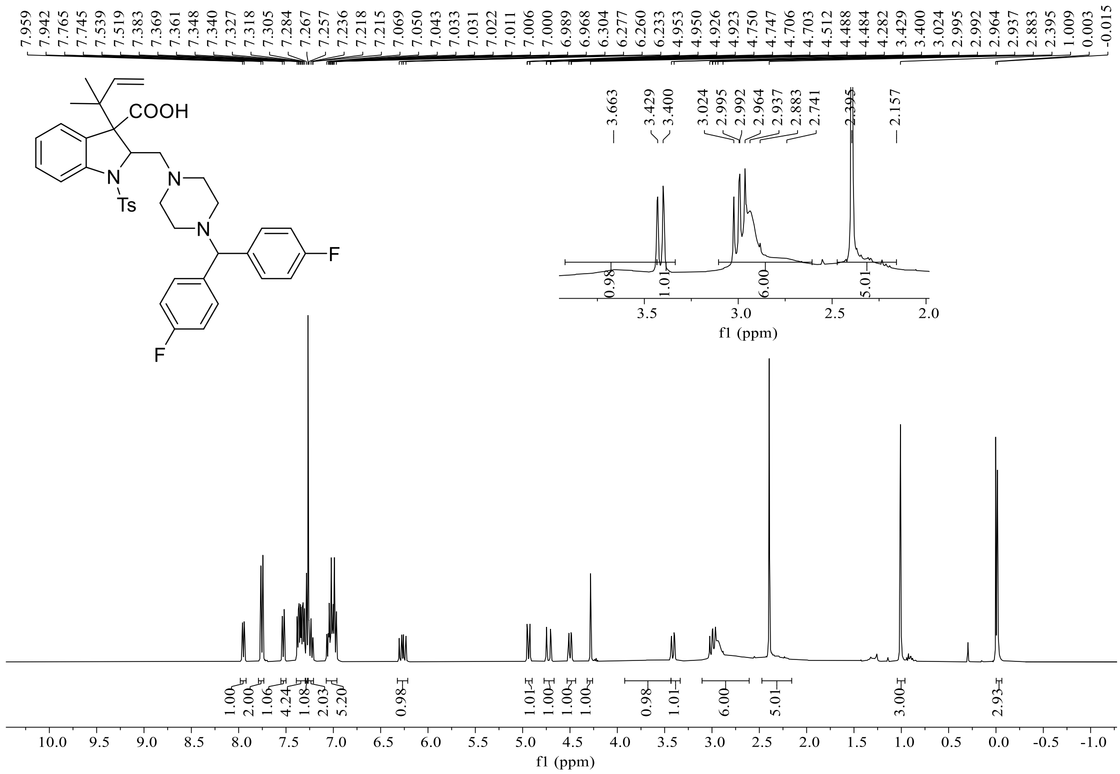

Supplementary Fig. 135. ^{1}H NMR of compound **3df** (400 MHZ, $CDCl_3$)

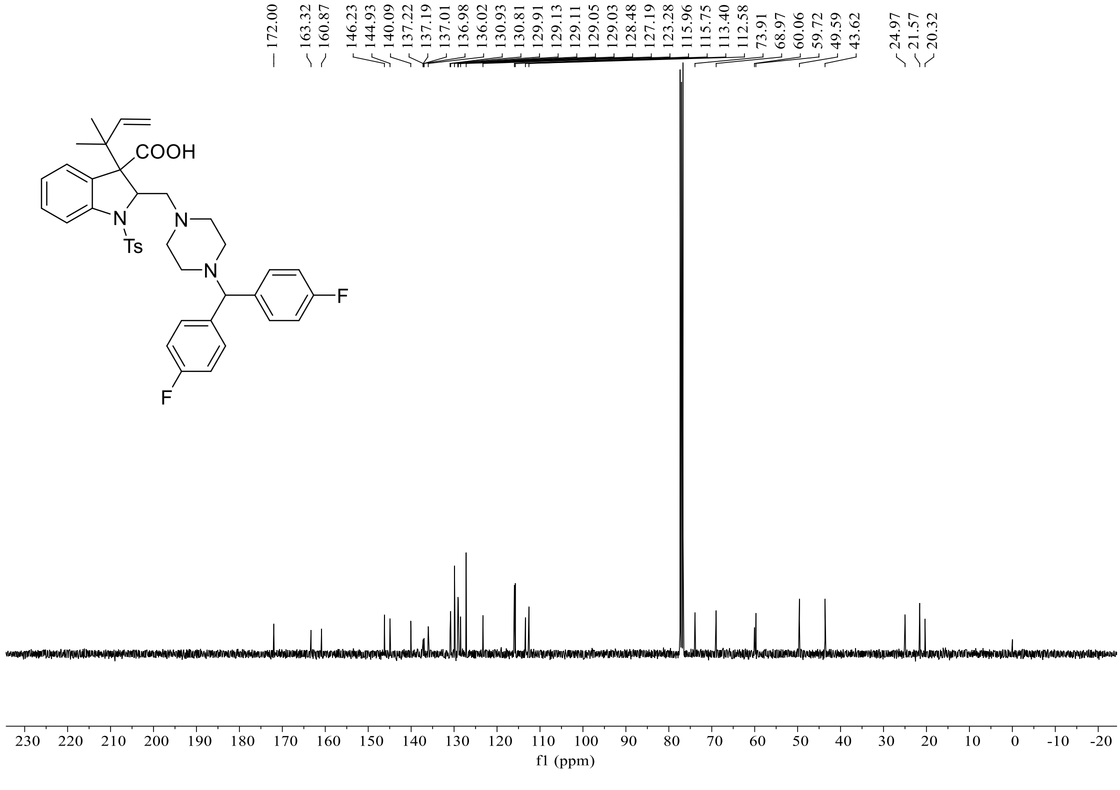

Supplementary Fig. 136. ^{13}C NMR of compound **3df** (100 MHZ, $CDCl_3$)

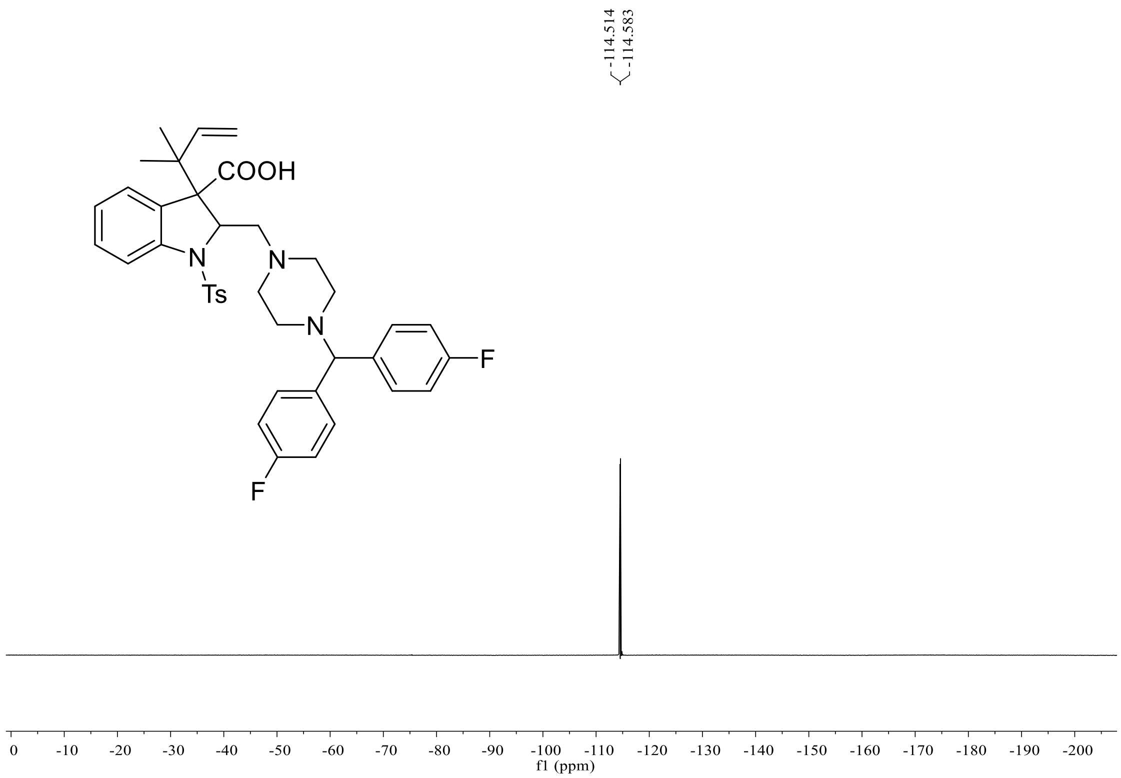

Supplementary Fig. 137. ^{1}H NMR of compound **3df** (400 MHZ, CDCl$_3$)

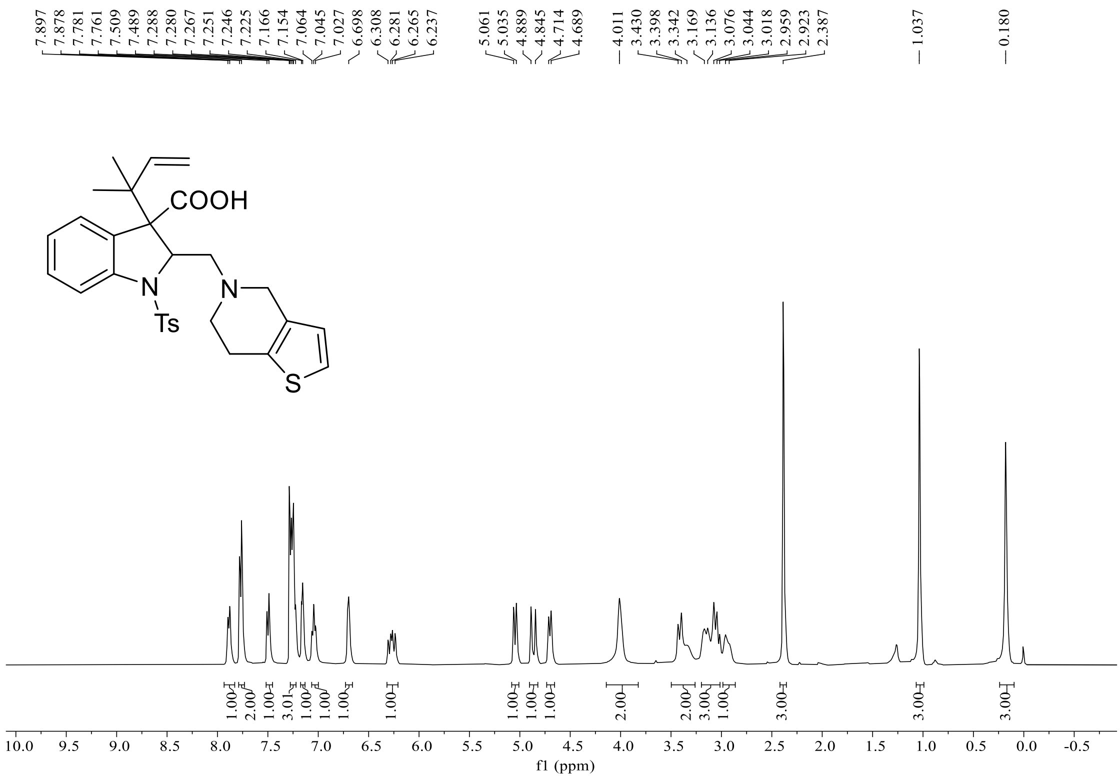

Supplementary Fig. 138. ^{1}H NMR of compound **3dg** (400 MHZ, $CDCl_3$)

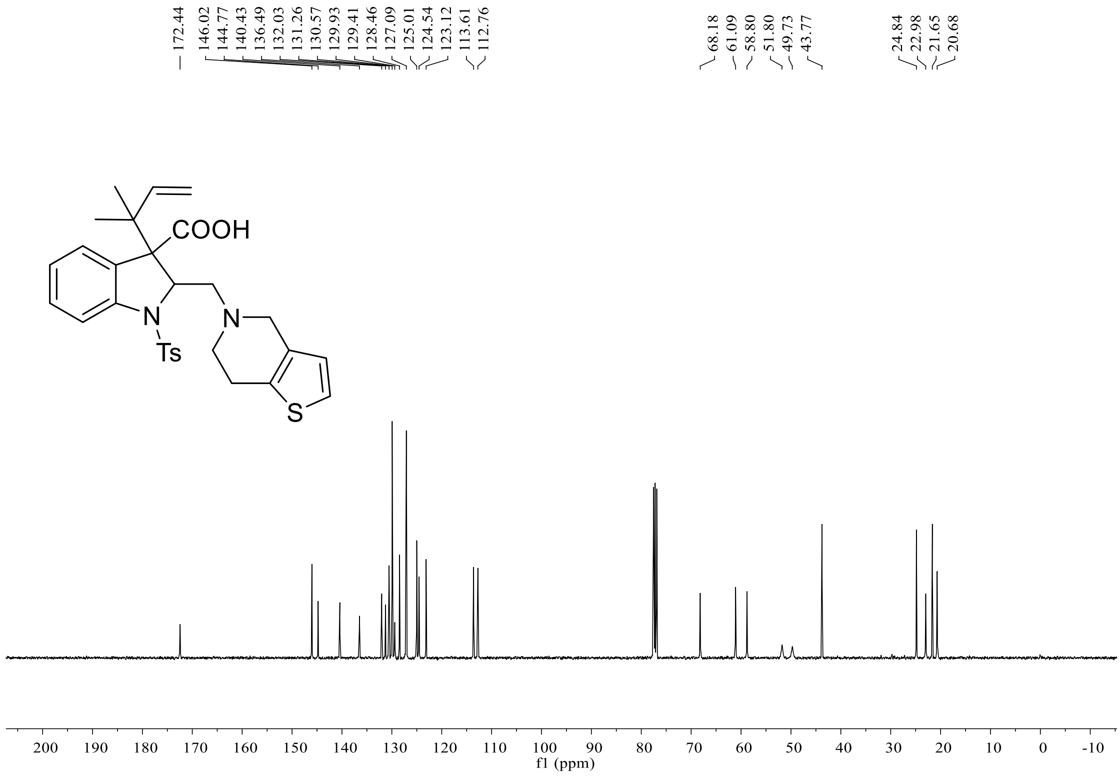

Supplementary Fig. 139. ^{13}C NMR of compound **3dg** (100 MHZ, $CDCl_3$)

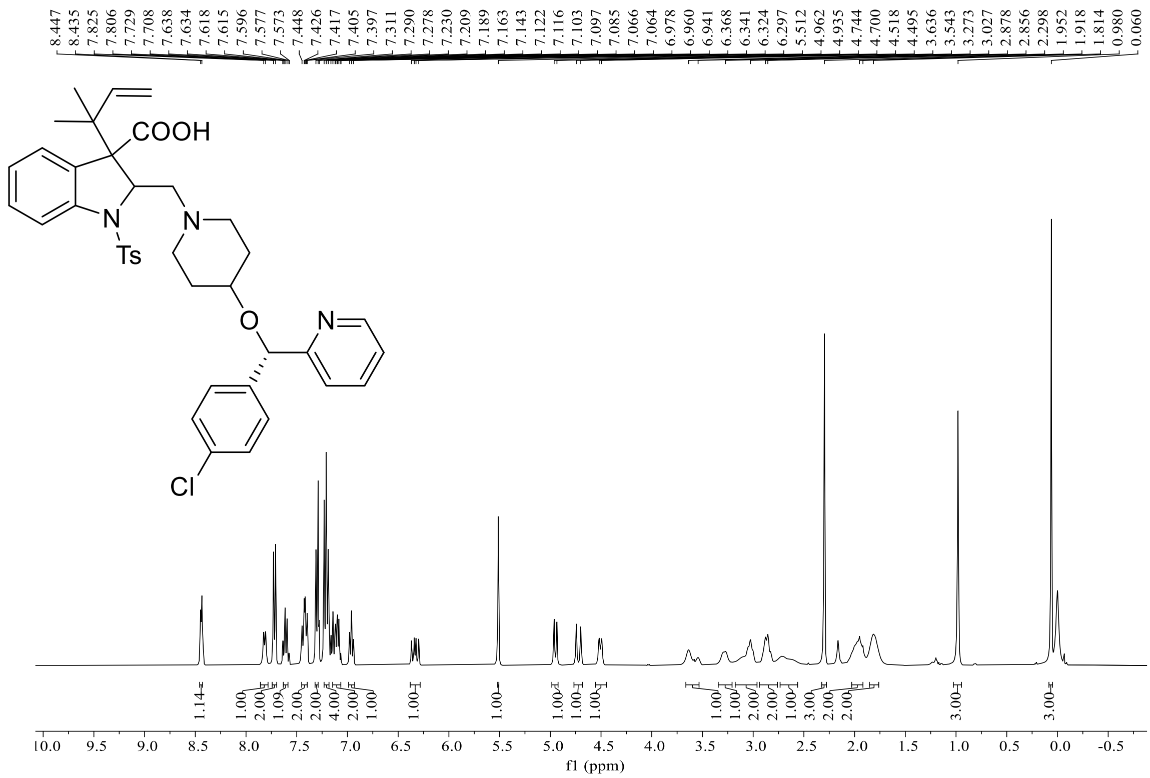

Supplementary Fig. 140. ^{1}H NMR of compound **3dh** (400 MHZ, $CDCl_3$)

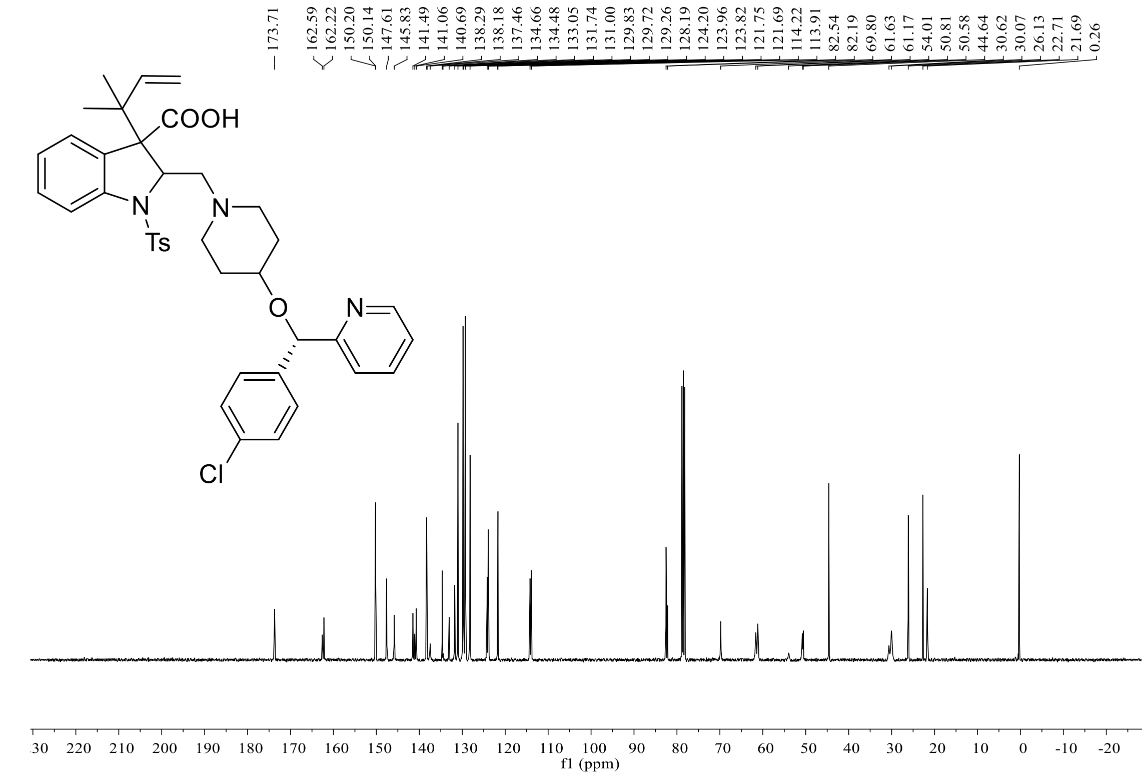

Supplementary Fig. 141. ^{13}C NMR of compound **3dh** (100 MHZ, $CDCl_3$)

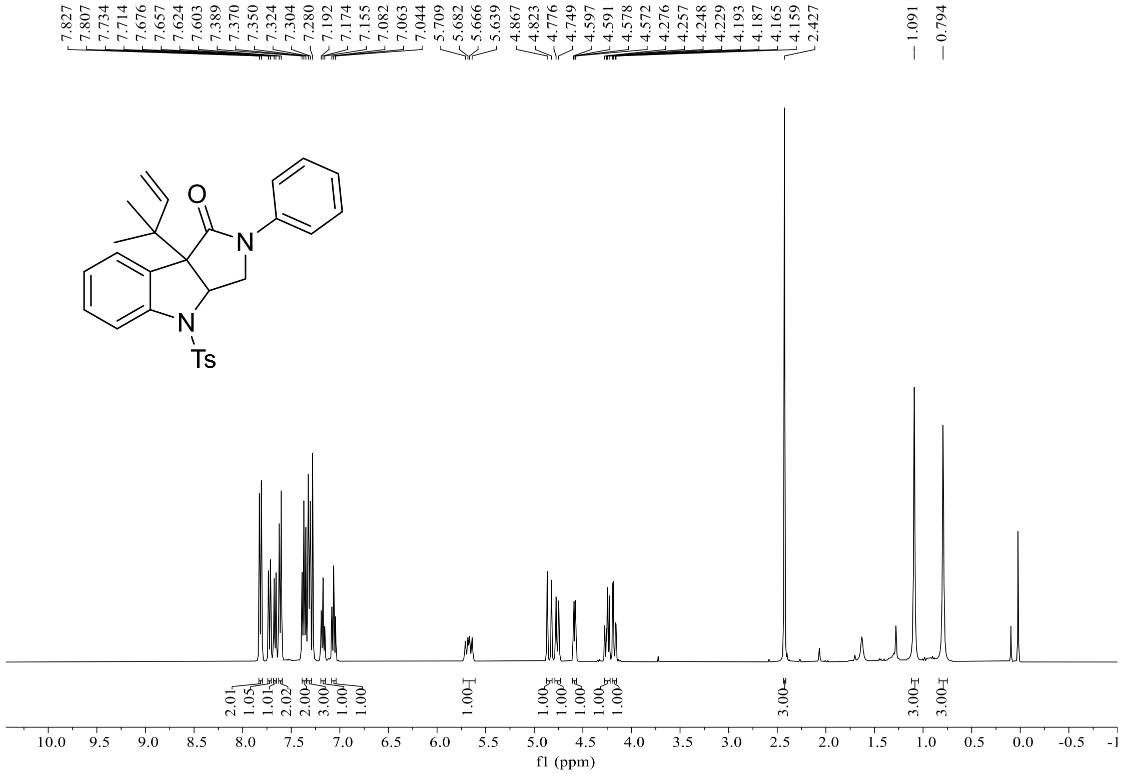

Supplementary Fig. 142. ^{1}H NMR of compound **5a** (400 MHZ, $CDCl_3$)

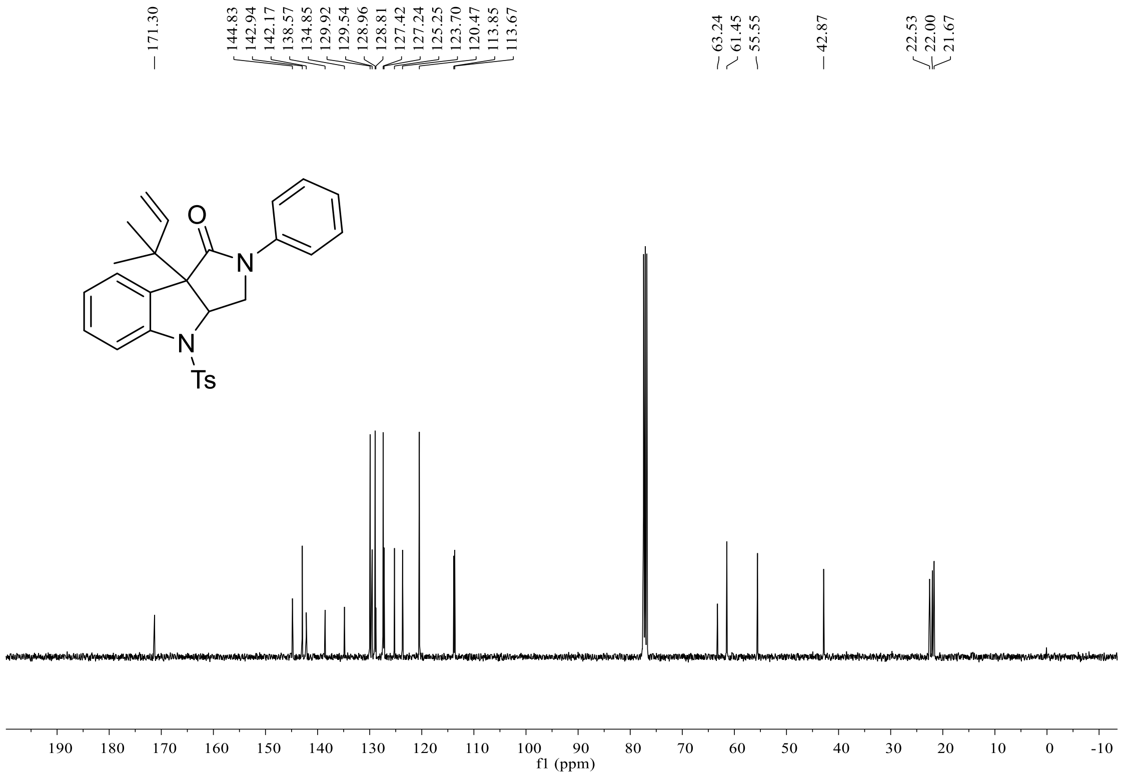

Supplementary Fig. 143. ^{13}C NMR of compound **5a** (100 MHZ, $CDCl_3$)

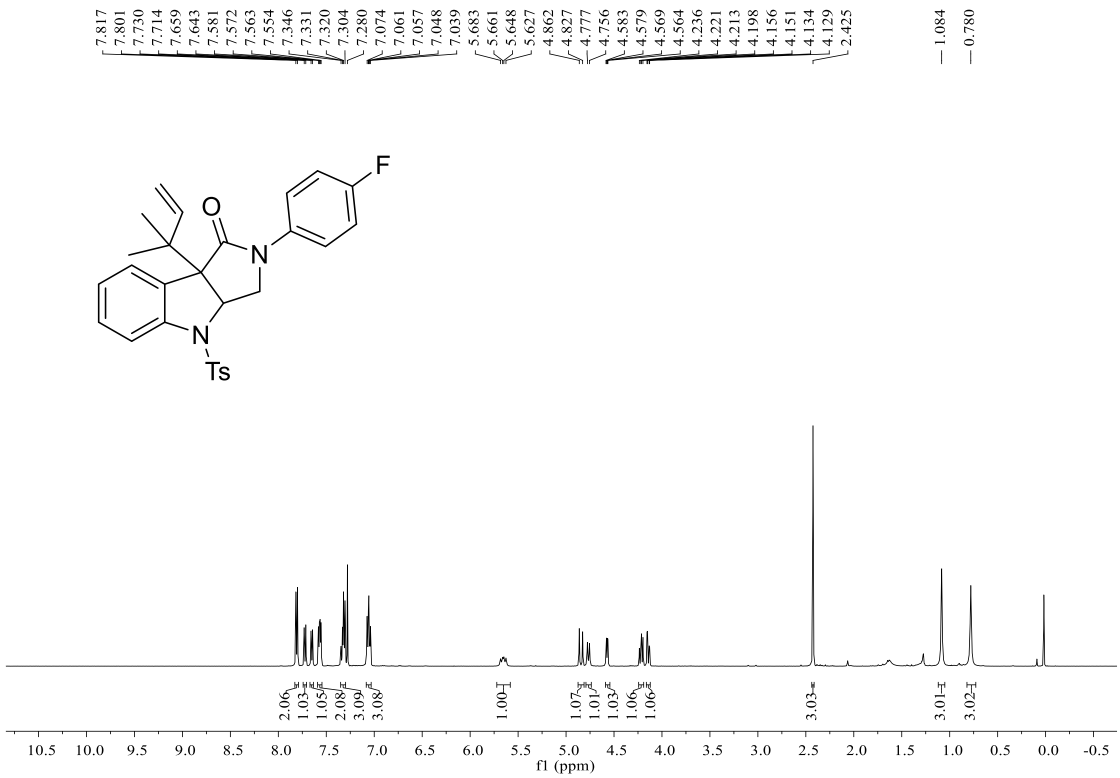

Supplementary Fig. 144. ^{1}H NMR of compound **5b** (500 MHZ, $CDCl_3$)

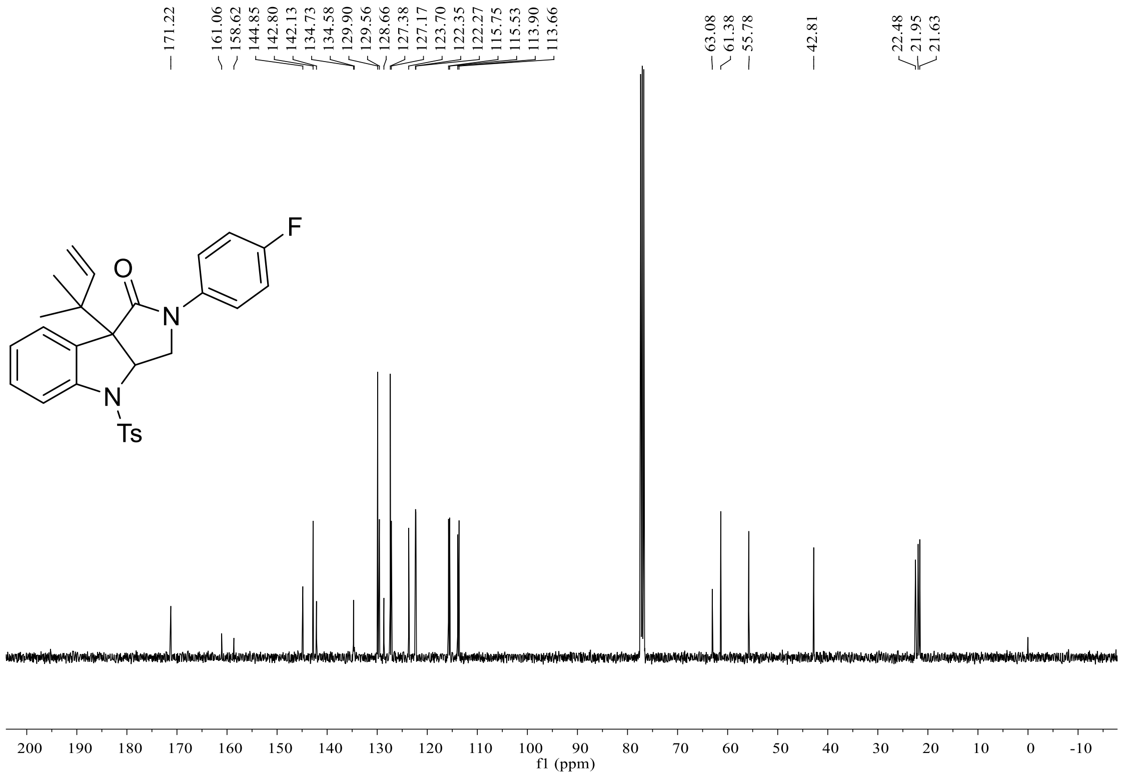

Supplementary Fig. 145. ^{13}C NMR of compound **5b** (125 MHZ, $CDCl_3$)

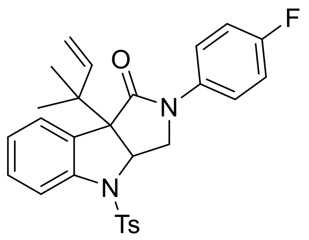

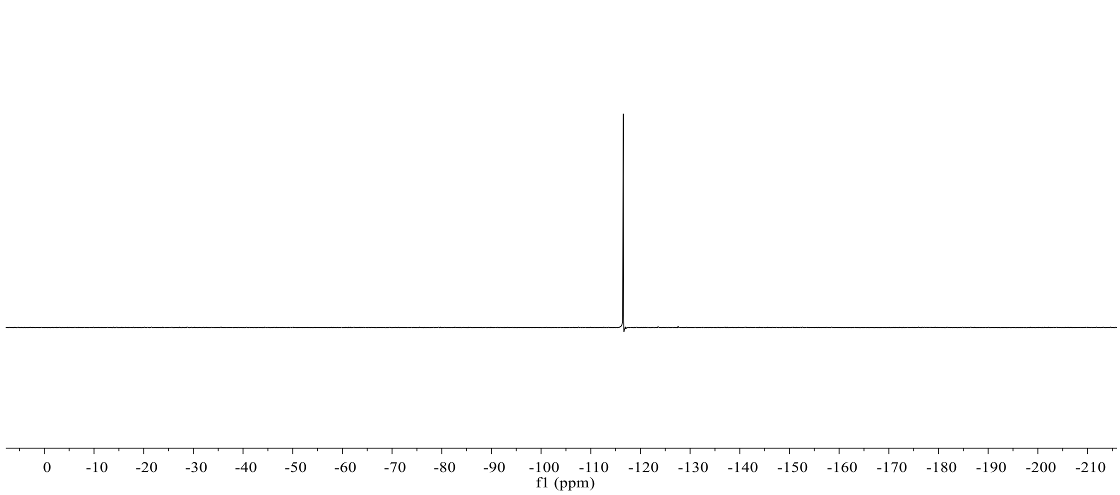

Supplementary Fig. 146. ^{19}F NMR of compound **5b** (376 MHZ, $CDCl_3$)

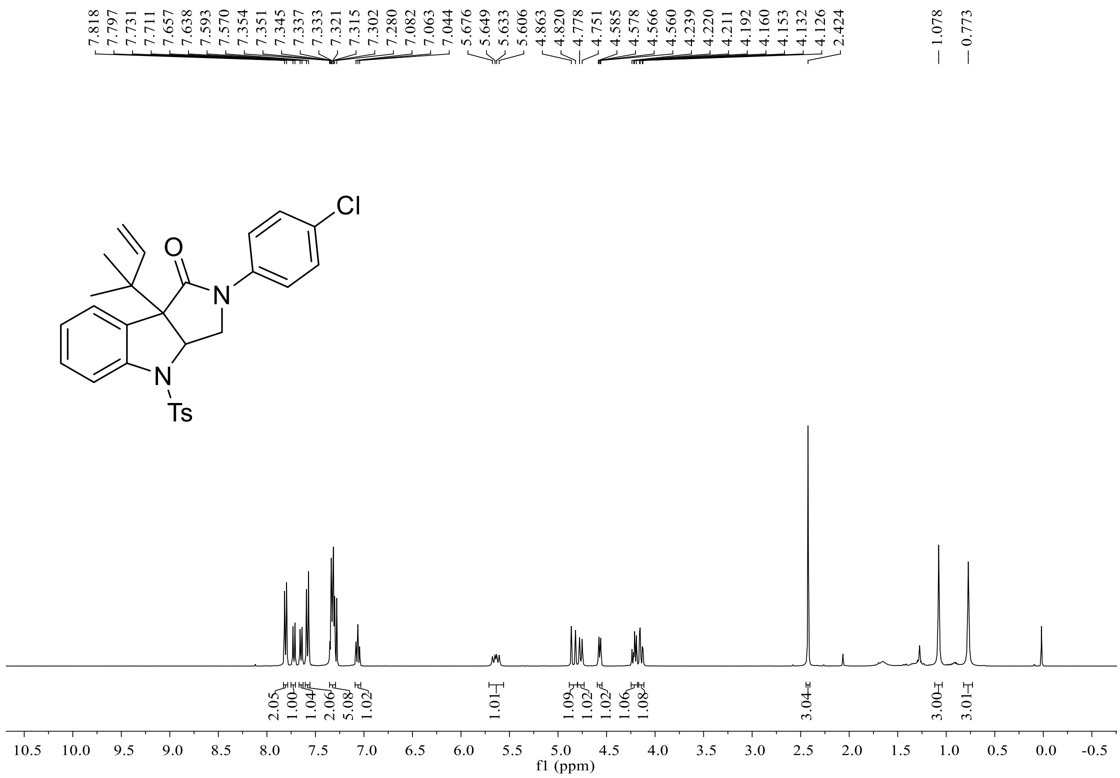

Supplementary Fig. 147. ^{1}H NMR of compound **5c** (400 MHZ, $CDCl_3$)

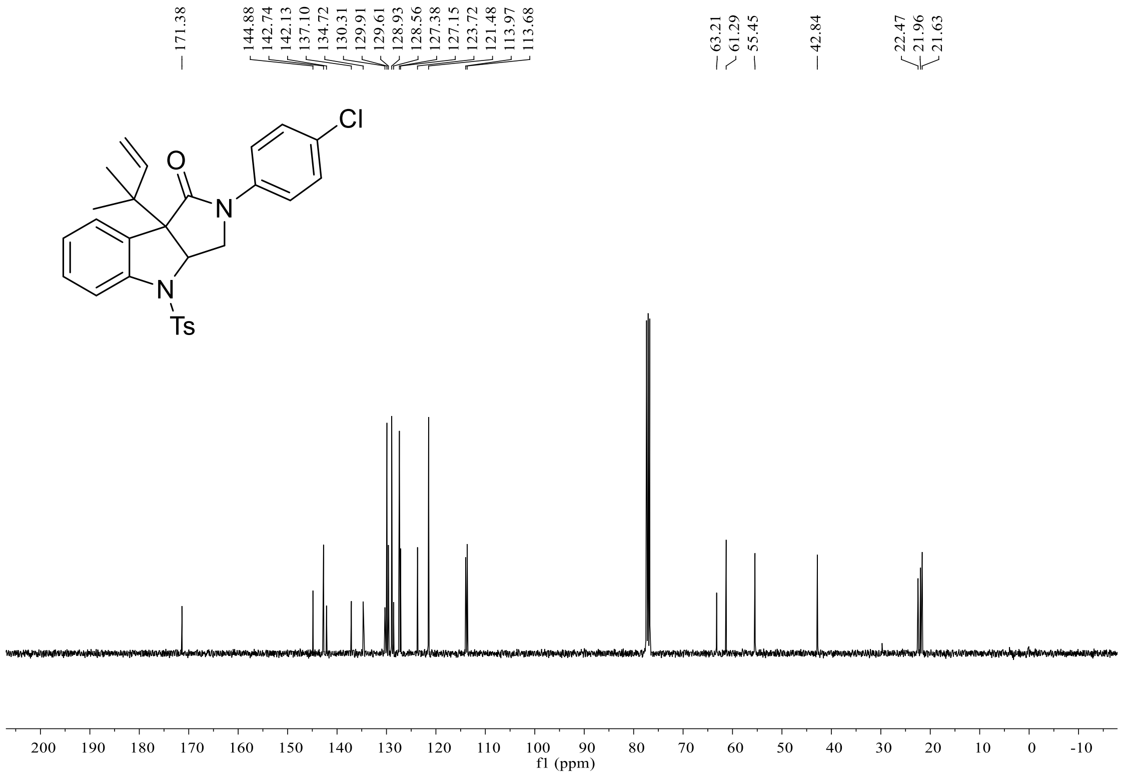

Supplementary Fig. 148. ^{13}C NMR of compound **5c** (100 MHZ, $CDCl_3$)

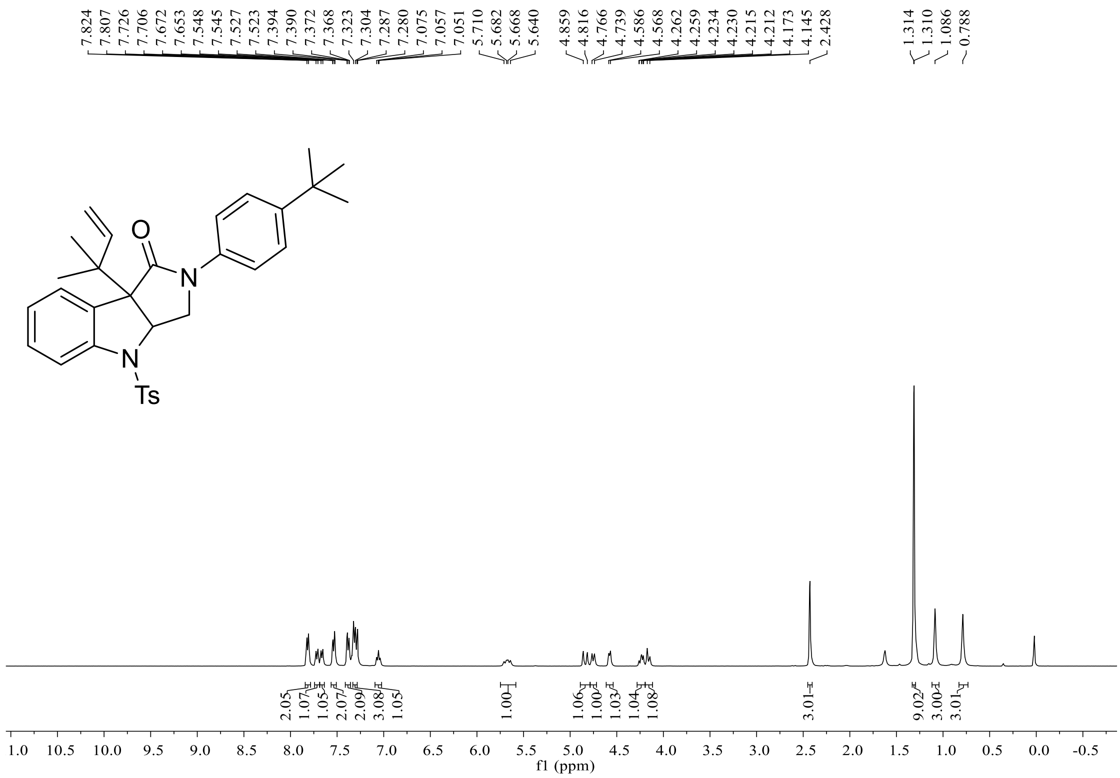

Supplementary Fig. 149. ^{1}H NMR of compound **5d** (400 MHZ, $CDCl_3$)

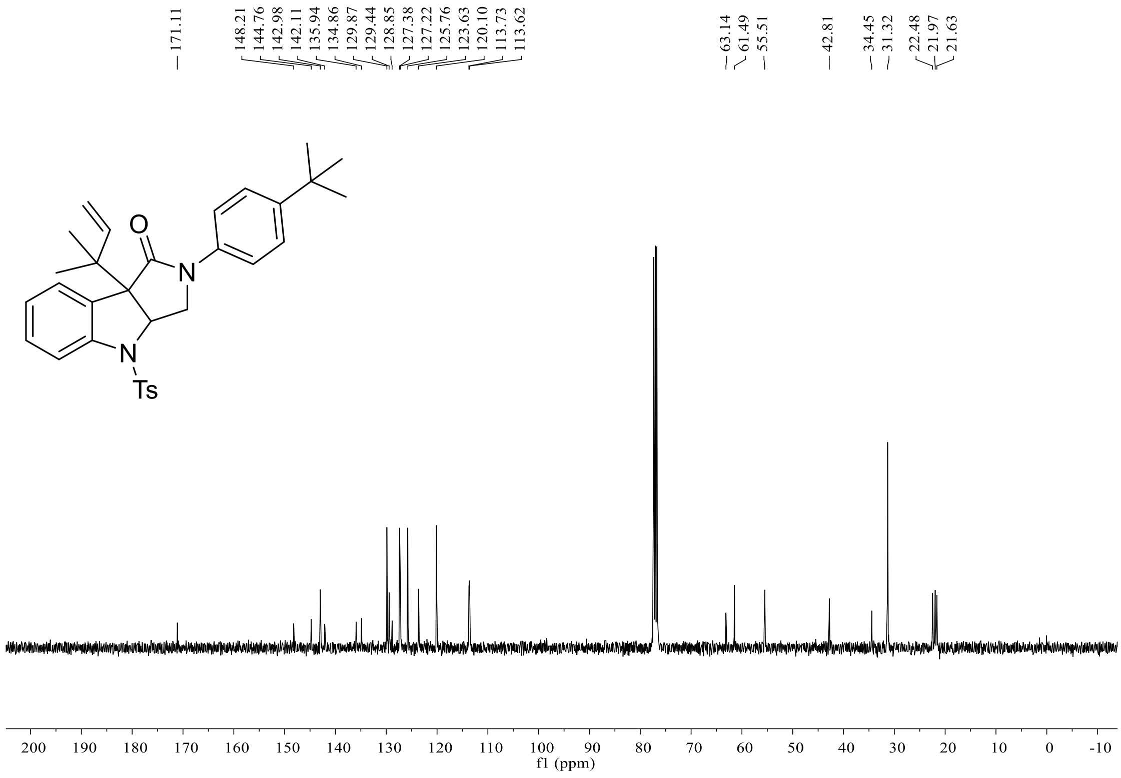

Supplementary Fig. 150. ^{13}C NMR of compound **5d** (100 MHZ, $CDCl_3$)

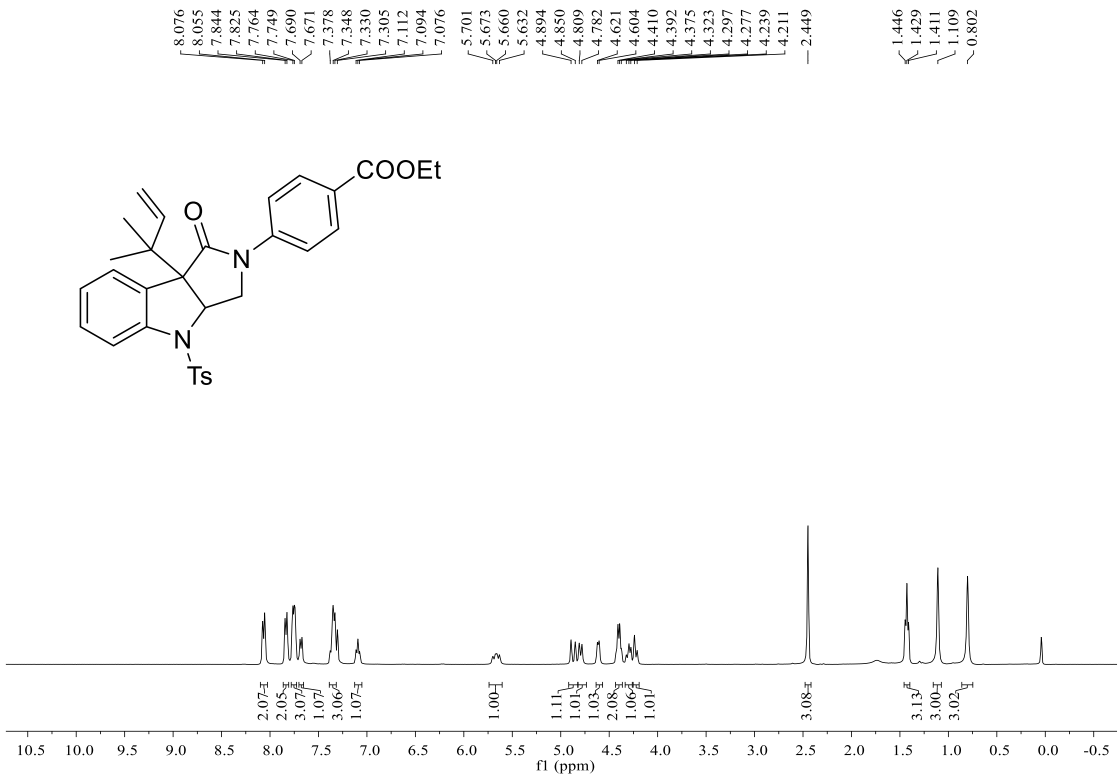

Supplementary Fig. 151. ^{1}H NMR of compound **5e** (400 MHZ, $CDCl_3$)

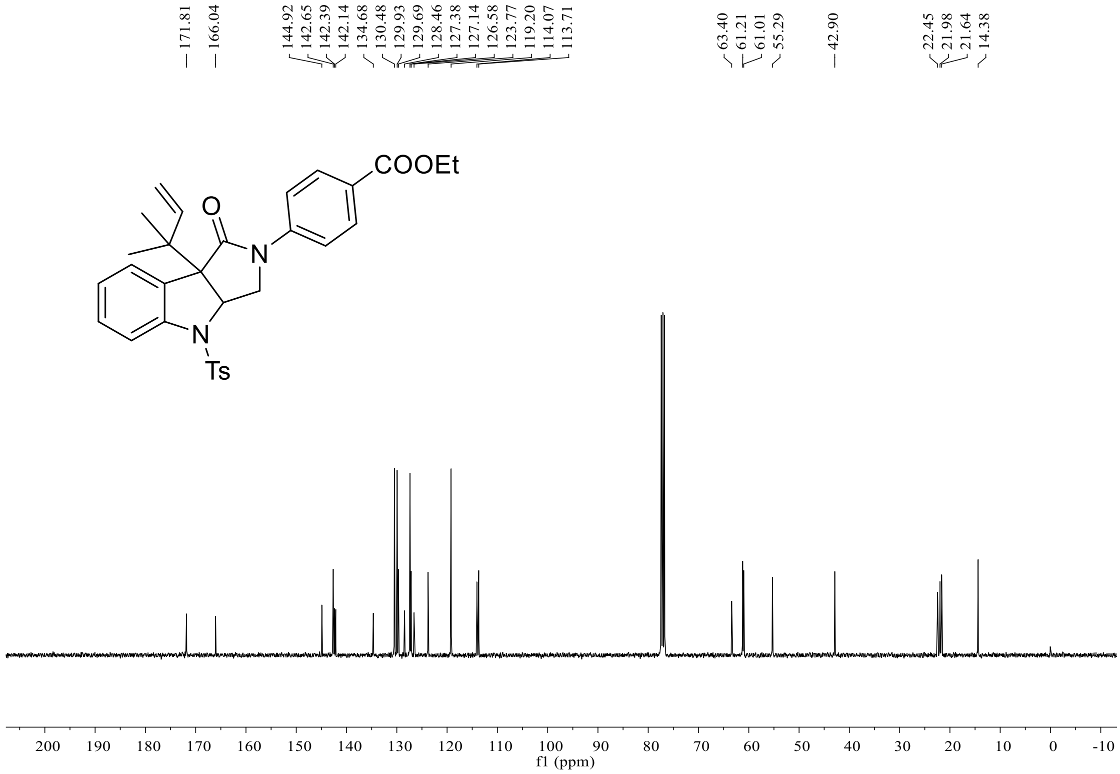

Supplementary Fig. 152. ^{13}C NMR of compound **5e** (100 MHZ, $CDCl_3$)

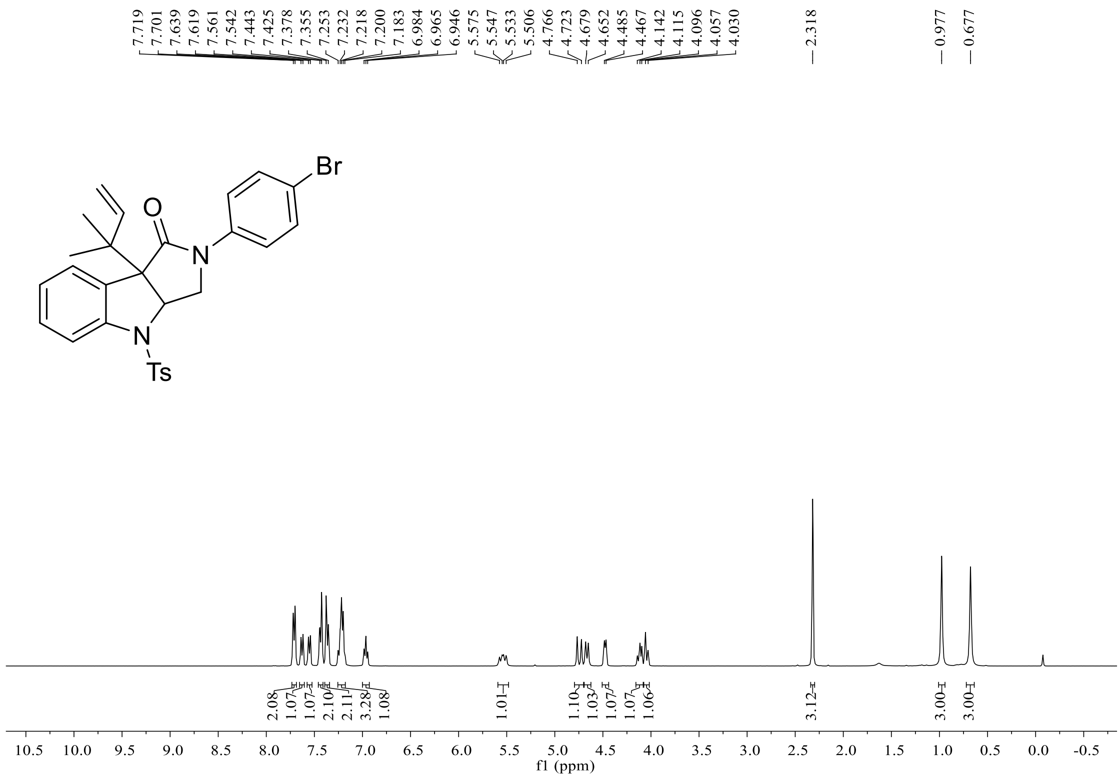

Supplementary Fig. 153. ^{1}H NMR of compound **5f** (400 MHZ, $CDCl_3$)

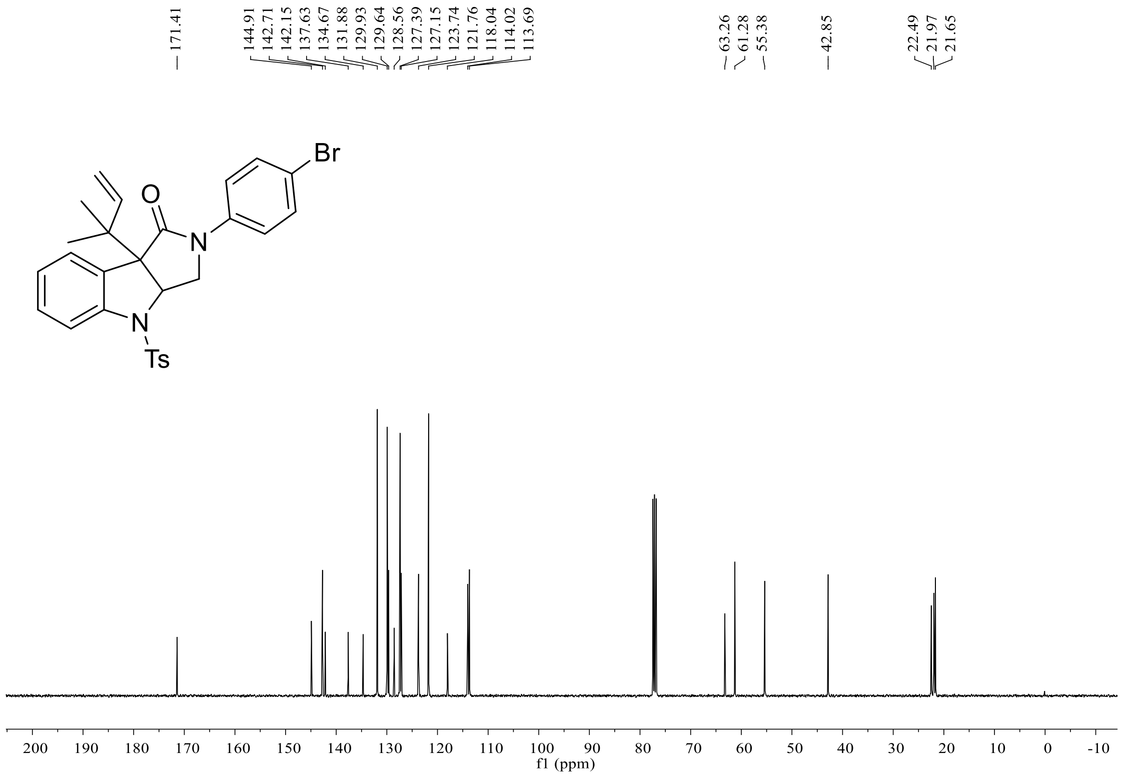

Supplementary Fig. 154. ^{13}C NMR of compound **5f** (100 MHZ, $CDCl_3$)

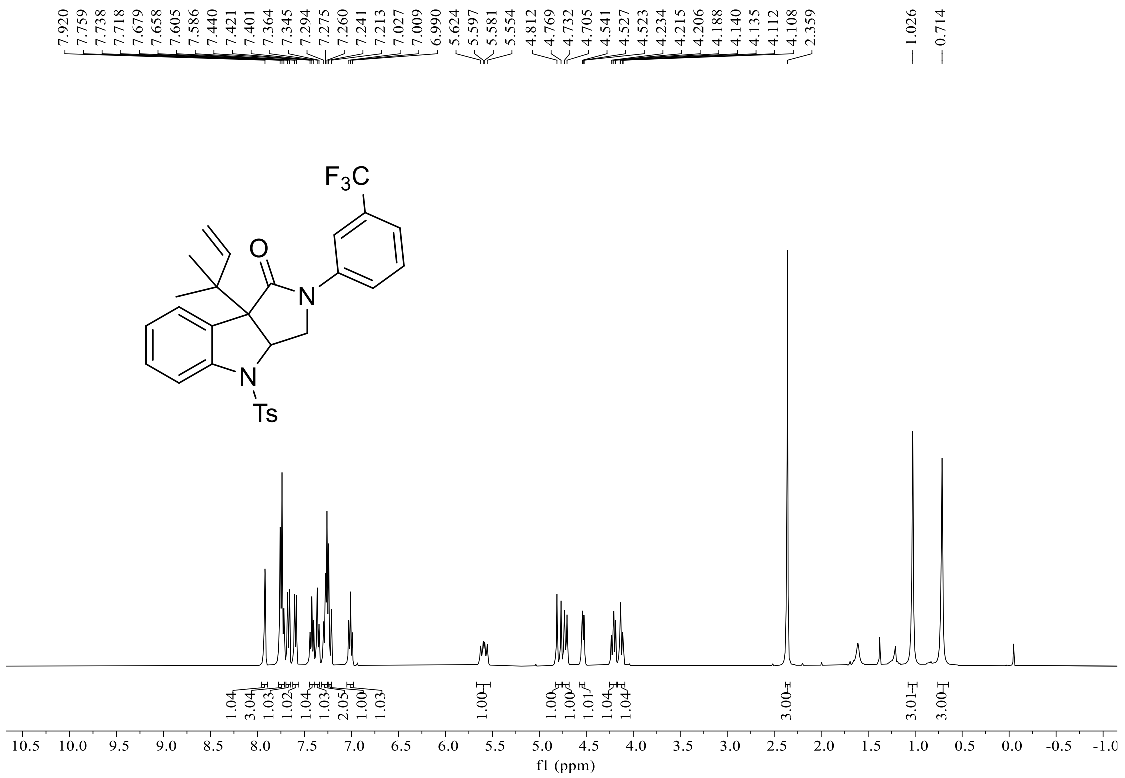

Supplementary Fig. 155. ^{1}H NMR of compound **5g** (400 MHZ, $CDCl_3$)

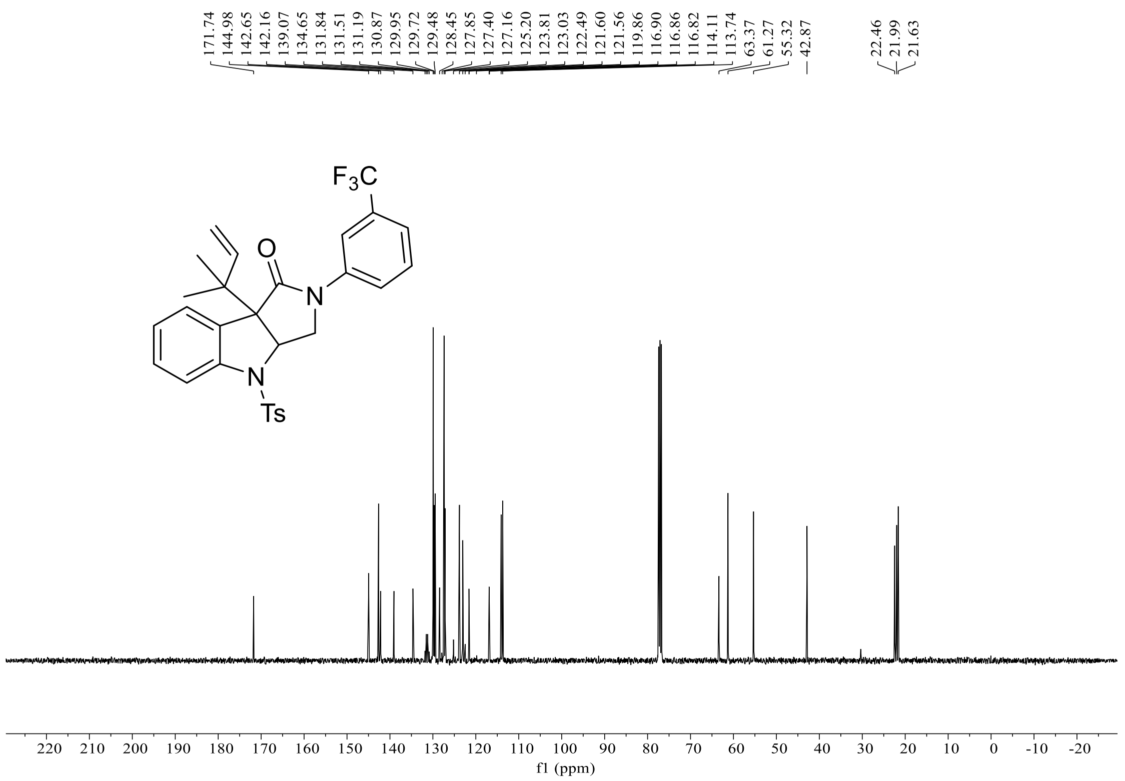

Supplementary Fig. 156. ^{13}C NMR of compound **5g** (100 MHZ, $CDCl_3$)

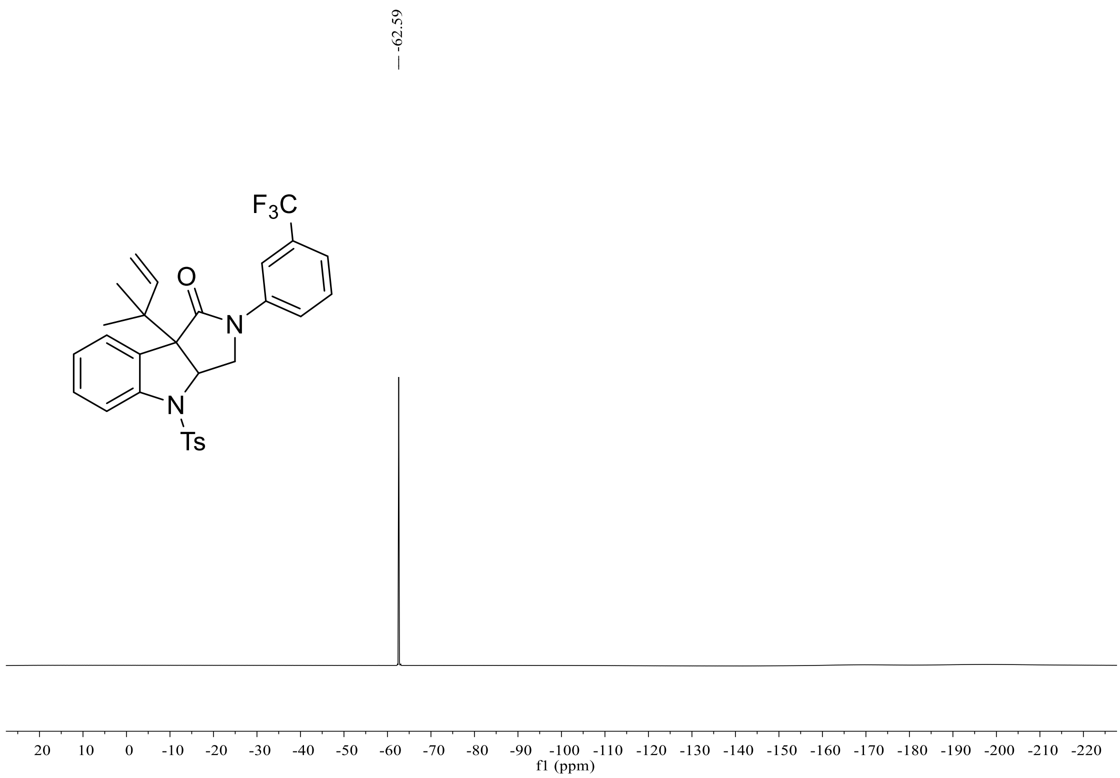

Supplementary Fig. 157. ^{19}F NMR of compound **5g** (376 MHZ, $CDCl_3$)

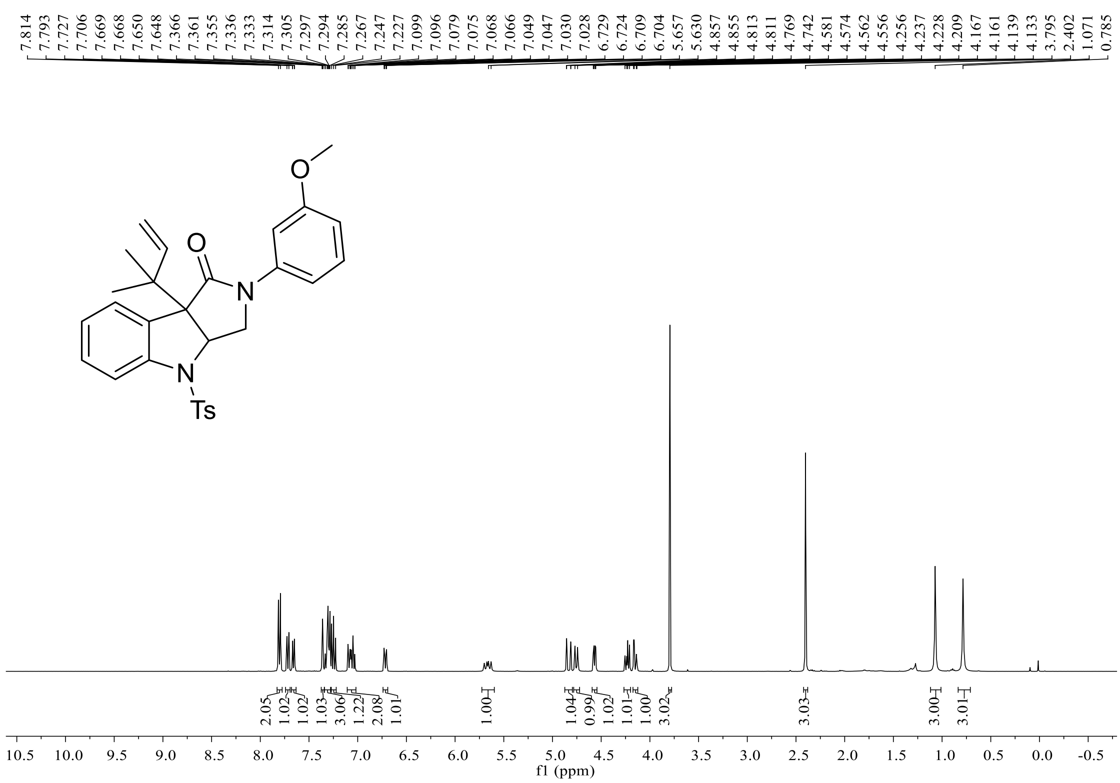

Supplementary Fig. 158. ^{1}H NMR of compound **5h** (400 MHZ, $CDCl_3$)

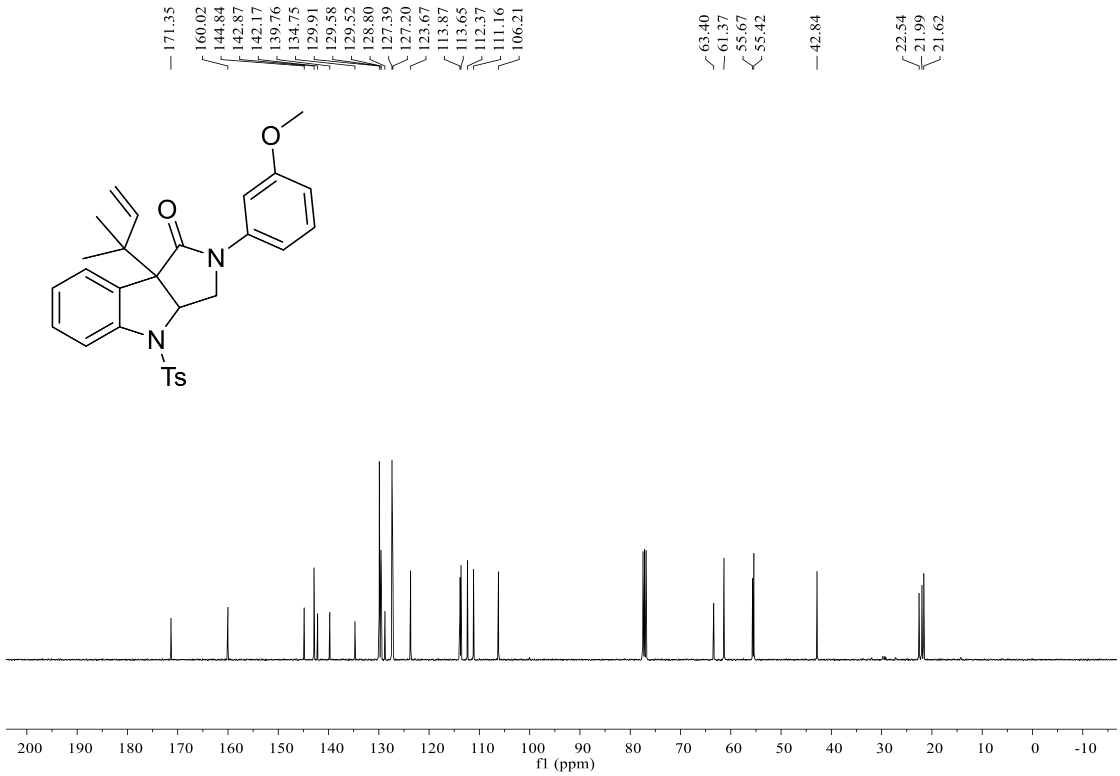

Supplementary Fig. 159. ^{13}C NMR of compound **5h** (100 MHZ, $CDCl_3$)

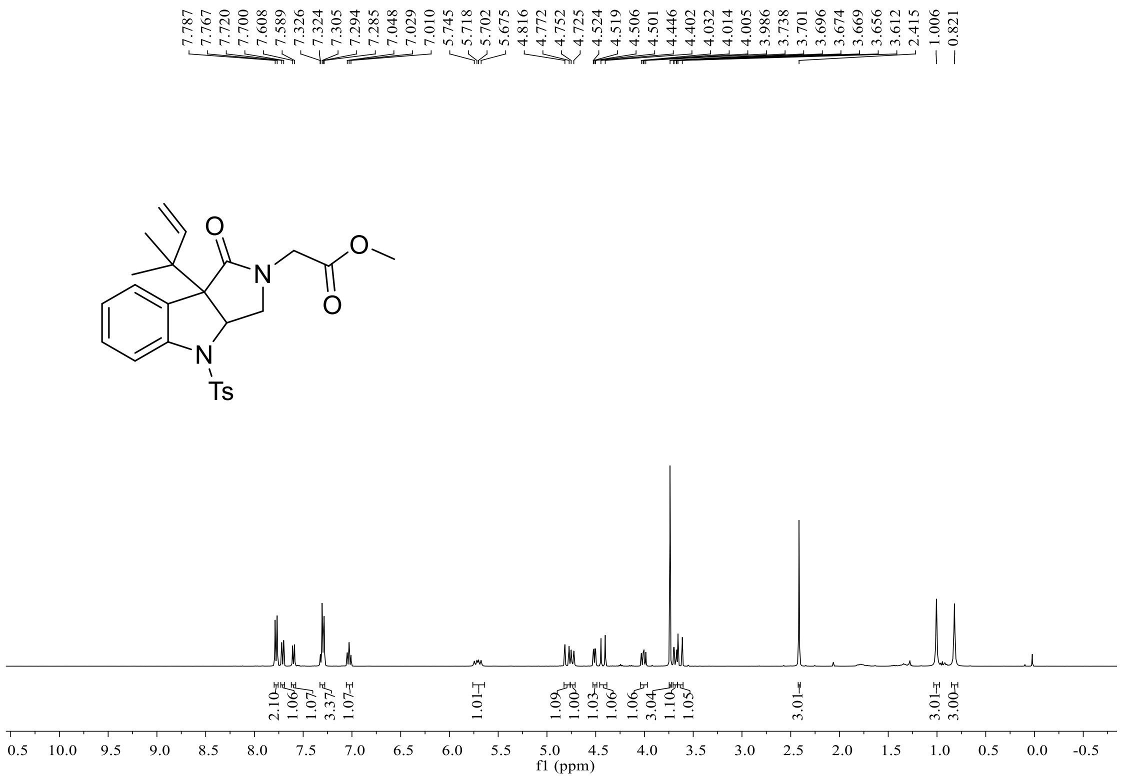

Supplementary Fig. 160. ^{1}H NMR of compound **5i** (400 MHZ, $CDCl_3$)

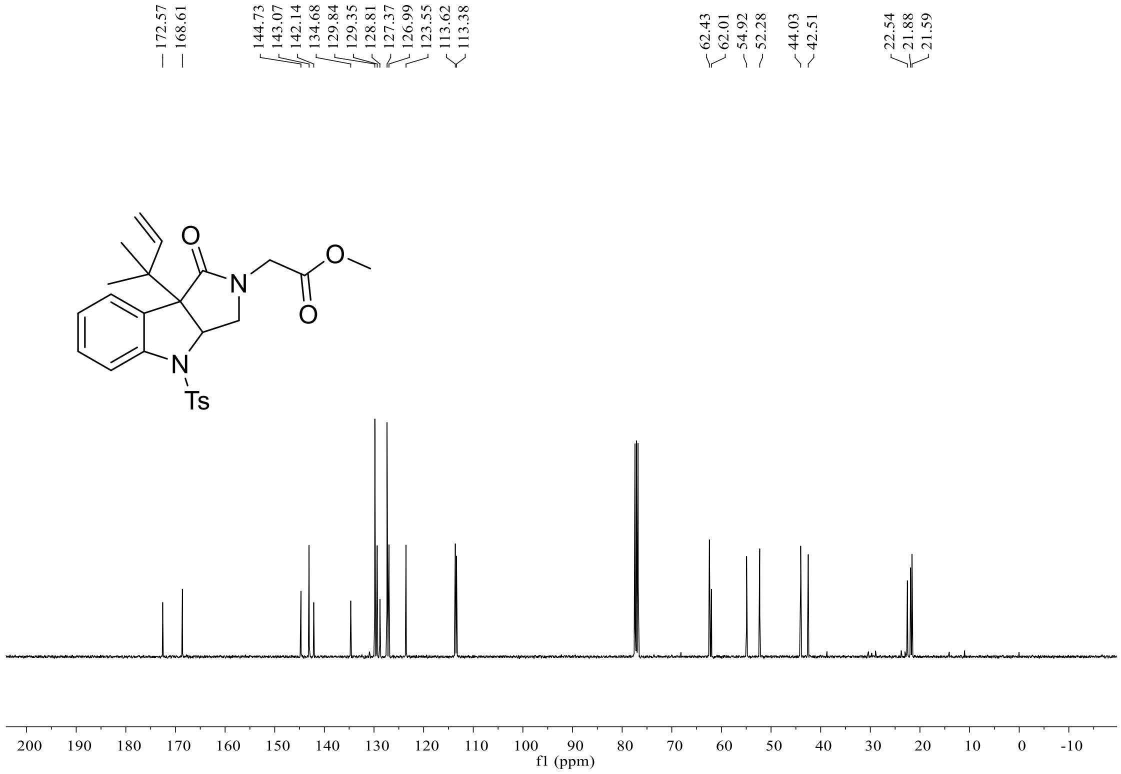

Supplementary Fig. 161. ^{13}C NMR of compound **5i** (100 MHZ, $CDCl_3$)

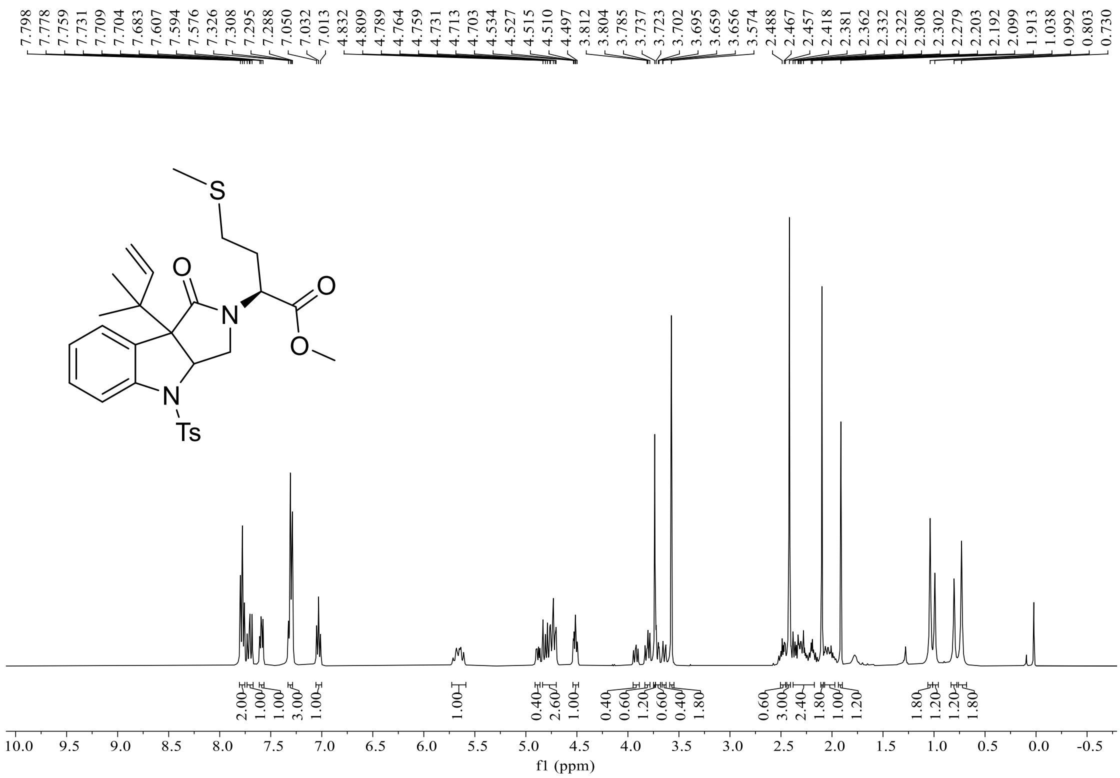

Supplementary Fig. 162. ^{1}H NMR of compound **5j** (400 MHZ, $CDCl_3$)

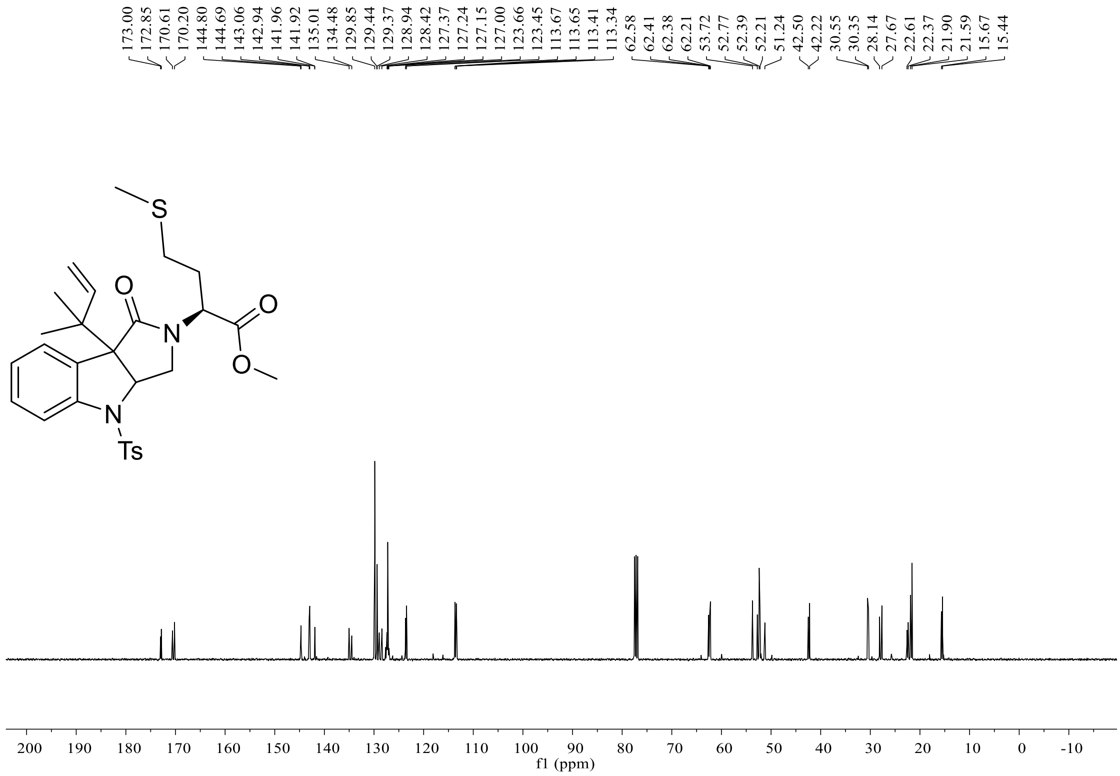

Supplementary Fig. 163. ^{13}C NMR of compound **5j** (100 MHZ, $CDCl_3$)

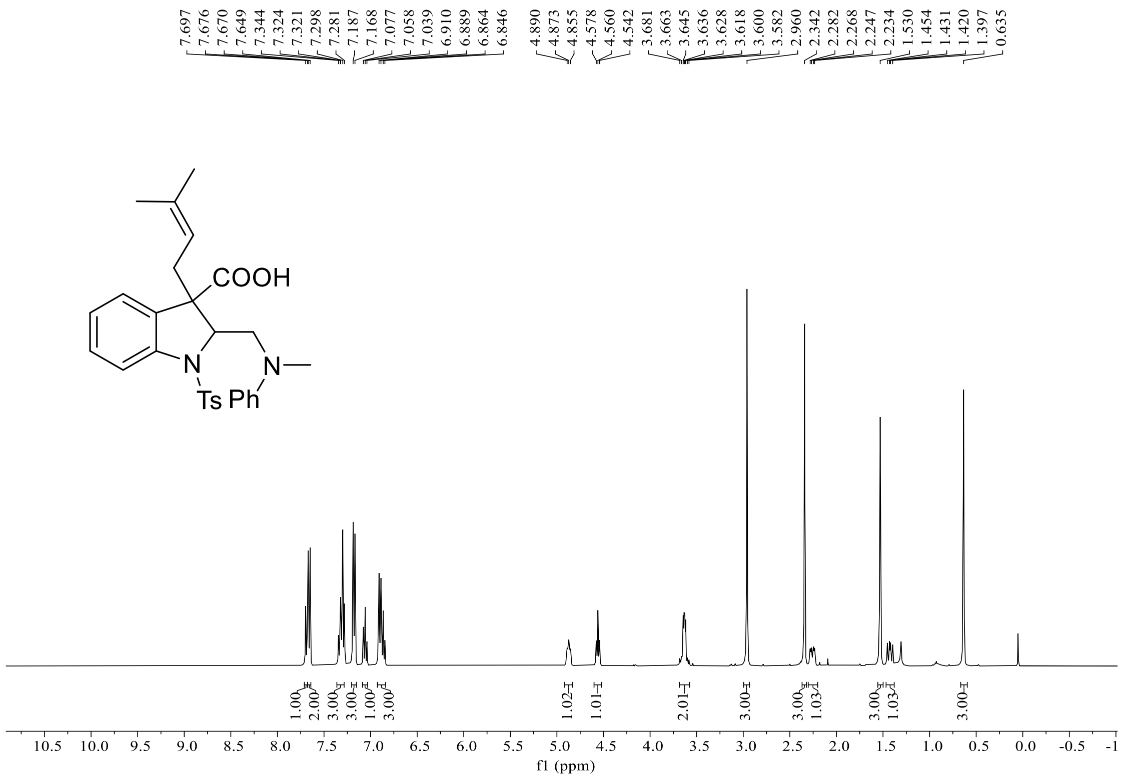

Supplementary Fig. 164. ^{1}H NMR of compound **7a** (400 MHZ, $CDCl_3$)

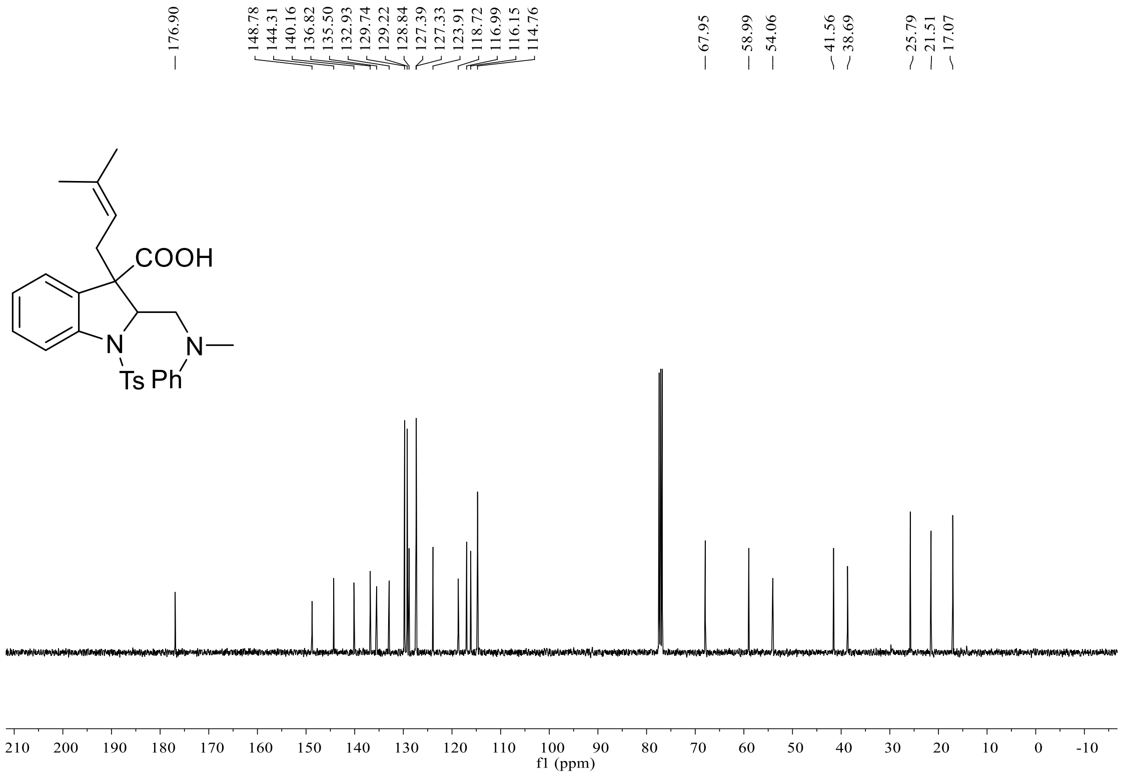

Supplementary Fig. 165. ^{13}C NMR of compound **7a** (100 MHZ, $CDCl_3$)

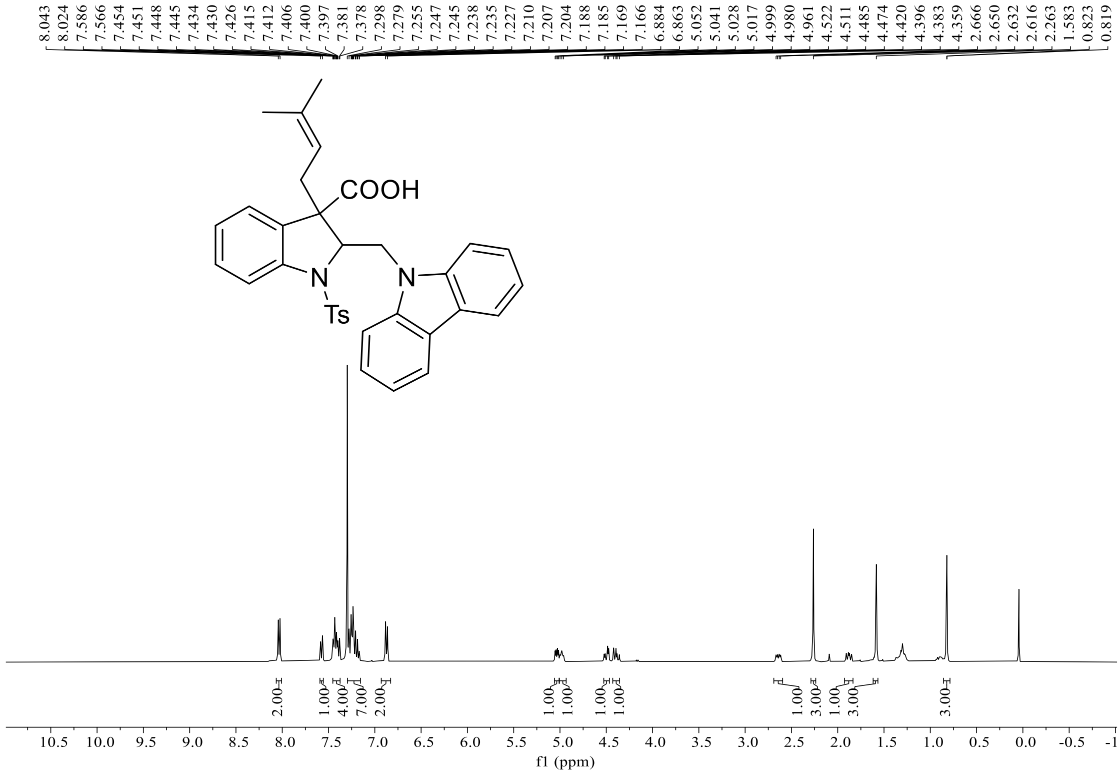

Supplementary Fig. 166. ^{1}H NMR of compound **7b** (400 MHZ, $CDCl_3$)

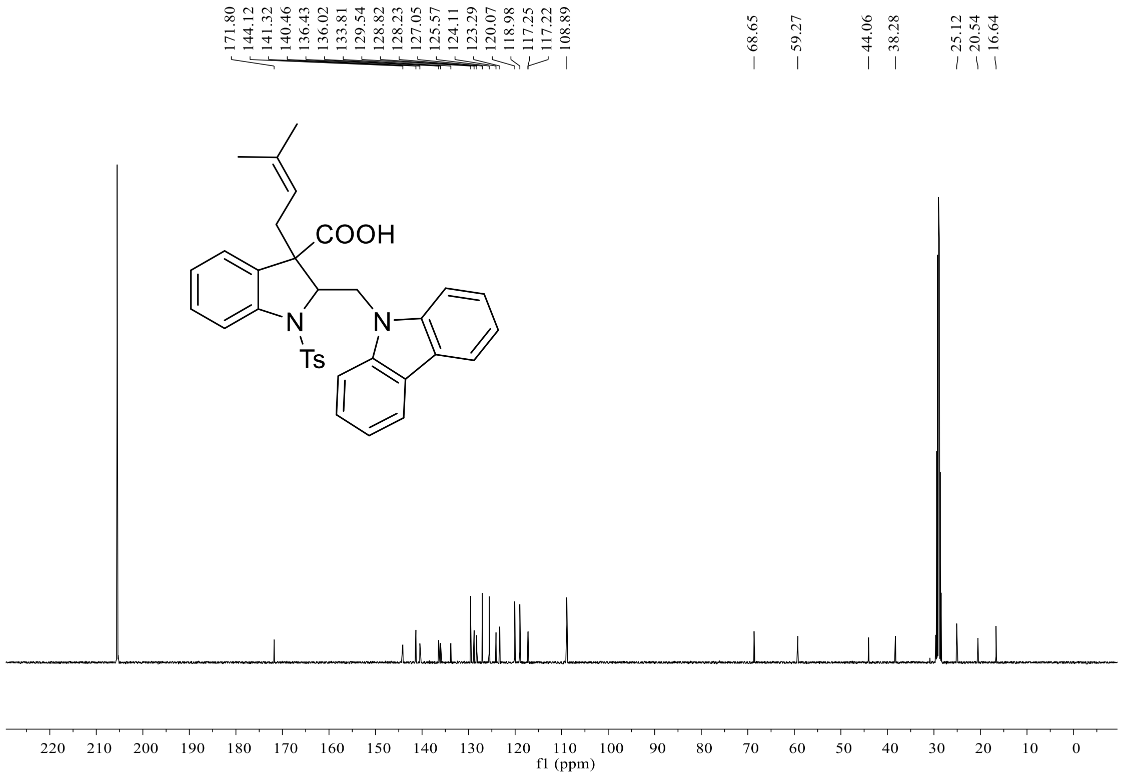

Supplementary Fig. 167. ^{13}C NMR of compound **7b** (100 MHZ, $CDCl_3$)

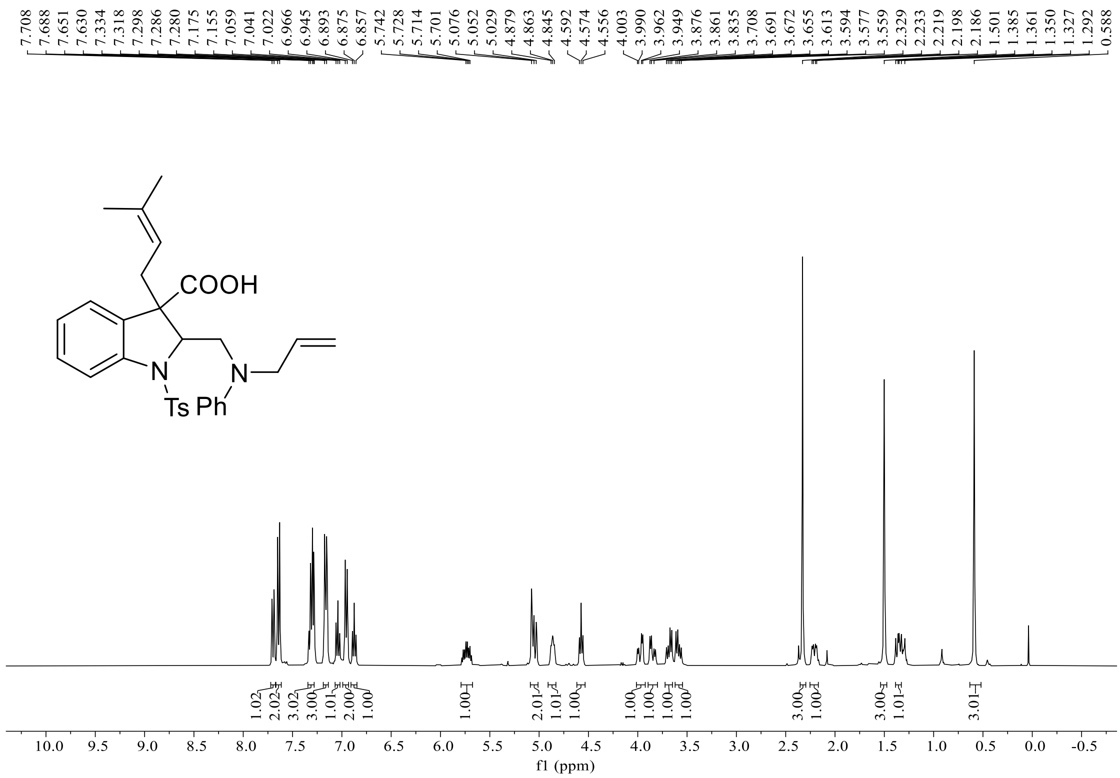

Supplementary Fig. 168. ^{1}H NMR of compound **7c** (400 MHZ, $CDCl_3$)

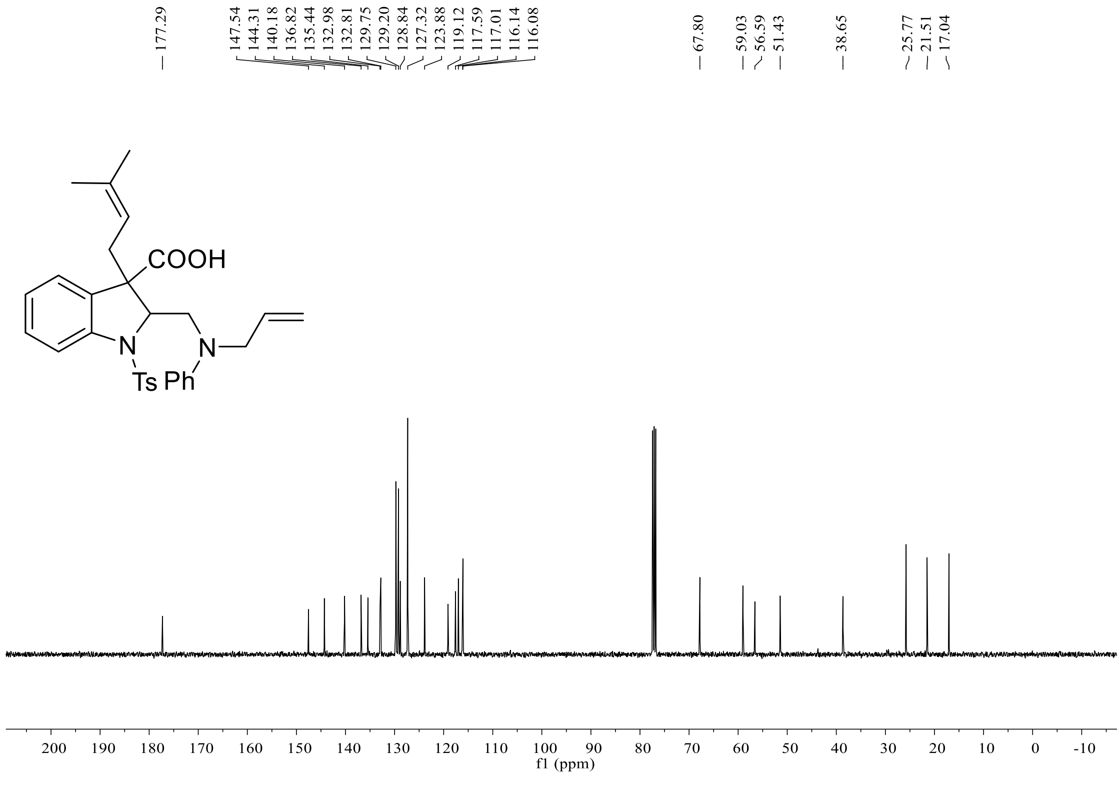

Supplementary Fig. 169. ^{13}C NMR of compound **7c** (100 MHZ, $CDCl_3$)

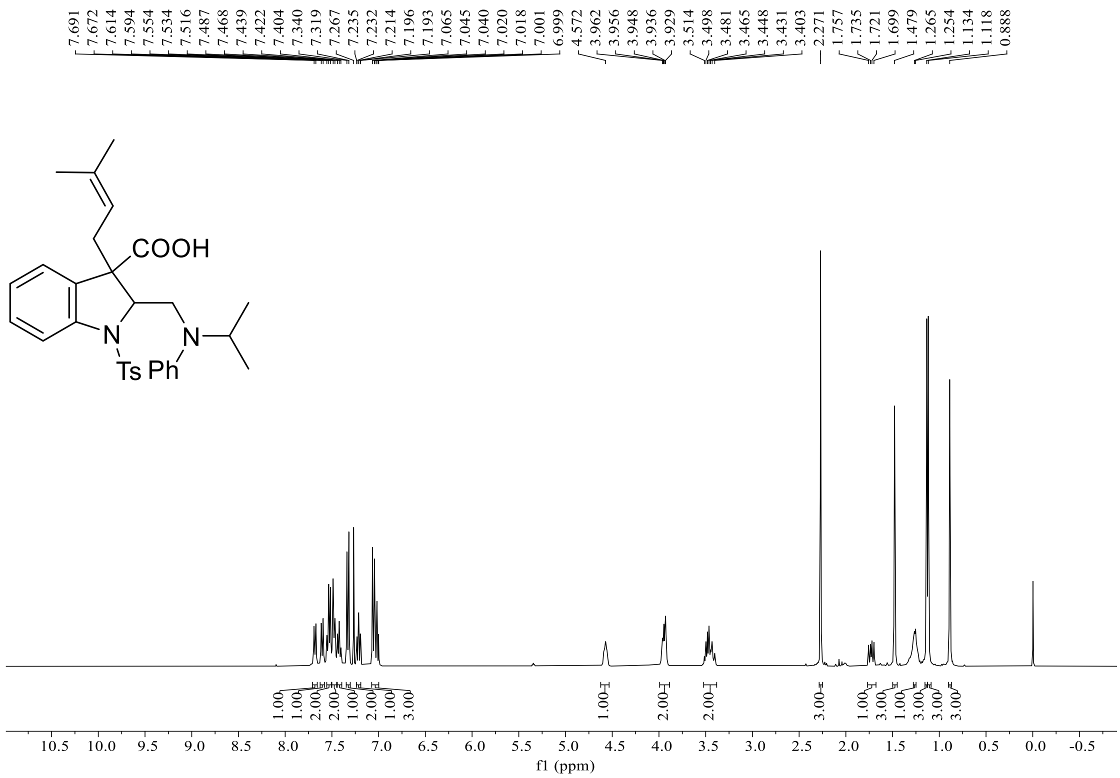

Supplementary Fig. 170. ^{1}H NMR of compound **7d** (400 MHZ, $CDCl_3$)

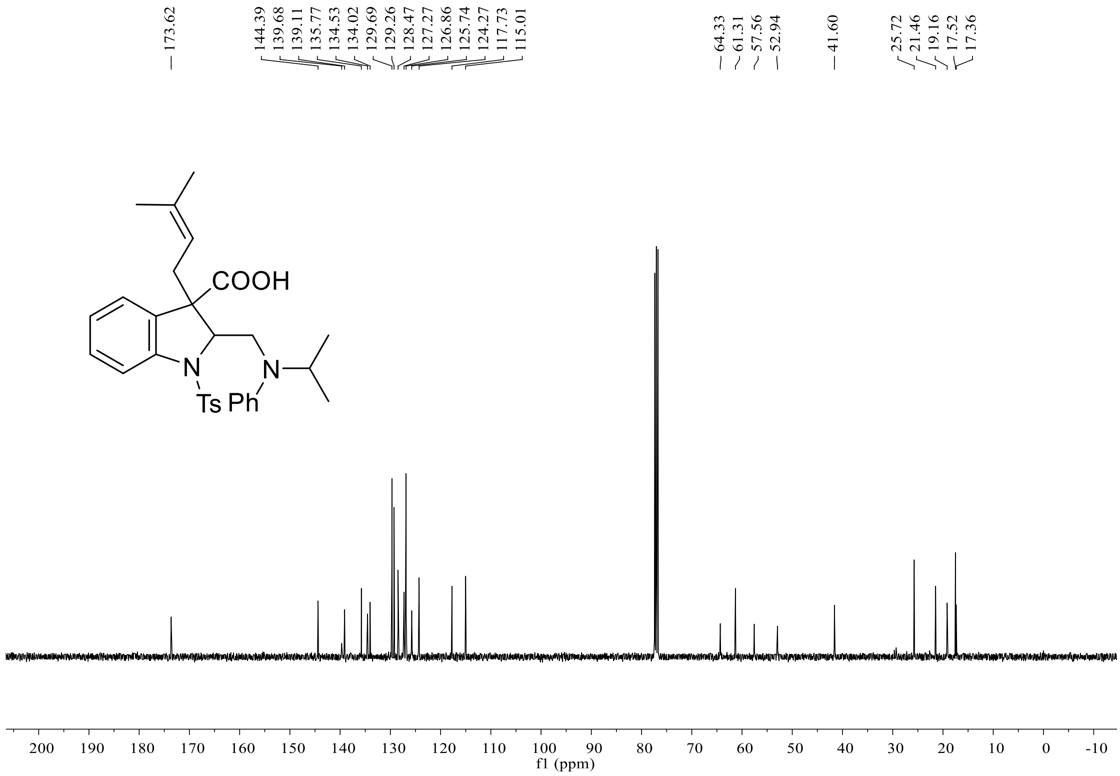

Supplementary Fig. 171. ^{13}C NMR of compound **7d** (100 MHZ, $CDCl_3$)

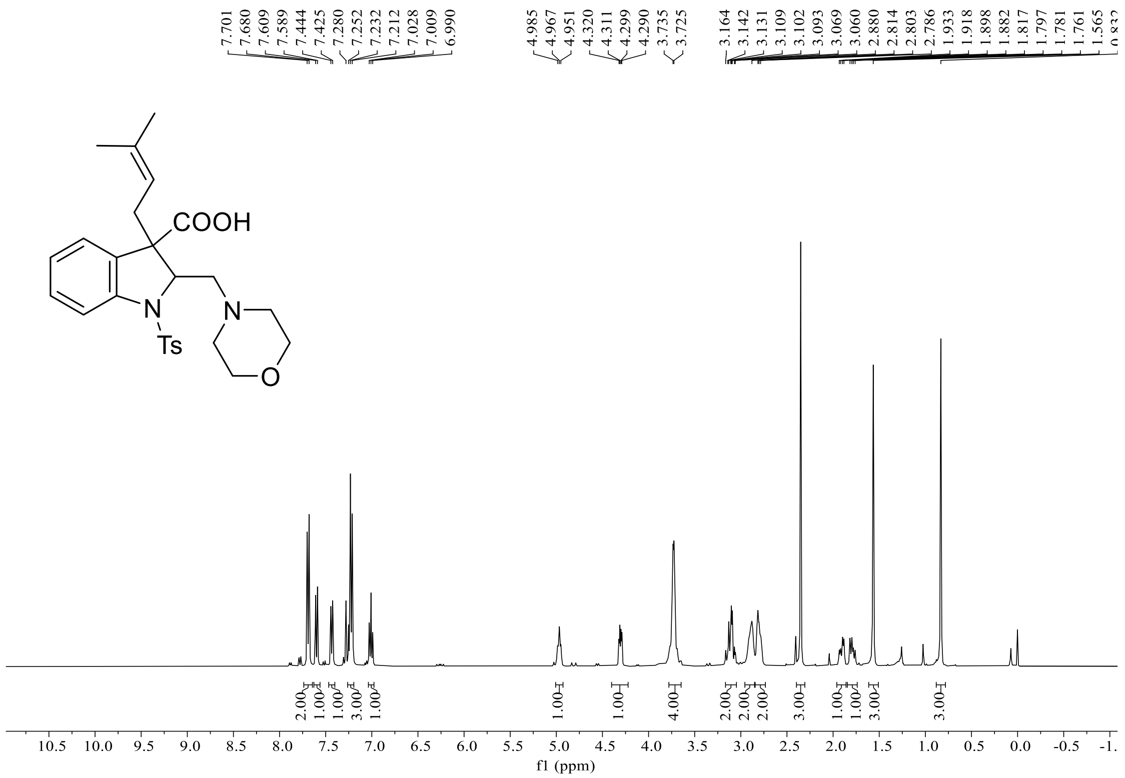

Supplementary Fig. 172. ^{1}H NMR of compound **7e** (400 MHZ, $CDCl_3$)

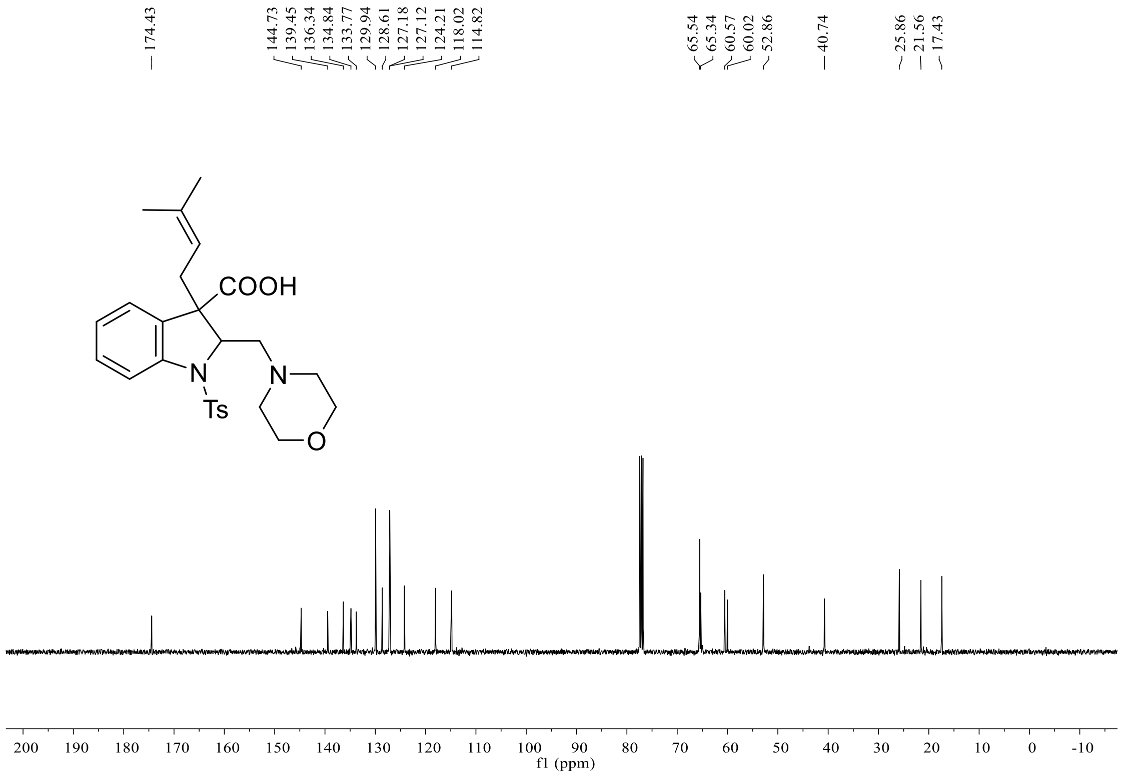

Supplementary Fig. 173. ^{13}C NMR of compound **7e** (100 MHZ, $CDCl_3$)

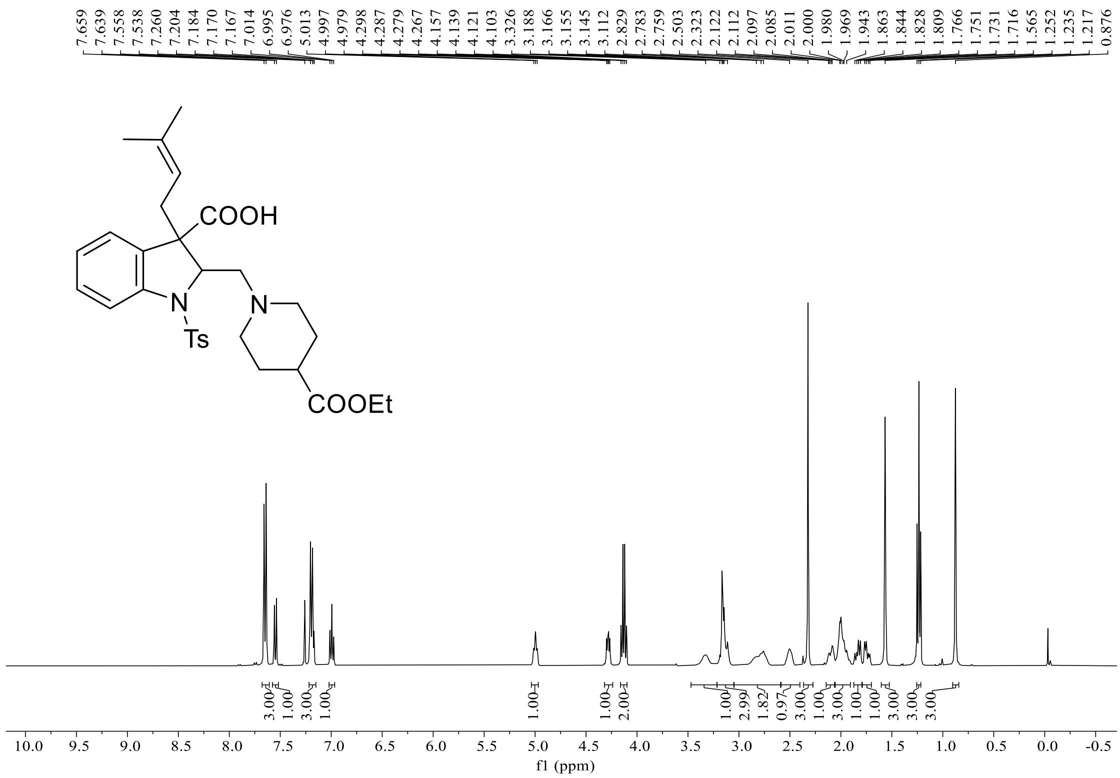

Supplementary Fig. 174. ^{1}H NMR of compound **7f** (400 MHZ, $CDCl_3$)

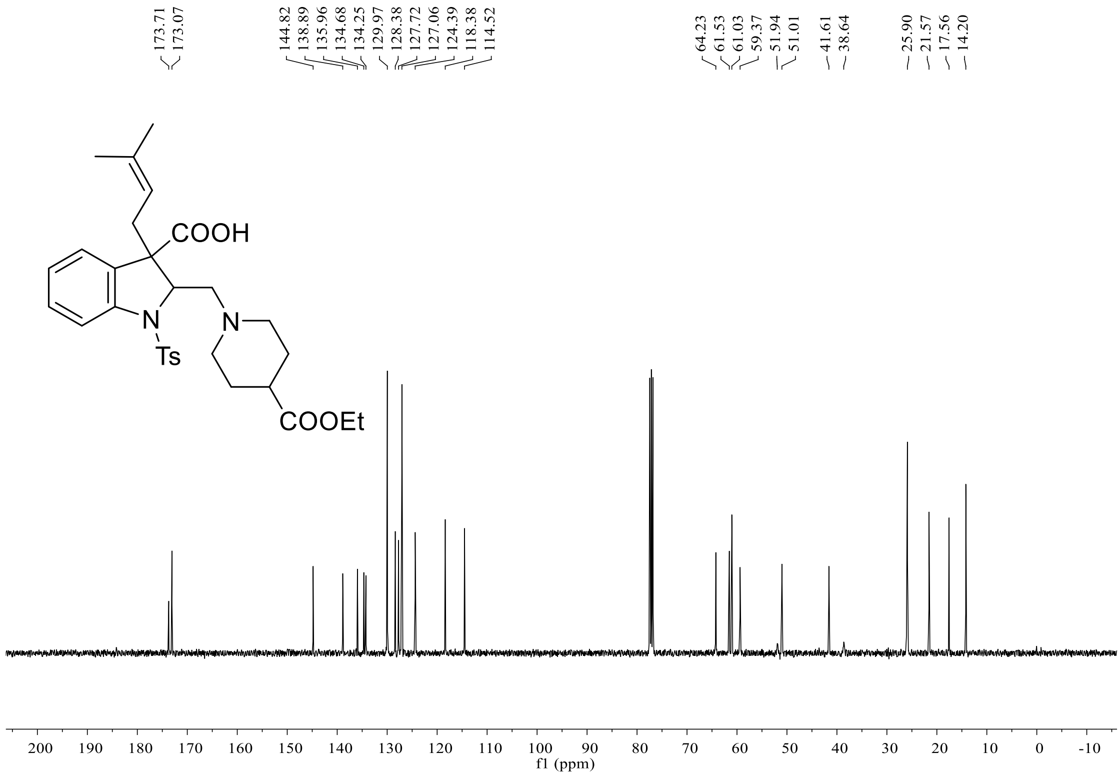

Supplementary Fig. 175. ^{13}C NMR of compound **7f** (100 MHZ, $CDCl_3$)

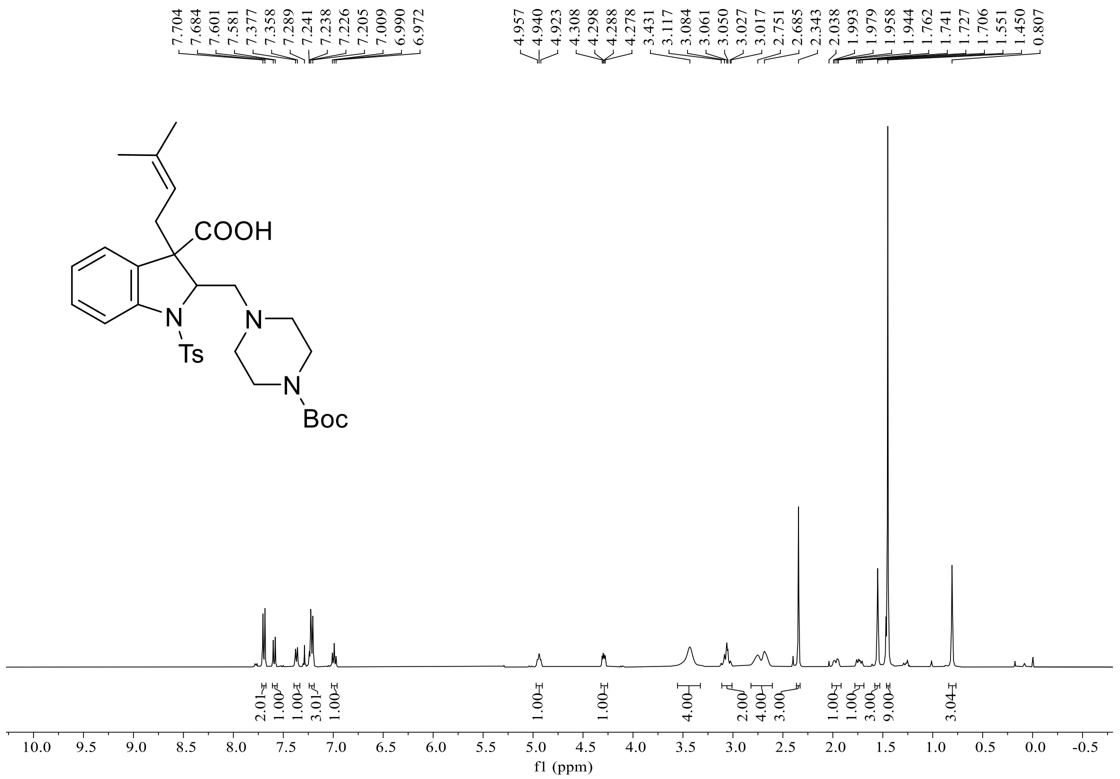

Supplementary Fig. 176. ^{1}H NMR of compound **7g** (400 MHZ, $CDCl_3$)

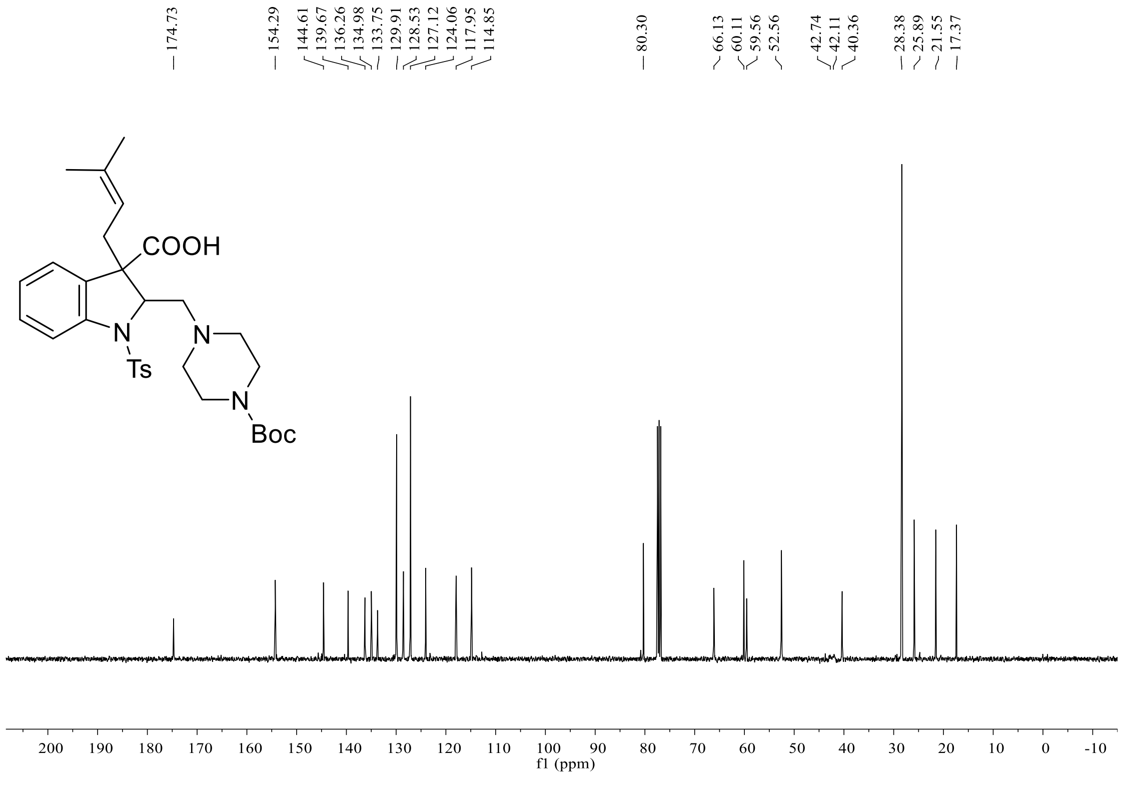

Supplementary Fig. 177. ^{13}C NMR of compound **7g** (100 MHZ, $CDCl_3$)

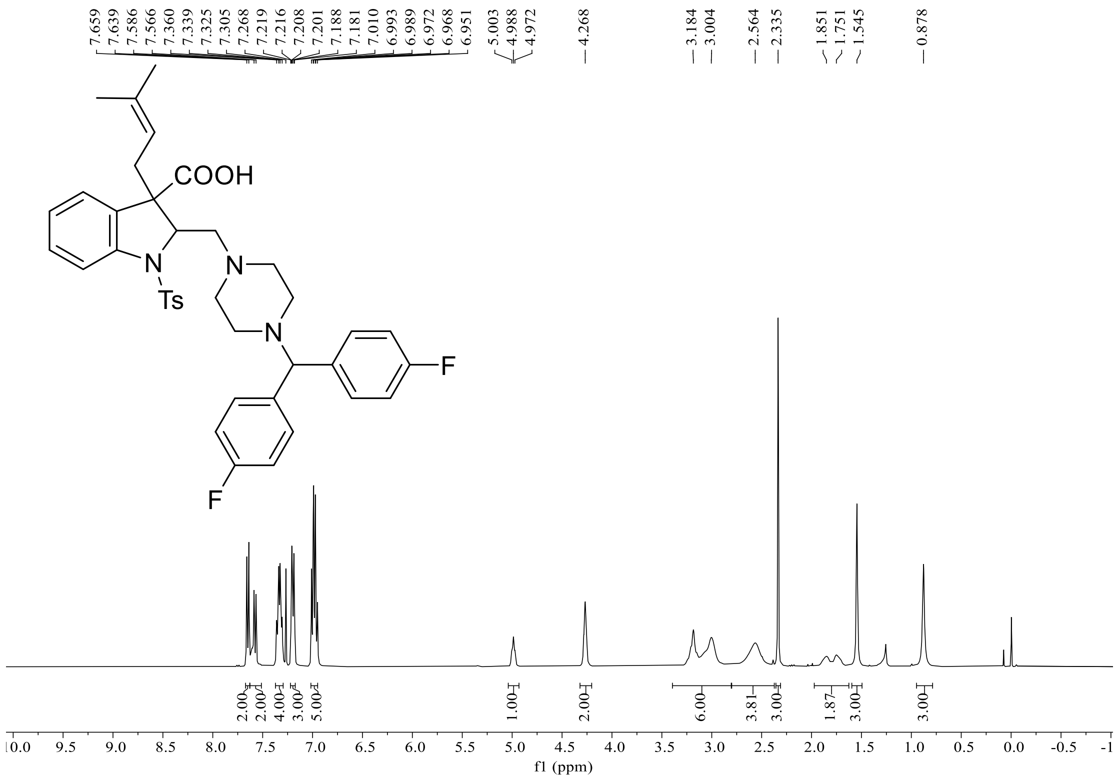

Supplementary Fig. 178. ^{1}H NMR of compound **7h** (400 MHZ, $CDCl_3$)

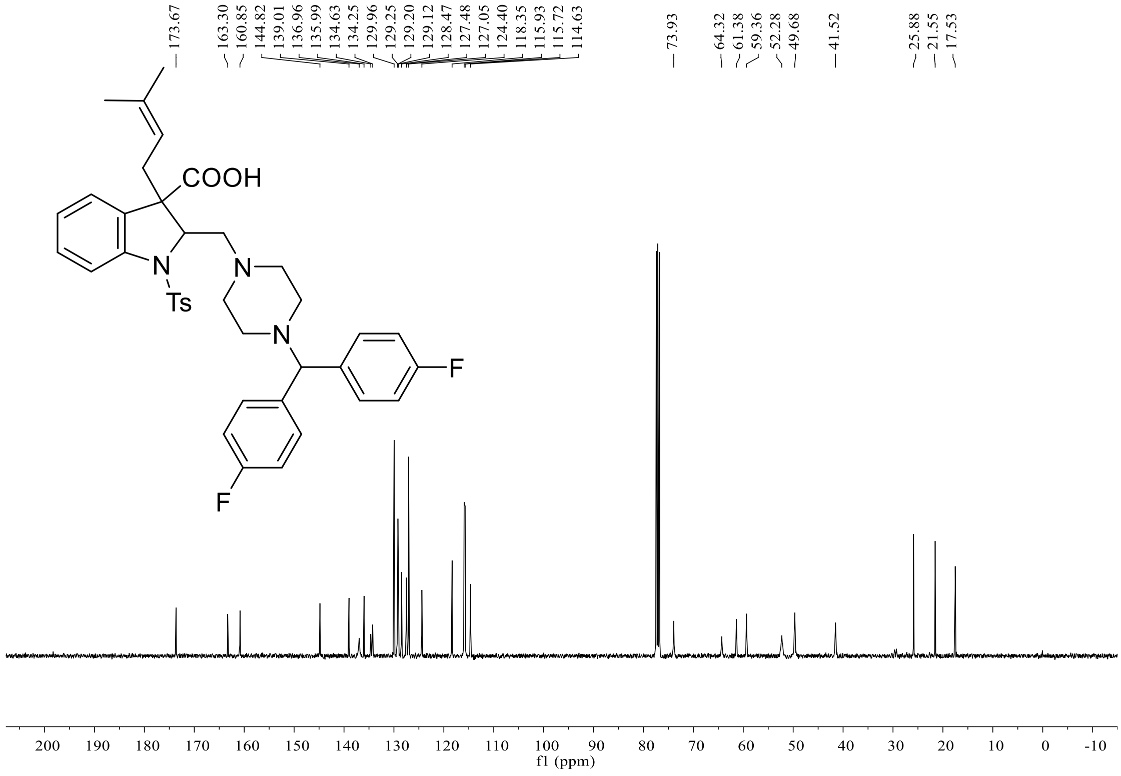

Supplementary Fig. 179. ^{13}C NMR of compound **7h** (100 MHZ, $CDCl_3$)

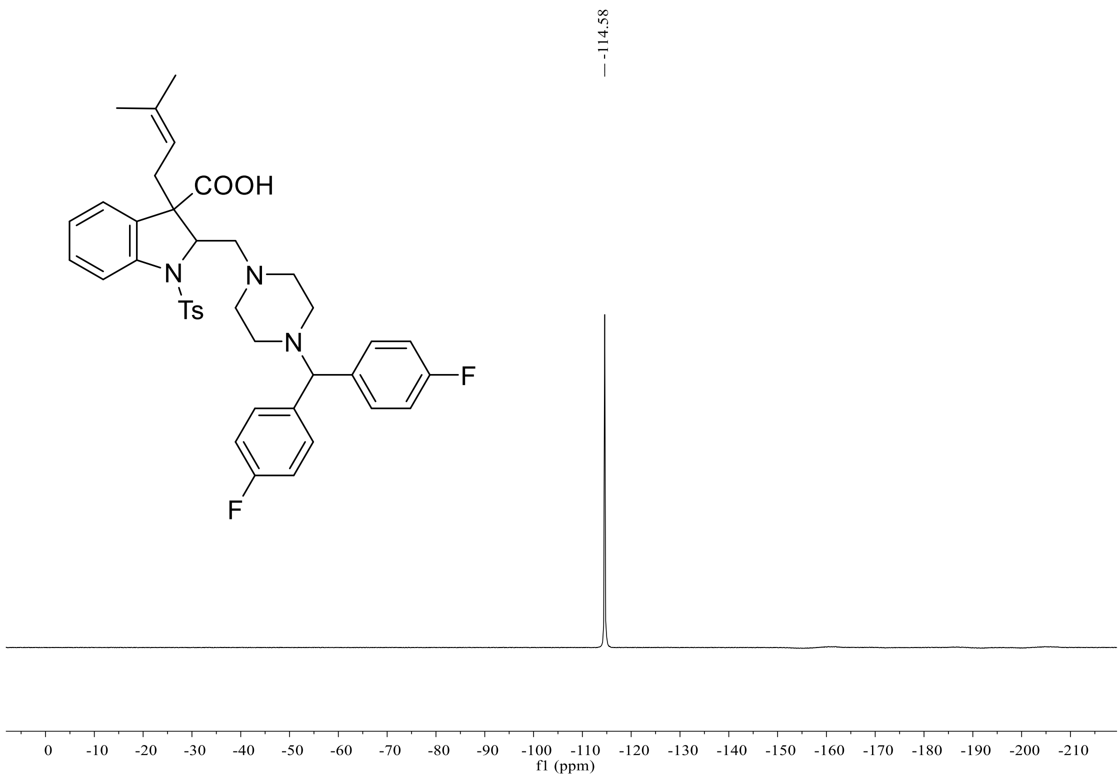

Supplementary Fig. 180. ^{19}F NMR of compound **7h** (400 MHZ, $CDCl_3$)

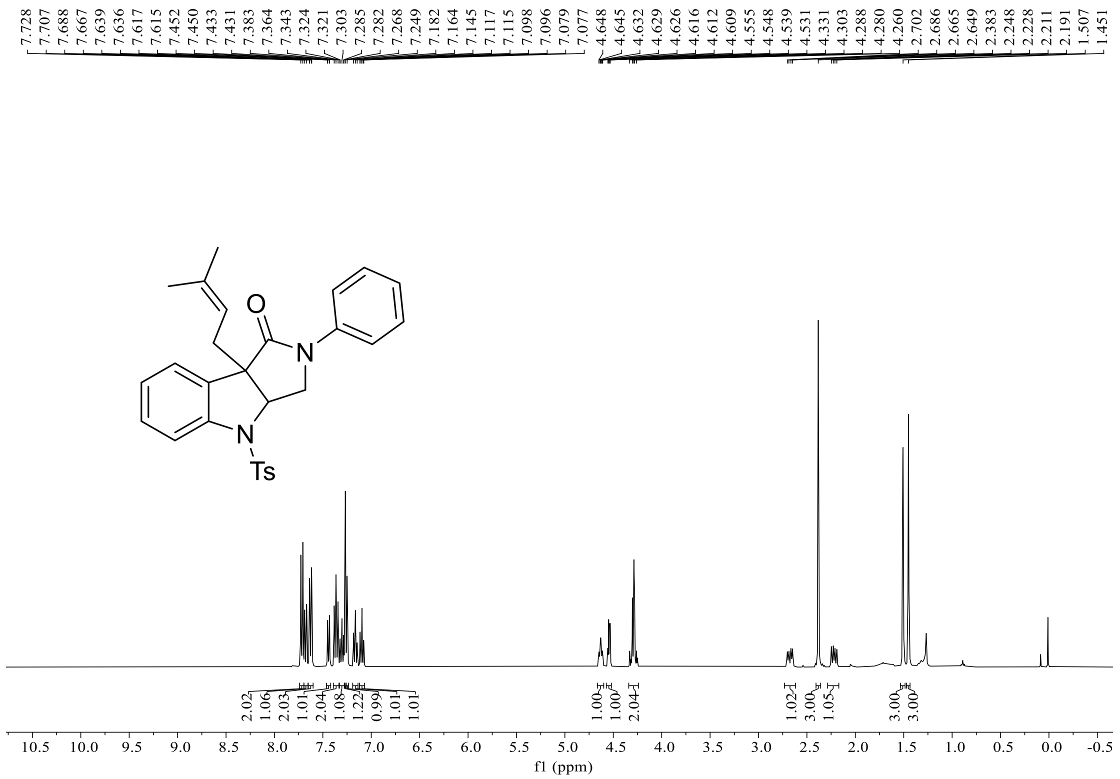

Supplementary Fig. 181. ^{1}H NMR of compound **7i** (400 MHZ, $CDCl_3$)

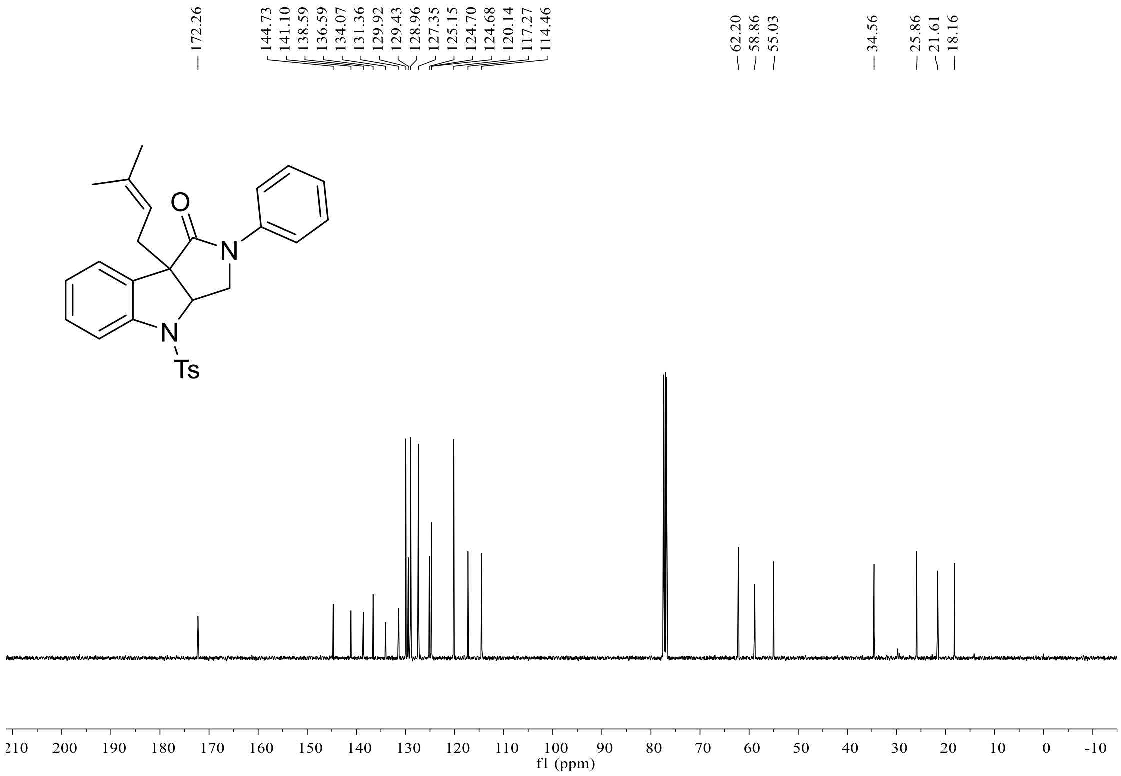

Supplementary Fig. 182. ^{13}C NMR of compound **7i** (100 MHZ, $CDCl_3$)

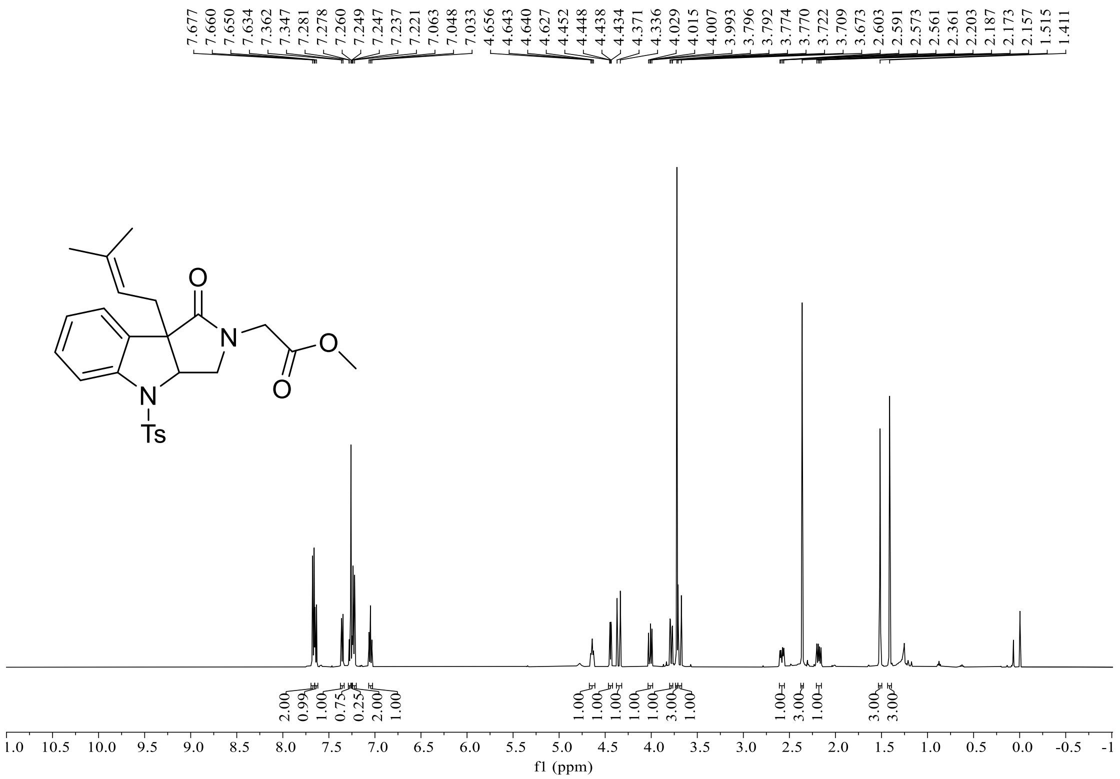

Supplementary Fig. 183 ^{1}H NMR of compound **7j** (500 MHZ, $CDCl_3$)

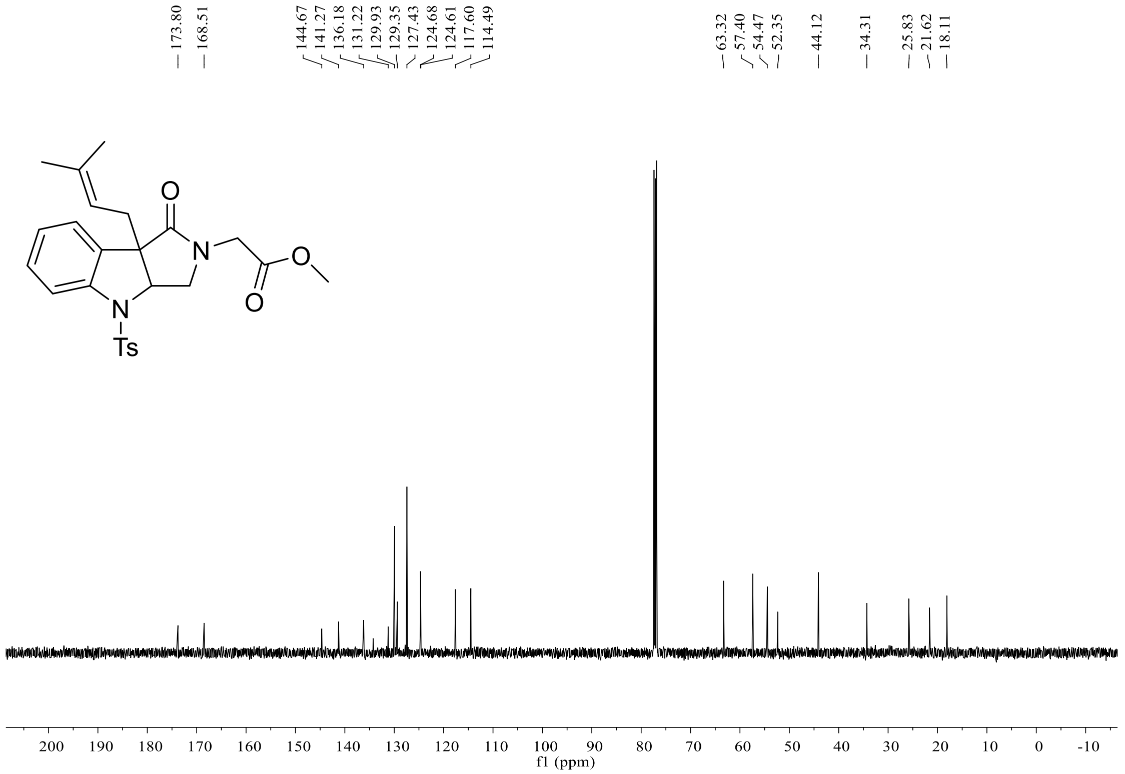

Supplementary Fig. 184. ^{13}C NMR of compound **7j** (125 MHZ, $CDCl_3$)

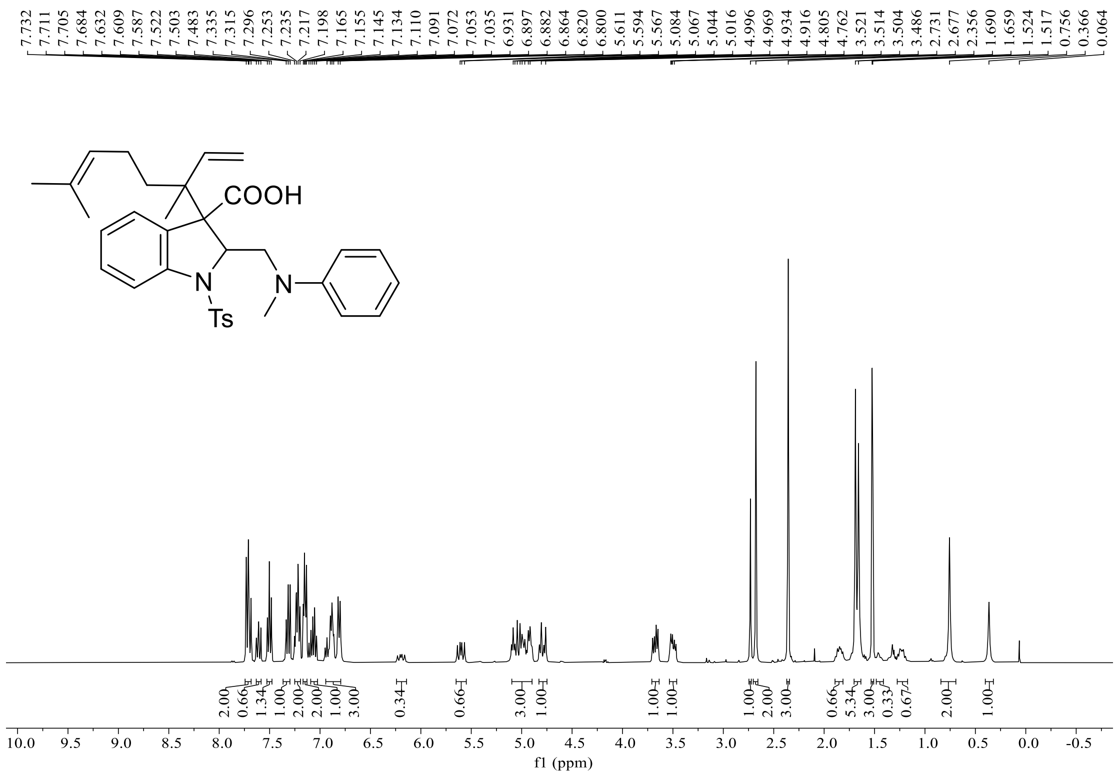

Supplementary Fig. 185. ^{1}H NMR of compound **9a** (400 MHZ, $CDCl_3$)

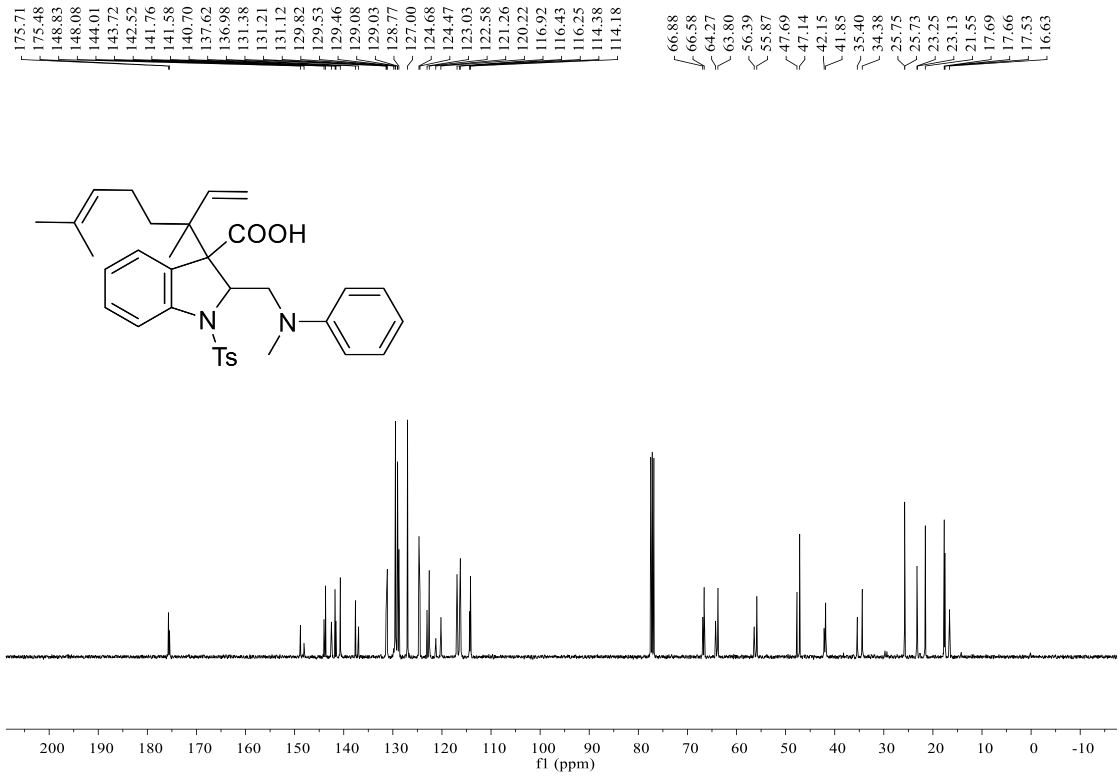

Supplementary Fig. 186. ^{13}C NMR of compound **9a** (100 MHZ, $CDCl_3$)

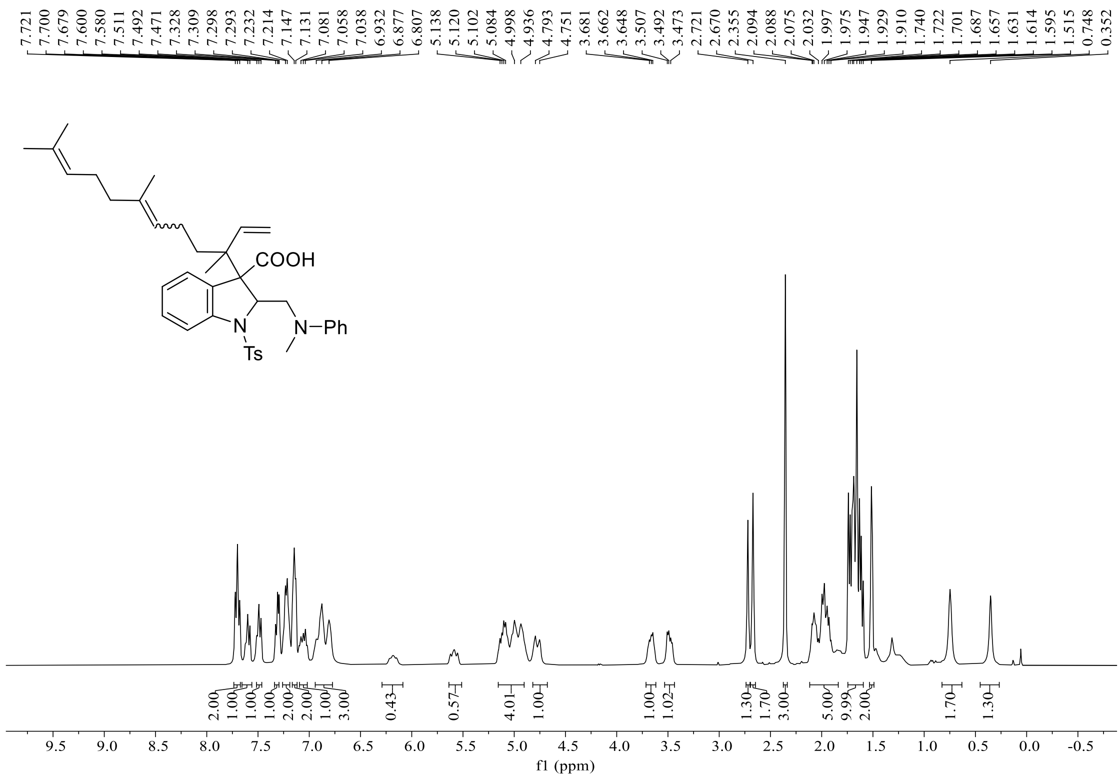

Supplementary Fig. 187. ^{1}H NMR of compound **9b** (400 MHZ, $CDCl_3$)

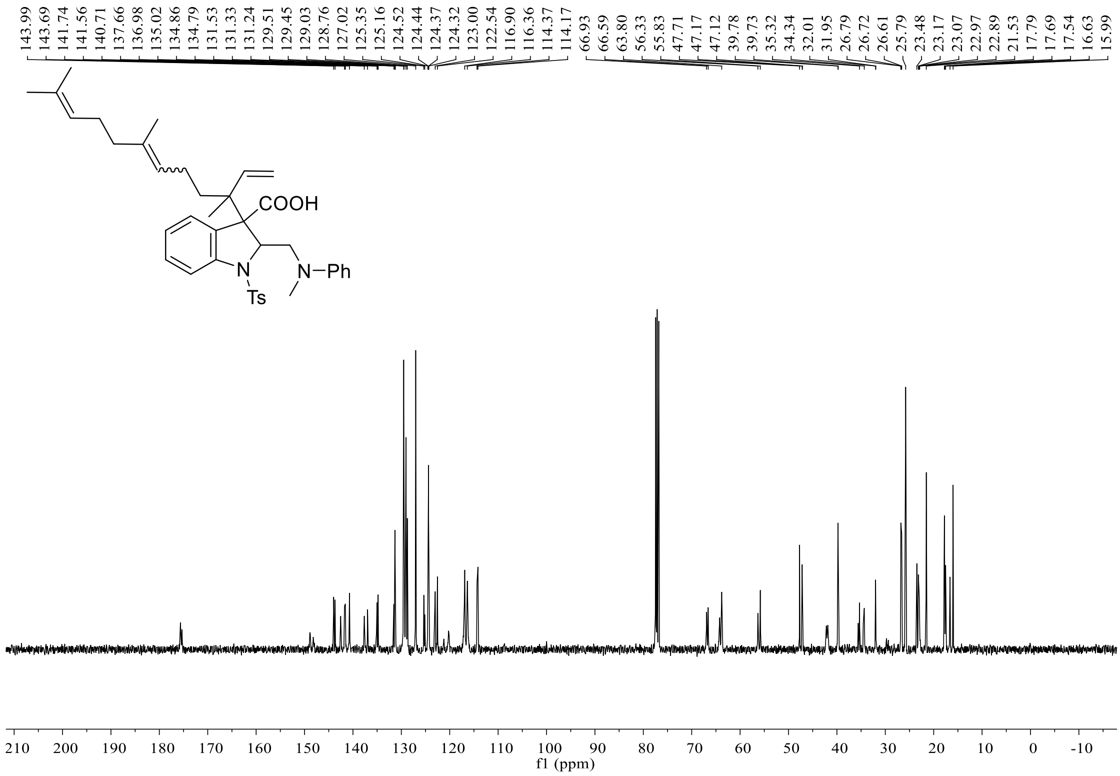

Supplementary Fig. 188. ^{13}C NMR of compound **9b** (100 MHZ, $CDCl_3$)

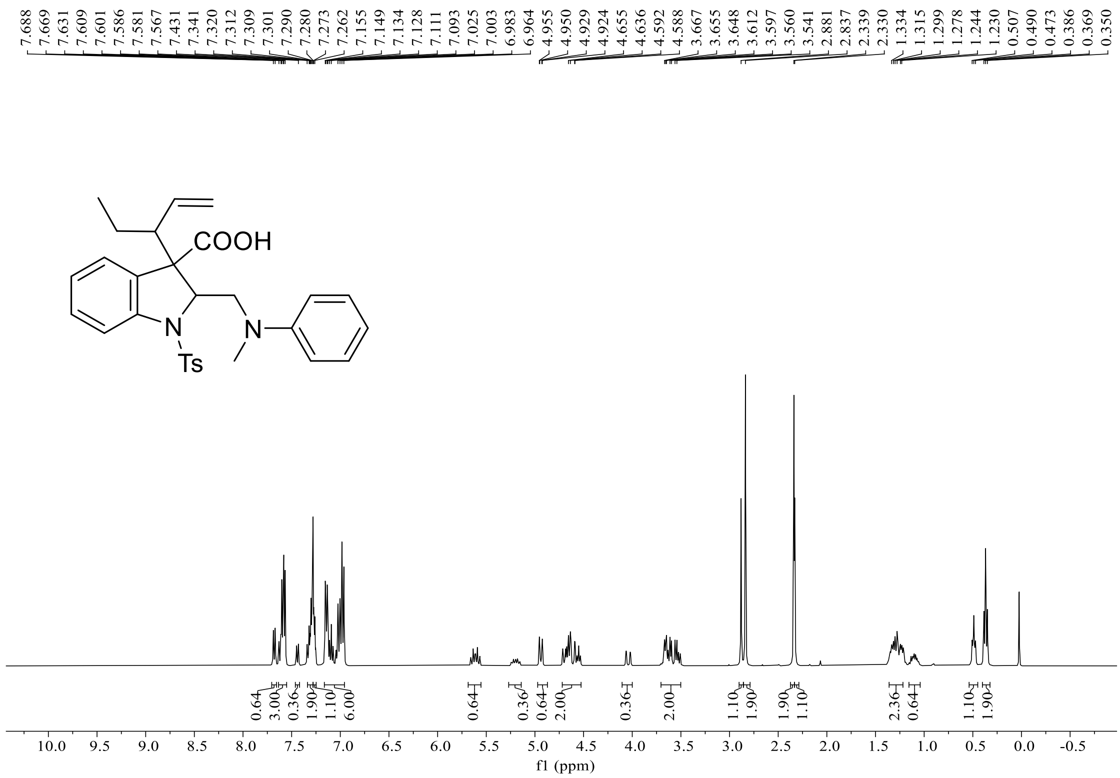

Supplementary Fig. 189. ^{1}H NMR of compound **9c** (400 MHZ, $CDCl_3$)

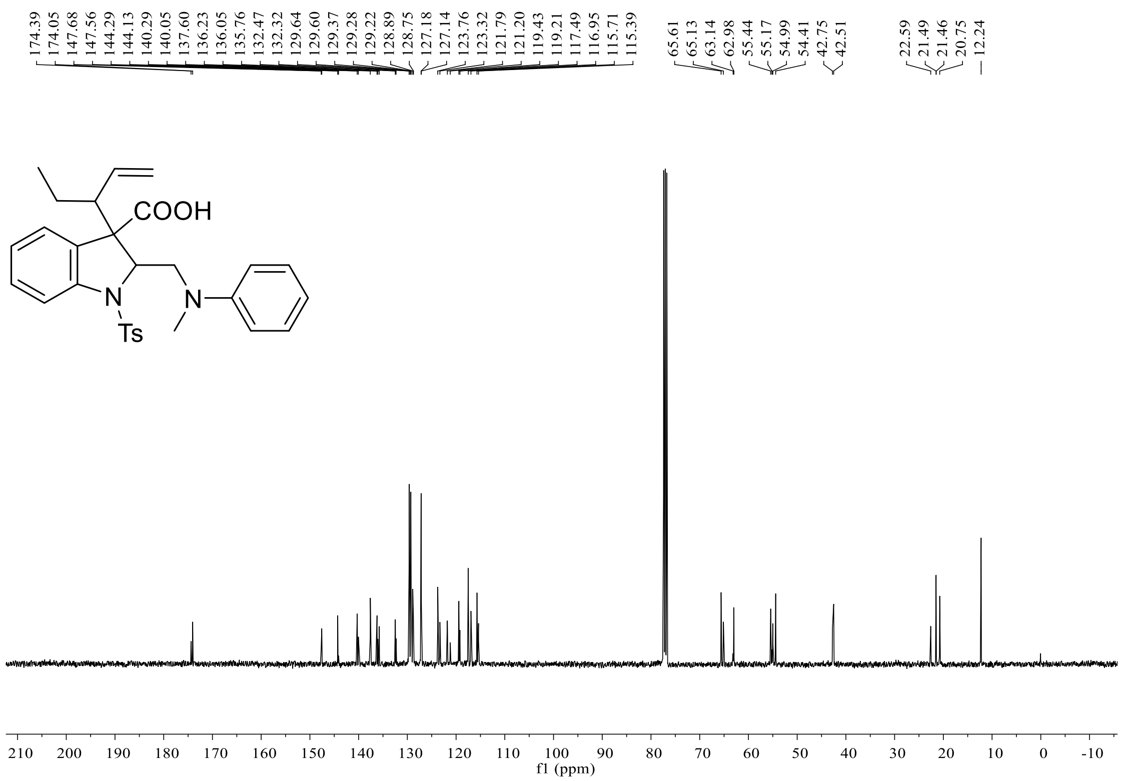

Supplementary Fig. 190. ^{13}C NMR of compound **9c** (100 MHZ, $CDCl_3$)

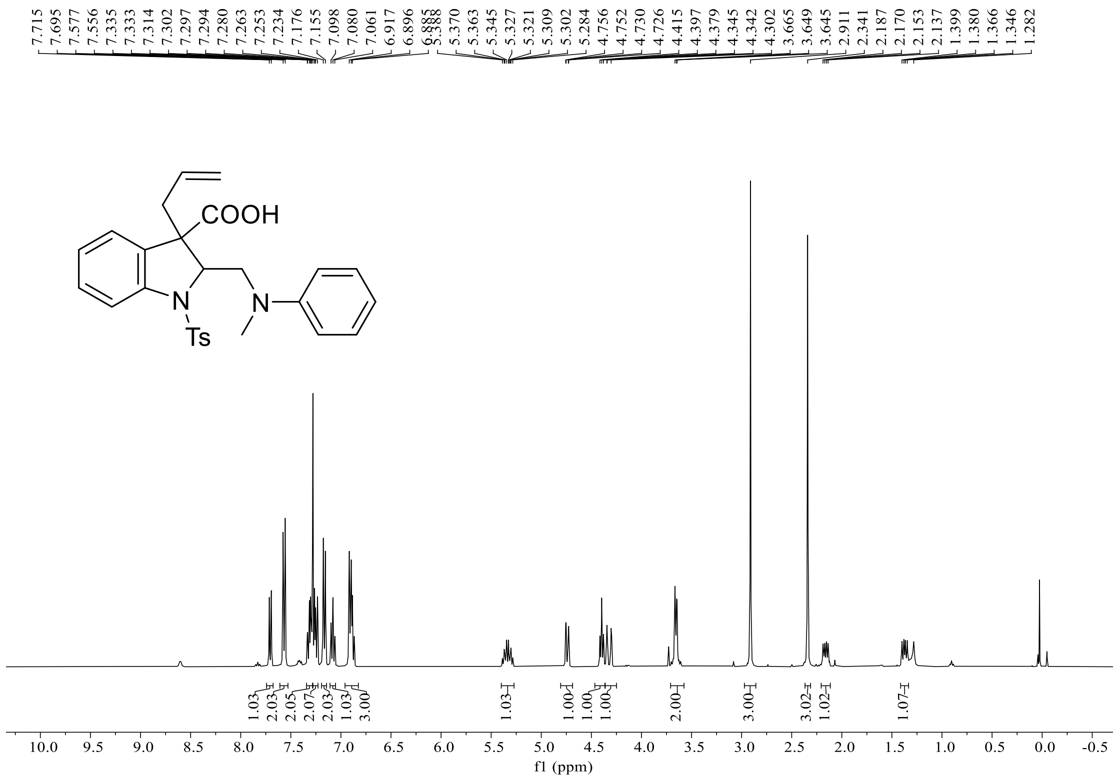

Supplementary Fig. 191. ^{1}H NMR of compound **9d** (400 MHZ, $CDCl_3$)

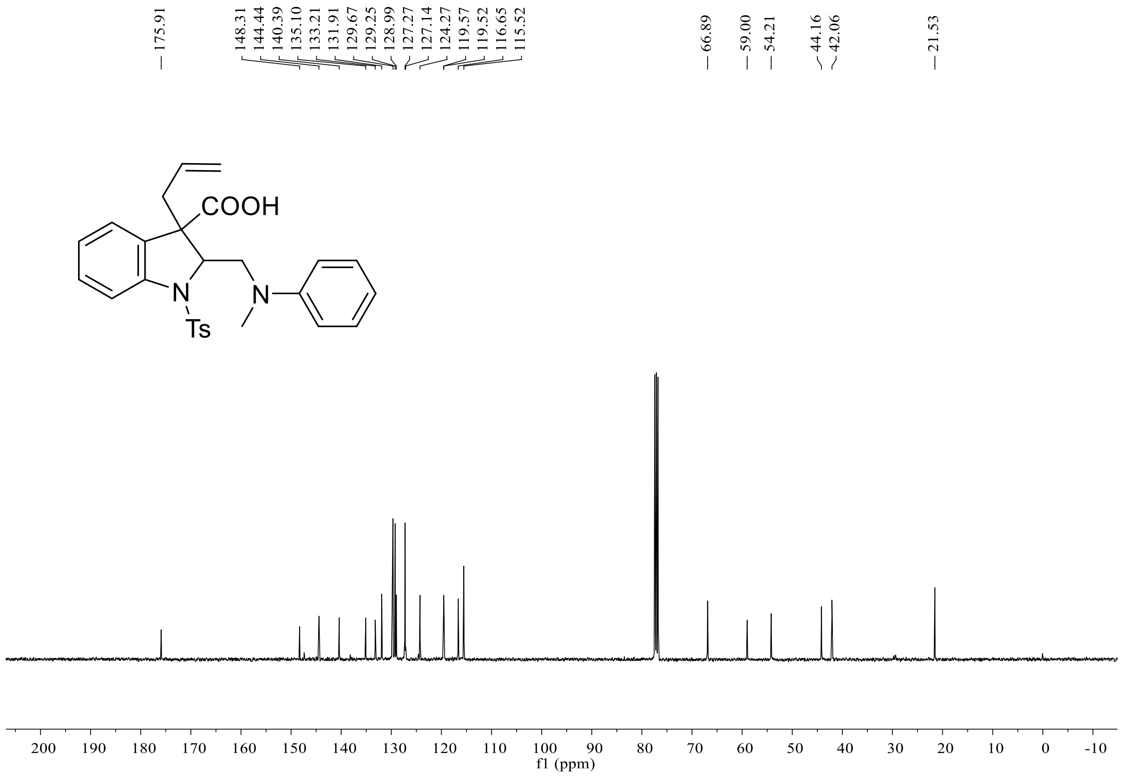

Supplementary Fig. 192. ^{13}C NMR of compound **9d** (100 MHZ, $CDCl_3$)

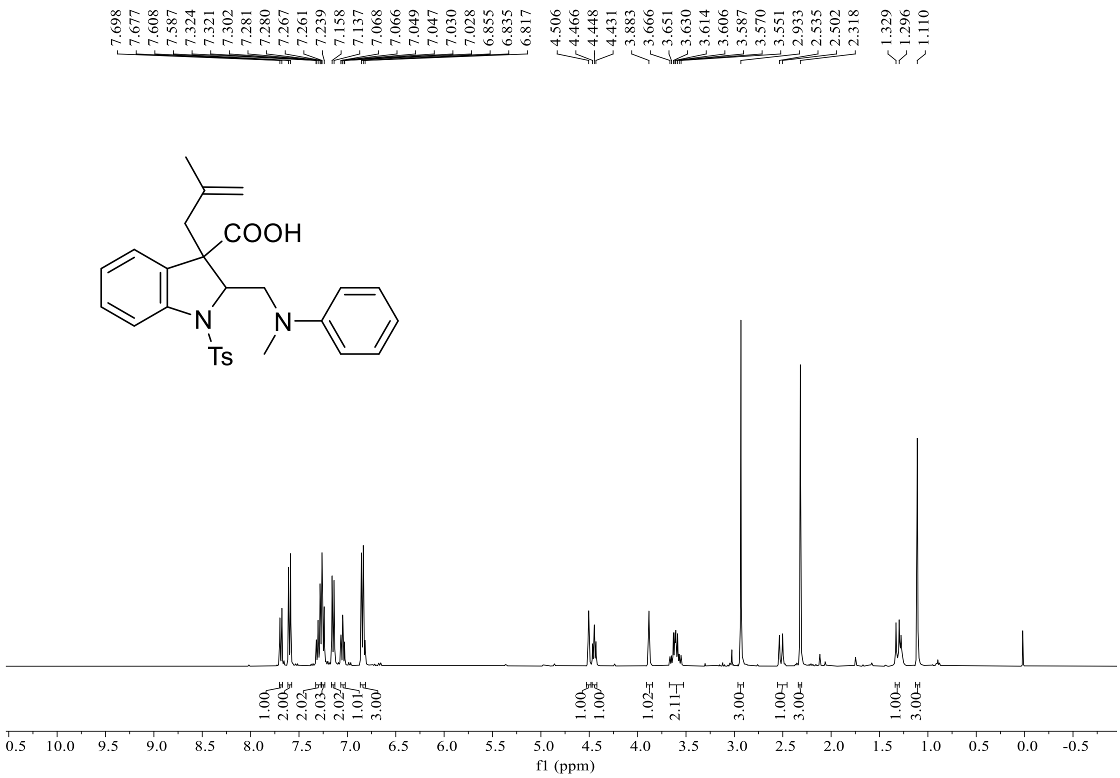

Supplementary Fig. 193. ^{1}H NMR of compound **9e** (400 MHZ, $CDCl_3$)

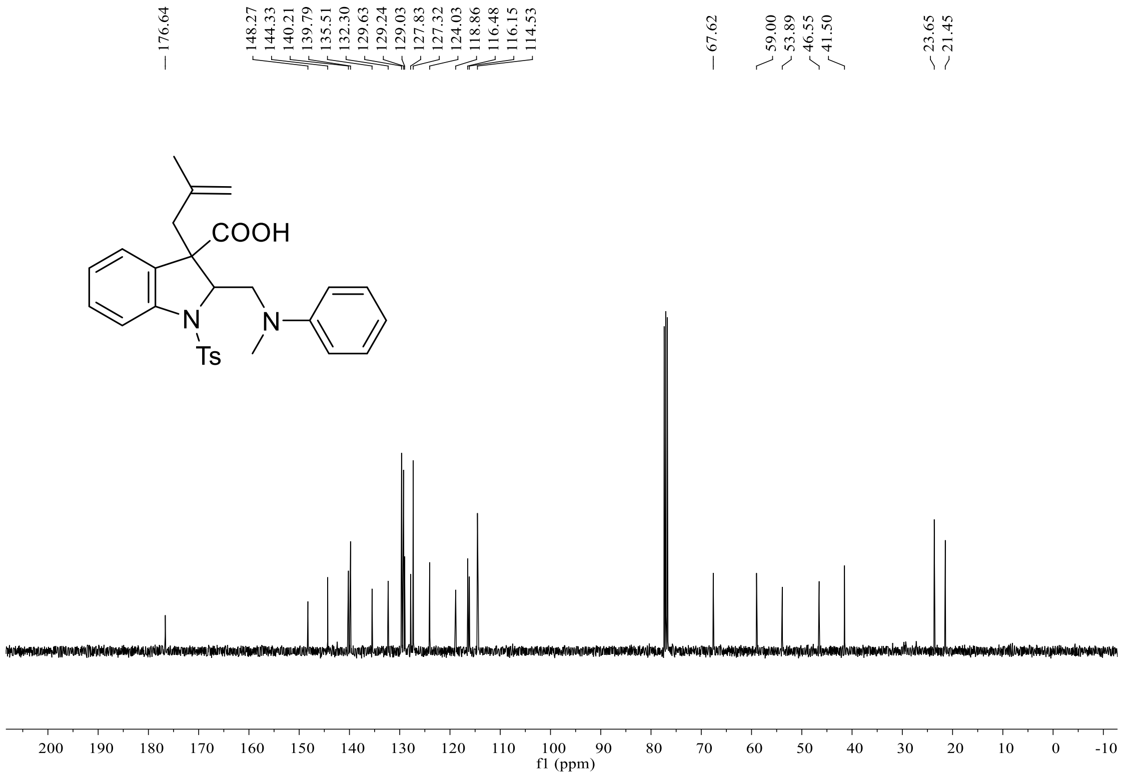

Supplementary Fig. 194. ^{13}C NMR of compound **9e** (100 MHZ, $CDCl_3$)

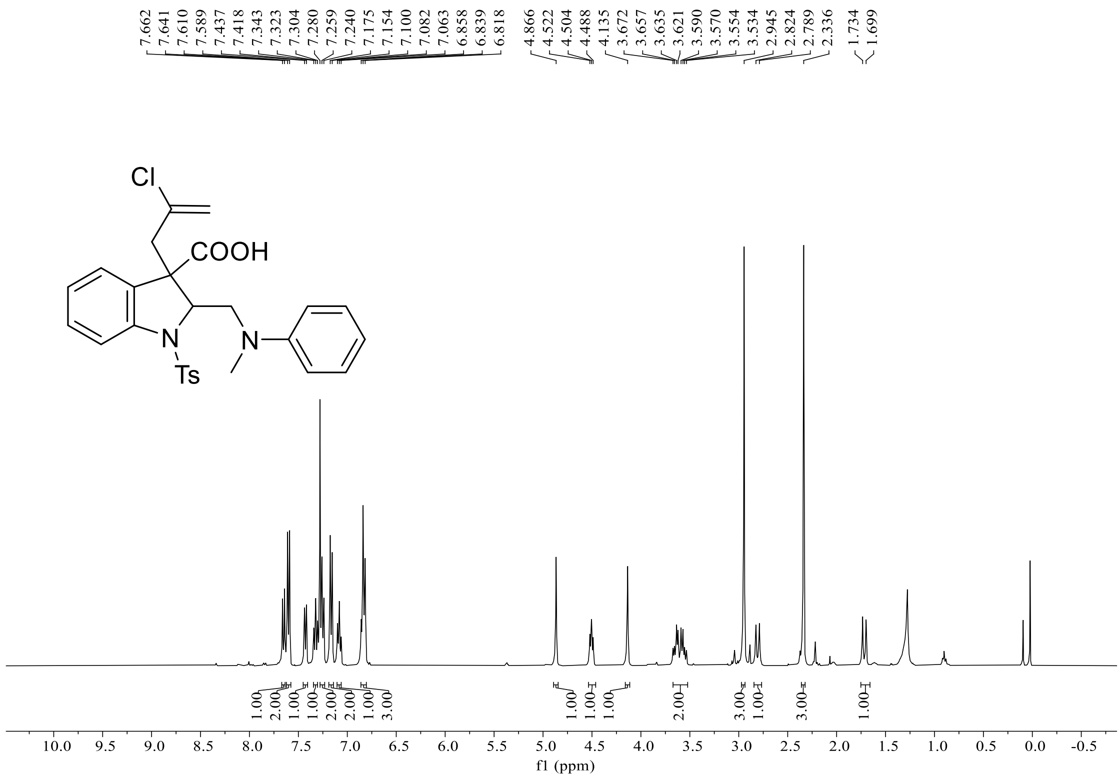

Supplementary Fig. 195. ^{1}H NMR of compound **9f** (400 MHZ, $CDCl_3$)

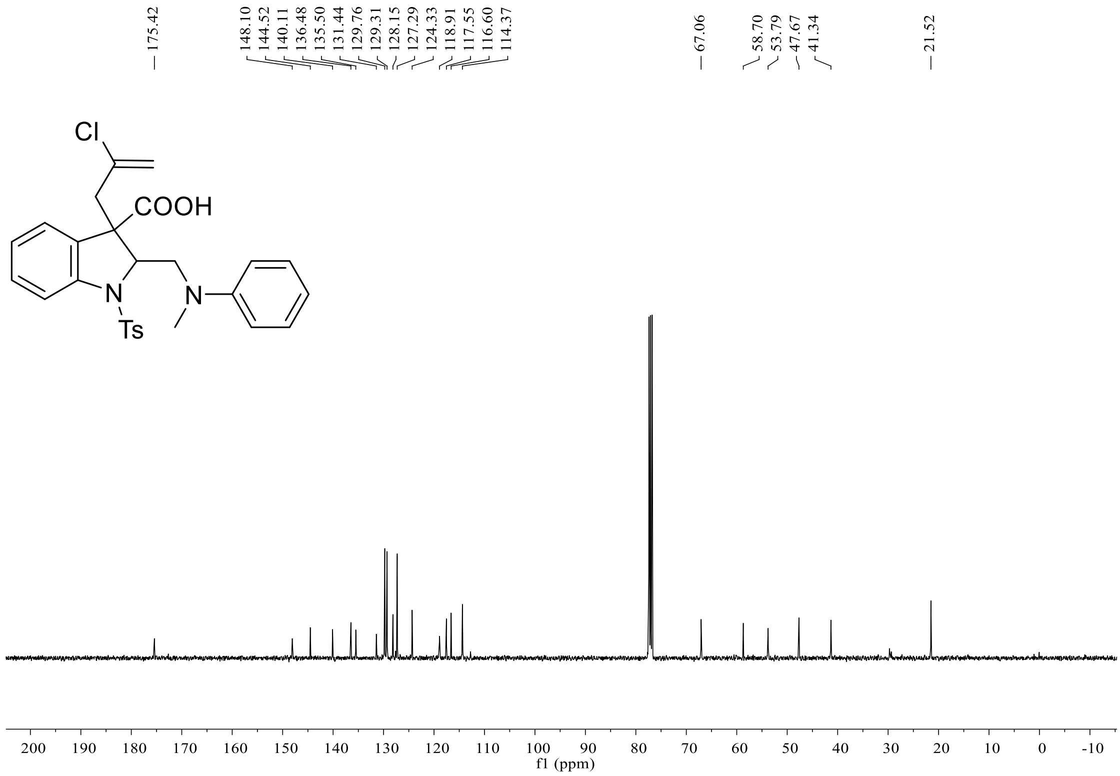

Supplementary Fig. 196. ^{13}C NMR of compound **9f** (100 MHZ, $CDCl_3$)

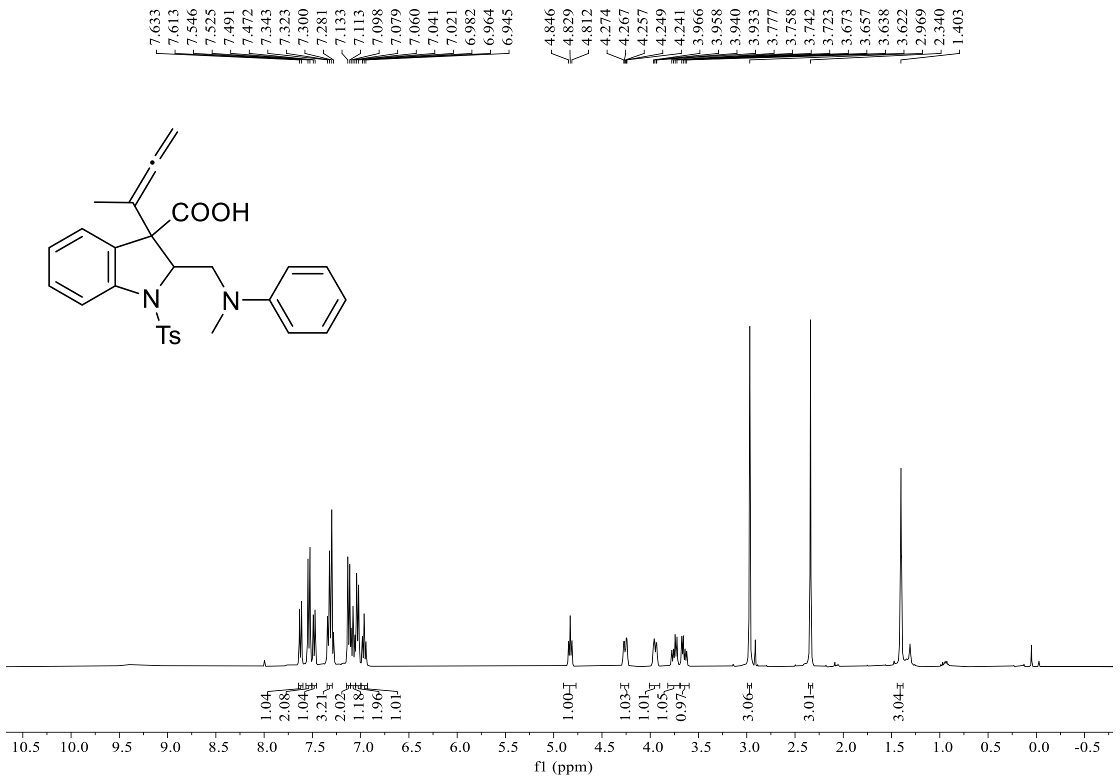

Supplementary Fig. 197. ^{1}H NMR of compound **9g** (400 MHZ, $CDCl_3$)

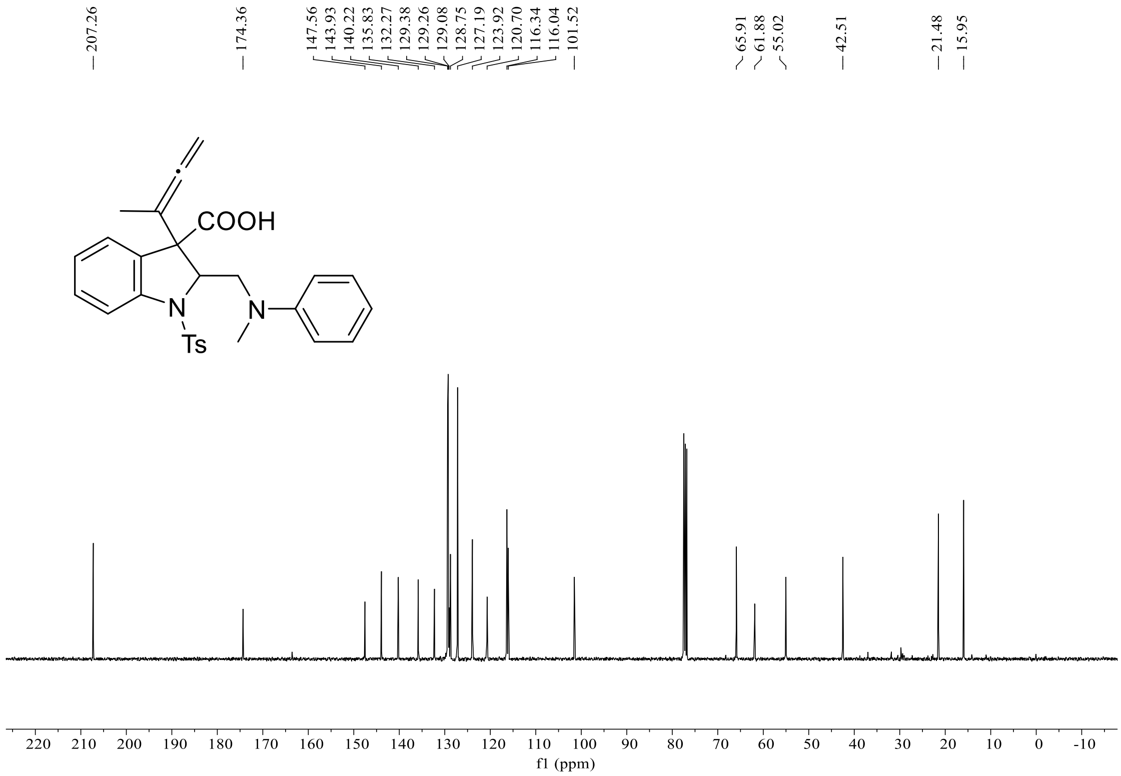

Supplementary Fig. 198. ^{13}C NMR of compound **9g** (100 MHZ, $CDCl_3$)

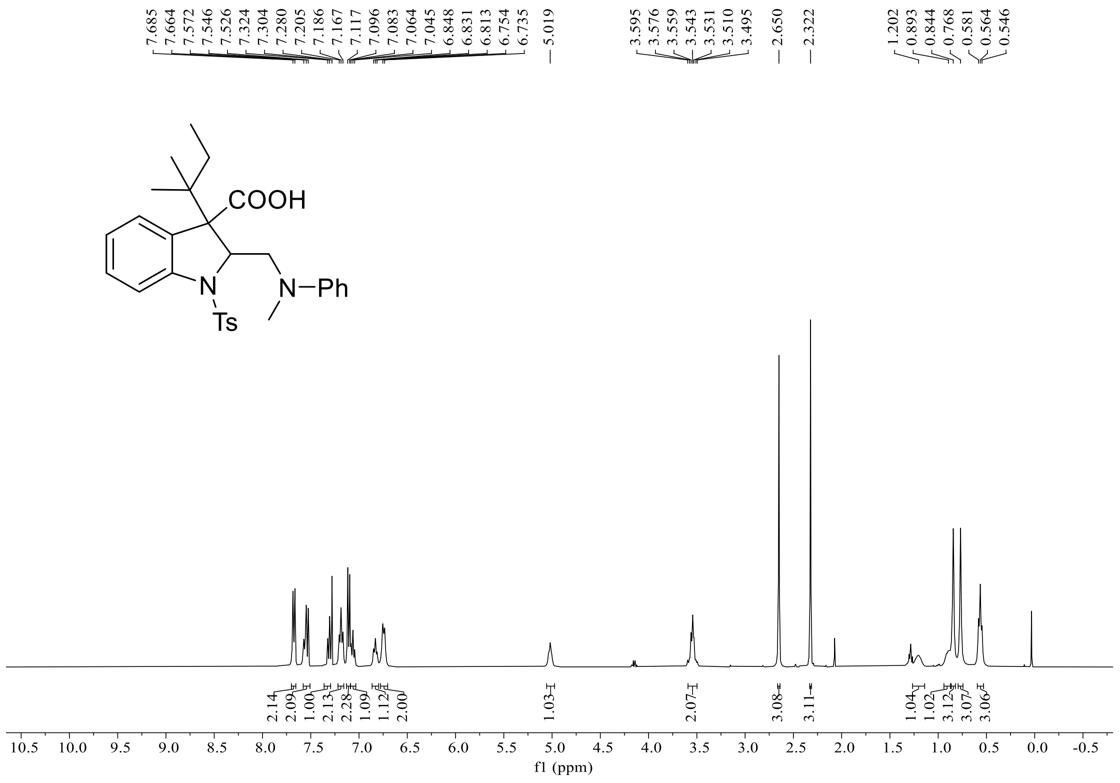

Supplementary Fig. 199. ^{1}H NMR of compound **10** (400 MHZ, $CDCl_3$)

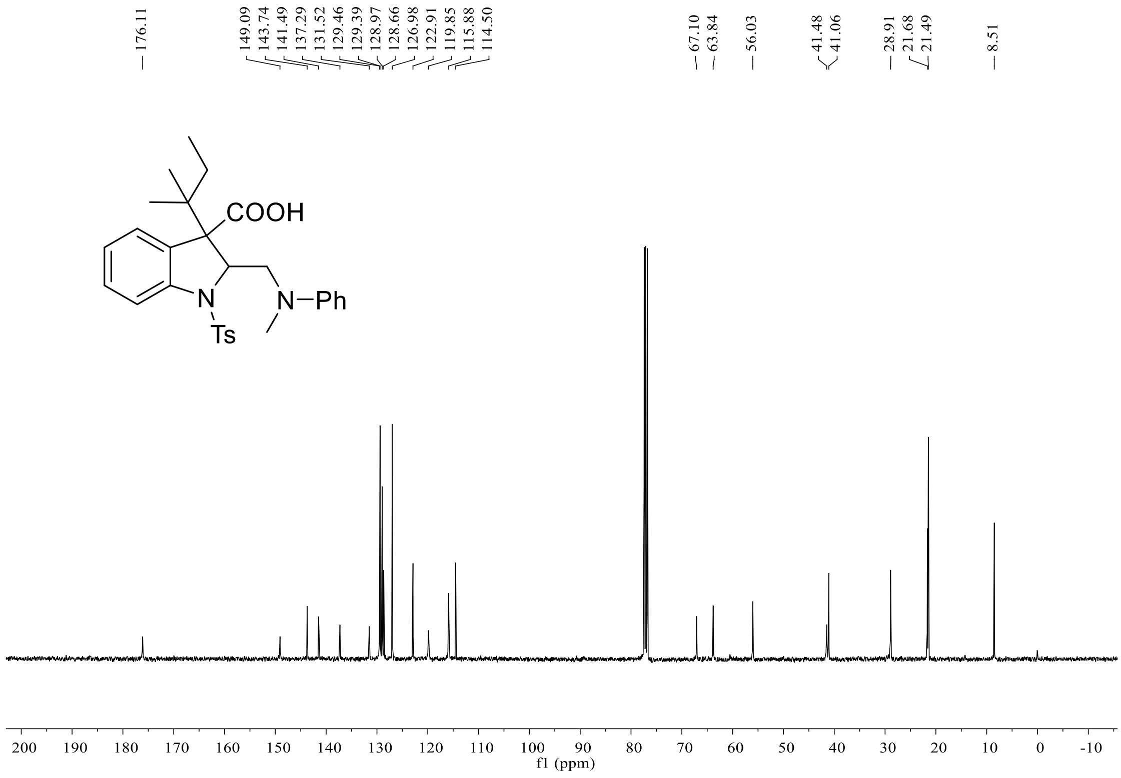

Supplementary Fig. 200. ^{13}C NMR of compound **10** (100 MHZ, $CDCl_3$)

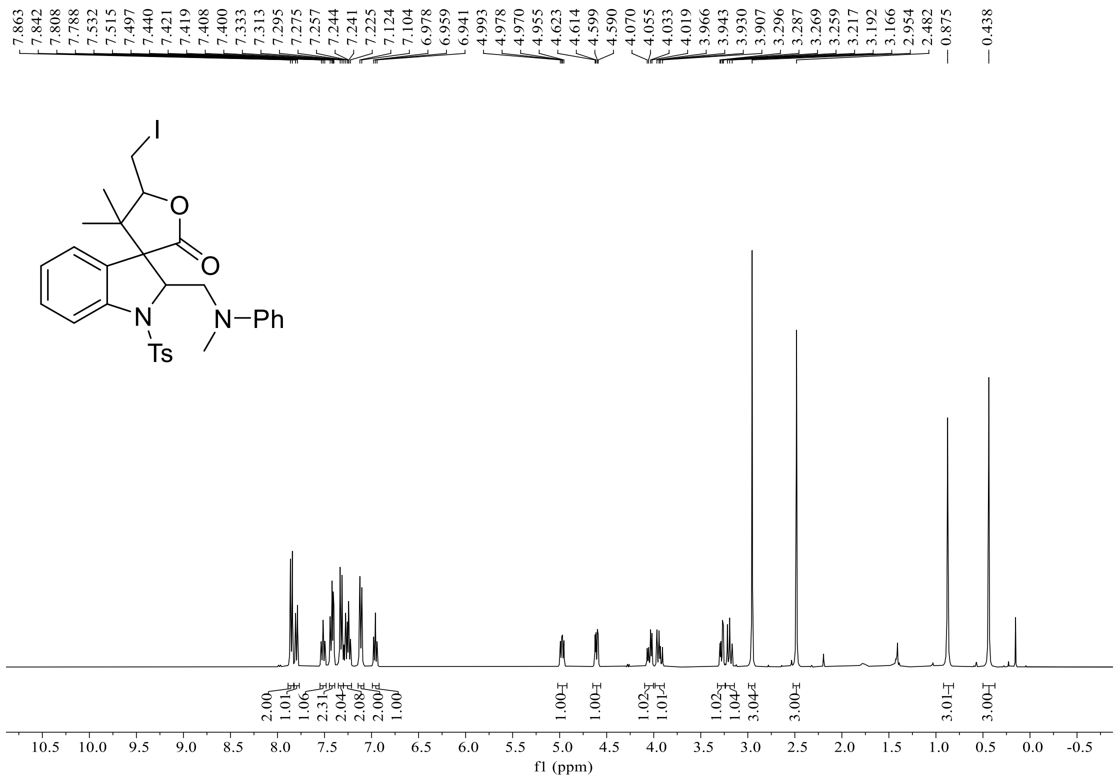

Supplementary Fig. 201. ^{1}H NMR of compound **11** (500 MHZ, $CDCl_3$)

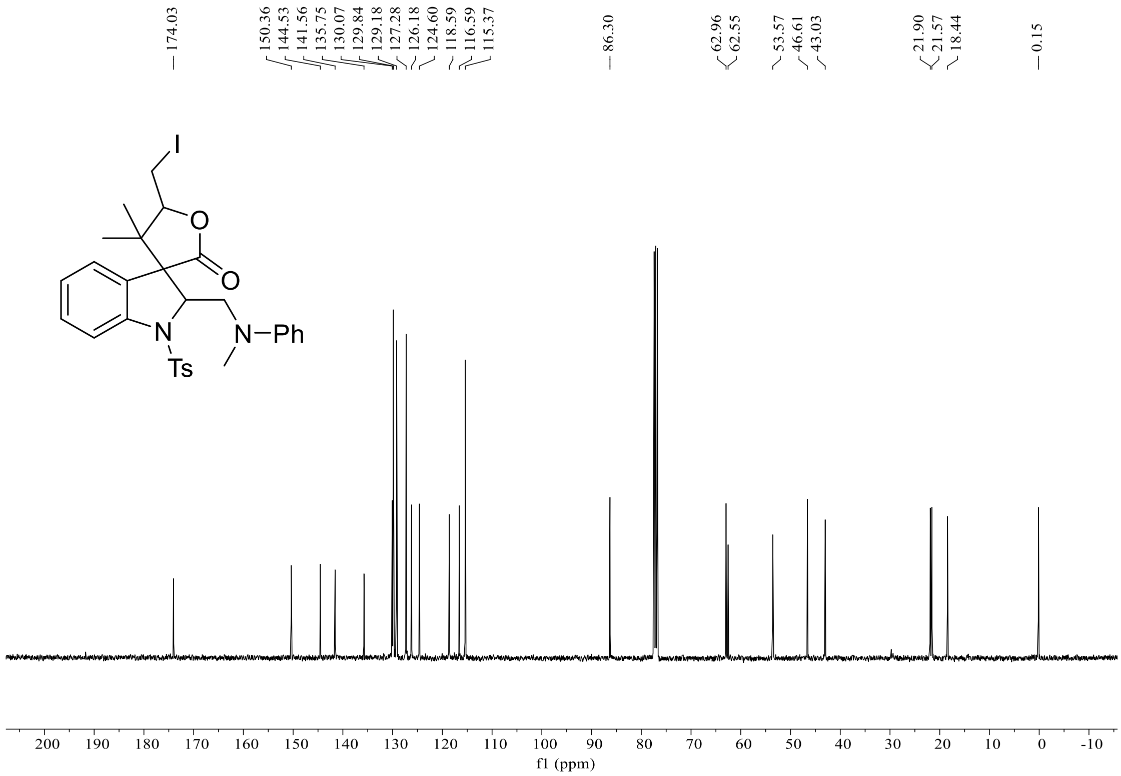

Supplementary Fig. 202. ^{13}C NMR of compound **11** (125 MHZ, $CDCl_3$)

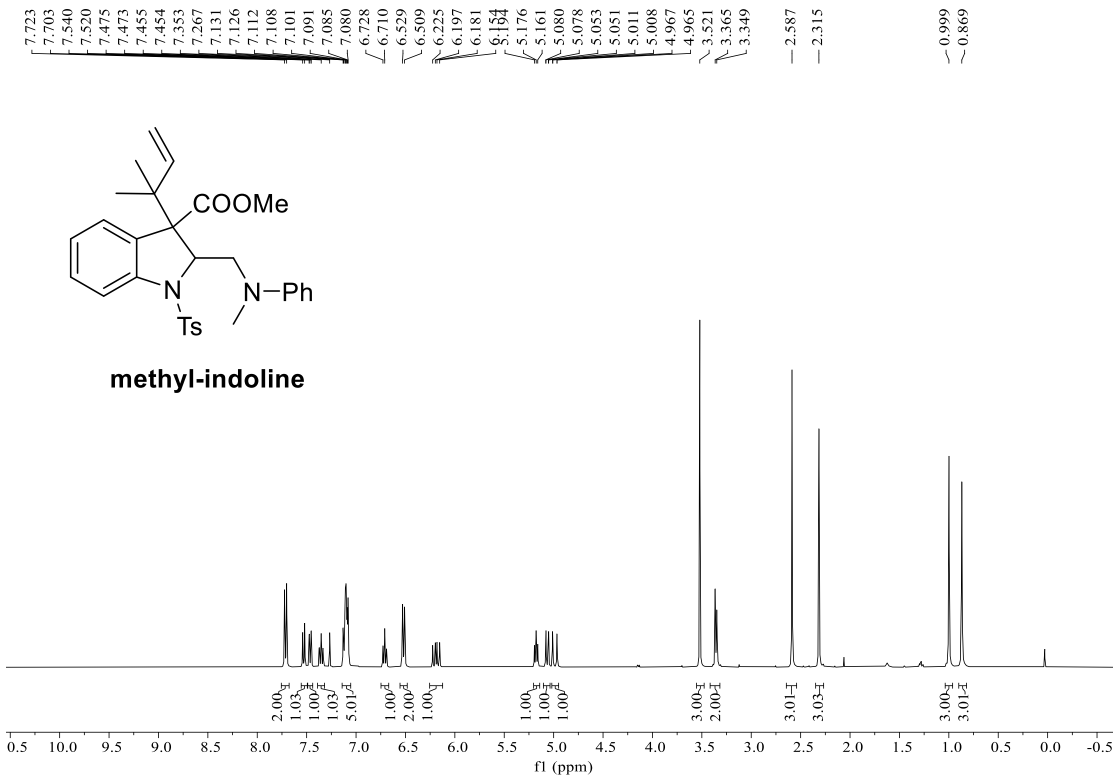

Supplementary Fig. 203. ^{1}H NMR of compound **methyl-indoline** (400 MHZ, $CDCl_3$)

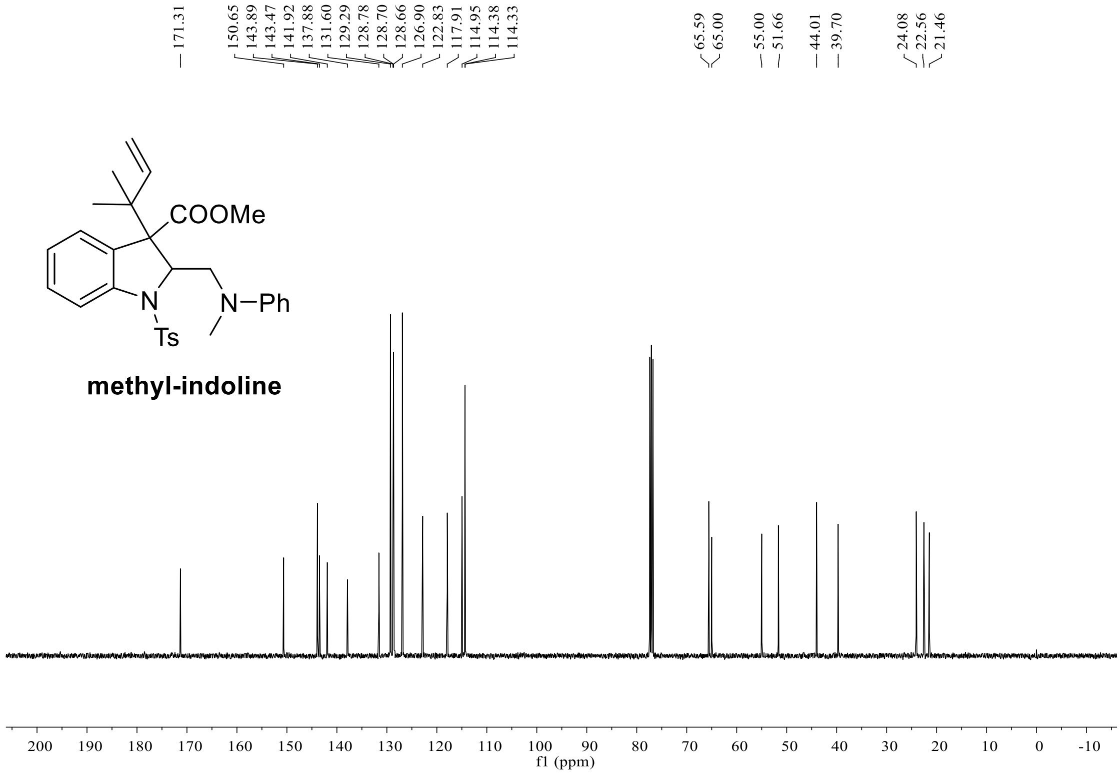

Supplementary Fig. 204. ^{13}C NMR of compound **methyl-indoline** (100 MHZ, $CDCl_3$)

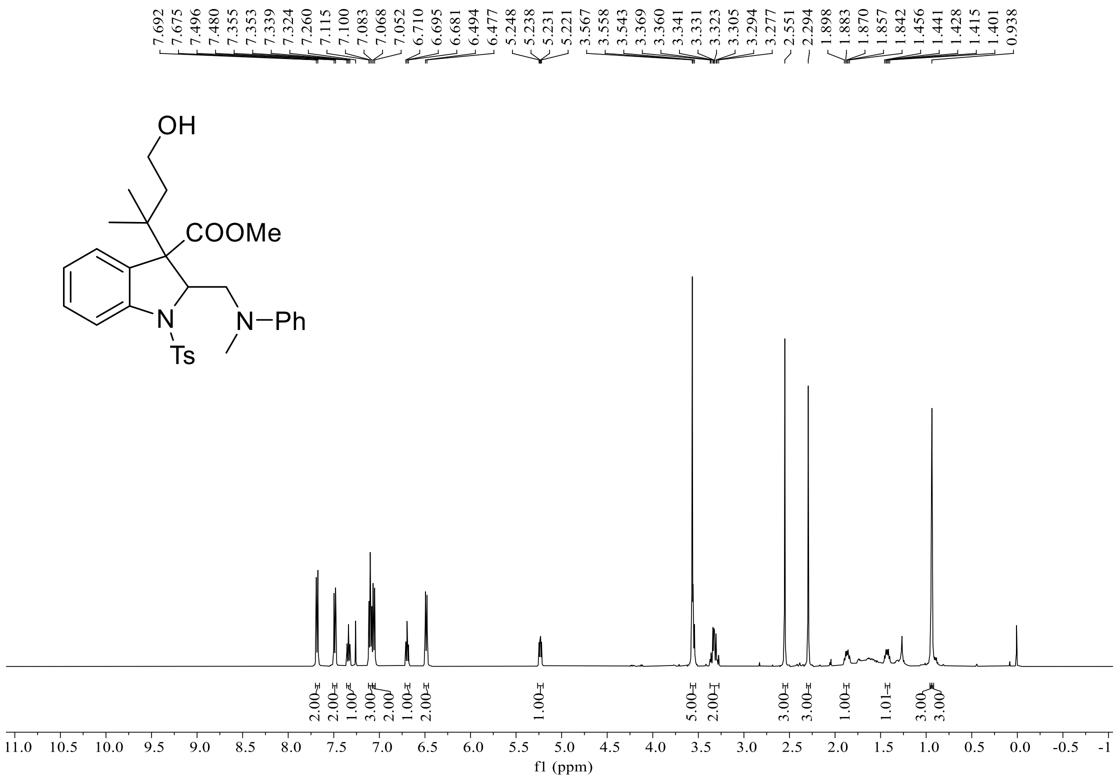

Supplementary Fig. 205. ^{1}H NMR of compound **12** (500 MHZ, $CDCl_3$)

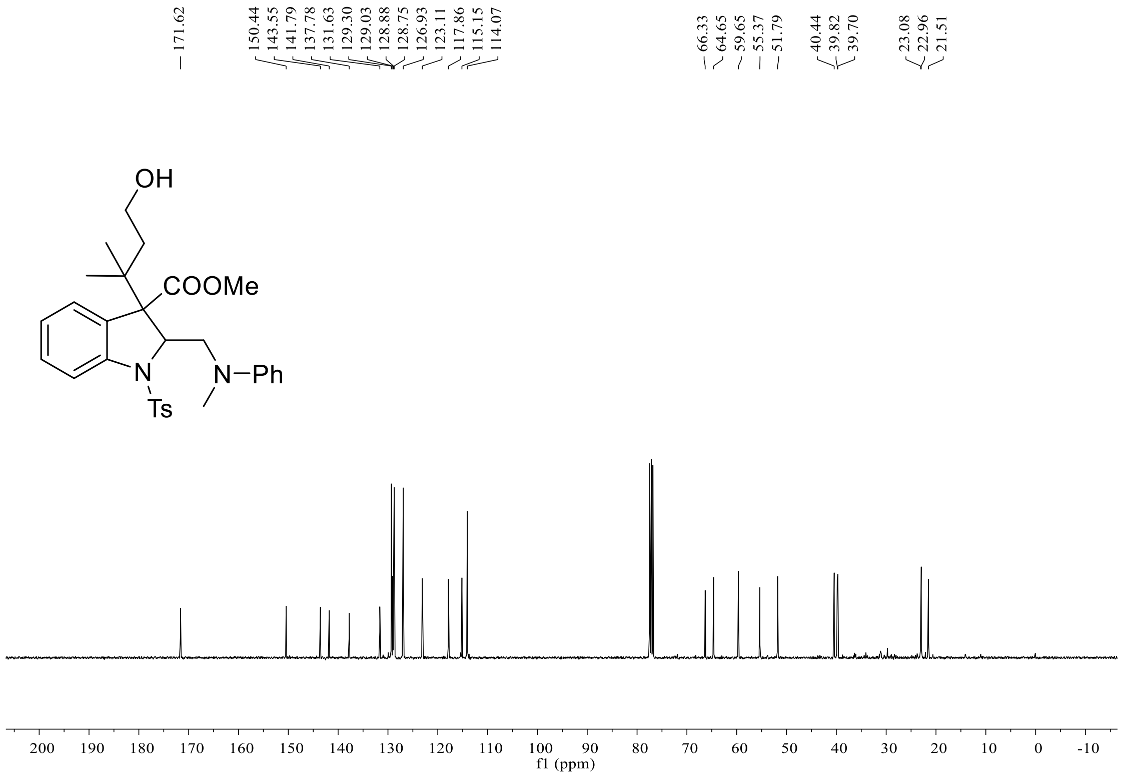

Supplementary Fig. 206. ^{13}C NMR of compound **12** (125 MHZ, $CDCl_3$)

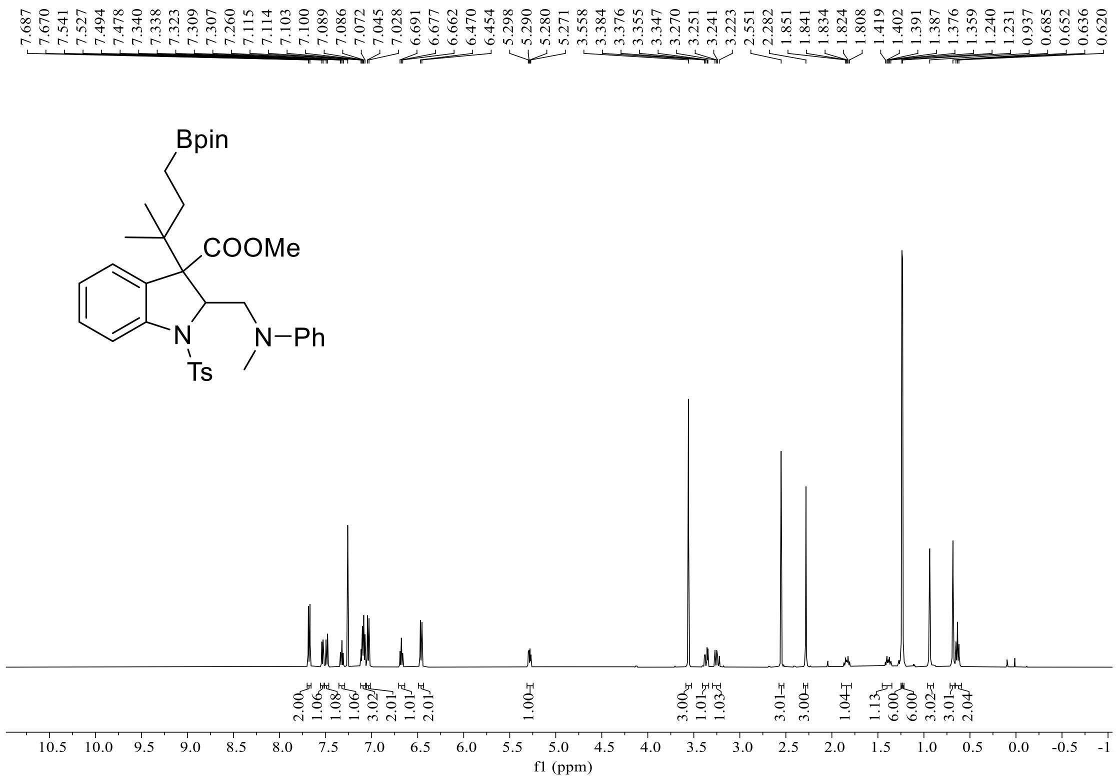

Supplementary Fig. 207. ^{1}H NMR of compound **13** (500 MHZ, $CDCl_3$)

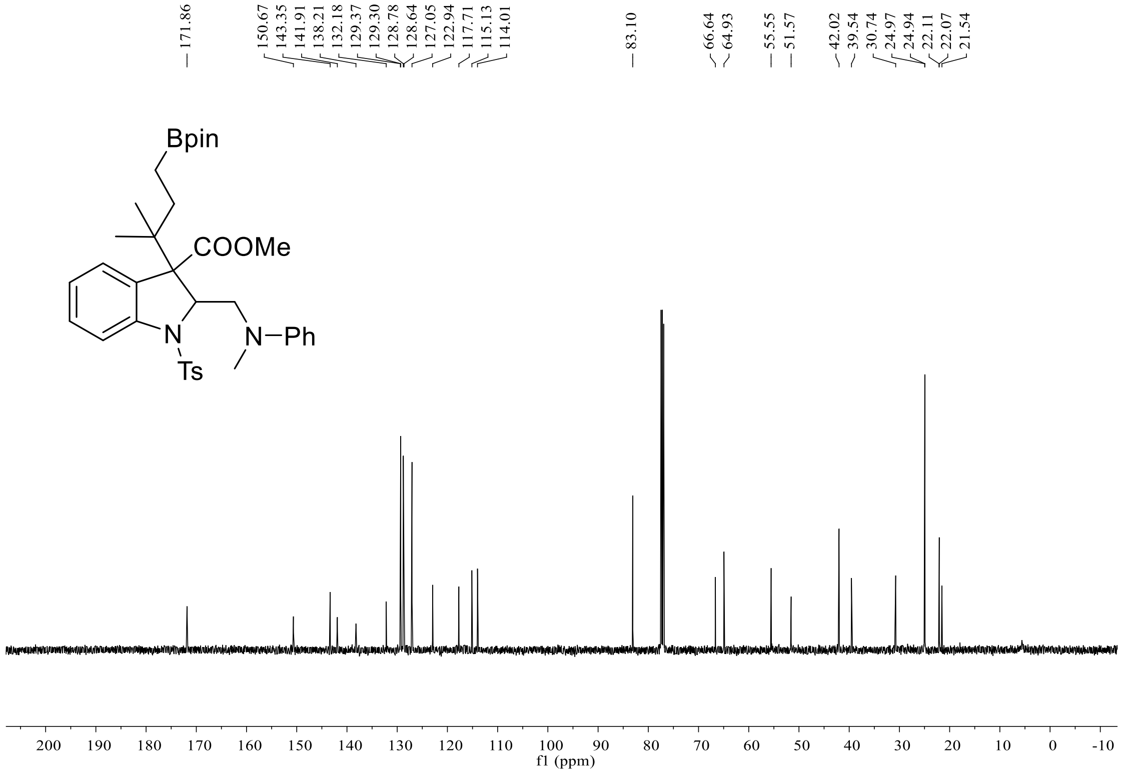

Supplementary Fig. 208. ^{13}C NMR of compound **13** (125 MHZ, $CDCl_3$)

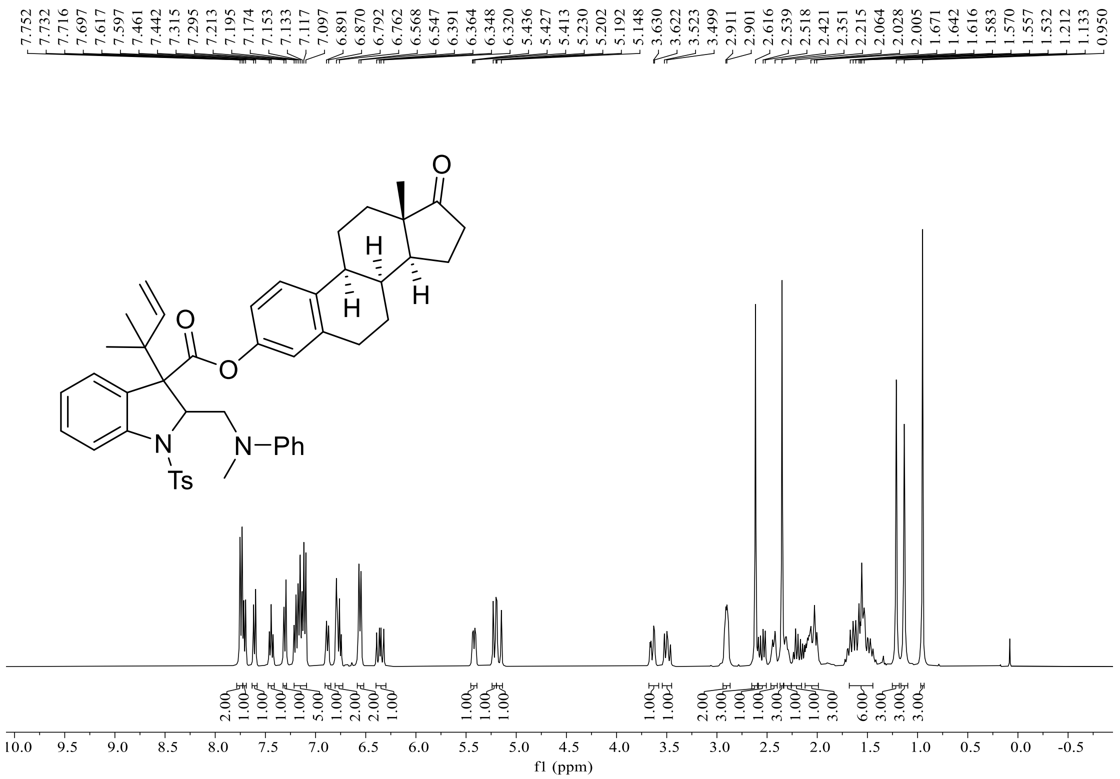

Supplementary Fig. 209. ^{1}H NMR of compound **14** (400 MHZ, $CDCl_3$)

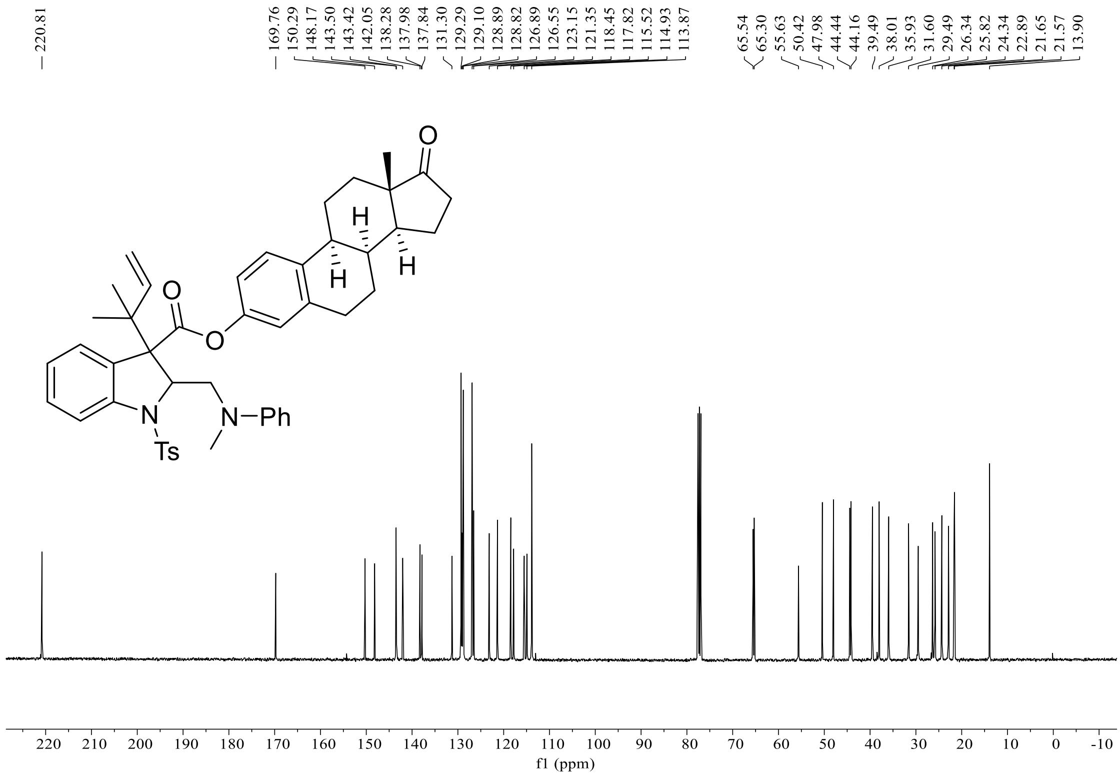

Supplementary Fig. 210. ^{13}C NMR of compound **14** (100 MHZ, $CDCl_3$)

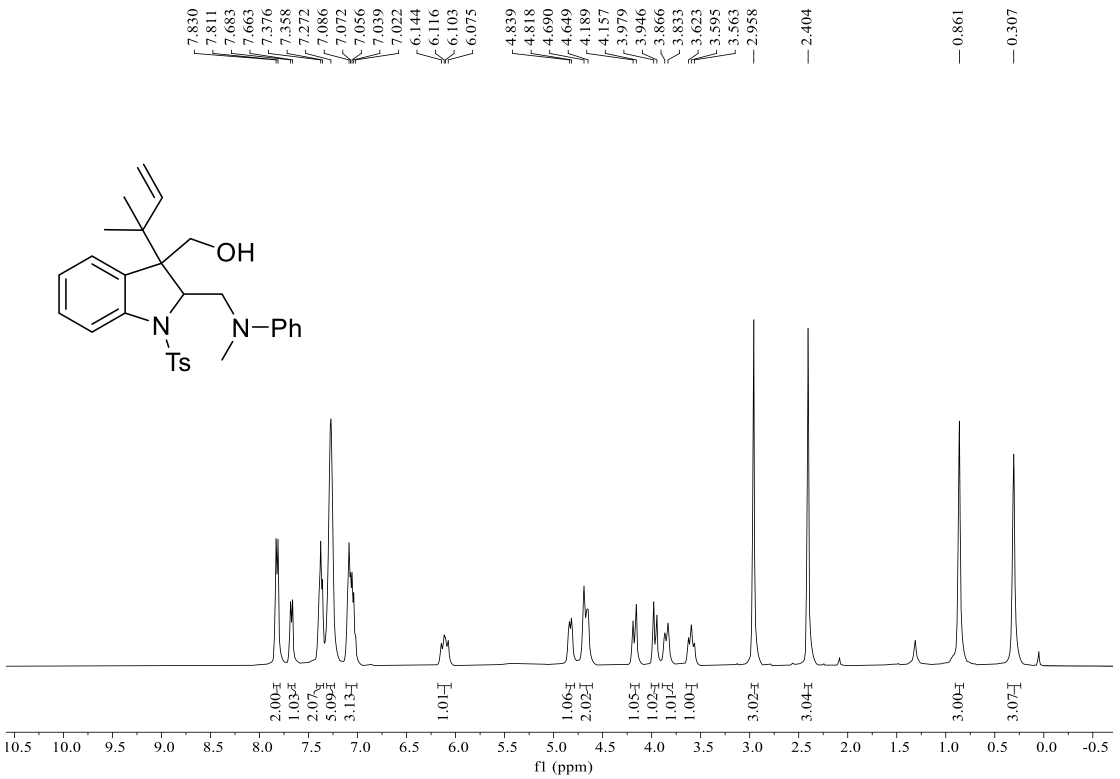

Supplementary Fig. 211. ^{1}H NMR of compound **15** (400 MHZ, $CDCl_3$)

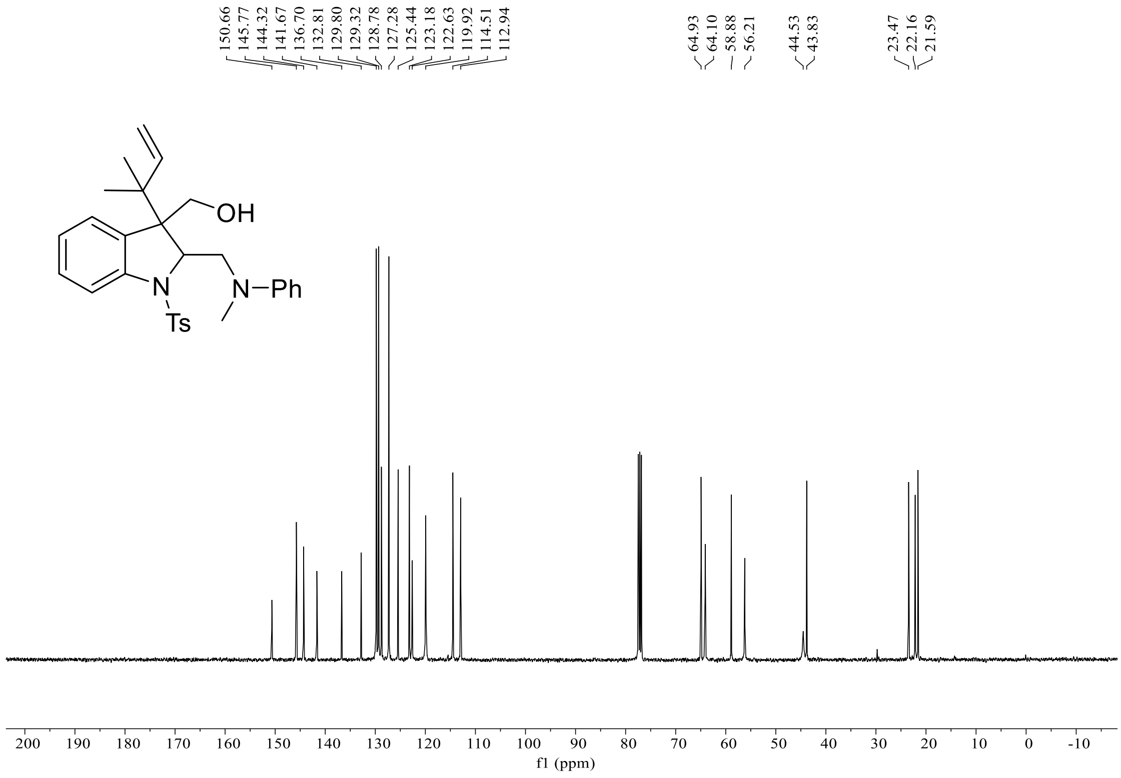

Supplementary Fig. 212. ^{13}C NMR of compound **15** (100 MHZ, $CDCl_3$)

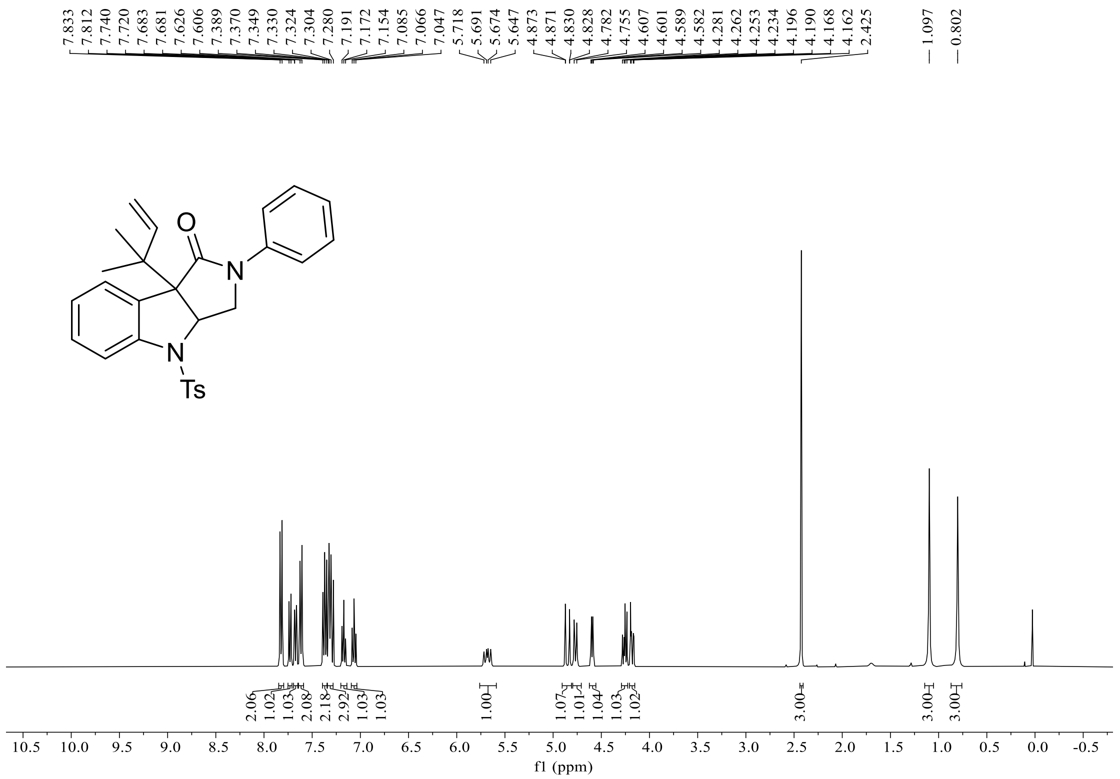

Supplementary Fig. 213. ^{1}H NMR of compound **5a** (400 MHZ, $CDCl_3$)

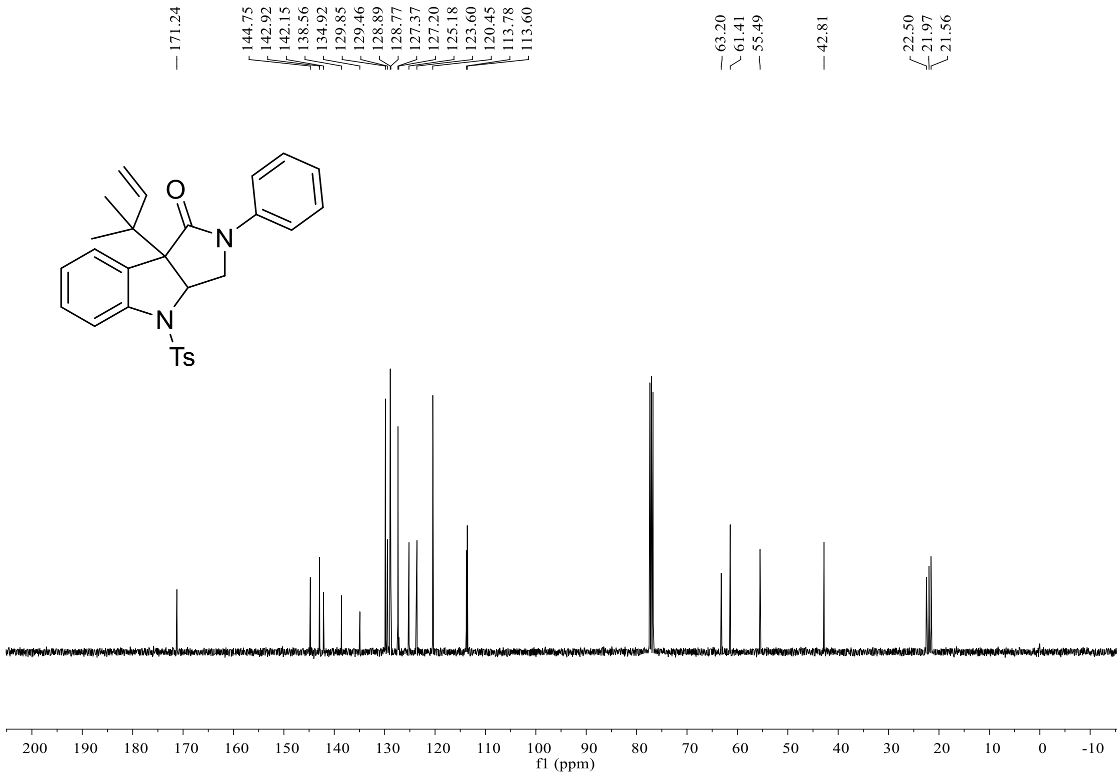

Supplementary Fig. 214. ^{13}C NMR of compound **5a** (100 MHZ, $CDCl_3$)

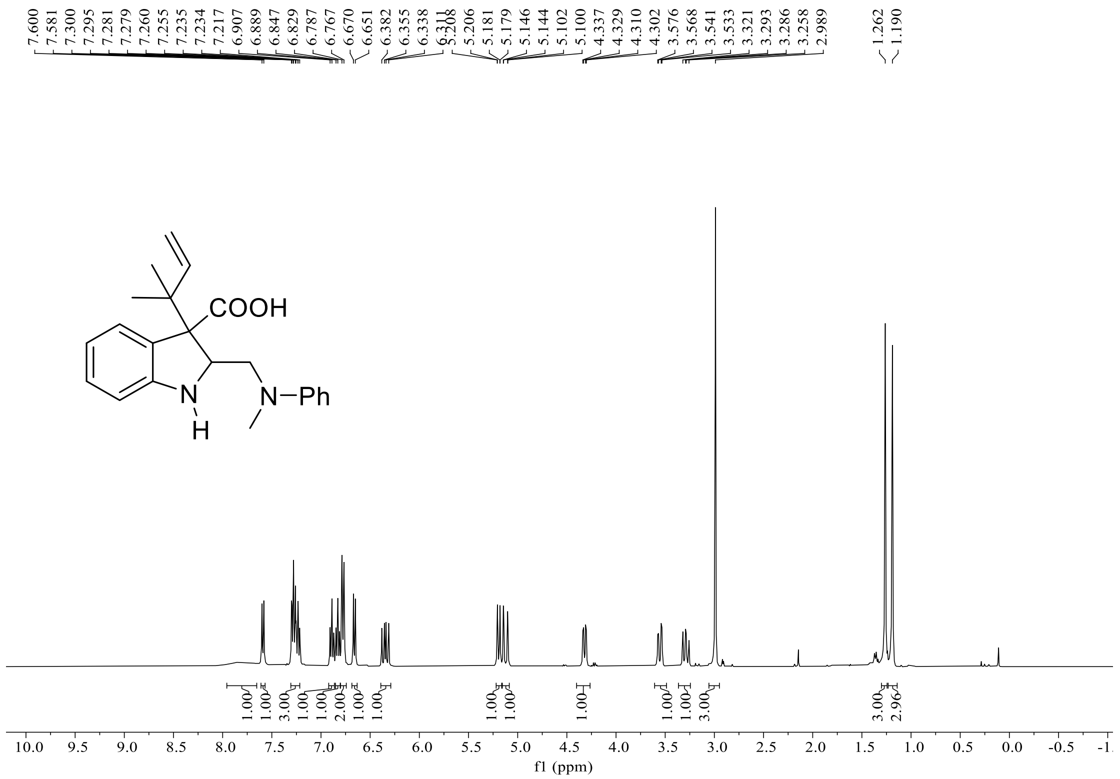

Supplementary Fig. 215. ^{1}H NMR of compound **16** (400 MHZ, $CDCl_3$)

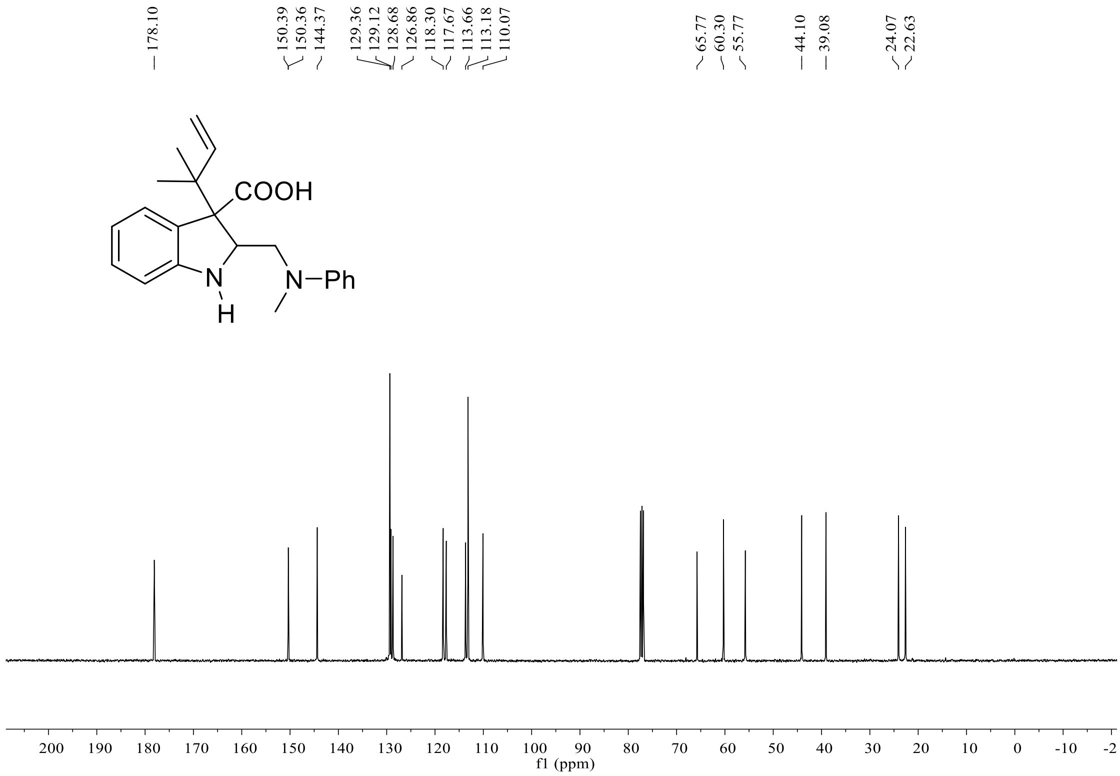

Supplementary Fig. 216. ^{13}C NMR of compound **16** (100 MHZ, $CDCl_3$)

7. Supplementary references

[1]. Kleinmans R., Glorius F., et al. Photoredox-enabled 1,2-dialkylation of α-substituted acrylates via ireland–claisen rearrangement. *Chem. Sci.*, **12**, 2816-2822 (2021).

[2]. Zhao Y. C., Chan, P. W. H, et al. Copper catalyzed *N*-formylation of α-silyl-substituted tertiary *N*-alkylamines by air. *Green Chem.*, **22**, 5296-5302 (2020).
